# The importance of information acquisition to settlement services literacy for humanitarian migrants in Australia

**DOI:** 10.1371/journal.pone.0280041

**Published:** 2023-01-06

**Authors:** Julianne Abood, Julie Green, Michael J. Polonsky, Kerry Woodward, Zulfan Tadjoeddin, Andre M. N. Renzaho

**Affiliations:** 1 Translational Health Research Institute, Western Sydney University, Campbelltown, New South Wales, Australia; 2 Murdoch Children’s Research Institute, Parkville, Victoria, Australia; 3 Department of Paediatrics, University of Melbourne, Parkville, Victoria, Australia; 4 School of Social Sciences, Western Sydney University, Penrith, New South Wales, Australia; 5 Deakin Business School, Deakin University, Burwood, Victoria, Australia; 6 Centre for Sustainable Communities, University of Canberra, Bruce, Australian Capital Territory, Australia; 7 Maternal, Child and Adolescent Health Program, Burnet Institute, Melbourne, Victoria, Australia; Drexel University, UNITED STATES

## Abstract

**Background:**

Due to the diversity and range of services provided to humanitarian migrants during the settlement phase of migration, acquiring information across multiple service domains is intrinsic to the effective utilisation of settlement services. There are research gaps investigating how humanitarian migrants experience and navigate unfamiliar, multiple, and often complex information and service systems of host countries. This study seeks to understand the impediments to humanitarian migrants’ effective utilisation of information about settlement services and to identify strategies that can be implemented to overcome these barriers.

**Methods:**

Service providers were purposively recruited from organisations funded by the Australian Government to deliver settlement programs. The study applied an inductive thematic analysis approach to identify key themes that emerged from the data.

**Results:**

From the perspective of service providers, the themed findings identified how humanitarian migrants gain knowledge about services, their information needs, information seeking practices and skills, and information specific to service domains. The findings illustrate the importance of acquiring information, knowledge, and skills across multiple information platforms and service domains as being integral to the effective utilisation of settlement services for humanitarian migrants. The study identifies systemic barriers to information and service access and suggests different strategies and approaches to improve access to context specific key information. The study identifies factors that inhibit the effectiveness of the Australian settlement service provision model and emphasises the need for targeted training of mainstream referral services. The study highlights the important role that settlement service providers play as mediators of information, adept at tailoring information to humanitarian migrants’ individual and community information needs.

**Conclusion:**

The findings provide important insights that highlight the different roles that policymakers, researchers, and service providers can play to inform new approaches that improve the effectiveness of information and settlement service provision, as part of contributing to optimum settlement outcomes for humanitarian migrants.

## Introduction

Historically, major global migration and displacement events are triggered by conflict within and between countries, severe economic and political instability, and climate related disasters. In 2020, the United Nations reported that there were 26.4 million refugees globally, the highest number on record [1]. In addition, 4.1 million people were seeking international protection and awaiting determination of their refugee status (asylum seekers), a 45% drop from 2019 due to COVID-19 mobility restrictions [1]. The United Nations High Commissioner for Refugees (UNHCR) is mandated to aid and protect refugees and asylum seekers, and to assist with their voluntary repatriation, local integration, or permanent resettlement in a third country [2]. Resettlement continues to be the main, and often, only durable solution offering protection to those refugees most at risk [3]. In 2020, the UN reported that approximately 34,000 refugees were admitted for resettlement globally, a large decrease from the 107,700 refugees resettled in 2019 [1]. This sharp fall in the number of refugees resettled reflects the effects of the ongoing COVID-19 pandemic, host countries decreasing the number of refugee admissions due to economic constraints and/or tightening security screening for refugees from ‘high-risk’ countries [1]. However, the number of refugees in need of permanent resettlement continues to increase each year. In response to this dilemma, the UNHCR and International Organisation for Migration (IOM) launched the ‘Sustainable Resettlement and Complementary Pathways Initiative’ (CRISP) 2020–2022, to support resettlement host countries to expand the scope and size of resettlement programmes, and ‘enhance their protection impact and quality’ [4] (p:18).

Host countries commonly provide a range of specialist services for humanitarian migrants (persons granted refugee status or another form of protection) to assist with their resettlement and integration. Settlement programs are an important part of host country’s migration policies to ensure humanitarian migrants adjust to the new environment and achieve full social and economic participation in their new society. Humanitarian migrants are considered to be most in need of settlement services due to the negative effects of their forced migration, experiences of war and persecution, torture and trauma, loss of family and friends, and physical and mental health issues [5]. The content of settlement programs is guided by the UN ‘Global Compact on Refugees’, a framework to assist host countries to develop a multi-stakeholder approach for providing services to facilitate integration, such as cultural orientation, language and vocational training, as well as programs to promote access to education and employment [2,6].

The Australian Government funds four classifications of settlement services for humanitarian migrants: pre-arrival, on-arrival, post-arrival, and English language. This study focuses on the provision of the on-arrival ‘Humanitarian Settlement Program’ (HSP) and post-arrival ‘Settlement Engagement and Transition Support’ (SETS) program, available to humanitarian migrants for the first five years after arrival. Both programs are delivered to clients using a case-management, needs-based approach. The HSP program provides intensive foundational support for humanitarian migrants in the first 6–18 months from arrival [7]. The SETS program is designed to improve social participation, economic wellbeing, independence, personal wellbeing, and community connectedness [8]. Targeted initiatives and supports provided through the HSP and SETS programs are guided by the Australian National Settlement Framework’s nine priority focus areas (language services, employment, education and training, housing, health and well-being, transport, civic participation, family and social support, and justice) [9]. These programs are complemented by access to a broad range of mainstream social and human services, such as welfare, education, employment, and health services which are available to all Australians [10].

To achieve key settlement outcomes in all nine of the focus areas, it is necessary for humanitarian migrants to access, understand, and apply different sources of information across multiple service domains. The process of resettlement in a new country is a complex experience and is marked by the need to access new information relating to the social, cultural, economic, and political conditions of a new context, and to develop new information practices to engage with information through a variety of providers and formats [11,12]. Knowing how to access, use, and communicate information effectively is critical to the integration and social inclusion of new migrants [13,14].

Humanitarian migrants’ information needs differ from other migrants due to pre-existing vulnerabilities and disadvantages related to their forced migration [11,15]. For example, limited host country language proficiency acts as a barrier to seeking, accessing, and using available services and information, and has consistently been identified as negatively impacting all aspects of resettlement [16–20]. Humanitarian migrants’ experiences of fractured family and social networks negatively impacts on their access to information, especially in the initial phase of migration as they establish networks and acquire the language of the host country [20–22]. The negative effect of poor host language proficiency is compounded for humanitarian migrants who may not have strong literacy skills in their home language, have resided in refugee camps for extended periods of time, have little or no formal education, or have limited prior experience using modern technologies and information platforms [23,24]. In addition, the quality of available information post-migration is often inadequate to meet the information needs of humanitarian migrants, being culturally and linguistically inaccessible or inappropriate, or is overly reliant on digital literacy [25,26].

Settlement service (SS) providers are generally the first point of contact for humanitarian migrants when they arrive in Australia, and the support they offer has a significant influence on humanitarian migrants’ future settlement outcomes [27]. Abood et al., explored the provision of SS in Australia through the lens of literacy, using Masinda’s ‘settlement services literacy’ (SSL) conceptual framework [21]. Basic SSL refers to the set of skills enabling individuals and communities to effectively access information and gain knowledge to effectively navigate and utilise SS [28]. SS providers reported that humanitarian migrants’ utilisation of services and supports, or basic SSL, was impeded due to a range of barriers related to information access, such as, lack of knowledge about available services, being unfamiliar with the types of services available, lack of translated information, complexity of information and service systems, the need to navigate multiple service systems with different service models, and the lack of capacity of mainstream services to cater for their diverse and often complex needs [21]. However, that study did not investigate the importance of information acquisition to achieving basic SSL competency, the complex range of information and information sources, or other types of literacy necessary for humanitarian migrants’ effective SS utilisation.

### Aims

There remains a research gap when investigating how humanitarian migrants experience and navigate unfamiliar, multiple, and often complex information and settlement service systems of host countries [29]. Basic SSL requires that individuals have the capability to access a broad range of information and services in a variety of formats and contexts. From the perspective of SS providers, this study aimed to expand and elaborate on the concept of basic SSL by identifying factors related to information acquisition that either enable or constrain humanitarian migrants’ effective utilisation of available SS. This study investigates the importance of acquiring information and identifies the range of knowledge and skills necessary for humanitarian migrants to effectively navigate, access, and utilise multiple, often complex, information and service systems of the new environment.

### Concepts of literacy

The concept of literacy has moved beyond the simple notion of the technical skills required for reading, writing and arithmetic (‘three Rs’), to a plural notion encompassing multiple forms of literacy related to both individual and social contexts. During the 1960 - 70s the concept of ‘functional literacy’ emerged as the notion of literacy was first expanded and linked with socio-economic development [30] (p:9). This concept was further developed in the 1980 – 90s as a ‘lifelong learning’ journey of social practice contributing to citizenship, cultural identity, socio-economic development, human rights, and equity [30] (p10). For Scribner, the usefulness of ‘dissecting literacy’ into its many forms encourages the development of new systems of literacy that recognise the ‘complex social and psychological factors sustaining aspirations for and achievements of individual literacy’ [31] (p:8–9).

In 1994, the term ‘multiliteracies’ was coined by the New London Group, convened to consider literacy in relation to rapidly changing modes of communication and information [32]. The concept of multiliteracies recognises the multiple domains in which an individual operates and provides an understanding of how multiple ways of knowing are produced through the different domains in which an individual is situated [33,34]. The multiliteracies framework is based on the notion that knowledge and meaning are historically and socially located and produced, and the complexity and interrelationship of different modes of meaning are intrinsically related to language literacy [35]. This plural and multi-dimensional understanding of literacy encompasses many different contexts and genres of physical, social, cultural, and political realities of individuals and communities, that are constantly evolving to adapt to new technologies and new circumstances [36]. In 2004, UNESCO formulated a universal operational definition as:

‘Literacy is the ability to identify, understand, interpret, create, communicate and compute, using printed and written materials associated with varying contexts. Literacy involves a continuum of learning in enabling individuals to achieve their goals, to develop their knowledge and potential, and to participate fully in their community and wider society.’ [30] (p:13)

It is important to acknowledge that both verbal (spoken language) and nonverbal (written language) communication is inextricably linked to attaining other literacies [37]. Thus, acquiring different types of literacy is dependent upon the level of literacy, numeracy, and language competence of individuals and is context and setting specific [38].

#### Information literacy

Information literacy is an extension of the notion of functional literacy, and is commonly defined as the capacity to locate, evaluate, and use information to create new knowledge, and is considered a core adult life-long learning skill [39]. The acquisition of knowledge, and its use, is inextricably linked with information literacy skills, and is a prerequisite to achieving other literacies [40]. Information literacy is considered as a socio-cultural practice of activities including both competency and skills-based literacy that enable effective engagement with an information landscape [34]. The concept of an information landscape is a principal element of the concept of information literacy used to describe the broad contexts or settings of knowledge domains of a social site (information environment) [11,12,34,41,42]. Like the varied nature of physical landscapes, different information landscapes require the development of information practices that will allow individuals to map its resources, and skills to make the information and knowledge within them accessible [34]. Therefore, information practice is informed by knowing about the sources of information, and being able to locate, evaluate, and effectively use information for any given need [34].

Like the concept of multiliteracies, information literacy represents multiple knowledges and literacies of information (digital, visual, data, critical, health, etc.) that are context specific and dependent upon ‘complex networks of people’s interactions with analogue, digital, corporeal, and social information sources’ [11] (p:5). Limited access to information and associated information skills, or ‘information poverty’, creates a cycle of alienation that restricts the capacity of individuals to fully participate in society, to make informed decisions, and over time, can affect individual’s ability to extend social networks, to gain employment, maintain health, and improve their education [13] (p:125). This cycle can be further exacerbated for individuals with low levels of formal education and/or poor (home and/or host) language proficiency [29].

#### Digital literacy

In today’s digital era, digital literacy is inextricably linked to information literacy. Digital literacy refers to a core set of competencies that reflect a combination of knowledge and skills required for accessing and navigating digital sources of information and online services [43,44]. Digital literacy is critical to the successful resettlement and integration of humanitarian migrants, and is inextricably linked to social inclusion and sense of wellbeing [43,45,46]. A study exploring new migrants’ use of the internet, found that 92% of participants used the internet to communicate with friends and family in their homeland countries and in Australia, noting that those who lacked this had ‘digital poverty’ which lead to significant social and economic exclusion [43] (p.13). Humanitarian migrants often lack the digital literacy skills necessary to competently navigate online service platforms and understand the reliability of internet resources [11]. Many humanitarian migrants face significant challenges in navigating websites, and online systems and portals required to access key government services, language support, accommodation, employment, educational information, transport, banking and financial services, and healthcare information [46,47]. This ‘digital divide’ is due to poor digital literacy, lack of experience using online technologies, and extensive reliance on mobile phones–these factors are further compounded by low English proficiency [48] (p:17).

#### Settlement services literacy

Information literacy and digital literacy are core components of SSL and to attaining other types of literacy, such as health, employment, and education, that are in turn vital to achieving key social and economic settlement outcomes [28]. The evolution of the concept of literacy has contributed to understanding the broader social context associated with the information seeking practices of new migrants [28]. First coined by Masinda in 2014, the conceptual framework of SSL refers to new migrants’ ability to access available services and information to assist with their resettlement and integration in the new environment [28]. For Masinda, SSL is an ‘ongoing process of competencies enrichment enabling immigrants to know, understand, access, critically navigate immigrant services and gain political skills to effectively mobilize the mainstream society so that immigrant services are part of the political agenda’ [28] (p:5). Due to the diversity and range of SS provided to humanitarian migrants, representing multiple service domains and information landscapes, acquiring knowledge through multiple information sources is intrinsic to achieving basic SSL competency. Therefore, it is necessary to incorporate an investigation of the concepts of multiliteracies, information literacy, and digital literacy to our understanding of basic SSL.

## Methods

### Study design and setting

This qualitative study was part of a mixed methods research project investigating settlement services literacy among new migrants in two Australian states, New South Wales (NSW) and Victoria (VIC). The study was conducted in Greater Western Sydney, NSW, and Greater Melbourne, VIC, both recognised as ethnically diverse regions and prominent multicultural hubs in Australia [49].

### Study participants and sampling

Settlement service providers were purposively recruited from organisations funded by the Australian Government to deliver HSP and/or SETS programs in the study locations. Organisations were invited to participate in the research study and to nominate an appropriate representative to take part in the interviews. Participants represented a range of roles working at different levels within their organisations, including roles in senior management (CEO, general manager, executive manager, research and policy manager); program management (program manager, team leader, senior project officer); and client services (case manager, settlement support worker, caseworker).

The perspective of SS providers is most appropriate for this study as they are specialist workers actively involved in the provision of HSP and/or SETS programs, have oversight of the practical application of the SS provision model, are firsthand witnesses to the experiences and complexity of issues impacting different cohorts of humanitarian migrants, and have well established networks with the community and key stakeholders.

### Data collection

This qualitative research study applied a grounded theory approach to data collection to inductively build a hypothesis, model, or theory from data ‘grounded’ in reality [50]. Qualitative research methodologies enable patterns in the data to emerge, helping to explain and justify the research question [51,52]. Interviews with service providers were guided by themed questions relating to the nine priority outcome areas outlined in the National Settlement Framework [9]. A semi-structured interview guide was used to investigate: the types of settlement services provided; the service provision model, program effectiveness, barriers, and enablers to services; the key challenges for humanitarian migrants during settlement; the range of formal and informal supports available; and suggested solutions for more effective service delivery and settlement outcomes for humanitarian migrants (see S1 File). These themes were explored using in-depth interviews to allow participants to provide information about their insights and perspectives related to program delivery and the experiences of humanitarian migrants.

After ethics approval (Western Sydney University Human Ethics Committee: H13063) was received, the interviews were conducted by two researchers, one in each state. Informed written consent was obtained from all participants for inclusion in the study. In-depth qualitative interviews with 26 service providers (18 women; 8 men) were undertaken, representing 19 organisations in Greater Western Sydney (n = 8), and Greater Melbourne (n = 11) between October 2019 and January 2020. Participating organisations were community-based, not-for-profit; migrant specific (n = 11), mainstream (n = 4), diversity (n = 2), and faith-based (n = 2) services (see S2 File). Interviews took between 45 and 120 minutes. All interviews were audio-recorded and professionally transcribed to ensure preservation of participant comments and accuracy of raw data.

### Data analysis

Data were analysed, coded, and managed with the assistance of NVivo 12 software. An inductive thematic analysis approach was applied to data analysis to allow for flexibility when identifying, analysing, and reporting themes and patterns within the data as they emerged [53]. Braun and Clarke’s six-step thematic analysis approach to coding was applied to systematically analyse the data [53]. First, each transcript was read and re-read to become familiar with the content and to identify information relevant to the research questions. Second, initial ideas for coding were identified as relevant topical categories reflective of the content of the data. As part of this process, ideas for coding and theme development were discussed in collaboration with the authors to guide the coding process, to promote transparency, and to identify and address any issues of positionality and reflexivity. Third, codes were collated and grouped into themes and subthemes as they emerged to provide an aggregated interpretation of the data. The fourth step involved the development of a ‘thematic map’ to review the relationships among the codes, themes, and subthemes of the data set. The fifth step involved a process of defining and naming the main themes and subthemes. Finally, narration of the findings was undertaken, using extracts from the data to provide evidence that supported the merit and validity of the analysis. The coding process occurred inductively without the use of a theory or conceptual framework.

## Results

The analysis of our findings identified four key themes. These were: (1) knowledge about services; (2) information needs; (3) information seeking practices and skills; and (4) information specific to service domains (i) education and training; (ii) employment; (iii) financial; (iv) health and wellbeing; (v) housing; (vi) transport; and (vii) legal. The themed findings identify barriers to information and interrelationships between service domains.

### Knowledge about services

SS providers in both states played a key role in actively providing information to humanitarian migrants about available SS, how to use, access, and navigate them. As a key component of the HSP and SETS program referral model, SS caseworkers routinely referred humanitarian migrants and their families to a range of specialist and mainstream services as needed. SS providers worked in partnership with a wide range of key stakeholders, (i.e., health services, education and training, employment, disability, welfare, and legal services) to deliver specialist information and education sessions outside of their expertise, remit, or funding capacity. SS providers acknowledged that understanding and navigating Australian service systems was often very challenging for humanitarian migrants, especially when humanitarian migrants were forced to navigate multiple service domains simultaneously. Some issues highlighted were:

‘… the service delivery models aren’t consistent with what people, how people are going to engage. And two, people’s understanding or service literacy is so low it precludes them interacting effectively with those services.’ (Senior manager, SP02VIC)

‘Look the mainstream organisations are very difficult to access … The cultural competency of their frontline staff could also be issue for access. The location of where these organisations are located. Is it accessible by train or bus … The complexity is baffling…’ (Senior manager, SP04NSW)

SS providers identified that humanitarian migrants had a general lack of knowledge or familiarity with the types of services available within service domains, what they were entitled to, and the eligibility criteria. SS providers needed to explain program specific concepts, such as home care or aged care packages, and provide information about the types of services available and what they provided. SS providers supported humanitarian migrants to navigate multiple service providers simultaneously, assisting with communication, and making appointments. One SS provider noted the need to develop resources that outlined available services, service pathways, and eligibility to simplify and improve information dissemination to humanitarian migrants. SS providers identified that providing information about services needed to be done in a sustained fashion and not as a one-off occurrence.

‘… knowing about what services are available if you’re not aware of what’s available, such as settlement services. Then you’re going to take a lot longer to settle, because you’re not going to know about legal services or whatever else is out there … because people aren’t accessing the services, because they don’t know about them, or they’re forgetting. It needs to be drilled in; you can access these services for up to five years.’ (Client services, SP04VIC)

### Information needs

Pre-migration and displacement experiences, country of origin, culture and traditions, age, gender, and mental health were identified by SS providers as the main factors determining individual humanitarian migrants’ information needs. Examples offered noted that:

‘Someone who’s got mental issues, post-traumatic stress or just depression, their ability to retain information is going to be a challenge. They’ll learn it today; they’ll forget it tomorrow. So, repetition is pretty essential because they just can’t concentrate.’ (Program manager, SP05VIC)

‘The Syrians … they speak four languages, they’re highly educated, they don’t actually need much of our support, they just need to be steered in the right direction … So, it’s a very different problem to the say someone coming from the Karen community who’s been born in a refugee camp, who’s only had school within a refugee camp … it’s their needs and settlement experience is quite different to someone … to another group so you can’t really generalise.’ (Program manager, SP06VIC)

Poor English language proficiency was considered by SS providers as the main barrier for humanitarian migrants when attempting to access information and services. SS providers regularly assisted humanitarian migrants by explaining different terminologies, reading letters, and completing forms. One SS provider shared their perspective about the importance of knowing the information needs of communities:

‘You need to understand if, you know, the literacy level of the community you’re working with in terms of their own language. There’s no point giving them something in their language, which is great, if they can’t understand it. So, ensuring that the information is in an accessible format to the community is really, really important.’ (Senior manager, SP08VIC)

SS providers noted the specific barriers to information for older women who were illiterate in their home language and had no prior experience of learning in a classroom environment:

‘Older ladies that are 50, 60, 70 and they are busting to get out of the house. They’re socially isolated, we are challenging them with a whole bunch of different concepts … They’re hungry for information, but they’re scared of the formal… they just feel they’re too old for formal schooling. The formality of schooling, they just go, I’m too old.’ (Program manager, SP01VIC)

SS providers pointed out that the ‘bombardment’ of new information being provided to humanitarian migrants in the early stages of settlement caused information overload, resulting in many humanitarian migrants to ‘drop off’, feel overwhelmed, and not able to retain the information. SS providers shared their insights regarding humanitarian migrants’ information needs:

‘So, what our experience is, when the refugee … arrives in Australia, there is information overload. Everybody wants to do everything with them … every information is being provided to them. So, there is only so much human brain can take.’ (Senior manager, SP04NSW)

‘Information sticks when it’s relevant to that point in your life.’ (Program manager, SP01VIC)

### Information seeking practices and skills

SS providers observed that humanitarian migrants’ information seeking practices in the early stages of migration were mainly reliant on family and their established community networks for access to culturally and linguistically accessible sources of information. Information was usually delivered through these networks in the humanitarian migrant’s home language and via ‘word of mouth’. SS providers noted that this information sharing practice was a significant contributor to humanitarian migrants’ acquisition of information. SS providers often worked in partnership with these community networks to promote and distribute important information. Barriers to providing information and communicating with humanitarian migrants were found to be mainly due to the lack of ‘language support’ available to SS providers. SS providers stated that the lack of access to free interpreting and translation services, limited translated resources (hardcopy and online), and limited access to bicultural workers, negatively impacted the quality of information and services that organisations provided, as well as the settlement experience and settlement outcomes of humanitarian migrants.

SS providers identified that it was essential for humanitarian migrants to be ‘computer literate’ to navigate the online websites and portals of organisations to access multiple key services and sources of information. SS providers recognised that humanitarian migrants’ language proficiency was a key factor to accessing online information and services, especially when online information was available only in English. SS providers found that different cohorts of humanitarian migrants had varying skills when accessing digital information and services, noting that some humanitarian migrants had no prior experience using computers and the internet. SS providers actively supported the development of digital skills for humanitarian migrants by providing access to computer courses and assistance with online applications and website navigation:

‘Language is an issue, and also now days, technology. A client who applies for citizenship, they have to go and pay online, and it is a big challenge. So, they have to come to us in order to do it online, because they have no idea about the terms used in there, for example, e-Me account…So sometimes this procedure takes hours and hours.’ (Client services, SP08NSW)

‘Every organisation have [*sic*] their website … you have to navigate the whole online portal which is extremely difficult … if you’re digitally literate … you’ve had that access to information. And then you can make a right choice about services.’ (Senior manager, SP04NSW)

### Information specific to service domains

**Fig 1:**
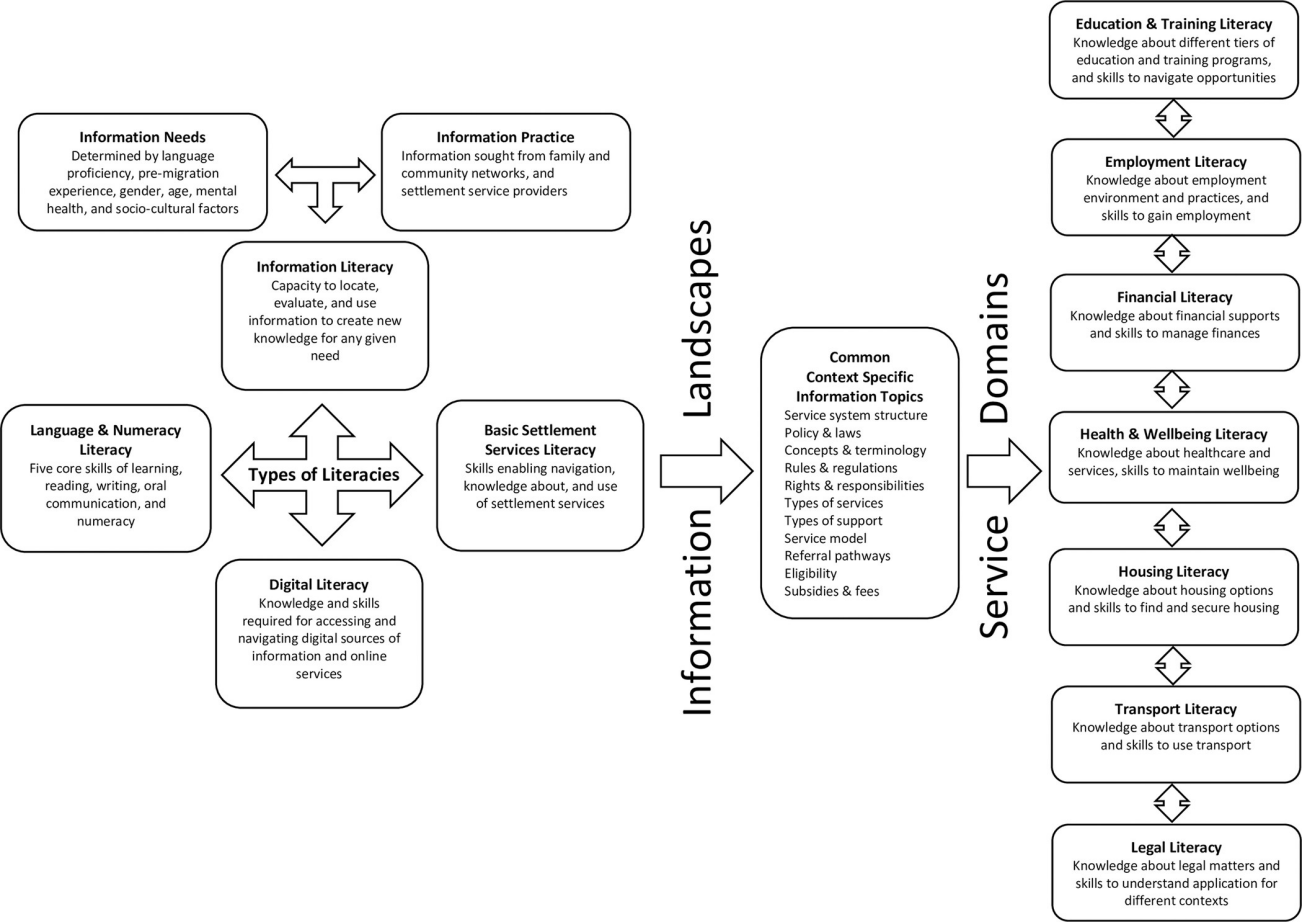
Interrelationships between the acquisition of information and different types of literacies (multiliteracies) necessary for basic settlement services literacy.

As integral to the SS provision model, SS providers in both states routinely referred humanitarian migrants to key mainstream services, such as education and training, employment, financial, health and wellbeing, housing, transport, and legal services. SS providers identified that each service domain’s information landscape comprised of specific knowledge and skill requirements (or literacies) necessary for humanitarian migrants to effectively navigate the services within each of these domains. A range of common context specific information topics were identified as relevant to all service domains (see Fig 1).

#### (i) Education and training

SS providers delivered a broad range of information to humanitarian migrants to build their knowledge and understanding about the Australian education system and the different tiers of the education system (early childhood, primary, secondary, and tertiary), the types of education services and opportunities available, and to assist humanitarian migrants with the application and enrolment process. Information provided to humanitarian migrants included assessment of what education faculty or opportunity would be most appropriate for humanitarian migrants’ interests, skillset, and future job prospects, the costs involved, eligibility, how to access fee support, and to identify potential scholarships. Examples of education and training information needs of humanitarian migrants were reported as:

‘In terms of higher education, yes, not knowing where to go and what’s most appropriate for their skills and is most likely to get them a job, that’s what we do a lot of, so, it’s not so much that they’re limited or unable to access higher education, it’s just knowing where to go.’ (Client services, SP09VIC)

‘For education around, early education in providing access to information is critical, and also not only once, but over a sustained period so that people have it ingrained, and they have the understanding they can access the opportunity at any time.’ (Program manager, SP07NSW)

Barriers to accessing relevant key information about education and training opportunities was described by SS providers as creating obstacles for humanitarian migrants as they struggled to navigate and utilise available services and programs. One SS provider offered an example outlining the complexity of information important for humanitarian migrants when considering enrolment at a university, that is, eligibility for fee loan schemes such as HECS (Higher Education Contribution Scheme) requires Australian citizenship, and humanitarian migrants need to be in Australia for four years before they can apply for citizenship, then the process can take 18 months. Another SS provider raised the point that education services needed to be more literate regarding the information needs of humanitarian migrants:

‘I don’t think schools are educated enough about specifically newly arrived and refugees, about how to work with them, and how to get them engaged … So, I think that’s about the literacy … from the school’s perspective, that they need to become more literate about settlement and how to work with them and what services should be offered through the schools.’ (Client services, SP04VIC)

#### (ii) Employment

The SS providers played an important role in assisting humanitarian migrants with gaining literacy around Australian employment systems and concepts. Pre-employment programs provided information to humanitarian migrants outlining Australian employment obligations and laws, taxation, superannuation, workers compensation, how to start a business in Australia, and workplace personal health and safety requirements. In partnership with TAFE (Technical and Further Education), SS providers regularly held vocational courses and training, as well as field trips to workplaces to provide humanitarian migrants with practical insight into the kinds of work available. SS providers assisted humanitarian migrants to develop the knowledge and skills needed to gain employment such as writing resumes, how to address the job criteria, job interviews, how to search and apply for jobs online, how to attach their resume online, as well as ‘soft skills’ such as understanding Australian workplace culture.

SS providers identified barriers to gaining employment for humanitarian migrants as further compounded by the lack of recognition of prior qualifications, not having local experience, and having no local employment history or local referees. Hence, employment literacy was found to be closely interrelated with aspects of education literacy. The complexity of bureaucratic systems and ‘red tape’ to access information regarding pathways to gaining recognition of prior qualifications and work experience was stated by SS providers as ‘rigorous’, ‘quite arduous’ and acted as additional ‘stressors’ for humanitarian migrants.

SS providers in both states agreed that the system around employment support was not working for humanitarian migrants. The Australian job network providers funded to assist humanitarian migrants to find employment were regularly criticised by SS providers for their lack of effectiveness, lack of cultural and linguistic competency, and lack of understanding of humanitarian migrants’ information and digital literacy needs. SS providers identified multiple barriers to these employment services that negatively impacted humanitarian migrants’ service utilisation and experience:

‘Certainly, job network providers, very difficult. It’s a distress for our clients. You know, they’re required or asked to commit to appointments, to look for work online. Many of my clients really need help with that, they haven’t got the confidence … You know, trying to navigate Australian concepts and systems. Very, very difficult … Especially with job active networks and if you don’t you’ll receive a letter from Centrelink saying your payments could be potentially cut off or you’re going to have to reapply, these are the requirements.’ (Client services, SP07VIC)

‘For example, client of mine, they say we are asked to go and look for a job. They’ve never touched a computer before, so they say, I sit in front of the computer with a blank mind for one hour … I’ve no idea how to look for a job. So she says I go and sit there for one hour and then I leave, so I can sign that I’ve been there.’ (Client services, SP08NSW)

#### (iii) Financial

SS providers commonly tailored financial literacy programs to include a range of information about available financial supports and subsidies, welfare payment obligations, the Australian banking system, how to apply for a concession card, budgeting for utility costs and paying bills, energy use management (for electricity in particular), paying fines, no-interest loans, available emergency relief and vouchers (food, electricity, gas, water), and understanding internet and phone contracts. Financial literacy programs included digital literacy skills to assist humanitarian migrants to navigate internet banking and online service systems as SS providers acknowledged that many government and social support services were only accessible online:

‘… we run IT and computer classes. So, in those, for example, we teach them how to navigate the websites, how to improve their typing literacy, computer literacy … nowadays I mean lots of things are taking place online. So how to check your inbox, how to open an account. So, all these things in general in a way that contribute to their financial literacy.’ (Program manager, SP10VIC)

Financial literacy was found to be closely interrelated to employment, housing and education literacies as SS providers reported that many of humanitarian migrants were dependent on welfare payments as their main source of income in the early stages of settlement. SS providers considered money management skills as essential as many of humanitarian migrants were struggling to pay their bills and other essential living expenses. One SS provider noted that ‘some clients were paying almost 60% of their income in rent’ (Senior manager, SP12VIC). Other SS providers pointed out that:

‘… the unemployment rates in these cohorts is 90%. And if you are going to be on Centrelink (Government welfare service) income you’re going to live in poverty.’ (Senior manager, SP02VIC)

‘… also they’re trying to manage, work is a priority for them, housing’s a priority, financial stability, education is for their children certainly, and stability. That’s very problematic for that community.’ (Client services, SP07VIC)

SS providers tailored financial literacy programs to respond to multiple socio-cultural and pre-migration factors of humanitarian migrants. Financial management programs were commonly targeted to women as SS providers acknowledged the cultural issues resulting from welfare payments being made directly to women:

‘… communities … still have very strong patriarchal value sets. So, you get women with really low levels of financial capability … never really having had to budget. So, if you want you could buy something. You know, buy it on hire purchase, buy it, rent to buy. You know, and then realising that the $600 appliances are going to cost you three and a half thousand dollars by the time you’re finished paying for it. That stuff, which are unfamiliar constructs for people are problematic.’ (Senior manager, SP02VIC)

‘There is also issues around learning banking. Banking back home wasn’t an issue for some of the communities who are pastoralists or who are more into agriculture, and so storing money in the bank was not a need back home. Or even managing money was not a need, they managed other livestock, not money … There’s a lot of cultural issues that impact on financial literacy.’ (Program manager, SP07NSW)

Financial counselling was provided to humanitarian migrants facing financial strain as unexpected expenses (such as a serious illness or death in the family or needing to buy a car) or cultural and family obligations arose (such as sending money home to family, dowries, and family reunion applications). Humanitarian migrants were made aware of the risks of using money lenders or ‘loan sharks’ and to signing contracts:

‘… to educate them not to sign for those energy companies, they ask them to come and sign up, and they end up receiving bills from different companies, and they are worried.’ (Client services, SP08NSW)

#### (iv) Health and wellbeing

SS providers commonly referred humanitarian migrants to a wide range of health and wellbeing services. SS providers offered a variety of health-related information to humanitarian migrants via information sessions and workshops to help familiarise humanitarian migrants with the Australian health system, the types of services available, eligibility, referral processes, costs and subsidies, bulk billing, health insurance and Medicare (Australian healthcare benefits), and how to access specialist and disability services. In partnership with health service providers, programs were commonly tailored to meet the needs of specific ethnic communities or different groups in the community to promote health literacy around cancer prevention, immunisation, Hepatitis, stress management, healthy foods, maintaining heart health, and diabetes. SS providers regularly held programs to promote humanitarian migrant’s engagement with services and knowledge around physical, mental, and sexual health, parenting, and healthy relationships.

‘Health and doing, being able to identify health issues, mental health, physical health issues are a key component and then being able to facilitate access … we don’t provide the services, but we would act as a conduit between the existing services and all of those, you know, around primary health and mental health supports for people.’ (Senior manager, SP02VIC)

Barriers to accessing health service information were found to be compounded by a range of cultural issues, language literacy, and factors related to humanitarian migrants’ employment and financial literacy:

‘… like imagine a refugee with very low literacy. So, there are lots of misunderstandings … because they have low income, so they want to know which services are free for them and then how can they use them and where to go, how to contact. So, they finally contact, then they have a language problem, so there’s no proper interpreting access for them or understanding … some refugees are coming from a patriarchal community where men are the one who working and then maybe women, they may not have the green light from the husband to go out alone or they may have to, because of language they have to accompany a child to go to a service.’ (Program manager, SP10VIC)

#### (v) Housing

To improve humanitarian migrants’ service literacy related to housing, SS providers promoted key information about rental bonds, tenancy agreements and contracts, and tenants’ rights and responsibilities:

‘Like for example people that are renting houses and have people living in garages, not knowing that is not a habitat approved room … And people running businesses from home like childcare and all that sort of thing is also a problem … And because of the problems of not understanding the contractual obligations, sometimes properties get damaged, or they don’t get used according to the contract.’ (Senior manager, SP02NSW)

SS providers noted that, as there was a long waiting list for community housing, humanitarian migrants were forced to look for accommodation in the commercial rental market. One SS provider identified numerous challenges that humanitarian migrants faced when accessing information about the commercial rental market:

‘And it’s really difficult if you haven’t had a rental history, you know, rent receipts, to obtain a bond loan, how do I navigate that? Even online, everything’s online … if you do not know the alphabet and you at a keyboard or a tablet, it’s not going to really make much sense to you.’ (Client services, SP07VIC)

Access to affordable housing was found to be reliant on factors related to employment and financial literacy, and interrelated to achieving other key settlement outcomes:

‘Housing affordability, understanding Centrelink, needing advocacy around Centrelink, being unclear about their, the support they can and can’t receive … where can you get concessions, financial resilience’ (Client services, SP09VIC)

‘… accommodation is very expensive, the rent is very expensive, and taking a lot from their income. Most of them, they want private school … it’s culture shock. They still find it hard. So, the school, private schools take a lot from the budget.’ (Client services, SP09NSW)

#### (vi) Transport

SS programs in both states provided key information related to transport literacy, such as how to use public transport (where to catch a bus, how to purchase and use an Opal (NSW) or Myki (VIC) public transport card), how to understand street signs, road rules and safety, child car seat restraints, concession cards, and a wide range of other related topics such as:

‘… to give information sessions around … navigating Australia’s concepts and systems … we have … driving programmes … But firstly, our clients have to attend five sessions … we bring in Vic Roads, insurance companies to talk about … the importance of insurance, fines, civic compliance, what that looks like, what your obligations are before …. they can actually participate in the driving programme. (Client services, SP07VIC)

‘… they don’t use their Opal card properly. Either they don’t carry their concession card, they receive fines, and sometimes they just trespass over the gate and they think it’s OK, and when they receive a $200 fine, they come to us.’ (Client services, SP08NSW)

#### (vii) Legal

Legal literacy of humanitarian migrants was supported by the delivery of legal orientation programs including information about Australian law and the justice system, migration law, child protection, family and civil law. SS providers promoted information about the types of legal services available and the eligibility criteria, such as matters for community legal centres vs private lawyers.

‘… like the refugees, they’ve got some legal issues and then their understanding and the literacy is very low… So, our case workers … review that case, they can contact other providers, or they can contact maybe a lawyer, maybe professional in this regards, to help them … the legal system also looks very complicated for them.’ (Program manager, SP10VIC)

‘What they are not aware is what services are there to represent them. Sometimes we get people coming to us and said I have to go to Court and said well you don’t have time, it’s tomorrow, you come today … So, I don’t think that there is a lot of awareness’ (Senior manager, SP02NSW)

Information sessions regarding traffic rules and fines, drink driving and penalties, and how to apply for citizenship were also commonly presented. SS providers facilitated referrals for humanitarian migrants and would often help complete forms. SS providers would often speak on behalf of humanitarian migrants for the initial appointment to ensure that the issues were understood, and an interpreter was requested for future appointments. Limited access to free legal services forced humanitarian migrants to seek private solicitors or migration agents. One SS provider noted the key barriers to using private agents as:

‘And one, it’s expensive. And two, there’s a lot of people that are real crooks. So, it slows down and complicates, you know, the process of getting family reunification, which then impedes and slows down the integration and settlement process.’ (Senior manager, SP02VIC)

## Discussion

Our findings illustrate the importance of information acquisition for humanitarian migrants as key to the effective access and utilisation of SS. Basic SSL was found to be more complex than just knowing about what services are available, how to access, navigate, and utilise services. This study identifies multiple types of literacy associated within basic SSL. In addition to language literacy, functional literacies, that is, information literacy and digital literacy, were found to be integral to achieving basic SSL competency. Additionally, the SS provision model encompasses multiple key service domains, such as, education and training, employment, financial, health and wellbeing, housing, transport, and legal services (see Fig 1). Therefore, for humanitarian migrants to effectively understand and utilise available information and services (basic SSL), it is necessary for SS providers to facilitate the development of knowledge and skills that are context specific to service domains.

As illustrated in Fig 1, common context specific topics of information were identified as being relevant to all service domains, such as knowing the structure, rules and regulations, types of services, referral pathways, and eligibility of each service system. SS providers reported that digital literacy skills were essential for humanitarian migrants to access online services and information across all service domains. Context specific skill requirements were identified as particular to service domains, such as the skills promoted to assist humanitarian migrants with finding employment (writing resumes and job applications, and interview preparation) or to access the online rental market. SS providers identified multiple barriers faced by humanitarian migrants when accessing information from mainstream referral services, including a general lack of cultural competency and a poor understanding of humanitarian migrants’ information and digital literacy needs. SS providers reported that both SS and mainstream referral services access to ‘language supports’, such as bicultural workers, interpreters, and translated information, was inadequate and a key barrier to effective communication and information dissemination to humanitarian migrants.

This study found that the language proficiency of humanitarian migrants was key to determining their ability to accessing information and impacted all aspects of their settlement experience and outcomes. Basic literacy skills of reading, writing and numeracy are indispensable for humanitarian migrants, particularly when acquiring knowledge and information associated with how to access different service domains [54]. Hence, strategies to promote information dissemination will remain inextricably linked to strategies that promote literacy, numeracy, and language skills. SS providers recognised the added challenges faced by humanitarian migrants who had limited or no literacy in their home language, who are experiencing pre and/or post migration stress or trauma, while learning to speak and read in a new language, and trying to develop the skills associated with navigating complex information landscapes and service systems of the new environment. However, research investigating the learning process for adult second language learners with limited literacy is sparse [55,56]. As Kalantzis and Cope point out, ‘education will not result in learning if the landscape is unseeable, unthinkable, incomprehensible, unintelligible, unachievable’ [35] (p:217). Research-based knowledge is needed to understand how to effectively teach this particular cohort of learners, and to offer new directions for further research that is presented in a way that allows information mediators or teachers to understand and transfer research findings into teaching approaches [55].

In addition to language proficiency, many humanitarian migrants’ information needs were found to be negatively impacted by a range of pre-migration, socio-cultural, and individual factors, such as mental health, gender, and age. SS providers were found to be effective in the assessment of humanitarian migrants’ individual and community information needs through the case-management and multi-stakeholder approach of the SS provision model. SS providers in both states worked in partnership with key stakeholders to effectively deliver service domain specific information to humanitarian migrants and to help build the knowledge and skills required to access available services and supports. SS providers applied strategies to meet identified information needs and practices of individual and communities, such as utilising ‘word of mouth’ networks for information dissemination or tailoring education programs to individual and socio-cultural needs.

Information dissemination will continue to be impeded unless key barriers to information are addressed, such as, improving access to language supports and the development of resources (online and hard copy) that simplify and collate key information specific to service domains. Addressing systemic access barriers to mainstream referral services is key to the success of a multi-stakeholder approach. A recent systematic review investigating SSL for new migrants in high income host countries found that systemic barriers relating to navigation and access to complex service systems, lack of translated information, poor access to interpreters, and culturally inappropriate services were common across included studies [26]. Mainstream referral services must be adequately trained and resourced as a requirement to deliver services that address the diverse and often complex needs of humanitarian migrants. Culturally proficient and responsive practice recognises the factors impeding access of services and develops strategies to promote specific workforce competencies of ‘knowledge, skills, and attitudes’ to remove barriers for all its clients [57]. In addition, more flexibility regarding eligibility to access SS programs beyond five years is needed to accommodate humanitarian migrants that are disadvantaged by pre- and post-migration factors that hinder their information acquisition and settlement progress.

As illustrated in Fig 1, the findings of this study identified multiple information landscapes, with context specific knowledge and skill requirements particular to each service domain. Therefore, the concept of multiliteracies can be applied to basic SSL, that is, the recognition of multiple or plural literacies inherent to basic SSL competency [58]. Well researched and documented types of literacy such as health [18,38,59–61] and financial [62,63] literacies, recognise the context specific knowledge and skill requirements important to humanitarian migrants to effectively engage with these service systems and information landscapes. In addition, this study found that the development of digital literacy skills was essential for humanitarian migrants to access key government, employment, housing, and financial information, services, and supports. Previous research has also found that both information literacy and digital literacy are interrelated to achieving and utilising services and supports made available to humanitarian migrants to assist them in achieving key settlement outcomes, such as employment, health, housing, education, and social integration [23,64–67]. Therefore, the identification of key service domain specific information, knowledge, and skills is essential to understanding and addressing the multi-dimensional complexity that humanitarian migrants face as they attempt to effectively utilise SS.

The findings of this study, and as illustrated in Fig 1, highlight the interrelationships between acquiring service domain specific information and to achieving other types of literacy as necessary for effective service utilisation and successful settlement. For example, this study found that financial literacy was closely interrelated with achieving digital literacy and other types of literacy, such as, employment, housing, and education. Good financial education can help humanitarian migrants become familiar with the new financial landscape, reduce poverty, and increase an individual’s, and their family’s financial security, standard of living, and overall settlement outcomes [63,68]. Thus, acquisition of service domain specific information, knowledge, and skills is crucial to basic SSL competency and to achieving other key settlement outcomes.

Finally, and most importantly, the findings of this study illustrate the critical role that SS providers play as mediators of information, or what Barton refers to as ‘guiding lights’ [69]. Literacy mediators provide a ‘bridge’ to information that may otherwise be denied or unduly difficult to access and create the context in which literacy activities are delivered [70]. The practice of literacy mediation by SS providers was found to be pivotal to the effective delivery of a broad and diverse range of important information to humanitarian migrants. Through case-management and consultation with key stakeholders, SS providers adeptly identified the information needs and practices of humanitarian migrants and their communities to enable the effective mediation, management, and delivery of essential information. SS providers endeavoured to deliver information in an accessible and culturally appropriate format that was sensitive to pre- and post-migration factors. SS providers applied both formal (reading letters, mediating communication, facilitating tailored information sessions and education programs with referral services and key stakeholders) and informal (promoting information via social networks) approaches to information dissemination, tailored to meet the information needs and practices of humanitarian migrants and their communities. Therefore, it is essential that SS providers’ practical insights are valued and that they are adequately trained, resourced, and supported to continue as mediators of information, as integral to the effective delivery of the SS provision model.

## Limitations

This research study has several limitations to note. First, the study obtained data from organisations funded to deliver the HSP and SETS programs, therefore, the findings do not represent the viewpoints of other SS providers across the study locations, although we did include a broad cross-section of SS providers in the study locations. Second, this study does not include an investigation of the needs of eligible migrants that have not accessed SS or ineligible migrants that cannot access these programs. Thus, further work examining new migrants’ experiences of SS is needed. Third, SS providers were asked to report on their perspectives of humanitarian migrants’ experiences of SS and were not explicitly asked to report about their competence in undertaking program delivery. Inclusion of humanitarian migrants’ perspectives would serve to provide a more balanced analysis of the factors impacting SS provision and identify potential bias. However, as SS providers are migrant specialist workers, who have established networks and relationships with community representatives and other key stakeholders, they are well positioned to understand the expectations and experiences of humanitarian migrants. Finally, SS providers were not explicitly asked to answer questions in relation to SSL, information literacy, or the concept of multiliteracies. Instead, responses were analysed inductively as corresponding to references made about skills and knowledge required to access services and information, and as related to service domains represented in the data.

## Future policy implications

Basic SSL competency for humanitarian migrants requires the implementation of a range of new strategies involving researchers, policymakers, service providers, and community members that are responsive to the local context, the target audience, and the Australian SS system environment. The application of the concept of multiliteracies to SS and information contexts ‘is useful to unpack the range of possible knowledge processes, to decide and justify what is appropriate for a subject or a learner, to track learner inputs and outputs, and to extend the pedagogical repertoires of teachers and the knowledge repertoires of learners’ [32] (p:187). Such an approach would enable an informed learning approach based on a curriculum design process that identifies key information, knowledge, and skill requirements that are specific to service domains and essential to the effective utilisation of SS [71]. Additionally, as mentioned above, this approach needs to be coupled by research investigating the learning process for adult second language learners with limited literacy, who are also disadvantaged by pre- or post-migration factors. The development of resources that presents key information in an accessible format and the formal engagement of community as information mediators would assist this process. Application of such approaches would allow policymakers to develop a more structured delivery of information that ensures key service domain specific information is delivered to humanitarian migrants in a more consistent and culturally and linguistically appropriate way that avoids ‘information overload’ and ‘information poverty’. The development of indicators and measures of key context specific information would allow SS providers to gauge humanitarian migrants’ progress, or basic SSL competency, and to provide targeted training based on individual needs.

Assisting humanitarian migrants to develop competence and confidence to source, utilise, and engage with multiple formats of information will empower individuals and communities to achieve key settlement outcomes, and overall wellbeing.

‘When individuals are able to augment their knowledge and understanding in terms of types of literacies and enhance their literacy skills, then they are able to promote welfare of their families and communities.’ [54] (p:1)

Empowerment of individuals is recognised as critical to ensuring services are culturally and linguistically responsive and appropriate to meet the diverse and often complex needs of new migrants accessing settlement services. Additionally, empowering humanitarian migrants in decision-making and encouraging the development of critical literacy skills has important implications for the scope, content, and delivery of information, services, and communication.

## Conclusion

The findings of this study highlight the importance of acquiring information, knowledge, and skills across multiple information landscapes and service domains as being integral to the effective utilisation of SS for humanitarian migrants. Our application of a literacy lens to a settlement service context provides an opportunity to identify and understand the complexity of essential information, the multiple types of literacies encompassed within basic SSL, and the interrelationships between service domains. The study highlights the important role that SS providers play as mediators of information, adept at tailoring information to humanitarian migrants’ individual and community linguistic, cultural, and pre- and post-migration information needs. The findings provide important insights relevant for policymakers, researchers, and service providers that can inform new approaches to improving the effectiveness of information dissemination and SS provision, as part of contributing to optimum settlement outcomes for humanitarian migrants.

## Supporting information

S1 File SSL research Study_Qualitative interview guide. (PDF)

S2 File Participant characteristics. (DOCX)

S1 Data (ZIP)

## References

[1] IOM. World Migration Report 2022 Geneva, Switzerland: International Organisation for Migration.; 2021 [1–540]. Available from: https://publications.iom.int/books/world-migration-report-2022.

[2] UNHCR. Resettlement. Geneva, Switzerland: United Nations High Commissioner for Refugees; 2020 [Available from: https://www.unhcr.org/resettlement.html.

[3] UNHCR. Projected global resettlement needs 2022. Geneva, Switzerland: United Nations High Commissioner for Refugees; 2021 [1–138]. Available from: https://www.unhcr.org/en-au/protection/resettlement/60d320a64/projected-global-resettlement-needs-2022-pdf.html.

[4] UNHCR. UNHCR Projected global resettlement needs 2021. Geneva, Switzerland: United Nations High Commissioner for Refugees; 2020 [1–142]. Available from: https://www.unhcr.org/protection/resettlement/5ef34bfb7/projected-global-resettlement-needs-2021-pdf.html.

[5] RCoA. Factsheet No.1 Liverpool, NSW: Refugee Council of Australia.; 2018 [Available from: https://www.swslhd.health.nsw.gov.au/refugee/pdf/Resource/FactSheet/FactSheet_01.pdf.

[6] UN. The global compact on refugees. New York, USA: United Nations 2018 [1–60]. Available from: https://www.unhcr.org/the-global-compact-on-refugees.html.

[7] Department of Home Affairs. Humanitarian Settlement Program (HSP) Canberra, Australia: Australian Government; 2020 [Available from: https://immi.homeaffairs.gov.au/settling-in-australia/humanitarian-settlement-program/about-the-program.

[8] Department of Home Affairs. Settlement Engagement and Transition Support (SETS) Program Canberra, Australia: Australian Government; 2020 [Available from: https://immi.homeaffairs.gov.au/settling-in-australia/sets-program.

[9] Department of Home Affairs. National Settlement Framework Canberra, Australia: Australian Commonwealth Government; 2016 [Available from: https://immi.homeaffairs.gov.au/settlement-services-subsite/files/the-national-settlement-framework.pdf.

[10] Settlement Services International. Joint Standing Committee on Migration inquiry into settlement outcomes Sydney, Australia: Settlement Servcies International.; 2017 [Submission 27:[Available from: https://www.aph.gov.au/Parliamentary_Business/Committees/Joint/Migration/settlementoutcomes/Submissions.

[11] Lloyd A. Shaping the contours of fractured landscapes: Extending the layering of an information perspective on refugee resettlement. Information Processing and Management. 2020;57:1–13. 10.1016/j.ipm.2019.102062.

[12] Lloyd A, Wilkinson J. Tapping into the information landscape: Refugee youth enactment of information literacy in everyday spaces. J Librarianship and Information Science. 2019;51(1):252–9. doi: 10.1177/0961000617709058

[13] Lloyd A, Anne Kennan M, Thompson KM, Qayyum A. Connecting with new information landscapes: Information literacy practices of refugees. J Documentation. 2013;69(1):121–44. 10.1108/00220411311295351.

[14] Oǧuz ES, Kurbanoǧlu S. Strengthening social inclusion in multicultural societies through information literacy. Bilgi Dunyasi 2013;14(2):270–90.

[15] Martzoukou K, Burnett S. Exploring the everyday life information needs and the socio-cultural adaptation barriers of Syrian refugees in Scotland. J Documentation. 2018;74(5):1104–32. 10.1108/jd-10-2017-0142.

[16] Blake HL, Bennetts Kneebone L, McLeod S. The impact of oral English proficiency on humanitarian migrants’ experiences of settling in Australia. Int J Bilingual Education and Bilingualism. 2019;22(6):689–705. 10.1080/13670050.2017.1294557.

[17] Pandey M, Maina RG, Amoyaw J, Li Y, Kamrul R, Michaels CR, et al. Impacts of English language proficiency on healthcare access, use, and outcomes among immigrants: A qualitative study. BMC Health Serv Res. 2021;21(741). doi: 10.1186/s12913-021-06750-4 34311712 PMC8314461

[18] Poureslami I, Rootman I, Doyle-Waters MM, Nimmon L, Fitzgerald JM. Health literacy, language, and ethnicity-related factors in newcomer asthma patients to Canada: A qualitative study. J Immigrant Minority Health. 2011;13(2):315–22. doi: 10.1007/s10903-010-9405-x 20938742

[19] Wali N, Georgeou N, Renzaho AMN. ‘Life is pulled back by such things’: Intersections between language acquisition, qualifications, employment and access to settlement services among migrants in Western Sydney. J Intercultural Studies. 2018;39(1):85–101. 10.1080/07256868.2017.1410114.

[20] Hebbani A, Obijiofor L, Bristed H. Intercultural communication challenges confronting female Sudanese former refugees in Australia. ARAS [Internet]. 2010; 31(1):[37–61 pp.]. Available from: https://www.researchgate.net/publication/48381306_Intercultural_Communication_Challenges_Confronting_Female_Sudanese_Former_Refugees_in_Australia.

[21] Abood J, Polonsky M, Woodward K, Green J, Tadjoeddin Z, Renzaho AMN. Understanding settlement services literacy and the provision of settlement services for humanitarian migrants in Australia—A service provider perspective. Australian Journal of Social Issues. 2022(00):1–22. doi: 10.1002/ajs4.204

[22] Allard D, Caidi N. Imagining Winnipeg: The translocal meaning making of Filipino migrants to Canada. J Association for Information Science and Technology. 2018;69(10):1193–204. doi: 10.1002/asi.24038

[23] Floyd A, Sakellariou D. Healthcare access for refugee women with limited literacy: layers of disadvantage. Int J Equity in Health. 2017;16(1):195. doi: 10.1186/s12939-017-0694-8 29126420 PMC5681803

[24] Chao X, Kang B. Health literacy among Bhutanese adult refugees in the United States: The sociocultural approach. Adult Education Quarterly. 2020;70(3):258–76. 10.1177/0741713620904047.

[25] Fatahi N, Krupic F. Factors beyond the language barrier in providing health care to immigrant patients. Med Arch. 2016;70(1):61–5. doi: 10.5455/medarh.2016.70.61-65 26980935 PMC4779347

[26] Abood J, Woodward K, Polonsky M, Green J, Tadjoeddin Z, Renzaho AMN. Understanding immigrant settlement services literacy in the context of settlement service utilisation, settlement outcomes and wellbeing among new migrants: A mixed methods systematic review. Wellbeing, Space and Society. 2021;2:1–19. 10.1016/j.wss.2021.100057.

[27] Settlement Council of Australia. Inquiry into migrant settlement outcomes. Canberra, Australia: SCoA.; 2017 [1–58]. Available from: https://www.aph.gov.au/Parliamentary_Business/Committees/Joint/Migration/settlementoutcomes/Submissions.

[28] Masinda MT. Immigrant Settlement Services Literacy. Int J Social Work. 2014;1(2):1–13. 10.5296/ijsw.v1i2.5335.

[29] Alencar A, Tsagkroni V. Prospects of refugee integration in the Netherlands: Social capital, information practices and digital media. Media and Communication. 2019;7(2). doi: 10.17645/mac.v7i2.1955

[30] UNESCO. Plurality of literacy and its implications for policies and programmes. Paris, France: UNESCO; 2004 [1–32]. Available from: https://unesdoc.unesco.org/ark:/48223/pf0000136246.

[31] Scribner S. Literacy in three metaphors. American J Education [Internet]. 1984 23 February 2022; 93(1):[6–21 pp.]. Available from: https://www.jstor.org/stable/1085087.

[32] Cope B, Kalantzis M. "Multiliteracies": New Literacies, New Learning. Pedagogies: An International Journal. 2009;4(3):164–95. 10.1080/15544800903076044.

[33] Kalantzis M, Cope B. Multiliteracies. 2017. In: Encyclopedia of Educational Philosophy and Theory [Internet]. Springer, Singapore.; [1472–9]. Available from: https://doi-org.ezproxy.uws.edu.au/10.1007/978-981-287-588-4_112.

[34] Lloyd A. Information literacy landscapes—Information literacy in education, workplace and everyday contexts. UK: Chandos Publishing UK; 2010. Available from: https://learning.oreilly.com/library/view/information-literacy-landscapes/9781843345084/.

[35] Kalantzis M, Cope B. The Teacher as Designer: pedagogy in the new media age. E–Learning and Digital Media. 2010;7(3):200–22. 10.2304/elea.2010.7.3.200.

[36] UNESCO. Aspects of literacy assessment: Topics and issues from the UNESCO Expert Meeting. Paris, France: UNESCO; 2005 [1–45]. Available from: http://unesdoc.unesco.org/images/0014/001401/140125eo.pdf.

[37] Leung L, Finney Lamb C, Emrys L. Technology’s refuge—The use of technology by asylum seekers & refugees. Sydney, Australia: UTSePress; 2009. Available from: http://library.oapen.org/handle/20.500.12657/39671.

[38] Nutbeam D. The evolving concept of health literacy. Social Science & Medicine. 2008;67(12):2072–8. doi: 10.1016/j.socscimed.2008.09.050 18952344

[39] Catts R. Indicators of adult information literacy. J Information Literacy. 2012;6(2):4–18. 10.11645/6.2.1746.

[40] Nzomo P, Fehrmann P. Advocacy engagement: The role of information literacy skills. J Information Literacy. 2020;14(1):41–65. 10.11645/14.1.2695.

[41] Lloyd A. Stranger in a strange land; enabling information resilience in resettlement landscapes. J Documentation. 2015;71(5):1029–42. 10.1108/JD-04-2014-0065.

[42] Lloyd A. Information literacy and literacies of information: a mid-range theory and model. J Information Literacy. 2017;11(1):91–105. 10.11645/11.1.2185.

[43] QUT. Migrant’s use of the internet in re-settlement. Queensland, Australia 2012 [1–48]. Available from: https://core.ac.uk/reader/19793579.

[44] WHO. Draft WHO European roadmap for implementation of health literacy initiatives through the life course. Copenhagen, Denmark: World Health Organization; 2019 [EUR/RC69/14 Rev.1:[Available from: https://www.euro.who.int/__data/assets/pdf_file/0003/409125/69wd14e_Rev1_RoadmapOnHealthLiteracy_190323.pdf.

[45] FECCA. Digital access and equity for multicultural communities ACT, Australia: Federation of Ethnic Communities’ Councils of Australia; 2016 [1–15]. Available from: http://fecca.org.au/wp-content/uploads/2017/01/feccadigitalconsultationreport.pdf.

[46] SCoA, GTFA. Supporting the digital inclusion of new migrants and refugees Australia2020 [1–21]. Available from: http://new.scoa.org.au/wp-content/uploads/2021/02/Supporting-the-digital-inclusion-of-new-migrants-and-refugees.pdf.

[47] Coles-Kemp L, Jensen RB. Accessing a new land: Designing for a social conceptualisation of access. CHI ’19: Proceedings of the 2019 CHI conference on human factors in computing systems [Internet]. 2019; 181:[1–12 pp.]. Available from: 10.1145/3290605.3300411.

[48] SCoA. Communicating with migrant and refugee communities during COVID-19: Learnings for the future ACT, Australia: SCoA; 2020 [1–19]. Available from: https://scoa.org.au/wp-content/uploads/2021/02/Report-Communications-during-COVID-19-FINAL.pdf.

[49] Australian Bureau of Statistics. Migration, Australia—2018–2019 Financial Year: ABS; 2020 [Australian Bureau of Statistics Website:[Available from: https://www.abs.gov.au/statistics/people/population/migration-australia/2018-19.

[50] Glaser BG, Strauss AL. The discovery of grounded theory: Strategies for qualitative research: ProQuest Ebook Central; 2004 [Available from: https://ebookcentral.proquest.com/lib/uwsau/detail.action?docID=3410814.

[51] Harris T. Grounded theory. Nursing Standard. 2014;29(35):37–43. 10.7748/ns.29.35.32.e9568 25922026

[52] Engward H. Understanding grounded theory. Nursing Standard. 2013;28(7):37–41. doi: 10.7748/ns2013.10.28.7.37.e7806 24128248

[53] Braun V, Clarke V. Using thematic analysis in psychology. Qualitative Research in Psychology. 2006;3(2):77–101. 10.1191/1478088706qp063oa.

[54] Kapur R. Types of literacy 2019 [1–34]. Available from: https://www.researchgate.net/publication/332875093_Types_of_Literacy.

[55] Condelli L. Forward. 2020. In: Teaching Adult Immigrants with Limited Formal Education: Theory, Research and Practice [Internet]. Multilingual Matters; [vii-x]. Available from: https://doi-org.ezproxy.uws.edu.au/10.21832/9781788927000.

[56] Young-Scholten M, Peyton JK. Introduction: Understanding adults learning to read for the first rime in a new language: Multiple perspectives. 2020. In: Teaching Adult Immigrants with Limited Formal Education: Theory, Research and Practice [Internet]. Multilingual Matters.; [1–9]. Available from: 10.21832/9781788927000-005.

[57] Katrivesis M, Robertson H. Culturally Proficient Service Delivery: Good Practice for Frontline Staff. Research to Action Guide. NSW, Australia: NDS Centre for Applied Disability Research; 2018.

[58] Renzaho AMN, Polonsky MJ, Ferdous A, Yusuf A, Abood J, Salami BO, et al. Establishing the psychometric properties of constructs from the conceptual ‘Settlement Services Literacy’ framework and their relationship with migrants’ acculturative stress in Australia. PLoS ONE. 2022;17(4):1–20. doi: 10.1371/journal.pone.0266200 35381034 PMC8982835

[59] Drummond PD, Mizan A, Brocx K, Wright B. Barriers to accessing health care services for West African refugee women living in Western Australia. Health Care for Women Intertational. 2011;32(3):206–24. doi: 10.1080/07399332.2010.529216 21337243

[60] Kaczkowski W, Swartout KM. Exploring gender differences in sexual and reproductive health literacy among young people from refugee backgrounds. Culture, Health and Sexuality. 2020;22(4):369–84. doi: 10.1080/13691058.2019.1601772 31032722

[61] Nkulu FK, Hurtig A-K, Ahlm C, Krantz I. Screening migrants for tuberculosis—a missed opportunity for improving knowledge and attitudes in high-risk groups: A cross-sectional study of Swedish-language students in Umeå, Sweden. BMC Public Health 2010;10(349). 10.1186/1471-2458-10-349. PMC2905331 20565732

[62] Natoli R. Factors contributing to financial literacy levels among a migrant group: An analysis of the Vietnamese cohort. Int J Social Economics. 2018;45(5):729–44. 10.1108/IJSE-11-2016-0341.

[63] Zuhair S, Wickremasinghe G, Natoli R. Migrants and self-reported financial literacy. Int J Social Economics. 2015;42(4):368–86. 10.1108/ijse-09-2013-0203.

[64] Kumar R. Refugee articulations of health: A culture-centered exploration of Burmese refugees’ resettlement in the United States. Health Communication. 2020:1–11. 10.1080/10410236.2020.1712035. 31931620

[65] Teixeira C. Finding a home of their own: Immigrant housing experiences in Central Okanagan, British Columbia, and policy recommendations for change. Int Migration & Integration. 2011;12:173–97 10.1007/s12134-011-0181-9.

[66] Sheikh-Mohammed M, MacIntyre CR, Wood NJ, Leask J, Isaacs D. Barriers to access to health care for newly resettled sub-Saharan refugees in Australia. Medical J Aust. 2006;185:594–7. doi: 10.5694/j.1326-5377.2006.tb00721.x 17181498

[67] Kim MS, Song IG, An AR, Kim KH, Sohn JH, Yang SW. Healthcare access challenges facing six African refugee mothers in South Korea: a qualitative multiple-case study. Korean J Pediatrics. 2017;60(5):138–44. doi: 10.3345/kjp.2017.60.5.138 28592976 PMC5461277

[68] OECD. Responses to the refugee crisis—Financial education and the long-term integration of refugees and migrants: Organisation for Economic Co-operation and Development; 2016 [1–8]. Available from: https://www.oecd.org/daf/fin/financial-education/Financial-education-long-term-integration-refugees-migrants.pdf.

[69] Barton D. Understanding textual practices in a changing world. 2009. In: The future of literacy studies [Internet]. London: Palgrave MacmillanPalgrave Advances in Linguistics; [38–53]. Available from: 10.1057/9780230245693.

[70] Green J. Bringing literacy to life: investigating literacy in health promotion [Dissertation/doctoral thesis]. Melbourne, Australia: Universtiy of Melbourne; 2008.

[71] Bruce C, Demasson A, Hughes H, Lupton M, Abdi ES, Maybee C, et al. Information literacy and informed learning: Conceptual innovations for IL research and practice futures. J Information Literacy. 2017;11(1):4–22. 10.11645/11.1.2184.

