# Supplementary material for: The importance of information acquisition to settlement services literacy for humanitarian migrants in Australia
Source: PLoS One. 2023 Jan 6;18(1):e0280041. doi: 10.1371/journal.pone.0280041 (PMC9821785; doi:10.1371/journal.pone.0280041)
Supplement: S1 File — (PDF) [file pone.0280041.s001.pdf]

Settlement Service Literacy (SSL) for migrant populations in Greater Western Sydney and Greater Melbourne 2018-2020 Qualitative Interview Guide for settlement services providers

Before we begin the interview, please note that for the purposes of this research study, reference to newly arrived migrants applies to the first 5 years of settlement in Australia and includes both forced (such as refugees) and voluntary migrants (such as economic migrants).

Q1. What services are being provided by your organisation that assist newly migrated people to settle in Australia. These could involve education, health, social, legal or any kind of other social support services?

Prompts:

- Services being provided by your organisation that assist newly arrived migrants.
- What are the relevant services that other organisations are providing in terms of helping newly migrated people to settle in Australia?
- Can you tell us about who you collaborate with to do this work?
- Are there organisations with whom you choose not to work with and why? (Prompt: some faith-based organisations may conflict with the beliefs of some migrants/service is not culturally sensitive or appropriate, not accessible by public transport etc.,)
- Are you aware of any services that are needed but are not available?
- Can you tell us about any other issues around access to these services that migrants are facing?

Q2. What is your understanding of how migrants adjust to Australian culture and society and the kinds of issues and challenges migrants are facing?

Prompts:

- Can you tell us about your understanding of how migrants you work with understand Australian culture and society?
- To what extent do you see your clients are being exposed to Australian culture?
- What are some of the opportunities provided to migrants to practice their own cultural values and practices?
- What are your impressions of how the cultural values and practices of migrants are being recognised and respected by the people in their community?
- What are some of the issues and challenges around this process of cultural adjustment that migrants may be facing?

Q3: What types of programs are responsive to social support, cultural understanding, and acceptance, and improving health of migrants in Australia?

Prompts:

- Can you tell us about the types of programs that are currently being implemented to provide?
    - Social support
    - Cultural understanding and acceptance
    - Improving health and wellbeing

Q4: What types of programs are available for migrants to enhance their financial literacy, income generation and managing money effectively?

Prompts:

- Can you tell us about the programs that are available?
    - financial literacy
    - income generation
    - managing money effectively
- What kind of financial challenges do your clients face while adjusting to life in Australia?
- What are some of the culturally specific dynamics that impact and challenge the management of financial demands among migrants? (Prompts: sending money back home, gender imbalance in financial decisions, financing of dowry vs housing costs)
- How do they overcome these challenges?
- What are the services or supports provided by other organisations to support your clients with their financial challenges?

Q5: What programs and supports are available to support your clients when they face legal challenges?

Prompts:

- Can you tell us about the programs and supports available for your clients?
    - with legal issues around identity, visas, inviting family members to Australia, etc.,
    - physical violence, or other forms of violence or discrimination at different places (home, workplace, school, public places etc.)
- What do you think are the key laws and provisions that migrants need to learn when they:
    - first arrive in Australia
    - within the first five years of settlement
- In your opinion, what is the level of awareness of migrants to accessing key legal services and provisions when needed?
- What kind of challenges do clients of your service face when accessing legal services and provisions?

Q6: What are the key reasons for the movement of your clients from one place to another, or from one suburb to another suburb?

Prompts:

- Relatives living in different area, migrant from the same country of origin, workshop, any religious reasons etc.
- Are you seeing any trends in mobility, possibly in the early years after migration or after several years? (Prompts: economic opportunity, job/ employment, religious or political reasons etc.)

Q7: Can you tell us about the services available for migrants to access education and literacy programs?

Prompts:

- School education for their children, adult literacy programs, or any educational literacy programs.
- How do children of your clients' access school or university education?
- Can you tell us about any special packages, or subsidies provided for such educational opportunities?
- Are you able to outline the kind of employment opportunities that you are aware of that are offered to migrants' children when they finish school or university education?
- How about the employment opportunities for migrants more generally? Are there any special provisions to ensure their employment?

Q8: Can you tell us about the programs and supports available that promote migrants' sense of belonging and inclusion in Australian society?

- Programs or supports available that help to create and enhance migrants' sense of belonging and inclusion? (Prompts: sense of cultural inclusion, cultural identification, emotional support)
- What are your observations of how your clients meet and interact with people from their community to maintain their sense of belonging and of being culturally connected?
- Who do your clients contact for social and emotional support when needed? (Prompts: Family members, relatives, community leaders, religious leaders, etc.)

Q9. Overall, what do you think are the key challenges migrants you work with face while adjusting to the Australian culture and settling in Australia?

Prompts: These could be economic, legal, cultural or social etc.

Q10: And finally, what would you like to see as possible solutions to helping/supporting migrants to adjust well to life in Australia?

This is the end of the interview. Is there anything you would like to add? Thank you for your participation in this interview and we appreciate the valuable information you have provided.
