# Supplementary material for: The importance of information acquisition to settlement services literacy for humanitarian migrants in Australia
Source: PLoS One. 2023 Jan 6;18(1):e0280041. doi: 10.1371/journal.pone.0280041 (PMC9821785; doi:10.1371/journal.pone.0280041)
Supplement: S2 File — (DOCX) [file pone.0280041.s002.docx]

**Participant characteristics**

| **Participant** | **Position** | **Gender** | **Organisation** | **Services provided** |
| --- | --- | --- | --- | --- |
| SP01VIC | Client services | F | Not-for-profit, community-based organisation, migrant specific | Settlement, education and training, aged care, disability, youth |
| SP02VIC | Senior manager | M | Not-for-profit, community-based organisation, universal service provider | Settlement, youth, emergency relief, financial, community services |
| SP03VIC | Program manager | F | Not-for-profit, community-based organisation, migrant specific | Settlement, migration, aged care, disability, family, youth |
| SP04VIC | Client services | M | Not-for-profit, community-based organisation, migrant specific | Settlement, migration, aged care, disability, family, youth |
| SP05VIC | Program manager | F | Not-for-profit, community-based organisation, universal service provider | Settlement, education and training, employment, community services |
| SP05bVIC | Senior manager | M | Not-for-profit, community-based organisation, universal service provider | Settlement, education and training, employment, community services |
| SP06VIC | Program manager | F | Not-for-profit, community-based organisation, migrant specific | Settlement, housing, youth, migration, aged care, family violence, families |
| SP07VIC | Client services | F | Not-for-profit, community-based organisation, migrant specific | Settlement, education, employment, aged care, youth, disability, family, and children |
| SP08VIC | Senior manager | F | Not-for-profit organisation, universal service provider | Settlement services, aged care |
| SP09VIC | Client services | F | Faith based not-for-profit, universal service provider | Settlement, legal and justice, education, employment, mental health and wellbeing |
| SP10VIC | Program manager | M | Faith based not-for-profit, universal service provider | Settlement, youth, family, emergency relief, counselling, community services |
| SP11VIC | Program manager | F | Not-for-profit organisation, migrant specific | Settlement, education, employment, community services, sport |
| SP11bVIC | Program manager | F | Not-for-profit organisation, migrant specific | Settlement, education, employment, community services, sport |
| SP12VIC | Senior manager | F | Not-for-profit organisation, migrant specific | Settlement, youth, education, employment, counselling, family, senior support |
| SP01NSW | Client services | M | Not-for-profit, community-based organisation, migrant specific | Settlement, youth, community services |
| SP02NSW | Senior manager | F | Not-for-profit, community-based organisation, migrant specific | Settlement, aged care |
| SP03NSW | Senior manager | F | Not-for-profit, community-based organisation, migrant specific | Settlement, aged care, youth, community services |
| SP04NSW | Senior manager | F | Not-for-profit, community-based organisation, universal service provider | Settlement, aged care, disability, children and youth, community services |
| SP05NSW | Senior manager | M | Not-for-profit, community-based organisation, migrant specific | Settlement, community services, arts, family violence, employment |
| SP06NSW | Program manager | M | Not-for-profit, community-based organisation, migrant specific | Settlement, aged care, disability, women and family, youth, housing |
| SP07NSW | Program manager | M | Not-for-profit, community-based organisation, migrant specific | Settlement, community services, arts, family violence, employment |
| SP08NSW | Client services | F | Not-for-profit, community-based organisation, migrant specific | Settlement, aged care, disability, women and family, youth, housing |
| SP08bNSW | Client services | F | Not-for-profit, community-based organisation, migrant specific | Settlement, aged care, disability, women and family, youth, housing |
| SP09NSW | Client services | F | Not-for-profit, community-based organisation | Settlement, aged care, community services, disability |
| SP10NSW | Program manager | F | Not-for-profit, community-based organisation | Settlement, aged, family, disability, emergency relief, financial, legal assistance |
| SP10bNSW | Client services | F | Not-for-profit, community-based organisation | Settlement, aged, family, disability, emergency relief, financial, legal assistance |
