# Supplementary material for: The importance of information acquisition to settlement services literacy for humanitarian migrants in Australia
Source: PLoS One. 2023 Jan 6;18(1):e0280041. doi: 10.1371/journal.pone.0280041 (PMC9821785; doi:10.1371/journal.pone.0280041)
Supplement: S1 Data — (ZIP) [file pone.0280041.s003.zip › SP_01_NSW.pdf]

Interviewer: So today is Tuesday the 22nd of October, and I'm at (Service name) with (name)?

Respondent: Yes.

Interviewer: So we're starting the interview at, do you know what time it is?

Respondent: Oh, I think 10:44.

Interviewer: Oh yes. 10:44 a.m. and before we begin the interview I'd just, for the sake of this interview, this research study, the re… any reference to newly arrived migrants, which is probably the same…

Respondent: Yep…

Interviewer: … definition that you use, applies to the first five years of settlement, for refugees and migrants.

Respondent: Yep.

Interviewer: OK. So, question number one. Let's begin. The first set of questions are about services being provided by your organisation that assist newly arrived migrants and refugees. Can you (and these services can be around education, health, social, and any other kind of social support, legal). Could you tell us about some of the services being provided by this organisation, (service name), that assist newly arrived migrants?

Respondent: OK. There's a couple of activities for the new arrival. Normally, actually, we design the activity regarding to the request or to the lack of services, sometimes we've found, OK. For example, we run a program called English for Living. Do you mind if I give you an, a little explanation about those programs? Or just like, name the programs only?

Interviewer: At this poin… where's that music coming from? Is that from us?

Respondent: Yeah. Sorry. Yeah.

Interviewer: Do you mind if we, just so it's not… yeah no, no, it's alright, I thought it was outside but I'm hearing it closer.

Respondent: Yeah.

Interviewer: What we might do first, because we break down the questions as we go along, maybe we'll just name them first.

Respondent: Yeah OK.

Interviewer: And then at the end because there is a section on literacy and education, so we might be able to fill that out there.

Respondent: So, we have English for Living. We have horticulture course. We have computer course. We have citizenship classes. We have homework support for kids. We have sewing classes and I'm trying to remember all of them. So those are the main info session classes or well as driving program.

Interviewer: Oh great, OK. Fantastic. And can you tell us how these services came about, like, how did you come up with those specific…

Respondent: Yeah...

Interviewer: …programs?

Respondent: … we, as I mentioned before, we first of all respond to the request of the clients. If we found that there's a big number of the clients are serving, like are, sorry, facing, some lack of services, let's say, driving classes, or citizenship classes, OK, especially for the test, so we respond in their way which we help them in order to escape this barrier.

Interviewer: Great. And your position?

Respondent: I am a case worker.

Interviewer: OK. So, what is your role with your clients? What do you do with…?

Respondent: First of all, we like, some different ways, sometimes we'll help assist them with understanding the forms, filling some forms, refer them to the services available if we are not have the services, the maker. Assisting them if they have any issues, raise the awareness to the Australian cultural system or the education system or the other, because like, it's totally different from their countries or this is, like my main job, is to like, address them, teach them what to do, or refer them to the right services. So and so.

Interviewer: Great. And what sort of resources have you had provided to help you do that work? So you, what's your source of funding?

Respondent: Yeah. The source of funding is, we have two sources of funding, we have the Immigration Department, the S.S.I. and we have the CoAct program also that support us with funding this organisation.

Interviewer: Great. Great.

Respondent: And also, to be like fair, sometimes we have small grants, let's say, now we're working with Food Faith on the horticultural program so we have a small grant which also we spend it this way.

Interviewer: Oh great. OK. What are some of the services that other organisations are providing in terms of helping newly arrived migrants to settle?

Respondent: There's different services at the (NAME OF LOCATION) area because like we focus on (NAME OF LOCATION) area. We have [indistinct 05:50] which is also provide nearly the same services, plus they have other programs especially for aged people, like, or the disability, disabled people. We have let's say, Mission Australia who also assist with the, supporting some families at home or they have like, anything the families need help with. We have some domestic violence organisation, dealing with domestic violence. And as well, like for example, let's say, we provide assistance by giving a room for N.D.I.S. process, disabled people, as well for the staff. OK. Instead of like, the family wants to sit them like, away from the area, so they, they, we have like enough rooms so we give them rooms here in order to see their clients.

Interviewer: Oh, to do out reach.

Respondent: Yes.

Interviewer: Yeah. Great. So can you tell us about who you collaborate with? I know you've touched on that a little bit there, with, to do your, the work that you do. Who are your main partners?

Respondent: Like, main partners are, I can, Food Faith, like this is the closest people we work with because we're under the same program together. But also we have a link with the Western Sydney Health District. We have as I mentioned, the Mission Australia, we have the WASH House, we have the Anglicare, we have St Vincent de Paul, Salvation Army, all the other organisations because we participate in inter agency meetings, with all those other organisation so we share all the information between both of us.

Interviewer: Oh great. Great. Are there any organisations with whom you choose not to work with, and why?

Respondent: No, like we don't have any organisation because like, each organisation has a different programs, OK, so depends on the needs on the people for intermission also we have Relationship Australia, one of the good organisation we work with as Centrelink and all the others so there's no organisation we don't like to work with, OK because all the organisations are, like, have the different programs and those programs is, like, needful for our clients.

Interviewer: Yeah, great. Are you aware of any services that are needed, that are not available?

Respondent: For sure the most important thing is legal aid. We don't have, we have a, like a legal aid but there's not enough staff there so it's very hard to send people to Blacktown or maybe to the Parramatta so the legal aid is most needed organisation in this area.

Interviewer: Great. Are you aware of any services that are over utilised by newly arrived migrants?

Respondent: What do you mean by that?

Interviewer: Like, long waiting times, to, a lot of demand for one, for that program?

Respondent: Yes, as I said, like, we have the legal aid that there is like a need because like, the booking is very hard.

Interviewer: Right.

Respondent: Especially with the lack of people working. I know, like the organisation like Parramatta legal aid they have only two or three people working in the organisation which is not enough. Yeah, this is the, like, normally like, something different with the health department especially with the specialist and all the waiting list to do the operations, so some people are really in need, OK, so the health issue is one of the main things.

Interviewer: OK, oh that's good. OK. And on the other side of that, are you aware of any services that are underutilised? That there's not a good uptake on them and if there are, why? What do you think the reasons are?

Respondent: One of the things that actually it's the, like I mentioned Centrelink, especially in Centrelink at (NAME OF LOCATION), there are backed by like, numbers which is like a big number living in the area, like the amendings Centrelink services which is not enough. So yes, Centrelink is the first priority I'd say.

Interviewer: Right.

Respondent: Yeah.

Interviewer: But do you think that, do people use that?

Respondent: They use it, but, like I, sorry I have to mention something, why it's a need, because that most of the time they don't provide interpreters, they have only one day interpreter for nearly four hours so it's not enough, especially Arabic interpreters, is what I mean. There's another things with the, comes to my mind now, I'm sorry for that…

Interviewer: That's alright.

Respondent: … for the job providers, OK, the majority of them, they are, let's say, not polite, they don't provide interpreters for their clients, they didn't respect the clients demands, or the clients circumstances and other things which is sometimes, like, they put us on some pressure, my clients, they said that we try to contact them we try to blah, blah, blah, blah, but they are not responding.

Interviewer: Right.

Respondent: Yeah.

Interviewer: OK. That's good feedback, thank you. Can you tell us about the methods that you use to measure the effectiveness of your services, that you provide?

Respondent: Like, it's very hard to talk about effectiveness of our organisation our self because it's like, it's not nice, so it's better to ask the clients. I can proudly say that (SERVICE NAME), even though it's a small organisation with only, like four people working in this organisation, not all of them are full time except the managers only, the only person that works full time, all of us works part time but at the same time, the amount of services we provide is too big and at the same time clients are very, very happy with our services, so they think that we have the magic stick, OK, just like, to fix their issues, and how we co-operate, how we have, I don't know, but normally we lift the clients to their goals or to their target which is, we feel happy that we give our clients a peace of mind most of the time and relaxation, or just like forget about the hardness of being in a new country, new environment, new people and especially the development of language which is like…

Interviewer: So do you like have any like evaluation forms or do you, do you just, is it just verbal sort of feedback from your clients, or?

Respondent: Yeah, we have, we used to have one actually, but we don't rely on it too much, because you know sometimes, like, we need a balance, OK, because like, you can't ask a client who was happy with your services it's just like OK, fill this form, because like, he's happy, so we need the other party to just like give us their feedback and so we yes we have a feedback, normally we use it especially after any info session on any class or anything to see if the service were good or was the, like, lack of services. Also we rely on the social media, we have a couple of Facebook and I think Instagram, I'm not too familiar with this one but I'm not sure, I'm pretty sure for the Facebook, so we get feedback from there yeah.

Interviewer: Oh good. OK. And can you tell me about any other issues regarding access to service, to settlement services that you are aware of, that migrant people are facing?

Respondent: Yeah. The thing is, what I, like, personally from my experience, I've been working with the community for, back, nearly 20 years in Australia. So, first of all, they, there's a need for let's call it, an orientation to the Australian system because the way they educate them in the embassy's overseas one day call the visa that OK, you have your rights everybody tells them about their right but nobody tells them about their responsibilities so there must be a balance for those clients to understand what's their rights and what's their

responsibilities. So all of them know, this is my rights, OK, but you have a responsibility to do, one, two, three, four, OK, so if you don't do, one, two, three, four, you…

Interviewer: What would you suggest that those would be? Those responsibilities?

Respondent: It would be that more orientation, or more info sessions to teach them that OK you have rights and you have responsibilities, you can access this service but let's say for example, I still face a problem even though we have a schedule or a booking for our organisation to see any one of our team, people still come without an appointment OK and most of them, they say OK I just have one question and it takes sometimes half an hour because it's not a small issues it's a big issue, so, understanding that there's a difference between our countries and this country, OK, our system and this system, OK, it's very important for clients, so and to teach them all those things, they will feel that OK we are happy to live here or just oh no, we can't accept this things. So this is one of the issues.

Interviewer: That's great, thank you. So the next lot of questions, they're about how migrants adjust to Australian culture and society and the kinds of issues and challenges that they may face. So can you tell us about your understanding of how migrants you work with, understand Australian culture and society?

Respondent: Look, this question is, has many branches. Like I can tell, like, first of all, like, because most of them, they watch the English movies or the western movies so they think the life is matching those movies which is not right, OK. So, at the same time also, it depends on the background which means like let's say somebody from Lebanon or from Syria, well he's from Syria, so it depends also which area in Syria he is from. If he is from main cities or he's from villages or something so, there's a big difference between people like, people who live in cities they are more able to co-operate or more able to just like fit within the system. But for the others it would be a little bit very hard so it looks like, they feel like they have to design a system for themselves. I can give you a small example actually which is very important in my point of view at least. I remember when I first start, we have a group of the African men, we have a program called mentoring program, so we invite people with, we have a report action from police and from Blacktown city council and things, so other services, try to understand what they need, how we can help them to settle down in Australia. So the first thing they raise, they said OK we have to change the law to take the power of women in this country and give it to men because like, it's not fair for them to just like, all their money goes towards the women's counsel, the women have or has the power now, OK what it needs to be, in the men's hand. We are not in our culture, so the men are the power, they are, they call them the gut of the house, OK, so this is one of the issues

which really, like, very hard to teach them, no, no, just hold a sec OK, there's like, rights and responsibilities, rights and responsibilities.

Interviewer: Yeah, that's great. OK. And to what extent do you see your clients being exposed to Australian culture?

Respondent: Like, it's, as I mentioned before, it depends on the clients, the background of the clients, so as I said, like, cities or the villages, or they are well educated or not well educated, but the most of them, they like things, that say, the order, the law, the things that they like it, but when it comes to their personal issues they feel that OK they have to flexible. OK. So let's say, about the most of them, the fines, the police fines, like parking or offence or anything, so they think that, OK, I just like, drop my son in a no stopping, just like, one sec, OK, to pick up someone or drop off someone, so it's very hard for them to understand that you are not allowed to do so. So this is one of the things, it's very hard to understand that, only one second. So I make one mistake, OK, there's no car, I didn't block the way or I parked in, like a loading zone, OK, there was nobody there, so it's easy. So, this is the thing that they have to, back to the thing, rights and responsibilities. You have the right but it's your responsibility to park here because you are not allowed to, this is not for you OK. So this is the example, especially like if I can say something, with the Centrelink. People think the Centrelink is the wages, so normally longer to make them understand that this is to support you, not to just like steal, not to do anything your own, in order to live OK but this is the amount you have to take, that's only, this is your right, so they feel that OK, just like increase it $100 so it could be easier for us. No, if you need to increase your money, just like go and find a job.

Interviewer: Right. Thank you. And what are your impressions of, oh sorry, what are some of the opportunities provided to migrants to practice their own cultural values and practices?

Respondent: Yeah, we, I make I can say, we assist people like, we have lot of activities run by different groups, let's say, we have the Samoan group, we provide them, we have big meeting rooms, we provide, like, make the rooms available for them for free and most of the time we provide them with tea, coffee and things for free also, in order to like, having a group for the Samoan, we have another group for the Pakistan people, we used to have like a different group, they are not belong to make it out from outside but they need their space to meet so we provide them with the space to make it easier for them, and to assist them and most of the time it comes, sorry, it comes with an idea, like we need, blah, blah, blah, so we try to see if we can help them or assist them to make this available for them.

Interviewer: Oh good, great. And what are your impressions of how the cultural values and practices of migrants are being recognised and respected by other people in the community?

Respondent: It's, well, it's well recognised by the community because as I said now, like we use, I remember once we have a group of Pakistan people, they used to practice some religious activities, so we make it available for them because they don't have any space OK and so this is the type of services we assist those groups in order to practice their cultural issues, their religious issues or whatever they like, as long as it is within the understanding of others, it's not like a race or discrimination or things against them or other things so as long as it's not discrimination of others or other things like this which we are aware of, they are more than comfortable to practice.

Interviewer: Do you feel like there's an overall acceptance in the community…

Respondent: Yes.

Interviewer: … for people to express their…

Respondent: Yes.

Interviewer: … own way of being…?

Respondent: Yes. Yes. Yes.

Interviewer: Great. And what are some of the issues and challenges around the process of cultural adjustment in, to Australian culture, like?

Respondent: I can say the funding is the most important thing, because like, this country is costly, OK, so anything you need to practice or to do you need to have money in order to have a space or to provide equipment or food or anything, so this is one of the challenges. Some of the groups they, depends on donations between themselves OK because even if you need to do a donation, you need to have a licence or just like an authority to gain money from people so this is one of the things we also lack a process with the lack of sensitivity.

Interviewer: Right. Thank you. OK, so the next questions are about migrants' sense of belonging, what we were talking about before, and inclusion in Australian society. So can you tell us about the programs or supports available that help to create and enhance migrants sense of belonging and cultural inclusion?

Respondent: Yeah, I can, I can talk about like, all our programs actually design this way. We try, we trying to have a multicultural community like we are, our organisation is multicultural with ethnic communities, Asians, which is like it's a, have a diversity of backgrounds. So, in the English for Living we have like a group of different backgrounds, we have from Pakistan, from Lebanon,

Syria, all things, all those in together. The horticultural program also the same. With the homework support for kids also we have like, the same, like, group. So all our groups are mixed.

Interviewer: Great, OK.

Respondent: It's not like, one way or one colour.

Interviewer: Yeah OK. And what are your observations of how your clients meet and interact with people from their own communities to maintain their sense of belonging in cultural connection?

Respondent: Talking about belonging, it's really like, it's an issue by itself. OK. It's very hard for them to figure out their belonging and you can't tell it from seeing that OK let's say, Fairfield. Most of the Iraqi people like to live at Fairfield. If you move to Cabramatta, so you can see the Vietnamese aren't living there, because in our culture we have this social life OK, so people feel like they have more power if they are like linked together with others, and together, especially like let's say, if they want to go to church, if they want to, like to the mosque or any other places to worship so that's why sometimes it's very hard to belong to other activities, like other groups, unless we design something to make them like, interact with those activities, let's say for the horticultural program which is the first time we run this program, it runs by TAFE in the future, so we have maybe a group of 12 people from different backgrounds OK sitting to learn something new together so this will build something new because they are doing something which is related to agriculture OK so it's nice to have like, OK we plant this and now we plant this way and now we have the different to plant so it's a time of grabbing them to belong to this program and from this program they belong to this land and from this land they belong to this country.

Interviewer: Oh beautiful. And who are the key people that your clients contact for social and emotional support when needed?

Respondent: Well the, this is a good question actually. We try to link them to the, like, depends on the issue for them. So, from my experience they have lot of issues with the domestic violence because people misunderstand the Australian culture. There's no violence OK. So we link them to the proper service. Most of them we refer them to the services, if they needed say family counselling which is also a good program to assist them in order to skip their issues or family dispute, and for the kids actually also I like to refer them to the, there's couple, like, nearly two different services but they are doing like, there's different service providers, but they are doing the same thing, some of them they do from let's say zero to 12 years of age, the other do it from 12 to 24 OK. Especially with the kids they, like I will make target, we have a youth worker which works on this domain, kids are suffering from English barriers,

different way of education system in Australia, so most of them, they, difficult, let's call it mental issues, so we like to deal with them in early ages before it's accelerated, and so it will be very hard to control.

Interviewer: Right, and cultural people, people would be church leaders, or?

Respondent: Yeah, we yeah, we try that, some of them they ask, like we need to practice our beliefs, or we tell them that OK, there's like say, there's two mosques here, we can ask them if they would like to attend, if they, like, actually have a white one, one client only once he said like I have to pray in a mosque and I said well OK, there is a close mosque here, like five minutes walk, and there's another one if you like, that's up to you, and then people they will ask OK well we need like a, because you know the different churches like different, different, preachers there so we tell them that OK this is a Coptic church, there's a Catholic church that's all we tell them, is the churches or worship places located.

Interviewer: Great. OK so the next questions are around the programs that are responsive to social support and improving health of migrants. So you've touched on a few of these already, so, can you tell us about the types of programs that are being implemented to provide social support?

Respondent: With the social support, like, we, we in contact with the clients to the, like, either if they would like to practice a social support by having a social life like visiting each other or any activities, other activities or also social life which is related to the religion which they would like to practice these things. So, for like, say for religion for example, we hold a quota staff for a Madan in our premises during the Madan which is like we invite Muslim people to just like participate and also we invite Christian people which is some of them, that we are aware of, we invite also service of providers to raise the awareness that OK, this is the practice of, during Ramadan. This is from religion prospective. From the social life there's a lot of activities, especially like say, for other people OK. Some of them run by different organisation or different programs either they took them on an excursion or played chess or play, what do they call it? playing cards or things or we link that to those services if they are all ages. For the young ages we have also let's say, for the youth, we have a junior day, we run a couple of activities either movies or the excursion or the other things so this like, makes, we link them socially to the other people, to the other communities as well.

Interviewer: Right, fantastic. What about health and wellbeing programs around that?

Respondent: Yeah. This is one of the important things. We run like between five to ten sessions about health awareness. Last week we have a session about the prostate cancer, before we use to have, we have a session about the stress management, so we use the other health departments to process us with those

courses and also at the same time we have a good link with the doctors who work in the area here, so if there's a common issue, we are raising, or any issues, we raise the awareness between our clients through the info sessions and the other activities and also we were member of another program called Hepatitis, so we run lot of activities because for example this program they found that the Egyptian community have a high number of Hepatitis C so we raise the awareness within the Egyptian community. As soon as I meet a client from a Egyptian background I ask him how long he has been or she has been in Australia and at the same time if they have been a short period of time I ask them to just like go and do it, like to make sure that everything is there, is under control.

Interviewer: Great. So are you aware of any things that are promote, or any, we've called it enablers and barriers, that your clients have experienced when accessing any of these programs, are there anything that comes to mind?

Respondent: Like, all the, like, it never happen actually because you know sometimes it's because we work as a partnership with the other service providers, if there's anything, because when we refer someone there's someone will respond to our email and to check like all the details about the clients and things, to see if there's any, any barriers or anything that like, let's say, I have a client who need speech therapy, once I remember, so I refer them to the service provider and then they because the social provider was like not within my broad area, so what we did, is we were able to assist this client with the taxi vouchers, or I don't know what they call them…

Interviewer: Oh yes…

Respondent: So in order to make it easier for this client to go to Blacktown, OK, they can go by train actually but we need to make it zero for them so we have that through this scheme in order to...

Interviewer: Oh that's a great example, thank you. Now did you…? That's alright. Could just see someone trying to get to you.

Respondent: Yeah.

Interviewer: The next questions are about financial literacy. Do you want to get that? Yeah. That's fine.

Respondent: It keeps ringing like this, I'll just…

Interviewer: Alright.

Respondent: Yeah, OK, sorry.

Interviewer: OK, so, can you tell us about any programs that are available for financial literacy?

Respondent: Yes we have programs, like, we have program called I.P.A. Vouchers to assist clients.

Interviewer: What was it called?

Respondent: I.P.A. Vouchers.

Interviewer: I.P.A.

Respondent: Emergency like, OK, I can't… remember the name…

Interviewer: That's OK.

Respondent: Known as I.P.A. It's emergency something for power assistance for things. So people who need for electricity or gas OK, we are able to help them OK, if they have like a higher month of money or they have financial hardship or things, or we put them on this program, we assist them financially, as well as we send them to the other service provider, let's say for St Vincent de Paul, Salvation Army, Anglicare, some of them they have programs which they can give cash money or maybe they can assist them with the shopping cards, or…

Interviewer: Right, great. What about income generation, any programs you're aware of?

Respondent: Like, I didn't, income…

Interviewer: No work, sort of style programs, or, how to generate an income?

Respondent: Yeah, with the programs actually like, I forget to mention something, we are working now with S.S.I on their program which is for to reorganise the overseas qualifications for people.

Interviewer: Oh great, OK.

Respondent: And also, if there's a need for the skilled person so we refer them to the job positions, this is one of the programs. The other programs for assisting clients is we work also with TAFE most of the time to just like, if we have a group of people they would like to learn about carpentry for example or about plumbing or things, so we try to organise a special course for them, like a compact course, or short period of time, so we are working with TAFE now to do a course about food handling, OK and hospitality, so those are some of the support we provide to the clients or that just like to push them towards finding work and also this help them in the way of belonging, when they start working the start seeing.

Interviewer: Great. Are you aware of, you’ve touched on it before, but the kinds of financial challenges that clients face while adjusting in Australia?

Respondent: Yeah. There’s some big challenges actually, it’s also one of the questions, or the issues that clients raise. Because like the amount they are getting from Centrelink is very limited, is very tight and they are not used to it, like having this amount of money because like it is very small or very low income. With the life expenses from electricity to shopping to things, so we raise this awareness with the clients about what we call it, we invite Centrelink to talk about money management, OK, how to manage their money, how, because like this is, like, you have $500, you have to live within $500. Some of them they don’t, they feel that OK we can have a bigger house for $1,000 per week which is OK, now we can’t offer, you need to just like manage your budget OK. So we assist them with running a session about managing budget, managing electricity use, managing water use, OK…

Interviewer: Oh great.

Respondent: …and other stuff, in order like… Also it’s a time of belonging. When they feel that OK this is enough for us, they feel that OK we belong to this country.

Interviewer: Great. And are there, do you, are you aware of any culturally specific dynamics that may impact or challenge their management of money? So say, you know, sending money back home or things like that that are culturally expected or?

Respondent: Yeah, this is, as I mentioned, like because why they can’t manage their money because most of them they left their families overseas, especially like their fathers or mothers which is, elder people, and there’s not enough money just like to live here, they would like to transfer money to their families that are very very tight for them and that’s why they start to go to the black markets so OK I can work couple of hours cash money, this will support me, which also puts them in a trouble and then that’s why they feel that OK, this country is bad because like they are following us with everything, so…

Interviewer: Yes.

Respondent: We try to raise the awareness you see. Rights and responsibilities.

Interviewer: Great. Yes! So how do they overcome these challenges, so the financial challenges? What sort of solutions?

Respondent: Yeah. Like, raise of the awareness, that OK what you are doing is wrong, OK. Because I have a couple of people who like, we run a session about this in, forget about the cash money, try to work on tax because this will protect you from compensation, for sick leave, for, for, one, two, three, four, five…

Interviewer: Great.

Respondent: Some of them, they follow the instruction, the others they didn't follow it. I remember one of the clients, he start working and I raise the awareness, listen, it's a dangerous job, OK, if something happen, because he's an electrician, I said, if something happen to you, nobody will protect you and what happened, like, I missed him for a couple of months and then he came back with a broken hip, OK, and he said I can't work anymore because like I was at work and then I fell down, I can't get compensation, I said because, you have made a big mistake for life OK. If you are working on tax you would be covered and you would live safely in this country. So, in my point of view the of awareness of like, rights and responsibilities. So, this will help them in order to manage it. Because there's lot of other people who convince them OK come and work for me for $100, they would think OK $100 is good, so that means that I can get in one week, like which is equal to what I'm getting in two weeks from Centrelink but at the same time I can keep Centrelink payment, OK, which is also but that's not right. So we try to raise the awareness that this is wrong. You have the right to work but your responsibility if something happened, you are not covered.

Interviewer: Great. And are you, I know you've named a few already, some of these other services that support your clients with their financial challenges?

Respondent: Yeah, and as I said there's a program run by, I can't give you the right name actually, yeah, it's Energy N.S.W. which help with the, yeah, help with the I.P.A. Vouchers OK.

Interviewer: Oh right.

Respondent: Also like, I can give like few examples actually, Anglicare, they have a program for assisting for $3,000 without interest if you would like to buy a car. It goes through a bank OK, they assist them with this one. There's another programs like run by private organisations like let's say, what's bank, I remember they can give you up to a $20,000 if you like to have your own business or things with the law interest so we try to link people to those activities OK if they need like financial assistance. The most important this is we try also, we have a link with the couple of private sectors, we have a builder or contractor so he sometimes offer people to work with, people who don't need very high skills let's say paintings or we can link them to those type.

Interviewer: Great. OK. Thank you.

Respondent: You're welcome.

Interviewer: You doing OK? Yeah, we're not going too quick?

Respondent: I hope I'm giving you the…

Interviewer: Yes. Yes.

Respondent: … the right answers.

Interviewer: Great information. Thank you. So the next questions are about programs that support your clients when they face legal challenges. So can you tell us about the programs around, that support clients around with legal issues around identity, visas, inviting family members to Australia, that sort of thing?

Respondent: Yeah, we try like, normally with this issue, it's very sensitive for me, because I know if you refer them to the private solicitor or to the private migrant agents they would charge them lot of money so we try and, we have couple of, also organisation, like a government organisa… not a government organisation, N.G.O. OK, who can help with the supporting functions for clients if they are eligible for, like people who are over five years so sometimes it's very hard for them to contact those places. People who are working so they can't pay, but who I'm talking about, people who has, who have, sorry, low income, so we try to link them to those legal providers, to assist them with their visas or their things. In our organisation we assist in filling the citizenship forms, like, if the visa expired, we assist them with all those things for free, we don't charge anything for the clients.

Interviewer: Great. Great. What about supports for around clients experiencing physical violence or other forms of violence? In different places, it could be home, work place, public places? Or even discrimination?

Respondent: Yeah. So it depends. If its in the work place, like, as I said like, with the information session which we run every week we have a different topics, every week we have like speaker from like we say legal aid, from the fair trading, from the other different organisation…

Interviewer: Oh great.

Respondent: … they raise the awareness that if something happened like at the shopping centre, at the clinic and the, whether you're Asian, with anyone, so those are the right places to go to. So mostly it goes through us, like say someone has a problem with the Asians so we refer them to the Ombudsman to assist them with their issue with electricity or gas or things or the Centrelink or any government body, OK, or with any private issue. So we, we, like, it depends on the case, OK. Somebody, like…

Interviewer: And you work with the WASH House.

Respondent: Yeah, I can give you something actually, like, last week somebody, client, got an A.V.O. from the police OK, for something he was like mistakenly did, OK,

he didn't intend to do it, but the police wasn't patient and the police, my point of view is stand by one side against the others, that's why they gave him the A.V.O. so they see that OK we are victim. We are, we have to take our rights, so I ask them to follow some steps we have supported them and they took it to the court and the judge dismissed the case against them which we feel that we did something good for them, they were very happy and especially the things come, like, before Christmas, and they said OK, now we can celebrate the Christmas because we win, which sometimes you feel that, especially like the impact of this case on them, like, we are trying to be a good people, we are trying to find a job, we are trying to people like with others so why it comes to our side, this way, so, yeah, this is one of the things we assist with.

Interviewer: Great. Thank you. So, what are the key laws and provisions that migrants need to learn when they first arrive in Australia, what do you think?

Respondent: There's heaps of things clients they can… if you don't mind for a second thanks…(answering phone call)

Interviewer: No, no, it's alright. Yeah.

Respondent: "Hello, no darl, if you don't mind because I'm in an urgent meeting so I can call her later if you please, thank you, thanks, bye." Please forgive me.

Interviewer: No that's alright.

Respondent: So, what was the question sorry?

Interviewer: Just what you think the key laws are that migrants need to learn when they first arrive in Australia.

Respondent: Yeah. It's very important for the new arrival to understand the new system, in Australia, the western system OK, because like, in our country if you know someone of your, like, I remember in my previous job I used to like, be in contact with the politicians and the, help people and people used to ask me, like, is it possible to ask the minister to give me blah, blah, blah or the M.P. to give me blah, blah, blah, that we have a good relation with the local M.P. here (NAME) and because people like saw us sometimes like laugh or shake hands or things they see that OK, we are good friends, they ask me is it possible to ask him to support our visa or things so this makes me want to think about inviting (NAME) to my group and there was around 50 people attending and then he talk about what he can do or what he can do, OK, in order to raise the awareness OK, listen, OK, you have to understand the system. In Australia a politician is a federal member of parliament but he can't act as the federal member of our countries OK, he have the rights but also he has the responsibilities. OK. So, yeah, this is the, the, the things that, that's why I run the info session every week OK to talk about different topics, to say like, OK,

if it's no stopping that means no stopping, OK, if it's like, one minute parking, it means one minute parking, if it's five, four hours parking, it's four hour parking, OK, so you can't say, just like one minute after four. No. Four, that means four. That's it. So this is the challenges which I am trying to cope with it, know that to give them the base to live in a, this country.

Interviewer: Great. And in your, in your opinion, what is the level of awareness of migrants to accessing these legal services? When they need them? Would most people be aware, or?

Respondent: Let me tell you something actually that, that comes to my mind from my experiences, that, people sometimes they rely on their relatives here, like we call it, word of mouth, so some of them they give a propaganda, they think OK, then I don't use this services, they will not help you, they will not, blah, blah, blah, those things, OK, and then they try to give them other ways which is sometimes illegal ways or sometimes they get them in trouble and then they finish up and they say sorry, we follow the instructions of others which is not right OK. So also, that's why at the beginning I said, the orientation OK, the presentation which is done at the Australian embassies overseas OK, I think it's not enough. There must be a focus on this one to teach them OK, you have rights but at the same time you have responsibilities. In this country you can't like, I remember one of the clients he tried to give money to the police officers because he apply for a job so if he got that fine so he would be in trouble, he tried to give money to the police, so he said OK, see you at the court.

Interviewer: Right.

Respondent: So they think that it's easy for us to just like, skip it. Or if like let's say, if I know you OK and you work for let's say, this position of things which is, you can help, so how can you help me with this one? It's easy just like… but I work as also the Justice of the Peace so some people they came in without the I.D. the original I.D. and they say, you know me, OK I know you but I didn't see the original I.D. I have to see the original I.D. OK And they keep insisting that, you know me, I'm your clients I have been with you for one year blah, blah, blah, I understand all this, I respect, but you have to show me the actual one. How can I trust that this is the right thing? This is the responsibility OK. You have the right to sign it, but you have the responsibility to give me the original I.D. and it's my responsibility to see the original I,D, to be able to practice my right to sign it.

Interviewer: Great. And, can you identify any challenges that your clients face when they're accessing these legal services?

Respondent: The expectation. Another way, like the belonging and expectation. People think that if I, let's say, we have in our culture, I swear to God that this is truth OK. And this country doesn't work like this. Here you have to give

documents, you have to do documents OK, so people think that OK, the most common thing is like regarding to the bonds, housing bonds, so they say like, we give the house as it is when we receive it, so there was nothing, it's clean, it's nothing blah, blah, blah, but they charge us, or they took all the bond OK. When I check everything I found that no, we are wrong, because you left a rubbish bin behind, you left a spot on the carpet, marks on the walls, so, Ï just left a small mark, even if it's a small mark or a big mark, so you have to practice your responsibilities OK. So this is one of the things so they found that OK, like it's not fair. They feel that's it's not fair because like we paid the bond for nothing. While at the same time no, you paid the bond for an legal issues or wrong issues you make, while you was living at this room and this house.

Interviewer: Great. OK, so the next… there's only a few more questions, the next question is around the movement of your clients from one place to another. So what do you think the key reasons are for the movement of your clients from one place to another or from one suburb to another suburb?

Respondent: The most important thing I think is the families as I said, like the social life OK so they feel like that OK we need to be close to those, to the other people because if we are in trouble, if we are, if we need something, some help, OK so those relatives would be close to us or those friends would be close to us OK. Some others, like a few cases they feel that like let's say, (NAME OF LOCATION) area is one of the low rank areas in Australia and they feel that if they move to Parramatta or maybe to the cities so that means they are in a higher rank of life OK.

Interviewer: Yes, yes.

Respondent: So they feel like OK.

Interviewer: Fair enough. And are you seeing any trends at all in mobility?

Respondent: Like, most of them they use the public transport OK, but after a few like, because we have the driving program which assists them in order to reorganise their overseas driver licence and then we assist them in order to practice the learner driving licence and then also assist them with the educational, teach them the private sector. So, when they have the licence they start to just like, linking to the program of Anglicare, having a few thousand dollars from them, so it's easy for them to buy a car and start their own way of life.

Interviewer: Right. And are you seeing people move around in the earlier years or after they've been here for a while?

Respondent: No, the majority is the beginning.

Interviewer: At the beginning.

Respondent: At the beginning.

Interviewer: Oh right.

Respondent: They try to be close to their friends and relatives.

Interviewer: OK. Great.

Respondent: Yeah.

Interviewer: OK. So the next set of questions are about access to education and literacy programs. Can you tell us about the services available, (excuse me), to migrants using your service in terms of school education for their children, adult literacy programs or any other education or literacy programs?

Respondent: OK. For, let's just start with the program we are running, the homework support which is, we assist them because there's a big, big difference between our system and our counties and Australia. So, this program will assist the kids in order to escape the barriers of education. One thing, the other thing also we have a good link with a coupe of schools around (NAME OF LOCATION) and surrounding area, we assist the class with filling the school forms. We also invite the, some teaches from school or principals to talk about the education system in Australia. As well, we try to assist clients by telling them what's the difference between public and private school in Australia OK. And then how to access like preschool, how to access primary school, how to access high school and what the responsibility of kids to be good at school, also support them with their homework if they have any issues or things we try to help them.

Interviewer: So that's touching on the next question which is, what do you think some of the issues or barriers are for children of your clients to accessing school or university?

Respondent: With university, actually thanks for reminding me, with university also we assist a couple of classes in order to enrol at uni, because there was a little bit barrier but by co-operating with S.S.I. to recognise the overseas qualifications which is very costly and then they can't offer it, we assist couple of clients also with a different programs like I have a pharmacist who would like to recognise his qualifications but he has to do couple of courses, so he tried to escape the study because he can't offer and then by the assistance of S.S.I we found a place where he can pay only $500 to do the uni which he is supposed to do…

Interviewer: Oh great.

Respondent: … in order to assist him. So, we trying, like, I can't tell you about one thing, in particular, but there's a couple of things we tried to think which is the right

service, or like, we keep looking we keep searching to see the right place for this person to achieve his goals.

Interviewer: Great. And can you tell us about any special packages or subsidies provided to support education opportunities?

Respondent: Yes, for sure, we, like, we have a, I forget to mention it, it is one of our successful program, it's called the mentoring program. We run it at the moment with the Chisolm College. We try to assist clients by getting their own models, let's say for, to talk about their experience in Australia and this encourages other students to know that OK, it's easy, it's not very hard for us to achieve what we need to achieve. I can proudly talk about a couple of clients, which we support them, they are finished uni at the moment , they found their places, one of them used to work for a research centre, a medical research centre, so like, there's different ways of assisting, different ways of approaching people and then link them to the right service.

Interviewer: Great. And are you able to outline the kind of employment opportunities that you're aware of, that are offered to migrants' children when they finish school or university?

Respondent: Yeah. It's a little bit like, this question is a little bit tricky because like, in our culture either you have to study engineering or medicine, which is, this is like big, big, big talk of education in our countries. So sometimes it's very hard to convince people like, don't think that way OK. In this country whatever qualifications you have, some of them they don't have qualifications and they have a good position OK. And I said, listen, in this country the plumber may be that he can get more than the doctors can gain in a whole year so plumbing is something important. So, look, it depends on the clients. We like, have a chat with the, first of the all with the kids, we have a chat with the parents. We try to convince them not to push them, OK, we know this is right for you. Have a couple of clients who, like, they found that it's very hard for them to co-operate well at school because of the English barriers and things but their English language wasn't so bad so I can talk about couple of them, they are doing now electricity at TAFE, some are doing plumbing, some are doing social or community studies OK, community service studies, so we try to just like, it's easy for you to start this way if you've like, you feel that you don't need to go to uni you can do this one, but if you feel that OK you finish this one, you find a job, you start working, so what you studied at TAFE it can be implemented at the beginning of uni and you can keep doing it to get a, or gain a uni degree, which is also helped them.

Interviewer: Great. OK. And how about the employment opportunities for migrants more generally? Are there any special provisions to ensure their employment?

Respondent: Like, this one is, there's a program at Centrelink links to the job providers which is, I don't trust this program, I don't believe in, because there's say like a big gap in linking both things OK. Because there's a lot of skills, what they need only just like a small English language, small English skills, in order to find, to work in their domain OK but sometimes they change like the service providers and then they're like, we can't find you a job in let's say, hair dressing or something like this, we, it's easier for us to find you a job in cleaning. Some of them they like, I don't like to work in this domain, OK, we are good at this, like at hair dressing so we would like to do hair dressing, so this is the thing which is sometimes is very hard. There's a, like also we have a double partnership with the service provider called Sarina Russo which also they have programs OK, a small or a short education system for clients OK, to practice their skills and then find a job in their domain.

Interviewer: Oh great.

Respondent: Which is yeah, we also try to work with them in this domain to achieve the clients' goals OK. So, it's really, it's really like, because, couple of weeks ago I met a professor in engineering OK he said I have 35 years of experience in teaching, he has five, I don't know what they call it in Australia like he have, yeah, he has five inventions recorded under his name in Russia OK, but he said listen, my English skills is zero. So I can't do anything, but if you just like ask me to draw anything I can do it. But because, like the English skills so you can you can go anywhere OK. In my point of view, those people, if you try to put them in their domain OK, with a little supervision with someone, they will practice their skills and they will be very beneficial for our community, for our social, like in general.

Interviewer: Yes. Fantastic. That's great. So overall, what do you think, just in general from what we've talked about from this whole interview, what do you think are the key challenges migrants you work with face, while adjusting to Australia, Australian culture and settling in Australia?

Respondent: There's lot of challenges. The English barrier is one of the challenges, with like the most priority thing. The second thing is the lack of jobs OK, which is like also very hard, but if you like link them together so you can see that OK, if you don't have English skills, so for sure you will not have a good job OK. So how to fix it, I don't, like I don't know, I'll leave it to the expert but there's also a program with the education system, with be A.M.E.P. they call it, the English for migrants, so the way also they teaching English is, is not the right way, OK, because it's, they mix up between someone who has let's say a uni degree or someone who never been at school at the same class because they are level one OK so the understanding of the education people or the educated people would be higher than the others OK, so this is one of the challenges they feel that, I don't like to be in the class because like, they need, like the

teacher has to explain it ten times to my colleague at the class while I got it from the first time OK. So this is a challenge for them because they feel that they are wasting their time. Also, I think the one of the challenges is, by understanding the system, the Australian system, would be easier for them in order to feel OK we're alright now OK.

Interviewer: Great. That's great feedback. OK. Final question! What would you like to see as possible solutions to helping supporting migrants to adjust well to life in Australia?

Respondent: In my point of view I feel that if they, if they understand the, let's call it, if they read between lines OK, the Australian system, it would be easier for them, just like, to live in peace OK, but misunderstanding like, by having the English barrier OK, and the short of money so this prevent them from trying to achieve what they are looking for, so it's really like, they feel that OK, we are, like, turning in a circle, we can't go anyway, just like turn around, turn around, turn around, especially like when you have people like over 40, the percentage of not understanding, the absorbing the information or the knowledge OK will be like, degrees, it's not degrees OK, it's different from like the Asians OK so that's why it's, it's not one of the challenges.

Interviewer: OK. Thank you. So that's the end of the interview. Would you like to add anything else that we haven't covered?

Respondent: Thank you for this program actually. I'm really glad to be part of it and if there's anything I can help with, I'm more than happy to do it because like this is for the sake of our community and the new arrivals to Australia.

Interviewer: Thank you very much. That's fantastic. So we end the interview at 11:56.
