# Supplementary material for: The importance of information acquisition to settlement services literacy for humanitarian migrants in Australia
Source: PLoS One. 2023 Jan 6;18(1):e0280041. doi: 10.1371/journal.pone.0280041 (PMC9821785; doi:10.1371/journal.pone.0280041)
Supplement: S1 Data — (ZIP) [file pone.0280041.s003.zip › SP_01_Victoria.pdf]

Interviewer: So this is (SERVICE NAME) Interview 1, (NAME OF THE INTERVIEWER), and the participants name…

Respondent: Is (NAME).

Interviewer: Excellent, and the time is let's say 11:25. Should do. Alright, so before we begin, we want to, for the purpose of this study, when we refer to newly arrived migrants, that applies to people in their first five years of settlement, and includes both forced and voluntary.

Respondent: Yep.

Interviewer: Excellent. So question one, I'm also going to say question one, because I think in my view it helps with the transcription and everything else, because you can quickly look and say it's question one. So the first set of questions are about the services being provided by (SERVICE NAME) that assist new migrated people to settle to Australia. So education, health, social, legal, an services you provide. So could you tell me about the services currently provided by the organisation that assists newly arrived migrants?

Respondent: So for newly arrived migrants that have been here for under five years, we have a settlement engagement and transition support program. So that provides casework and low and medium casework for new arrivals. Primarily refugees, or partners of refugees. And within what we call the SETS program we also apply for funding to run group education and capacity type building programs. So at the moment with the SETS program we run a youth type activity called Hub Club, in (NAME OF LOCATION), which supports newly arrived primarily mainly Iraqi new arrivals, but there's a little bit of a mix. But it's primarily Iraqis. That runs one day a week, that's for 16 to 25 year olds. We also run a program called Women of the World, which is a capacity building program not just for new arrivals, but it's for migrant women and it's to help them develop skills to work, to be able to deal with a crisis. So new arrivals attend that one, but we've got women that have been living here for over five years as well too. So they're our (NAME OF LOCATION) programs, and we have caseworkers that run out of this site. In the west, at (NAME OF LOCATION) with SETS, I've got two workers there. We also, sorry, we also outpost at (SERVICE NAME) too.

Interviewer: So that's three locations.

Respondent: We outpost here, there and everywhere, depending on the programs. So we outpost at (NAME OF LOCATION), which is an English language school provider, it's a TAFE. So we provide casework there as well too. In the west we also outpost at an English language provider called Community Plus. We have been running a road safety program, which is a capacity building program around road laws and responsibilities for new arrivals. What else are we running out the west? Just trying to think. That's all we're running in terms of the SETS program and casework as well too. We also do, it's not the only thing we do. We also work with primary, sorry Men's Shed program, we do a multicultural men's shed program every second fortnight on a Friday night.

And we also provide, we do info sessions for parents at some local primary schools, at three local primary schools, where the schools identify gaps in parents' knowledge. So it's new arrival families. And we'll either get in guest speakers or we'll develop a bit of a syllabus to help identify, help teach the parents whatever the issues is that the family are dealing with. Could be Centrelink, I'll give you an example.

Interviewer: So does the school identify that? Like we've got a lot of people that we think are having issues, or a few families having issues with Centrelink, can you come in and provide some…

Respondent: Yeah, we'll get a Centrelink, someone from Centrelink to come in. It might be because everybody wants, like Centrelink wants you to report everything digitally now, and so we've got barriers around language, not understanding how to use the app. So we don't want people to just go into Centrelink and spend a whole lot of time waiting in Centrelink and all that sort of stuff, if you can do stuff by yourself. But people need support with that. So the school don't know exactly how to do that. It's a partnership. So they'll approach us. We've got the relationships with Centrelink. We know how to put the cultural lens on things as well too. Some of the schools, you might have a couple of teachers in there, or a coordinator or whoever, and we'll do a little class. That's an example of. We've done a lot of stuff around consequences for actions and the way the schools discipline children, and how we want to work with parents as well too. So it's not just only the schools do the disciplining, it's parents have to do the disciplining. See, overseas when your kids go to school overseas, the discipline comes from the school. Not necessarily from the parents.

Interviewer: Yeah, right.

Respondent: So that's conceptually for people here, people think why aren't the school doing something about it?

Interviewer: That's interesting.

Respondent: Actually as parents, you have to do something about it. So we talk about that. This is the way the school operates and we do it for these reasons. Are you able to operate like that well too, so we've got a partnership approach with the way parents… and it's consequence for actions. We also talk about ages and stages, child development as well too. And people that have come on forced migration, there's also trauma involved too, so that can impede learning. So we also talk about that. There's a variety of stuff, we have people from child protection come in because kids are often the first ones to understand language, and they're very quick to understand what their rights and responsibilities are, and they can dish that up to their parents, and say if you smack us or whatever, we're going to call Child Protection. That's an example of, and it happens. And the parents are scared, because they think the kids are going to be taken away. And believe me, Child Protection are not going to come and take kids away. So we have to just destigmatise this whole thing that yes, there is child protection, but just because you're disciplining your kids, it

might not be smacking, it might be they've had a blue at home. We just have to say Child Protection are there for this reason, not because you're having a fight with your kids. So parents are, you end up with intergenerational problems as well too. So we want to be able to arm the parents with the right information and the kids have got some truth in what they say, but it's not the full truth. So the dynamics change. The power balance changes in families as well too.

Interviewer: And so all of these are programs that you would offer through these school partnerships.

Respondent: Yeah, so we do that. And then M.R.C. more broadly, we're a registered training organisation, which provides training to the greater community but we do have a focus on migrants and new arrivals. So we're doing individual care, disabilities, Cert III, Cert IV and now we're going to be taking on community services. And the reason we're going down that pathway is because we know there's jobs at the end of it, and there's strength with our students because they've got a second language as well too. So we're trying to build on where the jobs are, one. So they'll do a course and there's a job at the end of it, that's one thing. But the strength is they've got a second language as well too. And what we've found out through people starting these courses, and a lot of it is women. It is opening up to guys now, but it has been primarily women.

They're going on to nursing now. So all of a sudden they're feeling empowered, they're going hey actually I can do this. I can work and I can study. So it's leading on to really good educational and employment pathways for this group of people. We're also an N.D.I.S. provider as well too. That's just new. When I say new, we just sort of, because N.D.I.S. seems to be chopping and changing all the time. With some of our students that we're able to train, we're hoping to employ them as well too, to work in the N.D.I.S. field.

And we also run an enormous amount of senior citizens programs. I can't even tell you how many there are, there's so many, for migrant senior cits. Some of those communities have been longstanding European communities that have been in Australia for years and years, and some of the other communities are new and emerging communities. And we also have a diversity and disability program as well too that runs programs, so it's migrants with disabilities for people with disabilities. And there's an advocacy arm attached to that as well too. I think that's all of them.

Interviewer: That's a lot.

Respondent: For a small organisation, I can't tell you, even now I meet people, I'll go in, because a lot of people work part time. And I'll go into St Albans and I'll see someone and I'll go, who is that person? And we also have pro bono legal that we have run out of our offices as well.

Interviewer: So you have legal services as well.

Respondent: Yeah, pro bono once a month as well.

Interviewer: You’re really covering a lot of territory.

Respondent: We do cover a lot, yeah.

Interviewer: And it seems that you have, you’re very adept at being able to work with what’s existing already and take opportunities. That’s what it seems like, from having all these different programs as something presents itself, you can really…

Respondent: I think what (SERVICE NAME) is very good at, it’s very grassroots. What we’re really good at is our team come from the communities, so they really understand what’s going on. So I’ll be applying for a grant, I’ll give you an example. I’ll be applying for a grant. In my head, because I’m white, Aussie, (NAME), it’s got my idea and what I see from the outside looking in. But then when we have to get into the nitty gritty details, family violence could be, this Women of the World program, I work with my colleague here, she’s from the Iraqi community. I have to say, how you think this will go? How do you think, if we run a program like this, how does this sound? Am I too full on? Am I too in your face? So you have to always put everything through a cultural lens. The value in having, because it’s not just language. I can get an interpreter, it’s not language, it’s knowing how the community thinks. And there’s so much nuance in that. I don’t understand it. I’ll be honest, I don’t. I need someone to translate.

So there’s strength in having a really diverse workforce is really important. If you want to do genuine work, not just tick box work. Because we can all do tick box work and say oh yeah, we’ve done it. But it can be quite disingenuous. But I can honestly say we work very much on the ground, and it is reflective of the community needs and sometimes trying to weave in some difficult… not difficult, sometimes they’re uncomfortable messages. So those are the programs, yeah.

Interviewer: So that is a lot. The next question is if you can tell me a little bit about how some of those services came about. So was there any specific design models? Where resources are provided? That’s a very big question.

Respondent: So (SERVICE NAME) has been around for 30 years. Historically we’ve been a settlement provider. And the program that I work in, I think historically that was the big program, which is the SETS program. But it’s come under different iterations over the years. Every three years there’s a new rollout with funding. So it changes its name every year. So historically that’s been one of our big programs. The R.T.O., we jumped into that space maybe three years ago and that was…

Interviewer: Sorry, R.T.O.?

Respondent: Registered Training Organisation. So about thee or four years ago, all these private R.T.O.s were losing their licenses because they were shoddy, basically.

And we applied to become a registered training organisation. We actually got a license, which was pretty amazing. And our manager, who has taken this on, she is really experienced, Sue. Especially at a very grassroots level. She did a lot of work in community houses and neighbourhood houses and did training from that point of view, so she's really passionate. But there was a definite skill shortage. Like aging population skill shortage and the government funds what they call the free courses. Which means it's free tuition. There's always a cost. They say they're free, but they're not, the tuition is free.

So we were able to, we thought we're not going to compete with the TAFEs because what we do TAFEs are doing as well too. What sets us aside is this is one of our training rooms, we have no more than 15 people in a class. You go to TAFE, there's 25 people. The classes go for six months, our classes go for 12 months. We also have English language support for people that may be a little bit shaky with their English language as well too. We have tea and coffee here provided, we have a microwave. We're really flexible. Sometimes you might see a Mum, they might have a sick child but you can bring the kid to the class. You know what I mean? We try and be as flexible as possible. Our teachers are here for an hour after class, so if someone needs help with homework or that sort of thing, there's extra levels of support. So we go longer with the classes, but we're setting people up to succeed. We're not going to try and push them through, because we know that language barrier is there. And we're going from strength to strength with that as well too.

And people are getting jobs at the end. It's about training people to get a skill so they've got a job at the end. And we're having people that are just walking into jobs. A whole bunch of particularly women, all of a sudden, who have never had a wage in their life, they've got wages now. They're getting off Centrelink. They're empowered. Their whole world is opening up. It's beautiful. Do you know what I mean? It's really great.

And also now we've got a lot more guys coming in now too. Coming in for the disabilities and for the aged care as well too. And that in itself is a big skill shortage as well too, because caring tends to be a female dominant, where we want guys in there now. So we're getting some guys in there as well too. So that's just also meeting a need, community need as well too. So I don't know how, I think they just saw an opportunity. And when you've got the right people who have got the right level of expertise, they can see where the gaps are as well too. N.D.I.S. was another one. We've become and N.D.I.S. provider because we can just see the needs there. There's such a growth.

Interviewer: Potentially for new migrants, need to access N.D.I.S.

Respondent: They're so underrepresented. N.D.I.S. we have advocates, disability advocates and they are flat out all the time. Because people go to the doctors, they don't understand, first of all they don't understand N.D.I.S. They don't understand the jargon, they'll go to the doctors, the doctors will fill out a form saying no, you haven't got any problems. Even though it's someone they've been seeing their whole life. And people don't have the English to advocate, saying this is what I'm like on my worst day. And our advocates then go back and they

basically represent, go in as a support person and then we're able to get, they're approved for case management plan. They're approved for funding. So we've got this whole big bunch of migrant people that are just underrepresented with N.D.I.S. Because they don't understand it. And even my friends that have been part of the disability, have been recipients of disability with N.D.I.S., just Aussies who are fluent with English, they struggle with it. So it's about trying to meet the needs, but also with us becoming an N.D.I.S. provider, it also means we can then employ people from the community to support people as carers and disability workers as well too. So our growth will be in the R.T.O. and our growth will be in the N.D.I.S. sector. This office here, that's what we'll be filling. So the R.T.O., we've started that here this year, and with the N.D.I.S. we hope to have that more prevalent here as well.

Interviewer: And then the other core work is through SETS, those other programs. So more generally, because obviously this would take a very long time, what are some of the services provided by other organisations? So at the level of say R.T.O., at the level of SETS?

Respondent: There's other organisations. So with SETS providers, so there's Spectrum M.R.C. so we do partnership work with them as well too. So there's Spectrum. There's Arabic Welfare, who's another SETS provider. That's in the north. Whittlesea Community Connections, they're another SETS provider. In the west is SETS, there's My-Care, Spectrum is in the west there too. Wyndham Community, they're the main, they're the ones in the west. And Jesuit Social Services. They're the other one as well too. I know all the managers from all those organisations really well. We all meet.

I think historically there had been competition, I think previously. This is the problems, what happens when you have to apply for a tender, everyone's competing, so you compete for the money. However we often run programs, we refer into each other's programs because we might not be providing something. Someone might come here for something but we know they're providing a service, so we'll refer people across, and vice versa. Sometimes I'll refer across too. So we're pretty generous like that. The managers, we all get along really well. We talk about how are you going with reports, operational stuff. How are you going with that? How's this going? Yesterday I met with Spectrum about a funding round at the moment that we're applying for together. I'm writing up the funding grant. We're working out budgets and all that sort of stuff. So it's collaboration is key. You can't do it by yourself.

Interviewer: So in terms of collaboration, you've mentioned the schools where you do the parent information days.

Respondent: Yeah, we do a lot of work with TAFEs, schools.

Interviewer: You have some outposts, Spectrum. Any of those other service providers that you just said, Arabic Welfare, Whittlesea?

Respondent: We refer into Arabic Welfare. We refer to them for migration advice, but we don't do partnership with them. They're a little bit of a closed shop.

Interviewer: OK, so who else do you collaborate with?

Respondent: Spectrum, Wyndham Community we've done collaborations with. My-Care we refer in. We all refer to each other. I know we refer out, but it's mainly Spectrum that we collaborate in partnership with. Wyndham Community more stakeholder, not necessarily partnership, it's more stakeholder involvement. Depends on what's going and it depends on, just depends. Just depends on their need and sometimes their program, they might be going down a different pathway than what we have, we are as well too. But we do check in, because if we're applying for funding, we don't want to apply and start up a project that's already going. So we don't want to duplicate. So that's the other thing. So we look at either joining, or if there's a point of difference, then we apply for funding and do something with a point of difference attached to it.

Interviewer: And are there any organisations who you choose not to work with? And if so, why?

Respondent: No, we're open to everyone. There's always opportunities, oh yeah.

Interviewer: And are you aware of any services that are needed, that are not available?

Respondent: Services that are needed but not available. My God. I think generally speaking for any new arrivals and migrants, the lack of interpreting services across the board is an issue. If there was one overarching services that isn't available, it's appropriate language support.

Interviewer: Right, I can see obviously how many problems not having language support poses.

Respondent: I've just come back from overseas, I was stumbling just in the supermarket, just trying to work out what's what. That was just me on holidays. Imagine what that's like daily for a person.

Interviewer: That's right, renting a house, getting a phone bill.

Respondent: Concepts.

Interviewer: So are there any services that are over-utilised by newly arrived migrants, and if there are, what do you think the reasons are for that?

Respondent: I think Centrelink is probably over-utilised, and that's because people aren't getting employment as quickly as they'd hoped. And it's, I think that's, a lot of people come here and they just think they're going to fall into a job. And it's hard getting a job in Australia. There's a few things. Language is a huge barrier to getting a job, but it's not just language, it's cultural. Things that we appreciate in Australia in terms of workplace culture, being proactive is one thing, teamwork, approaching your boss when you see there's an issue. You

see overseas, a lot of people, it’s very autocratic, so the boss is the boss and you don’t challenge the boss, you just go, you do your work. You don’t challenge the boss. When I say challenge, it could be something as simple as a questions, you know what I mean? You just do what’s expected. So I see a lot of people that have got high skills and high qualifications from overseas and they struggle to get employment in their field.

Conversely though, I see people that, I think this is a bit of attitude as well too, and expectation. I see people that have come here and haven’t had a great lot of, they haven’t had great skills or great English, and they go into often those lower level jobs because they want to work at all costs. So they see that as an opportunity. With someone who may have had a university degree or had a white collar job before, there’s no way that person will go down to that job there. They don’t want to be there, they want to be back here. They don’t always see that sometimes you’ve got to go down to go up, and that’s a real challenge as well too.

Gender is a big thing as well too. Depending on where people come from in the world will depend on whether they want their wives or the women to work as well too. That’s a real big thing, and that’s something that we struggle with in the north. We’re working really, we’re working a lot with women because the way Australia is, it doesn’t matter who works. If you’re a husband and wife, the expectation is that if one of you doesn’t work, the other one will. There’s no, that’s the way it rolls here. We’re trying to get mums with small kids to really start considering what a working life will look like going forward.

Interviewer: So question five we’ll ask more about income and that, so maybe we’ll pick it up there.

Respondent: Yeah, sorry, so going back to that, Centrelink is one.

Interviewer: And so are there any services that are underutilised, and if so, why?

Respondent: Parents Next I think is underutilised, because Parents Next creates really great opportunities for parents to start their lives again, and we’re working with the communities. Because people, going back to what I was saying about gender, Parents Next provides support for parents that have got their youngest kids under six. So when your youngest child is under six, they want you, if you’re on Centrelink benefits, to basically be looking at getting off benefits and working. And a lot of people don’t want to do that. They just want to stay at home, they don’t want to work. They’re OK, get the money.

Interviewer: So that’s one of the reasons why parents, because people want to stay at home, this program Parents Next…

Respondent: It supports parents to get, and primarily women, majority women, back into the workforce when the youngest child is over six. They don’t see this as an opportunity, they go why won’t they just leave me alone? But they’re mandated to go. Some areas are mandated, some places aren’t. Some people

grab it with both hands and they go, fantastic opportunity. And it is, it's a great program. But not everyone sees it as a great program. So we're going through a cultural shift, gender shift. How many services are underutilised? Parents Next is one of them. I think there's a lot of services which are probably underutilised, but I just can't think off the top of my head.

Interviewer: Also we are going to be speaking to Spectrum, we're going to be speaking to Wyndham Community, so the idea is that overall we collectively get the picture. So this isn't an exhaustive process either. And do you have any methods that you use to measure the effectiveness of the services you provide?

Respondent: Yeah, so our methods are, when we run programs or we have casework, we do surveys with our clients. When we're running programs, we're always checking in with them what they like, what they don't like. We observe body language, participation, questions, going back to that critical literacy, they're asking questions, their involvement. So the questions as well too.

Interviewer: So this is kind of informal or formal?

Respondent: Oh no, we do… depends on the program. Depends on how we're doing. With SETS we're actually mandated to what we call score client outcomes, which is a bit clunky.

Interviewer: I can imagine.

Respondent: Because the way that they've developed this scoring matrix, it's based on a medical model. The way I see it, if you're sick, just say you break your arm. After six weeks, your arm has been in a cast for six weeks. It went from broken, it's healed. I'll give you an example. Or you might be seeing a doctor ongoing for whatever reason you've got. There's very measurable outcomes, whether you've improved or you've regressed or you're the same, do you know what I mean? Very measurable outcomes. In our work, we might see someone once to fill out a form, giving you an example of. Or we might see somebody several times. If you're seeing someone several times over a particular issue, you can measure an outcome from then to then. But often our work, it's really hard to measure really short term. Someone comes in for one thing today, might be to fill out a form. Six months later they come in, they haven't been able to, I don't know, they can't pay a bill, so they need some support around advocacy and creating a payment plan. So we're having to make ourselves measure very… it's what we have to do, we have to measure these things and we have to…

Interviewer: Tick the box.

Respondent: Yeah, and they have an outcomes. So they have this matrix and there's outcomes. So we have to look at the outcome and say how do we get to that outcome? So sometimes it's a little bit trying to put a square peg in a round hole. I can't, I've had to develop my own scoring tools. And even tomorrow with the team we're going to go through it again, do another refresher, because it's really subjective as well too, this matrix. The way I read something and the

way you read something, it could be both viable, you know. It's not one size fits all. It's a bit frustrating. I think settlement services are quite unique. Some things you can measure quite clearly. If you're running a program for ten weeks, you can say knowledge was this then, and it's this now. You can measure that. But it's a little bit different with low intensity casework. It's a bit difficult to measure.

Interviewer; Makes sense.

Respondent: But yeah, we are measuring, we are measuring.,

Interviewer: And finally, this is only question one by the way, it's the longest of all…

Respondent: Oh God.

Interviewer: The others are a lot shorter and you might have covered some already. And can you tell us about any other issues regarding access to settlement services that new migrants are facing, that haven't been mentioned, I guess.

Respondent: I think the best thing you can have with a settlement service in terms of access is having staff that speak the language, and understand the culture. I think that's really…

Interviewer: So is that a barrier from other services, do you think that don't have that?

Respondent: I think if you don't have that, I think your front staff are key to access. You've got to have really good front of house staff. Because they've got to exercise patience. They've got to somehow, even if they don't know the language, got to be somehow able to get enough out to decode what service someone needs. So if you've got poor reception staff, I think that's a barrier. You've got good reception staff, you're on fire.

Interviewer: Excellent. Any other issues regarding access?

Respondent: I think that's, yeah.

Interviewer: Alright, so question two. So the next questions are related to how migrants adjust to Australian culture and society and the kinds of issues and challenge migrants are facing that you see.

Respondent: Oh, language barrier, yeah.

Interviewer: So can you tell us about your understanding of how migrants that you work with understand Australian culture and society or the level of understanding?

Respondent: It's ongoing. I don't think it ever ends. I just think people are constantly learning. Culture. I just think they're always learning, there's always something to learn. And I think a lot of people, you know that saying birds of a feather flock together, so people, when you're from a particular community, people tend to, not always, but people tend to hang out with their family and

their friends, because one they can understand language, there's a familiar… [stutters with the word ending] that word, to that. So there's that.

Interviewer: I wonder how that's going to be transcribed.

Respondent: They're familiar. Yeah, so there's that. I think the younger people are, the more adaptable they are, that's really clear. So especially we see with our youth program we run, the difference in young people that are attending the local secondary college, I don't know, they're just so part of the mainstream. We've got people that are a bit older, say late 20s, and they're attending TAFE, there's a real difference in…

Interviewer: Even just from late 20s to teens or primary school there's a difference?

Respondent: Yeah, yeah.

Interviewer; I thought you were going to say…

Respondent: The other way around?

Interviewer: …50, 60 olds find it…

Respondent: There is, there is, but I'm just giving you the example of young people. So young people, they've gone to English language school, then they're in mainstream local school. I'm just going to talk about gender and opportunities and positive relationships. That's quite, they're quite Aussie and mainstream with those views. We've got boys, older boys, even women who are a bit older…

Interviewer: As in you were saying late 20s?

Respondent: Early 20s.

Interviewer: Early 20s even.

Respondent: Early 20s, they're pretty conservative with their views around, this is just an observation, one group of people, quite conservative around relationships, patriarchal roles, quite patriarchal still. Yeah, just positive relationships there was a whole bunch of stuff. It's confronting, they're quite… and part of that is because that group there, that older group, they haven't gone through high school for many years because they've been away, but you know that coming of age sort of thing, they've sort of missed that because they've been in survival mode as well too.

Interviewer: And the coming of age happening in a much different cultural context, versus as you say, people in high school is happening here.

Respondent: Yeah, yeah, and older people. Older people really struggle too. They feel they're too old to go to school. You might get some of them that arrive, they're 50, 60, 70. We'll say come on, you can go to English language school,

it's free, six months of free school. And they go, I'm too old. Too old. But funnily we run a program with ladies, with women, and we've got some young mums who have got the kids at the local school, at (NAME OF SERVICE) and we've got a bunch of older ladies that are 50, 60, 70 and they are busting to get out of the house. They're socially isolated, we are challenging them with a whole bunch of different concepts. Gently challenging them. And they're eager for information. They're hungry for information, but they're scared of the formal… they just feel they're too old for formal schooling. The formality of schooling, they just go, I'm too old.

Interviewer: It's interesting how you're talking about different age levels having different understandings or adaptability to Australian culture and society. Do you see any different population groups specifically, say women or men, or from specific regions, any difference in that regard, do you think? Or it's individual basis?

Respondent: Yes to both. We find that there are women from overseas that had careers overseas. I'm talking gender now. So there were some women overseas, they were young, they were teachers, they had jobs, they carried their own. So I think there are people that come from certain parts of the world where there's a big push for, I'm not going to say equality, but you can live your, you can have an income, you can have your own money, that sort of stuff. And then there's other people that are very patriarchal as well too. But it depends, just because you come from the same country, it's like saying Australia. Just because we come from Australia, how we think in Melbourne is completely different to Cairns, completely different to Townsville, completely different to Wagga. There's different mentalities. But I think there are threads of, with the Middle Eastern community, and I'm being really broad, there's a patriarchal community there generally. Not 100% but generally yes. With a lot of African communities there's patriarchal communities. However with Liberians they're matriarchal. So I don't want to say the whole.

Interviewer: But it seems you point to some, there's cultural, but then also class as well.

Respondent: Gender, urban, if you're from an urban background, if you're from a rural background, if you're from a refugee background. It's complex. It's really complex.

Interviewer: And so to what extent do you see your clients being exposed to Australian culture?

Respondent: They're thrown into it, aren't they, here? They're thrown into it. How are they exposed? You go to school, you have to learn a new healthcare system, a new income system, educational system, how to go shopping, how to use money. We're very tech, we're high in tech here. Some countries are cash only. We're doing everything on the card.

Interviewer: So I guess it's also an issue of culture, so things to do with values and that kind of thing as well. Are people being exposed to that, or are they I guess retreating to staying within a community with which they're more familiar?

Respondent: Values are such a broad thing. I think on a whole lot of levels, as humans, we have a whole lot of the same values. I think there are. We value work, most of us value work, we value education. We value safety, we value a roof over our head, we value food on the table. Those values I think are pretty broad. When we say value, does everyone value sport like we do in Melbourne? No, but some do. They might value a different type of… it's such a broad question, the values. I just think about, what are our values?

Interviewer: Well it is hard, if you don't see Australian culture as a specific thing, an objective set of characteristics.

Respondent: I think the way, if I'm being me, we try and be accepting of everyone. I think we do try. Generally speaking I think we do, compared to other people. We are open, hence we've got, in Melbourne, especially around here, it's totally multicultural. I think the thing that we're trying to look at, I think there is equality in gender. I think there is disparity there, and I'm not, when I say that, I don't want to say that's not happening in Australia as well too, it's just that we're not talking about it. It's just that this is my job and I'm looking, at the moment that's what I'm looking for. If that makes sense. I'm not looking over my next door neighbour's fence and thinking, is there gender equality? I'm not looking for it there. Because it's my job, we're looking for where are the gaps? What are the things we're not hearing about?

Interviewer: And this seems to be, you've mentioned gender a few times, so perhaps this is one area…

Respondent: Gender's a big one. Gender's big.

Interviewer: …in which, would you say though this is one area in which migrant communities are being exposed to more, we have a patriarchy here also…

Respondent: Yeah, we do have patriarchy here also.

Interviewer: We do have patriarchy, but maybe less rigid, I don't know, it's hard to say.

Respondent: I think do we have patriarchy? Yes, but I work with women here that are from Middle Eastern communities and they buck the system. And they're cool, you know what I mean? They're cool in the way they are. But they buck the system, and they had to challenge some stereotypes I guess.

Interviewer: Once coming here?

Respondent: Some women just want to work. They want to work because they want to work. They want to work because they want to earn money. They want to work because they want a social outlet. They want to work because they just don't want to be stuck at home with kids all day, do you know what I mean? And I'm just trying to think of people I know, I know a lot of people. I can't imagine anyone saying to their husbands, I want to go to work, and their

husbands are going to say no, I don't want you to work. I can't imagine anyone I know having that conversation.

Interviewer: But it might happen from some communities?

Respondent: Oh 100%, it happens with other communities. Not always, but there is that. So gender is a big thing, and I think positive relationships. Consent is another one. Consent is another thing that we're looking at as well too.

Interviewer: Alright. So what are some of the opportunities provided to migrants to practice their own cultural values and practices?

Respondent: They do. They do. What we're trying to do I guess I help them straddle both, an old culture and a new culture. So you don't have to leave everything you've known and loved behind. You can live that. But there's this life here as well too.

Interviewer: Yep, so they have opportunities to do that?

Respondent: Yeah, well people go to church, they cook their own food, they have dances, they have religious celebrations, big weddings, you know. Just your celebrations, I think those things happen pretty frequently.

Interviewer: And so what are your impressions of how these cultural values and practices of new migrants are recognised and respected by the people in their own community?

Respondent: Sorry, say that again?

Interviewer: So what are your impressions of how cultural values and practices of migrants are recognised and respected by people in their community.

Respondent: I think they are, yeah. I think people do that.

Interviewer: So they have the opportunities, but also it's still respected and something you should do.

Respondent: Yeah, yeah.

Interviewer: And what are some issues and challenges around the process of cultural adjustment that migrants may be facing? So specifically cultural adjustment. So you've already mentioned gender, you've mentioned relationships, consent.

Respondent: Gender is a big one. Yeah, gender. Work. Intergenerational is a big thing too.

Interviewer: What do you mean by that?

Respondent: I guess traditionally, kids go to their parents for advice. But they're new here, so the parents are learning a culture. And because kids pick up English

quicker, the power imbalance of knowledge changes. So going back to that child protection example, so you go to your parents and you say I need help with this. And they're going to say I don't know, I don't understand anything.

That's a very broad term. Where kids can go and get information, they'll find information through school, through friends. They're I.T. savvy as well too. And so they can find out information. And that can be disarming for parents, because parents can't keep up with that level of information. And I think Aussie kids, all Aussie kids have got everything. Everything that walks and talks, got all the bells and whistles, and new kids want all the bells and whistles as well too. Not everyone can afford all those bells and whistles as well too, so that can be really, because kids are just wanting to blend in. Parents don't, potentially don't have the money. Don't understand the value maybe as well too. Of certain things. I think intergenerational, I think the younger people, they just integrate quicker.

Interviewer: Alright, question three. These questions relate to migrants' sense of belonging and inclusion in Australian society. So can you tell us about the programs or supports that are available to help enhance and support migrants sense of belonging and cultural inclusion? So specifically the programs.

Respondent: Yeah, so that's what we do. That's a lot of what we do. How successful are we? That's really hard to know. I don't know, honestly. It's so, I don't know how to answer that. When you run a program…

Interviewer: So what are these programs, just briefly?

Respondent: So Women of the World is a capacity building program, the WOW group, OK. Part of that, and we run that out of (NAME OF SERVICE) Primary School, so some of the parents are from (NAME OF SERVICE) Primary School, and (NAME OF SERVICE) is fantastic. They're a gun primary school. What we wanted to do with those mums is to create opportunities where the parents were more one, they were growing a circle of friends, one. So that's a protective mechanism as well too. So they're socially connected. But they also feel like they're part of the school as well too. So we run the programs on a Friday afternoon, so they don't, they come for lunch, we have a nice lunch together. We run an info session, we have a few English language games. We're trying to improve English language.

And then the mums who have got kids, they hang around and they go to assembly afterwards. So we try and get that level of inclusion of there being, they're still at the school, they're participating in assembly. The kids see their parents there, so that's part of the inclusion as well too. We've got mums that go to that program though that are still really isolated, do you know what I mean? So they come to that and they're having a great time, but that's it. And then we've got the older ladies…

Interviewer: Why do you think that is?

Respondent: Part of them are grieving. I think they're still grieving what they've left behind. They're standing, if you've left all your big party of family behind, you're having to make new friends, lack of language, lack of culture lack of money. But it depends on your attitude as well too, because I see other women that are a bit different. They come out, they're chafing at the bit. It's different for different people. Sometimes I think the higher you are, the harder you fall sometimes. Yeah. I don't know, I think there's lots of little steps along the way, and what we do, there's lots of little steps that we help to bring people together with services.

But I don't think… I think about what makes me feel connected. I'm connected to my family. That makes me feel connected, I feel like I'm connected to the gym, I'm connected to a football club. I'm connected to going to the beach at Christmas, I'm connected to going to the snow. That's what makes me feel connected to Australia and I understand all that, and that's all privilege, you know what I mean? But that's what makes me feel connected here. I understand what the food it. I understand what I can say to you, what I can and I can't say, and I sort of can understand your body language. Maybe I can, maybe I can't, but you sort of, there's that you know where you fit in the groove.

But I think a lot of people that are coming here, they're still trying to work out where they fit in the groove. They've got a bit, but it's not always what you know. I don't know, if that makes sense. I hope it does make sense.

Interviewer: Yeah, it does make sense. It does. And so just because this also is a scoping study, so alongside Women of the World, are there, can you name some other programs that help people fit the groove, as you say?

Respondent: Our youth programs do. We've had actually great success with those. So with our Hub Club program, the big thing that the young people, they were all going to school, and so basically they were bored. They had their family at home, they had their church who they were going out with, but outside of that… and they were going to school, but they didn't have any friends. So we had run a program which was a very Aussie, like a bit of an introduction to Australia, but it was very fast paced, lots of games, trust building exercises, a lot of silliness, always food on hand.

Any program, if there's no food, don't run it, because people won't come. And what they wanted to do, a big thing for them was they wanted to improve their English, so we had a pretty sort of hard rule where we try to speak English where we can. If we're not getting the message across then we'll use first language, but we really try.

After 18 months, even less, even after 12 months, some of those young people went on to TAFE. They did Certificates III. So they were at school, then they jumped off and they've done Certificate III. Some people have got jobs. Some people are doing volunteer work. Some have got quite good leadership skills, so they have done some leadership stuff in a voluntary capacity as well too.

That group, after about 18 months, now we're going through a bit of a natural attrition. They've outgrown Hub Club and what we offer. They've made connections with school, they've made connections with services. They've made connections, their lives are busy with other things, mainstream things. That's what we wanted that program to do. Like a stepping stone.

And developmentally with young people, it's like when young people are leaving home or wanting to leave home. They go out and then they come home. And then they go out for longer, but they always touch base with coming home. They're a bit in and out and we're seeing this with our original group. They're a bit older. They'll come in, they still come on Tuesdays. They'll come for 20 minutes. They just come in and say hi, how are you going? And then they take off. We don't see them for another week. So they're slowly but surely cutting the umbilical cord from Hub Club. But now we've got another group of 20 young people that have come in, so we're starting a whole new program with those.

So that's, in terms of do they feel Aussie? I don't know, probably not. But are they more connected? Yes, they are. Are they more confident with navigating the world and services, and being able to call on a friend to say I'm going to an appointment, can you come with me? Yes, they are. There's that level…

Interviewer: So it's about building networks, building connections and networks.

Respondent: Yeah, yeah, networks.

Interviewer: It seems to be a really effective strategy.

Respondent: Yeah, but it takes time. Nothing is fast, it takes time.

Interviewer: And so what are your observations of how your clients meet and interact with people from their own communities in order to maintain a sense of belonging and cultural connection?

Respondent: I think a lot of people, churches, places of worship are a big one. That's very strong with a lot of people from a lot of different denominations and different beliefs. So that's a big one. People go to a lot of weddings. Around here the weddings are massive, like 500, 600 people. Like it's a full on event. The Burmese are the same in the west, it's normal, 700 people to a wedding, the whole, everyone goes. They're amazing, they're just amazing. So I think church is a really big one. Sport, if people are attached to a sporting club as well too. Bahn here, her family, they basically started a whole soccer club. But they've got kids from everywhere going now, it's a really fantastic soccer club. And the same as well too, sport is important. Different sports are important and I think if there's, that's how people are connected. So it's pretty similar to Australia people. We're really connected by sport. And dance is another big important thing as well too.

Interviewer: OK, as in organised dance groups?

Respondent: Just dance, culturally dance is a big part. I see it with all the new communities. Dance and music is really important. It’s a strong part of celebration and culture as well too.

Interviewer: And who are the key people that your clients contact for social and emotional support when needed?

Respondent: Priest, 100% the priest. The priest is a big one. That’s what we’re working on at the moment, to open that up, if you don’t want to go to the priest. Because there can be a lot of shame that goes with that as well too, because the priest…

Interviewer: This is across denominations?

Respondent: Yeah, people go and see, usually people try and work things out internally. Depends on people’s backgrounds, so there might be community leaders in some, so the priests, religious leaders, that’s one group. People might try and resolve things internally with their families. With some people there might be, with some communities there might be religious leaders as well too, where the families might come together too, the heads of families might come together and they’ll try and work stuff out as well too. It’s pretty foreign for people to voluntarily go and seek a counselling service. People might go to the doctor, that might be another one as well too. People don’t traditionally go and see counselling, because counselling is considered you’re crazy, or you go to a psychologist, you’re crazy. We never say the word counselling. We just say there’s services. We talk about the problem and the symptom. Do you want to get rid of that problem? Would you like to talk to this organisation?

Interviewer: Yes, it’s interesting. A colleague of mine is actually undertaking a PhD in exactly that. He’s from Sub Saharan Africa and it’s about Sub Saharan African accessing counselling services, and barriers to that, and the cultural barriers to that.

Respondent: Who wants to go and talk to a complete stranger about your problems? Even now. But most people don’t. Most people want to try and navigate something within your circle of friends. You want to try and work things out locally. Only if it’s, and that’s education. We know that talk therapy helps. But that’s a very foreign concept for a lot of people from overseas. It’s a really western model. I ran a program about the harm of gambling and trying to open up, because we know people are playing pokies and doing all their money and it’s responsible for family violence and a whole lot of stuff, and it’s happening here in the north and everyone’s very [sh noise] about it.

So I did a, we did some education stuff about the church as well too, because the church was saying we can’t tell you how many people are gambling, it’s causing all these problems. So we went and did an info session with Gamblers’ Help, one of the churches. So we’ve gone there with our Power Point and all this sort of stuff, and we’re feeling pretty onto it, and one of the members, one of the people that were there said where in the Bible can you tell me where it says not to gamble? And I went, I was just choking on my words. I didn’t know what to do. But one of the volunteers in the crowd came

out and he said actually, and he's quoted some passage. I didn't really understand what it meant, but it meant you can't gamble, in the way their interpretation was. So they didn't want to hear about Gamblers' Help, they wanted to know where is there a passage in the Bible that says you can't gamble.

Interviewer: Fascinating.

Respondent: It's complex.

Interviewer: Alright, question four. The next questions are about programs that are responsive to social support and improving the health of migrants in Australia. So can you tell us about the types of programs that are currently being implemented to provide social support?

Respondent: Yeah, there's heaps. We've got the WOW group, the Hub Club, Men's Shed, Multicultural Men's Shed.

Interviewer: Education Hub, that was what it was, sorry.

Respondent: Yeah. What else are we doing at the moment? (SERVICE NAME) runs heaps of senior social programs, heaps. They're absolutely invaluable.

Interviewer: And they're for new migrants as well?

Respondent: New and old, doesn't matter. There's heaps. It's really important for the seniors, because we've also got, a big barrier is transport as well too. Which is honestly, that's probably the biggest barrier. If you said I'll pick you up from home, I reckon you'd just get so many more people that would go to things. People just for a variety of reasons, they're not catching public transport. The senior program. And our disability programs as well too.

Interviewer: Disability programs.

Respondent: Diversity and disability programs are really big as well too.

Interviewer: Excellent and how about, that's linked actually, how about health and wellbeing?

Respondent: When people are socially connected and they're having conversations, they're healthier. They're just better. It gives them something to look forward to. The health outcomes, just being socially connected. I think that's a really big thing. The social connections are really important for everyone. And that relates to social connections results in just better health. Just better health. Better health in better mental health, better health in it's a protective mechanism as well too, because people that are socially isolated, I guess they're more vulnerable to things around family violence and things like that. So even just that protective mechanism is really important for people's health as well too.

Interviewer: So in that regard those WOW, Men's Shed, Education Hub, all those things contribute to health and wellbeing. In a way they're health and wellbeing programs, perhaps.

Respondent: Yeah, and with all those, with all those social, those activities, we weave health messages in with all of those things anyway. So if we're looking at traditional health, we talk about healthy foods, we talk about, we invite people in from health services to talk about women's health, healthy foods, why we shouldn't drink coke. We do measurements of what's a healthy breakfast? Kids' lunch boxes. Just exercise as well too, just trying to get people active and exercising.

Interviewer: So you're weaving these health, education, literacy in, but there's not any health programs per se?

Respondent: Let me have a think. We did the, in (NAME OF LOCATION), they had From Slippers to Sneakers, which was a health program. It was for seniors, which was a walking group. That was just a walking group, per se. But health is quite a big element in all our programs, but we do it, sometimes it's strictly health. We might have guest speakers come in and we'll talk about food, diet. In the north we've done a bit around…

Interviewer: Come in to speak where, sorry?

Respondent: So at (NAME OF SERVICE) for example, we'll have someone come in and talk…

Interviewer: From the Education Hub?

Respondent: Yeah, from an education point of view. Food, exercise, because diabetes an high blood pressure is pretty dominant here with new arrivals.

Interviewer: Not just new arrivals actually. The whole population.

Respondent: Yeah, but we tackle them because we want to put on a lens for them. In the west with men's health, they do the same. They'll do some stuff around some sort of general health as well too. They're big… and with the youth programs that we run, there's always exercise. There's games that we play, exercise, because they've just got to burn off energy because they're young and fit and they need to burn off energy. So yeah.

Interviewer: And are you aware of any enablers or barriers that your clients have experienced when accessing these programs? You said transportation is one, are there any other barriers or enablers? Even with the program design itself for instance, what works, what doesn't work for instance.

Respondent: What works is ringing people the day before to say come along to the program. Letting them know, because I'm like, I don't know, will I got, won't I got? We ring them up, tell them we miss them, come on, it's not the same without you. Want them to feel like they're part of the team. So we do that,

that's a good way of getting people to attend. If the weather is poor, you'll have a poor attendance. So in winter the numbers drop because I think people just don't want to leave home. Fair enough. And you can tell if it's pouring with rain, we'll be waiting for people to turn up. Two people will turn up. They've turned up because they've got a car and they're not going to get wet, do you know what I mean?

Interviewer: So weather and transportation are obviously linked as well.

Respondent: Food, you've got to have food, 100%.

Interviewer: You've got to have food.

Respondent: If you don't have food, don't run a program. Just don't bother. And especially if you're going to do something like at lunchtime, people are hungry. And culturally you're sharing food, you're sharing. And after school, if you're going to have an after school program, young people are hungry. You've got to have food. It's not just that, we're teaching, when you're having food and things like that, everyone cleans up together. So it's sharing the cleaning up, it's sharing doing the dishes, it's sending home people with a care package of leftover food. Do you know what I mean? It's all part of that which makes you feel part of. I think it's all those little tiny nuance-y building blocks.

Interviewer: Great. So question five, these next questions are about programs available for migrants to enhance their financial literacy, income generation and managing money effectively. So can you tell us about programs that are available for financial literacy, income generation or managing money?

Respondent: So financial literacy, we just did some stuff on energy efficiency, just recently. That was around info on how to reduce your bills, your electricity bills.

Interviewer: OK, so it would also be managing money I guess as well in a way.

Respondent: Well it's just about being cost effective with energy, because a lot of people from overseas, they don't have to pay for electricity or gas. So when they come here, not only do you have to pay rent, you've got these whopping big bills. Or maybe they did, but they were really token amounts of money overseas. So managing the heater, putting a jumper on inside is a new concept, as basic as that sounds. So the energy efficiency bills are ones that we've had to look at. Financial literacy, we refer people, payment, plans, we look at payment plans for paying bills for people as well too, so they can do little fortnightly direct debits out of their income to pay bills, so they're not lumped with a big bill at the end. That's another thing that we look at in terms of financial literacy. If people are in strife, they've got big debt, we will then refer them to somewhere like Uniting Care or Anglicare, where they have financial counsellors, and we'll refer them in there so they can help them map out things. Map out their finances if it's beyond our scope. Was that…

Interviewer: And anything to do with income generation?

Respondent: Getting jobs, just getting jobs. So in terms of income generation, we try, going back to Parents Next, we encourage people to continue on with schooling because education is what's going to get you off and into work. That's one thing.

Interviewer: So you run Parents Next?

Respondent: No, Parents Next is run on this floor, one of them, they're everywhere. But we work with them, we get them to come in and talk to groups of people that we're working with to talk to those people we are working with to show them these are the opportunities that are available. Do I have jobs that I can give someone? No, no. But we create pathways around education. These are the opportunities that are available.

I did a session, I was working with TAFE recently and we did a, what do you call it? An industry tour. So we hired a bus and we put a bunch of students on the bus and we took them to a construction site, because we know there's jobs in construction. We took them to a nursing home because we know there's jobs in aged care. We looked at the gaps. So we wanted to show them, this is what this sort of construction site, these are where the jobs are at, just an example. We're trying to expose them so conceptually you can see what it looks like. And we did a whole lot of stuff around soft skills as well too. And all that nuance-y stuff that is valued in Australian workplace culture, because people just had no idea about that.

Interviewer: Yeah, wow. And so what kind of financial challenges do your clients face while adjusting to life in Australia?

Respondent: Well the majority of them have to go straight onto Centrelink and they're just dirt poor basically. Some aren't, some are. But a lot of people are struggling to make ends meet. A lot of people don't want to be on Centrelink, they're busting to get a job, but they're just struggling to get an in. And one of the other things that's a little bit frustrating, we really promote volunteering, volunteering as an opportunity to get work. And a lot of people, depending on where they come from, they just don't see the value, they see why would I work for nothing? I should be paid. They don't see the opportunity that you're promoting your skills and yourself. Often job opportunities become available, it's part of networking as well too. So that's a bit of a challenge.

Interviewer: Yeah.

Respondent: So we try and promote that as well too, volunteering.

Interviewer: So the biggest financial challenge is not having any finances, basically.

Respondent: Immediately and it means that kids can't play sport, per se. It means they can't buy a car at the moment. One of the other challenges is, people don't have cars, they don't have access to cars or they don't have a licence immediately. That's another big barrier to work. So we run a road safety program where we're able to fund people for some driving lessons as well too. And when

people do get their, because they come back and tell us when they do get their licence and that, the whole world has opened up. All these opportunities that weren't there before, all of a sudden have become, because they've got wheels.

Interviewer: It does open a lot of, in such a sprawling city as well.

Respondent: Because a lot of people live out, I'm just giving you an example, out Melton, public transport is just, you're out whoop whoop. So if you had to travel from Melton to Sunshine every day, if you had a car, you know, just doing your shopping, oh God.

Interviewer: And so what are some of the culturally specific dynamics that impact and challenge the management of financial demands among migrants?

Respondent: Impacts? Can you repeat that again?

Interviewer: So what are some of the culturally specific dynamics that impact or challenge the management of financial demands…

Respondent: Oh, high rent.

Interviewer: High rent?

Respondent: High rent.

Interviewer: Also in terms of you know, who gets decision, do you have to send money back home for relatives…

Respondent: Oh that happens, yeah, that's another thing. People send money back home. Rent is high, rent is high.

Interviewer: But is that culturally specific though, or is that just generally? This question is really about how…

Respondent: Sending money back home is one. But I think rent is high, and because people are on a fixed income when they're on Centrelink there's not a lot of room to move. And generally speaking, after your lease is up after 12 months, the rent goes up, and people have to move. Because they can't afford that increase. And if they have to move, it costs money to move. You might have to go out further, so then that increases your transport costs to come in. Potentially it could mean that your kids have to change schools, you know there's new uniforms, this and that. So even though you're settled, that's a very real thing, people having to move. Affordable housing, it's a big thing. It's a big, big thing.

Interviewer: And so how do people overcome these challenges? Perhaps sending money back home, or do they overcome them?

Respondent: There's nothing to back them sending money back home. They send money back home. People send money back home. I don't know, they just do.

Interviewer: That's something they don't know how to overcome it, they just do it, it's just a cost they incur?

Respondent: Because they're here and they're safe, and someone's overseas and they're dying and they've got no food, or they've got to pay off corrupt police. Or whatever it is that they've got to do, things that we don't experience here, they know are very real experiences overseas. And it is, for some people when you're overseas, you're in a second country, there's no welfare system, you're not working, you're stuck, you're in limbo and you're in limbo, and people come from collective communities as well too, so there is an obligation. When you're part of a collective community, there is an obligation that you pool your money in. Your money is not just for you, you pool your money.

Interviewer: Is that perhaps one way in which they overcome challenges, if you're part of a community? Is there any financial support for if they do here?

Respondent: Churches are really good at that. The churches here. So churches, I know churches, you send the plate around for the church, the ongoing costs, but then there's like, I'm going to call it a benevolent fund, it's just another fund, and the church will decide. It's usually sending money overseas. It will be for something to do with people overseas. So the churches are really good like that too.

Interviewer: And are you aware of other services or supports provided by other organisations that support clients with financial challenges specifically?

Respondent: Just Centrelink, the Centrelink we send them to them. Anglicare.

Interviewer: What's Anglicare, they have financial counsellors, you were saying?

Respondent: Financial counsellors, Uniting Care, financial counselling. Consumer Affairs if things get a bit messy, if they need advice because they feel like they've been ripped off somewhere. Tenancy Council of Australia, that's another one. Not sort of financial, but if someone's there about some sort of dodgy landlord or rental practice, that's where you'd send people for advice. So it's a little bit hand in hand there. It's legal, but it's always about money. It always comes back to money.

Interviewer: Which leads to the next set of questions about legal services. So question six, this next question, is about programs available to support your clients when they face legal challenges. So can you tell us about programs and supports available to your clients that help with legal issues. About identity, visas, or inviting family members to Australia and things, can you do that?

Respondent: Yeah, yeah, sure. Yeah, so we used to have migration agents here, but we had to discontinue that at the beginning of the year because of financial constraints. So there are agencies out there that have free migration support.

So we refer clients out to Spectrum and out to Arabic Welfare, My-Care. We have a pro bono lawyer, who is a migration agent that comes in once a month and he sees clients as well too. So we can refer them into (NAME) here. Legal, other than migration, if they're not satisfied, because there's huge waiting lists for free migration, we give them, what's the Mara website, which is a migration agent website. We just say you have to choose from there. We can't tell them who to go with. If people have got real legal problems, we'll refer them down to the local community legal centres. So Northern Legal is one. Brimbank Melton Legal is another, West Justice is another. They're probably the main ones that we go to.

Interviewer: And are they, do you have any programs available to support your clients with legal challenges to do with physical violence or other forms of violence, or discrimination, whether that's at home or the workplace?

Respondent: So if it was physical violence, we'd ask the clients what they want to do. So if it's that, we'd be looking at safety planning, do they want to involve the police? That's another thing as well too, some people don't want to involve the police.

Interviewer: And so is this a part of the casework that you run?

Respondent: Yeah, that's the casework, yeah. So when we do casework, even if it was medium intensity, and family violence is pretty intense, we'd be referring them to say maybe a 1800 Respect, that's an agency for example. Women's Health West is another agency that works with family violence. So you refer them into a program that's a little bit one stop shop. Women's Health West is fantastic. If the person is going to leave the family violence situation. So they can help them out financially. They'll have a social worker. It's all wrap around services, doctors, legal, financial. That's what they're geared up to do. So our job is to really make sure those linkages are strong into those funded programs. That's their bread and butter, sort of thing.

And also we'd also, if something was particularly complex, we'd refer them back into what used to be called complex case support as well, too. We could refer them back into a complex case support program, which is run through AIMS Australia for new arrivals as well. So that's another way. But yeah, it would be mainstream family violence, 1800 Respect would probably be the first one. We'd want to know of they'd want to go to the police. We'd help them with safety planning, but then it would be referring out to the services, what they need. Which are really the mainstream services for the whole… what's the other one? There used to be, what was it? In Touch I think it was, In Touch was an ethno-specific family violence program for women. That's a Victoria-wide services. I don't know if they're still operating. They might be.

Interviewer: Alright, so what are some of the key laws and provisions that migrants need to learn when they first arrive in Australia?

Respondent: You need a license to drive.

Interviewer: OK.

Respondent: You have to wear you seatbelt, you kids have to be buckled in. There's a lot around road safety, and we run a road safety program. So I know there's a lot around road safety, that's one. Another thing, stuff around assault. So when I say assault, can't smack your kids, you can't smack each other. Stuff around family violence, we have to talk about that. Just physical, emotional, all of that too, we have to talk about that. Health and safety.

Interviewer: Health and safety as in where, at work?

Respondent: Sorry, I'm just going back to the question, it was health and safety?

Interviewer: No, it was what are the key laws and provisions that migrants need to learn when they first come to Australia?

Respondent: Oh yeah. Kids have to go to school. Just kids having to go to school. Depends on where they come from, depends on what we have to teach them.

Interviewer: So in your opinion, what is the level of awareness of migrants to accessing key legal services and provisions when needed?

Respondent: Ongoing. Depends. Some people don't know anything, some people know some stuff. That's what we do. We promote that. That's what our programs are doing all the time. So educating about what services are out there, what they do, what's available.

Interviewer: So it's very context specific on the actual migrant.

Respondent: Absolutely, and even though we do all these info sessions, one thing I know, one thing I've observed over the years is that information sticks when it's relevant to that point in your life. So I might talk about family violence service, right. They go oh yeah, that's really interesting, but then you forget all about it, because no family violence going on in your life, do you know what I mean? It's not relevant. So you hear it, it's interesting enough, maybe it is, maybe it's not. But then all of a sudden, something's happening within your family, or you know someone. Then it becomes important because there's an issue at hand. When we educate people, there's so much information when you come to Australia. This bombardment of all this new information. And people just don't remember everything, because it's just too much. So I think information just needs to be available all the time. I'm not saying educating people is not valuable, because it's definitely valuable, 100% it's valuable. But people don't always remember, people don't always know what the services are. People are sometimes scared of services as well too. They don't always fully understand what services are available. So I think it's ongoing. It's just ongoing.

Interviewer: So that's kind of linked to the next question, what kind of challenges do clients face when accessing legal services?

Respondent: They're discriminated against, 100%.

Interviewer: Discrimination.

Respondent: Oh my God.

Interviewer: In what ways.

Respondent: The colour of their skin. Police will discriminate because of the colour of their skin. I've seen it first hand. They won't use interpreters.

Interviewer: As in police won't, or community legal centres?

Respondent: No one. Community legal centres are better, but just this thing, not having the right language supports in place. Colour of your skin is a big one. I took a colleague who is African, and as soon as we walked in, I went as her support person, this is a lawyer, she's a lawyer, and they said to me, are you her case worker? My God, her English is better than mine. And that was from a woman. I can't say what I wanted to do to that person, but I was ashamed. I was ashamed and I was embarrassed.

Interviewer: This was at the…

Respondent: The police station. (NAME OF LOCATION) Police Station, shocking. That's a thing, yeah. But community legal centres on the whole, the ones we work with are pretty good. I think young graduate lawyers come with a whole lot of enthusiasm and they're just bursting out of the blocks. They're not jaded.

Interviewer: So question seven, these questions relate to the movement of your clients from one place to another. What are the key reasons for the movement of your clients from one place to another, or from one suburb to another?

Respondent: Affordability.

Interviewer: So not just permanent moving, just in terms of why they would go, relatives living in different areas, or…

Respondent: Affordability is one. Relatives, being closer to relatives. For those people that can afford to buy a house, it will be either rental or affordability, or they can afford to maybe build a home or buy a home.

Interviewer: So this is also asking about just day to day, why would people be saying I'm in Broadmeadows now, I need to go over to Kana, or I need to go over to Sunshine. I need to go over to…

Respondent: Just on a day to day, not to live?

Interviewer: Yeah.

Respondent: Might be work, might be education. They would be the main things.

Interviewer: Work and education, so pretty standard. And are you seeing any trends in mobility at all?

Respondent: As in people moving from one suburb to the next? Oh yeah.

Interviewer: Or even things like economic and opportunities, employment?

Respondent: So in terms of mobility, people moving from one suburb to the next, in the north we're seeing, I guess historically people used to live in Broadmeadows. Now they're moving out to the new suburbs. So it used to be Broadmeadows, Meadow Heights, Roxborough Park. Now they're moving out to Craigieburn, Doreen, Whittlesea. So they're going further out north, affordability.

Interviewer: That's affordability.

Respondent: Yeah, affordability. And not only that, I'll be honest, it's affordability, but those areas have improved. They've got new schools out there. Infrastructure has been put into place as well too. But honestly it's housing affordability. In the west, so everyone used to come to Footscray. Footscray is now gentrified, so no one can afford Footscray anymore. People still settle in Brimbank, but people going from Brimbank to Melton, that's massive, that's a massive shift, and that's around affordability as well. We've had Bhutanese that were living in Glenroy, just down the road here. They've all gone to Melton now. One went and bought a house, then everyone else went and bought a house. So that was around affordability. The same with the Burmese from Werribee, and the Karen, part of the Burmese, they've gone from Werribee to Melton now too.

Interviewer: So Melton is a big area.

Respondent: Melton is hot, Melton is really hot.

Interviewer: Are there much services out in Melton?

Respondent: They're growing, they're growing. Melton Council is fantastic to work with. They're wanting to put in services.

Interviewer: Brilliant. Question eight, so this next question is about migrants' access to literacy and education programs. So can you tell us about services available to migrants in relation to school education for their children, adult literacy programs or any other educational literacy programs?

Respondent: So English language schools are great for young kids, because they give them six months of intensive support. And then after that six months, the kids are, they go into mainstream school. The problem is, the gap between being really well supported and then going into the mainstream, there's a gap. That's when kids can fall down, because they're not getting the support. Parents can't support them with homework, schools aren't, might not, I don't know, can't always support them as well as they need to. Some schools have homework clubs, but that's an issue, that's a huge gap. The transition from English

language school to mainstream school is a really big thing. That's a huge gap. And in terms of parents, new arrivals get 510 A.M.E.P. hours. Some use them all, some don't use them all and they don't always use all their English language hours because they've got caring responsibilities for kids, or for elders.

Interviewer: And 510, if you're from…

Respondent: Refugee or humanitarian background that is, people are eligible for that.

Interviewer: Doesn't seem to me, particularly from the Arabic speaking background for instance, from non-Latin based language, or Roman alphabet…

Respondent: Sudanese, half the Sudanese don't even have a written language. They don't have literacy in their own language. So they have to learn. That's hard, that's hard. If you can write even, what's Arabic? It's right to left, and we're left to right. Yeah, there's challenges. It's six months fulltime, that's what it equates to be. And then sometimes they can get additional hours. I think it's called C, I think you can get another 210 hours, I think, or 200 hours.

Interviewer: What is C? It's an acronym? I've forgotten what it is though.

Respondent: I can't remember what it is. It's another top up they can get as well too.

Interviewer: And so do you have any programs here? You obviously have Education Hub, but do you do anything literacy?

Respondent: We're not English providers, however all our programs, we are doing our darndest to get people to speak English, conversational English. We're making stuff up along the way, I swear. We're becoming little English teachers. We're trying to be anyway, somehow, somehow.

Interviewer: But from the beginning that's something you identified as a key issue, is language.

Respondent: Yeah. One of the things in terms of what's just happened recently, so there's this network of school hubs, so there's a whole bunch of what they call the school hubs network in the north, where schools, they provide support for parents. It used to be playgroups for kids. So mum would drop kids off to school and then they might have little ones, and then they'd have playgroups. They used to be ethno-specific playgroups. Anyway they run a whole bunch of different programs now. At (NAME OF SERVICE) where we are, they were running conversational English because they had all these parents, primarily mums, who wanted to learn English, but they couldn't get to the school because of drop off and pick up times. Childcare responsibilities. So they were running English classes at the school. Now what's happened, thank God, thank God this is really great actually, the school hubs, they've recognised this, it's not just one school, it's a whole bunch of women, and dads as well too. But mainly women, because the dads will go to school and do English where mum is stuck at home looking after the kids. They've recognised that English

language needs to be delivered in a variety of different ways to meet the needs of people. And the school hubs, that's something that they're going to be addressing as well too. So it's just a more flexible delivery of English. With the view that eventually they'll end up going to a TAFE or there will be a pathway. But just trying to get them, to create opportunities for them to be able to practice English language in a different setting. Because childcare is a really big thing.

Interviewer: It's a big barrier to it? Which was the next question about barriers, but I think you've already…

Respondent: Childcare, transport, elder care, looking after other family, people that have got disabilities or that are aged as well too.

Interviewer: And are there any barriers to children to accessing school, university education?

Respondent: Yeah. They have to care for sick parents. Carer responsibilities. And that's particularly sad, it's awful. Someone could have really severe mental health problems, severe physical health problems. That kid will need to stay at home from school to care for that parent if there's no one else there. And then they miss out on school. And I'll be honest, schools don't check up and say where are the kids?

Interviewer: Yeah, right.

Respondent: We've found that out quite a few times. When the kids are in school, the school is all on hands, but if the kids don't turn up to school… So that's a barrier for kids.

Interviewer: For children accessing.

Respondent: Yeah. I think what is good, going back to uni and that, is that we do have pathways. So they can do their certificates, they can grow into a university degree if they can't just jump straight into a bachelor. The pathways are a really good option. So there's opportunity, just taking that way to get there, that's a way as well. But language is a big…

Interviewer: Languages is a big issue as well?

Respondent: Language is a big issue as well too. And the other thing that's an issue is HECS. So this has changed I think in the last couple of years. You used to just have to be a permanent resident to get a HECS loan. Where now you have to be an Australian citizen to get a HECS loan. So if you're a new arrival… so we do heaps around citizenship, filling out citizenship applications and whatnot. You have to be here for four years to apply for citizenship. So that's OK, you do your paperwork, you send that off. It can take 18 months before you do the test, the backlog is that great. So you're bombed out, you can't get HECS.

Interviewer: Can you get fee help? Because that's different from HECS.

Respondent: I don't know.

Interviewer: I think it is. Anyway, so they'd have to pay up front.

Respondent: Yeah, or maybe they can do a pathway and pay less. So even though they'd be on a concession card, you might get a concession rate to do a certificate, do you know what I mean? But you can only get, those are the restrictions. That's a barrier, that's a barrier there. And we push hard, we push hard for everyone to get citizenship. And people want to become citizens, you've got no idea. People really want to become citizens. Want to be fully fledged.

Interviewer: I can imagine.

Respondent: But that's one of the reasons we push it as well too, is the HECS.

Interviewer: Alright, and can you tell us about any special packages or subsidies provided to support educational opportunities?

Respondent: 510 hours for new arrivals is something. Other than that, I think everyone's pretty much on a level playing field. It's just if you're a concession card holder you get concession rates. But that's just the same as the rest of the nation.

Interviewer: And are you able to outline any kind of employment opportunities that you're aware of that are offered to migrants' children when they finish school or university?

Respondent: Nothing specific.

Interviewer: Just the same as anyone else. And how about employment opportunities for migrants more generally? We've spoken a lot about this throughout, but are there any special provisions to ensure their employment?

Respondent: So I know that big contracts, so when there's these big like construction contract now, when they tender, when they put in their tenders…

Interviewer: Is this from government or from even private sector construction?

Respondent: I don't know, so like the tunnel, the Westgate Tunnel and that, I don't know.

Interviewer: That would be private partnership, it would be both.

Respondent: I'm not sure. John Holland is the construction company. And when they do, and then there's other ones as well too. But I know when they put in the tender they have to put in that a percentage of their workforce, they're opening it up to people, they won't discriminate for age, disability, they'll employ migrants, people that have got out of gaol, vulnerable people.

Interviewer: But not discriminating, versus having quotas is something very different. It's easy to not discriminate, or to say…

Respondent: I'm just saying, so they've got to be seen to be, employment is open to everyone. And I think there's a quota, they're supposed to employ X amount of people from certain backgrounds. My understanding is that, so you need to do a bit more investigation around this, my understanding is those really big contracts and I'm talking about construction contracts, there's some clause when they do the tender, they've got to show how they're trying to employ the most vulnerable community members, ie migrants, refugees, people that have got out of gaol and all that sort of stuff as well too. How that plays out in real life, I don't know, but there is a clause. There is something in there. A lot of people though do go to factory type work.

Interviewer: Yes, so in terms of employment opportunities, obviously you've already mentioned that there's limitations in terms of cultural differences, in terms of language barriers. So a lot of people go into factory work, that's one avenue open, are there other kinds of opportunities for migrants generally speaking?

Respondent: Depends on the employers, you know. I know out Werribee, they've employed some farmhand people out in farms, out in Werribee. I know in Shepparton there's been people that do fruit picking, seasonal work out there. I know Victoria Police are trying to employ Africans to join the police force, and people that have got a second language from the police force. And the same, I don't know if it was C.F.A. or the Melbourne Fire Brigade, there was also I think it might have been the C.F.A., they were trying to recruit people from migrant communities to join those services, but it meant they had to go and live in the country. So that was just the fire brigade though. So there's opportunities there. It's like, not an internship, what do you call it? Like a traineeship, cadetship type thing. But that was the thing, they had to go live in the country, and there was resistance to go and live in the country. VicPol were doing a big recruitment there to try and get in particular people from African backgrounds, but not just African, it was migrant communities as well too, because they want people with culture and different languages. How successful that is, I don't know.

Interviewer: Alright, and question nine, overall, what do you think are the key challenges migrants you work with face while adjusting to Australia?

Respondent: English language.

Interviewer: English language is the…

Respondent: Top one.

Interviewer: Excellent.

Respondent: Top one, and just culture, ongoing culture. I think English language and securing employment. Those are the two main ones, I would say.

Interviewer: Alright, question ten, what would you like to see as possible solutions to helping or supporting migrants to adjust to life well, life in Australia.

Respondent: Oh God, it's such a big question.

Interviewer: What are some key things you can point towards?

Respondent: I think going back to the kids going from English language school to mainstream school, there's a really big gap there, where they're really well supported and then they're…

Interviewer: Left to flounder.

Respondent: In mainstream school, I think that's a really big gap there. Kids are very vulnerable.

Interviewer: So more support once they get to a mainstream school.

Respondent: In the transition, yeah.

Interviewer: At least the beginning.

Respondent: Yeah, that's a really big thing. I think that's a really, really big thing.

Interviewer: What about for adults?

Respondent: I think for adults, in particularly men, I really feel for a lot of men who can't get work. I know I've gone on about women and all of this sort of stuff, but I really do feel for men that just can't get work when they get here, for whatever reason. So supporting men into employment. Because this is where a lot of family breakdown can come from. They've gone from working, earning money, their days are full, they'll come here, they think they can get English and then they can just get a job. So I think that this sort of transition into employment, I think there needs to be some support there.

And sometimes they've maybe got skills that they had overseas that aren't here. So trying to help people identify that skills that they had, they could be transferable into another job, and one thing I noticed was I did, we were running a program for people that had high levels of English and that were professionals in their own country. The women who were really well educated, they just said look, you know what? We know we're not going to get that same job that I had before, but they could identify the skills that they had, and they were willing to transition into different roles. They were really quite flexible. Where a lot of the men were just hanging on to their old job, and hanging on, and hanging on, and hanging on, hoping they would just fall into that, the same job as they had before. So they were less flexible.

Interviewer: It's interesting, that dynamic.

Respondent: I found that amazing. All these women, they're just going nup, I know I'm not going to be a principal at a school, so I'm not going to try. But I've got all these skills and I can, they were open to change. Where there was still this resistance to change. Still hanging on.

Interviewer: There's something beyond, sorry, interview, but I think there's even something beyond that even in Australian society about men being much less flexible and much less adaptable, much more rusted on to their gendered identity.

Respondent: 100%, I think so and I'm sure there might be patterns, when Toyota closed down and these big places, people that have been doing the same thing for 30 years.

Interviewer: Or down the Latrobe Valley with the closing of, the shutting down to the State Electricity Company.

Respondent: Yeah, I think there's something in, I'm just saying that's an observation. But I do feel a lot for a lot of men that come here, where women I guess their job has been to raise the kids and look after the home. And that doesn't change when you come here. They're still raising the kids and looking after the home. That is their job, that is part of their identity. They've got that structure. Where men, other than going to school, what's their structure? They're bored to tears, they're frustrated, they haven't got money coming in. What is their identity? So I think there's a real gap there, and I don't know how to fix it. But there's definitely a gap there.

Interviewer: So it seems that employment, education and language skills seem to be the key kind of issues. Excellent. So this is the end of the interview. Is there anything else you want to add?

Respondent: No.

Interviewer: Excellent. So thanks very much for your participation, we actually really appreciate all the information that you've provided.

Respondent: That's fine.

Interviewer: Interview concluded at 1:22.
