# Supplementary material for: The importance of information acquisition to settlement services literacy for humanitarian migrants in Australia
Source: PLoS One. 2023 Jan 6;18(1):e0280041. doi: 10.1371/journal.pone.0280041 (PMC9821785; doi:10.1371/journal.pone.0280041)
Supplement: S1 Data — (ZIP) [file pone.0280041.s003.zip › SP_02_NSW.pdf]

Interviewer: Okay. So we will start the interview. It's Tuesday the (DATE), I'm at (SERVICE NAME). And I will start the interview at 14:28 with the manager. Okay, so for the purposes of this research, whenever I refer to migrants or newly arrived migrants, I'll be referring to people that have arrived in Australia in the last five years and includes refugees and migrants.

Respondent: Good.

Interviewer: Okay? So the first set of questions I've got here are about the services being provided by your organisation that assist newly arrived migrants and refugees to settle in Australia. So could you tell me about some of the services that you provide?

Respondent: Alright. At the moment the organisation is running two pilot projects. One is exclusively on settlement for newly arrived people from migrant or refugee background in the first five years after residence in Australia. This project is funded by the Department of Home Affairs, and unfortunately the limitations of the program excludes asylum seekers. And yeah, we are not allowed to work with asylum seekers or people that don't have a refugee status or a [inaudible – 00:01:51] status of some kind. We're also running a program that we call the (NAME OF LOCATION) Community Engagement Program. This one is funded by the State. And that program doesn't cover the limitation of time. So and it's really good for our clients because it's an easy transition in the same organisation from the initial five years to they continue with it after that.

Interviewer: Oh excellent, yeah.

Respondent: There has been a lot of debates about settlement limited to the first five years. For some people it's not necessary to reach five years, they feel really settled and participating in community life earlier, but for others the five years are not sufficient. Depends on your circumstances, your past arrival, previous arrival situation, education. So it's a little bit difficult to have one of those one-size-fits-all situations in this sense. But I suppose that the government has to have some guidelines and it was decided that five years is not insufficient. For us in our experience, we have our doubts. And this other program that we have now is facilitating that transition.

Interviewer: That's great.

Respondent: In the same environment, yeah. And we also run a program for seniors over 65, and believe it or not, some of those are newly arrived. And it's trying to maintain independence and maintaining people outside institutions. So we provide assistance so they remain independent after retirement and in their senior years, yeah.

Interviewer: So that would be a combination of people from the first five years to longer.

Respondent: To whatever yeah.

Interviewer: Yeah to whatever.

Respondent: So that one doesn't have any limitation also. And we have some newly arrival. It's a program too.

Interviewer: Great, great. And can you tell us how some of these services came about? Now you've already mentioned the funding sources for - but can you add anything to that around how did you come to run those particular programs or…

Respondent: Right. NMA has been providing settlement service since 1889(?) when we become incorporated. So it's 30 years of service. But during those 30 years have been a lot of changes in government policy, migration waves and funding. Unfortunately our organisation being only a small organisation had funding reduced quite considerably, and now we are part of the SSI consortia under the NSW Settlement Program. So the government in 2015 changed their way of providing funding. Instead of going to individual organisations, it was all the funding went to SSI, ASA, main organisation that as a leading organisation and then all the migrant resource centres and organisations such as (service name) become subcontracts.

Interviewer: Oh, okay.

Respondent: So it's a consortia, it's a consortia of 23 organisations, including all the Migrant Resource Centres. And that's what settlement services deliver, the majority of the settlement services are delivered in New South Wales to SSI. They won the tender and then subcontract.

Interviewer: Right. And do you still have autonomy within that…

Respondent: Yes.

Interviewer: …to run what you identify?

Respondent: We have to present a work program. And of course a work program has to be in accordance to the guidelines of their funding. But it was exactly the same before the consortia.

Interviewer: Great, great.

Respondent: So it's not, it's just not, it's not…

Interviewer: Hasn't changed the way you're doing it.

Respondent: There are certain outputs that we have to address, and that's normal in any funding situation. But yeah, we are desperates(?) in the local area and that's respected by SSI.

Interviewer: Great, great. What are some of the relevant services that other organisations are providing in terms of helping newly migrated people to settle in Australia?

Respondent: Well of course we have to start from AMES, the Adult Migrant Education Service. And that's something that they have entitlement to 510 hours of English tuition once they arrive in Australia. For some people 510 hours are enough to have a functional level of English, but for some others it's absolutely nothing. So then TAFE continues after the 510 hours. They can do more English proficiency courses at TAFE. And some of them, those courses are linked to specialised programs. So English for special purposes. So in relation to learning and developing the language, those are the main services. AMES at the very beginning and TAFE after that.

Interviewer: And TAFE, alright.

Respondent: I don't think that the university does any of that. I think that probably they have support in people that are doing courses, but not only with English.

Interviewer: Yes.

Respondent: Yeah. So that's one of our main programs(?) I can say or services that we link all the time. The other one would be health, especially mental health. Because some of our clients come from very traumatic backgrounds, and it's very difficult for anyone to learn a language, understand the system, understand the laws of the country, if your mental health is not there. So we do a lot of work on mental health services and connecting and working in close partnership with the Multicultural Unit at the (NAME OF LOCATION) Local Health District. And we run information sessions, we explain what we mean by mental health and wellbeing, and we try to make people feel comfortable with their own insecurities being in the new country. So if you don't have that peace of mind, you cannot achieve any other ones. So mental health services are vital. And of course employment agencies, Centrelink, everything that you need to be functional in the new country. And we work with all of them because we cannot do it. We cannot do it on our own. One of the things that we have trouble with, and I have to be honest, is with some of the jobactive providers. Because they don't always have an understanding of the limitations of people from different backgrounds.

Interviewer: Oh I've heard this before.

Respondent: Yeah. And it's related to literacy. Because some people are illiterate in their first language, so makes it even more difficult to acquire the second one. And some of the job search organisations are not totally understanding that.

Interviewer: Right, okay. That sort of touches on both the next questions was, can you tell us about who you collaborate with to do that work? So you've really, did you want to add anything to that other than the main ones that you've…

Respondent: Yeah of course. From the organisation…

Interviewer: There's probably a lot, yeah.

Respondent: …from the organisational point of view, we wouldn't be able to perform our job without the support of (NAME OF LOCATION) City Council. Because the Council is providing us the premises for free so we can have office space. And not only that, they have resources and expertise they are always sharing with us, and there is a designated position for multicultural services in Council and we were in close collaboration with that.

Interviewer: It's the only Council in the region that has one.

Respondent: Well there you are. Yeah. And we convene the (NAME OF LOCATION) Multicultural Interagency, so through that, convening that group we are in touch with everybody. Police services and everyone else.

Interviewer: Right.

Respondent: Yeah.

Interviewer: And then the next question was are there any organisations who choose not to work with and why? Well I mean you mentioned the issue with the employment agencies.

Respondent: Yeah but we still work…

Interviewer: But you still work with them.

Respondent: …we still work with them.

Interviewer: But there is an issue there, yeah.

Respondent: It's only that in sometimes we recommend in a very humble way, but we recommend that they provide proper training to the staff on cultural differences, cultural intelligence. Because not everybody in the front counter is aware of issues and it's not until those issues are spelled properly that they become more sensitised. Yeah we work with housing, we work with real estates, with everybody, yeah.

Interviewer: Okay, great. Are you aware of any services that are needed but not available?

Respondent: There is a big need for suicide prevention. In the last year, and this is a community that is not traditionally served under the settlement context and is a Pacific Islander community. They are not considered newly arrived or

migrants because they come through with a New Zealand passport. So it's a different kind of issue, but there are settlement issues with the community. The fact that they come from an English speaking country or to the New Zealand, with a New Zealand passport, it doesn't mean that they don’t have settlements. And in the last year there were about four or five young people suicides with that community and there are no services through there.

Interviewer: After the interview I'll tell you about a program when I was with the (SERVICE NAME), I was working with (SERVICE NAME), and (SERVICE NAME) also put some money in for their project to train up some (NAME) people(?).

Respondent: Yeah we [inaudible – 00:12:34] with that one.

Interviewer: Oh well that's up and running.

Respondent: Yeah, yeah.

Interviewer: So they want to do something out here.

Respondent: Yeah there was none but it was a gap. There was a gap of services.

Interviewer: Yes, yes.

Respondent: And another, from the CALD(?) perspective I think that the local use services are very how can I say, unresponsive. They are not really responsive to CALD issues and they keep saying for example that oh we don't have enough CALD youth. And that's, it's not, yeah it's not…

Interviewer: Or that we welcome everybody.

Respondent: Yeah, yeah.

Interviewer: But it's not…

Respondent: Yeah look…

Interviewer: …it's not actually culturally specific or making an effort to be inclusive.

Respondent: Yeah. And it's really sad because there is so much that could be prevented if those services were properly delivered in an appropriate way to the community would solve a lot of issues

Interviewer: That's right.

Respondent: Not only from the youth point of view but from the family unit point of view.

Interviewer: And another thing, I keep going back to the (SERVICE NAME) work, (NAME) is opening an office soon.

Respondent: He's coming now.

Interviewer: And they are very good…

Respondent: They are.

Interviewer: …at engaging CALD.

Respondent: That's great.

Interviewer: Young people.

Respondent: Yeah we know that he's coming, they are coming, yeah.

Interviewer: So as they soon as they launch, I'd be…

Respondent: Knocking at the door.

Interviewer: …knocking at the door, because they'd be happy to connect in with you.

Respondent: Hopefully yeah.

Interviewer: So are you aware of any services that are over-utilised, like that have long waiting times and that the demand is very high for that service?

Respondent: No. No I'm not aware. Legal Aid. Legal Aid is always, they always help. Sometimes it's a waiting list yeah.

Interviewer: Alright. And on the other side of that, services that are underutilised either because some sort of barrier or people don't know.

Respondent: All the mental health. All the mental health issues because of the stigma attached. People, if you have some sort of mental health issue, you are either crazy or abnormal. So there is not an understanding among the communities about the subtle differences in between being demented or having anxiety or a temporary stress situation. There's no awareness of that. So the services are not utilised.

Interviewer: Okay, thanks. Okay. Can you tell us about the methods that you use to measure the effectiveness of the programs you run?

Respondent: Alright we, under the subcontract we are to report in a program that is used by the government that is called DECS. And in that program that's the official data input that we have to do. It's done every six months and in that particular system, we have an assessment of every individual contact interaction that we have with clients. It's called a score. So you cover pre-intervention and a post-intervention way of recording how the client is progressing. That's from case work mainly. Now, when we are running programs, capacity building programs, we have our own systems of evaluating the program depending of the client worry that we have. Like for

example we have evaluation forms that had only pictures that are old fashioned way but effective way. And then we have forms when people can actually write and tell us how they feel about our program.

Interviewer: Oh good. Great.

Respondent: Yeah, yeah.

Interviewer: And can you tell us about any other issues regarding access to settlement services that migrants are facing?

Respondent: Access to settlement services or to service in general?

Interviewer: Settlement services.

Respondent: Well I don't think that they would have any problem accessing settlement services because usually what happen is that when they first arrive into the country, they are connecting with the IHS program. And then they could be there from six months to one year depending of their needs of their group or the family or the individual. That's an intensive program when bank accounts are opened, school enrolments are taken care, short time accommodation is organised for them. It's intensive. Enrolling in their 510 hours AMES, recognition of private qualifications. All it's done in there. Then they are exited into the settlement sector. So there is one initial and then into the settlement after the six months. And that's when we, they are referred to us and that's when we continue providing information sessions of our local services and how we do case work. We help them to fill forms, citizenship, the whole other area. So after the six months initially intensive, they come to settlement. And I don't think that it would be problems accessing because of that referral.

Interviewer: Right. And in your experience, after that six months period, do people have a good idea of what's available?

Respondent: Not always. Not always. Because then again depends of the individual situation of the newly arrived. For some people that have a very traumatic experience, torture and trauma, they found that in those six months what is done is a very basic enrolling in Centrelink, opening a bank account, getting housing all that sort of thing. But the rest of the community participation, the counselling, whatever is necessary, has to continue after those six months. And that's when it's our responsibility to do that. For other people that are coming to Australia as a migrant or [inaudible – 00:18:51] by choice, there are not many but there are some, is a lot this year(?). Yeah. Yeah.

Interviewer: Okay.

Respondent: And we also work with families of, it's the family of the skilled migrant in some cases. So it's also a different situation in those cases.

Interviewer: Right.

Respondent: Yeah, yeah.

Interviewer: Alright so the next question which you've alluded to already is about how migrants adjust to Australian culture and society. Can you tell us about your understanding of how migrants you work with understand Australian culture and society?

Respondent: I have a problem with migrants and adjusting. Because when you are, you and I understand what we meant. But for a newly arrived person probably implies I have to do whatever I'm told. So adjusting for some people mean to become the norm. And for other people we have a proper balance in between cultural values and religion and the new acquired culture. So it depends of how it's explained to them. Because especially some services have the understanding oh well you have to adjust. And for them adjusting is you become like us. So it is a difficult way to define. But I think that one of the things that may have an impact is the perception of the media on certain groups. The perception of the community influenced by the media of certain groups. And in the last cohort that we received, the Iraqis and Syrians, definitely that was a big thing. Because of the misunderstanding of why they are here. They are here exactly because they are opposing what is happening over there.

Interviewer: That's right.

Respondent: And the media didn't do a good job in providing, and doing a differentiation.

Interviewer: Wasn't explaining anything.

Respondent: If they are here it's because they are trying to run from whatever is happening over there, not because they are approving what is happening over there.

Interviewer: That's right.

Respondent: So that could be one of the biggest barriers that they have too. They have to explain themselves all the time, and some people feel like stigmatised by it. Instead of being proud of where they come from, they feel that is an embarrassment to say I come from here or there. Because of the way that the mainstream(?) perceives that.

Interviewer: That's right. And to what extent is your clients are being exposed to Australian culture?

Respondent: Well once they are walking on the streets they are exposed. Being exposed doesn't mean that they understand the culture, or that the mainstream(?) understand them. So for some communities they become very insular and they become very self-supportive because of that problem. Because they feel not accepted. They feel, yeah separated. And it takes a little bit of time, a little bit of education, a little bit of supporting community leaders and make them understand systems. And help them to realise that Australia is no different to any other country, that you have extreme thoughts and extreme ways of life like in any other country. Thank God we didn't have the violence that other countries do have. But it doesn't mean that every single Australian is going to be welcoming and understanding. But they have laws to protect them and it's our duty to inform them that if they feel discriminated there are avenues and there are ways that they, legal ways that they can use to prevent themselves from that. Yeah so it's, so it's very difficult to give, in anything related to CALD, it's very difficult to give a uniform answer to a question. Because we are talking about individuals.

Interviewer: That's right.

Respondent: We are not talking about…

Interviewer: And it's good to identify all the different scenarios and impacts and things that can happen there(?).

Respondent: And I generalise.

Interviewer: That's right.

Respondent: Because otherwise we will be making the same mistake that the media's making.

Interviewer: That's right. What are some of the opportunities provided to migrants to practise their own cultural values and practices?

Respondent: From our perspective…

Interviewer: Yes.

Respondent: …or the general?

Interviewer: Just from…

Respondent: Well for us for example when we as a group of different services celebrate Harmony Day or Refugee Week, and you've been part of some of those.

Interviewer: Yes.

Respondent: We try to focus on not preaching to the compare(?) really, but try to focus on the positive contribution that every culture and every nationality and every

any(?) group or religion have to the area. So positive images, we celebrate culture in many different ways with food, with dance, with music, with achievements. A couple of years ago asylum Sudanese, sorry a Bhutanese child, or young person got the top score in the HSC in the area, 99.9.

Interviewer: Oh my gosh.

Respondent: So we celebrate that and all that sort of thing. Like for example for our 30th Anniversary one of the speakers was a 22 year old girl that when come to Australia she was only a young person, but she, they already say she'll help her aunties and her parents and everything and now she is doing a double major in law and theology.

Interviewer: Wow.

Respondent: So what we are trying to do is to tell that given the opportunity, people can achieve. So and respecting cultural difference and religion and we put in our Facebook when is Ramadan, when there is Easter, when there is [inaudible – 00:25:44], when there is anything. So culture is celebrated at all levels, yeah.

Interviewer: Great. What are your impressions of how the cultural values and practices of migrants are being recognised and respected by the people in their community? Like in the broader community, yeah.

Respondent: I have to be very careful in explaining my perception. Because we don't have a lot to do with the wider community as a one-to-one okay? So I can only answer that question through the answers of our clients.

Interviewer: Yep that's perfectly fine.

Respondent: Yeah, and that is not a personal perception. The majority of our clients are very grateful to their neighbours because it's a one-to-one relationship that is built up. But once they establish that contact things are working well. But for example when they have to go to the bank or to Centrelink or even to TAFE and it's a big no personal contact they are scared. Because some experiences are not the best. Sometimes they go to the bank or even to the doctors and if they don't have a fluent English they perceive that they are annoying the person behind the counter. And we are trying to make them realise that they are not annoying that person. That person is the one with the problem, not them.

Interviewer: Oh good, good.

Respondent: So but it's very difficult. It's very difficult because when you are tense, you are upset, anything affects you. And if you have a hostile approach you don't come back to those services.

Interviewer: That's right.

Respondent: You feel that are not(?). But to be honest in this area we are very lucky because Centrelink has a Multicultural Service Officer that is amazing. Council has a Multicultural Service Officer. Health has a whole unit, a Multicultural Health Unit. We have refugee health coming here. FACS, sorry what it's called now? The Department of Community and Justice.

Interviewer: Yes, how many times do they need to change?

Respondent: Yeah the Department of Community and Justice, they have multicultural workers and officers. So in this area we are not that bad in that sense, yeah.

Interviewer: Great. Okay.

Respondent: I tell you one thing, it's surprising, the university has an engagement officer. We've been sending the engagement officer invitations to come to the interagency. He came once and never returned. So community engagement from the university is not something that we see much.

Interviewer: Oh.

Respondent: Yeah.

Interviewer: Okay.

Respondent: Yeah. Not a criticism.

Interviewer: No, no, right.

Respondent: Maybe the person is too busy. But yeah not even a participation in Refugee Week or Harmony Day.

Interviewer: Oh, okay. That's good feedback. What are some of the issues and challenges around this process of cultural adjustment that migrants may be facing? I think you've already…

Respondent: Yeah that's what I mean.

Interviewer: …touched on a lot of all that. Yeah.

Respondent: Yeah that's what I mean. Culture is…

Interviewer: If there's anything. I know that, there's that word again.

Respondent: No, no, no, but I mean, yeah I understand. You have to use that word. That which is a [inaudible – 00:29:31]. I think that is individual but the main issue is feeling safe, feeling accepted, and feeling that they have the right to be part of the society. And that's very difficult too, that takes time. Because at the very beginning, especially people coming from very violent situations, whatever happened it's better than where they were. So it takes time to build their understanding of their rights and their obligations of course. Not only

their rights, their obligations as members of the society. And it takes a little bit of time for them to realise that if they are not happy with their job service provider they have the right to change. That they have the right to say no this is not for me. Because at the very beginning that's not something that they even dream of doing.

Interviewer: Right. Oh good, thank you. So the next set of questions are around migrant sense of belonging and inclusion in Australian society. So could you tell us about the programs or supports available that help to create and enhance migrant sense of belonging and cultural inclusion? Now I think you've already touched on a lot of those around obviously Harmony Day, Refugee Week, all those sorts of things and those cultural specific festivals and all the special occasions.

Respondent: Yeah but that's maybe part of the mainstream society. Those events build their confidence and validate them as a different cultural group. But it doesn't really provide an avenue to feel part of the community. That's when you have to have services that are in the mainstream promoting this, and being part of other activities that are not exclusively CALD centre. Like Refugee Week or Harmony Day. It has to be a more integrated approach in that sense too, but we are, we need those Harmony Day and Refugee Week to build the confidence of people, to make them feel proud of themselves so they can come out and be part of the wider community. But I think that a lot of work has to be done in wider community events to be inclusive. To invite this participation. Like for example (NAME OF LOCATION) City Council has a big event, (NAME)(NAME OF LOCATION). That is once a year and a lot of communities participate and there is a lot of promotion. But we are still sort of having a little bit of difficulty getting out of their ghetto sort of situation. Keeping it, we have to become more community inclusive. Be that event, like their spring festival in (NAME OF LOCATION), like expos in TAFE, open days at the university. All that sort of thing has to be more promoted among the CALD communities.

Interviewer: Great.

Respondent: Yeah.

Interviewer: Great. That's good feedback. What are you observations of how your clients meet and interact with people from their own communities to maintain their sense of belonging and cultural connection?

Respondent: That's a really incredible situation. Anything to do with celebrating culture, most of the communities work together and interact together. But when it's a problem they don't want their own community to know. So for example, if there is an issue of the massive violence, they will never go to a councillor form the same culture. Because they don't understand the confidentiality of

the profession, and they feel if this person is from one community, my community will know what my problem is. So when it is, of course if there is an issue of family breakdown or yeah, divorce in the family or whatever, they prefer not to be served. The majority of the people, I'm not generalising, but they prefer external services to their own.

Interviewer: Sure. What if interpreters are being used? Do you think that still, that has an impact, that sort of issue?

Respondent: But that's the problem, the interpreters have to be booked by the service providers. And the client not always know about the availability of interpreters. And then again, when there is an interpreter I think that is essential that the service provider, whoever it is, government or non-government, explain to the person that this is a confidential information. And not always have it(?). And also now, a lot of services have been, are facing a difficult situation because interpreter services it's no longer free for anyone, not even for us. We are, CALD is a specific organisation and we have to pay for using interpreters.

Interviewer: Wow, so is it just health now?

Respondent: This is not free anymore.

Interviewer: For health?

Respondent: Mm?

Interviewer: Just for health providers?

Respondent: We only for health but not for us. So yeah. Health…

Interviewer: It makes it tricky.

Respondent: …but health we provide interpreters for health services, not for anything else. The same with Centrelink. Centrelink has a lot of online interpreters and on the phone interpreters, but only for Centrelink issues. So if you have somebody, a person that is coming here with an issue because they don't know how to renew their driver's licence, we have to call the interpreter and we have to pay for that.

Interviewer: Wow. Wow. Well that's a big barrier.

Respondent: It is, yeah.

Interviewer: Who are the key people that your clients contact for social and emotional support when needed?

Respondent: Usually services like us, and then we refer them to counselling or appropriate support services if there is something that we cannot provide. But they

wouldn't go directly to our counselling services because it's not in their concept.

Interviewer: Right, yep.

Respondent: So referrals is very important. They are very important.

Interviewer: Great. Okay the next questions are around about programs that are responsive to social support and improving health. So you may have answered some of these already. Can you tell us about the types of programs that are currently being implemented to provide social support?

Respondent: By us or in general?

Interviewer: By you.

Respondent: Okay. So we have no specific groups that are running different programs, but some people, or some communities, some community groups at certain point in their settlement process, a certain point in their settlement process they need time to be among themselves. They need a few months of just sitting together and talking without being bombarded with information sessions, with capacity building programs, with all that sort of thing. And unfortunately funding bodies need outcomes. It's outcome based programs. So they don't see or they don't see the value of just a social gathering. It has to be always an outcome. So your outcome is not our measure us unless we put it as a mental health or wellbeing program, they wouldn't accept it. So there is always has to be what are you going to be doing with this group? What is the programs that you're having? Are you running information sessions? Are you delivering courses? All that sort of thing. And sometimes people need time to breathe. Because overload of information is no information at all. And if you are not mentally ready to accept that information, it's difficult. The same, everybody needs employment, but you need to be ready. Because maybe you get employment but you cannot keep it. So learning English, understanding employment laws or personal health and safety and all that sort of thing that the community doesn't have yet is a barrier. And you can't…

Interviewer: Oh there's so many layers.

Respondent: Yeah and yeah employment is important. Yes it is important, but it's also important to maintain it. And in order to get that and to be sustained in an employment to have a steady income that help your family, you need to, you cannot run before you walk. And that's sometimes funding bodies don't understand that. And they want people to jump instead of having a process. Process is so important. It's probably more important than the end result. The process. How do you do it? How do you get there? How do you keep an employment? How do you keep an enrolment at TAFE? How you don't get

discouraged and what is there to support you when the going get tough? And yeah and this is something that we need to get stronger in that. Some of the programs that we do, for example tomorrow we start a program that is an introduction to aged care and it's run by TAFE. Apparently in this area in the next two years aged care will be the biggest employment opportunities. So because of their English limitations of our clients and the financial barriers, we are running this pre-courses, introduction to courses in partnership with TAFE that people don't have to pay. So they have an idea about the course and the certificate will be without having to lose money.

Interviewer: Oh great.

Respondent: So they are usually 10 weeks, 10 week courses, that it gives an overview. We finished one on childcare last term, and this term we are doing one in aged care.

Interviewer: And being bilingual or multilingual is a huge asset.

Respondent: It is a huge asset. And so people that are coming to our groups are participating in that. That's an example. So we also run conversation and English classes. Mainly for people that are not employed, and they already finished their 510 hours with AMES, but because they are not employed and they are not studying, whatever they learn they forget. Because they are not practising at home. So we have two wonderful, wonderful ex-teachers from TAFE that are retired. So they volunteered to run those classes every Thursday here. And it's mainly conversational. Keeping people fluent or getting there. It's not about writing an essay or having a perfect grammar, it's about being functional. And that's another of the groups that we are running. We have a music therapy group. And that group is funded by STARTTS. So people with torture and trauma backgrounds are sort of healing through music.

Interviewer: Wow, yeah.

Respondent: And they, yeah and they are learning their own [inaudible – 00:42:29] music wherever they come from. So yeah, and then we have your information sessions when you have Centrelink come in to explain how to become a citizen, how to open a myGov account. Your basic things so…

Interviewer: Oh great.

Respondent: Yeah.

Interviewer: Great. There's the whole range.

Respondent: Yeah.

Interviewer: And what about health and wellbeing?

Respondent: We work very much in partnership with the (NAME OF LOCATION) Women's Health Centre and the Multicultural Unit in the (NAME OF LOCATION) Local Health District. And we are part of the governance committee in there, so we try to advocate at a higher level. So whatever is done is input in policy. Not just putting a bandage on an individual case. Make the service appropriate. So we do it at one-to-one but we also do it at their level. And the same with FACS and other services, yeah. So our advocacy role is not only as an individual but as a CALD community in general. Yeah.

Interviewer: I think that next question you've already – we might move on. So the next set of questions are about programs available for migrants to enhance their financial literacy, income generation, managing money effectively. So can you tell us about any of the programs you, that are available for financial literacy?

Respondent: I think that most of the charities have programs of financial advice. In this area there was one particular, in (NAME OF LOCATION) there was one particular organisation that was doing financial advice. But the difficulty that we found referring people to that service is that they try to indoctrinate people to one particular religion. So that was a big issue for us. So we are no respect(?) on that area. So if we ever have that, we call Centrelink because it's government, that will be not based on any other ulterior motive. And there is one particular unit in Centrelink that provide a sort of financial advice and how to plan your income and how to estimate how much so you don't get into debt and all that sort of thing. We also once or twice have to contact the Department of Fair Trading because some of our clients sort of fell into the trap of their term(?) free loans. And they didn't understand that they have to pay as they go and not waiting until the three years otherwise the interest will…

Interviewer: Oh right, yes.

Respondent: …will be – because people thought okay I get my goods now and I don't have to pay anything until three years from there. And it's not like that, you have to make normal payments. So yeah, financial advice is provided by Centrelink, is provided by the STARTTS, is provided in some cases by some charities. Yeah.

Interviewer: Great.

Respondent: If we do it, if we need to run a session, we don't do it ourselves, we call up on one of them.

Interviewer: Alright. And what kind of financial challenges do your clients face while adjusting to life in Australia?

Respondent: Alright. Telephone, internet and electricity is something that they don't know how to manage. So a lot of people get their mobile phones cut off because they don't understand that they are in a program, they are in a contract that gives them certain usage. The same with the internet, especially when kids, teenagers use it and abuse it and the parents don't understand that. And electricity is another one that we realise that people don't know how to manage. The amount of people that come asking for support with electricity vouchers is amazing, and especially in winter time. Their electricity bills are $2,000 or, it's astronomical. Because they don't know, they are not told how expensive to run an air-conditioning is.

Interviewer: Right, yeah.

Respondent: For example, or electric, cheap electric heaters. Mainly we see that in winter but some, we had a lady from an African community that had six or seven kids, and she had a bill of $3,200.

Interviewer: Oh my god.

Respondent: Because they all have an electric blanket and they, yeah.

Interviewer: Wow.

Respondent: And coming from a tropical country, they feel the cold even more. And they are not aware of that, yeah.

Interviewer: Great.

Respondent: Another one is traffic fines. Not understanding that they have to pay it on time and all that sort of thing and yeah, it's a big one.

Interviewer: Wow. And are you aware of any culturally specific dynamics that impact and challenge the management of financial decisions?

Respondent: Yes. A lot of communities feel an obligation to send money overseas. Because families are still in refugee camps. Family, close family, brother, sister, parents are still suffering in there and they're very deprived situations. So they sacrifice a lot here to send money. Also because it looks good to send money. I have to be honest, yeah. And some people do it because they want to show that they are doing really well here, but their family is suffering here. So that need to send money overseas is creating a lot of problems in the family units in here.

Interviewer: Right. And how do they overcome these challenges?

Respondent: With time.

Interviewer: With time, okay.

Respondent: With time and with self-experience. Because you can't, you can't say, you cannot determine what is an individual obligation to their family. You cannot say oh you are here and you don't have to send money overseas. No you can't. Because you don't know how that person feel. And sometimes these days survivor guilt that takes into, it take a place in there. Because a survivors and feeling safe in Australia, they feel guilty that their relatives, mum, dad, brother, sister, even children, are still suffering the way they were. So that's survival guilt is a contributing factor to financial difficulties, because they keep sending money. They go without here in order to alleviate the problem over there. And I have to respect that because it's a human, it's human nature.

Interviewer: That's right. Are there any services or supports provided by other organisations to support your clients with their financial challenge?

Respondent: Yeah as I said, look many, many of the charities or many organisations that help, give the vouchers, support them with electricity vouchers or things like that. And they are now also vouchers for water consumption. Because even if you are renting you have to pay for your water now. So there are vouchers for water supply. Always food vouchers from St Vincent de Paul or Salvation Army.

Interviewer: And how do people find out about all these different things that they can get?

Respondent: They come to us and we refer. And then they learn, yeah.

Interviewer: And then word of mouth and…

Respondent: Yeah, yeah.

Interviewer: So a combination?

Respondent: Yeah, yeah. You don't advertise that because it could be a little bit offensive in some cases. But once the client approach us and said I have this difficulty, okay you go there, you go there, you go there. Yeah. We make the bookings for them too, yeah.

Interviewer: Thanks. Okay. So the next set of questions are around support around legal challenges. So could you tell us about the programs and supports available for your clients with legal issues around identity, visas, inviting family members to Australia.

Respondent: That's a big money making exercise. Because unfortunately there is a lot of migration agents that are probably not skilled enough to give, I don't know how to put it without being in any way offensive or making an justice, but some migration agencies that are one of the services, legal services that many of our clients used are not as skilled as others so clients have to do

documents again and pay again. Either for their own migration issues, so to bring somebody here. Traffic fines, driving under the influence is big. Domestic violence situations, but those don't get reported as much as they should be. Coming to us and telling us I am a victim of domestic violence is easy for them, but to report to the Police is not. Legal Aid is something that because of their financial situation is very much, we referred a lot to Legal Aid. But Legal Aid does only certain areas of the law. Like if there is a family law issue, they get referrals, all that sort of thing. So private legal advice is very rarely seek from our client body, because of the cost. So yeah, pro bono services we know that there are certain, especially for immigration advice when you are a migration agent you have an obligation to provide certain number of hours pro bono. So we need to refer, we find out who is in that situation. But legal representation is very expensive. So most of the cases going to the Courts or appeals in the Courts, yeah is through Legal Aid. And sometimes we really have to advocate for the clients to get an interpreter in the Court, yeah.

Interviewer: Right. What do you think are the key laws and provisions that migrants need to learn when they first arrive in Australia?

Respondent: Look one of the things that many people get in trouble with is with tenancy agreements and tenancy contracts. Because it's not explained to them in the fine writing. Their English is not there. And for example if there is a contract for a mum and a dad and two children, that what is supposed to be living in this house. But if the sister had a problem with the husband and she wants to get out of there, she comes to live in there and she brings another two or three children with her, so the household becomes totally different to the one that is in the contract and that's a very common problem. Like for example people that are renting houses and have people living in garages, not knowing that is not a habitat approved room. So for them it's nothing, not a big deal, but it creates problems with contracts. And people running businesses from home like childcare and all that sort of thing is also a problem. Mainly also the competition to get housing is a problem. Because if you have somebody on Centrelink benefits that is from a CALD community and somebody that is not, usually the owner of the property will go for the one that is not. So it's, and I understand they're running a business, they are for profit. And because of the problems of not understanding the contractual obligations, sometimes properties get damaged or they don't get used according to the contract. So there is a reluctance too. Yeah so I think that that's one of the biggest – sorry the question was around?

Interviewer: No, no that's good. It was, we were trying to break it down into some of the, like when they first arrive in Australia and then within the first five years. So that housing tenancy would be…

Respondent: The housing, the traffic law, the yeah, the use of child restraints, all that sort of thing needs to be…

Interviewer: Oh right yes [inaudible – 00:57:00].

Respondent: …yeah needs to be explained and yeah. And school also is a problem because in some cases, especially larger families, when the kids come from home from the school they don't have any space to do homework. And then they get in trouble at school because there are seven or eight brothers and sisters and only one computer and yeah. All that sort of thing. And the parents don't understand the value of homework, not always, so it's a difficult one.

Interviewer: Yeah well that feeds into another question, but that's great. That would be a big issue. In your opinion, what is the level of awareness for migrants of accessing key legal services when they need them? How aware do you think?

Respondent: Well unless they get in trouble, it's automatically they become aware. Because either they are taken into custody or they have to go to the Court. What they are not aware is what services are there to represent them. Sometimes we get people coming to us and said I have to go to Court and said well you don't have time, it's tomorrow, you come today. We cannot book anything for you or that sort of thing. So I don't think that there is a lot of awareness, because you cannot anticipate what is going to happen.

Interviewer: That's right. Unless you have experience.

Respondent: Yeah. And there is not a course on Australian law that you – it's a gap in there. And it's a gap that we cannot fulfil because unless it's individual cases. But I suppose that is the same with the mainstream. Not everybody knows about everything in the law field. So they become aware of Legal Aid when we tell them, or when a relative that been through the same situation. They also, their perception of Police services, that is not always a punitive service. That sometimes it's a protection service. So that is an understanding that is not there all the time. When you say the Police they say I'm in trouble. I don't see this person or somebody that is going to help me.

Interviewer: Yes. I could see, yeah. I hadn't thought of that. That would be – okay that's that set of questions. We're on the last page. We're nearly there.

Respondent: That's alright.

Interviewer: How are we going?

Respondent: That's okay.

Interviewer: 3:30. Oh we've been going for an hour have we?

Respondent: I'm sorry.

Interviewer: No, no, no. Don't say sorry. It's all great information.

Respondent: Okay.

Interviewer: As long as you're fine.

Respondent: Yeah, yeah I'm fine.

Interviewer: Okay. So these next ones are around movement of your clients from one place to another. So what do you think are some of the keys reasons why people are moving from one place to another or one suburb to another?

Respondent: When they firstly arrive, the problem that I mentioned before, actually provide what they call short term accommodation. And when they are exited it's up to the client to find themselves. They are supported of course but they have to find something that they will available to afford. Now, sometimes people move because their personal circumstances change and they need to move from one employment or what-have-you or a study of what-have-you and they have to move from one suburb to the other. In some cases it's because some suburbs are more affordable than others. They offer bigger housing than others, if they have bigger families. Or because that's where the majority of the community goes. You have your doctor that speak your language or your shops when you can buy your spices. That has influence on movements and yeah.

Interviewer: And are you aware of any trends in that sort of mobility, possibly in the early years after migration or after several years?

Respondent: Usually people tend to go at the beginning where their community is. Once they become more independent, economically independent and more aware of services in other areas, they start to move. But I think that they need to be around your own is one of the driving forces for people locations and movements, yeah.

Interviewer: Okay thank you. Now these questions are around education and literacy and you've already given me a great example of one of the issues and challenges. Would you be able to tell us about the services available to migrants using your service, in terms of school education for their children, adult literacy or any other education or literacy programs?

Respondent: Right. The recent cohort, especially from 2017 onwards, had a level of education higher than the previous ones. So all depends of your pre-arrival experience. Because if you were born and growing up in a refugee camp, your chances of having an education are limited. Very limited. But if you are a victim of a recent political situation but you had normal schooling, you are

more able to acquire a better education here. And that's something that we see from adults in relation to children, and from adults in relation to [inaudible – 01:03:34] their own education or being able to practise their professions in the Australian context. For some people that come with university degrees or professionals in any fields, doctors, dentists, engineers, yeah it is very frustrating. Because they find that they have those qualifications doesn't mean that they can work here. And for example for a doctor is incredibly expensive to pay for the examinations to be able to practise. Every exam is about $5,000.

Interviewer: Wow.

Respondent: Yeah. So the same with an engineer. And they have to do, after they get that they have to do the internship or an engineer have to find somebody that will be willing to take it as under some sort of apprenticeship. So it's very emotional draining because they had these. And some of them, we had a case of a doctor that had practised in his own practice for 10 years, then he was serving for three years in a refugee camp as a doctor, and when he came here he has to save money to go to the examinations. And if you miss your exam you have to save the money again. And then if you want to work you have to go to the middle of nowhere to be able to get a position and all that sort of thing.

Interviewer: Oh my goodness.

Respondent: So it's not an easy process. The fact that you are well educated doesn't mean that is an advantage. And the more educated people are, more aware of that what they could be, is creating more anxiety and more mental health issues.

Interviewer: Right, yeah.

Respondent: Does it make sense?

Interviewer: Yes.

Respondent: You understand what I'm saying?

Interviewer: Yeah definitely. What do you think some of the key issues or barriers, and you've touched on this, for children of your clients to accessing school or university education?

Respondent: I think that some communities have an incredible, put incredible pressure, pressure on the kids to achieve academically. And unfortunately not everybody is cut out to be a brain surgeon. So the problem is not the kids or the system in this case, the problem is the parents that feel that because they brought the kids to Australia under very difficult circumstances, either by choice or by refugee situations, it doesn't mean that the kids will have to go

to university and get a degree. I remember before I started this job I was teaching and then I went into a regional position when I have to explain to parents about the education system. And the parents felt that the kids were a disappointment if they didn't take advantages of the fact that they have everything given here. But the kids could do what they could do. Some kids are naughty, I'm not saying that. Some kids don't value the education, but some kids they cannot do it because they don't have what it take. And for some communities, if a child decide to be a plumber or a carpenter, it's not good enough because you have to be a doctor or a lawyer. And that's sad. Because that makes the kids feel bad and made the parents feel bad when there is no need. So I will never remember, forget this, we have an information session and in order to overcome that we make, we investigate how much recent graduate from medicine gets an hour, and how much a plumber gets an hour. And the plumber was getting three times more than the doctor. So until we explain that, they wouldn't see that in order to be successful and to have a good future, it's not always necessary to have a tertiary qualification of that sort. An academic qualification.

Interviewer: Great.

Respondent: Because in their country, sorry…

Interviewer: Yeah, no, no.

Respondent: …in their country if you are a doctor you are great, but if you are a plumber you are nothing.

Interviewer: So it's totally different.

Respondent: Yeah. Yeah.

Interviewer: Can you tell us about any special packages or subsidies provided to support educational opportunities?

Respondent: Besides the study and all that?

Interviewer: Mm.

Respondent: Some universities and some schools, they have what you call, the word in English doesn't come to me. Scholarships. They have scholarships, but I am not aware of any other. Yeah.

Interviewer: Are you able to outline the kind of employment opportunities that are offered to migrants' children when they finish school or university education?

Respondent: Well if they finish university I suppose that employment opportunities will be in their field. Yeah. But when the kids go to high school, they're at a different settlement stage than the parents. So the kids are exposed to the

mainstream culture through school, through their friends, through whatever. So McDonald's, Kentucky, K-mart, all that would be the same like for anybody else. Providing that those services are willing to take them. But the aspirations of the kids will be exactly the same.

Interviewer: What about employment opportunities for migrants more generally? Are there any special provisions to ensure their employment?

Respondent: Well the Department as I said, the funding body for settlement has the three Es as a top priority. English, education, employment. So that's what we've been taught. That's what we have to implement, the three Es. And there are a lot of, as I said there are a lot of programs in there. The biggest issue is who is doing what. Because we are not funded to provide employment services. The jobactive is funded to provide employment services. But most of the active service providers are not doing a very good job with CALD communities. We had a case when somebody needed to have a first aid certificate in order to apply for a position. And that's part of what jobactive has to do, to provide whatever. They receive I think that is $9,000 for every client or something like that. Don't quote me on that because I'm not sure but they have…

Interviewer: No. But it's a good budget.

Respondent: It's a good budget to provide the client with whatever they need to get employment. And we have to argue to the enth degree in order to get this person $270 to go to a first aid course. And that's just an example. Yeah some of them are really going there and every fortnight and fill the papers and do their job searching, but they are not progressing because they are not really supported to progress. In some cases, I'm not saying that is everyone, but in some cases it's just a tick the box and that's it. Because they are for profit.

Interviewer: Yes. And yeah it's happened before hasn't it?

Respondent: Yeah.

Interviewer: Okay. So we're nearly there. Overall, what do you think are the key challenges migrants you work with face while adjusting to the Australian culture and settling in Australia?

Respondent: Face you mean? Sorry I don't…

Interviewer: No sorry. Face. What do you think are the key challenges that they face while adjusting?

Respondent: Well as we said, safety, housing, employment, social contacts, [inaudible – 01:12:31], mental health. Look, migrants and refugees are human beings, and

they have the problems that any other human being will have, aggravated by the trauma and torture that they suffered and the barriers of the language. So any problem that anybody you are 50% more because of all these original barriers. But it's not something that is exclusive to migrants. It's more intensive because of the additional barriers, but the issues are no different.

Interviewer: Okay, great. And finally, what would you like to see as possible solutions to helping or supporting migrants to adjust well to life in Australia?

Respondent: I think that one of the things would be to have, you know in the school setting you have an individual program for an individual child. And of course the children are for a certain period in primary school and for a certain period in high school. But when it comes to settlement, I think that it needs to be flexibility on and some confidence in the professional judgment of the service providers to determine who, when is a person ready to move into the next step.

Interviewer: Right, yeah.

Respondent: Because I understand that some professional or some CALD(?) settlement organisations are looking into clients. How many clients do I have. But because unfortunately that's the way the funding works. You have to present your numbers in order to get funding. But not everybody's ready in five years and you need to keep these people. And people are ready in less than five years and you don't need to keep these people. So the professional judgment of the settlement provider has to be taken into account. So if we said this person is not ready for whatever reason, this person it has to be accepted.

Interviewer: Right. Good.

Respondent: Yeah.

Interviewer: Fantastic.

Respondent: Okay.

Interviewer: Now considering all of those responses in that topic area, is there anything else you'd like to add that we haven't touched on?

Respondent: Well for us as an organisation is the fact that you know, the recent tendance(?) in the government to provide funding to bigger organisations because they consider that is more infrastructure, there is more governance, there is more accountability. So it's always a threat for us to keep losing funding. And it's always this incredible need to prove yourself in competition with bigger service providers in the same field. But we in our experience can honestly say that bigger doesn't mean better. Because for us for example, being in touch with grassroots with the people in the street all the time and

knowing exactly what is going on is something that is a lot easier than for a bigger organisation that have a corporate mentality.

Interviewer: That's right.

Respondent: And unfortunately we are in an economically driven environment when that sort of thing is not always considered. So for us as an organisation it's always a constant struggle to keep the funding going, yeah.

Interviewer: And you know just from coming, being part of your refugee work events or things like that, there's nowhere else you see that sort of community engagement and…

Respondent: Yeah.

Interviewer: …events like that.

Respondent: Yeah.

Interviewer: It's amazing.

Respondent: Yeah because you have to be there. You have to be there at their level, not at…

Interviewer: And they're also at home, like they've got ownership or you know…

Respondent: Yeah and they feel comfortable.

Interviewer: …they've had, it's home.

Respondent: Yeah that's what it is, yeah, yeah. And unfortunately this is something that is not always is considered a fussy approach or something like that, but it's not. Because you…

Interviewer: Really? A fussy approach is it?

Respondent: Yeah, yeah.

Interviewer: Geez.

Respondent: Yeah. But yeah it's not, because people move on and progress so it has to be valued. Not only big organisations can provide a service. There is value in the small community services still. Yeah.

Interviewer: Great. Well thank you.

Respondent: No thank you.

Interviewer: Thank you. Sorry it took so long, but that was fabulous.

Respondent: Probably my fault because normally I talk too much.

Interviewer: No it was not talking too much at all. It's very valuable.

Respondent: Good.

Interviewer: So we'll conclude the interview at 15:46, so that's very long. So thank you very much.

Respondent: Is that a good thing or a bad thing?

Interviewer: It's a good thing.

Respondent: Okay.

Interviewer: Because it's quality. Quality.

Respondent: Okay. That's good.
