# Supplementary material for: The importance of information acquisition to settlement services literacy for humanitarian migrants in Australia
Source: PLoS One. 2023 Jan 6;18(1):e0280041. doi: 10.1371/journal.pone.0280041 (PMC9821785; doi:10.1371/journal.pone.0280041)
Supplement: S1 Data — (ZIP) [file pone.0280041.s003.zip › SP_02_Victoria.pdf]

Interviewer: So (SERVICE NAME). (INTERVIEWER NAME)

Respondent: And (NAME), interviewee.

Interviewer: And (NAME), also participating.

Interviewer: Excellent. Alright. So as I go through, I don't know if you find this useful (NAME) but I did for the first one, just I say question one after each question. I find that helps with transcription.

Interviewer: OK.

Interviewer: When you come to analyse you can see it's going to be [makes noise]. But I don't have to if you…

Interviewer: That's alright. That's OK.

Interviewer: Excellent. So obviously at any time, you know, you can ask questions or if you want to stop. So the first set of questions about the services being provided by your organisation, so (SERVICE NAME), that assist newly migrated people to settle in Australia. I should mention, sorry, that newly migrated people is referring to people within, that have migrated within five years, and that can refer to both forced and voluntary migrants. So could you tell us about the services that (SERVICE NAME) provides to new migrants?

Respondent: Well, because we're a place-based NGO in the City of Greater (NAME OF LOCATION), that's our place, we do provide services into surrounding local government areas, (NAME OF LOCATION), some of that, into (NAME OF LOCATION) and stuff. But our place is(NAME OF LOCATION). And (NAME OF LOCATION) being the most culturally diverse LGA in the country, so 70% of people in (NAME OF LOCATION) speak a language other than English and 53% or 54% of people are first generation migrant or refugee background. So from our position, everything we provide is into those cohorts because the majority cohort in (NAME OF LOCATION) isn't Anglo-Australians, it's culturally diverse Australians. So whilst we're a generalist service provider, our community and taking a place-based framework for service delivery are majority cohort are that group of people. Not always within the first five years, but migrant… people who have arrived here on migrant and refugee visas.

Interviewer: So I guess in terms of settlement services specifically, so the services provided for migrants that have come within the last five years, what services do you provide specifically for them?

Respondent: Well, you know, the way we… and you might be able to sit this one down.

Interviewer: That's alright.

Respondent: So I think it's not as clean cut. Because we would use a framework in thinking through… so you think, it is the that sort of… so if you use that kind of human service, do you know what I mean, where a big part of the pyramid is always universal services, secondary, smaller part, and then tertiary. The process of settling, really if you want to be succinct about it, people are arriving to a secondary needs-based service system and the process of settlement is that process of transitioning from that into the universal services. We deliver services in here and here. But all of those services fall within that remit. You know, so we deliver services through a single service model with common outcomes. So then, so when I say SECL provides a whole bunch of services, we provide a service made up of a whole bunch of component pieces. So in a traditional sense you would say, yes, we're funded by the Department of Homeland Affairs to provide the settlement, the SETS programme. We work on a sub contractual arrangement with AIMS to provide the HSP, Humanitarian Settlement Programme, and then we have a range of primary prevention work that we're commissioned to develop by state government through the Department of Multicultural Affairs and Social Cohesion.

So those would be those targeted secondary services that speak directly to… but of course they feed into all of the universal services that we also provide with financial counselling, housing, material aid, emergency relief, youth, we have a very big youth programme. The Youth Service also delivers some of those component pieces of those settlement programmes.

Interviewer: So all of those kind of financial youth services are also provided to new migrants but they're also provided to the whole community that is in need?

Respondent: Yeah, so they're provided under that universal framework. But our constituents in our place are going to be those cohorts.

Interviewer: OK. Would you have to adapt those sorts of things? I mean, in other words, if you went, let's pick… if you said, and I know (NAME OF LOCATION) is the most diverse, if we went to the least diverse would you say you would adapt the same sorts of programmes to cater for those, to facilitate the settlement activities?

Respondent: I don't think that that… culture isn't… you know, we wouldn't see culture, we wouldn't use culture as the lens.

Interviewer: Yeah, that's alright.

Respondent: As the determinant. But, you know, you have to do things like provide language support and stuff like that. But in terms of how you provide the service, yeah, no, not so much. You know, I think… we use a service framework called The Bridge Social and Economic Mobility

which was developed by a group called EMPath in Boston. So we've been working really closely with them and implementing that as an evidence-based service framework. And yeah, you know, I would be really keen, you know, I would push hard here to not let people use culture as the determinant of what (?), it's how. Really, it's good for how, not very good for what (?), if that makes sense.

Interviewer: Yeah.

Interviewer: Excellent. So the next question was about how these services came about, you've just spoken about some design, the framework that you based it on. And I think you've also mentioned at the very beginning where those, what your target community is when you talked about the diversity in the LGA here. So I think we can move on from that.

[CHAT]

Interviewer: And so what are the relevant services that other organisations provide in terms of helping newly migrated people to settle in Australia?

Respondent: Well, pretty much, everything. You know, if you think about it. Even though there is, you know, the, I think, settlement services as a targeted service, their job is to facilitate that process. People are interacting immediately in both parts of that. So their kids are going to school, they're often being, you know, interacting with the social welfare system, health system. So even though they're arriving to a secondary system, they're participating immediately in that, in the right space, universal. And I think those are really important differences. You know, the difference between targeted, time-limited, entitlement determined by visa versus a universal rights based big part of the pyramid.

Interviewer: So we've talked about like, you know, because again you talked about education. And I know this is probably off the topic, a little bit off but do you think those services are in like education system, I just, we actually didn't include them, like schools. Do they, do you think they have specific things to help facilitate engagement as well or do they just see this, again as you do in similar, this is a universal approach to education and there might be special needs for individual students and groups?

Respondent: I think, well, you know, in Victoria young people from a refugee background have an entitlement to 12 months in an English language school or an adult, and then there's the 510-hour programme. And I think migrant kids get six months in a language school, they transition into mainstream. And I think there's a whole bunch of, you know, main… there's a whole bunch of transition programmes and integration programmes, schools offer all of those things. I think the complexity comes when the determinants of success are not built, you know, aren't built in properly to those programmes. So the kids, you know, fall out

of the system because they can't cope with the linear pathways through those systems. And they add, you know, they add complexities. For schools, I guess it's how do you provide those kind of wrap arounds [indistinct 9.28] within the resources you've got without feeling like you're taking away resources from the rest of the school community. You know, it's always the tension, how much for how many.

So you know, I think that there are particular things that have happened in the last 15 years in terms of refugee communities in particular, you know, so if you look at the sort of between the mid-90s and now, displacement times have gone up from, you know, seven, an average of seven or eight years to, in protracted refugee systems, to 18 plus years. And the human capital implications of that are extraordinary. So, you know, so if you get a young person who was displaced with her or his family at the age of five, arriving in Australia at the age of 17, the implications of that in terms of that process and every other process are enormous. So I think, are we responding well to all of those things? Probably not. You know, it's very different working in, you know, the 90s when the big cohorts coming into Australia were people from the former Yugoslavia where, you know, it was a civil war, people were displaced into neighbouring European countries versus people that are now coming, you know, from Iraq, Afghanistan, eastern African countries where displacements are sometimes now becoming intergenerational, very different, different, you know… again, the determinants, which is that refugee experience, are very different against different cohorts.

Interviewer: Yeah. Big challenges actually. And so can you tell us a little bit about who you collaborate with to provide services? So you mentioned AIMS, are there other organisations?

Respondent: Yeah, AIMS. Pretty much everybody. You know, you're pretty… because if you're doing, if you're trying to do that well you're interacting with all of those services that make up the big part of the base of the service [indistinct 11.51]. You know, so it's the health services, it's the education services, family support services, all of those things that are offered in that base piece.

Interviewer: I guess what we're trying… thinking probably more about specific, not the health system, but other, I guess other non-profit integration organisations. Are there any other particular ones?

Respondent: Yeah, there's the migrant resources centres, there's the English language schools, there's the youth services provided through local government and some of the other NGO organisations that are in our region, housing services.

Interviewer: Yeah, so again those are the government… so you work in partnership with obviously council and government?

Respondent: Yeah, so we work in really close partnership with City of Greater (NAME OF LOCATION). That's our key local government. They provide us with a lot of financial support but we provide a lot of, you know, a lot of that intelligence to the service and capability back into local government in return. So they would be, you know, the key stakeholders for us. And then some of the bigger NGOs, you know, Life Without Barriers, Red Cross. Monash Health have a refugee health clinic which is really good, have been well-developed under the guise of the Victorian Refugee Health Network, which was also by the Victoria Foundation For Survivors of Torture. So, you know, sort of how you progress those core issues across a big health system.

Interviewer: And are there any organisations whom you choose not to work with and why would that be?

Respondent: No. I mean I can't think of any organisation that we wouldn't work with.

Interviewer: And are you aware of any services that are needed but are not available for new migrants?

Respondent: Yeah. I think more than the issue of whether or not people using specific settlement services have sufficient literacy to engage properly with those services. I think it's that whole, the whole pedagogy of settlement that is the… so I think that a better understanding… you know, there's a lack of understanding I think in terms of how people acquire sufficient service literacy in a generalised sense to be resilient in that process is a much broader issue than specifically. You know, because people are pretty… you know, when you come into a targeted service, those services will look, will spend a lot of time finding the service user and engaging those service users in that process. But I think the expertise to let people integrate into those universal services, which are critical, is not well-understood. Sometimes the settlement programme service sees itself, you know, as, I think as the entity. Really, it's just, it had to be a transitional thing, it has to be better integrated. And I don't think the integration points between the settlement programmes and the universal services is broadly… I don't think the interface is good enough, still. I don't think you should mainstream settlement support. You know, I wouldn't think it would be an appropriate strategy to push that secondary services and put them into those. I don't think that would be the appropriate response. But for the much better interface between the two so they're not in the sense of they're competing entities.

Interviewer: Yeah. So I mean the example we used, like for example, with the Migrant Health Centre, ultimately you would want them to transition to just seeing a regular GP rather than just going to that group. So that…

Respondent: That's right. Yeah, absolutely.

Interviewer: Yeah. And there's not a process…

Respondent: No. And time is going to run out, you know, because the provides five years. You know, the process is probably a 10-year process but the government will fund that secondary component for five years. You know, so the HSP runs, I think the SETS programme is at least, is up to five years in… the Humanitarian Settlement Programme provides that case management support for six to 18 months. But they're also, you know, the Humanitarian Settlement Programme is unit cost. It's a unit cost model. And, you know, so I think it's counterintuitive to a degree to structure something like this. So the provider gets paid for delivering certain units of service, which is a bit counter intuitive to the notion… but against an outcomes framework that's looking at the independence and capability of individuals and families to make that transition. But when you pay providers to deliver in service units, they'll deliver service units, and they'll maximise the delivery of service units. So if you get paid every time you take someone to the doctor, you'll take people to the doctor a lot more because you get paid than spending more time on how you build the capability of that family or individual to negotiate that by themselves. You know, so there's a… it is kind of counter intuitive. It's contrary to the outcomes. You know, the intended outcome is a little bit contrary to the funding model, you know.

Interviewer: So it's almost it is that the needed service is almost a transition service in a way, that's what seems to be missing? And also, you did mention also the literacy, which is interesting, what this…

Respondent: I think that, you know, if you… and I, in previous guises, have done work on looking at the levels of service literacy and it can be really disconcerting to talk to people that have been five or six years in country and to talk to them about, you know, even at a population group (?) about their understanding of that bottom part of the base and the knowledge and understanding is really, really low. Critically low, often. Which I think is a failure in both places.

Interviewer: I think also (?) you will find if you ask the general community it wouldn't be necessarily high either, I mean in some…

Respondent: No, it's different cohorts. Certainly, yeah.

Interviewer: In similar types of services.

Respondent: Yeah, and certainly we see people from, you know, Australian backgrounds who have lived here for multiple generations who part of their, part of the problems that they're confronted with is not having, not being able to access critical support at the right time. You know, and then winding up in crisis. Yeah.

Interviewer: And so are you aware of any services that are over-utilised by new migrants? And if there are, what do you think the reasons for that are?

Respondent: Well, I think that the only service that you'll… you know, and again I don't think there's any services that are probably over-utilised. I think there's increased vulnerabilities for different reasons at different times for different people that will spike that. You know, I think there is… you would say that the people on being welfare payments, one after another, remains a critical problem. You know, so when the government, you know, when the Commonwealth looks at the cost of the resettlement programme, which I think is 18,750 for a year for people from refugee and humanitarian backgrounds, you know, they'll run a spreadsheet and they'll say, you know, at the end of a four years period 75% of these people will still be welfare dependent, with a compounding cost. So 18,750 people come in year one and then another… and then… so the single biggest cost which dwarfs everything else is the cost of income support for people. You know, apparent to that problem is the structural changes in labour market in Australia where, you know, really significant decreases in the entry level employment. You know, so I think that's the other thing that's probably changed in the last 30 years is that employment was a great chunk, a great plank in the resettlement process for everyone, migrant or refugee backgrounds.

But the really significant decline in entry level employment and the increase in the demand and technical and professional hours in contrast to the profile of people coming in is making that, is problematising that, the role of employment in settling people or integrating people. Because those, certainly in an area like this which traditionally had a large manufacturing base which has been shrinking, the competition increases between local people and new arrivals for a shrinking, you know, pool of opportunity and long-term unemployment in that group, which then has a whole bunch of flow-on effects in terms of intergenerational opportunity or not. You know, so that's a compounding issue. So I'm not sure that we're being creative enough in how we think about sustainable employment pathways and what they really mean for people with significant displacement issues pre-arrival.

Interviewer: And are you aware of any services that are under-utilised? Yeah, that's right. Over-utilised. Are you aware of anything that's under-utilised by new migrants?

Respondent: Yeah, you know, I think there are always structural, you know, complexities in any of those big surface systems. But I think, you know, our position really would be that a lot of those, you know, the family support services, almost all of those services in our area in the universal service system have been designed and established for white Anglo-Australian communities, where the majority of service use of population is no longer that group of people. So we see under-

utilisation of services by the [indistinct 23.17] or across most of those services for a combination of reasons. One, the service delivery models aren't consistent with what people, how people are going to engage. And two, people's understanding or service literacy is so low it precludes them interacting effectively with those services.

Interviewer: Do you have any specific examples of a kind of, just an example?

Respondent: So I think all of the family support programmes. You know, programmes offered to provide support to families under strain would have really low, we know they have really, really low participation rates in general. Health is probably a little bit better. And then you tend to get doctors showing up within the first 10 years that come from the first language for people to meet a market demand. So a little bit better. I think in that post-compulsory education there would… and then in tertiary education, the participation rates are commensurate with what you would hope and what you would like to see, you know, over a long, over the broader…

Interviewer: And again, is this for new migrants or is this generally? Are you talking generally about the population here in (NAME OF LOCATION) ?

Respondent: Yeah. For new migrants and in both categories, and, you know, and I think the NDIS is really problematic. You know, it's… and I think it's surprising how many people feel like they're not going to get a service in the absence of having an advocate, you know, someone who can do it for them, set up the appointments, negotiate the entry point, deal with a warm referral, all of that sort of stuff.

Interviewer: OK. Alright. And can you tell us about any methods that you use to measure the effectiveness of the services that (SERVICE NAME) provides?

Respondent: Yeah. So we have an outcomes measurement tool that we use. So we have a client data system. So we try to be pretty data driven in terms of how we decide, you know, how we make decisions and what our evidence base looks like. And then we have an outcomes… built into that is an outcomes rating and measurement. So we look across our services, we document the outcomes through our data system.

Interviewer: So what do you define as outcomes? Are they just successfully completed or they've transitioned into other things?

Respondent: No. So we have three common outcomes for across the service, which are built across five indicators and different pillars of our, against those five indicators against six pillars. So it's, you know, so the common outcomes for us are, you know, stability, resilience, and mobility. And then there's a series of indicators and people transition. So our service model is an entry into an early intervention process and then longer-

term support. And people transition from one piece to the other or they don't or they exit and pursue goals themselves. So the case are, we don't really call it case management, but our participant agreements, you know, identify goals and actions that work towards achieving that first level of stability across our outcomes framework and then look at how, then what are the goals that lead to resilience and then mobility.

Interviewer: Excellent. And can you tell us about, I mean you've mentioned quite a few already, but could you tell us about any other issues regarding access to settlement services that new migrants are facing?

Respondent: Yeah. I think that the big ones are, you know, language support, clearly, you know, to people who use their interpreting budgets and provide interpreting. You know, I think a really good example would be, you know, I like to use this one, but, you know, four or five years ago we would, I was doing some training for a large group of midwives and they were saying, look, you know, a problem with a lot of these communities coming into our hospital is that the hospital looks at someone and goes, yeah, but this person's going to need an interpreter, they're going to need a female practitioner, they're going to need all these things. And I said, yeah, but if you change the paradigm and say she doesn't need any of those things, all she needs is an obstetric service, that's why she's here, the hospital needs an interpreter, the hospital needs a female obstetrician, the hospital needs… those are the things the hospital needs. The woman doesn't need any of that, she just needs the obstetric service to which she's entitled. So it's that paradigm that occurs all the way across the board. The schools say the same thing. You know, we can't meet all of these needs. All these additional needs. They're not additional needs, they're actually your needs. The kid and the family just need access to education.

Interviewer: So it's basically they see them sort of, you're suggesting, it sounds like you're suggesting they see them more as costs rather than just the complexity of the service they need to provide?

Respondent: Well, I think the problem is that, you know, people do arrive into a needs-based secondary system and that's appropriate. People get off the plane, they need, some things need to happen. You know, you need a house, you need, and you need orientation, you need all of those things. But that needs-base then stays with people, especially services that have been designed around people like us, the needs-based lens through which those services then see those cohorts stays as they progress into the universal service spaces. And then it's like, well we just provide the same service to everyone because that's equity. But that's not equity. That's nonsense. And you have to adjust what your needs as a service provider are to meet the entitlement of changing groups of people. And that's I think the piece that's not happening very well. Does that make sense?

Interviewer: It makes a lot of sense to me actually. Yeah.

Respondent: So I think that's, from an advocacy perspective, that's our agenda really, is, you know, how do we shift, how do you shift these services to reflect the population and the majority cohort in a place? And that's hard when in Victoria at least, you know, the Department of Health and Human Services, which is the big controller, the big administrator of those services in the main, you know, places 80% of that service delivery in large church-based non-government organisations. They aren't place-based that operate from centralised positions and roll out, you know, generic models state wide or nationally into communities that don't match those processes. And that's problematic.

Interviewer: Definitely. And so the following questions are related to how migrants adjust to Australian culture and society and the kinds of issues and challenges migrants are facing. So can you tell us about your understanding of how migrants you work with understand Australian culture and society?

Respondent: Yeah. I think that, I think it's on, you know, probably on a continuum. I think people from migrant backgrounds generally because the shape of our migration programme is really geared towards, you know, skill shortages and people don't have… you know, so there's a compulsion to work immediately really and you couldn't come here if, you couldn't survive, you know, because the wait time for any Commonwealth assistance on anything precludes that. So I think the integration and that cultural integration happens quicker for people. And also they're coming from a completely different, you know, they're coming out of choice, which is the fundamental difference. I think the things for people from refugee backgrounds are, you know, the acquisition of language and the acquisition of a diverse social capital base can be really problematic for people. You know, and certainly where language… you know, if you look at AMEP, the Adult Multicultural Education, you know, the 510-hour programme, if you look at that the outcomes for different cohorts of people for the last 30 years of that, they're appalling. You know, so 80% of refugee background women are going to come out of that programme… you know, so they have a, they work across the four domains of language, speaking, listening, reading, and writing. And they use that International Second Language Proficiency rating which is a competency-based descriptor with zero being self00evident and I think six is the top, which is a native speaker, tertiary educated with capability and language. Two is functional, and it's also… and three, you need to be speaking, you need to have a language competency of about three to get into a tertiary institution and two to get into a vocational education programme.

So women from refugee backgrounds with significant displacement history which will necessarily mean almost always they have fairly low formal education, the outcomes for people, you know, from those backgrounds is, you know, 80% of those people are going to leave the 510-hour language programme with a language competency of less

than half of what they need to engage in a vocational pathway post that. Well, that's a calamity. You know, it's a pretty… I think it's a pedagogical problem in that you're using classroom-based education processes designed for people with relatively high levels. So you have abstract learning environment stuff. So you know, and the pedagogy is designed for people with reasonably high levels of education and you're applying that to people, you know, who are contrary to that and getting poor outcomes as a consequence. And that's always been the case. It's been the case forever. So I think that the outcomes of the language problems are so low for people…

Interviewer: That that's a barrier to cultural integration?

Respondent: Well, they don't have enough English to do the next piece of any pathway you would design post that, on arrival language programmes. And yeah. And then of course, you know, not having English in a country like Australia precludes so much else in terms of that, you know. Because if you don't move beyond your bonding capital pretty quickly. So if you look at a good settlement case really nine times out of 10 there will be at least one significant relationship that individual or family will have formed outside of that bonding capital space every time. When that doesn't happen, then you get much lower, you get much poorer outcomes.

Interviewer: In terms of, I guess, do they have the ability to… do they understand the Australian culture and society more generally? Because that also could be another inhibitor.

Respondent: Another inhibitor. Yes and no. I think in a place like (NAME OF LOCATION) , you know, the Australian culture is in a state of flux, which is interesting. You know, so as the… I think in Australia that, you know, where you've got the dominant culture, which was defined for a long time by, you know, Commonwealth, white, Anglo identity, which is now… the culturally diverse base of the community is so big now, regardless of policy settings at the Commonwealth, you can't stop it. It's changing. And as those guys die and new identities are emerging… so I think people, I think the interesting thing is, you know, the pushback that people experience because people are also, you know, looking as influencers not just assimilation. You know, so it's not just about, you know, I'm not just going to assimilate, I'm going to ask, if I'm going to change… if I am going to change then I'm going to change you. And that's the space. You know, I think in terms of some people, you know, and I think necessity is the driver. So you know, certainly people that are working, that are doing those things are, have a good understanding. Some communities have become quite insular. You know, like one of the big communities around here in (NAME OF LOCATION) is the Burmese Muslim community. And so I would… you know, their integration points are really low, you know. And there's significant issues. And then their perception of, we're

operating outside of the cultural norms in Australia then pushes them deeper away from that position. You know, so they…

City of Greater (NAME OF LOCATION) has the highest rate of youth unengaged, disengagement for young women age 15 to 24 and the entire statistic comes from young women from that community who leave school at 15, get married at 16. You know, so there's… these are cultural determinants that are, you know, outside of what… they know it's outside of the norm but rather than having… rather than there being a capacity having been built and negotiated that allows them to transition away from those and achieve a better integration outcome, then people withdraw away from, as a form of protection.

Interviewer: And so what are some of the opportunities provided to migrants to practice their own cultural values and practices, I suppose?

Respondent: They're pretty, you know, they're… most communities will have, and I think that's the part that probably also could be thought about more clearly, is the role of ethno-specific community organisations and associations in this process. And even though the SETS programme has a component piece that's aimed at building capability within those leadership groups… the associations in [indistinct 38.50] there's heaps of them. So if you Googled here, you'll find, you know, Vietnamese, Burmese, Afghan, you know, Sudanese, South Sudanese, North Sudanese associations, a plethora of them.

Interviewer: So are they religious-based? They do with sports, they do with…?

Respondent: All of them. But their core interest is really cultural maintenance, you know. It's giving people opportunity to do those things to, you know, to maintain their cultural identity. They often do things like run language schools for children to make sure kids don't lose their cultural language, festivals, dates and holidays of significance to that community. But they're not very good at the cultural transition. You know, so it's about how do you maintain your cultural identity, which is fine…

Interviewer: Yes, they might see it as a conflict.

Respondent: Yeah, and they do see it as a conflict. You know, so there's a natural, well not natural, but a significant tension in there because the lived experience for their constituents is, you know, these really quite significant transitions which are inevitable because if you don't make those transitions then your life will be miserable.

Interviewer: And so you almost kind of responded to this next question but what are your impressions of how cultural values and practices of migrants are being recognised and respected within their community?

Respondent: I think Victoria probably does it better than… I think we do it very well. You know, I don't think we do it perfectly but we've got a state government that's very supportive, has a strong multicultural policy. You know, we've got a Commonwealth government that's I think a bit stuck in some sense of assimilation. They don't have multicultural policy per se, they have a multicultural statement.

Interviewer: Sorry, I should have clarified. How is their cultural recognised and respected within their own communities rather than the broader say Victoria? So migrant communities respect and value their own…

Interviewer: So going back to your community groups, do they see these as important things or do they see them as…

Interviewer: As, you know, a barrier to transitioning?

Interviewer: Yes.

Respondent: Yeah, I think, I think at that point at which culture, you know, culture becomes mixed up with a whole bunch of other things that aren't necessarily culture. So there's, you know, unexamined, unreflected patriarchal value sets that problematise that aren't necessarily cultural, they're not cultural in their orientation but they're maintained and insisted upon by, in any community, a more powerful group for whom benefit is derived from maintaining those structures and processes. That said, those things certainly create tension. But I think a healthy tension if it's supported and managed in the appropriate ways. Yeah.

Interviewer: Alright.

Respondent: But clearly I think that room for, you know, cultural celebration is good and healthy. You know, I don't think that… I think we've been a bit lucky in that there haven't been a lot of bombs go off, you know. When the terrorism stuff goes it creates huge problems for people, you know, enormous. So you know, every…

Interviewer: So we're lucky we haven't had that here in Australia as to what's happened in France?

Respondent: It doesn't matter. Just globally, it's been, you know… when stuff happens, doesn't matter if it happens in France or you know… you walk down, like walk down Sydney Road and all the halal butchers and shops have put a little Australian flag in their window. Like basically saying, please don't break my… don't smash my shop. And women who are… you know people who are identifiable as being from a specific cultural group, you know, find it very difficult to negotiate public space because people, you know, verbally assault them all the time. So in the absence, when that, when that stuff is going on so much, certainly like Muslim Australians and the Muslim communities which are easily identifiable, it creates terrible problems for people.

And I think, you know, it's still pretty tough to be black in Australia regardless. I think, you know, so if you talk to people from any of those groups that come, that are black, there won't be very many days when someone doesn't say something to you still. So that's quite problematic. You know, I think Australia is still pretty, is still a pretty racist country when it comes to black people generally. That's a generalised statement. There's a high level of racism and I think discrimination against people.

Interviewer: So building on that, are there any other… building on this racism, discrimination that you're outlining, are there any other issues and challenges around the process of cultural adjustment that migrants might be facing?

Respondent: Yeah. I think that there's a real absence, you know. And I think it's good to see that there's a bit of a move away from, you know, demand for and thinking about human services in a generalised sense, there's starting to be a bit of a move away from, you know, funding sort of demand driven models, which is problematic, and moving much more towards early intervention and primary prevention. But when you look at the primary prevention and public health sort of frameworks, the penetration into communities, non-Anglo communities is really low. So you get community, you get lags in community attitudes that people then start to align with some sort of cultural value set when really what's happened is that work just hasn't taken place. And family violence is a good example. So you know, in the big health service, their longitudinal sort of community attitude surveys around that stuff, you see significant lags in the attitudes towards, you know, and gender equality as a core determinant in family violence. And you see a lag in attitude around gender equality. But when you look at the work that's happened in mainstream Australian society, for want of a better way of describing it, and then the work that's taken place around that in multicultural communities, almost nothing's been happening.

So the lag is unsurprising, you know, that there would be a lag. You know, so we're developing a primary prevention strategy around family violence and multicultural communities in the southeast corner of state government and that's immediately apparent. You know, there's that, just exposure to those ideas and frameworks is so low right across… you know, you see significant lag.

Interviewer: So it seems it's linked to this failure for universal services to penetrate into new migrant communities.

Respondent: Yeah, absolutely. Yeah.

Interviewer: Alright. So these next questions relate to migrant sense of belonging and inclusion in Australian society. So can you tell us about the programmes and the supports available from SECL that help to create enhanced migrant sense of belonging or cultural inclusion?

Respondent: Yeah. We don't do anything… we don't do stuff I guess specifically other than operate our service in a way where access and equity and the rights-based framework. So that's how we do it, you know. We operate within a rights-based not a needs-based framework at SECL. So that means really, you know, we need to, we position ourselves where people have, you know, are entitled claimants to the things that we offer which is, you know, is a shift in that lens away from… so…

Interviewer: I think we're saying more about, so more about other services specifically, not about… it might be embedded in all the services you do but to talk about, you know, to understand living in Australia.

Respondent: Yeah, we do. So we would run through a whole bunch of our programmes. We run things like citizenship programmes or it might be a six-weeks, because getting through the, you know, actually lodging the citizenship application is a [indistinct 47.30]. So we would run six weeks and we know, so we do it, we have a very, a fairly involved community consultation process we run throughout the year. We engage with our critical communities, the Vietnamese community and Burmese community, the Afghans, we would run our consultation processes. We would then establish a series of community education against what people had identified. And they might be, you know, when the changeover so that, you know, you have to be able to have some level of digital literacy to negotiate your Commonwealth benefits now because you've got to go on MyGov and, you know, that. So we would liaise with Centrelink where we get Centrelink to come in. We would run a series of workshops to try and build the capability of people to interact with what is really critical, you know, and now all of a sudden you can't do it on the phone, you can't do it in… because you have to have a login detail. You have to be able to access the app, you've got to… and try… and try and [indistinct 48.33] the Centrelink for, you know, what's the best way… if they're never going to move away from automated service systems now. There's no point even having that, you know, there's no argument there. It's gone.

Interviewer: So these are more about the actual functional integration but rather than the broader, I don't know what to call it, understanding Australia, I don't know what you were to call it.

Interviewer: Well, I think sense of belonging is a good way of putting it. Like do you feel, you know… new migrants, how do you support them to feel at home I suppose and feel a part of the broader society?

Respondent: Yeah. I mean we would… we would collaborate with… so we would auspice, you know, we probably auspice half a dozen smaller community groups who would help them raise the funds then we would auspice the grants so that they can run various programmes within their community. I think about five or six, you know, $3,000-5,000 grants that we would auspice. We do work in project management and grant

writing workshops, project management, that sort of stuff. There would be a whole host of community activities we would run around how you do certain things, you know, practical sort of courses, short courses, maybe five/four/three/two sessions that people would come to to interact. We also do a fair bit of work in sort of codesign. So we do, we're running two strategies with about six communities where we're… one's codesigning a family, what does family support look like? And there's I think 12 people on that codesign group. It's about a 24-month programme to design a family, what family support should look like, and they're working with Indian, Afghan, Tamil, and Sri Lankan communities. And there's another group working on women and economic inclusion and participation and that's with the Rohingya and Burmese women, four groups of women. So codesigning education and participation pathways, what does it mean, how does it look. And I think that stuff makes a big contribution.

So rather than saying, we don't know. All we know is that, you know, the unemployment rates in these cohorts is 90%. And if you're going to be on a Centrelink income you're going to live in poverty. So we don't know what's the best… we don't know the solution. So we engage large, you know, codesign groups and pay them to participate, almost a consumer participation kind of model, to work with us to design either a change process for the supports that are in place or what is a new process that should be in place to facilitate. And I think those are the things that make people… you know, participation is the thing. I think there's enough ethno-specific infrastructure that supports that cultural maintenance stuff. I don't think that's a problem. But I think what makes people feel at home is that participation more broadly. So for us, it's how do you facilitate and support participation rates across different domains in the community? Social, economic, and cultural domains.

Interviewer: And so what are your observations as how your clients meet and interact with people from their own communities in order to maintain their sense of belonging and cultural connection?

Respondent: Yeah, I think it's very varied. You know, so in some communities those community associations are very strong. And people… you know, because communities have got to be stratified just like any other community, class, subpopulation groups, gender, all those things will stratify. So… but a lot… you know, so like the Vietnamese community, the Burmese, they have a whole… there's a lot of really well-evolved community associations that are pretty high functioning. You take a group like the Afghan community, which is stratified, you know, on class, which is linked to ethnicity. So you know, they've got Pashtun, Pashtun's who were the traditional ruling class. And you've got the middle group and then Hazaras who are always at the bottom of the… and Tajiks in the middle and Hazaras at the end. So they become… the community here then becomes stratified on, along those lines. But then there's also gender and then there's also political. So

there were people that supported the Mujahideen and people that were engaged and working and supportive of the Soviet backed regime between 79 and 89. And so all those things mean the Afghan community associations don't tend to be very much… people cluster more in family and broader sort of clan groupings and the associations, whilst there's a tonne of them, they don't tend to be very functional. People don't tend to identify much with them, as much.

Interviewer: And… yeah, interesting.

Respondent: So it's really a huge variance. And some people will see a necessity to move out of their cultural context to succeed and others will see staying within, you know, residing within that kind of bonded capital space as sufficient. And some of that will be age. Like what's my aspirations in Australia? My aspirations are my kids get a good education, get a good job. But my own aspiration is, you know, just to achieve those things. You know, I'm not very interested in… I'm not going to get a whole bunch of Australian friends, I don't really want to learn how to play footy and cricket. You know, I think there's a huge variance. You know, there's a woman on our board, she's great. She heads up the junior AFL competition for all Victoria. But she came from Malaysia. She's a Malaysian Chinese. And she does this great presentation, it's just brilliant. So you know, her parents came and her mum was like, right, you've got to play sport. We're here, we've got to assimilate. You know, if you're going to succeed you have to play sport. And then we got a buy a car, we got a Kingswood station wagon. This was in the 70s, grew up in Noble Park, you know. And then she goes, you know, every weekend we would go and stay in a country town somewhere in a pub until… so we could get a sense of that. We ended up… she goes, everyone's got a dog, we got a dog. She's just got these slides, you know. First a picture of the four them, she and her brother and mum and dad. With the kids, with this great big dog. And then the last one's the Kingswood, the end of the Kingswood station wagon carpet and all those stickers people used to get. Mildura and Swan Hill and [indistinct 56.00] and all those places. So you know, I think…

Interviewer: So it's from cultural group to group and also in between families even?

Respondent: Yeah. You know, really big variation on… and again, you know, whether you come from a big community that's relatively and numerically quite large and so it has the ability to provide, you know, to… or you come from a community that's quite small. And I think… so I don't think there's really… I think it's varied and complex in terms of trying… you couldn't really make an analysis on a population kind of thinking, except when things happen that change the narrative, the perceived narrative in the broader Australian community around attitudes to new arrivals. Which happens all the time. You know, so… and there have been some terrible and unfortunate incidences. So our Immigration Minister saying things that then force that contraction back in again because people become quite anxious.

Interviewer: And so who are the key people that your clients contact for social and emotional support when needed? Family or relatives or community leaders, religious leaders?

Respondent: Yeah. I think… I think probably family and friendship networks would be the principle one. It was interesting because we did have this discussion with that codesign group in the family support services and someone had asked, you know, whether that group should have, you know, Imams and people from the temples and stuff involved. And the group's consensus was, no, they've got nothing. They don't have anything, they don't have nothing… they don't have anything to offer this conversation. Again, because it's about the maintenance of something rather than the transition process of something. So you know, those guys tend to be pretty fixed in what they see, you know. Be a good Muslim, be a good Hindu, be a good… be a good this. Be a good Afghan, be a good… be a good Vietnamese. You know, you're losing your culture or you're letting your kids lose their culture. Or how could you let your daughter marry that bloke, that Aussie bloke. You know, that stuff. So there was all those tensions. So again, I think that there's a lot of diversity in how and where people go to get that critical support. But you would think it would be, it would move out. You know, people would… people's, I think people's family, where they've got it, here and offshore, it remains a really critical, around advice and support. And then friendship networks that are probably within your community. And then you would hope that broader range was certainly what people will acquire, relationships, critical relationships in that kind of bridging capital space. And then, you know, it becomes available. And because you really need it.

Interviewer: Yeah. And so the next question is about programmes that are responsive to social support and improving health of migrants in Australia. So can you tell us about the types of programmes currently being implemented that provide social support and that provide health and wellbeing? So that would be here.

Respondent: Yeah, so Monash Health is the big one out here. And you know, I think…

Interviewer: We're thinking about your organisation. So your organisation. Do you guys have any specific programmes?

Respondent: Around… well…

Interviewer: So health and wellbeing.

Respondent: Health and doing, being able to identify health issues, mental health, physical health issues are a key component and then being able to facilitate access to… we don't provide the services but we would act as

a conduit between the existing services and all of those, you know, around primary health and mental health supports for people.

Interviewer: So that would be something that as you interact with them you might identify issues and then you would direct them? So there's not a programme per se, it's just basically…

Respondent: We're not a… we don't provide a counselling service at the (service name), no. But we would, but our… you know, the people delivering the service that they would make their assessments, identify and negotiate and then work with people to move into the appropriate supports and programmes to meet those identifying issues.

Interviewer: OK. Yeah. So… and other kinds of services would be, you run financial services, I saw you had some stuff out there about financial literacy. What kind of programmes do you guys have in terms of dealing with financial issues?

Respondent: Yes. So we have a large financial counselling team here. We have about 10 financial counsellors across the region and they would have a large multicultural cohort that is accessing that service. We also… and then we also provide a lot of… we have a microfinance programme. We facilitate NILS and step up loans, which are no interest loans and low interest loans, slightly larger. And they're great. They're also a good tool for us in trying to disrupt people's utilisation of pay day lenders and that really crook after pay, you know, all of those crap High Street, bogy credit products which are… and rent to buy schemes. And these communities are particularly vulnerable. Because if they don't know… so you know, what do you do? You're going to notice some school saying, you've got to pay all these voluntary fees or your kid won't be able to do swimming and all these things. I need $300. So they go down the road and borrow it at 30% interest, you know. Well, if we can turn a NILS loan around so that way they do a lot of community education and support to engage communities. Because for us it's a critical disrupter to trying to get, to break that nexus between people and those really poor credit products and much more appropriate products available. So we have that. We do a lot of work in financial capability and financial management.

We have large, quite a large, we call it community wellbeing, which is basic crisis support. People come in and can't, I don't have any money to buy food today. So you know, we would have about three and a half thousand people a year would come to our service as a… you know, would access our service through that door. And again, there would be a reasonably high density, around 58-60% of that cohort will be new arrivals, first five years. So it's high.

Interviewer: And so what are the key financial challenges that clients face when adjusting to Australia?

Respondent: Unemployment.

Interviewer: Unemployment.

Respondent: Unemployment. Which then, you know, just cascades down, you know, as the higher cost of private rent. So people, you know, (NAME OF LOCATION) was for a lot [indistinct 63.31] was a really high, you know, really high rates of primary settlements. People would get off planes and come and settle in (NAME OF LOCATION) . But now people, (NAME OF LOCATION) 's rate of new arrivals is going down like that and people moving into (NAME OF LOCATION), which is the next LGA out. And so… and then buying or renting in (NAME OF LOCATION) because (NAME OF LOCATION) 's becoming too expensive. But they'll still come to (NAME OF LOCATION) because (NAME OF LOCATION) is where all the infrastructure is. The cafes, the market, you know. If you go to (NAME OF LOCATION) you go down(NAME OF LOCATION), the entire street's Afghan restaurants, dry goods shops, you know. There's an Afghan supermarket on Lonsdale Street, the main street in (NAME OF LOCATION) . So people will come from (NAME OF LOCATION) but they can't afford any longer to rent. So yeah. I think, you know, it's unemployment. It's just…

Interviewer: You mentioned also about understanding sort of these other new products as well. How does that… is that one issues? Just when you were talking previously.

Respondent: Oh, the NILS and those loans?

Interviewer: No, not understanding the problems with the other sorts of systems that exist. Not understanding like, you know, you go and borrow and it's…

Respondent: Yeah, it's terrible. Yeah. And I think it ties back in more broadly around that whole issue around service literacy… how much effort… it's a bit like, you know, I couldn't read the manual of my car and change the carburettor. So why do we keep giving people these, you know, manuals that don't account for what it actually means to acquire reference points for services and supports that they've never seen, that have never existed before. You know, and I don't think we spend anywhere near enough time thinking about that. You know, there's no good just translating something from English and giving it to someone and they read it and they go, oh yeah but I still can't change the bloody carburettor. You know, I still couldn't do it. I can read it. Yeah, sure, I can read all the words. I can understand all the words but I still don't know what you're talking about. So I think those kinds of things, you know. And some of it's, you know, it's those change processes, that's the other thing. I think the primary prevention, health promotion activity doesn't move beyond the core, the Australian community. And so, you know, I think you would continue to see around some of the health stuff, you know, you just look at Diabetes Australia's website.

You know, so Eastern African background people have the highest rate of type two diabetes in Australia. But there's no Eastern African background languages let alone any specifically designed capability understanding pieces, targeting into those communities. So that kind of washes across the whole thing, you know.

Interviewer: In terms of other… in terms of focusing that on the financial, are there any specific challenges, cultural specific challenges of migrants?

Respondent: Yeah. There are.

Interviewer: Relating specifically to money?

Respondent: Yeah. And we ran a little project for a state government around women's understanding of money in particular and their access to finances. And I think… but it comes back to that sort of, communities that have, still have very strong patriarchal value sets. So you get women with really low levels of financial capability. So that's a gender problem. That's probably, it's pretty widespread across a lot of communities. It's not unknown in our own community. But it's a significant issue. And having financial competence, you know, is low. Financial competency around, in a whole bunch of domains is low. I think some of that stuff around, you know, the implications of different credit and different financial instruments and products is pretty low. And you could see some pretty predatory practices by some of the Telcos and stuff like that, which have been looked at a bit.

Interviewer: It used to be, there was many a case years ago, there was insurance issues. There was a whole big thing with insurance companies, yeah, which they had…

Respondent: Paying for insurance they're never going to use, insuring for stuff they didn't have. Yeah. I think that thing about, you know, never having to have… never really having had to budget. So if you want you could buy something. You know, buy it on hire purchase, buy it, rent to buy. You know, and then realising that the $600 appliances are going to cost you three and a half thousand dollars by the time you're finished paying for it. That stuff, which are unfamiliar constructs for people are problematic. Yeah.

Interviewer: How about other cultural things? For example, if there was issues of sending money home or dowries or there's… are those financial issues that are in the community?

Respondent: Yeah. I think dowries, I'm not… I don't have an intimate understanding of how… I know like within the South Sudanese community it remains a significant issue because the older, the elders in the community still have an expectation that dowries will be met. The dowries can be as much as a small mortgage. Like $150,000/$200,000. Really big. Well, they translate what… you

know, if you used to get cows and translate the value of the cow into dollar value and then apply it to a couple that are going to try and get married. Underage marriages are an issue still in some communities. Kids getting… you know, people getting… especially young girls getting married. I don't… certainly within I think the Afghan and some of those communities where there's a press to get people out of difficult situations and the key to that becomes marry this guy, marry your cousin, marry this bloke. And that's a pathway out. So forced marriage. I think you can… you know, you have to be able to disaggregate arranged marriages from forced marriage because of that, you know, they are different things.

Interviewer: One's illegal, one's not.

Respondent: One's illegal and one's not. You know, and we did a lot of work with a couple of the communities in (NAME OF LOCATION) around where they wanted help to try and do a, you know, community attitude shift around… the community leaders approached us and we did a project with them around trying to change attitudes and, you know, girls getting married at 15. But they're still quite despairing. They said, you know… so it used to be that the schools and other organisations could track when this was happening because the girl would move house, which was a really good indicator that this was going on. So what they did was they just started getting the boys to move. So we did all this work with the communities, it was over an 18 month… it was a reasonably long project, in collaboration with the leadership of the communities, which identified this as a problem. Shifting the attitudes still within the community is really hard. I mean, not, you know… it's hard to change people's minds. Or it can be very hard to change people's minds on stuff. And in a recent conversation was saying, yeah, but now they know that if the girl gets married and moves to the boy's house, everybody's going to know. But if the boy moves, no one's tracking that.

Interviewer: In terms of other financial… are there other support organisations for financial services that you work with or exist in the area?

Respondent: Yeah. There's a pretty broad network in this region of Melbourne around that crisis support, material aid, that's reasonably well integrated. In the City of Greater (NAME OF LOCATION) , there would be six, five or six providers including Salvation Army and that. So there's an integrated network that exists.

Interviewer: How does this broader the financial literacy rather than the immediate support?

Respondent: I don't think that there's a whole heap of work outside of the work we would do that's going on that I'm aware of. Yeah.

Interviewer: OK. Now we're going to move…

Respondent: Which again, would be good to see happen because we can't do it all.

Interviewer: No one organisation could do it all.

Respondent: No one organisation could do it all. And I do think it is an issue for people.

Interviewer: How about in terms of moving onto like legal challenges that the migrants face and I guess what kind of support do you give for them for like legal sorts of issues? I mean you mentioned a couple already, but…

Respondent: Yeah. And also we're collocated with (NAME OF LOCATION) Legal Service. So people come here and so they're… when you walked in, we sort of crossed the main of this building but they occupy the other side of the glass door. So we have a common intake in front of house. So people come here for that legal aid and legal services and we would refer.

Interviewer: So you just refer out to them?

Respondent: We are referring to them. So they run a drop-in service I think three or four nights a week in the afternoons from about four until seven. And we would interact with them a lot around a whole range of things. And where people have specific legal issues that we see through our assessment process we would…

Interviewer: So then you… so you don't have particular legal services other than you direct out?

Respondent: No. So we don't provide any… we don't provide any kind of legal counsel or legal programmes other than we have a thing, we run a programme for the Victorian legal aid called… that child support… so we are… that programme supports women to access and get child support where they've divorced or separated from their spouse. So often they'll be… and the men will just walk away and the woman's left with two or three dependent children. And so we negotiate the sort of reinstatement or access to the suite of supports that are supposed to be there, some of which is child support payments from the spouse.

Interviewer: And how about any issues, for example, because you mentioned earlier about like, you know, violence, family violence, or nothing about that either?

Respondent: We work in… we're in the preventative, in the prevention space. The response space, we're linked in with crisis support specialist providers in our region, yeah.

Interviewer: OK.

Respondent: Which is a problem. And they are… we did mount the argument for a recent tender, which we weren't successful for unfortunately. But there is a big… the complexity for these communities is that… and this is a good example of where this universal, you know, response is inappropriate. Where for people who are… if you're in a family violence situation and you want to access the crisis support services funded by state Commonwealth, you have to be prepared to leave. And the implications for women from these communities in leaving is hugely more significant than it is in other, you know, within the generalised population in Victoria. So people don't access the crisis support services unless… because you leave, you then you have a devastation of your social capital, you lose your bonding capital, and you don't have any bridging capital. So you die in social isolation. So they don't access, they don't access the crisis support unless the implications of not doing so are you're going to lose your children. So when you're in a family violence situation then the kids are exposed then mandated reporting kicks in. Then we'll respond. But it's only at… typically… so that whole crisis support around family violence is really disabled when it comes to women from, you know, culturally diverse communities. And that's quite a good example of why this is so problematic.

Interviewer: While you don't do the services, do you give them advice about where, so about who to talk to and that? So how familiar do you think they are with the potential services that are available?

Respondent: Again, it would be on a continuum. You know, some people are quite well orientated to them. Some without any idea at all. So it's a, you know, case by case. But yeah, we get quite high-density family violence as part of the presentation through the crisis support. So the people come, you know, a woman comes in and says, I don't have any money, but the underlying issue not I don't have any money, it's I'm in a family violence situation. So then we would have the whole case work around that, around the pathways, referral pathways, how they're facilitated and choices and actions and risk plans and safety planning. So we would do things… so while we wouldn't do, we wouldn't provide any therapeutic or counselling response and we don't have a crisis, like we don't have crisis accommodation, we would… the workers are skilled up to do, establish safety plans with the women. She would take that safety plan away, make sure that the, you know, that would be inclusive… and we have a fairly well designed, which we developed in partnership with EMPath and Our Watch around the service flow and what people have to do and mandate our people to do in response to family violence.

Interviewer: OK. So would people know… do you think they would come here to seek advice about legal services or they know you don't offer them so they would go…?

Respondent: Yeah, they would. So we would get lots of people that would come to the door with a presenting issue but seeking, looking for what should I do about this or this or this or this, and what do you think I should do about it. Yeah, that's… that would happen all the time.

Interviewer: And what are the key challenges to accessing legal services?

Respondent: The funding, you know, the resources, the available resources.

Interviewer: They're just not available as much as it should be?

Respondent: So demand outstrips supply significantly. So if you came in here at four o clock, I don't know today, but there will be 30 people there, it's a numbered system. They have a limited number of appointments. You don't make any appointment. You come, you get a number, and you wait. And when the time's up, then you come back next… the session's over and you come back again the next time.

Interviewer: So then they'll just go to the end, OK.

Respondent: Yeah. So you know, the… I think that and I think the other, and you know, it is a legal… but access to migration agents to support family reunion, you know, that's disastrous. Because really I think that's the other thing is family and family reunion are a key component, you know, are a really critical plank in good settlement and 15 years ago when the Commonwealth withdrew migration agent support for new arrivals from the Settlement Grants Programme, it created a huge hole. So people's ability to sponsor family is being, you know, shrunk, it's been impeded by a lack of support for that. So it's really… and there's a lot of crooks in that space too. So people get ripped off. Because you can't actually fill out the application. So even if we wanted to, it's illegal for non-registered migration agents to fill out a huge component of the applications themselves. So you have to have the resources to engage someone to do that with you. And one, it's expensive. And two, there's a lot of people that are real crooks. So it slows down and complicates, you know, the process of getting family reunification, which then impedes and slows down the integration and settlement process.

Interviewer: Of course. So the next questions relate to mobility of clients. What are the key reasons why your clients might move from one place to another, one suburb to another?

Respondent: Yeah. Mostly it will be cost. You know, cost of living, pressures will move, will create, will make people move. You know, so (NAME OF LOCATION) and (NAME OF LOCATION) have become a lot more expensive, private rental so people move out to the next suburbs, move away. They simply can't afford… you know, people just simple can't afford the cost of rent in these suburbs anymore. So that is a problem because then you get big anchors, communities established, but then

people can't stay within that, they can't stay close because it costs too much to… it's a bit like, you know, in Melbourne, the big public housing estates which were set up in Carlton, Fitzroy, Flemington, North Melbourne. So typically people would move into the public housing, establish themselves then move off into the local community. But, you know, I don't know if you're from Melbourne, there's no way you're going to move now from [indistinct 81.37] Gardens, the towers there, and rent a place in North Fitzroy or Fitzroy. You're buggered. And in Kensington, the same, Flemington, the same. So then people stay in problematic housing or move far away from what was their…

Interviewer: In Craigieburn or…

Respondent: Yeah. So you know, people would move off the Carlton estate but they'd move to Epping. You know, the outer outer north. And so you break all the social connections and all of the stuff, all the things you learnt about the community then become… you know, you have to break that.

Interviewer: OK. So in some ways you're suggesting they're not moving to communities, they're moving… they're not moving somewhere purposefully in the sense of, I want to be here. They're moving more away just because I can't afford to be where I am?

Respondent: That's right. Economic drivers, you know, would be the main… some people, you know, so things can go wrong and people will move away because they feel safer. It's, you know… so some people actually choose not to locate themselves within a large pocket of their own, you know, of the diaspora. They'll choose purposely to move and situate themselves outside of that, their cultural community, again, because it will force you to acquire and facilitate all those other things they know are important. I don't know if I would do that. You know, if I moved to, I don't know, if I moved to Venezuela I'd probably find out where the…

Interviewer: The ex-patriots lived.

Respondent: Ex-patriots were.

Interviewer: Do they move much for jobs or is there much movement for employment? I guess we're trying to figure what that anchor is.

Respondent: Yeah. I think the anchor's the cultural, you know, that sense of place and space and that's really attractive. And like I said, from (NAME OF LOCATION) to (NAME OF LOCATION) , an enormous amount of people will come into (NAME OF LOCATION) because it's culturally comfortable but they'll live in the neighbouring LGA. People will move for jobs. So there were some, there's been some… and there's been some reasonably successfully. But you have to move a lot of people, you can't just move one or two. So you know…

[CHAT]

Respondent: AIMS did that, you might have seen it, you know. They relocated over three years I think 200 Korean-Burmese families from Melbourne to Nhill to work in the duck meat processing company, which has been really successful. And then I think Deloitte did an economic impact analysis which said, you know, those 250 families created a net economic gain for that shire of $50,000,000 a year. So it's really big. So it can go really badly wrong, too. But, you know, it's moving a critical mass to an identified opportunity. I'm a bit curious whether that… because the reason why Nhill was dying was because, you know, the Australian young people had been leaving because there's no opportunity in Nhill. So you get a group of people that, the Korean-Burmese, and the adult population will go and meet in a duck meat processing plant but they're highly aspirational for their kids.

Interviewer: Yeah, so…

Respondent: So what do you do? So the second, you know… and they will work, you know, killing ducks and raising ducks and processing ducks but they don't… there's no way they're going to want their kids to do that. So their kids are going to… they're going to get as many of those kids as they can, they're going to get through primary and secondary and tertiary studies and those kids are going to leave to pursue opportunities not available in Nhill. So whether you're going to have to just keep doing that, I don't know. Or… but it can't stop there because it will only hold for a generation and then you run out of rope.

Interviewer: Are you seeing any trends in mobility in the early years of migration? I mean are any… you said they're all moving to the low cost areas or the communities. Are you seeing any changing trends?

Respondent: I think, you know, the trends are… I think the trends that you see are policy driven in the broader migration programme, which is becoming, you know in the last 10 years, has become increasingly targeted to bringing people that will meet specific perceived skill gaps or transitioning international students onto permanent residency visas. And that's pretty targeted. So I think if you compare that economic mobility of the broader migration to 15 or 20 years ago when it wasn't as targeted and a larger component was family reunion, now I think it's 75% is independent skill and 25% of the migration is targeting family reunion. So it's been rejigged over that time. So I think the policy, because of who they're picking and who's getting permanent residency through the migration programme is changing, has changed the demographic more than anything else. So you just pick people that are highly skilled, speak good English, have, you know, have a professional skill fill the perceived gap or opportunity in the labour market. So that will have facilitated that change. The refugee and humanitarian programmes, that sort of 75%, you know, unemployed

four years post arrival has not shift much over the same period of time. It's stayed pretty, you know, it's stayed pretty constant.

Interviewer: Alright. The next questions are about access, migrant access to education and literacy programmes. So do you have any services here that provide education and literacy programmes for new migrants?

Respondent: Yes. So this provides a drop, a five day a week drop-in service in the afternoon that supports young new arrivals. Because we're collocated with the biggest language school in the region. So we provide a drop-in service where young people come in for assistance and we run a homework programme, homework support programme for those cohorts. And it's a broad base, social support, that's what the drop-in does. Young people drop in to use the computers, to get help putting a CV together, they've got a problem at home. We've got 10 THM properties we manage, they become homeless, there's family breakdowns. So they come through for all that stuff. And we're contracted to run a number of programmes that look at chronic school absenteeism and support for families to address that issue. We don't provide vocational training or education programmes ourselves, per se.

Interviewer: Adult literacy programmes?

Respondent: No.

Interviewer: No, OK. OK. OK.

Interviewer: And what do you think are the key issues or barriers for children of your clients to accessing school or university education?

Respondent: Cost.

Interviewer: Cost?

Respondent: And education background. You know, the education backgrounds of their parents and how well equipped their parents are to be able to support those pathways. You know, so a little bit frustrating. You still see a lot, a lot of initiatives focusing on kids when the enabler is the parent. So when there's something going terribly wrong in the education process it always seems to be a bit inane to be focusing on children, you know, because the kids don't get themselves to school. Kids don't do those things. You know.

Interviewer: Do you think the parents not valuing education? I mean what do you mean by, you said the…

Respondent: I don't think that. I think that education is really highly valued by all new arrivals. I think that people understand that it's a, you know, going to be the key determinant of success for their kids. But I think there is probably, in some communities it will be a gendered problem

around perception of how far do you need to educate your girls. That will be an issue. That will be…

Interviewer: It's not just new migrants?

Respondent: What's that?

Interviewer: It's not just new migrants?

Respondent: That's right. So there's still…

Interviewer: There are communities that…

Respondent: Yeah. That's right. It's not at all… yeah, that's right. So there is still a gendered problem there. And then that's probably the most significant one. And I think the other things that impede kid's access to education relate to the broader issues that would be occurring and impacting on all families, you know, regardless…

Interviewer: Of whether they're new migrants or not?

Respondent: Of whether they're new migrants or not.

Interviewer: And…

Respondent: And, you know, and the cost of tertiary education. You know, can you afford, you know, can you afford to pay? You know, you could afford the HECS, the burden of debt that you accrue to do an undergraduate degree but the postgrad, hardly any postgraduate studies are HECS anymore, which is a disgrace, you know, really. I can't understand how we allowed that to happen.

Interviewer: Nor can you get Austudy for many of them as well. So you have to be working.

Respondent: And you can't get Austudy. So there's not a lot of… you know, this isn't just going to apply to new migrants.

Interviewer: There are loans in Australia?

Interviewer: Yeah, for postgraduate study.

Interviewer: There you go. And so can you tell us about…

[CHAT]

Interviewer: And can you tell us about any special packages or subsidies provided to support education opportunities? So I know there's the 510 hours, there's the 12 months of…

Respondent: Language school.

Interviewer: Language school. Any other things?

Respondent: And the rest is pretty much what's available to everybody. And in Victoria there was a really good, they called it the Education Maintenance Allowance, which was a reasonable… it wasn't great. But there was a bucket of money, some of it went to the school and some went to the family. And the government decided that they would take the component that went to the family away leaving it with the school and that's very problematic. You know, because also then people also have to humiliate themselves. They have to go gap in hand to the welfare coordinator or something at some school to access that stuff, which is appalling. If they were going to take a piece away, take the piece the school got away. If you had to do something…

Interviewer: I think… because I was on the school council and I think they did it because they felt that it wasn't always being allocated in the…

Respondent: In the right (?) direct space.

Interviewer: Yeah.

Interviewer: [indistinct 92.20] children, I have the other side and I had to go to welfare people at school when I was in primary school. So the opposite experience, which was, which for my whole, for my brothers and my sisters and well, not a very good experience, nor for my mother.

Respondent: No, yeah. No, that's right. So I mean I would have privileged the piece that went to parents and tried to work out ways to get it, appropriately… I mean, it would be still… and I get, it's pretty horrible because the range of things even within the state funded education system, the number of activities in secondary and primary schools now that are…

Interviewer: Voluntary fees.

Respondent: Yeah. But you can't, you're not allowed to go if your parents can't afford it. You know, so swimming lessons and special art stuff and all that stuff. So… and if you don't get to do that stuff, it impacts on the whole, you know, the whole of the process. Not just… because that notion of being excluded is pretty strong. So I find that really quite depressing. But it's not just about this cohort, it's just that these cohorts have a higher vulnerability, you know, to living in poverty.

Interviewer: And are there any, can you outline any kind of employment opportunities that you're aware of that support migrant children when they finish school? Anything specifically supporting migrant children or is it just…

Respondent: Well, it's just the same stuff that's there for everyone, you know. And occasionally someone does put little boutique programmes together but I think that they're… you want to make the stuff that you can scale up, work properly for everybody rather than try and take… because again, I think there's always the temptation to try and use culture as the determinant in your design and thinking process, which is bad, you know, of what you're going to do. It's fine for how but what has to respond to the, you know, the circumstance and they're not culturally determined. So that's the challenge. But people always want to go for that, oh well we'll have the specialised approach for Somalis or the specialised approach for the Iranians (?) or… which is dumb.

Interviewer: And so in terms of employment opportunities for migrants more generally, are there any special provisions that you're aware of to help with that?

Respondent: Yeah, I mean AIMS has always run a reasonably well credentialed support for professional migrants. You know, they have a pretty… I think they have to pay, I think there's a fee associated with it. But they have specialised support but it's for professional migrants. And then the rest is the traineeship programmes, the, you know, job actives.

Interviewer: Yeah, so that's still just from government services?

Respondent: Yeah, government services. The employment…

Interviewer: It's not targeting migrants per se?

Respondent: No.

Interviewer: OK.

Interviewer: And so overall, you mentioned quite a number already, but overall what do you think the key challenges migrants that you work with here face while adjusting to Australian culture and life in Australia?

Respondent: I think getting that… I think the key challenges are quickly getting a diversified social capital base because that's the enabler… I don't know if you've ever looked at, and I'm quite fond of it, the [indistinct 95.54] integration framework. They were the UK researchers. I think that's brilliant. It's a bit of Maslow's put into it. I use that, I think it's perfect. So I think it's… and that, the enabler is that social capital line. I think that's critical. So how you, you know, better accelerate the acquisition of that social capital, which then goes onto enable all those other things, education, employment, you know. It's like they said, 75% of jobs are never going to be in the newspaper. So if you don't anybody, if everybody you know is, you know, Indian and doesn't have a job, well, you know… and your only access to the employment support is through a government funded employment programme then

you're never going to access 75% of opportunity. That's a big problem when you're also already coming off of, you know, a complex base for a whole bunch of other reasons.

Interviewer: Yeah, for sure. And so finally, the final question, what would you like to see as possible solutions to helping or supporting migrants to adjust well to life in Australia?

Respondent: I'd like to see better integration between that secondary and universal, better interface if not integration. And integration is a bit tricky because you wouldn't want to lose the specialist, the specialist component piece. You wouldn't want to see it subsumed into the universal space. But a much better interface and a much stronger, you know… some better research on, and understanding of, service literacy acquisition processes amongst new arrivals would be, you know, would be really helpful. And that they would then become invaluable tools around some of the other broader health promotion and advocacy to force and push the hot parts of sectors on that base to, you know, to improving their interactions and positioning to people that they're not… I mean you know, the biggest, I had a meeting with the biggest family support, the lead in the child first, the biggest family support agency in this whole region, you know, and they were saying… I was commenting about, I was doing a tender, we had to calculate what our interpreter uses would be for a year, which was hard. And they said, you know, we get a credit loan from Department of Health and Human Services but we never use it. It's like… I feel like saying, how could that be? How could you be here providing that service to this community and never use your interpreting, never use any of it, you know. That's the biggest…

Interviewer: Do they have people working from the bilingual…

Respondent: No.

Interviewer: Yeah, right.

Respondent: They said they were quite culturally diverse and that was represented by the general manager who was born in Sweden, came to Australia as a five-year-old. Which didn't really seem to represent my understanding of cultural diversity. But anyway. So that's some of the problem. You know, that's what I mean by adapt… you know, these big services and service systems have to change to meet the entitlement of the group of people that are different than they've traditionally developed their processes around.

Interviewer: Yeah, definitely.

Respondent: So those would be the things. I'd like to see some big pedagogical shifts in some of the critical ways that some of the on arrival educational programmes, the AMEP in particular, are offered. Because

I think that they're just terrible problems that have been there perhaps forever, for 50 years. Part of the problem is, you know, that's also unit cost. So you get big providers like TAFEs have got about and, you know, you get paid $8 an hour for a bum on a seat in an English class. Well, you can't organise… so then, you know, and you need 18 of those bums on a chair to…

Interviewer: To pay a person.

Respondent: To pay the teacher and the overheads and all the rest of the costs associated with delivering it. Well, you can't. So you're inevitably going to have the five professional Hong Kong migrants sitting next to the three women who came from rural Afghanistan sitting next to delivering the bloody English programme. So you know, the 80% are going to do well and the 20% are going to do really badly. But the 20% are going to be 100% of a cohort. And that's the problem. So you know, there was some great work done 10/12 years ago saying the whole thing needed to be reconfigured really around what were people's aspirations and what was the utility of the language for them, what was it going to mean.

And you know, even here when writing this codesign with these women, economic participation group, the women were all saying, we need English classes. And the facilitator is saying, well you just got stuck. I can't facilitate them through this. I said, well there is. I said, ask them if you could wake up tomorrow and speak perfect English what would you do with it? And I said, you know, get them to tell you that. Because they're always going to say, oh we can't speak English and that's why everything's so hard. So yeah, but if you could speak English, if you could, if we could wave the magic wand and you woke up tomorrow with English spoken as well as I speak it, what would you do without it? And if people can describe to you what they would do with it you could probably start to think about then where's the best place to locate the acquisition of the language from that isn't, you know, a room with a whiteboard and a chalk and talk teacher, which sits with the professional migrants from Hong Kong very well. But whilst that continues to break down, if people can't get enough language in the funded period of time the rest of the process will elongate and be problematic, highly problematic.

Interviewer: Alright. Excellent. Well, that's the end.

Interviewer: Is there anything we didn't ask you?

Respondent: No, I don't think so. It was pretty comprehensive.

Interviewer: Yeah. Definitely a lot in that. So thanks again for your participation.

Respondent: My pleasure.

Interviewer: And we really appreciate…

Interviewer: Your assistance.

Interviewer: Yeah, and expertise in the area. So interview ends at 11:57.

[CHAT]
