# Supplementary material for: The importance of information acquisition to settlement services literacy for humanitarian migrants in Australia
Source: PLoS One. 2023 Jan 6;18(1):e0280041. doi: 10.1371/journal.pone.0280041 (PMC9821785; doi:10.1371/journal.pone.0280041)
Supplement: S1 Data — (ZIP) [file pone.0280041.s003.zip › SP_03_NSW.pdf]

Interviewer: (SERVICE NAME) and I'm interviewing the Acting C.E.O.

Before we begin, references to migrants will be inclusive of migrants and refugees and applies to the first five years of settlement in Australia.

The first set of questions, they're about the services your organisation provides. So, can you tell us about the services being provided by your organisation that assist newly arrived migrants?

Respondent: Sure. The core program we have is the S.E.T.S. program. Previously it was the S.S.P., now Settlement, Engagement and Transition Support Program, which is the government funded, main program to support people here, up to five years, both for migrants and refugees. We primarily do case work in that space. We both do low-level casework. People might come in as a one-off, might need help with filling out a form or contacting their energy retailer, something like that, to more medium, complex casework in that space that is ongoing. That's available for migrants, as soon as they've exited the H.S.P., (indistinct – 1:35) Support Program with S.S.I., up to five years in Australia and they can come and go as they like, completely voluntary in that space.

Also under the funding, we do community engagement programs as well, so currently we have a women's group called Strive. It's been ongoing for a couple of years now. It has a wellbeing focus, so it's really what women like, but things like we did a swimming activity session once. We had a family counsellor come in and do stuff on relationships, it really varies in that space. We also have a parenting program at the moment that's been really popular and we've had a childcare worker come where you can bring your kids which is really hard to find and we've had citizenship form assistance sessions, men's groups in the past, but I don't think they're currently running now, but the idea of our program was in response to the communities needs as they go through… oh, the other one was safe driving.

It supports under the W.D.O., the Working Development Order as well, but focuses on migrants and refugees and there's a lot of issues regarding people getting fines, not understanding the fines, not understanding insurance requirements, how to engage with police if they pull you over, so we've been running these sessions for a little over a year. We have different people come at different stages, so we've had the police come in and explain that the random breath testing really is random, it's not necessarily targeting communities and how to present yourself there. We've had Legal Aid come in and do sessions there too, so that's a really…

Interviewer: Oh great.

Respondent: Yeah, basically how to understand street signs. People come in with parking fines they don't understand with limits and things like that and we do outreach programs as well, so we might go… we recently did a candle making workshop, so that's under the S.E.T.S. clients services program and we also have a Community Capacity Building Program which is really looking at engaging community leaders and representation to develop programs they would like to do.

The big driver at this time is in the digital space, so we've seen it's an area where people have requested support in, so linking them with websites and things like that, but again, that changes over time, so in the past we've supported African people in that space and help them form organisation in how to hold meetings, how to take minutes and how to follow up in those kinds of areas as well.

Our core funding in the migrant refugee space, but all our other programs we have with funding are as focused as migrants and refugees, so we've got funding for playgroups and again, we focus on… it's open to anybody, but our clients are those who are migrants and refugees, particularly asylum seekers who aren't eligible for a lot of other programs and then we have aged care, Commonwealth Home Support Program, we have Parents Next and N.D.I.S. and we have a youth based program under S.E.T.S. funding and we do youth casework, ongoing groups as well, so we have Drop Zone, a social group that meets every week and they do physical activity as well, like information on wellbeing and we do school holiday programs in that space too. A lot of ongoing change, but every other program, we work as the (SERVICE NAME)., focus on migrants and refugees, so anyone could access them, like it's open to everybody, but knowing what our speciality is and I suppose the need from the community is there as well.

We used to be the Complex Case Support Program provider which is really complex casework which no longer exists, got defunded, but we were the biggest provider in New South Wales.

Interviewer: Who was funding that?

Respondent: It was through D.S.S., and under the new H.S.P. program, it was combined into that, so Special Intensive Support Services, which is now in a tier of H.S.P., but funding wise, it's significantly less funding, so therefore the actual work you can do with clients is significantly less. We are a sub-contractor of that, but we've reduced the amount of clients we're seeing in that space. We just weren't able to get the outcomes we were hoping in other spaces to deliver there.

Interviewer: You’ve answered quite a few of these already, so can you tell us who you collaborate with to do this work?

Respondent: Definitely. We’re part of the New South Wales (indistinct – 7:07) partnership, which is, so 22 organisations across New South Wales have come together under the S.E.T.S. funding to be a partnership and we are the lead organisation with that, so that’s now in the second round of funding there, so it’s all the former M.R.C.’s. Everyone’s changed their name in that space and that’s 11 community-based organisations, so we are a very strong partnership in that focus for our settlement work. We have more formal partnerships. We’re partnered with (NAME OF LOCATION) Multicultural services and (NAME OF LOCATION) Services and our refugee mentoring program which is, we have people who are mentors, mentees, linked together. That’s partnered with a service in Wagga Wagga and then with core communities, so we have quite a few partnerships that are funded, but I think all of our programs requires partners.

With our playgroups, we are partners with local schools, (NAME OF LOCATION) Council, P.C.Y.C. for our youth programs, outreach services from here, so we partner with Anglicare, Salvation Army, so we provide support for clients in the hub we are here and partner with (NAME OF SERVICE) for our disability support programs. When I look at the annual report, there’s always a whole list of people we’ve worked with in different capacities over the year. It’s growing and growing, resources are scarce and working together becomes more manageable, more feasible so that we’re not repeating things, but also that we get to the communities we need to.

Interviewer: Are there any organisations with whom you choose not to work with and why?

Respondent: It hasn’t come up, but there could be. It may just be that it doesn’t relate to our core beliefs, so in our job space, we have Parents Next, but in a compliance framework, it’s less so than a lot of the other programs, but we’ve chosen to not go down some of those paths as it doesn’t link in with our core beliefs.

I don’t think we’ve been approached by anyone so far that we’ve been against, but we’d be very vocal and clear we wouldn’t work with people we didn’t feel comfortable, but it hasn’t come up to date.

Interviewer: Are you aware of any services that are needed, but not available?

Respondent: Yes, so a huge issue for both migrants and refugees is transitioning to school, so we have playgroups, but there just isn’t enough supported

playgroups in this area and then a lot of young people are going to school not having any previous out of home experience.

Interviewer: Is that particularly across the community or for migrants?

Respondent: Migrants and refugees. There's lots of reasons, but a lot of it is financial with day-care being expensive, lower employment for mothers in the migrant refugee community and expectation of what motherhood looks like can be very different. We have support networks to leave your child with somebody else, so our playgroups are now focused on transition to school, prioritising those people, young children in that stage so they're prepared for school and have partnered a lot when kids do go to school and the challenges with not having that experience. There's a really big gap in this area for migrants and refuges and we're constantly looking for more support. I think a lot of the issues come from… to get support as a family, you need under that quite high level support and the reforms are looking at changing and we'll see what happens when that comes through, but I know from my casework days when managing caseworkers, there's that constant challenge. Parents need that lower level support. It really isn't available and that's why we started Parenting Place. It has a diverse mix of migrants and refugees and because of funding requirements, this is a bit broader in this space and the lack of confidence and knowledge in parenting has been really apparent and it's been a six week program and we're six months in now and again, we're trying to engage clients, interpretation, communities being able to access when things are difficult. For me, that's a big gap and with the younger people, it's primary school aged children, so youth programs for 14, 15 year old's which is great and if we can get traction in that space with high schoolers, we've got the I.E.C.'s, but those primary aged children…

Interviewer: There's a big gap between the service delivery between zero and eight. It used to be with F.A.C.S., but I think it's five now. Youth services were from 12, but there's that gap in between.

Respondent: Other programs are all cost related, so it can be quite prohibitive for people who haven't got the income to access them. We're continuously looking to develop in that area and find ways where we can work both with the parents and the children in that space.

Employment's always a big one. It's just so challenging. There's been so many attempts in supporting migrants and refugees into employment, but how that actually plays out is very different. It's a constant want and desire of our community to be able to work, but even matching them in to work is just really challenging. We did previously do the R.E.S.P. program, Refugee Employment Support Program, but we were really struggling, under the framework that they use, to get meaningful

employment for people, because it just needs a lot more intensive support, so we have a lot of volunteers involved in the M.R.C. and a lot of that is to support us, but also for people to have something on their resume with local experience.

N.D.I.S. is a whole other thing in itself, but getting migrants and refugees who are eligible, on to N.D.I.S. a humungous challenge and it's something I've recently put an application in for funding. It's such a process to understand it and get through it, that a lot of our clients just don't bother, because it's just too hard and there's been a lot of grants put forward to support in that space, but under the Complex Case Support Program, we used to do a lot of support there, but you'd have to do a lot of hand-holding through the whole process, because it is so different from where people have come from as well.

Interviewer: People providing these services find it challenging.

Respondent: I've been touching on it since its fruition, but still I struggle to understand all the loopholes and everything else. I think getting people on to N.D.I.S. is a space that needs support and migrants and refugees with a disability that may not yet have a diagnosis or their expectation of what they can do are being challenged, because it's very different in this environment, so it can be very confronting and space that there's scope for more support in.

Interviewer: That's really good. Are you aware of any services that are overutilizsd, in terms of long waiting times, difficult to get employment?

Respondent: In the local area?

Interviewer: Yes.

Respondent: One of the big places people come to for support is through E.P.A. vouchers, electricity and gas vouchers and I know wherever you go… although we have a lot of organisations that provide that support in (NAME OF LOCATION), there's still always challenging getting appointments, getting linked in, so I think emergency relief and financial support is always really hard to get. Also psychologists and psychiatrists are much harder. I just returned from maternity leave a few months ago, so things have changed so much in this environment, but I know from my client facing days, I think there were two or three Arabic speaking psychiatrists in the area and most were in Bankstown and had long waiting lists and high prices and had lots of issues. Getting language specific support is really difficult in the mental health space and there's places that provide trauma in regards to refugee trauma, but provide other specific mental health and there's some gaps in that space for language specifics.

Interviewer: Do you find Trans-Culture Mental Health helpful?

Respondent: I think they've changed how you can refer, so I think now you have to be referred from a medical practitioner. You can't be referred from casework space. Red tape. We trialled having counselling Arabic male and female services here, but didn't get the uptake we were expecting, so it's a tricky one. It was great in group spaces, but the one-on-one didn't.

Interviewer: More acceptable for them.

Respondent: Yeah, but definitely psychiatrists, there were challenges there in that space and I feel like I should mention housing being an issue. Wait lists, support for housing is just ridiculous. We're not seeing it as much, but a couple of years ago there were a lot of single women on refugee visas and older single women, so shared accommodation was not what they were looking for. One bedrooms are hard to come by and really expensive, so it was really challenging to find housing support in that space. We're seeing more larger families now, so it's a bit of different space there, but still, 15 year waiting lists there. It's ridiculous and with a build up of the new high-rises which are lovely, but they're now out of a lot of people's affordability.

Interviewer: So when you say waiting lists, is this for Community Housing?

Respondent: Yeah. It comes from a few years ago in my experience, but any type of housing support is just hard. We had Wesley Mission doing outreach for a while here which is really great, but if someone can't afford rental properties, there's just nothing else available in that sense, unless they've been through domestic violence, so it's really challenging there.

Interviewer: Are you aware of any services that are underutilised? So, not a good pick-up.

Respondent: I think there has in the past. I'm just thinking, when there was the initial increase in an Iraqi intake, a lot of people got additional funding, people that came forward for support and there was 12 months funding, but it just didn't happen. Another society had another program, but didn't have the numbers they were expecting.

Interviewer: And do you think that's because it's new and the community don't know about it?

Respondent: Completely. There are so many different supports and people don't know who… there's just too much going on and people focus a lot on the initial, that first year or two and if people don't uptake, there's too much going on in their lives, trying to do the basics, but sometimes not ready for other

engagements and there's no traction that people support. I think there used to be more things available, but now not as much. I touch base with caseworkers and if there's anything better, to let me know.

Interviewer: And we've got all these other questions around different education. It might trigger something. That's great thank you. Can you tell us about the methods that you use to measure the effectiveness of services you provide, so the methods that you measure?

Respondent: I think one thing we're constantly looking at is making sure we provide good outcomes, frameworks, measure the work that we're doing. To start with, when we look at regular consultation, that's more formal consultations, but we also do a lot of ad-hoc with our group, so with our youth groups to see what would they like next and if what they've been having has been what they're looking for.

We have a one-page evaluation form that's in Arabic and English and is very simple. There's questions, ratings, a smiley and sad face, people with different literacy can respond to it as well, so for our all groups, we're doing that quite regularly and we're now trying to capture the pre-level of understanding as well, so a specific like with the Parenting Place, a focus of the group, what the initial level is. We do find a lot of our clients just say everything is great, so getting really valuable, proper feedback is challenging and I do find that particularly in the refugee community, they say "it's great, we love it", but they won't come back. Obviously something wasn't quite there.

In our casework services, under the government funding, we have a score outcome, which is our online rating system. I personally don't think it gives a very accurate, in-depth understanding of the work you're doing, but we now have an online feedback form and have started implementing that. Depending what program you're with, at least once a fortnight, asking your clients to complete the feedback to get a much more detailed feedback from people. We know if you ask every single person you see, it doesn't happen, so asking our aged care workers to take it out once a month and actually ask the people they're engaging with to respond on iPads. We're hoping to get more detailed information through that.

It's interesting in the Parents Next space where we have a lot more non-migrants or might have been here a lot longer time, that they're much more open to providing feedback, so it's interesting with the two different programs there.

Interviewer: Oh good. Can you tell us about any other issues regarding access to settlement services that migrants and refugees might be facing?

Respondent: A big problem is what Visa you're on. If you're a refugee great, you have the open services and if you're a permanent migrant, it's reasonable great too, but if you're on a temporary Visa, asylum seeker or even on a part temporary Visa, you just can't access anything. Most of our services aren't open too, so it's really challenging in that space. We're not funded to do anything in that space, but we try with our F.A.C.S. funding, but it's a complex issue with domestic violence or relationship issues, there's just nothing and you get shut doors wherever you go, because no-one's funding in that space and people obviously refer to us because we are an (SERVICE NAME) and there government's really cracking down on who we can see in that space as well and the flipside of that is people over five years. The focus is on the beginning five years, but your settlement doesn't end at five years with challenges you face. I know it's a big part of the review of settlement programs and one of the big finds is that everyone was saying the same thing, but the government decided not to change in that space. We find with our older clients that they may not have accessed us for ten years, but when they need to access for aged care in this crazy system, they need support there and as I said with our older, single ladies coming through, they don't have anywhere else to go with help to read their letters and those bits and pieces, so often the most vulnerable fall through the gaps there and puts a lot of pressure on service providers who are still seeing them without funding. That's a really big issue at the moment.

Interviewer: OK, that was the first set of questions. The next set of questions are around how migrants adjust to the Australian culture and society and the kinds of issues and challenges they may be facing, so can you tell us about your understanding how migrants you work with, understand Australian culture and society?

Respondent: Quite recently we've been doing a lot of work with the Aboriginal elders in our area and it's something we haven't had much interaction previously and our clients don't understand that part of Australian culture and were really interested in it as well. (NAME OF LOCATION) is very Iraqi and people have that comfort zone here, so a lot of the time, I think some of our older clients, I don't think they engage that much with the Australian culture, because they don't have to. They're able to get away or learning how to speak English as well, but I think across other parts of our community, there's different levels of engagement and understanding as well. Australian culture is so diverse and in the past, taking clients to Bondi, this is Australian culture and getting people out of the (NAME OF LOCATION) bubble or Western Sydney bubble which has been great.

I started here as a caseworker five or six years ago and I was the only non-Arabic speaking caseworker and initially it was "oh?" They actually liked seeing someone they could practice English with as well, so I think having

increased opportunities to interact with people from other backgrounds is really important. There's that fear of doing it, but when they start and realise people aren't going to tell them off for not saying things exactly correct, it builds confidence in that area of understanding culture.

Interviewer: Great. What are some of the opportunities provided to migrants to practice their own cultural values and practices?

Respondent: I think the community are quite established, so some Syrian communities that are coming through and new, aren't established at the moment so for example, (indistinct – 31:50) one of our largest communities in the area and they have a lot of associations and cultural heritage and do their own events and things like that, so I think there's quite a strong community in pockets of the community and so on our community fund that we have, we have a lot of energy and effort go into there and that's supporting communities be able to practice and celebrate their own community and culture and as I mentioned earlier, we've been supporting the African culture since before I came here, so a long time. There's a space and venue to celebrate the culture there as well.

Interviewer: What are your impressions on how the cultural values and practices of migrants are being recognised and respected by the community?

Respondent: I think in (NAME OF LOCATION) it's quite well respected. I think it's known to be a very diverse area and we don't see… it's not something people report much, but I think at school level… I think adults need to come and discuss issues in that space, but maybe in the schools there's less recognition of culture between peers and challenges in that space and a year ago we encouraged young people to understand people's cultures and had an event for young people to then be able to express their culture, but I think we're seeing more tensions.

Interviewer: The next questions follow on from that, around a migrant's sense of belonging and inclusion in Australian society, so can you tell us about the programs or support available that help to create and enhance migrants sense of belonging?

Respondent: Within us here?

Interviewer: Yes.

Respondent: I think a lot of our community engagement activities are in that space, so I think having that connection to Australia and to their own community as well, because we are focused in those areas, have the space to come and meet. There's a group of women that have been coming here for 30 years, just to play bingo in our community space, where they can feel it's theirs

to be in. Seems less important for men, because in (NAME OF LOCATION), there's all those cafes where a lot of men hang out in, they play chess there, but for women, there's a different space, a space where they can be together. We found that to be very important and we've found we don't need to facilitate there ourselves. Those outreach programs have gone to different parts of Sydney so they feel much more connected to being Australian, rather than just being kept to where they are now, which has been really positive and they haven't had the confidence of knowledge in how to go to other parts before.

Interviewer: Great. What are your observations of how your clients meet and interact with people from their own communities, to maintain a sense of belonging and cultural connections?

Respondent: We have quite established communities here, it's pretty great. You may bump into an old client in the street and people who are their Aunties or cousins, you don't know how many generations have been here before, so there's a strong connection. Every now and again, something's happened and they feel ostracised in their community and that's really challenging and they might not necessarily let us know, but they might go somewhere else, because if they're physically in this area, it's challenging or they might not want to see someone from their same background, but I think in general it seems pretty strong. Most clients we engage with have a religious area they go to, so it seems pretty good.

Interviewer: That sort of answers part of the next question which is who are the key community people that your clients contact for social or emotional support?

Respondent: Definitely a level of community leaders within there as well, but I think anywhere, people have their friends and family and when asked "who can support them with this?" I say "who would you go to?" You wouldn't necessarily go to a service for this, you'd go to someone… as I say, a lot of our community do have that support and I think it's good to draw on it.

Interviewer: The next set of questions are around programs that are responsive to social support and improving health of migrants, so can you tell us… you've probably mentioned some of these before. If so, we can just skip over them if there's nothing else to add. The types of programs that are providing social support?

Respondent: Internally?

Interviewer: Yes, I think you've already covered that off.

Respondent: Yeah.

Interviewer: What about health and wellbeing?

Respondent: We've previously had some programs that have particularly focused on health and developing partnerships with local G.P.'s but again, that's a short-term funded program. Sydney N.D.I.S. is health focused and aged care programs. As things flow depending again on funding, but also the community needs, so we've had Healthy Heart Program for women that was physical activity and things like that there. We've had some youth programs we really focused on with wellbeing and photography, trying to target those 18 to 22 age brackets harder to engage. Unfortunately a lot of things are short lived, because of the funding cycles there.

We did a creative journey with wellbeing, photography with families, reflecting on their journey, but it was really focused on forward thinking as well in that space and we've had a men's poetry thing on wellbeing a few years ago.

Interviewer: Thank you. The next set of questions are around programs around to enhance financial literacy and income generation. Can you tell us about any of the programs available for financial literacy?

Respondent: We have a relief program which is separately funded, but we do budgeting with clients, so for us to gauge and understand where their needs are and how to support them is a process we go through with them and we have Anglicare who do financials and (SERVICE NAME) do counselling from our office, to help people develop literacy in those area.

Interviewer: Do they have called workers? How do they cater for your clients?

Respondent: For the (SERVICE NAME) we do, we have an Arabic speaking lady and it's interesting, she has clients come back to her. Anglicare don't have interpreters, but we've also been doing group sessions as well that came from the client's requests to do group sessions regarding financial literacy as well. Sometimes for some of our females that come through, it's the first time they've actually had to manage the family budget, so definitely a space we've been working on as well.

Interviewer: What about around income generation?

Respondent: We have quite a few different employment supports and had not great long traction in the long term. I think a lot of the time… say we've had a six week program, which was the aim to be able to develop plans for employment and lots of people came to the program actually helping to develop their English or realising they need to study more. I think maybe two people got employment from going through. We just didn't get out of

it as much as we'd hoped to. We're limited currently in that space. If people are coming for employment assistance, we refer them to organisations that can provide more support.

Interviewer: Great. What kinds of financial challenges do your clients face while adjusting to life in Australia?

Respondent: Lots. A big one which I never really appreciated till I was on the front line with this work is that clients have come from wealthy backgrounds. Not a lot, but a portion, high level jobs, to then come as a well-established adult with a family to then relying on Centrelink income is really challenging and during budgeting, what clients are spending on their children and they say "our kids expect it. That's what they got back home". It's really hard for them to not provide it. People want to go to private schools, religious schools, that have fees attached to them. Those adjusting to having a different level of income is really challenging and having to ask people for money is definitely, really challenging. I think the other part of that is the unexpected costs. So often we get requests for energy bills, because people aren't budgeting for their first energy bill. They have no idea it was going to cost that. I think for people that have come from a different background, maybe haven't engaged in services before; paying your rent on time, how important those bits and pieces or they'll pay whatever they have, maybe a hundred dollars one fortnight and two hundred dollars, but it's just not sustainable, so that education in that area and understanding money full stop. A huge spectrum in that space, but definitely challenges.

Interviewer: What are some of the cultural dynamics, specific dynamics that impact and challenge the management of financial demands?

Respondent: I think one would be who manages the money and particularly if that changes over time can be really difficult. For example, someone starts getting a youth allowance and the young person gets money directly into their account, but then it's expected they pay to someone managing that, I think that can be a difficult one for sure and it can really play with the expected power dynamics in a family. You mentioned earlier about spending, so spending certain amounts on things and then not being able to is difficult to. We do get some financial support, we provide Woolworths cards because that's the easiest way to provide financial support, but then that doesn't let you go to your local green grocer or butcher to get the things you need, so being able to access that and health costs as well, I think that's a challenge. I know a lot of our clients have health issues because of ongoing trauma and having to spend money in places not bulk billed is a big one.

Interviewer: How do they overcome these challenges?

Respondent: I think partly time, learning how the system goes and I think it gets better over time and accessing supports. Where it is quite challenging, I'd say that's counselling capability. The other part is people do get dependant, so us as an organisation, letting them know it's a one-off, so people don't become dependent in that area.

Interviewer: Now the next lot of questions are around the support programs available to support your clients when they face legal challenges. Can you tell us about some of the programs or supports for legal issues around identity, Visas and inviting family members to Australia?

Respondent: Legal Aid provides outreach here and we have an immigration lawyer for that and we also have well, where it's starting, our migration agent service here at the (SERVICE NAME), but it's a paid-for service, but we help people fill out forms as well, so there's a high demand for both of those here.

Interviewer: And physical violence and other forms of violence or discrimination?

Respondent: We have a family law clinic. They're civil law and immigration and they have a special refugee law area which I think covers other bits and pieces. Say if someone needs legal support, we can go through there, but also our caseworkers can help them go through that process which again can be very challenging in that space. Previously we did court support.

Interviewer: What do you think some of the key laws and provisions are that migrants need to learn in their first year of arrival?

Respondent: Driving and insurance. I think the most issues we see, because people just don't understand their requirements of driving and if people have an accident and don't have insurance, they just don't understand they had to have it, so that definitely is a challenge. I feel like stuff like family and violence, people actually know as it's drilled in so much at settlement period, from what caseworkers have told me, so it didn't used to be… it used to be only one person came to a session, but now it's compulsory for every adult to come to the information sessions and they said they had a spike in domestic violence, people reporting domestic violence and that wasn't because there was an increase, but because people then knew it is against the law and they could report it, so I think that's covered quite well now.

Interviewer: Within the first five years, any key ones?

Respondent: I think there's such a variation. With young people, we're seeing different levels of issues come through like drug related offences, gang-based and

legal knowledge for young people once you have a record, what that means as well. Youth workers have said they've seen trends increase with drug use and joining gangs.

Interviewer: And in your opinion, what level of awareness of migrants, to accessing key legal services?

Respondent: I think here it's quite here because they know we have outreach. I don't know about elsewhere, so I don't know if we said we're full, which we sometimes are, what people's knowledge would be beyond Legal Aid. We have the (NAME OF LOCATION) Legal just down the road, but people prefer Legal Aid.

Interviewer: What sort of challenges do clients of your service face when accessing legal services?

Respondent: I think cost would be a big one, but also expectations of what a legal service can do, so I think for our advice clinics here, people might want to bring over a family member from overseas and the immigration advice keeps coming back with the same thing and not being able to understand the boundaries of what the service can provide.

Interviewer: The next set of questions are around clients moving from one place to another. So, what do you think the reasons are for the movement of clients from one place to another?

Respondent: As in geographically?

Interviewer: Yes, from one suburb to another.

Respondent: We don't have many leaving (NAME OF LOCATION), but a lot coming to (NAME OF LOCATION) as that's where the community is. Previously we had a lot come from (NAME OF LOCATION) or (NAME OF LOCATION) as there's the Arabic community. A few clients that have moved out of (NAME OF LOCATION) have been because of fractions with them and the community or for cheaper housing.

Interviewer: The next set of questions are around migrants' access to education and other programs. Can you tell us about some of the services available to migrants using your service in terms of school education for their children, adult literacy programs and any other educational literacy programs?

Respondent: With the children, the high schools we have around here are great, so they all have I.E.C.'s, Intensive English Centres and they have wrap-around services for the young refugees and migrants. A lot of outreach work in the schools. I think one of them, they were having to say no as they were

really overwhelmed. We used to have a homework support program here. We stopped doing it as they're doing it at school. In the primary school there's a gap. Vinnies used to do a program for homework for kids, but that's stopped now, so I think there's a gap in that space, compared to what there is for high schools. For adults, there's the A.M.E.P. classes that are Tafe there. We get mixed feedback. When there was the increase in settlement, people were saying the classes were huge, everyone spoke Arabic and you wouldn't learn anything and I think sometimes that's a challenge when everyone in your local area has the same language as you, it's challenging.

Our migrant populations can be better if they're a mixed speaker, mixed classes. We do conversational English classes here in the library and there's a mixed uptake in those. Seniors come along just for a social catch up more than the English part there and we did do a Connect English Program that linked people one-on-one, a really great program, but was only funded for a year. Someone used to do a tutoring program, but had long wait lists and was really challenging. I think a lot is done by Skype now if they can't attend classes.

Interviewer: What do you think some of the key issues and challenges are for children of your clients in terms of accessing school at a University education?

Respondent: I think the big challenge is for those around 17, 18, that have missed schooling and particularly when it was really busy here and the I.E.C.'s were full, they just weren't getting able to access and the nearest seniors college is (NAME OF LOCATION) which for some people is another whole world away and accessing school if you're a bit older or even if you're 19, that can be challenging for our young people and again, that would be the same if you wanted to go on to Uni as well. We have had a few families had their children go to Uni which is great and exciting. It's the unknown when going through that system and I was talking to a youth worker last year, the work he did was in regards to enrolling people to Tafe or Uni.

Interviewer: Great. Can you tell us about any special packages or subsidies, provided to support educational opportunities?

Respondent: I know there's some grants available. I know S.S.I. has one and I know for a while some of the Uni's have subsidised courses for people who come from a migrant or refugee background, but I don't know much more than that.

Interviewer: Are you able to outline the employment opportunities that you're aware of, that are offered to migrant children when they finish school or Uni?

Respondent: What they’re going into mainly?

Interviewer: Yes.

Respondent: Construction type work, not necessarily with as many qualifications. I think warehousing was a big area for young men, but I don’t have that many people I’ve engaged with.

Interviewer: How about employment opportunities more generally for migrants and refugees?

Respondent: People are getting into aged care which is great and I think is more casual work. It’s not as stable for our communities. People are going into community welfare and people might come from an engineering background and go into community welfare which there are pros and cons. It’s that they can’t go to their chosen field and skillsets and their language ability to be going into that as well. People go into interpreting, counselling. I’d say aged care is something people are getting into quite a bit at the moment.

Interviewer: Overall, what do you think are the key challenges for migrants you work with face while adjusting to the Australian culture and settling in Australia just generally?

Respondent: I think just time. there’s an expectation that people come and they’re ready for everything straight away, when really, like all of us, they need time to settle in somewhere and depends on someone’s journey and their ability, how long that takes can vary. I think a big issue is no matter how much is being invested here in Australia, half of them is constantly overseas and trying to talk to their loved ones, there’s been a lot that have been separated, separate from kids, so that’s an ongoing challenge people don’t necessarily take in to account in someone’s current settlement journey.

Also, their own expectations of what it’s going to be like to settle, those rose tinted glasses sometimes and the reality can be quite different, particularly for people who have been employed, had houses and then coming to somewhere where their status is quite different and people like or don’t like being labelled a refugee as well, so knowing you want a job right now, but might take time in that space as well.

Interviewer: That’s great feedback and finally, what would you like to see as possible solutions to helping or supporting migrants to adjust well to life in Australia.

Respondent: I think funding cycles that don't last. There's programs that come up and people get settled in to and they enjoy and then it just changes all the time. I think for a lot of our refugees, they've gone through a lot and stability would be helpful. I think a high-level case management program helps the most vulnerable to settle properly. I think with all the changing systems happening in Australia, people need a lot more support sometimes than currently what's being provided, to really be able to get the best and I think having that early intervention. There's no-one that looks at things holistically and a lot of our clients that go to the G.P. or have financial issues, they don't understand necessarily there's ten different services for your problems. I think one place they could go to, to get that support would just help everything and you don't have to remember then ten different caseworkers as well as doing everything else, so I think that would be really helpful.

Interviewer: That's great input. Thank you. That concludes the questions. Is there anything else you'd like to add?

Respondent: I'm sure things will come to me as time goes on.

Interviewer: You've covered a lot of ground, it's been fantastic the points you've made. Thank you, it's really valuable information.

Respondent: It's good to talk. The more removed you get from clients directly, you don't get to talk as much about things going on, so it's good in that space as well.

Interviewer: And you've been able to reflect on it with your time off. It's great, thank you, I really appreciate your time. We've finished the interview at 11:20a.m. Thank you.
