# Supplementary material for: The importance of information acquisition to settlement services literacy for humanitarian migrants in Australia
Source: PLoS One. 2023 Jan 6;18(1):e0280041. doi: 10.1371/journal.pone.0280041 (PMC9821785; doi:10.1371/journal.pone.0280041)
Supplement: S1 Data — (ZIP) [file pone.0280041.s003.zip › SP_03_Victoria.pdf]

Interviewer: Alright, so this is (SERVICE NAME). Interviewer (INTERVIEWER NAME) with…

Respondent: (NAME) [indistinct 0.12].

Interviewer: Excellent. Thanks (NAME).

Respondent: No worries.

Interviewer: Thanks a lot. So before, I mean you've already mentioned it anyway, but just to clarify, this survey is particularly concerned or specifically concerned with new migrants within the first five years, both forced and voluntary migrants. So refugees and migrants. So the first set of questions is about services that (SERVICE NAME) currently provides and that can be in terms of education, health, social, legal, or any services. So can you tell us a bit about the services that are currently being provided? So I guess rather than specific, because obviously you have a lot of programmes, kind of a bit more broader in what service areas you work in.

Respondent: No worries. So I might just start off with a little bit of history just to give a bit of scope if that's OK.

Interviewer: Yeah, for sure.

Respondent: So (SERVICE NAME) have been I suppose an established entity since, I can't remember the year, but 38 years ago. And with that we kind of developed, it was more of a localised settlement service, and so that's been our number one primary service for the past 38 years, is engaging the local people and migrants who are coming to Australia to help them settle here. And so then over the years we've developed that. And so currently we are, we have various different services just in light of how government has impacted various funding and, you know, various policies have impacted migrants as well. So we have Settlement Services, which is, we have a Humanitarian Settlement Programme working with people who initially arrive to Australia. General Settlement Services for people up to five years. And then we also have Family Services who can work with people beyond the five years or from day one beyond, you know, five years if… anyone who's from a migrant or culturally and linguistically diverse background. And then we have our aged services through homecare packages coming off Home Support Programme as well. And we do some disability support coordination. We also have a social enterprise called Multicultural Home Support Service and that provides… and support, Home Care Support, personal care and home care to either, you know, to people in their homes. So that could be… majority of that work is working with older migrants and, older as in age, and then, yeah, and also people with disability.

Interviewer: So do these disability services or family services that you provide, they're obviously for the general migrant population, but are they also for migrants within the first five years as well?

Respondent: Correct. So yeah, so they're for general population. And so we have a breadth of migrant populations that we work with. So it could be the older… so people who've been here long-term cohorts. So you know, Italian, Greeks, yeah, Macedonian, Croatians, yeah. They're our longer-term migrants. Vietnamese, Chinese as well. But those who have been here, you know, shorter-term, if they do, they can access those other services if they, you know, need aged care services or the people who have a disability. Yeah.

Interviewer: Awesome. And when… your primary services, are there any organisations, any other service providers that you collaborate with or cooperate with in primary services?

Respondent: Yeah. So we have various partnerships or we have various contracts, partnerships, MOUs, and, you know, service level agreements with, I don't know, I haven't read my annual report yet. So you know, there's hundreds of service providers that we actually work with. So in terms of Settlement Services, (SERVICE NAME) are subcontracted to AIMS Australia, who's the lead contractor for Humanitarian Settlement Programme. (SERVICE NAME), so we… that's how we provide initial settlement support to refugees or humanitarian entrants arriving in the (NAME OF LOCATION) suburbs in five LGAs. And then with General Settlement Services, (SERVICE NAME) is the, I suppose, the biggest sole entity providing General Settlement Services in I think it's nationally but, you know, number one in Victoria. And I say that because someone like SSI, they subcontract to 24, you know, different subcontractors. So yeah.

Interviewer: SSI is…?

Respondent: Is a, sorry, Settlement Services International.

Interviewer: Yeah, OK.

Respondent: Thank you.

Interviewer: Do they operate in Victoria or just New South Wales, right?

Respondent: New South Wales. Oh, they do have some programmes in Victoria as well.

Interviewer: OK.

Respondent: And so part of that is… I suppose part of the Settlement Services, I'm not sure if I'm going too far or how… we have Community Support Programme as well. So we're working with community members and,

or what we call assurers of support, in Melbourne to bring people over here. And I suppose that's, Community Support Programme is where the community actually support the settlement of people who they know, the humanitarian entrant, rather than the government supporting through the Humanitarian Settlement Programme. OK. So that's like a paid, a user-paid service as well. So we help with the applications, we help with… and the settlement. So there is phases that, you know, that would go through. So, and that also includes liaising with immigration about visa applications and if it's valid or if it's not or, you know, and so forth.

So in terms of how Settlement and Family Services is broken up in, at (SERVICE NAME) is that we have, we've just gone through a bit of a, I suppose, a restructure. So we've got our Integration and Family Support. And this is our case management. So we work one-on-one with individuals and families at any stage of the settlement to make sure that they, yeah, if they need case management that they settle positively here. We also connect and we have group work and community development work where, yeah, we can connect our, connect people to groups such as Parenting in a New Culture, which is, which (SERVICE NAME) holds the IP for. So that's a programme where people can come learn about how, you know, their parenting skills, developing them, what the conflicts with, you know, Australian culture and, you know, maybe do a bit of myth busting as well. And so that's a really important programme that we run. We also… but there's also other group work that we do. You know, we have community gardens, play groups, English conversation groups. You know, we used to do driving groups or, yeah, or information sessions and, you know, just social groups as well.

Interviewer: So there's really quite a lot of services on offer?

Respondent: Yeah.

Interviewer: And are there any services that are needed but are not available that you recognise? Anything that comes to mind?

Respondent: I think what we are working on at the moment more specifically is around employment and education. So we do, we are partnered with say Brotherhood of St Laurence to provide Youth Transitions Programme, which is assisting youth to access employment and education outcomes. And we're also partnered with MRC (NAME OF LOCATION) to provide Skills First Reconnect, which is, you know, engaging people between the ages of 15 and 64 who have been disadvantaged some way in, yeah, during employment or have had disrupted education, yeah, to get into pre-accredited, to enrol into pre-accredit training or gain employment. So I think this is something that we're working on and so we've just recently have… we've just recently partnered with Refugee Talent, who are basically a recruitment

agency to provide employment opportunities for newly arrived migrants in Australia, yeah.

Interviewer: And so are any services over-utilised currently? Being used too much do you think?

Respondent: No.

Interviewer: Anything, any under-utilised then?

Respondent: Not under…

Interviewer: So they're available…

Respondent: They're available. Nothing's under-utilised. Yeah. So over-utilised, you know, we could always be doing more. So yeah. So…

Interviewer: So the utilisation, then being, the services that are there are being used and obviously, you know, I presume do more if you had more funding, et cetera, et cetera.

Respondent: Yeah, exactly.

Interviewer: Yeah. Alright. So the next questions are related to how migrants adjust to Australian culture and society and the kind of issues and challenges that migrants might be facing from your perspective. So can you tell us about your understanding of how migrants you work with understand Australian culture and society or what level their understanding is?

Respondent: So understanding the process that clients go through, I suppose we call it Settlement Service Orientation. So in the Humanitarian Settlement Programme understanding settlement services is called Settlement Services Orientation. You know, just more like a module really of Australia. So pre-arrival to Australia, people have orientation or should have orientation. I can't remember what the acronym stands for but it's AusCo, right. So they go to an AusCo session, they learn about Australia. But that's really, really general in terms of, you know… and so what we often find is when people actually arrive here then they have to know the localised way of doing things. So you know, orientation to Melbourne metro is totally different to somewhere in a regional area or even different to another state depending on wars (?) and so forth. You know, an example being when does my child go to school? That's different depending on the state. So in terms of… can you repeat that question? Sorry.

Interviewer: Yeah. Just about what's your understanding of how migrants that you work with understand culture and society here in Australia. Yeah.

Respondent: Yeah. So… and I think it depends on how, depending on what they understand depends on what connections they also have in Australia. So maybe there's big connections here and, you know, in the (NAME OF LOCATION) LGA we have, you know, majority of clients who are coming here are Iraqi and Syrian background. Having said that though, it's, yeah, so what clients get to know is from the community and from their connections. And then, and then really it's the younger people, once they start going to school and so forth that they start really getting to know our service systems and so forth and what it's like to be in Australia. Whereas it may take longer for older migrants to be able to engage in service systems to be able to understand. So say for example, you know, they'll be referred to, you know, medical doctors or so forth. So they go through that process. Enrolling into school, engaging in job active service providers when, you know, if they're job seekers, which most are in the initial term. But it's really, it's a learning and so… people may have these expectations on arrival but really soon, you know, a few months down the track it's like, OK, this is totally what I didn't expect. And you don't know until you're in it.

Interviewer: As in the culture and the society is not what they expected?

Respondent: Into this culture. Yeah, exactly. Yeah. Yeah.

Interviewer: So it's very contextual. So it depends on age, it depends on probably your family connections maybe or…

Respondent: Exactly.

Interviewer: Or the community that's already established.

Respondent: Yeah.

Interviewer: Excellent. And so is there a lot of opportunities or maybe a few opportunities for new migrants to practice their own culture?

Respondent: Are there… well, it depends on the established culture that's here. People can practice their culture. We… and we actually encourage them. So you can check out our Refugee Week video online. You know, that really shows that people do practice their culture and… but it takes communities, a strong, united community to be able to do that. You can't practice your culture on your own, yeah. And so there's also, you know, different, you know, different… other factors that might inhibit people practicing their culture and then that could be either lack of disengagement or, yeah, just engagement from their culture or… but it really takes… so that's why, for example, I was saying before in our Parenting in a New Culture Programme we don't say, this is how your parent in Australia. It's like you already have parenting skills, these are some of the laws you may need to know in Australia or this is, these are some of the systems that you may need to know about in Australia. But we don't want people to all out lose their

culture because that's not going to help their settlement. So we're always encouraging people to explore their culture and how it can be practiced in Australia as well. Yeah.

Interviewer: So what are some of the, I guess, the opportunities? Like are people… you know, is there opportunities for whatever kind of religious denomination?

Respondent: So there's religious denominations, there's different not-for-profit organisations or incorporated associations that people establish. So say for example, we have another programme at (SERVICE NAME) that we're also partnered with Brotherhood of St Laurence, it's called Refugee and Asylum Seeker Programme. And that's encouraging various cultural groups to explore governance of what it actually means to run a community organisation in Australia or in Victoria. So say for example, we have a Tamil Women's Association, we have… we're working with a Yemeni association, we're working with the (NAME OF LOCATION) Afghan Association, and we're building their capacity to be able to lead their communities forward. So in that, you know, those associations, so they've developed schools or language centres here primarily for their language. Then, you know, and then they have cultural events every year and they take these annual events really seriously in that, yeah, it's a cultural celebration. So there's dances and food and so forth.

Interviewer: Are any cultural groups, I guess, better at, in encouraging or promoting their culture than others?

Respondent: I think it depends on… I'm not going to specifically say which ones. But it goes back to what I was saying about… I think maybe it's the perception that we might have of people from different countries, yeah. So you may have a country, you know, and they have a particular… but they may have different ethnicities within that country. So while things may be similar then there's also something that's actually specific to their ethnicities. So it could be say, you know, that's colonisation for you. But it could be like Iraq, say for example, you've got the Kurdish, you've got the Islamic Arab people, and you also have the Assyrian Chaldean. Or you have South Sudan, which has 64 language and tribal groups within South Sudan or in Ethiopia you have the Oromo, then you have Amharic, you have the Tigrinya, you have Tigrinya, so do you know what I mean? So even… so yes, while there might be nationally that state is, you know, has specific cultural elements to it, then they've also got various different ethnicities that also explore their culture. So I think it's always that tension around that. So I can't really specifically say that this cultural group from South Sudan is amazing, that doesn't talk for all of that country. So it depends. So it goes back to how united a group of people are. Yeah. And how united a group of people are and how they encourage cultural engagement. Yeah.

Interviewer: Excellent. And a related question to that, are there any challenges that you notice that, I guess, plays a barrier to new migrants practicing culture?

Respondent: Are there any challenges? There's lots of challenges and it depends on the cultural group I suppose. So we still, you know… I think the challenges would be not necessarily ignorance or naivety, but, you know, the concept of the other. People, there are sometimes people who are scared of knowing the other and who that is. So that could be a barrier. So say for example…

Interviewer: So people already in Australia or new migrants?

Respondent: So people already in… or it could be the same either way. But people in Australia who are already here, these people are coming to our country, why can't they get to know, you know, the laws here or something. And then, you know, and then also media can target various groups as well and then that impacts people's settlement. Also…

Interviewer: So that puts pressure on new migrants in they might not feel comfortable to practice their culture?

Respondent: Yeah. And so say for example, you know, the most recent one being what's happened between Turkey and the Kurds. You know, I was very mindful that, you know, there could be people who are impacted or some clients who are impacted by such, you know, international stories and circumstances. It still impacts people here and I think that's something that say local Australians don't necessarily understand if they're not from a migrant background that even international politics or circumstances or whatever their family may be going through overseas still impacts people here and their settlement. So say for example, we have migrant services here, migration services at (SERVICE NAME) and we've got three programmes that kind of facilitate that. So through our settlement funding we assist with some family reunification because that's core to people's settlement in Australia because they just want to be, yeah, they just want to be linked back together with their family. We also have the Community Support Programme, which is assisting people to migrate here. And we are also subcontracted to the Settlement Services International providing IAAAS, just put IAAAS, which is, you know, migration advice and assistance for people who are here on shore seeking protection.

So migration and issues with migration are really, really important as well. In terms of the other impacts and challenges, it's OK if you're… you know, it's usually the general ones like English and so forth. But now that there's a stronger focus on people gaining employment and under the Targeted Compliance Framework, which is a framework that job active service providers need to adhere to. There's a stronger focus on job seekers graining suitable employment. Sometimes when people

come their expectations are, I want to… because currently we have a lot of highly skilled professionals coming. Engineers, doctors, teachers, yeah, very highly skilled people coming. They just want to slide right back into that. And so that's another challenge. The challenge is dealing with people's expectations around how you go about settling here or what's the pathway to gain what I would call meaningful employment, not just suitable employment, which is anything to give you money, but meaningful employment that would, you know, really assist with your settlement and hopes and aspirations. Yeah.

Interviewer: So now I'm going to talk a bit about sense of belonging and inclusion in Australia. Do you have any programmes here at (SERVICE NAME) that kind of, I mean probably Community Support Programme, you've probably mentioned some, but what are those programmes that support sense of belonging and inclusion for new migrants?

Respondent: So I think we gear a lot of our programmes to create a sense of belonging and inclusion. And I think it's… sometimes migrants may come here expecting I'm going to get… so it starts with day one. sometimes migrants come here expecting that they may get a case manager who speaks their own language. You know, and that's not necessarily the case because of resourcing and so forth. But of course we need to use interpreters and so forth. So it starts with that, and it's like, no, well, we need to make sure that we're inclusive of everyone. But we'll, you know, do all we can to make sure that you understand and, you know, services or how to navigate Australia with someone who speaks your language. You know, we have Youth Hub Club. So we provide a space for youth aged between 15 and 24 and anyone can come to that. And you know, majority of our programmes we have to reflect on, OK, how do we develop the English acquisition of people? And so making sure that people feel welcome and in that well we may have, the majority of the time we might speak English, we have like… our Youth Hub Club has an advisory committee where they're the leaders. Like the youth become the leaders of the actual group and the system and facilitate other youth to feel, and new people, to feel welcome. Yeah.

So it's giving people like leadership opportunities to then, you know, assist other people to feel welcome and belonging. And I think with more established groups it's, yeah, yeah, in terms of their belonging, that just naturally happens. You know, in terms of newly arrived migrants, we connect them with their cultural specific groups. But it's also in other settings that we create belonging or events that we create belonging, saying it's OK to be of a particular background because you can all celebrate together. All these different backgrounds and cohorts of people can celebrate together. So really using, you know, say Refugee Week as an example, to be able to create a sense of belonging of all cultural groups. Yeah.

Interviewer: Excellent. And who are the kinds of key people that you see new migrants going to for emotional or social support when needed?

Respondent: Yeah. So depending I suppose on what phase people are at. Now, you know, in this research it's my understanding that we're focusing on migrants who have been here within the first five years. So it's really about building those connections and there's different people. So it could be humanitarian… so if we're talking about an organisation it could be a case manager that they've been working with. It could be potentially they've got referrals to, you know, if, you know, mental health services or so forth. It could be just having a chat to a teacher at their English school as well. Or it is relying on community. We also have bilingual or multilingual workers here as well and sometimes it's really difficult for them because they could be in any space and people know that they work with Settlement Services or a settlement provider or they work with an English school and so they'll go to them because they know the systems as well. So I have lots of staff here who kind of get caught up sometimes in, you know, doing their shopping but they'll see a client and they'll engage them in a conversation. So I think like, yeah, bilingual workers or multilingual workers are really important and they manage a lot in terms of people's emotional support. Yeah.

Interviewer: And so these next questions are about any programme (SERVICE NAME) runs that are responsive to health and wellbeing of new migrants. So do you have any, yeah, do you have any programmes that you kind of implement around health and wellbeing?

Respondent: Health and wellbeing. So we do and usually… we used to have a refugee health nurse collocated with us. Currently we don't. And they would provide initial support to newly arrived migrants coming to Australia, especially if they have any critical health needs. But it's really how we provide health and wellbeing is connections with other stakeholders. So say for example, in our (NAME OF LOCATION) office we're collocated with Foundation House. Foundation…

Interviewer: Also here, right?

Respondent: And here. Yeah, down… yeah. In (NAME OF LOCATION) it's like one building but here it's just downstairs. So we have, it's about making sure you know those connections and also we have good connections with various general practitioners as well. Yeah.

Interviewer: So through referrals kind of thing?

Respondent: So through referrals. But it's also through relationships and understanding who the best person may be to, you know, to go to. Yeah. But it's through referrals generally.

Interviewer: And so those…

Respondent: But… sorry. Just in terms of our… we also have planned activity groups and that's usually run, they're run through our aged, the aged team. And so, you know, that promotes health and wellbeing as well for older migrants to be able to run, engage…

Interviewer: The older new migrants? Sorry…

Respondent: And it can be older new migrants if they're referred into those programmes and so forth. Yeah.

Interviewer: Excellent. And so perhaps you don't run health services but the ones say that you might have connections with, do you ever, do you have, do you have a feeling about what some of the enablers or barriers to accessing those programmes might be?

Respondent: I think in terms of when it comes to anything around mental health there's always usually this cultural stigma. Sometimes it takes people a while to engage with say mental health services as well. But in terms of general health practitioners and services, that's fine, they usually engage with them. Also, there's also… I haven't really kind of dived into research around this but in terms of youth and engaging in health services it could, potentially that they would engage with, you know, school health nurses and so forth. And there's a lot of stuff like that I've learnt through my refugee health nurse that was here that migrants don't necessarily know or young people don't necessarily know and it's only until you're engaging with the school health nurse or so forth that they, yeah, understand. So it could just… yeah, because you know, there's various topics that may not be discussed within the family. You know, that could be, you know, sexual health or it could be for, you know, LGBTI, you know, specific issues and so forth. Yeah.

Interviewer: Great. So the next questions are about programmes that (SERVICE NAME) offers for financial literacy or income generation, management of money.

Respondent: Yeah. So we… so one of the foundation outcomes for Humanitarian Settlement Programme is money management or managing the money, I can't remember. And so we do have, there's orientation around that, how to manage money. We do have specific programmes, we're not currently running any, but around financial literacy as well. We also have here collocated with us Gambler's Help, because it's been identified that with Somalian communities that gambling is a real issue. And so therefore there's a financial counsellor that we can refer to in that case. And we also refer to other financial counsellors located here as well. So not at (SERVICE NAME) but at other organisations. And otherwise it's also education either one-on-one. We sometimes…

Interviewer: Is that through case work?

Respondent: Yeah, through case work. You know, we work with some very complex cases where, you know, there's been various, you know, circumstances such as domestic violence or… and so forth. Then potentially, you know, a family member may be subject to financial abuse and doesn't have any financial literacy so it's actually working one-on-one with them about, OK, what's a budget, how do you pay your bills. And so there's various points where we engage with people and so trying to understand their financial literacy and why, like, if someone comes to us during our intake times and says, I can't pay my bill, can you help me get a utility grant, then we can do that. But then we go further asking the question, why can't you pay your bill? You've been here three or four years, what's lacking in your understanding? And so usually we identify trends in that sense. And then potentially then establish a group around that or, yeah, engage with people in a group setting if they want to do that, yeah.

Interviewer: And so what kind of challenges do you see new migrants facing in terms of financial challenges, sorry, when coming to Australia?

Respondent: Financial challenges. So it is around, OK, the cost of living, what does affordability mean? It's usually around things like housing and employment. And just this, just the mere cost of electricity in Australia is huge compared to, you know, somewhere back home. So say for example, you know, learning a different environment as well. So during the winter it could be that you're running a gas heater for three months straight 24/7 and we've had people come to us with, you know, thousands of dollars' worth of bills. Yeah. So it's not just about financial literacy, it's about, OK what's impacting your finances? So OK, I don't want you shivering during winter but how can you go about reducing your bills, reducing your resources, not resources, your energy and so forth. And yeah. So it's not just about how to go about paying for that. It's about, OK everything costs money, you know, so how do you go about… you know, I need money for Myki, I need money for food, and their understanding. So what is hard is the understanding of how much things actually cost and then implementing that and making sure that you're budgeting.

The other, in terms of financial literacy or engaging with finances is that most people when they come here they become, they go on New Start allowance, yeah. Or for youth they can go on Youth Allowance. And they may get parenting payment depending on the ages of their children. But it's understanding that. OK, how do we go about, yeah, understanding how much money they get a fortnight but what obligations they may under, you know. So say for example, currently there's an exemption for refugees to be engaged with a job active service provider until six months, yeah, of arrival. After six months, or on the 26 weeks, they're referred to a job active service provider and so then they have to start their mutual obligations. Now this, just to note, this is changing from the 1st of January next year where there will be a year exemption for people to engage with a job active service provider.

And so, you know, the idea around that is to make sure people are getting employment and so forth. But what can happen is that maybe they don't meet their obligations and therefore they go into financial hardship because…

Interviewer: If the payment gets cut off?

Respondent: If payments are suspended, suspended or cut off all depending on what has happened. And they may take a while to re-engage. But in that people, you know, may go into financial hardship and therefore we need to have good connections with other organisations who provide emergency relief to then, you know, make sure that people are assisted or advocate somehow. Because it could have been something simple. So that's another role that we would take in terms of, yeah, people's employment (?).

Interviewer: And are there any cultural specific financial challenges? For instance, having to send money back home or dowries or…?

Respondent: Yeah, that's [indistinct 38.56]. Dowries haven't come up lately. Or yeah, in maybe in the South Sudanese community but I don't know if you can call them a newly arrived migrants. Maybe there's some, there's only triple [indistinct 39.13] but yeah, dowries or yeah. But majority, you know, people sending money back home is a significant one. Because, you know, you're here and you feel obliged to support. Having said that, yeah, I also know migrants who have some animosity against that, like a resistant sending… they've been here for a while now and it's like, I just can't… I can't just keep sending money back home, so how can I bring my family here? Or why can't they come? So there's all that. And sometimes, you know, they go through great stress as well because it can be a huge stressor for people if they can't survive in Australia as well with the money that they've got but then expected to send money back home as well. Having said that, there are people who do have huge assets back home or have had to sell assets as well so they might be able to be more financially viable in Australia. Or there could be people who have assets back home but can't actually touch any of that at all. So they, you know, that could be potentially another stressor in that… yeah. It's like, I have this money, I have this asset back home, if only I could sell that.

And so sometimes people do go back home and sell businesses but that puts… or back to a second country close to home. But then that, if they attempted to enter their country of origin, where they're fleeing from, then that could potentially put their visa at risk. So…

Interviewer: Their visa for here?

Respondent: Visa for here. So their permanent, yeah, their humanitarian visa.

Interviewer: Yeah, right.

Respondent: Because, you know, yeah. You know, part of that lore is that you shouldn't be returning back home, you're seeking protection from there. So therefore there's, you know… you're safe here, you're jeopardising your safety. And so then, you know, immigration may question the validity of their case.

Interviewer: Yeah. So the next questions are about legal support or services that you offer here for legal support. Yeah, do you provide any?

Respondent: We don't necessarily provide legal support specifically. But again, we handle the legal services located here on site who provide legal support. Yeah. We do provide migration advice. So I suppose that's legal at times. So yeah. So yeah, we do provide migration advice.

Interviewer: So that would be to do with visas, inviting families to Australia? But in terms of other things like if there's any legal issues out in the community or any violence or anything like that, that would be referred to a legal service?

Respondent: Yeah, referred to. Yeah. But we would still want to engage in that to make sure that it's culturally safe to do and so and yeah, and you know, or depending on who, what we call, who holds the clients. So if I'm working with a family and they need legal support then we would facilitate that, depending on what the issues are. Yeah. Might not. Just sometimes it's not just a referral, it's like we actually have to facilitate the engagement. Yeah.

Interviewer: Yeah. And what are some of the key kind of laws or provisions that new migrants might need to come to terms with when settling in Australia?

Respondent: New laws?

Interviewer: Yeah.

Respondent: It's everything. So new laws. Yeah, it's really everything. So I think the biggest one though, and I don't know where this fits, and depending on the cohort, but sometimes laws around child protection. Are we still going?

Interviewer: Yeah, yeah. I just, my biggest fear is…

Respondent: That it just stops.

Interviewer: It would stop and then… anyway…

Respondent: It's OK.

Interviewer: That's my biggest fear.

Respondent: And that's OK. So around child protection, what that means. People think, oh, you know, if I just smack my kid or if something happens to my kid then my kids are going to be taken away from me. And like… and so there's, at times there can be a real fear around that. Or it could be just the driving laws or fines. Like we will do a lot of advocacy around fine infringements or if, you know, either fines for driving or usually it's around public transport as well until people know the laws. Yeah.

Interviewer: Not having the right ticket?

Respondent: Yeah, not having the right ticket or not having money or just needing to get the train. Yeah. So yeah. And you don't… and no matter how much you speak to people about this, it's not until they're in it and it's like, oh OK, I get it now. Yeah, right. So yeah, it could be anything from civic laws to, you know, criminal laws that, yeah, people need to get used to.

Interviewer: Alright. And are there any challenges for new migrants in accessing legal services?

Respondent: I think the biggest challenge would just be their understanding of legal services. So if they have someone like (service name) who can facilitate that then that's OK. They can go to lawyers from culturally specific backgrounds as well or that could, depending on what the issue is they may not want the community to know. There's also a fear around community knowing personal business. And yeah, there was another point I was going to make. Yeah, access. But yeah, it's really around making sure that people have linguistically, people have, yeah, translators and interpreting services as well. Yeah, specific. So when we're dealing with clients we want to make sure that… there's different classifications of translators as well. So if we're dealing with anything with complex medical issues or legal services then we want to make sure that we have the highest level of translator who would be able to articulate that appropriately. Yeah.

Interviewer: Good. So these questions relate to mobility of your clients. What are some of the key reasons why new migrants might be moving from one suburb to another?

Respondent: OK. Mobile, not mobility aids. Mobility.

Interviewer: Yeah, sorry. [indistinct 46.22]. Yeah.

Respondent: Well, this has actually been a question that we've been thinking about because… OK, so you might have read recently or heard or I'm sure you read the news around, you know, regional focus for employment and migrants, right. When it comes to humanitarian entrants, usually

they have a link here or usually they have a family member that they know and they want to come here first for family. And so once people settle in this region, like this particular region, like especially people with families, it's really hard for them to go regional areas or so forth. You know, that's… but then it's hard for them potentially to find employment and yeah. But there are… usually people move because of community. So we have people who arrive up here in the north but actually their community is out in the west of Melbourne. So there's a lot of transience between west Melbourne and the north. Usually people… I haven't, yeah, I haven't really had anyone transfer from, we call it a transfer, sorry, from the southeast to the north. Like I think maybe that's a little bit more stable. But I think something that we are looking into is how do we get people to access job opportunities that are out there in the regional areas? And this is a real, it's a real difficult thing.

Interviewer: So people moving for work at all or mostly just for their…?

Respondent: Some people will move for work. But when it's a family then it's really hard. If it's a single person or a small family or a couple then it's easier for people to move. But then it's like, OK, where's my community? And they still might travel back to Melbourne for culturally specific foods and, you know, spend a day in, I don't know, Footscray or Dandenong or whatever making sure they get all the food and then go back, you know. Because, you know, that's not in those regions. So yeah. I think it'd be thinking about where people settle, a thought that I've been having is thinking about people and where they settle initially and having that conversation pre-arrival, no matter who their proposer may be and where they may be living could benefit say regional employment so that people can settle straight away in those regions. But usually people settle where their community are because they feel it's safest. Well, not safest, but yeah, they feel… it goes back to that sense of belonging and yeah, what that means for people. Yeah.

Interviewer: Is housing affordability an issue for anyone out in the north? I've heard in the west it becoming an issue, and southeast actually.

Respondent: Yeah. So you would see that people are moving out as the growth corridors get bigger. Yeah, people are living on the fringes. You know, there's still pockets say within the north that are still affordable. They may be affordable but there's no, you know, there's no supply. So yeah, it's really looking for other… so you know, in the north you've got Craigieburn, Epping. In the west you've got beyond Caroline Springs or down even to Wyndham, Melton, you know. But those, even those people out there depending if business have been set up, they might… the west (?) might go to Footscray or something for their culturally specific foods. Or still people will travel to make sure that they get that community engagement as well. Yeah.

Interviewer: Excellent. So the next question is about migrants access to education and literacy programmes. So what kind of services does (service name) run in terms of literacy and education?

Respondent: So (SERVICE NAME) runs… access… yeah. So there's a few ways that we do this. And we always try… and it goes back to what I was saying about English acquisition. So did you say employment or is this just…?

Interviewer: Just education, literacy, yeah.

Respondent: Yeah. So education, literacy. So we have groups out in the west that we do, which is called All Talk Time, and it's a facilitated approach to conversational English, which is guided by the group. So we have an Arabic men's group that come out there. And I think that's more changing to a more of a mixed group now. We also try and establish our, some of our group work within AMEP service providers as well. But we use, you know, using English. So yeah, so people are always using their English. We also have… so in terms of employment pathways, what's important is education. So people may need education, but even understanding the laws around education and why they can't go straight into university to study that medicine degree, it's like a pathway to get there. So depending on what kind of study people have done before depends on, do they need to meet the Australian standards to become an engineer and if so then you need to go through this educational pathway to do that. For youth it might be a little bit different because they're, you know, they go, they, and children, they go and do their English and then they're slotted into whatever grade. Having said that, if they've had disrupted education before then that's difficult for them to at times catch up and so they need a little bit more of assistance and aid at school.

We have… so we're partnered with Brotherhood of St Laurence to provide Youth Transition Programme and then that's about education as well. So we have a youth worker who would facilitate, you know, who does some case work around getting people into education, yeah, and depending on what they want to do. And sometimes they complete that and then come back and need assistance to getting into further education as well. Yeah.

Interviewer: And are there any key issues or barriers for children of your clients in accessing school or university education?

Respondent: It's… accessing school, not necessarily. Depending on what their experiences, you know, they could be early school leavers and disengagement from school depending on what their circumstances are. But in terms of accessing school, not necessarily. So when they, when people come here, they're enrolled into school within, once they've got long-term housing, which could be, you know, in the first few months of arrival to Australia. So there's no reason as to why someone, like a

child, shouldn't be in school, unless they, you know, might have a medical condition or something. The hardest thing is we have had trouble with people accessing, who have a disability, children who have a disability, accessing schools for them. And that's more the…

Interviewer: The schools don't take them on because they say they can't cater for their disability?

Respondent: Yeah, some schools can't take them on. Or when they, I don't want to use the term, the more appropriate term isn't coming to me, but a special school. Even accessing that, people need to be on NDIS or have a certain plan and, you know, it's… yeah. The hardest thing around that is getting medical conditions and so forth right and documented to be able to access NDIS. So I think that's, you know, in terms of getting the appropriate support in education, it's around ensuring that migrants have the appropriate documentation, yeah, and, yeah, around that. You know, schools and, you know, access… when adults go to English language and they've got younger children, you know, there's free childcare provided under AMEP when they engage with their 510 hours. But we need to make sure that the kids are immunised or have a plan for immunisation as well. So yeah, in terms of children accessing school it's not a huge, or the inhibitors aren't huge. Yeah.

Interviewer: Yeah. And what about employment after school? Is there any kind of special programmes or provisions for that?

Respondent: Yeah. So, again, the YTSP Programme we have. We've also set up a…

Interviewer: The Youth Transitions Programme? Sorry, yeah.

Respondent: Yeah. So we've also set up an employment pathways programme as well at (SERVICE NAME) that can facilitate…

Interviewer: For youth?

Respondent: For youth and adults.

Interviewer: And adults as well. Are there any other kinds of employment related opportunities for migrants generally speaking?

Respondent: So we try to think a little bit creatively around that. And we might be expanding this programme. So for employment it's, our community development worker has been working with a group of engineers alumni. And so yeah. We want to create a network of people with specific professions. So it's not just about, yeah, they've got their… they meet the Australian standards. Even if they meet the Australian standards, they have correct English, and so forth. It can still be really hard for people to get employment opportunities. So meeting together,

the idea of this group is to meet together, identify the challenges, create a network between a specific profession of refugees, and being able… and then they're able to support each other and give advice and so forth. So I think we're trying to think a little bit creatively about how we go about assisting people. Sometimes people come to us and say, just, you know, give me a job. And it's like, well we can't do that. Like and the expectations are quite high. It's like, well we can't do that. So… but we can assist you and coach you find employment opportunities. And it takes a lot of networking to do that and understanding where the jobs are at and yeah.

Interviewer: Alright. And so overall, what do you think are the key challenges for new migrants that you work with in adjusting to Australia and settling in Australia?

Respondent: The key challenges?

Interviewer: Yeah.

Respondent: Like there's a lot of positives, but yeah. The key challenges are generally, you know, your usual lack of English and lack of employment opportunities. And people just taking whatever employment opportunities, which may be… but usually they're the lower paid jobs as well. Housing affordability or potentially… well it used to, about a year ago it was more around overcrowding and housing affordability. And I think it's hard for people to understand the pathways, and this is where we're coming in, it's a challenge for people to understand the pathways to education and employment opportunities as well. Just even something as simple as you even need to judge when to get your qualifications recognised because if you need a pathway or a bridge programme to get into your, you know, preferred employment then potentially if you've already got your qualifications recognised then you'd need to pay a full fee for say a diploma or a certificate or something, you know what I mean? So it's even, like into the detail and so you just can't come here and within two weeks get your, you know, your qualifications recognised because there's an actual process. So I think sometimes the bureaucracy or the red tape that people may need to go through to actually get, to gain, to get into education or employment can be quite arduous and stressors. On top of that, you know, you still have people with traumatic experiences and that can surface at any point, you know, during their settlement period, yeah, as well, which can then impact that.

And yeah, and so I've seen recently a lot of people with mental health issues try to, which impacts their capacity to work or function, try to apply for say disability support as well. And you know, that's been knocked back because their evidence hasn't been sufficient. So it's been… that takes a toll. And so people have to meet, you know, employment obligations or so forth or, you know, might have had to move, yeah. Have to meet employment obligations or job active

service provider obligations when they're actually really having a hard time and actually don't have the capacity to work even though they may look physically well or so forth. The other thing is, yeah, there have been some domestic violence or family violence issues. And like when people, when you have a separated family as well, how maybe various communities take that and the stigma around divorce or separation and what that may mean as well. So yeah, we've had a few instances where... and challenges around that. Yeah.

Interviewer: Alright. And finally, what would you like to see as possible solutions in helping or supporting migrants to life in Australia?

Respondent: I remembered. I remember my further study comment. So in terms of support, other supports or… and so forth. I think the, generally, the settlement period is between the first five years of arrival. Within our Family Services we work with migrants who have been here beyond five years. And so I think there needs to be an acknowledgement of government and policy that people's settlement period doesn't finish at five years. They still experience racism, they still experience discrimination, they still experience other circumstances that, you know, that impact their settlement and that could surface after five years. And so having something like our Family Services is great but that's only limited to families. There's also the concept of asylum seekers or people seeking asylum. They may have been here for a few years and therefore… and may reside in Australia, having said that, yeah, their needs and conditions makes, you know, mean that they can't actually settle here in Australia. And that impacts… and that can impact families. And like having the fear that they may need to return home one day, for example, the Tamil family that's, you know, in Christmas Island at the moment. You know, what impact is that having on the children? What impact is that having on the family cohesion? What impact is that having on the community here onshore? Or… yeah.

So I think there needs to be the wellbeing of our, of people who are arriving here and, yeah, it needs to be paramount to, yeah… yeah, paramount to their settlement. So when it comes to asylum seekers I would really like something further for, you know, in terms of settlement, you know, or just change the laws, again. Like and yeah, the immigration policy, I don't think it's acknowledge of how much it can then impact anyone, whether they're a humanitarian entrant or whether they're persons seeking asylum or, you know, or whatever visa class they may be on. It hasn't really been acknowledged how, yeah, the current immigration policies can impact people's lives.

Interviewer: Yeah. Alright. So this is the end of the interview. Is there anything else you want to add before we wrap (?)?

Respondent: Yeah. I think just working with migrants here in Australia is amazing and you can get some fruitful, amazing stories from, you know,

working with people from different cultures and so forth. And I think (SERVICE NAME) way of being able to engage and participate with them, with various people, just really, you know, is a positive story. So I'm hoping whatever you come up with today or in your research can really impact policy. But yeah. I just want to… I think it's really important to share the positive stories and news of people who are settling. I'm hoping that my answers weren't too negative today. But yeah. Yeah. It's been, it's amazing working in this sector and I just want the sector to grow so that we can welcome more migrants to Australia. Yeah. And that's it.

Interviewer: Excellent. Well, thanks for your participation. We really appreciate it.

Respondent: No worries.

Interviewer: Interview end at 3:28.
