# Supplementary material for: The importance of information acquisition to settlement services literacy for humanitarian migrants in Australia
Source: PLoS One. 2023 Jan 6;18(1):e0280041. doi: 10.1371/journal.pone.0280041 (PMC9821785; doi:10.1371/journal.pone.0280041)
Supplement: S1 Data — (ZIP) [file pone.0280041.s003.zip › SP_04_NSW.pdf]

Interviewer: Okay. It's the 13th of November. I'm in (SERVICE NAME) and I am with (NAME) .

Respondent: Sorry I've signed in with 12th of November. Can I correct that?

Interviewer: Oh right, yeah sure.

Respondent: I've been signing every invoices with 12th of November.

Interviewer: Don't worry at least you got the year right. Sometimes I'm totally out.

Respondent: Yep.

Interviewer: Okay. So let's start. So before we start the interview I just want to note that any reference to migrants or newly arrived migrants applies to people that have arrived in Australia in the last five years or less, and includes refugees, forced migration and voluntary migration.

Respondent: Mm-hm.

Interviewer: So refugees and migrants.

Respondent: Yep.

Interviewer: Okay so the first set of questions are about the services that you provide at (SERVICE NAME) that assist newly arrived migrants. So can you tell us, begin by telling us about the services you provide?

Respondent: So that's general settlement services?

Interviewer: That's general, yes.

Respondent: Okay. So my service is one of the largest settlement services provider in (NAME OF LOCATION). So settlement services assist and a settlement service is funded by Department of Home Affairs. It used to be under of Department of Social Services, now it has moved to Home Affairs and our settlement period definition is very similar to yours, zero to five years. And within this settlement service is a program. We provide, you know I mean if I am to put it, I can be more specific to my organisation, but in general the services that we provide within this portfolio is casework which is advice, referrals, you know maintaining or establishing that connection to other organisations. We provide community development, community engagement support in that we also work very actively with a specific organisation, churches, religious institution, build that relationship with those organisation and hence have a gateway to the larger community. We also, you know we're very focussed around youth. There is a very specific focus on youth settlement, it stand on its own as well. We do consultation. Everything that we, every response that we have as in project and initiative has to be

consulted with the community. However we are actually now moving bit away from consultation at this point in time, because after 2015 especially in (NAME OF LOCATION), after the additional intake (NAME OF LOCATION), we as an organisation felt that (NAME OF LOCATION) and especially refugee cohort were being overly consorted and it was exhausting. So now we're moving towards this co-creation.

Interviewer: What is it?

Respondent: It's co-creating everything together. So getting that voice on our governance, on our steering committee, and getting community leaders on-board that represent their community and also [inaudible – 00:03:35] consulting as, yeah if when needed, not regularly seven consultation a year, but having that sort of informed decision made. That mechanism been included in the governance of the program.

Interviewer: Excellent. How long have you been doing that?

Respondent: It's been happening for a while now. Probably two years. With every project we sort of – it's not possible with every activities to do that, but with every project we try to sort of get that happening now. So that's the majority. But organisational, as in organisation we have our own portfolio. So in settlement we have caseworkers who are the frontline workers who do all the casework, group activities with the client. And then we have in that casework space we have Iraqi caseworker, we have women caseworker, women settlement worker. We have been a family focus, family settlement worker, and we have Vietnamese(?) settlement worker. And we also have a specialist team that focuses on specific issues. We have housing specialist, we have education, employment and training specialist. We have domestic violence specialist, that is funded by FACS but sits in the bigger settlement team.

Interviewer: Wow. Wow.

Respondent: And we have community development and policy officer, and then we also have other ad hoc fundings from State department, from other departments that is complementary to what we provide in settlement.

Interviewer: Wow, it's quite comprehensive.

Respondent: Yes. And we also, in my, so I also manage employment portfolios to implement contract. I also manage disability employment services and our cohort being in (NAME OF LOCATION) is again refugees in that space. And I also manage another employment program which is funded through Department of Jobs and Employment. A Parents Next which is basically making our parents ready to get into employment. Anybody who have kids under the age of six, they can access this program and get themselves acquainted with jobactive model.

Interviewer: Right.

Respondent: Yep, so that's what we do in a nutshell.

Interviewer: That's fantastic, thank you. So what are some of the relevant services that other organisations are providing in terms of helping newly migrated people to settle?

Respondent: So top of my head, health is, you know health is, I mean federally, State-wide you know a lot of money has also been invested in health and that's such a, it's such a critical cha – it's a critical space as well in terms of especially – now before I go into that, I just wanted to mention that being in (NAME OF LOCATION), 95% of our client are from refugee background. The 5% of client that we see through our Vietnamese portfolio are migrants especially because they are on spouse visa. Okay so they come under a family stream visa. So everything that I will be basing on today is from that refugee perspective.

Interviewer: Okay great.

Respondent: So in your second question, look Refugee Health which is funded by, which is State funding. They work with newly arrived refugees from the day they arrived, even before that you know, so they actually, from they actually coordinate with the second settlement country which would be Jordan, Lebanon. They have their health data with them and they work along with the humanitarian settlement program provider which is SSI. So SSI, we work with SSI very closely because after exiting that initial 18 months case management with SSI, all the referrals comes, majority of the referral comes to us because we're the largest settlement provider. So Refugee Health we work very closely with Refugee Health in that physical health space. That could include anything physical but also nutrition, early childhood issues.

Interviewer: And immunisation and those sorts…

Respondent: Yep. All those thing comes there. Now we also work very closely with STARTTS which is in the mental health space. So a lot of our clients have that Post-Traumatic Stress Disorder background, so most of the time we do have to get the referral to counselling and which we rely on STARTTS and the Arabic(?) picking GPs through enhanced primary package, EPCs. We refer them to GP, GP refer them to five sessions of counselling with their Arabic(?) psychologist. So we do it and we also work with Transcultural Mental Health. What else? We worked a couple of years ago, around a year and a half ago, we worked with One Door which used to be called Schizophrenic Foundation before but they changed their name. So it's One Door now. So we do actively seek partnership in that space. And again, so that's just the major, but then we have a lot of other local partners such as

Smith Family, TAFE, Navitas, schools, you know that's all I can think top of my head.

Interviewer: Okay that's fantastic. Can you tell us about who you collaborate to do this work? So that's probably answered already I'd imagine.

Respondent: Well I would actually ask you to have a quick look at (NAME OF LOCATION)'s settlement, (NAME OF LOCATION) City Settlement Action Plan. (NAME OF LOCATION) City Settlement Action Plan. So 2015 when we had additional intake and not so much of resources being given to (NAME OF LOCATION), we the sector, when I say sector the (NAME OF LOCATION) settlement provider and everybody that would be affected by the intake, came together as a sector and then started having that proactive discussion as to how do we increase, you know how do we have a service collaboration on an effective level? We started having that discussion as it is, in order to maximise settlement outcomes and as a result of that the City came together and created a action plan.

Interviewer: Oh great.

Respondent: Three years, two years action plan. Now we extended that by a year. Now that action plan is actually being led by us and (NAME OF LOCATION) City Council. Now in the, through that action plan, we have actually brought together 24, 22 to 25 organisations that are working locally along with the State government to discuss, to point out the initiatives that are being taken. And the progress report was launched in February that also showcased and highlighted the challenges. So now the whole action plan is being evaluated through (NAME OF SERVICE).

Interviewer: Great. Wow.

Respondent: So I really recommend you to…

Interviewer: Who's doing that at (NAME OF SERVICE), do you know?

Respondent: Ann. I think (NAME). Do you know (NAME)?

Interviewer: No. Do you know which department?

Respondent: (NAME)…

Interviewer: I might follow up with you later about that.

Respondent: It would be really, I think that would be really good for you to understand the players(?).

Interviewer: Yes, I want to talk to her, yeah. Are there any organisations that you choose not to work with for any particular reason?

Respondent: I can't think of any. Look as long as our visions are aligned and mostly are non-for-profit, the visions and mission statement and goals and objectives are very similar. So in my experience of being here for a decade, almost a decade, I have never come across that. But if it's a private sector, there are certain things that we do look very closely in terms of vision and alignment and what's the agenda mandate. But again by saying that as well, we also work very closely with business because when we are settlement space, employment is paramount to everything. So we do work very closely with small businesses. So yeah, no I can't think of anything.

Interviewer: Right. That's fine. Are you aware…

Respondent: But, sorry.

Interviewer: Yeah, go on.

Respondent: But there is a, this huge fear of competition. Because what department, what government does is there's always that funding competition you know. The partners that we work with are actually competing for the same fund, same grant. And that could create a bit of animosity probably on a different level, but in terms of working together there has never been a problem.

Interviewer: Oh good. Great. And are you aware of any services that are needed but not available?

Respondent: One of the things settlement action plan brought forth was how less is being invested on domestic violence space. Especially after the enhance(?), after the intake. In 2015 the intake was a bit different to what we had earlier. Earlier we would actually get a lot of clients on 200 visas. So 200 visas is refugee status. So there might be people coming to Australia who had no connection to Australia, had no one from the family in Australia. But after 2015 in order to, because we were taking additional intakes, what the government did was they increased the number of 202s. So 202 visa are usually people who the family connection here in Australia.

Interviewer: Oh right, yes.

Respondent: So most of the refugees being (NAME OF LOCATION) as a settlement hub and Iraqi and Syrian, Middle Eastern background, most of the people that came from on-board on overseas had connection with (NAME OF LOCATION). So in a way it was easier for government to do that because it expedited the whole thing. But what then happened was many women that came with their husband had to completely be reliant on husband sides of family. Hence there was DV, unreported case of DV. Anecdotal evidence of how DV was increasing. So all those things. And we did not receive any enhancement funding. Particularly for services. And I want this to be,

because we do have, there are funding implication anyway so I am sure this is going to be confidential.

Interviewer: Yes of course.

Respondent: But there was no enhancement funding whatsoever for the case management purpose in DV. There were other ad hoc fundings to other, you know telephone counselling probably, centralisation, child protection, but there was no funding on DV case management.

Interviewer: Right, right. Okay great thank you. Are you aware of any services that are over-utilised? Like you know long wait time.

Respondent: Sorry what was that?

Interviewer: Over-utilised, overused. Like in high demand.

Respondent: Oh yeah. So can you repeat that again?

Interviewer: So are you aware of any services that are over-utilised and if so what do you think the reasons are for this?

Respondent: Look I, again I would say domestic violence services. Because the demand, we can't keep up with the demand. And again, with every funding you have contractual obligation, you have KPIs and you can't go really beyond your KPI because then your quality will be compromised and the organisation would be questioned. So keeping and maintaining that demand versus supply mentality is extremely difficulty in DV. Especially given the fact that it is a crisis support as well, not just case management, but it has to have that crisis component attached to it.

Interviewer: Yes.

Respondent: You know so that has been, yeah that's, that's in a, that's, there is a big demand to more services. I would also say refuge. Sometimes what we've seen is again in DV space newly arrived refugees would want to stay in (NAME OF LOCATION) because, I'm just giving an example, that she does not know how to catch a public transport whatsoever and does not want to travel to Manly…

Interviewer: So it's a familiar space, yeah.

Respondent: …for a refuge with her child, six years old child. So I would, we have seen a massive need for refuge you know.

Interviewer: Spaces, yeah.

Respondent: Yep. What else? Because look I think there's also more focus around women issues now. Because what has happened historically and that's always

happened in its own way, when we talk about settlement it's not gender specific. So it's basically one-size-fits-all. However our experience in settlement has always been gender, it has to be gender specific the way male response to some and if changes challenges, it's completely different to women and the timeline attached to it is completely different. So now because as a result of not having that specific focus, 18, 20 years down the line, you know 18, 20 years from when it started, we still have women who can't speak English because they actually missed out on getting to go to these classes. And you know their priorities were very much around kids and school pick-up and drop-off.

Interviewer: And they have less interaction with the outside world.

Respondent: And missing out on a lot of those activities. So now when we saying is let's have women-specific [inaudible – 00:18:23]. And also the fact that reproductive health. We do not get to talk about reproductive health as much as we want to.

Interviewer: I notice the breast screening van was pulling up.

Respondent: Yes so cancer is a taboo. And we don't talk about cancer. We can't talk about cancer. So all those women-specific, it has to have, not just I'm not saying women-specific only but it has to have a gender [inaudible – 00:18:45].

Interviewer: Yes. Great. And on the flipside of that, are you aware of any services that are under-utilised, that there's not a good pick-up on them?

Respondent: Oh I can't think of any.

Interviewer: Okay. No that's fine.

Respondent: Look I wouldn't say under-utilised because there is no need. But I would say under-utilised because the cohorts, at that point in time the cohort that we're serving might not need that service yet. Zero to five years. Now if you tell refugee communities that there's so many children services available, [inaudible – 00:19:38] there and you can go and seek speech therapies and you know all these beautiful thing they do, they might be able to do that. Because, not because they don't want to do that, but first of all they might not understand the concept. If my child is not speaking even when he's four, he's going to speak when he's six. You know, that late, but I think there is a massive cultural clash in context of parenting. The Western and Eastern parenting context. So for that it's probably there are services who are being under-utilised but not because it's not needed. So we have started as action plan and has brought forth that as well.

Interviewer: Oh great.

Respondent: So we've started very progressively looking into, as soon as they come how do we get them informed.

Interviewer: Okay. Perfect. Where are we up to? Can you tell us about the methods that you use to measure the effectiveness of services you provide? So you already mentioned the active participation on boards or steering committees.

Respondent: Yeah. Look we do, we do, you know one of our contractual obligation is data reporting to department. And we do get the data, number of clients participating in certain group activities, and we also assess what group activities is really famous. Some activity that never, that we never struggle to find client is English language in a social setting. So that's there but also the whole department focus and sector focus is also now more into, not just into let's get the data but also towards impact. What, how are we measuring impact and what's happening? So for that we try to impact is very difficult to measure at times as we are all aware. But we actually do post and pre-sessions feedback.

Interviewer: Oh good.

Respondent: So if we're doing anything we get that evidence of what the knowledge, skills. There are certain things that we mark them on. Knowledge, skills, confidence, competence to use access services. So there's certain milestones that we compare their pre-session knowledge skills to post. So and then we score them.

Interviewer: Oh good. Thank you. And can you tell us about any other issues regarding access to settlement services that migrants are facing?

Respondent: Look I think access is always a, it's always been a big question. And I assume it's not, I've seen in DV, I've seen in employment. It's not just in the settlement space but it's in everywhere. Especially if we're dealing with the same cohort. So not knowing the language and understanding the system, it's very difficult. So what our experience has been is, when the refugee, when anybody, you know people from refugee background arrives in Australia, there is that information overload. Everybody wants to do everything with them over that, and every informations are being provided to them. So there is only so much human brain can take. You know so and after that, after they are exited, our service is not mandatory, our service is voluntary. So we get clients when they really need to access us. So in that, you know when they have family domestic violence, school issues, bullying issues, all those thing, they come to us. So they do access when there is a need right? So I think the most important, the thing that we have noticed so far is there's so much of information existing inside us. Every organisation have their website. If you are to know anything about AMEP, you have to go to Navitas, TAFE. You have to go to RTOs and you have to navigate the whole online portal which

is extremely difficult. So what we've noticed is there is no one place where a newly arrived person can go and get away portal. From that portal everything could be linked right, so that's what we've found out. So in response, again I'm just saying this to probably you could have a look at that as well, in response to this (SERVICE NAME), my service has partnered with (SERVICE NAME) and a (NAME) company that has worked, a company called Integrate that has worked in creating a platform for newly arrived refugees in Germany. So what they did was they basically brought everybody together, the municipalities, it worked in the municipalities way. So they with 6, 1 or 6 municipalities, now they have moved across 54 municipalities and all those municipalities are accessing this platform to provide information to their client.

Interviewer: Great.

Respondent: So I think that's one-stop digital shop(?). So that's what we are planning to do and I think that's what's going to, if you're not digitally excluded, if you're digitally literate, which most of the young people are, you've had that access to information. And then you can make a right choice about services.

Interviewer: That's great. That's great. Okay so the next set of questions are around how migrants adjust to Australian culture and society. So can you tell us about your understanding of how migrants you work with understand Australian culture and society?

Respondent: So that's refugees?

Interviewer: Yes.

Respondent: Yeah sorry I'm very, very particular with the definition.

Interviewer: Yes, no that's alright.

Respondent: Yep, so how refugees, can you say that again, adapt?

Interviewer: Or the people you work with which are refugees, adjust to Australian culture and society.

Respondent: Yep, I think they adjust very well. They, our experience have been they're very open, receptive, but what we felt is the process of belongingness can't be forced. It happens. It doesn't happen in a linear way, it happens in due course of time when people are ready. And so until that time we have to lead by example of how multicultural we are as Australian society, Australian cities. And our experience has always you know have shown that how it has worked. I can only base my experience in (NAME OF LOCATION). (NAME OF LOCATION) is one of the most multicultural community where Serbians, Italians, Greeks, Syrians, Iraqi, Burmese, Africans are living

together. So they're very, very open to the idea of being integrated. Probably they're not defining it, but the way they are actively involved in the community and learning from their cross-cultural interaction, that is, that definitely happens. And it's very organic.

Interviewer: Yeah great, great. So I suppose, I'll just read out this question and you've probably already answered it. To what extent do you see your clients are being exposed to Australian culture?

Respondent: Very much. We have a lot of volunteering opportunities where people go out and volunteer. Even our organisation is not just, does not only have settlement focus, it has youth focus. It has aged and disability so there is the [inaudible – 00:28:04] volunteering opportunities across the board. Our client have also participated in most of the activities, initiatives that Council runs that allows that participation in wider communities of network. The Multicultural Advisory Committee that I sit on, 80% of the committee members are actually from that [inaudible – 00:28:31] background of different cultural background. And you know, so I think they're very, yeah, yeah. I think I've answered that, but I think there's that fluidity. I haven't seen now again in (NAME OF LOCATION). I'm not picking on behalf of Greater Western Sydney, but in (NAME OF LOCATION) we have always seen that fluidity. Wanting to know more, that sort of attitude.

Interviewer: Yes, great. And what are some of the opportunities provided to migrants to practise their own cultural values and practices?

Respondent: We are very much focussed on that, because we believe that learning more about your culture, preserving your culture, your language, if we do that then the second generation of migrants, refugees that are coming up would be more confident and would not have that conflicting identity crisis sort of situation that we had before. So for that, that's also the reason we work very closely with the specific organisation. We support language school. We every organisation that we work closely with, we help them in Harmony Day even. We invite all of them to participate in our Refugee Week even, but also participate individually with organisations [inaudible – 00:30:03]. We are very mindful of the importance that preserving your cultural heritage brings to settlement.

Interviewer: And now you've touched on a few points here for the next question. What are some of the issues and challenges around this process of cultural adjustment?

Respondent: Look I would say language. Without knowing language you would not feel, without having language you might have a very slim chance of getting employed. Without getting employed you might not feel very a part of the larger community, you don't feel you're being, you know you're contributing and the hence the sense of belongingness might not, you know but might not

to that level. Might not to be to that level. So I think language is very critical and I think that's why women settlers struggles a lot without having that language. I also think qualification. We need to look at what, we need to start looking at refugees from strength point of view. Not from a deficit model.

Interviewer: Yes.

Respondent: Yeah? So everybody now especially Syrian and Iraqi, most of the Syrians and Iraqi that we had after the intake in 2015, we had lots of engineers, dentists, we had lots of doctors and very, very qualified doctors that have been working in hospitals for 25 odd years. Now, I don't know how aware you are of jobactive? Jobactive is another federally funded program. Now jobactive are pushing this doctor, dentist to be a cleaner, to be some – so I think it's also giving a little bit of flexibility in pathways around what could be done. What is, you know what could be done to get this overseas qualification recognised. And if that can't be recognised, what are the pathways that they need to go through? Without the…

Interviewer: That's right. How best can we use their skill…

Respondent: Exactly.

Interviewer: …and knowledge, yeah.

Respondent: And also support them through that process right? And I think, I mean if we look at our settlement policy and how we re-settle refugees, it's probably come, it's probably on the third best nation to provide that support to settlement in re-settling refugees. But I think our government also start to need to start looking at the Canadian model where community are sponsoring, the community are taking the refugees and have that group of communities to support to get job, to assist them in language. So I think all those models need to be explored and we need to look at, very closely at European model and also Canadian model. Does that make sense?

Interviewer: Yes.

Respondent: I know I've jumped from one to another but I think that's very important.

Interviewer: Great. Now the next set of questions are around this migrant sense of belonging and inclusion in Australian society, and I think you've already answered most of these. But I'll read them out and you can add anything else if you think of anything. Can you tell us about the programs or supports available that help create and enhance migrant sense of belonging and cultural inclusion?

Respondent: Sorry, can you…

Interviewer: So can you tell us about the programs or supports that help create and enhance migrant or refugees sense of belonging and cultural inclusion? So you've already mentioned Refugee Week and broader…

Respondent: Yeah, can I add something on that?

Interviewer: Yes, yeah.

Respondent: I mean I think government needs to have a balance between social integration side of settlement and economic integration side of settlement. The government, the existing current government is extremely focussed on economic integration of refugees and I think the point they're trying to make is when you're employed, when you have employment, then you feel belong, then you, you know everything falls onto your plate. However we can't bypass social integration as well and again to bring you back, settlement doesn't happen in a linear process, linear way. We're not, we don't only have doctors settling in (NAME OF LOCATION). We also have a farmer from North East Iraq with the dry land farming experience and is not literate in his own language. So I think we need to have a policy, flexible policies that actually focuses on both side of integration, yeah, and then have a plan around including having women inclusion more from social integration. Because my fear is if we are focussing so much on economic, we are actually putting them in a box and you know we're actually, we're already labelling them saying they are welfare dependent and they're not going to get off and we have to get them off. That sort of mentality does not work in the long run.

Interviewer: No. Okay thank you. And what are you observations of how your clients meet and interact with people from their own communities, to maintain their sense of cultural connection?

Respondent: Very, very cohesive, very, you know we run a men's group. Usually it is, we found we've actually we've been told by different LGAs, (NAME OF LOCATION) and whatnot that working with men could be challenging because they usually bottle up and they don't want to talk about anything and they would want to deal with their personal affairs at home. But the men's group that we organise on a weekly basis, it started from 20 men now it's grown to 80 men. And some of the men are coming from (NAME OF LOCATION) regularly on a weekly basis.

Interviewer: Wow.

Respondent: What I'm trying to say is they're extremely, they participate very well. As long as they feel that their voices are being heard. And these people are not, sorry to use this word, but they're not pushovers. They come with their own agenda saying this is what we need. As long as those voices are heard, as long as they feel they are on that path of you know, some path of something

greater, finding something to themself, they're very, very, they're very active. They're very active. And only sometimes we do struggle with young mums' participation, but that's not just in refugee cohort, that's across the board.

Interviewer: Yes. Great. Thank you. So the last question in that section is who are the key people that your clients contact for social and emotional support when needed? So community supports as well?

Respondent: So look I think yep, so we have case, in internal procedures, the caseworker works with them from zero to five years. So that is a long time. And they develop a really good connection with caseworkers. But outside the organisation they have specific organisations, community leaders, formal, informal. Not just the president or chair president of their specific organisation. Formal, informal leaders, religious institution, pastor, father, [inaudible – 00:37:38]. They play extremely important role. And they are the ones that the community relies on. And if we look into (NAME OF LOCATION), majority of client settled in (NAME OF LOCATION) are Christian minorities, and they were persecuted in Iraq because of their religion. So they tend to retract back to, if anything happens they go, their base is their religion.

Interviewer: Great, thank you. So the next set of questions are about programs that are responsive to social support and health. So can you tell some of the programs that you're currently implementing to provide social support. And think you've covered some of those already.

Respondent: So we do, you know like I said it's very responsive to [inaudible – 00:38:36] but there are also certain things that never changes in settlement and housing, Centrelink, health issues. That's the crux of the whole, you know doesn't matter where the cohort is arriving from, that's going to stay there forever. But else(?) than that everything that we do are responsive. But social support I look at it from, you know let's have a look at it from the women's settlement program. So we do, for newly arrived refugees we do lots of information sessions but then we're not only sticking to that. We have created different groups that focuses on leadership. We are actually currently, we've organised a leadership training with 10 newly arrived women who worked with this organisation for leadership development and they pitched in their ideas. No their idea is going to, we're going to take further forward their ideas and provide a little bit of seat(?) funding to their ideas and we'll, you know we will see how goes. The other social group we constituted(?) is mums' group, where the playgroup mums, we invite everybody. And because mums are very busy demographic, they have lots going on. So what we usually do is while doing a playgroup we get different service providers on-board. We get Police to come and do storybook reading. We get Refugee Health to do a

session on diabetes and nutrition. So we try to accommodate their time as well as provide them more information.

Interviewer: Great stuff.

Respondent: And then what other? Then we do men's group. We do, yeah so we do, we're very active on social support side of it. While acknowledging employment is important, but we also provide – and then with young people we work very closely with (NAME OF LOCATION) High School, (NAME OF LOCATION) High School. We outreach from intensive in this centres. So before going to the usual regular classes, nearly all our refugees young people are put into intensive English classes, and they're taught, to their level they are taught English. So they have different levels in IEC so we work very closely with them. We take them to excursions. We organise excursions. So yeah.

Interviewer: Great, great. And health and wellbeing?

Respondent: So we work, yep we, in health space we work very closely with again Refugee Health and STARTTS, but we also do ad hoc swimming classes and for mum and bubs and Zumba for elderly clients, you know as long as their health permits but with doctor consent and whatnot. And yeah so we do a lot. The social of health we do it our own way, but the information delivery side of it is always partnered with, because we're not a specialist organisation in that sense.

Interviewer: Yeah, great. And are you aware of any things that work or barriers, enablers we've put it as, or barriers that your clients have experienced when accessing these programs?

Respondent: Yeah. Look the mainstream organisations are very difficult to access [inaudible – 00:42:02]. The cultural competency of their frontline staff could also be issue for access. The location of where these organisations are located. Is it accessible by train or bus, you know all those things, are also you know, that also either creates or enables, creates barrier or enables them to access you know. The complexity is baffling because if we look into health system itself, interpreters have always been an issue for access. So what that has done is young people, as soon as they come, they take the parents role and parent take the kids role. And that has, we've seen a massive family breakdown because of that. Because the parents are relying heavily on kids. Medical centres, again coming back to cultural competency, not understanding where they're coming from. The PTSD triggers attached to that. Those are all, you know those are enablers or that create barrier.

Interviewer: Yes. Great, thank you. So the next set of questions are around…

Respondent: Sorry can I add quickly…

Interviewer: Yes, yes.

Respondent: …something on – and the media perception around refugees and how it was a topic, is always topic around election time. It also creates a very, it stereotypes the whole refugee population. So you know, that actually, not just impact the parent or elderly of the house but also younger generation. Which then have identity crisis and they don't want to be associated with their origin. So yeah. What has happened with the African community is going to happen again if we don't start…

Interviewer: Addressing it.

Respondent: …addressing. You know, having that microscopic understanding of what creating sense of belongingness is.

Interviewer: Oh great. Thank you. Great feedback. So the next questions are around financial literacy and income generation etc. So can you tell us about some of the programs that are available for financial literacy?

Respondent: Yep.

Interviewer: And we've got two other dot points. Income generation and managing money effectively, so yeah.

Respondent: So financial literacy, are we talking about budgeting?

Interviewer: Yes, yes.

Respondent: What are we…

Interviewer: Understanding how to manage money I suppose.

Respondent: Yeah. So mostly around yeah.

Interviewer: Yes.

Respondent: Look it's, the reason I raise that was, in this space, in settlement setting we do have like I mentioned before it's a mixed basket. We do have doctors, we do have people illiterate in the language. So I'm going to exclude people illiterate in the language and focus more on the people who are educated in the language that sort of understands the concept. Sorry can you repeat the question?

Interviewer: So the first part's just about telling us about the programs that are available, and then we'll talk about some of the culturally specific stuff.

Respondent: So the program that are available, I can talk about the financial counselling grant. That was funded through, I think that's the, that's federal funding I think, and we applied, we were not successful. But then Salvation Army in

Bankstown we have (NAME OF LOCATION) MRC, so they do provide financial counselling. So where people have difficulties understanding money management. How do we manage? That concept itself is very, could be new concept to people. Because what happens is, in that context when newly arrived refugee and migrant comes they're role reverses at times as well, right? Gender role reverse. Before men, only men use that back home wherever they are, with a patriarchal(?) society. Men are considered to be the house, main household you know person and hence look after all this finance. And because their life here, they have you know role division so both of them then get Centrelink and then hence have to manage their budget. And that concept could be, I don't want to stereotype again, but there have been a lot of women struggling. Because they have never done that before. The bank account was always on husband's name and they were, husbands were the provider. They were the housewife, they would run the house and they had a certain budget allocated to the house but now once you're on Centrelink money is being taken left right and centre. All the Family Tax Benefit, everything comes to you as well as a mum, and you would have to manage that. So I think that's where the bit of confusion is. But as soon as they start, and it's a bit confusion at the initial, at the start. But once they understand the concept of Centrelink and what Family Tax Benefit A and B is and what the payments are, then they start their very, they start to manage it well. So they consolidate(?) their budget. I don't remember what is it called. But the services I find in the counselling, they're playing critical role. Especially with what we've seen is people on Centrelink usually take the rent, you know Centrelink usually take the rent away, put it, you know there are some people who do that. And when their accident happens, when there are deaths and when there is sudden family death and when there is, it goes haywire. And it is extremely difficult at that point in time when they don't have support for them to go down to, you know into more debt and not knowing. And with all this cash, what do you call that? Cash collector? You know they give you cash loan? The loan sharks?

Interviewer: Oh yes, yes I know what you mean.

Respondent: They go down to that loan shark and it's extra…

Interviewer: Oh, and then the interest and yeah.

Respondent: And so the roles that these charity plays in providing that sort of financial literacy is very critical. And there are services, not much, we still need a bit of focus in this space, but it is there.

Interviewer: Great. And you've already touched on some of the financial challenges, but can you talk about any culturally specific dynamics? Besides the gender one.

I mean I'm sure that's one, a big one, that impact and challenge the management of financial demands.

Respondent: Sorry, say it again?

Interviewer: So the culturally specific dynamics, like sending money home or dowries or, you've already talked about the gender imbalance, yeah.

Respondent: No I would say like I mentioned earlier as well, in 2015 the visas of class that we had was 202s. 202s is proposal(?) visa. So we basically, a family member from here would have to propose to government saying that I have my sister living in Syria, living in Jordan and she has five family member, and I'm able to, they are able to purchase their ticket and I'm able to provide them accommodation. And all the living costs will be looked after by me, right?

Interviewer: Wow.

Respondent: So one, so that's, that's, for the government it was very smart move. For the community it created a lot of tensions. And so what that did was these newly arrived families came to Australia with a huge debt. As soon as they arrived to Australia the first thing they started asking the service provider was where can I get food vouchers? Where can I get electricity vouchers? Which we've never seen happen before. Before usually at around one year mark they would start asking questions around food vouchers. Here since 2015 the first thing they want to get more is food vouchers. And also the loans they had taken with no interest loans it was increasing heavily. And you've actually brought a really good point of survival guilt. When you're in Australia and you have your cousin back home, and again the Eastern culture of family is not just your, it's not independent, it goes beyond your immediate family to cousins. If we're looking at Africa it goes to your community as a tribe. So there was a lot of money being spent and sent to different countries. But now, especially now is very, very critical time because there is a massive conflict happening in Iraq with what's happening with Iran and Iraq, all those conversation, the protest. People are sending much more money to their relatives than before as well. So the protest has spiked the international sort of concern what is to happen in Iraq. So families are now, again I'm assuming there will be a lot, there will be many families sending money back home. And I've actually seen a woman myself who was actually was really struggling to buy her medicine but was sending $200 to her brother in Lebanon.

Interviewer: Wow.

Respondent: Yeah.

Interviewer: And in terms of these financial counsellors, are they aware of these dynamics…

Respondent: They are.

Interviewer: …and can they actually support people through that?

Respondent: No. Can I say something?

Interviewer: Mm.

Respondent: Financial counselling is very important, but financial counselling cannot address poverty. So this issue that we have here with refugee cohort is not about managing your, most of the time it's not about managing your budget. It is mostly about I am living under the poverty line, what do I do? If you look into Newstart Allowance, it is $300 or $400 a fortnight. So the maximum, a maximum amount that you can get through any payment is aged pension, which is around $723. And imagine paying rent and paying everything and paying whatnot, medicines and specialist visits, and…

Interviewer: No. I know, it's impossible.

Respondent: …it is not about, I think government, if it's about financial literacy, we really need to know is it, are you assuming that people are not budgeting it correctly, or is there another, is it just the tip of the iceberg? Is it because we're living under the poverty line? And there's no way…

Interviewer: That's right.

Respondent: …you know, there is no – so the living cost has increased. I was looking at this, the insight or The Drum conversation around Newstart Allowance. Living costs since 1993 has increased. You know it's not just, it's probably tenfold, twentyfold. But the Newstart Allowance has not even gone up. So really it's not just about managing the budget.

Interviewer: No. Okay thank you.

Respondent: But thank you for bringing that up because that has always been our issue. I mean how do we manage $400 and you have rent of $600, what do we do?

Interviewer: Yeah, that's right.

Respondent: And there's no way, you've actually banged your head and…

Interviewer: It's even hard to live on that weekly, let alone fortnightly.

Respondent: No, exactly yep.

Interviewer: So the next questions are about legal services. So can you tell us about the programs and supports available for your clients with legal issues around identity visas, inviting family members to Australia?

Respondent: Yep. We work very closely with Legal Aid. Now Legal Aid has different arms of it. Legal Aid is focussed on immigration, family. We also work with (NAME OF LOCATION) Legal Centre. We work with Immigration, IARC, Immigration Right and Responsibility Centre. So there are a lot of assistance in that space. However what we lack, I don't know if it's on your question, but I'll explain it now. What we lack is form filling. Form filling has always been an issue. All these people wants to invite their families over. They're not, like I said they might be illiterate in the language, they might be literate in the language, does not know English at all. How do we fill out that form? So what has happened is that that void, that vacuum has created you know, I mean migration agent are taking opportunity and actually doing, you know filling the form not in a correct way and jeopardising the whole process. And then this vulnerable client paying up to 1,300, 1,400 extra dollars to migration agent. And so because Legal Aid would not, does not have capacity to fill out the form. So what they say is fill out the form and we will review it. And Legal Aid, immigration appointment are booked out. It's because we outreach, they outreach from us. And it's always booked, it's always booked out.

Interviewer: Okay, thank you. And for physical violence and other forms of violence or discrimination, what sorts of programs and supports available?

Respondent: Yep so the, in (NAME OF LOCATION) integrated domestic, through integrated domestic violence we provide, through integrated domestic violence program funded by FACS(?). We provide case management. But like I mentioned earlier, it's very difficult for us to manage, you know that sort of push and pull between demand and supply.

Interviewer: And what are the key laws? What do you think the key laws and provisions are that migrants need to learn when they first arrive in that first year?

Respondent: Well I'll say it can be really anything. It could be road rules and safety you know. How do you, you know how do you use garbage bin and all those things. Very, very, orientation to Australia, how do you catch a public transport? How do you speak to your neighbour? How much can you speak to them? What's the Australian way of living? I think those orientation has to, they need more of that. And I was actually told by this young girl yesterday that they need videos. Just don't talk, give us the videos of how to catch a bus. Because the case manager might not be able to take this person to everywhere, but if you have a videos of generally speaking how you need to catch a bus and how you tap the Opal card or where you put the money on Opal card, those things. Videos, you know so…

Interviewer: Oh, very practical, yep.

Respondent: Yep. All those things. So really yeah.

Interviewer: And within the first five years? So more of the same?

Respondent: So I would say now, I would say the orientation would have to happen within that one year, six month to one year period. After one year they're settled down, then it's mostly around employment, English and training. So mostly around that. And also a bit of social support, excursion, creation, you know that sort of thing happens.

Interviewer: Great. In your opinion what is the level of awareness of migrants to accessing key legal services when they need them?

Respondent: Yes I would say it's pretty good because our experience have been it's word of mouth. Anybody knows Legal Aid and where they outreach they go there.

Interviewer: Great. And what kind of challenges do clients of your service face when accessing these legal services?

Respondent: Again, now you know, it's booked out, form filling, procedures and protocols that the organisation have.

Interviewer: Great, okay. Now the next lot of questions are around movement of clients from one place to another. So what do you think some of the key reasons for the movement of your clients from one place to another or suburb to suburb?

Respondent: Social support, religious landmarks, church institution. All those things. Amenities. You know access to transport, all that. And cultural shops and restaurants and grocery shops and the potential of getting job through those local businesses.

Interviewer: Yeah, great. And are you seeing any trends in mobility?

Respondent: Yep. A lot.

Interviewer: Okay. Especially in the early years after migration or after several years or…

Respondent: Yep so government focusses on regional settlement and we don't see that working without procrastinating. Because at the end of the day your right to movement is your constitutional right and they can take them to Wagga or Armidale, but if they have support here…

Interviewer: They can't make them stay here.

Respondent: …they're going to come back. So we've seen that a lot happening. And yeah, until and unless, that's what I've been saying to them, until and unless the whole community leader organisation, they build church there, it's not going to happen.

Interviewer: And just, it's not a question but it just made me think, if people are settled in Wagga and they don't want to stay there, are they…

Respondent: They're coming back.

Interviewer: Do they have to stay there…

Respondent: No.

Interviewer: …for a set amount of time or anything? No?

Respondent: No. They come, well we've seen, we've had that experience as well. So there were families, a lot of families from Wagga travelled to Sydney, particularly to (NAME OF LOCATION) and (NAME OF LOCATION) because they had jobs. Not (NAME OF LOCATION) for jobs, (NAME OF LOCATION) for social support, (NAME OF LOCATION) for a better jobs.

Interviewer: Okay. Great. Okay so the next set of questions are around education and literacy programs. So can you tell us about some of the services migrants are using in terms of school education for their children, adult literacy programs or any other education or literacy program?

Respondent: So they use playgroup a lot. Transition to school groups. IECs, Intensive English Support, intensive in the centres. They also use the (NAME OF LOCATION) Senior College, because after a certain age you can't go to school or IEC so they're referred to (NAME OF LOCATION) Senior College and we only have one college there. So they do that and TAFE with AMEP, MTC, oh what is MTC, Migrant Training Solution, Navitas. Basically yeah, those are the avenue the access.

Interviewer: And do you yourself have any programs here?

Respondent: And(Service name), sorry (Service name).

Interviewer: Do you have any…

Respondent: Yeah.

Interviewer: Yes?

Respondent: Yeah. So we have education, employment and training and we work with I think more than 14, 15 registered training organisations to provide the in-house courses tailored.

Interviewer: I saw your short courses list downstairs.

Respondent: Yeah. Tailored for the community. And we also under the Smart and Skilled we try and get the language support. So yeah we do a lot of that.

Interviewer: Great. And what do you think some of the key issues or barriers are for children of your clients to accessing school or university education?

Respondent: Sorry when I think of these children I can't just, I can only think of this brilliant people that we work with, young people. But in general, look we do have a bit of health issues. The impact of parents having Post-Traumatic Stress Disorder, you know incontinence issues, parenting differences, eight years old still sleeping with parents. And not understanding that it's not appropriate in Australian way. So I think those, we've seen that a bit. We haven't, I really, oh to be honest with you I haven't seen identity crisis. I haven't come across that but I'm sure that's there as well. Yeah.

Interviewer: No that's great. Can you tell us about any special packages or subsidies provided to support educational opportunities?

Respondent: Look I think that would be a really good question for TAFE, but I think as far as I know they have under the Smart and Skilled courses provided, delivered by TAFE, there is RTOs pays, sorry don't quote me on this, but I think they do have certain concession and things like that, but I'm not very sure.

Interviewer: Are you able to outline the kind of employment opportunities that you're aware of that are offered to migrants' children when they finish school or university education?

Respondent: Look I think migrant children are, migrant and refugees children grow up, I've seen the majority of them grow up to be Australian. So wherever Australian kids are working, that's the same avenues for them. I would differentiate(?) parents because of the language skill, but kids that goes to school and Uni here are Australian. And I don't think they're struggling else than probably, I don't know, identity crisis. I have no idea but I'm just assuming that could be the lens we could use but yeah else than that, employment wise it's very similar.

Interviewer: What about employment opportunities for migrants more generally?

Respondent: Employment opportunities for adult migrants is challenging to be honest with you because of language barriers, not having local experience, overseas qualification not being realised. But what we've slowly seen happening, and even in global context if you look into the Starbucks, they've actually made commitment of taking, by 2022 they want to take around 50,000 refugees in Europe in Starbucks so…

Interviewer: Wow.

Respondent: …we've actually, 50 or 20, but I think we've actually seen this happen. Giant companies waking up to the strength of refugees. But now in Australia we haven't, we are yet to see that sort of you know. But still there are ad hoc businesses working, you know business owner from similar background, and clubs that understands their plight and give them that sort of go. One thing that I would like to put into employment space is if department looks at

adding up the volunteering experience of refugees and migrants as the job, so it's jobactive under the jobactive model, then it could immensely benefit the whole community. Because they won't just be applying for jobs because they have to, otherwise their Centrelink income would be cut off. But if they are volunteering actively and getting Centrelink payment based on that, they're actually building their local experience. And they're, as a volunteer they would also have accountability that they need to meet. You know so I think yeah.

Interviewer: That's great. That's great. So we're nearly there. Overall, what do you think are the key challenges migrants you work with face while adjusting to the Australian cultural…

Respondent: Look I would say again if I, socioeconomic integration and that has to have balanced way of moving forward. That's, and education, you know in terms of overseas qualification recognition is always there, domestic violence, and navigating the system. Yep.

Interviewer: Okay, thanks. And finally would you like to see, what would you like to see as possible solutions to helping or supporting migrants to adjust well to life in Australia?

Respondent: So I think I would start with overseas qualification, and pathways, a flexible approach to that. I would also move toward more Australian community, community at large coming together and assisting refugees and not just being extremely dependent on government funding. And I would also like to mention domestic violence. More case management services, holistic service provision in domestic violence space. I would also say employment, you know including volunteering as a jobactive criteria could really assist people in building their confidence. And also I think as a larger community, as Australian community being more open and receptive. And I think, yeah I think that's very important.

Interviewer: That's great. Fantastic. What an amazing amount – is there anything else you'd like to add that you think you haven't covered.

Respondent: No. I think [inaudible – 01:09:05].

Interviewer: I know you've covered a lot of ground there.

Respondent: Yes.

Interviewer: Some fantastic input, thank you so much.

Respondent: Yeah. No I feel like I've spoken way too much.

Interviewer: No, not at all. Oh it's 20 past 3:00 so you better get cracking.

Respondent: I know, that's alright. Yep.

Interviewer: So the interview ends at 3:18. Thank you.

Respondent: Thank you very much.

Interviewer: Thank you.

Respondent: I hope your research goes well.
