# Supplementary material for: The importance of information acquisition to settlement services literacy for humanitarian migrants in Australia
Source: PLoS One. 2023 Jan 6;18(1):e0280041. doi: 10.1371/journal.pone.0280041 (PMC9821785; doi:10.1371/journal.pone.0280041)
Supplement: S1 Data — (ZIP) [file pone.0280041.s003.zip › SP_04_Victoria.pdf]

Interviewer: Alright, so (SERVICE NAME). And (NAME) with (NAME). Excellent, thanks (NAME). So, the first set of questions are about the services that are provided by (SERVICE NAME) just generally; so, it doesn't have to be specific programs, but generally what services are provided for health or education or legal or social aspects.

Respondent: Yep. So, we offer –

Interviewer: For new migrants, sorry.

Respondent: Yep. So, I guess the … we pretty much offer services in all areas. So, that would include education; basically, we'd start with case management. So, case management services. Case management can lead in any direction, really. And we would … we would not say no to assisting a client in any area.

Interviewer: Yep, so that case management can be tailored to include any support?

Respondent: As long as the client is eligible for particular services, if they've been here within the five years, then we could concentrate on any area of the settlement, which could be education, employment, health, mental health, knowing how to cross the road, you know, it could be literally anything. But we would like to focus on helping them become … learn the systems of Australia and helping them to become more self-sufficient in Australia.

Interviewer: Yep, brilliant. And so, are there any organisations that you collaborate with or work with in providing services?

Respondent: Yeah, lots of organisations; we partner with organisations to deliver programs, specific programs.

Interviewer: What would they be?

Respondent: So, a council for example, City of (NAME OF LOCATION). Other settlement services such as MRC (NAME OF LOCATION). Occasionally Arabic Welfare. We partner with Centrelink to deliver information sessions. We had one today, here, for newly arrived HSP recipients, so people that have been here less than six months. Partner with Uniting Care, I could list heaps.

Interviewer: So, government, other service providers.

Respondent: Local government …

Interviewer: And then, obviously, religious organisations like Uniting Care as well?

Respondent: Yeah, yeah.

Interviewer: Yeah.

Respondent: They wouldn't see themselves as a religious organisation, not-for-profits, you would say, other not-for-profits. They do – they are church-based, but the people that work there aren't really, you know.

Interviewer: Yeah. Uniting is, I guess, I'm not … anyway. That's another story.

Respondent: Yeah.

Interviewer: Excellent. Is there anyone that you don't work with on anything, for any particular reasons?

Respondent: Like do we have a black book of people that we don't work with?

Interviewer: Perhaps if, there could be some conflicts with faith-based organisations or anything?

Respondent: No, no. We can't do our work without partnerships, and we are open to any and all partnerships, really. I don't think there's anyone that we would particularly say no to. Even if a bank approached us, we would probably … providing that we were helping our clients. We would take the opportunity.

Interviewer: Excellent. And are there any services that you're aware of that are needed but not available for new migrants?

Respondent: Very good question. Services, or …?

Interviewer: You can say programs, if you can't think of, say, general service, it could also be what kind of programs are needed but are not there?

Respondent: There's probably quite a few gaps. I would say there's more need for more English, opportunities for English, practice English outside of a classroom setting. So, there's probably not enough casual English classes or sessions. There's a massive gap, and I think I'm talking generally here, for young people below the age of 16. There's not a lot of programs for them, because I think it's expected that because they're engaging in school and primary school, that they're getting that there, which often isn't the case. We always get questions from clients about what can my kids do? What can I do with my kids, what's available for them? We have to say, nothing; because we usually work with 16 and up, and above, for after-school programs and things like that. So, there's not much for actual children, other than young people, which is interesting. There is for toddlers and smaller children, like there's play groups and things like that. But it's for the middle years and the younger primary …

Interviewer: Yeah, it's interesting from some of these other providers as well, I'm getting more of a picture that schools are, in a way, providing services as well.

Respondent: Yeah, I think it's almost expected that they are. And if they're not, then there's a gap there. I don't think schools are educated enough about specifically newly arrived and refugees, about how to work with them, and how to get

them engaged. There could be distrust at times, from the families or parents, that they're to go to school, not to do extra-curricular stuff. So, I think that's about the literacy about what logistically probably from the school's perspective, that they need to become more literate about settlement and how to work with them and what services should be offered through the schools.

Interviewer: So, are any services over-utilised at all do you think?

Respondent: Over-utilised, maybe … I can tell you what's in high demand, and that would be migration services, people wanting to bring family over. And if you go … if you want to talk about over-utilised people if they get a refusal, they will keep trying and trying and trying again. So, it's … (NAME), when I was in here earlier, I spoke about service hopping. If, let's say we have a limit of doing three applications, if three of them get refused then we won't see a client again. Sorry, that's three refusals. Do you understand that little bit about the migration space?

Interviewer: Not a heap.

Respondent: So, migration agents who do applications for people to bring family members over as proposals.

Interviewer: Yeah.

Respondent: Yeah. So, they can be refused by the Department of Immigration or accepted, depending on the situation. So, three refusals and we'll stop, but they could go to another service and keep trying, potentially; which would be a waste of resources because of time, because we know it's going to get refused. We don't know if they're attending other services because we don't see the databases [inaudible – muffled 06:54]. So, that could be over-utilised, I guess, in a way.

Interviewer: Yeah.

Respondent: Mainstream services, I don't think so. Most things are under-utilised, I would say.

Interviewer: So, what things are under-utilised?

Respondent: I think access to council, council like … people don't access council services.

Interviewer: Counsellors or council services?

Respondent: No, council services.

Interviewer: Yeah.

Respondent: The council, particularly in (NAME OF LOCATION), struggle to get newly arrived people engaging with their services.

Interviewer: What kind of services do they have?

Respondent: Oh, they deliver information sessions. They've got youth services; they have aged care services. All sort of things.

Interviewer: Specifically targeted for new migrants or just for any main council …

Respondent: Mainstream, mainstream. Mainstream services, any one in council. Being in (NAME OF LOCATION), (NAME OF LOCATION) Council understand that if you work at (NAME OF LOCATION) Council the constituency is more than likely going to be from a diverse background. So, they don't specifically target how programs are targeted at newly arrived; they just expect that because the cohort of people living in this area is newly arrived –

Interviewer: Yeah.

Respondent: Of a different background, and also as it should be, for them really. And they struggle to get a lot of people involved. So, they do information sessions regularly, with probably topics like road safety.

Interviewer: Would that be about orientation?

Respondent: No, no. To different topics. Might get guest speakers to come in, they might do a road safety event or things like that, and they would struggle to get newly arrived people there. Other services might be mental health services, I think as well, that would be under-utilised; the stigma that's involved in mental health. So, people would be reluctant. And I think to an extent, youth services as well. Just because there's not a great understanding for culturally about what a youth worker is. How that's … it's a specific targeted approach to working with young people, and it's a foreign concept for a lot of people.

Interviewer: So, they wouldn't see the value in …?

Respondent: No. No, not until some … one of their … a young person who's engaged and they might see the benefits then. But the trick is getting them engaged.

Interviewer: Alright. And so, thinking again, about services here from (SERVICE NAME), do you do any kind … do you do any measurement of effectiveness, and if so, how?

Respondent: Oh, evaluations?

Interviewer: Yeah.

Respondent: We try to do. We're not great at it, and we're still trying to rejig how we do evaluations. But we … I guess every … we do a lot of group activities. So, every six weeks or so, we try to do a bit of a questionnaire type of thing. What are we currently doing? In terms of one-on-one work, we like to get clients' feedback, obviously. And we try to get clients to rate the service.

Interviewer: Yeah, OK.

Respondent: Through a reporting tool. So, it's, what they call a scoring. So DSS like us to do scoring with clients based on their satisfaction or … of the service. Or, their circumstances, have they changed? We try to measure from the last time they were here how those circumstances might have gotten better based on different criteria. Or their goals, have they reached their goals?

Interviewer: So, is there some requirement from the –

Respondent: DSS.

Interviewer: Yeah, for Humanitarian Services Program?

Respondent: That would be more for – I think it's for Humanitarian Settlement Program as well as SETS which is … you're familiar with the acronyms?

Interviewer: Yeah.

Respondent: So, yeah, there's a requirement to score. I don't think we … it's very subjective. And it's … there's not a requirement that we do it with the client.

Interviewer: Yep.

Respondent: Although we encourage the staff to do it with the client and help them make them score themselves, rather than us doing it subjectively on behalf of the client. So, we can move towards that. And we … we're meant to score groups as well, as I said, we do that. Not every session, but every say, six weeks or so. And we also have our own internal evaluation tools which we try to use, just basic questionnaire-type things. Occasionally we do focus groups, but not very often.

Interviewer: I imagine there's a tension in terms of funding between evaluating and providing services?

Respondent: Yeah, of course, yeah, yeah.

Interviewer: The next questions are related to how migrants adjust to Australian culture and society and what kinds of issues they may … challenges they may face. So, what's –

Respondent: That's a big, meaty question.

Interviewer: What's your understanding of the migrants you specifically work with, of how they understand Australian culture and society? Do they understand Australian culture and society?

Respondent: It takes a lot of time to get to a point where you could say that they have a, even a general understanding. So, when I first arrived, I would say no, there is very little understanding of … of what Australia is, really, yeah. Systems,

lifestyle, all that stuff. And there's a … in this area, and probably in the west where we work as well, there's probably … because a lot of clients, a lot of people we see have already family members here. Then there's less … that they come, they have their community, they're introduced to their own community, initially, with their family and friends. So, they're not forced to get out of that space, it's still in that silo of their own culture. It's not until they start enrolling in English language classes that they really get out. And even still, when they're in the AMEP classes, the English classes, they're still with people of their own culture and background and language groups. So, they tend not to … even try to practice English or learn about Australia, or very few opportunities to even meet Australians, non-newly arrived people, to learn about how we might do things differently or our culture.

Interviewer: Yeah.

Respondent: Even food.

Interviewer: So, you've gone, I guess the challenges there, one of them being –

Respondent: But what we're trying to do through some of our programs is give more of an opportunity for people to learn specifically about Australia and … So, one of our youth programs which runs on a Tuesday is … our focus is really about practising English and teaching the young people about Australia. So, we might have an Australian sports themed day where they learn about AFL and cricket. We might do Australian food a different day and they eat kangaroo or talk about healthy eating in Australia. Different themes around Australia which is … which they're not exposed to, or we've found they haven't been exposed to at all.

Interviewer: Unless they're moving out of their cultural group.

Respondent: Even if they are moving out of the … it depends on who they've become friends with, I guess, outside of … it's rare that they are becoming friends or hanging out with people outside of the …

Interviewer: This is for children or for adults?

Respondent: Both, I would say.

Interviewer: Both.

Respondent: Yeah.

Interviewer: Yeah, right.

Respondent: Yeah.

Interviewer: And so are there other kinds of … so, challenges, you mentioned language, obviously this seems to be an issue with people not moving out of their

cultural groups. Not that they have to, but not making other connections aside from their cultural groups.

Respondent: Yeah, or they do that very slowly.

Interviewer: Yeah, are there other challenges alongside that?

Respondent: Challenges in terms of settlement?

Interviewer: In terms of understanding Australian culture and society?

Respondent: Yes. Moving out of the geographical area as well. We've found that a lot of people, even after the first six months, they have never been to the beach, or never gone past … outside of (NAME OF LOCATION). They're still stuck geographically within this area because they either haven't been taught in orientation properly about how to use public transport, or they're too fearful to leave, because it's their comfort zone. Or they just … I don't know.

Interviewer: Who would provide orientation services?

Respondent: That would be the AMES and the HSP program. They initially have orientation in the first bit of HSP.

Interviewer: So, what about for voluntary migrants, because they wouldn't do the HSP?

Respondent: No, they wouldn't, that's a good question. Voluntary migrants will … voluntary. So, people who are proposed by family often wouldn't have orientation. We don't work with, say, economic migrants so much, the people who would come here who aren't refugees. Refugees are really the bread and butter of (SERVICE NAME). So, it would be expected that those who have links here already would rely on their family members to show them around and teach them things. And we wouldn't necessarily know what their level of expertise in those areas is. So, we don't know what information they're actually getting. At least if they are going through the orientation services, we know what they're being told.

Interviewer: Yeah, yeah. And so, in terms of practicing their own culture, are there … do you see there's opportunities provided to migrants for that, and what might they be?

Respondent: Definitely. Oh, there's lots of cultural events and festivals throughout the year. They could be specific to specific cultures or it would be a big open generic one such as Refugee Week, we have a big event that celebrates all cultures and celebrates newly arrived people in general. I think people are encouraged as a part of settlement, it's one of the nine pillars, really, the settlement counsel is to maintain links to their own culture. Encourage them to freely practice.

Interviewer: Isn't there encourage … I mean …

Respondent: In the wider community.

Interviewer: What about the wider community?

Respondent: I know … well, I don't know, to be honest. I think, here, because there's so many people that are of newly arrived background that it is for the most part. Councils certainly promote it and encourage it, and I think, understand it. I don't see – I haven't seen in my years of working at (SERVICE NAME) – I haven't seen any barriers to that, to be honest.

Interviewer: Yeah, any barriers to practicing culture?

Respondent: Yeah. And I haven't seen a lot, for example, I haven't seen racism – I know it's out there – but [inaudible – muffled 18:21] individuals or groups who would want to stop that from happening.

Interviewer: Yeah.

Respondent: Certainly not in the open. I know it's out there, but I haven't seen it, personally.

Interviewer: So, these next questions relate to a migrant's sense of belonging and inclusion; what programs or services does (SERVICE NAME) provide which help to create or enhance sense of belonging and inclusion in Australia?

Respondent: All our programs have an element of that.

Interviewer: Yeah.

Respondent: Because we're constantly encouraging people to have a positive settlement experience which is about belonging, really. So, to learn about how systems work and how to access services. If we can encourage that, then obviously they're getting, hopefully getting a sense that we want them to be able to belong and access the services. Does that makes sense?

Interviewer: Yeah, yeah it does. So, you would do it through whatever you're doing, whether it be case work, whether it be running information sessions with partners?

Respondent: Yeah, yeah. Helping people feel included, both within (SERVICE NAME) and in the wider community. We probably don't do enough advocacy to the wider community on settlement and settlement issues. We do where we can. But I think our CEO wants to have more of an advocacy, I guess, he wants to become more active in that area. And if we can help educate others about settlement, which would make people feel more welcome. Then that would be fantastic. But I think what we do for clients at the face value, like I said, everything we do has some sort of element of that.

Interviewer: And so, who are the key people that your clients might contact for social and emotional support, do you think?

Respondent: So, first and foremost, it would their immediate family, I would say. It would also depend on what level of emotional support they would need, I think, what type of support they would ask for, who they would go to. But certainly, in most cases I would say family unless it was quite serious. Unless it were – we're talking mental health issues where there would be more stigma and they wouldn't want family members to know that, therefore it would get out into the community and then they would feel a bit isolated or they could be shunned from community or … there's lots of issues associated with that. So, they might come to their case worker – I would hope, their case manager – who should be encouraging them and asking them every time that they see them, how are they going, if you need assistance, come and see me. If they don't have a case manager, then hopefully if they have gone through some sort of initial program like HSP, they would know to talk to their GP.

Interviewer: Yep.

Respondent: I don't know if that would happen all the time, but what I do know, that most of our clients would be connected with a GP who is of the same cultural background and of the same language group, so they would feel comfortable talking to them.

Interviewer: Who connects them in doing that?

Respondent: Initially it would the HSP case manager.

Interviewer: Yeah, OK.

Respondent: Then, if they don't have a case manager, hopefully it would be a family member who's been here longer who would connect them to their family GP. Otherwise they could come – up to that five-year period – they could still come and speak to somebody and be case managed through the SETS program and they could … yeah, the case worker could assist them. Whether they would know to do that or not is a burning question, which is all about the literacy. But obviously the referral pathways are many and varied. So, you know, we have Foundation House for trauma and torture; we have lots of mental health service providers. So, it would all depend on, I guess, the client's level of knowledge about their own … about what kind of support they would need or want, and where they would go to, first and foremost, yeah, it would be up to them and where they … what their knowledge is like.

Interviewer: Yep. Yeah, right.

Respondent: It's complex, it's a really complex one.

Interviewer: And so, in terms of, you're speaking GPs, so this next question's about programs that (SERVICE NAME) might have that respond to, or help sorry, improving health or wellbeing of migrants. Are there any programs at (SERVICE NAME) that do that?

Respondent: Yes. We're actually working on a healthy eating program for young people at the moment, in partnership with (SERVICE NAME) Health which is …(SERVICE NAME) … used to be … something (SERVICE NAME) Health, has merged recently. (SERVICE NAME) Health. Obviously in HSP there's a big, a very big focus on health there. Especially, even pre-arrival. We need to know any serious health issues and some people get straight off the plane and go straight to hospital if we know that there's illnesses or serious illnesses. So, health is a priority in HSP.

Interviewer: Who do you – just out of interest – who do you connect with outside of Australia?

Respondent: It's usually – it's a good question, I'm not an expert in HSP, because I've never actually worked in it – but I believe that there's not a lot of contact outside of Australia. It's most with the proposer here, the connection here who has the contacts with the client overseas. So, it usually comes, I believe, through second hand knowledge from the proposer.

Interviewer: Who would the proposer be?

Respondent: But they do the health checks before they come, so the service provider, we get referred clients by AMES, so they [would] know from the health checks and things, the pre-screening that's done overseas by … I'm not sure who does it, whoever the international health agency is, they would have the records. They would send it on to AMES, who would then send it on to us who are giving us the clients. Then we would contact the proposer, I would imagine, and talk to them, who's usually a cousin, or a brother, or a sister, or a husband that could give us more information to us exactly how the client's going. Even at the airport, on the other end, did they make it OK to the airport? How are they going on the plane? That kind of stuff. And things could be put in place for when they arrive.

Interviewer: Great.

Respondent: Yeah, but we have good connections with the health services, I think specifically community health and working with clients. And population health teams in the different community health areas who can deliver programs for clients, we can work in partnership with.

Interviewer: Excellent. And so, do you see any enablers or any barriers for new migrants in accessing health services?

Respondent: Barriers would be, gosh, advertising and community in general [out there]. It's amazing how many young people, newly arrived, just live on fast food. It's amazing. It's a huge issue, actually. There's a healthy drinks alliance that's started here as well, which is about teaching newly arrived people about not having the sugary fizzy drinks. So, there's a big push on healthy eating and minimising the risk factors for diabetes, heart disease, and all that sort of stuff. We would be the biggest enablers for getting them access to the health services.

Interviewer: Yep.

Respondent: I would say the settlement services would be. Probably even some good supports would even be the schools and [inaudible – over talking 25:50].

Interviewer: OK.

Respondent: Who see them every day, that can incorporate health stuff into their curriculum which they do occasionally. Barriers would be their own, probably inside and education about health from back home, which is often not a thing. They go to the doctor if they're feeling sick, and that's kind of it. I don't think there's a lot of …

Interviewer: So, there's not preventative health education?

Respondent: Yeah, there isn't. From my understanding, I don't think there's a lot of preventative stuff, it's very reactive. And that's just based on talking to people and understanding what their knowledge is when they arrive, especially the young people, they're not aware of a lot of things. Sexual health, for example, is not a thing, very unaware of a lot of things. A lot of basic things that we're taught at school. So, there's actually a bit of a gap, those who arrive, those who have … after school age, and they arrive here; those who are lucky enough to get into high school and primary school learn that stuff at school, which is great. But those who are 17, 18, miss that. And they might go straight to uni, or once their English is good, they might start working and they've missed that crucial education about health.

Interviewer: Yeah, definitely.

Respondent: Yeah, so there's a gap there.

Interviewer: Alright, so the next questions are about any program (SERVICE NAME) has that helped to enhance new migrants' financial literacy or ability to manage money or income generation.

Respondent: Yep. Money Matters.

Interviewer: Money Matters is a –

Respondent: If you've heard of that program? We don't deliver it at the moment, but I know it exists.

Interviewer: Have you delivered it before?

Respondent: We have delivered it before, yes.

Interviewer: Why would you … because (NAME) also mentioned … no, no, (NAME) did mention about financial, and she said, oh we do do programs but not now. Is that to do with funding, or …?

Respondent: No, I think what happened was you have to put staff through the training which we have done … it's a time consuming thing and then the staff who probably have done that training in the past have left, and we haven't put more staff through the training. I think that's what happens, the knowledge leaves when staff leave. What we haven't done is put everybody through the training, and then we can ensure the sustainability in the programs. But, we probably … yeah, it's a good question, we should keep doing that. What we would normally do is refer out to financial counsellors, things like that, if people run into trouble. We do help through case management. Helping people apply for utility relief grants and things like that.

Interviewer: Yeah.

Respondent: But we don't go into great depth in educating people about how to manage their money.

Interviewer: Yeah, alright. So obviously … but you are still providing advice through [inaudible – over talking 30:06] case work?

Respondent: Yes.

Interviewer: And so, what kind of challenges are you seeing clients facing, financial challenges, sorry.

Respondent: Bills is the big one. We're always seeing clients come in who can't pay their bills or are struggling to pay bills.

Interviewer: Why's that?

Respondent: I think they just had no idea how expensive things can be. Like electricity and gas. Like, they just had no idea. And based on, for example, in the Middle East, based on my understanding, electricity is cheap as chips.

Interviewer: Yeah, this has been common …

Respondent: Yes. From stories I've heard, is in summer people will go away for three months and leave the air conditioning on in their home while they're away for three months. And then come back and they can still pay their electricity bill, it's not an issue. They come here, it's a huge difference, huge difference. So, I think their complacency from overseas has come with them here, and they're like … it's just a shock. It might go on, have a family of six kids, winter time, they've got a heater in every room, and suddenly their bill is $1,000 for a month or for three months, and it's like, how did that happen? So, it's about educating them on usage. For even myself, and I'm sure you … bills are high, right. It's not easy. But if you're unaware that they're going to be that high, then it's an issue.

Interviewer: Yeah. And are there other challenges, financial challenges? And is employment an issue, for instance?

Respondent: Employment's an issue, yes. I mean, it's the biggest issue, probably, for many reasons. I think … look, everybody wants to be employed to have that extra bit of … I mean, to be able to live the life that they want to live. Centrelink obviously isn't enough for many people, but you can get by on Centrelink, just, in some cases. But I don't think that people have those budgeting skills to know how to get by on that amount of money, which is fair enough, because it takes time to learn. I mean, it is … it's a small amount. So, to learn how to budget to that extreme, takes time. So, yes, there are significant struggles to bring up a family on Centrelink.

Interviewer: Are there any kind of cultural dynamics that might raise financial challenges in terms of who's in charge of household budgets, sending money back home. Anything to do with specifically with being a new migrant that could culturally make finances hard?

Respondent: Yeah, I think that lifestyle and wanting to maintain the same lifestyle that they had overseas, here, can have a big effect. And coming to terms with the fact that maybe we can't. Maybe you have to cut down on things, maybe you can't buy that nice new car or fur coat or whatever it is. So, that's a big challenge for a lot of people. Because we are talking about professionals, a lot of people in this area, specifically, who are doctors, engineers in their own country, middle to high class in Syria or Iraq. And now finding themselves … they're not anymore, they're refugees living in Australia on Centrelink. It's hard to come to terms with. I think that's the biggest thing, I think.

And they are learning, maybe I can't send that hundred dollars a week home to my family because I need to feed the kids. Coming to terms with that is very difficult because they want to be able to send that money home. And then having the backlash from the family at home, still in Iraq or Syria, saying you said once you got to Australia, you'd send the money. And then they have to tell them, I can't, it's actually harder than I thought. So, there's a lot of challenges, a lot of complex challenges.

You know, kids going to school, there's a lot of unforeseen costs. You have to buy books for the kids, you have to … lots of stuff they weren't prepared for.

Interviewer: Definitely, school uniforms.

Respondent: Yeah, all that stuff.

Interviewer: Growing up, that was an expense, they are actually quite expensive.

Respondent: Yeah. Really expensive. And some of the schools are great, and they'll give second hand books and second hand uniforms and things like that. But then there's a bit of stigma about accepting second hand stuff. So, a lot of people won't or don't want to accept second hand uniforms. So, there's that cultural thing as well, stigma about that. So, it all again, comes down to expectations and managing that.

Interviewer: So, is that part of that … I imagine that might be part of the case work or the services provided here about managing expectations?

Respondent: Yeah, yeah, definitely. I once worked with a client when I was a case manager, an asylum seeker client … who … we had to find them housing because they were leaving community detention. And we had six weeks to find him a house, a private rental accommodation. And it was a massive struggle, and managed to find this one family two places, but they refused them because they had a shower over the bath. That was the reason. Perfectly affordable house, actually quite nice houses, but nope, can't do that, can't have a shower over the bath because in their country you have to have a shower with shower [inaudible – muffled 35:59], yeah.

Interviewer: Yeah, right.

Respondent: It's just interesting.

Interviewer: That is. It makes things tough.

Respondent: It does.

Interviewer: But I mean, it's a tough … the whole thing is tough.

Respondent: It's very tough. But in a way, they're choosing to make their life harder for themselves because of that barrier that they've put in front of themselves and wanting that … the luxury stuff.

Interviewer: Yeah. So, the next questions are about any programs that (SERVICE NAME) might offer for clients when they face legal challenges. So, do you have any programs that support …?

Respondent: We don't have specific programs that (SERVICE NAME) runs, apart from the case worker referrals. But we have (NAME OF LOCATION) Community Legal Centre who come once a fortnight, I think it used to be once a week, now it's once a fortnight. And they actually run … have appointments outside of our office here and see clients.

Interviewer: OK. Yep.

Respondent: I'm sure (NAME) will probably mention.

Interviewer: Yeah. Mentioned that you, kind of … well you refer, but you … it's not just referring, it's kind of liaising almost.

Respondent: Kind of, yeah. Yeah, it's a strange arrangement. But, yeah, they see clients out of our office here. We … yeah, we basically just facilitate the appointments and them seeing a legal person.

Interviewer: OK. And is that for all things, so, in terms of … is that to do with visa issues, inviting family members …

Respondent: No, visa issues, we have our own internal migration agent, who work for (SERVICE NAME). So, if you classify that as legal, then yes, we do offer legal services in terms of migration. They're not lawyers, but they are …

Interviewer: Migration agents, yeah.

Respondent: Migration agents, yeah. So, yes, we do do that, providing for specific services. So, we don't offer all migration services. So, we wouldn't help somebody come over as a student or as a visitor, who want to do visitor visas, those sorts of things. We'll do very specific things in terms of migration.

Interviewer: Which is?

Respondent: Which is proposing, helping people propose family members to come over on humanitarian … as refugees. So, we only do refugee visas, basically. And there's two other funded programs, one other funded program, IAAAS, which is helping people do protection visas.

Interviewer: IAAAS, did you say?

Respondent: IAAAS.

Interviewer: Oh yeah, that was mentioned.

Respondent: So, Immigration Application Assistance Service. But we have to be referred to clients. You can't … not anyone can just knock on the door and say, I need help. That is assisting people with protection visas who are seeking asylum.

Interviewer: And so, in terms of anything that happens in the community or if there's domestic violence issues, or discrimination; that's for the referral?

Respondent: Yeah. We have family services who can assist with people experiencing family violence, but we wouldn't offer legal help, for instance.

Interviewer: And so, in your opinion what level of awareness do new migrants have in accessing legal services?

Respondent: We do our best. Again, that comes down to orientation. Their initial understanding comes down to orientation and what's explained to them about the legal system.

Interviewer: Yeah.

Respondent: We have occasionally done information sessions ourselves about accessing the legal system. And we've had people like the community legal centres come and deliver information. Yeah, it's interesting with … I've done various programs with the police as well.

Interviewer: Uh-huh.

Respondent: We do partner with Victoria Police a little bit, especially with young people. And they can talk about the law, and police, and what not to do, and what to do. Even have come on some of our youth camps with us, the youth liaison officers. Which is great, because they'd run activities with the young people, and they get to ask questions about the police, and the law, and things like that. So, yeah, we do, I guess, some stuff.

Interviewer: Yeah. So, you run youth camps?

Respondent: We have done that, yeah, we do. We do, we try to do at least one a year. Or two a year. One for the (NAME OF LOCATION) crew and one for the (NAME OF LOCATION) crew, yeah.

Interviewer: And what are some of the challenges in terms … that you see in accessing legal services for new migrants?

Respondent: It depends. So, sometimes there are things that a newly arrived person might need assistance with, but it's not … it doesn't fit within Victoria Legal Aid, or the free legal advice.

Interviewer: Like Community Legal Centres?

Respondent: Yeah, Community Legal wouldn't touch some things. They can only work on specific things. So, there might be occasions where a client might need to access a private lawyer for certain things.

Interviewer: Yeah, right.

Respondent: And that would be way too unaffordable for most people.

Interviewer: Yeah, yeah. So, there's not language barriers or anything like that?

Respondent: Yeah, there would be. From what I know, the legal centres always use interpreters.

Interviewer: So, call up TIS or have their own services.

Respondent: Yeah. But we would facilitate the appointments, and then let the legal places know that they need … would need to use an interpreter. So, if they come through us, we would make the appointments for them, and speak on their behalf for the initial … to make the appointment and tell them what the issue would be.

Interviewer: So, that they come through you, right. So, then, could there be people that … people that are not coming through you, maybe not aware?

Respondent: To the legal service?

Interviewer: Yeah.

Respondent: Well, I would expect so, maybe; who would have the language barriers. But I would hope that if they knew to call the legal centres, then they would know how to ask for an interpreter. I would safely assume that.

Interviewer: So, it’s linked back to the orientation?

Respondent: Otherwise, if they didn’t know … yeah, orientation. Or family members telling them, who would hopefully know about community legal. Otherwise they wouldn’t know if they didn’t have a family member here, or they hadn’t accessed settlement services before, they would have no idea, probably.

Interviewer: Does that happen?

Respondent: People falling through the gaps?

Interviewer: Yeah.

Respondent: I would expect so.

Interviewer: Yeah.

Respondent: I’ve heard of occasions where people haven’t accessed.

Interviewer: Would you … do you think it’s kind of rare, or do you think it’s …?

Respondent: I think, no. Actually, what happens, a big concern, actually, is those who have gone through the HSP and orientation period, often aren’t told or often don’t know or have forgotten for whatever reason that they can access future services up to the five-year mark of arrival.

Interviewer: Oh, yeah.

Respondent: So, we find a lot of people drop off after HSP, and don’t access services again.

Interviewer: Yep.

Respondent: And we don’t know why that is. Is it because the HSP provider isn’t telling them, or their literacy about settlement services isn’t sufficient enough to know what services are offered?

Interviewer: Yeah.

Respondent: So, yes, I think there are people falling through the gaps that would need services that aren’t accessing them.

Interviewer: Yeah. And so, these questions relate to how your clients move, are moving or not moving from one place to another. So, what are some of the key reasons why your clients might move around Melbourne, I guess?

Respondent: Oh, physically move around?

Interviewer: Yeah, physically, yeah.

Respondent: It's where the community goes.

Interviewer: Yep. So, where your community goes.

Respondent: Yeah, so … and affordability.

Interviewer: Affordability, as in housing affordability?

Respondent: Yeah, yeah, yeah.

Interviewer: Yeah.

Respondent: But the most of the are … the most of the communities … that's probably not true, but a lot of the communities are already in the more affordable areas.

Interviewer: Yeah.

Respondent: As it is. So, there wouldn't be as much of a need to move around. You find a lot of them … a lot of Arabic-speaking communities come to this area, because that's where their community's established, and it is affordable, and it's a growth area, so there's more affordable housing being built. So, there isn't … I don't think there's a huge number of people moving, specifically Arabic-speaking people, they're not moving out of this area to other areas. I think there is more influx into the area, than out. I would assume, yeah. The west is a bit different.

Interviewer: Yeah.

Respondent: But where there are a lot of people moving in and out because it's a much more diverse community for newly arrived peoples. But they are … I think people are moving further out, and that's obviously where it becomes more affordable. Yeah, so I think affordability is probably the main factor for people to move out. But the community and the demographics of the community would be the reason they come here.

Interviewer: Yeah. And is employment at all a factor?

Respondent: Employment … I think housing is the first … well, I hear people talk about how a lot of people want to live very close to where they work. But they're not finding the jobs. Work is … it's not happening as fast as we would like it to happen for a lot of people. I think housing is the key thing to get right first, then start looking for employment after that. I'm not sure whether the priority for people would be to stay with their community just by work being further away, or to go and live closer to work and be away from their community. I don't know, because that's an individual choice.

Interviewer: Uh-huh.

Respondent: Should employment be an option for them. It’s a good question. To be it shortly, I don’t know.

Interviewer: So, the next questions are about migrants’ access to education, literacy programs; so, does (SERVICE NAME) run any education or literacy programs at all?

Respondent: Not formally. No …

Interviewer: So, informally, then?

Respondent: In everything we do, we try to incorporate some form of English language acquisitions element to all our group programs, even in our case work.

Interviewer: Yep.

Respondent: We encourage people to use English where they can.

Interviewer: And so, the … what are some of those group programs again?

Respondent: So, we have our youth groups, we have some different community development groups, so we have a community garden group that happens once a week. We have some playgroups for parents with young children. What else is there? A parenting in a new culture groups that happen fairly regularly; so, there’s two different type … two different streams of the parenting in a new culture, there’s the fathers’ groups and the mainstream one.

Various other groups will pop up every now and again, just depends on need and what’s happening at the time. We run some employment workshops at the language schools as well.

Interviewer: OK, so you do, kind of, ad hoc – not ad hoc, maybe – but you do specific workshops?

Respondent: Sometimes specific, yeah, yeah. So, the employment stuff which we’ve done at the TAFEs is very specific, specific classes. Yeah, and then sometimes we do ad hoc information sessions which you could say is a group as well. Various youth groups. So, in (NAME OF LOCATION) we’ve had girls’ groups, different focuses. There was a hip-hop group running for a while. Lots of different things. But nothing specifically about education.

Interviewer: Yeah, so you just weave in literacy elements to all of those things?

Respondent: Exactly, yeah.

Interviewer: So, do you see that there’s any key issues or barriers for the children of your clients in accessing school or university?

Respondent: I haven't seen issues with schools.

Interviewer: So, there's no language issues or …?

Respondent: I mean, well …

Interviewer: Interrupted schooling?

Respondent: Interrupted schooling, yes. If their English language isn't up to speed when they first arrive, so they will … most people will go to the English language school first before they transition into mainstream school. So, we would hope that people are ready to go into the mainstream school by the time they do. And they're assessed, and if they need to stay in the language school longer, then they will.

Having said that, there can be discrepancies in what year level they go into, and where they're best suited. So, I'm not sure whether that's always right, what the year level is. Sorry, my train of thought is gone. The English is the main barrier for schooling, for sure, as is university.

Interviewer: Yeah. But then if it's working out at the school, the language school, is it 12 months?

Respondent: Yeah, six to 12 months, depends, yeah. Yes, but some people … depending if they're not assessed properly for their English then they might go straight into mainstream school; they might find themselves floundering a bit and struggling to get up to speed. So, it depends how they're assessed, and who's assessing them, and where they land, I guess. Yeah, could be a bit of an issue. But in terms of actually accessing school, I haven't seen any issues, everybody's been able to access school when they need it, when they want it. There was a period with the extra intake last year or the year before, when we had the extra 13,000 where the language schools, the AMEPs were at full capacity. So, people were actually sitting at home for months doing nothing, waiting to access school. That was a bit of an issue. But that's OK now. That was an issue for about six to nine months.

Interviewer: You would have thought the federal government would –

Respondent: Wouldn't have enough resources?

Interviewer: Consider that before you make announcements about … anyway.

Respondent: It was crazy, it was really bad. And people were just sitting at home, idle, doing nothing. Yeah, it was a bad time.

Interviewer: Alright. And so, are there any employment opportunities that you're aware of that are offered to migrants' children when they're finished school or university? Anything specific for migrant children?

Respondent: What do you mean?

Interviewer: I honestly don't think this is a great question. Other people have just said that there's not … that they're just … it's just the same as anyone else in terms of society.

Respondent: Oh, yeah.

Interviewer: Like, when they're finishing school or university.

Respondent: It is, it's not what you know, it's who you know. Because it's like anyone, really, often. Yeah, no, it's probably it's harder because they don't have any … it's almost like they're a blank slate with no history in Australia.

Interviewer: Yeah. No cultural capital perhaps, or no networks.

Respondent: Networks.

Interviewer: And maybe employment history.

Respondent: Employment history is the problem, there's a … yeah, it's like … it's a blank slate, there's … yeah, there's no networks, no employment history, even with schooling it might just be for the last two or three years in Australia. Yeah, it's difficult.

Interviewer: And what about employment opportunities more generally for new migrants; so not just children? Is there anything to help specifically with employment?

Respondent: Every man and his dog is trying … is running employment programs; employment programs specifically for newly arrived. And it's still bloody hard, like twice as hard.

Interviewer: Yeah, what are some the –

Respondent: Hard to find a … What are some of the issues?

Interviewer: Yeah.

Respondent: God, have you got an extra two hours?

Interviewer: It's really that difficult?

Respondent: It's really difficult. Biggest barrier is English, obviously. Racism. People have had to change their names for their resumes, just to get a look in.

Interviewer: Yeah, right. Yeah.

Respondent: So, we know that there's some sort of … there's biases and stuff out there. Not to say that there aren't great people who want to give refugees a shot, because there are, but qualifications getting recognised is a big issue. Work history, no work history in Australia, which people want the local experience. How do

you get the local experience without getting a shot, you know? It's a catch 22. Those are the main ones.

Interviewer: Just kind of links into the next question, but overall, what do you think the key challenges for new migrants that you work with are in adjusting to life in Australia?

Respondent: Uh-huh. Key challenges.

Interviewer: You might have said them all already, but –

Respondent: Probably. English language, number one.

Interviewer: Yeah.

Respondent: Because if you can't communicate or understand things out in the community, then you're not going to be able to access services, you're not going to be able to ask for services, and you're not going to be able to learn about services.

Interviewer: Yeah. So, what's happening with the English language? Because this has also been a common, very common theme. Are the services provided not sufficient? Are people not taking them up, or …?

Respondent: Yes, a bit of column A, and a bit of column B. The word in the community is, oh you don't need English, just get a job. But …

Interviewer: Just get a job and you'll learn English, or just get a job?

Respondent: Just get a job. Because that should be your priority, is to get a job. Rather than sit and waste your time learning English. And that's the word coming from the local community, their community. You don't need English, because we're all here, we all speak your language. Just get a job, you'll be fine.

Interviewer: I guess it comes down to how many jobs can they provide?

Respondent: Exactly, not enough. Nowhere near enough. I think it's slow going learning English, particularly for the groups here who are all speaking Arabic at the language schools and they're not practicing English. They're sitting in the classrooms speaking Arabic. They're not learning as quickly as they could. In a mixed group, in, say, the west where we also work, they're learning much quicker because they're not all speaking the same language, they're forced to have that common language of English to communicate with each other. So, they're learning a bit faster. A lot of people are dropping out of English classes; they're not engaging as well as they should be, because they're feeling pressure from the job actives to get work. Luckily, they're getting that extra six months in reprieve from next year, to not have those mutual obligations pushed on them. So, they can stay in.

Interviewer: So, it's currently six months, right, it's going to be a year?

Respondent: An extra six months, yeah. So, that will hopefully encourage people to … just to stick with the English for a while. Get it as good as you can. But then there's no pressure on them to actually enrol quickly. I think they have to enrol within six months, but they should be enrolling straightaway. So, some people will wait three or four months, or five months to enrol, and then start learning. Ideally, they should be learning straightaway. Yeah, I think those are some of the main reason why English is still a problem, and the 510 just isn't enough.

Interviewer: Yeah, I was considering that myself. I've looked … anyway, let's be honest … it doesn't matter. But 510 strikes me as not sufficient particularly if you've come from an Arabic speaking country. It's a very different language.

Respondent: Yeah, very different. And how long would it take you or I to be proficient in French, or Spanish, or German, or whatever? It would take a good year or two to be at that really good level where you could go and …

Interviewer: With the language that is quite similar to our own.

Respondent: Yeah, yeah. It all takes time to be at that level where you can actually confidently go into a job interview and sell yourself to get a job or whatever it is. Yeah, to think that 510 hours is enough for that, then I think people are kidding themselves.

Interviewer: And so, beyond the English language, is there … what are some other key challenges?

Respondent: To …?

Interviewer: To just … sorry, if we go back to settling in Australia.

Respondent: Knowing, I think, coming back to knowing about what services are available if you're not aware of what's available, such as settlement services. Then you're going to take a lot longer to settle, because you're not going to know about legal services or whatever else is out there. Community groups. I think isolation could be one if you don't have family here or don't know anyone. That is going to affect your mental health, it's going to affect your ability to engage in the community and settle better. Also, torture and trauma experiences, it's going to be a massive factor of people settling. And we know most people who are of a refugee background will have some form of traumatic experience in their lives, even if it's simply just leaving home, that's a traumatic experience. So, getting … coming to terms with that is going to delay, I think, a good settlement outcome. What else? Yeah. In my mind, those would be the main ones.

Interviewer: Yep, alright. And finally, what would you like to see as possible solutions to helping or supporting migrants to adjust well to life in Australia?

Respondent: Uh-huh. More English language hours would be one. Maybe more time and resources put into educating people about mental health and re-framing it. Re-

framing it in a way that would help people understand that there's nothing wrong with accessing help.

And also, I think I keep coming back to the key idea of this, of what this research is all about, which is settlement service literacy. Which is about, probably even prior to arrival, before they come to Australia, actually giving people the really good picture about what settlement services is all about, what they can access, what a youth worker is, for example. Really basic stuff that people come and don't understand. What is a case manager, what can they help you with? Really basics about that level, that first level of settlement literacy.

Yeah, I think that's really important, because people aren't accessing the services, because they don't know about them, or they're forgetting. It needs to be drilled in; you can access these services for up to five years. And I think that five years needs to be extended to seven or eight, or ten years as well. I think we all know five years isn't enough to … your settlement journey is over after five years. It's not. It goes on for many years after that, many years.

Interviewer: Yeah, for sure. So, that's the end of the interview. Is there anything else that you want to add?

Respondent: I don't think so.

Interviewer: Yeah.

Respondent: I think everything I wanted to say is in there somewhere. I don't know how articulate I was.

Interviewer: No, I think that was very articulate, actually.

Respondent: Thank you.

Interviewer: Fit in nicely, I think.

Respondent: Oh, good.

Interviewer: So, thanks for your participation, really appreciate it. I'll say this at the end of interview at four thirty-six.

Respondent: Oh, nice one.
