# Supplementary material for: The importance of information acquisition to settlement services literacy for humanitarian migrants in Australia
Source: PLoS One. 2023 Jan 6;18(1):e0280041. doi: 10.1371/journal.pone.0280041 (PMC9821785; doi:10.1371/journal.pone.0280041)
Supplement: S1 Data — (ZIP) [file pone.0280041.s003.zip › SP_05_NSW.pdf]

Interviewer: OK, I'm at (SERVICE NAME) on (DATE). It is now 11:37, and we're starting the interview with (NAME). So, thank you for taking part in this research. Just for clarity, for the purposes of this research study when I refer to migrants or newly-arrived migrants I'm referring to people, migrants that have arrived in five years or less to Australia, and it includes both refugees and migrants.

Respondent: Sure. Thanks.

Interviewer: All right, so the first set of questions are about services being provided by your organisation to assist newly-arrived migrants settle in Australia. So we'll start with if you could tell us about the services your organisation provide.

Respondent: Sure. We provide both targeted settlement services, but we also provide generalist services, mainstream services, predominantly though to migrants and refugees, partly because of the geography of where we work. [redacted] so if that's our geographic service footprint then you can't but not be working with migrants and refugees. The two targeted… we only provide one settlement program directly to clients, and that's the Humanitarian Services Settlement Program with the HSP. [redacted]

Interviewer: Right.

Respondent: And that's eligible to all refugees, but only eligible to a very small cohort of migrants. So family migrants and business, there's a lot of criteria around that, who amongst the family migration stream can access that program is… but that program does a lot of community development type stuff, as well as individual client stuff. And that program, (SERVICE NAME) [redacted], but it's delivered by 22 organisations across New South Wales.

Interviewer: Many of whom I've been…

Respondent: Yes, that you would have been in touch with. Yeah, but they are the delivery... the clients of that program are not our clients. [redacted]

In terms of mainstream programs then, probably the biggest one, although it's been defunded now, or it's about to be defunded, that we would have is a program, we had a disability program called Ability Links which was funded by New South Wales government, which has

now transitioned out because of the transition to the NDIS, and that was a program where we worked with a very person-centred program and strengths-based program, funded by New South Wales government to help people with disability, with or without a formal diagnosis of disability, to get support for the goals that they've identified for themselves, and achieve those goals. So it was very much a soft-touch kind of approach. It was very much, it could be any goal, and also that program used linkers to actually link people from community with disability into either service systems, but also into the activities that they wanted to do.

The other part of that program was Linked Organisations, so (SERVICE NAME) as a deliverer of Ability Links could work with a karate school for example. You imagine a teenager with disability, she wants to do Kung Fu, well why should she have to travel, or her parents have to take her seven suburbs away to join a karate class when there's actually a karate school three doors down from where she lives. So what (SERVICE NAME) would do or what the program would do is work with the karate school to say 'here's what you've got to do with a teenager with down syndrome to help them in their learning in a class like karate'. So it's that extra layer of inclusion.

Interviewer: And that program, you said it was more general?

Respondent: It's generalist, yeah.

Interviewer: Generalist, so that's available beyond the five years?

Respondent: Yes, that was available to any resident of New South Wales, any resident with or without a visa, with or without a permanent resident, any of those kind of things. It was a very open program. So I guess that's one part. We're also a small Job Active provider. We're also a provider of multi-cultural foster care, so we're involved in family preservation and in foster care as well. And we also have some work in some other employment programs as well, like Parents Next, and those kinds of programs. But the only dedicated programs that targeted settlement service program, where we actually deliver it to individuals is the HSP.

Interviewer: And can you tell me…

Respondent: Oh sorry, we also have a community hubs program as well which is funded by Scanlon Foundation, we're a provider of that program.

Interviewer: Would you be able to tell me a little bit more about the HSP program?

Respondent: Sure, yeah. Look…

Interviewer: Just in terms of what's the main sort of… I know English…

Respondent: Well it's not really English. I would say the targeted settlement services that are funded by the Australian Government are things like the HSP, the AMEP, the funding that the Australian Government gives to Torture and Trauma Services nationally, the TIS, and I've probably forgotten some, I feel like I've forgotten some. Oh, the OSCO, which they deliver offshore which is provided to refugees before they arrive in Australia.

Interviewer: Right.

Respondent: The HSP is the latest iteration of a settlement program for newly-arrived refugees, the people in the humanitarian program. There's a very high uptake of that program, but essentially the goal of that program is not provide everything to somebody on arrival, I would say that the goal of that program is to promote access to the universal services that already exist in Australia, and the mainstream services that exist in Australia. No program, no settlement program can be Medicare. No settlement program can be the Education Department. No settlement program can be all of the myriad of human services that all of us need to actually function in our society.

So the goal of the HSP has been for quite some time to be early part of orienting new arrivals, new humanitarian entrants to the services that they are entitled to. So enrollment in schools, enrollment in banks, learning how to use public transport. Very early, early, early, orientation to the service system and to the community. The program itself is very much targeted though towards individual clients. The latest… it's been around, I think it was called IHSS originally, it then got called HSS, it's now called HSP. The HSP has only been in existence about 18 months or two years. Probably the biggest changes that I've heard of in the HSP probably, is one of the good things I think, is that I don't know whether or not it will translate into better outcomes, but now everybody has a case plan, whereas before the case was actually a family of individuals and so the head of the household would often be male, or the head of that case would often be male, and irrespective of whether or not the head of the household is male or female, you can see how it might run into a few problems, when there might be eight individuals and they're all called a case. It's not very individualized in some ways.

Interviewer: No.

Respondent: Even though they're all probably leaning towards in the same direction. Now everyone has their own case, everyone from… if there's eight members of a case, and they still do use the word 'case', there'll be eight individual case management plans for those people. So there are changes that have occurred. But essentially it's a program that has been evaluated by Ernst and Young. We've done our own internal evaluation here of our delivery not of the HSP, but the HSS, and really it's that early stages sort of stuff. People get generally under

the old HSS I think the average discharge time for us was about six months, now the HSP allows us I think it's more like 12 to 18 months.

Interviewer: Oh great.

Respondent: But it's a program where it's really got a set of defined KPIs, when those KPIs are reached the client can be discharged. That's kind of like how it works. A good feature of the new HSP is that in the past there was also this Complex Case Support Service in the HSS, which was delivered by agencies across a separate tender, it was dealt with in a separate tender. Now my understanding is that all of the providers of what used to be called Complex Case Support, it's now called Tier 3 and it's within the HSP. So what that means is that within (SERVICE NAME) we can, if we have people who have higher needs than we can meet in the box standard part of the program, we can escalate those people with departmental approval, into a much higher level of support.

Interviewer: Oh, that's great.

Respondent: And that higher level of support is intended to be, as the old program was, complex case support, it's meant to be a place where a range of agencies can work together to actually address higher needs for a period of time, and then maybe the client can actually be referred back down to a lower level of support. So it's a level of intensive support, but it means it's not a referral out, it's a referral within the program, and so it can scale up. But it requires departmental approval.

Interviewer: Right. And I imagine if you're able to extend the case work from six months up to 18, that there would be also room, not just for referral within what (SERVICE NAME) delivers, but also outside of (SERVICE NAME) to the other people that… the MRCs and [redacted] other consortium partners.

Respondent: Well when they exit the HSP they are provided with a referral to the SETS program which is delivered by the consortium of the New South Wales Settlement Partnership.

Interviewer: So it does flow?

Respondent: It does flow, yes.

Interviewer: OK, great.

Respondent: And what I guess we have found as a provider, and I don't know whether this exists in other states and territories, but what we've found is that because we have a lot of those consortium providers are members of (SERVICE NAME) , in fact they helped to establish (SERVICE NAME) , they're founding establishing organisations of (SERVICE NAME) , that there's… [redacted]

[redacted] theoretically what can occur is that somebody can leave the HSP desk in a building and then maybe go up a floor or go down a floor and be at the SETS desk in the building. So there's a level of familiarity, and we provide HSP in a decentralised model, [redacted] They're still fundamentally the same, but the flow, there's more teams now in Liverpool and Fairfield than there were when I started five years ago, and that's just because that's where people are settling.

Interviewer: Right, yes.

Respondent: And because of the Proposer visas which is part of the HSP, sometimes it's a family member…

Interviewer: Which visas did you say?

Respondent: One of the refugee visas is called a 202.

Interviewer: Yes.

Respondent: And it's a Proposer visa.

Interviewer: Oh right.

Respondent: So if I'm a family member and if I propose somebody from my family to come to Australia, then it doesn't take rocket science to realise that they're probably, when they first arrive in Australia, they are probably going to nominate near where I live as the place that they want to be, and so the Proposer visa sometimes, the mix of Proposer and Non-Proposer visas is a decision of the Department of Immigration, the Department of Home Affairs, so if there's a lot of Proposer visas, the proposers will generally bring… people will come to where the proposers live. And at the moment a lot of the proposers, or were a couple of years ago, were living in Fairfield. And so it makes sense.

Interviewer: Yeah I just met with (SERVICE NAME) yesterday.

Respondent: But it's not a function of the refugees themselves, it's a function of what the Department of Home Affairs is doing. If they're deciding that they want mostly proposers, then they have to also wear the fact that wherever the proposers are living is where the people being proposed are going to settle. Like you're not going to propose that one of your close relatives comes to Australia and then goes to Wagga.

Interviewer: That's right.

Respondent: That doesn't make any sense.

Interviewer: Oh good, thank you, because I've been searching for information about HSP program, and it's very difficult to find that sort of detail online in documents.

Respondent: Right.

Interviewer: Just about the practicality of how that works. So thank you, that explains it well. So some of these questions may not be so relevant to your role, but if you can add some information it would be great. So what are the relevant services that other organisations are providing in terms of helping newly-migrated people to settle in Australia?

Respondent: Well yeah, that's probably not a question I can answer very well, that would probably be a better question for somebody else at (SERVICE NAME) to answer, but what I would say is that we must always remember that the role of settlement services, and sometimes I think even settlement services themselves forget this, is that the role of settlement services is not to be the be all and end all of what gets delivered to new arrivals. It's tempting to do that, but then what you do is you end up with a universal service system that isn't responding to the needs of people who are permanent residents of Australia. And that's very, very, very damaging because what we end up with, if we haven't got a Medicare system that's responsive to newly-arrived refugees, no amount of investment in settlement services is going to change that because we don't have GPs, we don't have practice nurses, we don't have any of the things. We don't have the skills to respond to the medical and primary care needs of refugees. What we need is a primary care system that's responding to needs of refugees.

Interviewer: Right.

Respondent: And so if we have a gap, say for example in primary care for… in education for example, if we have kids that aren't attending school, and not getting the kind of education pathways that we need, again no amount of investment in settlement services is going to turn that ship around, it's the investment… or not the investment, but it's the reorientation of the education pathways that needs to occur. The targeted services can only do so much, like the AMEP, like the Torture and Trauma Services, like the HSP in a sense, they can only take things to a particular point, but after that it has to be as Australian residents, people have to have a service system that's responding to their needs. You know?

Interviewer: Yep, great. Can you tell us about who you collaborate with to do your work?

Respondent: [redacted] We collaborate with, in terms of the service delivery

area we collaborate with other organisations who deliver the HSP across the country, so the providers in Queensland, in Victoria, in WA, and the other providers in New South Wales. We collaborate with researchers, we collaborate with, I mean there's so many different layers to the level of collaboration that's actually occurring. But in terms of, I think the question is primarily guided towards service delivery, there would be… our service delivery people would be meeting with people like Department of Human Services, they would be meeting with New South Wales Refugee Health Service, so they'd be meeting on regularly basises to… there's a structured meeting that is no longer funded, I understand, under the contract called the HSPN, [redacted]

Interviewer: and Health.

Respondent: But then mainstream organisations as well, the mainstream NGO sector and stuff.

Interviewer: Great, thank you. And are you aware of any services that are needed but not available?

Respondent: Well I suppose probably the biggest issue is that I think for humanitarian entrants [redacted] it's really clear that apart from people taking asylum but for humanitarian entrants they are eligible to the full suite of settlement programs. The difficulty is that that's a tiny, tiny, tiny part of the number of new arrivals that are arriving in Australia, and some of the family stream migrants are eligible for settlement programs, not the HSP but the SETS program, but very few of them even are eligible, so you have a lot of people who are arriving who aren't technically refugees, so they don't meet that refugee status dissemination but you'll have a family that arrives on a refugee visa, and then four or five years later somebody from their family might come in on a family or a spousal visa, well they're coming from a refugee-like situation. They're not refugees and refugees should stay what it is, it needs to be clearly defined, and it needs to stay that way. I guess the difficulty we have is that the eligibility is very, very, very tight on targeted settlement services, and we can't provide settlement services to everyone who arrives, totally get that, like that's not a sustainable way for

government to invest in settlement, but essentially what we're doing though, is that the vast majority of people who arrive in Australia are left to their own devices about how they integrate or don't integrate. They are new arrivals. If you were to take the number of people over the last five years, which is the subject of your study, who've arrived in Australia, the vast majority of those would have arrived and wouldn't have been eligible for any kind of settlement service of any description, ever.

Interviewer: So the HSP I understand that eligibility. What about the SETS program? Is that a little bit broader?

Respondent: The eligibility for that… a little bit broader. There are family stream migrants who are eligible, the criteria I'm not across exactly. (NAME), if you manage to secure an interview with him, would be a little bit clearer on that.

Interviewer: OK.

Respondent: But essentially it is also fairly limited.

Interviewer: Oh, so that's a very important factor that I hadn't considered.

Respondent: But I noticed in your summary that maybe in the summary that wasn't clear to people as well. I think that we have to be realistic about what settlement services can deliver, and I would emphasise again that settlement services cannot deliver the broad suite of universal services that have to be available to help all of us get around the world, whether it's public education, whether it's… the AMEP for example has a role, obviously a massive role to play in helping to acquire English language, to provide English language training, but again they're limited to that aspect of the equation.

Interviewer: So people for say language courses, English language courses, and they aren't eligible for the HSP-funded 500 hours or whatever it is…

Respondent: Whatever it is, yeah.

Interviewer: How do they… do they have to navigate it themselves?

Respondent: Navigate themselves, yeah.

Interviewer: Basically, yeah.

Respondent: I think through a TAFE or something like that, if you arrive in Australia on other kinds of visas. And what we would say is that the vast, vast majority of people who arrive on a skilled visa haven't got any settlement services, we're not saying that everyone in the skilled visa category, but people's circumstances change, and what we would say [redacted] is that there seems

to be very few mechanisms to acknowledge that everyone needs in a sense a degree of settlement service, something to help them orient to life in Australia, and some people might need a settlement-like service when they need it. So you can arrive here as a skilled migrant, let's say with your spouse, and then you have a child, and let's say then a parent acquires a disability or passes away, well then the safety nets for those types of situations are very, very, very difficult to navigate, and it's based very strictly on… of course it's very easy for the government to base it very strictly on which visa you currently hold. Like if you hold that, and there's hundreds of visas…

Interviewer: It's black and white.

Respondent: So it's just very black and white.

Interviewer: Yes, yeah.

Respondent: And I think there have been some advances made in relation to family and domestic violence and being able to access for women and spousal visas and stuff like that, but I guess the system doesn't really acknowledge that to a degree everyone needs an orientation to Australia. If you come from wherever, from Bangladesh on a skilled visa, and yes, you might be working in IT, and yes you know, often what's happening is the primary applicant is a skilled visa holder, but the secondary applicant, maybe their spouse or their children, aren't actually factored into these kinds of arrangements. And so what you can theoretically have is somebody arrive on a skilled visa to plug a skill shortage in Australia, and the other people on their application are just left to their own devices.

Interviewer: Right. Wow, wow. It's very interesting. And that Building Life in Australia manual, I've had a look at that, so I suppose that manual would be recommended to those people that aren't eligible. But then it's assuming that they could follow a manual like that, literacy levels and all that sort of thing. So yeah, left to their own devices really.

Respondent: And I think if you end up speaking to organisations that aren't in the heartlands of say Fairfield, Liverpool, Blacktown, that side of Sydney… because I remember when I went and spoke to one of the member organisations of (SERVICE NAME) over towards the eastern suburbs where they see very few refugee arrivals, practically none, five a month or something like that, quite low, but what they would see quite a bit is situations where a skilled visa holder would arrive, the spouse would then arrive, let's say the skilled visa holder is male, a spouse might then arrive a year or so later, that spouse has no access to settlement services, no access to anything around building connections with community, social engagement, which is really, really, really, really important, and so what you then have is maybe the spouse she has children, and her first contact with in a sense the world potentially, is when the children go to school.

Interviewer: Yeah, wow.

Respondent: But even then it's very limited because there's nobody really facilitating that. Now we do have a community hubs program that tries to help in those situations. We're not the funder of it, we're the deliverer of that program, but those kinds of programs aren't really delivered routinely in places, you know? And so it is right and proper that the focus is on refugees, that is right and proper. However it's very hard to get attention to seeing if there's ways that we can actually improve the settlement experience and the integration experience for people outside of some of the rigid categories.

Interviewer: Which are probably higher in numbers.

Respondent: Much higher in numbers, much lower in need.

Interviewer: Yeah.

Respondent: Probably. Not much lower, at times equal need, but probably as a population lower in need. Yeah, I would say definitely.

Interviewer: Wow. Wow, that's very interesting. I'm going to tweak some of my questions now in my survey to distinguish the differences there. That's great feedback. Are you aware of any services that are over-utilised? There's high demand for, long waiting times?

Respondent: Not really. Sorry, I don't really know, I'm not invovled. I'm sure there are, but you know…

Interviewer: A lot of these questions as I said are more directed at…

Respondent: …service delivery, yeah.

Interviewer: …service delivery. And under-utilised, the same sort of flip side of that. Measuring effectiveness of services, so you already said that you have…

Respondent: Well we've evaluated… the SETS program was evaluated in the last funding before the tender was announced, it was evaluated independently… it wasn't called SETS then, it was called the Settlement Grants Program, and it was evaluated by UNSW.

Interviewer: Was does it stand for now?

Respondent: Settlement Engagement Transition and Support. But if you Google online, you'll find the UNSW Social Policy Research Centre, the SPRC evaluated the Settlement Grants Program in about 2017 I think, or 16, 17 maybe. So there's a report there available on that. And Ernst and Young evaluated the HSS, which was the precursor… sorry, it's

EY, they don't call themselves Ernst and Young anymore, they call themselves EY. They evaluated, on behalf of… both of those evaluations were commissioned by the government.

Interviewer: Right. Do you know what year that was done, that one?

Respondent: 2015 I'd say. Yeah, about 2015, I think.

Interviewer: Great.

Respondent: We also evaluated our delivery of HSS as well, there's a summary of the findings available on our website.

Interviewer: Great, great. Thank you.

Respondent: And probably the thing that I would emphasise is that a lot of the time people focus a bit too much on the service side of the equation. The social capital and the building social capital, building social bonds, social links and social bridges is something that is very important for (SERVICE NAME), and the reason I'm remembering it is that one of the things that we found in our evaluation of HSS was that we seemed to have a very… we compared ex-clients of our program with the people in BNLA, and one of the things that we found was that our ex-clients of HSS seemed to have a higher level of social participation than the broader national average in BNLA. We used the same questions from BNLA as a comparison point. And as an organisation we invest very heavily in community engagement and in arts and culture. We have an Ingnite Program which helps people to establish their own business. Basically lots of different ways, lots of different touch points other than the very transactional sort of service system in a sense. And so we think that's incredibly important because…

The UK Home Office has developed a very excellent framework on integration. They developed it in about 2004, but they've updated it this year in May and released it, and they've come up with a whole series of domains of what actually contributes to integration, and there's an entire layer of that part of the puzzle that's about social connections, and if you haven't got healthy social connections within and outside of your ethnic group, and with the service system that you know, but also the ability to jump into other types of institutions that you've never heard about, and then you're probably not on a great pathway in terms of integration. Because when in everyone's life there are going to be things that knock us off our perch, and if you haven't got those social connections, those bonds, that social capital, than the chances of you recovering from those life events is diminished. Even refugees who have had terrible life events, often, before they've come to Australia, it's then events, a poor health diagnosis, a family breakup, those kinds of things.

Interviewer: Right. Thank you. So the next set of questions are around migrants adjusting to Australian culture and society.

Respondent: Sure.

Interviewer: You've already touched on it, your natural pathway… your train of thought is following our questions, so that's great. I suppose out of these little dot points under that question, if there's anything you think firstly your thoughts about how migrants and refugees understand Australian culture, and then some of the key issues and challenges that process of adjustment that they may face.

Respondent: Well I think probably one of the biggest challenges is that we often see it as them adjusting, when in fact it's… that's the biggest challenge.

Interviewer: Yes, that's right.

Respondent: I think that's actually the biggest challenge, and especially some of our political… public discourse tends to see it 'they have to integrate to us' and that's again, I go back to that UK Home Office framework of integration, integration is a two-way process, it's not a one-way process. It's multi-directional, so it moves in lots of different funny ways, and it's also important, it's a response to context. So the settlement of an individual or a family in say Fairfield might be different to the settlement of a family in Dubbo. And it's not that the Fairfield family are going to settle really, really well and the Dubbo family are going to settle really, really poorly, it's not as simple as that, but it is responsive to actually where they are, how things are going. And it is affected by things like their own circumstances, but also the sense of welcome they feel in the community, and stuff like that.

Interviewer: And whether they're the only family from their background or whether they're in a whole community of people from their background.

Respondent: Yeah, that's right. And either of those can be good… there's good and bad in probably either of those situations, there's strengths and weaknesses to all of those things. But I suppose again the issue becomes, and probably I'd use the example of (NAME OF LACATION) for (SERVICE NAME) work, we provide the HSP in (NAME OF LOCATION), and about a year and a half ago the government decided to create (NAME OF LOCATION) as a new settlement community. And probably one of the things that we emphasised in our work there is that you need to work with everyone in the community. We've paid as much attention to the host community in (NAME OF LOCATION), as we have… or not as much, we've paid attention to the host community in (NAME OF LOCATION),, and probably the thing that we would say is that we've worked with all of the community in (NAME OF LOCATION),. And so we haven't just worked with the people who are enthusiastic about

the arrival of refugees, we've attempted to calibrate what we do in relation to everyone in the community.

Interviewer: Oh, excellent.

Respondent: It is legitimate for people… there are people in the (NAME OF LOCATION), community who have higher needs than some of the Yazidis who are arriving in (NAME OF LOCATION),. The Yazidis are not the only people with high needs, potentially, in (NAME OF LOCATION), there are people already in poverty in (NAME OF LOCATION), there are people who are struggling, there are people who are experiencing domestic violence, there are Aboriginal and Torres Strait Islander people living in (NAME OF LOCATION), there are people who have insecure employment in (NAME OF LOCATION), all of those people, and the Yazidis are the (NAME OF LOCATION) community now, and you don't create a special pathway, in a sense, for one part of the community.

So an example that we've put… I think we've put it in our research… oh, and one of the things we've done is we've done research with the host community in (NAME OF LOCATION), we didn't initiate this, it was initiated by the University of New England, a researcher there called Sue Watt, and it's about to be published later this month, and what she has done over the last year and a half is she's done telephone surveys of (NAME OF LOCATION) residents, randomised representative samples of (NAME OF LOCATION) residents, asking them how they are feeling, taking a pulse about how they're feeling about the arrival of refugees. And there are people in the (NAME OF LOCATION) community who are concerned about the impact of the refugee arrivals on their jobs. There are also people who are expressing concern that they don't know if there's enough services to support them, so that concern is an empathic concern as well, it's not just a negative concern, and even the one about jobs as well, it's a two-way street. And there's also people that are concerned about will they integrate. But still integration is a two-way street.

And so an example that we would say, a concrete example of looking at the whole of community would be my understanding is that we were offered, (SERVICE NAME) was offered children's clothing, and some people wanted to offer children's clothing to (SERVICE NAME) for the Yazidi children, right? And they wanted to deliver that children's clothing only to an (SERVICE NAME) office. So that sounds OK, but when you bring a lens of whole of community into that you realise that hang on a minute, firstly what we want to do is we want the Yazidi families to know to go to charity shops when they haven't got money, and when they need to get low cost children's clothing. So we worked with that donor to say, no, you need to give those clothes to one of the charity shops in the town, because that's where we're sending them. But it also recognises that there are other children in (NAME OF LOCATION) who may have a need for low cost clothing. Because

you need to think of the whole of the community, and not allow… and the donation, it's not to invalidate the donor or the donation, but you can have a situation where it's not healthy for community…

Interviewer: You're setting up an 'us and them'.

Respondent: You're setting up an 'us and them' if you have this. And so I think that's a very concrete example of the way that we work, and what that's doing is the Yazidi families who do need access to low cost children's clothing are going to the charity shops, not coming into (SERVICE NAME) office and rummaging through boxes, they're actually going to a place where they might even find something that's better than what was in the (SERVICE NAME) office. And they get access to not just the children's clothing, they then realise that you can get an electric kettle there as well, and you can get this and that and the other thing. And then their independence and agency, and they're interacting with not another (SERVICE NAME) employee, but the employees of the charity shops, and that's an integration.

Interviewer: And that whole of community approach in terms of funding specifically designated for refugee settlement etcetera, how do you navigate that with opening it up to a broader audience?

Respondent: Well I guess that the issue is that current contract in HSP doesn't fund the community engagement that we're doing, so we've had a dedicated community engagement officer.

Interviewer: So it's open already?

Respondent: Yep. But I guess it's easy in (NAME OF LOCATION) in the sense that because it was designated refugee settlement area that's been the focus of our community engagement efforts, with the Council, with the local service providers, with the local community, so yes, but I guess the problem in some ways is that the Government and the contract doesn't really value that work. Sorry, I think it does value the work, sorry, that's not true, but because they feel it's a bit too touchy feely, they don't really invest in it. They don't say to the providers of the HSP we want a dedicated community engagement strategy from you. Now a community engagement strategy is as important in Fairfield as it is (NAME OF LOCATION). It's more pointy in (NAME OF LOCATION) because it's a new location, but it's still important in our work wherever we are, even though refugees have been arriving in Fairfield for 40 years, 50 years, 60 years.

Interviewer: Great, thank you. So I think we've already touched on all of those, some questions around social support, health and wellbeing, I think we've probably covered enough of that. In terms of financial literacy can you tell us about some of the programs you're aware of around supporting people around financial literacy, income generation, money management, anything like that?

Respondent: Well there's a degree of that in the HSP. There is a degree of the early parts of something like that, but I'm not aware of… I'm just not in the service delivery, so I'm not aware, [redacted] and also other providers do try to do additional work in that area.

Interviewer: Yes. And there's one question here around some of the culturally specific dynamics that impact people in terms of financial management or demands in terms of sending money home, dowry, all that sort of thing. So those questions have been really important I think.

Respondent: Sure. I guess though the decisions that people are making in relation to where they invest, they're permanent residents of Australia, and how they spend their money is their business really. I guess what we try to make sure is that people are not getting scammed, and I would think there would be a level of budgeting that we would be supporting as well, that would be my sense of things. Because it is very different to have your money coming in, let's say if you're on a Centrelink benefit, your money coming in every fortnight. It's very different to what people have been used to, maybe they've relied on extended family to cover certain things, and now they've got their own independent income coming in. But it's an independent income but we know that a Centrelink benefit is practically impossible to live on in any meaningful way, and we know that New Start is grossly insufficient for most people to live meaningful lives, they're living below the poverty line if they're on it.

Interviewer: OK. In terms of legal supports? Again programs around visas and supporting people with visas, family violence…

Respondent: Again I'd go to the service delivery. But again I'd say the providers of settlement services can't be anywhere. To provide legal advice in relation to a migration matter is against the law. If you and I were chatting over a barbeque you can tell me how you think I should get my visa because we're talking and having a conversation, but if you're a part of a settlement organisation doing the same thing to me, only registered migration agents can provide that advice to people in relation to visas or non-visas and stuff like that. It's very, very… it's as strict as interpreter, you know. No more than if I speak a language other than English I can't just waltz into a hospital and be the person that interprets your consent for a medical procedure. Just can't do it.

Interviewer: That's right. Great, thank you. Are you, these questions may not be relevant for you either, but they're around movement of people from one place to another, and the question is what do you think some of the key reasons are for the movement of your clients, so it's a bit different, you're not working directly, from one place to another, or one suburb to another? And the different stages of that first five years.

Respondent: Sure. I suppose what I would say that (SERVICE NAME) would say is that it's people's right to move, and they're permanent residents of Australia, and I know that in terms of regional settlement there's often this worry that people go to a regional area, but then they don't stay, there's a query on the 'stay' part of the equation. I would say that the best way to address the 'stay' part of the equation, especially in regional areas, is make sure that you get the community engagement right. If people are only in a regional area because they happen to have landed a job there, and if there's no social engagement, if there's no real efforts of the community, and in fairness the host community might be welcoming, but the host community needs a facilitator to help match that thing, and if people have developed connections in the local community outside of their job or whatever primary reason got them there, they're much more likely to stay in those places, because people don't like to move just simply to move, and even if they lost a job, if they're connected in the community they're far more likely I would say to stay there and to try and find a different job. But if they're very unconnected to that community, as soon as they lose that job they are off, they are gone.

Interviewer: That's right. If they're Muslim, there's no mosque, or there's no shop that sells the food that they're used to eating.

Respondent: Yeah. But even if none of those things are there, even if none of those foods and places of worship are there, if they're connected to that community and feel like they belong there and they have friendships in that community, they might even forgive the fact that it doesn't have the food, and it doesn't have the place of worship and it doesn't have this, and it doesn't have that. If they actually feel like they really belong at the place after two or three years say, and then they lose their job, well their choice is go all the way back to wherever it was and start all over again, or stay there and see. And they're far more likely if they've got friendship networks, if there are opportunities work-wise, to be able to get those opportunities, to hear that the job is coming up in such and such a place.

Interviewer: [redacted]

Respondent: Oh right.

Interviewer: Yes. So I imagine it would have been like that, it would have been Wagga of… you know.

Respondent: [redacted]

[redacted]

Interviewer: That's right.

Respondent: And so there's this huge concentration of them, they would have all gone over in the 1920s, 30s, that's when they went over, and they've set up shop there, and there are now grandchildren and great grandchildren and stuff like that, and the generation that left have passed away, because they'd be in their 100s now, they've be 110 sort of thing, or 100, so there might be one or two of them alive of the original actual people who emigrated, but then their nieces and nephews also went, so it's just a link… chain migration, not link, they call it chain migration.

Interviewer: Yes, our family's the same. Chain migration. Oh right, that's great. So the next lot of questions are around education and literacy programs, now the same applies to these questions. You've already talked a little bit about some of the early education…

Respondent: Well HSP helps them to enroll in schools and stuff like that, and by international comparison apparently we're doing quite well in terms of the integration into school systems in Australia of refugees at least. And New South Wales government also funds a targeted program, ESL centres, I think that's what they're called, no I've got that wrong, I can't remember the acronym.

Interviewer: It used to be ESL, it's called something else now, but I know what you mean.

Respondent: Yeah, they're attached to primary schools. I know but they have these centres attached to primary schools…

Interviewer: Oh yes, Intensive…

Respondent: IELC, Intensive English Language Centres, IELC, which is a New South Wales government initiative, to help in those areas where there are a lot of refugees arriving, mainly refugees arriving, and that's a really good help.

Interviewer: Are you aware, it's not in the questions, but I've done some work on it in the past in the community sector around supporting bilingualism. So in early childhood, as soon as children and parents engage in the education system the focus is on learning English at the expense of

losing your home language, your primary language. Are you aware of any programs that support?

Respondent: No, but I would have thought… when our children were in primary school my understanding was that bilingualism was highly promoted and they had native speakers of Chinese, they had native speakers of Arabic, they had native speakers of Greek, native speakers of… you know they couldn't accommodate all the languages that were in the school community, but they had classes, and if let's say if they had an Arabic class they had both a native speaker arm, and a non-native speaker arm, I think in the same class, but they recognised native and non-native in the way that they taught.

Interviewer: Oh right, where's this?

Respondent: [Inaudible – 52:24], near here.

Interviewer: Oh right, well that doesn't happen everywhere.

Respondent: Oh, it probably doesn't. But this wasn't a special… I think it was just a… and in the high school as well. I mean the high school sorry, I think was Saturday classes.

Interviewer: Right, yes.

Respondent: It was sort of a parent thing.

Interviewer: I think the area where I was around (NAME OF LOCATION) and that area, a lot of the early educators were telling parents to only speak English. And I suppose a lot of the settlement workers, migrant settlement services, and all those type organisations were going 'Woah', they're losing their home language and the dilemma of the parents. So another arm of the process.

Respondent: I think at (SERVICE NAME) what we would do from a policy perspective I would say that we recognise that having a job is really important in terms of settlement, or having paid work sorry, and we recognise that having English is an advantage as well, not just for work, but also intrinsically it has an advantage, but we would never suggest that English is somehow a requirement, people can actually make their way in the world, look at all the Greek and Italian migrants all over Sydney. I live next door to heaps of them who still can't speak English well. It's not like they haven't contributed to Australia for God's sake. It's what it is. Like yes, I would say they themselves would wish they could speak English better, but there were no English language programs available to them when they arrived, they went straight into a factory and they stood alongside another 100 Greek people doing difficult work.

Interviewer: And if they have a lot of their own community around them, they can speak their language, communicate quite easily with shop owners… I have a [redacted] who speaks very little English and she relies on all the Arabic speakers in Liverpool to get by. And she's fine if she's in the right place. Yes, that's right.

Respondent: She's in the right place. Thankfully she's in Liverpool and not here.

Interviewer: Yeah well that's right, she wouldn't survive.

Respondent: If she was in Hannah?? Beach she'd have a bit of a difficulty.

Interviewer: That's right. That's right. She'd be certainly isolated.

Respondent: Yeah, well she'd have more isolation.

Interviewer: OK, what do you think, if you can feed into any points here, are key issues or barriers for children of migrants and refugees? So children in education, schools and universities, the sort of issues they face?

Respondent: Well I think that… I'm not saying there aren't needs there, but I think that we generally do quite well I think in relation to the education of migrants generally, and their integration into the education system. I suppose the critical thing is that we still have a strong public education system in Australia, and if we didn't have that there'd be a huge financial… and there is a choice, and some families will make a choice that isn't about public education, but essentially having access to… it's the same with Medicare, I mean a foundational issue is going to be that if you have to pay through the nose to get either education or health, a basic level of education or health, then it's a massive barrier. So we must never forget that that's an absolute pillar about what… because if you are on a low income, and if you go to public schools, yes there are co-contributions to make, but if you can't pay you will never be forced to pay, ever. Well that's my hope. That's certainly my experience in the school that our children went to, and I think it would be the perception of just about every public educator.

Interviewer: Yeah, I think there's an expectation of encouragement, but if you can't…

Respondent: And many families can, but if you can't afford it, you can't afford it. So I think that those integration, we probably don't have the same… I know access to bulk billing to GPs in the poorest parts of Sydney… the poorest parts of Australia actually, is really, really difficult. The places with the lowest socio-economic background seem to often have very poor access to bulk billing GPs, but we still have… so there's problems with it, but if we didn't have a bulk billing situation in this country it would just be an absolute disaster. And again no amount of different service is going to address that fundamental issue, if you

don’t have that pillar of public universal services than everything else falls apart.

Interviewer: Great. Now a lot of these other questions are really quite specific to services so I’m not even going to ask them. So the last question, or the last few questions, there’s one around what do you think are the key challenges migrants and refugees face while adjusting to Australia? So anything that you’ve not mentioned?

Respondent: Well I suppose I think one of the key challenges is getting, because we’re talking here in your study about people who have permanent residency, I think one of the key challenges is them getting to the stage where they are able to equally participate socially, economically, culturally and civically, and each person will have their own journey on that, and younger people might adjust more quickly, and older people much more slowly if at all. Or gradually. But I guess that’s probably the challenge, but that’s such a broad challenge, but yes, that’s probably the main challenge. And getting that mainstream service system, which is funded by the tax payer, and the migrants and refugees are tax payers as well, to actually be responsive to what they need, and how they need it. What they need and how they need it as well.

Interviewer: Great, thank you. And final question is what would you like to see as possible solutions to helping or supporting migrants to adjust well to life in Australia?

Respondent: Well I’d probably go back to… I think we have the building blocks for the integration, we haven’t gone down the path of some of the European countries in terms of our attitudes to migrants and stuff. Now I know that it’s not as good as it was, but we haven’t had the terrible vitriol that’s happening in parts of Europe and in America, so I think we’re on a reasonable path still. But I’d still say that probably things like access to the universal service system, making sure that those access points are culturally responsive is really important.

And I would say that making sure we realise that the settlement service system can only do some much. Like I always kind of get worried when people say well when refugees arrive we need to do this as well when they arrive, and this as well, and they need to hear about this, this, and this, and it’s a bit like the school system, people kind of think there will be no time to teach children anything if everyone kind of though oh well we have to teach kids this in school, and we have to teach them that in school, and we have to teach… they wouldn’t do anything other than learn about stuff that they might need to know about at some point.

And I would say that the orientation of refugees, you can only do so much when they’ve first arrived, and adult learning principles would suggest that didactic telling people about stuff is OK, but at the end of

the day adults learn through experience, and they learn when they need to learn something. So you can't go with a cohort of refugees and or new migrants and kind of decide oh well they all need to know everything about everything, and then when they don't retain any of that information for various reasons, partly because they don't see that it's relevant to them, but partly because it's just an overload of information, so I think we have to be mindful of not overloading the settlement services part of the equation with unmanageable expectations.

And also I think we have to be very careful that if let's say ten years after a refugee arrives in Australia they don't know how to access, or their health isn't good, that's not the fault of the settlement service system that had a tiny part of their journey. But I think that sometimes they go 'oh settlement services didn't do their job', it's like well we all didn't do our job, if somebody's falling through the cracks, we all didn't do our job. There's a whole heap of people who didn't do their job. There's a whole heap of services who didn't do their job. There's a whole heap of community supports that didn't happen. It's not like oh well, point the finger over there. And if they had a two-hour session on suicide prevention, they'd be all right. Well two months into their life in Australia you can't do stuff like that.

Interviewer: No, definitely.

Respondent: And not with a whole room full of people. Because like when we've had those high profile cases like that man in the Lindt Café, you know, who was an original refugee, clearly unwell at the stage when he did that act, you kind of almost hear that not so much the settlement services, but we went wrong somewhere. But I mean how can we go wrong somewhere? The man had a mental illness clearly.

Interviewer: That's right, that was being ignored by his health practitioners, but his community for all different reasons.

Respondent: For all different reasons.

Interviewer: Who knows? Too hard basket.

Respondent: Too hard basket. Yeah…

Interviewer: Thank you! That's fantastic.

Respondent: Good.

Interviewer: So yeah, it was different interview than I've had with service providers.

Respondent: Oh, you've probably talked with service providers, yeah. But that's good.

Interviewer: But a whole set of other information that is really, really important, so thank you very much for that.

Respondent: What’s your role in the study (NAME)

Interviewer: I am… it’s 12:40 and we’ll end... I’ll turn this off... end the interview, and thank you very much.

Respondent: Great, thank you.
