# Supplementary material for: The importance of information acquisition to settlement services literacy for humanitarian migrants in Australia
Source: PLoS One. 2023 Jan 6;18(1):e0280041. doi: 10.1371/journal.pone.0280041 (PMC9821785; doi:10.1371/journal.pone.0280041)
Supplement: S1 Data — (ZIP) [file pone.0280041.s003.zip › SP_05_Victoria.pdf]

Interviewer: So interview (SERVICE NAME). And I am with…

Respondent: (NAME)

Interviewer: (NAME)

Respondent: (NAME)

Interviewer: And (NAME), interviewer. And the time is 10:30 AM. Alright. So the first set of questions are really about generally what kind of services, what settlement services you provide here at (SERVICE NAME). And that can be anything, education, health, social, legal. So can you tell us a bit about, I guess, the services that you provide in settlement services?

Respondent: Sure. So we deliver STSS (?), which is Settlement, Transition, and Support Services. And we deliver both client services and capacity building. Yeah. So that's for clients that have been in the country for under five years. So first, they receive settlement services, initial settlement services from AIMS Australia and after they exit, they either come to us or other providers of the same service sets. So client services is directly in case management and there's two models. There's a low intensity and a medium intensity case work. And the capacity building is around building communities and individual's leadership and governance knowledge. Yeah.

Interviewer: What do you mean governance knowledge?

Respondent: As in understanding how did they become an entity of themselves. Like how do they register as a group, as an organisation, and how do they understand the legalities of that. Like public liabilities, insurance, registering under, you know, NGOs and not-for-profit organisations and how do they then support the communities in, I don't know, if they want to run events, for example, how to apply for funds, how to acquire (?) funds, how to plan. Yeah. All of that.

Interviewer: So is this capacity services in terms of STSS (?) kind of a newer in the last few years?

Respondent: It is. It is, completely new. In the past it was all combined. It wasn't so prescriptive that we had to do that. Now it's more, you know, we want to build every community and make sure that they can empower their community members. So it's more about that, building their individuality in some way but also the knowledge and capacity about what's out there, what government organisations are there to help them, and how can they help them and support them. Yeah.

Interviewer: And do you see that as being… sorry, I'm touching on this a bit more because I think that's really interesting. I did see that it was kind of a

new thing and to me it relates directly to service literacy, right. Do you see it as being effective?

Respondent: Well, we're only in the beginning of this contract. So we only started in July. So we haven't, we haven't delivered that long enough to be able to see the results. But we can see that, you know, it's promising if you can impart all that knowledge into new and emerging communities, it will be positive in the future. But the question is how long will it take before we can see that positive results in communities. Yeah. Because it's not always easy to, one, engage with different community individuals and those that are perceived as leaders, their commitment to being in leadership positions could be, you know, challenged at times, whether or not they have the time available to support the communities. You know, their focus is more around becoming independent themselves, supporting their own families. So how do you then devote enough time to community? So we may educate one person and train one person and then that's no longer the community leader, and then you have to start again. So it would be a question of how can we weed (?) out those individuals and how we can impart that knowledge and then them having that impact in the rest of their community over a period of time. So it will have to be years from now that we will see the benefits of that.

Respondent: That's right. Because it is new, so therefore it's a different approach with communities and therefore working with leaders, they've got to sort of get an understanding of well, why are you here? You know, why are you here, why are you trying to help me? What's wrong with my organisation? So we've had sort of questions, people have been questioning us and so we've got to continue to build that trust. And even though we've already known them and been working with them in different ways, this is a whole new different way of, a whole new approach, and so therefore you have to again build a sort of a different relationship and a different level of trust.

Interviewer: Yeah, interesting.

Respondent: Yeah.

Interviewer: And in terms of… so it seems like you can, as you said (NAME), you can only see the effectiveness in a few years from now or whatever it may be. But do you have any other kinds of methods of evaluation for other projects that you run or even for the capacity services in a sense? And what are they?

Respondent: I guess… well, we do conduct surveys to see whether or not people are satisfied with the services they received. But also over a period of years, you know, you might be able to see, well this client arrived two years ago, where are they at now? Have they made progress in terms of their capacity to approach services themselves rather than come to us and say, I need help with this? So we can kind of see it in that direct

contact with clients whether or not there's been progress and services are effective. But in terms of the client's input, it's through their own satisfaction surveys and yeah.

Interviewer: Yeah. So you can measure the, I guess, how they find these. Do you do any, I guess, evaluation of your own practices?

Respondent: Yeah, yeah. Well, we do a whole survey once a year and across all clients just to see how, you know, what their attitude was to accessing a service, what could be improved. So and then that gets reported sort of back through the organisation. So we do that whole survey. Obviously with STSS (?) now there is a significant data collection requirement to report back to the government. So that's a real… and then… so that has to be a really strong focus of all our case workers now with that. Yeah. So and that's…

Respondent: There's some more on that periodic kind of thing. Like we tend to collect the data of what are the trends this month? What were the presenting issues? And then kind of then assess do we know enough about that or do we need PD in that area to develop our expertise? So how do we respond to that? Yeah.

Interviewer: Excellent. And can you tell us a bit about who you collaborate or if anyone to do these programmes, to do STSS (?)?

Respondent: So I mean as an organisation we really have always had a partnership focus. Like we don't see that, you know, we should do many things alone. So firstly, we auspice (?) what's called the (SERVICE NAME). Humanitarian Network, which is, yeah, which is around 40 agencies that sort of come together to look at refugee humanitarian key issues I suppose. There's a number of different working groups. So employment and training, [indistinct 7.34] housing and consumer, youth, events. What have I forgotten?

Respondent: Health and wellbeing.

Respondent: Health and wellbeing. And they're really trying to be action focused. So rather than just coming to a meeting we then look at doing… each of those working groups then looks to do a project together. So in that with some of the key organisations I suppose would be IPC Health, (NAME OF LOCATION) …

Interviewer: IPC.

Respondent: IPC Health. So they're a community health service. Yeah. (NAME OF LOCATION) Justice, so community legal centre. (NAME OF LOCATION) Council, obviously very important that we have that partnership. Where else?

Respondent: My Care.

Respondent: My Care.

Respondent: They're another provider of STSS (?).

Respondent: So another provider. So yes. So all of the job actives we try to get involved, Centrelink, so DHS Centrelink comes along. So yeah, so that's sort of a really useful network. As an organisation, yeah, then we've got a number of different partnerships with a number of youth, so from Multicultural Youth, Youth Off The Streets, White Lion. So a lot of youth partnerships. Yeah. So all the way through the organisation. We can sit here for an hour and talk about the partners.

Interviewer: And so are there any services that are needed, that you think are needed but are not available at all? And that doesn't just have to be from yourselves either, if it…

Respondent: I don't know if it's an issue of not being available but it's more around accessibility. Whether or not clients have to wait for a long period of time to receive that support or if they're equipped with the language support that they need, for example. You know, over the years we've seen, there's obviously a lot of issues with post traumatic stress disorder and just general trauma and accessing counselling services that are appropriate, it's an bit of a challenge. Yes, there's Foundation House, which focuses on torture and trauma counselling, but by the time they actually receive the service, you know, by the time they disclose to you that this is a problem, they're already raw with their experience. And then you make a referral and it might take another, I don't know, month or two before they are seen. And then, you know, in that period there's nobody supporting them with their emotions and what's going on. Trying to access other mental health services can also be a challenge when you try just through a community health centre, for example, at IPC generalist counselling. There's also waiting lists for that. If you try through your GP and get a mental healthcare plan if it's at that stage that can also take a bit of time. And once you do get through that point, is there any psychologist out there that will use translating services? Because it's not included in their budget or whatever it's planned for. So that can be a bit of a challenge.

So yes, it exists, but it's not accessible and it's not as fast as needed. Another difficulty I would say, it's in the area of support when it comes to family violence. There's a delay in the response but also the access to language support can be a bit of a challenge.

Interviewer: The things that language support…

Respondent: And crisis housing as well.

Respondent: Yeah, crisis housing. Yeah.

Respondent: Crisis housing, which again is usually from family violence to crisis housing.

Interviewer: Definitely. And so they're also seeing some limitation access to translation services, language services, if that's an issue for…?

Respondent: If it's a private counsellor, for example, they're not funded or, you know, they have to pay it out of their own pocket. So in most cases they will not access it.

Interviewer: Yeah, OK.

Respondent: It's not the same as a GP. A GP can access it but not a psychologist.

Interviewer: Not a psychologist. And so are there any services that are over-utilised?

Respondent: Well, services…

Respondent: I mean I suppose we haven't explained our model and I suppose as our models are different. But, so we have five case workers and a settlement coordinator who sort of looks after the team. All the case workers are from the communities of Burma, which is by far our largest settlement group in the city of (NAME OF LOCATION). So yeah. And so they are across the different communities. So we have Chin, Karenni, and Karen settlement workers. So they also have been really identified as leaders within their own communities. So they already have a profile within their community which makes their work somewhat easier. But also makes their lives a little bit tough because they don't switch off a lot. So our models working, has worked quite well in having that, always having that bicultural, those workers in those jobs.

Respondent: And because it is drop-in service, you know, clients just come in whenever they need any assistance. Yeah. So I think in comparison to other, other providers or just any other direct service agency, we get a very, very large number of clients presenting with issues. And in most of the cases the reason they come here, it's because they have someone who speaks first language. They can explain it a whole lot easier, they don't have to struggle or be under stress of thinking, how am I going to explain this situation to someone? So that makes it a whole lot easier for them to go, I'm going to go to someone who will understand me, understand the cultural context, and can speak to me in first language and guide me through this whole thing, the issue.

Interviewer: So do you mean coming here instead of going to the universal services like Centrelink or whatever it may be?

Respondent: Coming here. Yeah.

Interviewer: They will always opt for having (NAME OF LOCATION) as the go between?

Respondent: That's right, yeah. So a lot of the time, yeah, if they ever get a government letter, like they will generally come here and like get an explanation of what is this letter rather than take it to the government themselves. Yeah. So just an example. But yeah, so yeah, they feel there's a sense of, yeah, the community leaders, the case workers are well-known. So yes, and they can speak in first language. So we don't need to use… very rarely will we use translator services.

Interviewer: Yeah, excellent. And so when you say that…

Respondent: Or interpreter services.

Interviewer: Settlement services are over-utilised, you suggest that you could use more case workers? Like you've got greater people requiring access to case work settlement services than what you can, what you have the capacity to do?

Respondent: Yeah, yeah. And then we've got people coming in that are outside of the five years but still wanting service as well.

Interviewer: Excellent. Alright, so the next questions are related to how migrants are adjusting to Australian culture and society and what kind of challenges they may face in that adjustment. So could you tell us a little bit about your understanding of how the migrants that you work with specifically understand Australian culture and society, to what extent they understand?

Respondent: OK. Well, that would kind of depend on if we think, who we're talking about. If it's older migrants or if it's younger. Obviously for kids, they adapt much quicker, they learn the language a lot faster. But I guess the challenge there is that they're torn between two cultures and two expectations of this is how you behave. You know, at home they have to comply with what is traditionally expected of their behaviour. In mainstream society, at schools, they have to kind of blend in with that community as well. So they're constantly in challenge trying to become more independent but having the pulling from family saying, you must be home at this time, where other kids are out and having fun or whatever. So it will kind of depend on that. Like if we think kids adapt much faster but then they're also caught in that challenge and ongoing conflict of their own identity, their own sense of belonging in a two-world reality. For older citizens then the challenge is more around how soon will they be able to understand and communicate in English when many of them have got literacy issues in their own first language.

For most of the clients that we work with from the communities of Burma, they've spent on average 20 to 30 years in a refugee camp

without access to a government funded education system, for example. So they would not have had access to that for the last 30 odd years. And then they come here to a metropolitan city and we say, blend in and mingle, you know, when in fact they're like 40 years behind in their ability to interact in an active society. So it kind of depends on that. It depends on how long they've been in this situation of survival mode and just, just getting by versus trying to then work here, learn the language, and become independent. That can have a serious impact on their mental health, the pressure of having to conform and behave a certain way when they haven't had enough time to transition into it. It's like we immediately say, learn English, look for employment, these are your conditions, if you don't do this you lose your income. Just the thought of having to pay for bills, you know, now it affects their day-to-day functioning. It's like, oh my God, now I've got financial responsibilities, I've got obligations with government entities, and they're completely overwhelmed. So you know, yeah. I don't know what else I can say. I can talk for an hour about this. Yeah.

Interviewer: Yeah. But clearly a lot of cultural expectations, language is a barrier.

Respondent: Language, yes. Yeah, obviously language is the barrier to, you know, to any sort of social cohesion or employment or any participation, community participation. So… and the ability to learn language, as (NAME) said, is, yeah, very different to compared to whether they're younger or older and the level of education they've had in their own country. So yes.

Interviewer: It's been a common thing so far that language is an issue but also perhaps the services for helping people obtain those language skills perhaps aren't up to scratch.

Respondent: Well, we are an AMEP provider. So…

Interviewer: And so how is that? I mean actually I will scratch that because that's a question later in the thing. I ask specifically about literacy.

Respondent: Alright. Yeah, I can talk about that for a long time.

Interviewer: Excellent. Alright. And so what are the… to what extent do you see that your clients have the capacity to practice their own culture once here as well?

Respondent: Well, we try our best to make sure that that is still supported. As we were walking through there was a loom room because that's what is really important to the communities of Burma, it's weaving and sort of passing traditional knowledge and passions from different ethnic groups through their weaving. But we do encourage that a lot because there's that sense of you can't forget where you come from to just try and fit in. Your own identity is important and the level of contribution that that culture has in the broader society, it's really positive. We

focus at least, you know, when it's Cultural Diversity Week to really celebrate that and encourage that, but also on a daily basis for us here. You know, we always encourage our clients to wear their traditional costumes and to, you know, celebrate that, that it's beautiful, that it is, you know, art. It's, you know, just embrace it and celebrate it.

But I guess it also has that, OK that occurs here in this setting, in this, you know, where it's welcomed. But in the broader community it might be perceived as, you know, people look differently if they're wearing their traditional costumes, they're pointed at in certain suburbs or et cetera that, you know, it might not be seen as welcoming from the broader community. So then that makes people and particularly young people try to not be seen as the migrant or not be seen as the, you know, refugee. They try to blend in way too much and forget all of that to be accepted, which then leaves kids feeling a bit, you know, like no identity. It's like they've lost their sense of identity because of it. They try too hard to blend in and be accepted but then they forget that aspect of their lives. And yeah, it can create more negativity in the future I suppose.

Interviewer: So these next questions are about migrant's sense of belonging and inclusion, which is kind of a similar question. But can you tell us about any services that you provide here or any programmes you provide here that help to encourage sense of belonging or inclusion in Australian society for new migrants?

Respondent: We run a lot of groups and… so for example, we do conversational English. We have a group of volunteers that come pretty much every day of the week to the looming and weaving programmes. But they, you know, we have a variety there or some that have been here for under five and others that have been here for much longer than five years. But we sort of thought, OK well they're contributing in their volunteering activities, they're producing beautiful materials, et cetera, but we also wanted them and they wanted to develop their conversational English. So we thought, OK well we can do that as part of the volunteering. So it's not something different but it's incorporated in the time that they come to volunteer. And they're all quite happy that they can do that and that they are definitely picking up more of the language now than we've seen before. So that's one thing, which, you know, they do something that they love but they're also learning the language in a non-traditional way of learning I suppose, it's more casual. So it helps in that way.

We run some women's support groups as well but it also has a bit of a direction of empowering women and passing knowledge about legal systems and what's available out there, how does the education system work so that they can understand how to liaise with the primary schools or secondary schools. And also just general information about, you know, what's appropriate in the wider community and what is seen as something that you shouldn't do in public, for example, or what's OK

to do. Also about, you know, lunches for kids and how to pack lunches so that the kids are not seen as too different. And you know, embrace that part of it. Then we also have a sewing group that brings two cultural groups, the Chin and Karenni together. And they actually sew traditional clothes. But it's got two benefits I suppose, the learning the skill that they can then use for employment in the future because they could do alterations for example and things like that, but they're also maintaining, they're creating traditional costumes for themselves.

Interviewer: And so does that lead to a sense of belonging or inclusion in Australian society?

Respondent: It creates that… both, I suppose. Because once they feel that, OK I can contribute in a way, I can do something that is recognised as worthy, then they feel that they're being more part of the society. But also they feel that they belong to the group, not just the broader but they can belong in a group. And I think that initially for any migrant group, before they can think that they belong to the wider society they need to feel that they have a sense of belonging within their own ethnic group, that they have a place where they're not charged or questioned or anything but they can feel that they can feel the support of each other. Yeah. So we try to do that through those groups. And there's play groups that we run as well. So again, it's bringing mums and young kids together to try and understand what's out there and how the system works. You know, what happens after a particular age, what do you do with kinder, what do you do in primary education? So it's trying to create that sense of, you're here, you belong here, these are the services available to you.

Interviewer: Yeah.

Respondent: And so yeah. And then broadly as an organisation I suppose settlement clients will be participating in sort of broader organisational activities such as, this year we've run a peace choir, I know that some settlement clients have participated in… we've had (NAME OF LOCATION) Citizen's Academy, which is like looking at an experiential learning through the justice system in Victoria. We have an interfaith network, which is again obviously faith is an incredibly strong within the communities of Burma and so they're strongly involved with that. We have a learning festival, which is showcasing, will showcase the community culture and activities that they do. We have a volunteer's week event each year, a senior's event, we run a multicultural fiesta in Diversity Week, which is again celebrating all the cultures in (NAME OF LOCATION), a lot of cultural performances at that event. And then we have a Jobs Victoria service so that works really closely with the settlement team when it's getting to the point of people looking for employment.

Respondent: And we run two camps in a year as well. We do a Youth Leadership Camp for the interfaith and we do a family strengthening camp as well.

Interviewer: Excellent. Brilliant. And so in terms… so there's a lot of obviously integration in broader activities that you do with the new migrants as well. Who do you see that your clients turn to for social and emotional support within, is it still… this is getting to I guess inclusion. Because is it still very much inward looking or are they starting to talk to say people here or to religious leaders or…?

Respondent: Yeah. Faith probably would be probably number one I'd say. Yeah. Yeah. So obviously we've got a strong Baptist community and then a Buddhist community as well. They're probably the two largest faith groups when it comes to the communities in Burma. Yeah, I mean, as we mentioned before our case workers are also seen as community leaders. So they're kind of, with that hat on people might come to them with support through that. And obviously when they're younger and then they're kind of already sort of mixing with a wider range of younger people I think young people seek support from each other. So yeah. So…

Interviewer: Yeah, excellent. So these next questions are about programmes that you run that might respond to health and wellbeing needs of new migrants. So can you tell us a bit about any of those programmes, if you have any that is?

Respondent: Yeah. Well, I think that all the ones we mentioned before, they were there to respond to a need of emotional wellbeing. There was a lot of depression because people were not engaged and were not participating. So all the programmes that we run, it's about trying to minimise social isolation and try to bring people into our centre and bring them together to prevent any of those mental health concerns.

Respondent: The seniors of the community and who are, who particularly we've set up the loom and the sewing and weaving, they were probably the… they were having real challenges with learning English and you would, like they probably… if we went back and looked at their student record I wouldn't be surprised if a number of them might have spent four or five years in an English language class but they're very challenging for them to actually learn English. So it was very important to find different ways to engage the elders so that they can feel included, feel that they're giving back. And so the loom and the weaving that they do, and it's right in the heart of our building, has really given them a really strong focus. Yeah.

Interviewer: Yeah, great.

Respondent: We do collaborate a lot with the health services, though. Like the community health centres. And when we've seen a spike of issues we try to facilitate information sessions around those topics. We've got an immunisation programme happening upstairs as well so that kids who are not up to date can come here and get their immunisation. And in

the past we've also run breast screening programmes for women that have never done a screen test before. Yeah.

Interviewer: Excellent. And are you aware of any barriers or enablers that clients experience in accessing health programmes or wellbeing programmes?

Respondent: There's a lot with mainstream health services, there's a lot of gaps in knowledge both between the mainstream services and the community. So yeah, that sort of concern. So turning up to an emergency room, being able to be able… just those sort of basic, basic things that you and I might sort of understand, there's a real gap in that area. And it's been quite, I think it's a long-term gap that hasn't been, yeah, which is only just caught my attention recently. So it's like, right, let's do something about this. So, because yeah, because there's a real need for improvement in understanding health services, whether it's pregnancy, men's health, mental health, but just basically interaction, interacting with a hospital which is pretty challenging.

Interviewer: Yeah, OK. And so it's a challenge for new migrants navigating health systems, know what's there and to know what they can use?

Respondent: Correct.

Interviewer: And that you've also said that it's a challenge for those, for the mainstream providers. What challenge did they face in offering these services?

Respondent: Yeah. The tool (?) to communicate, to communicate… and also to get across their responsibilities as health providers in regards to the messages or the information that they have to provide in regards to risks and which sometimes can be seen as, you know, the client can be, why are you telling me that I've got, you know, 20% chance of the child not being born? And it's like from the perspective of the Australian health system, well they have a responsibility to tell people those things. So yeah, there's real challenges in those understanding the rules.

Respondent: But also just comparing, you know… I'm a migrant myself. But 30 years ago, like if you had to go to a hospital appointment there wouldn't be a question of can you bring someone else who speaks your language? You would just say, I need an interpreter and an interpreter would be provided. Nowadays it tends to be, who can you come with, rather than, what language do you speak, and offer that service. So there's, yeah, there's a difference there and it's a lot harder to get an interpreter in the public health system then it used to be 30 years ago.

Interviewer: So would it be that those public health providers, whether it be a hospital or whatever it may be, would have an interpreter that worked there or they'd have someone they can call in or they'd call up TIS or if that existed then?

Respondent: They call in, I think they call in for help. They don't generally have anyone, yeah, on staff that can do any of the interpreting for them.

Interviewer: Yeah. And so your experience, (NAME), is that has, that has degraded, I guess, over the years?

Respondent: Yeah, yeah. More often than not clients are asked to bring their own interpreter. Like bring a family member that can speak the language, yeah.

Interviewer: Alright. And so the next questions are about programmes that you might run to help migrants enhance financial literacy or managing money, income generation, that kind of thing. So do you have any programmes responding to those issues?

Respondent: We've delivered some partnerships with The Good Shepard in the past. And I don't know if it's still going with the South Sudanese groups but… so it was directed and targeted at women for them to understand how to manage funds and, you know, what to do with money and how to budget better. Yeah.

Respondent: We've currently, we've just partnered with NILS, the No Interest Loan Scheme, with St Vincent de Paul. I think it's really new, that was in about three or four weeks partnership. But we're collocating their service with us.

Interviewer: Does St Vincent de Paul do the no interest loans?

Respondent: Yeah. So they do the no interest loans. We are, I mean we've done… because we are an education provider as well there are times where we have done over the last, you know, five to 10 years, literacy short courses, financial literacy short courses. So it depends on the demand and what's going on at that point in time.

Interviewer: I mean… sorry.

Respondent: Yeah, go for it.

Interviewer: Do you see any challenges or what are they, I'm sure you see challenges, what are the key challenges that clients face? Financial challenges, sorry, that clients face?

Respondent: Where do we start? I guess for some, I mean if they've come under the visas where they have to pay for their own ticket, for example, or the sponsor has applied for a loan for them to come to Australia, they land and they already have a debt that they have to repay. Then they're on Centrelink benefits, which it's an unrealistic amount anyhow, and have to pay rent because it's private rental, that's the only option available. So you know, realistically, if you come to a new country and you start

off with a debt that could be anywhere between, you know for a family of five, it could be $15,000 in debt for travel cost and medical test or whatever. You know, it's pretty unrealistic that you're on a Centrelink income and now you have to pay that back but you also have to pay your rent, your utilities, your food, your clothing, et cetera. Yeah. So it's pretty disempowering for someone that is just new to the country and, you know, they're trying to adjust and they're trying to manage their own stress and post traumatic stress to then go, how do I manage all of this?

And for many of them they just immediately go, oh I just need to get a job. But without the language what kind of work can they obtain that it's going to be, you know, they're going to make enough of a profit I suppose to cover all their expenses. So it's challenging in many ways. Yeah.

Interviewer: Yeah. Are there any… so employment seems to be an issue obviously and the low level of Centrelink. I didn't know about the debt. Is that to, can that be… is that to the federal government?

Respondent: It's… well, in… yes. I suppose it's funded by the federal government. Yeah.

Interviewer: And are there any culturally specific dynamics that impact on financial, that impact financial challenges? Doesn't make sense but you know what I mean. Anything to do with sending money home, or who's managing money, because you say you do specific programmes for women, so are there any gender dynamics that make, that encourage financial challenges or…?

Respondent: Yeah. Well, many of our clients will be sending money back home to someone. Not all of their family members are here so they fill that obligation of supporting those that are left behind, whether in a refugee camp or in the middle of where things are, you know, back home. So… and yeah, I guess the imbalance of in the past only the males were managing funds in the families. And now we have single mums in the mix that have never been responsible for finances and they need to learn about that. But also just the fact that mums in most cases will receive the bigger income because they're receiving the money for the children so the family tax benefit and it's how do we help them to manage that and understand that now you need to combine your income to be able to meet your needs of rent, utilities, and food, et cetera. Yeah.

Interviewer: Great. So the next challenge is, the next questions are about legal challenges or support provided for legal challenges. Do you have any programmes specifically responding to that?

Respondent: Well, West Justice is literally across the road. We've got a really strong relationship with them, do a number of different projects with

them every year, whether it's work place, know your workplace rights, information sessions. We've done a non name project with West Justice around sort of one of the things, the challenges for communities of Burma is that the Australian government doesn't always get their names right and so their names can be different in a number of different Australian government systems. And it becomes a real challenge for them to navigate it and to actually change your name is actually there is an expense there. So actually we tried working with West Justice to actually be able to fund a number of community members to be able to change their name, which is going to be, we're going to be rolling that out again next year. So what else have we done with legal? Yeah, I mean there isn't, there's definitely, it's a strong area of focus I suppose with (SERVICE NAME) and ourselves working together.

Respondent: It's also based on what presents to the case worker. I mean we might have a client that says, I've got a fine, what do I do about this? So that's legal information that we negotiate with (SERVICE NAME). Or if it's issues around family violence or any other matter that is legal, they'll come and present and ask for help and guidance.

Interviewer: And then you would…

Respondent: We would help them through it, help them to understand, refer them there so they can access the service.

Interviewer: OK. And do you do anything about identity, sorry, visas or inviting family to Australia or anything? So migration services here?

Respondent: No, we don't.

Respondent: No.

Respondent: There's very few services that do anything around that because it's not funded anymore.

Interviewer: Oh really. And so what kind of challenges do you see your clients face when accessing legal services?

Respondent: Language again. So how do they understand a legal system that it is so completely different to what they've known, if there was any knowledge of legal systems in the past? One is that it's a challenge to get clients to trust the legal system and to understand that OK, if you must call the police there's no threat here. The police is there to help you. Because in most cases it was the police that persecuted them, yeah. Yeah. So all of that I guess and the language. It's how do they understand legal terminology and how do we try and explain that in a simpler way?

Interviewer: So the next questions are about your client's movement, as in mobility from one place to another around Melbourne. So what are the key reasons why your clients might be moving around Melbourne?

Respondent: Affordability.

Interviewer: Affordability, right.

Respondent: One of them, yeah. Where the community is, so that they have that added support.

Respondent: Employment.

Respondent: Or employment.

Respondent: Yeah.

Interviewer: Are you seeing any trends at all of mobility for new migrants in the first few years?

Respondent: Well, there's a lot of clients moving into Melbourne from other states because of the community being here and because they hear that housing is more affordable in Victoria, which is going to change of course with all the demand. But also I guess in our support of trying to get people into employment there was a big shift moving to Bendigo and, you know, to go and work in Castlemaine and so we've kind of linked a few clients and then they, you know, the news got around, more people wanted to go there and join their friends and also go and get work there. So there was a good number moving there. Yeah. But generally it tends to be around, yeah, housing affordability, employment, and community.

Respondent: Or coming back to the community. So I mean obviously there's… government's talking about the policy of settling people in regional areas but it's very, it's very black and white in regards to what happens when people are settled in regional areas is that they will stay there for a period of time but then will sort of navigate themselves back to where their community is based. And then you see that again and again. And so then we've got, you know, we've got a case worker that was, you know, he was settled in Hobart but, you know, he's back in Melbourne. So it's just to be with the larger community easily. Just like the type of food you eat, the culture that… yeah. Where you pray and it's like all these things are kind of important that you've got access to those services so in regional communities you don't necessarily have that.

Interviewer: Yeah.

Respondent: But also the backward idea that, yes, we want people to settle in regional areas from the government but yet they fund so many little settlement services there. You know, it's like if you want people to go

there then provide them the support systems that they need to settle. Whereas in fact they reduce the amount of support given in those areas.

Respondent: Yeah. So the current contract that we're working with is interesting because there have been very… they're shared, so for example, across the western suburbs of Melbourne there's… how many providers would there be?

Respondent: There's about six or seven.

Respondent: Six or seven. And because of the way that the tender was put out you could just apply for one local government area or a broader statistical division. So it's very difficult at the moment to actually understand the level of service and the actual communities that each provider is working with. So that's, yeah, an interesting challenge. Just to give you an example, so the city of Melton talks about there's a growth in that area but it's actually quite still challenging to actually work out how many actual EFT are actually working in the city of Melbourne when it comes to settlement because it's been fragmented across so many different providers.

Interviewer: So the next question is about access to education and literacy programmes. So can you tell me about the services that are available for migrants in terms of literacy programmes and education that you run?

Respondent: So OK… so, yeah, so we are an AMEP provider. So we're probably in an unusual position at this point in time where as an organisation where we are a settlement provider and AMEP provider and have an employment service as well, which means that we're kind of, in some ways, like a bit of a very nice position to be able to have an integrated service when it comes to our clients. The AMEP programme now has been running for over two years. We were successful in the last contract previous but we've been providing the skills for education and employment programme, the SEE programme, since around I think 2006. It's quite a long time. AMEP, the new delivering model has been challenging. There was a very short period of time to implement the programme. And there's been obviously a new way of assessing the programme against the ACSF as opposed to the new curriculum.

Interviewer: What's the ACSF?

Respondent: It's the Australian Core Skills Framework. It's the way that you test progression. So around reading, writing, learning, speaking, listening are the five core skills. And you test their progression over a period of time. Yeah. It's in a local government area like (NAME OF LOCATION), which is growing so quickly, it's… yeah, running education programmes is challenging just around like actually having physical infrastructure to actually deliver it from. So actually finding classrooms can be a constant battle. We have to get creative and end

up delivering in homework, like (NAME), like things like that just because we're running out of spaces constantly. So yeah. So obviously there's the AMEP programme. We're also, we're an RTO so then we can deliver the SEE programme and then we do Skills First English language training as well. We're also a pre-accredited provider. So we can do so pre-accredited or ACFI. So as an adult education provider, so what they are is they're short-course programmes, usually 20 hours is the minimum, up to 60 hours, and they give another, different opportunity to learn English, whether it's through conversation class or pronunciation. So that's another opportunity as well that people have.

We're going… so with such a… with the AMEP programme we're constantly working on continuous improvement. And sort of we've… it's been so… it's been such a, such… the implementation, I mean two years is like… probably now we're starting to now really be able to focus on that continuous improvement as opposed to just making sure everything's stable and organised. So yeah. So I mean there is a lot of, when it comes to English language programmes, there are a considerable number of options.

Interviewer: Yeah, it seems a lot. Can you briefly just help me? So you've got the adult migration education programme, skills, education.

Respondent: SEE.

Interviewer: SEE. And then Skills First is something different again?

Respondent: Yeah. So what happens, so yeah. So obviously AMEP is the first 510 hours. Yeah. Then if you're, if you're receiving support from Centrelink you can do the Skills for Education and Employment Programme, the SEE programme, was previously the LNP programme. That's 650 hours of English language. Then following that…

Interviewer: Can you have that on top of AMEP?

Respondent: Yes, yeah. And then there's like lots of other sub programmes of AMEP as well but we won't kind of go into those. I mean one that's important is the [indistinct 50.10] programme, which is around when people are starting to get to functional English it's a chance to do work experience. So that's a good programme. And AIMS Australia previously were very good at doing that. Sort of in changing the providers I would say that everyone's just starting to get into the implementation of that programme. So anyway, AMEP, SEE, which is 650 hours. Then at the end of that if they are still requiring English language training then they can go into state government funded English language training which can be full time. And it's the same curriculum, it's through all three programmes. And so yes. So that's kind of can be a transition. Now that being said, it can… if you're a motivated person that has some educational background and is doing,

practicing their English outside of the classroom, to actually, to actually look at being functional in English if you're starting from sort of a course level it's going to take you between 1200-1500 hours. So… and that's for someone that's motivated, got sort of… are motivated and got the capability and capacity to actually learn English. For people that aren't motivated or are struggling with their capacity to come to class, then that's going to sort of again balloon out.

So what's I suppose important and sort of where we're starting to focus is looking at how we identify quickly individuals that need that… can actually probably… need a different sort of support around just going… rather than having a cookie cutter approach for everyone, through those English language programmes, how we can provide some further individual supports. So for example, if you've got a class of people that are sort of at course level of English, which is sort of a basic, very basic, basic understanding, then you will find that there will be some people perhaps from the communities of Africa that have very strong verbal skills but can be challenging with their reading and writing. Whereas the communities of Burma are probably more even across all of those skills, the reading, writing, and speaking. So they learn them all sort of at the same time. Whereas the African communities are strong in one and not so in the other. So when you've got all of those people in the one classroom, it's really hard to get to work with each of those, around each of those skills. So next year we're looking at how we split them up. So we do the full-time programme but then split them up for another short course to actually work on those core skills. So whether it's reading and writing or whether it's pronunciation or… so you know.

So that's kind of one thing we're looking at next year. But then obviously as well as that, (NAME OF LOCATION) City Council, the libraries, they run conversation classes. And then there's ethno-specific groups who will run conversation classes as well.

Interviewer: So there's really a lot of opportunities out there.

Respondent: There is.

Interviewer: But yet it seems previous to this question, language is still a big issue.

Respondent: Yeah, yeah. That's right. Yeah. Because, yeah, it is because people learn differently, people have got different educational backgrounds or no educational background. Yeah. And to take…

Respondent: And also, you know, someone who's got mental issues, post traumatic stress or just depression, their ability to retain information is going to be a challenge. They'll learn it today, they'll forget it tomorrow. So repetition is pretty essential because they just can't concentrate. Yeah.

Interviewer: And I think that's, as you said John, 1200-1500 hours for someone that's motivated, has already some formal education experience, versus people coming from, you know, not having literacy skills and mental health problems.

Respondent: The village… yeah, that's right. And…

Interviewer: It's just a long time actually to learn the language.

Respondent: Exactly, that's right. And then you think about then once you're in Australia all the other additional challenges you have in your life, getting your kids to school, just navigating, navigating, you know, navigating the community. So yeah. That motivation to learn the language can easily go because there's so many other things that they're focusing on as well.

Interviewer: Yeah. And is there, do you see any kinds of employment opportunities available for new migrants and even for migrant children as they're finishing school or university?

Respondent: Yeah. So I mean (NAME OF LOCATION) City Council has like firstly just taken a really strong focus on social and economic inclusion. So we're working quite closely with them around refugees and looking at employment opportunities. We're a Jobs Victoria provider and what's worked really well for us is that we're small enough to be able to not have siloes when it comes to education, employment, and settlement. So literally people can talk to each other and just go across, they're a desk away from each other. We've also been lucky to find some good staff when it comes to employment as well. So yeah, we're passionate about people, getting people jobs. So we've done some… I think we've been working with (NAME OF LOCATION) Farms, who need workers and who have been sort of reviewing their employment models. And so we've got a great partnership with Fresh Select, who kind of do the broccoli and cauliflower and sort of leafy vegetables for Coles. So we've got a number of people employment down with Fresh Select. Now with those people that have got that employment they still do not have functional English. So what we've then been able to do is work with Fresh Select to continue to deliver English language in the workplace.

Interviewer: Really?

Respondent: Yeah. So… because we're very, very aware that it's very, they're a very vulnerable worker if they're working somewhere and they don't have functional English. And we've seen the sort of the backend of that when it comes to the automotive industry and the Vietnamese who were working the automotive industry, 20 years of working for some of those manufacturers came out with not actually any major language skills. So we're really focusing when we're working with employers to continue to build English language so that, yeah, they're not in a

vulnerable position. We're also working with… so we've gotten a lot of people work at Don KR Castlemaine within food processing. So a number of, sort of a number of food processing, other food processing areas. So yeah, we've had a really strong focus on employment. And it's obviously, there is obviously considerable challenges. But we do a couple of community, what are the jobs out there kind of forums, so employment forums for communities, which is around the job active system and anyone that's interested in employment. We kind of then showcase some of the best practice employment that's going on. So…

Interviewer: So knowledge about what jobs are available is a challenge for people?

Respondent: Yeah. So as well, a lot of the time it's actually focusing on sort of the people that are working with communities, for them to understand what jobs are available for their clients. And so yeah, so whether it's through some of the initiatives that we've been running or some other, you know, some other guest speakers from different industries talk about their experience about employing people from refugee backgrounds, which can be really enlightening.

Interviewer: For sure. And so overall what do you think the key challenges are that migrants face that you work with in adjusting to Australian culture or settling in Australia?

Respondent: Trying to think, after everything I've said before.

Interviewer: I mean you've probably listed most of the key challenges. This is a little bit of a final wrap up kind of thing.

Respondent: I suppose, you know, discrimination still exists. Even though we're a culturally diverse community there is still pockets of negativity towards migrants and refugees. So that definitely has an impact on their wellbeing, just the general day-to-day but also feeling that they can belong when they're constantly asked, you know, where do you come from and where were you born and why are you here? And you know, on top of everything else I said before, the language, the access to services, and…

Respondent: I'd put, yeah, I mean one of the things I suppose is the key, I would say the key challenges is the assumption that they're settled within five years. Yes. Because we… we know even there's lots of research on this to show that, you know, it takes longer than five years for some communities. And so it's always a little frustrating that there's no room to move outside of those five years.

Interviewer: And finally, building on that, what would you like to see as possible solutions to helping or supporting migrants to adjust well in Australia?

Respondent: Flexibility I think is essential. It's not having a one size fits all service. Yeah. People are different, they come from different experiences. If

you work with communities of Burma and you work with communities from Iraq, their level of engaging and participating and being able to advocate for themselves is completely different. And both is to do with their experiencing their refugee journey but also what's acceptable within their culture. You know, we know that for the communities of Burma speaking up or asking for, it's a challenge because it's, you know, a community that is quite humble and they don't want to be seen as causing a problem or imposing themselves on someone else. So they don't have that level of self advocacy as you would see in other communities where speaking up and saying what they need and what they want is much more easier for them to express. So having a more flexible model of delivering services that adapts to the individual, not just to the journey but understanding their cultural context.

Interviewer: So what are some of the barriers to having this flexibility? To allowing you to provide flexible services?

Respondent: It's the limit of time. It's one thin, it's like only five years and that's it. You know, then you're done, you should be on your merry way. Yeah. But also, you know, the… well, I guess in this contract it's not so fixed in how often you can contact an individual or how often they can contact you. But in the first 12 months or 18 months there's limitations to that to the amount of level of support that they would receive when they just arrive to the country. Like how often would they see the case worker, how often would they have a community guide support, for example, someone who shows them how to get to and from… so it takes a whole lot longer for them to build that basic knowledge that by the time they come to our settlement services they're still in basic need of that. You know, how do we show them how to get to from here to the hospital, for example, if they had a specialist appointment? They would have been, had their referral made at the initial arrival.

Interviewer: In the HS (?)…

Respondent: 18 months later is when they have the appointment and they don't know how to get there so we have to help them with that process, which is not funded. We just, you know, we have to meet the need because there is a need but there is no funding for that. So yeah.

Respondent: And then we run a community support group for the South Sudanese community which is funded by the state government through the Office of Youth, which was implemented last year. And that really, really that is… I mean about 15 years after the… we're now in a sense providing some services for people who have been here for maybe 10 years. Yeah. And so for that community at the end of the five years, just the settlement wasn't actually… it wasn't… there wasn't a lot of evidence of successful settlement yet.

Interviewer: Yeah. So it seems from these… it sounds from what you said before, that funding perhaps could be an issue.

Respondent: Yeah, yeah, yeah. Some flexibility around, yeah, around funding for people outside of the five years if it's needed, if there's some evidence to show that that would benefit them but also benefit the community as well. So yeah. In the long term.

Interviewer: Alright. So this is the end of the interview. But is there any other kind of burning issues, is there anything that you want to talk about or anything you want to add that you think is important?

Respondent: Don't know if I missed anything. Well, I guess we sort of talked about refugee and migrants but we haven't spoken about asylum seekers. I don't know if they're in the scope of discussion at all.

Interviewer: Because they haven't been granted… asylum seekers don't… do they have access to settlement services? Because they haven't effectively been settled.

Respondent: Only if they are granted a temporary visa of sorts. So once they get a TPV or a SHEV, if they have complexities they could access the SRSS service, the more intensive settlement. Yeah. But the lack of any support in the meantime is the question is that once they are granted that visa they've had no support for a period of five to seven years.

Interviewer: Is that how long you could be living…

Respondent: You could be living in the community for that long, not have any support, and then now they're expected to settle for the next three years. They've had no support or guidance whatsoever for many of them. Or they've had minimum support. And… because if they were, let's say, under the SRSS funding, or ASAS and CAS previously, the limitations were that you could not work and you could only study for, I don't know, 50 hours, the language. And then they were granted work rights but they had no English skills and they were not supported onto how to look for employment. So… and then now they're, you know, those that have been granted a particular visa, they left with no services, only accessing the complexity if they're complex. But if they're not complex they're left there to navigate systems on their own because they're not eligible for settlement services. So…

Interviewer: That seems like a massive gap to me.

Respondent: Yes. Definitely. And many of them are now leaving without any SRSS support, so no income support whatsoever and still waiting. So…

Interviewer: How long do you get income support if you're on… because presumably you have to be on some kind of bridging, temporary visa to be in the community. And how long does that get income support for?

Respondent: Well, it was… it was… it had no limit before. But since they changed the policy, it was just seen as, well if you're fit enough to, you know, to look for work then you should be looking for work. And their level of assessment of vulnerable criteria was just up here. So for anyone to meet that to then be able to receive benefits was almost unobtainable. Yeah. So if you had someone with mental health issues, you say, well is it a diagnosed mental health issue, does it need medication? Whereas in most cases people have anxiety and depression but unless it's psychosis or something else then you wouldn't fit that criteria. Many people have health issues but if they thought, well you're not dying so you can still work, you may have a chronic illness but you're not on your deathbed, so no, you don't fit the criteria. So many people are now in that situation where they're vulnerable but they're not vulnerable enough in their eyes to be able to receive that support. So many of them would become homeless because they've got no income to pay for rent. Yeah.

Interviewer: Alright.

Respondent: Yeah, that was good.

Interviewer: Alright. Well, thanks very much for participating in the interview, really appreciate your time and experience in the area of course. And the interview finished at 11:38.
