# Supplementary material for: The importance of information acquisition to settlement services literacy for humanitarian migrants in Australia
Source: PLoS One. 2023 Jan 6;18(1):e0280041. doi: 10.1371/journal.pone.0280041 (PMC9821785; doi:10.1371/journal.pone.0280041)
Supplement: S1 Data — (ZIP) [file pone.0280041.s003.zip › SP_06_NSW.pdf]

Interviewer: OK, I'm here at (SERVICE NAME) with (NAME), on the (DATE) 1:30 pm. We'll start the interview. OK, so before we begin the interview questions, I just want to note for the purposes of this research study, and it's probably the same as your definition, but when we refer to newly arrived migrants, or migrants, I am referring to people who have arrived in Australia in the last five years, and it includes refugees and migrants.

Respondent: So it doesn't include asylum seekers?

Interviewer: We can talk about asylum seekers for sure, because with some of the past interviews there's been specific issues around access for asylum seekers to services. So if that's relevant, sure include that. Alright, so the first set of questions are around the services that you provide here at (SERVICE NAME). So could you start by telling me about some of the services that you provide that assist newly arrived migrants and refugees?

Respondent: Ours is more like community engagement, that's my department, is the Community Engagement Division, and I'm (NAME) Service Team Leader. And we have a couple of programs or services that we provide to our clients, who are obviously refugee and newly migrants people. That includes we do a couple of projects like SETS programs, settlement, engagement and transition support, and funded through SSI, Settlement Service International. And that has different areas. One is like find services. That includes casework, and even our casework can be divided into a couple of areas. For example, meeting for specific casework and moderating specific caseworks, things like that. And like what basically it means is those migrants who come to this program, we support them in different areas, so they can settle in the community, [inaudible, 02:40] that is our coverage area for this program. So it means if they have some kind of problems, they come to hear, and our journalistic caseworker, they take intake assessments, and find out what's their goals, and what are their needs, and try to find where they should go, and where to refer them, and other services so that they can refer and get services, something like that. Including our caseworker, also supporting for example documentation and sometimes transitions, citizenship forms, applications, filling the application sometime, they need to enrol their kids in the schools. Our caseworker helps them filling out the form. Or sometimes we mobilise our volunteers to do these documents and all the things, that's another part. And we also provide, beside adult migrant English programs, we also do English conversation classes, so that they can come here and we have more than just teachers. They speak the English language, and we encourage them to speak as well. So there are these things. And also one thing is more focus on the client, the client system. Another thing is like we have community capacity building, and that's one of the areas of the SETS services. It means we have programs especially for community leaders or community people, or you can select emerging community leaders. And we provide some sort of capacity building training to them. For example, this year we are planning for an excursion visit to Parliament House. We are also trying to give them some sort of skills, like how to manage groups and how to speak, how to advocate, something like that. That kind of like activities we are now planning. In the coming days.

Interviewer: Would that be available for people with first five years, and then also people who have been here longer as well?

Respondent: Yes, yes. Our client groups means people who are here from the very beginning to the five years.

Interviewer: Right, for everything that you're talking about?

Respondent: Yes.

Interviewer: And do you see, in your experience, is that mainly refugees or migrants, or half/half? What would you say?

Respondent: Mainly refugees.

Interviewer: Yes, because of the eligibility.

Respondent: Yes, mainly refugees, but we come to any newly arrived communities.

Interviewer: OK, great. Sorry for cutting your train of the thought there, did you want to add anything to that?

Respondent: There are a couple of programs, like we also have H.S.V. dietary programs, specialised agency here, services like the clients who have multiple problems, for example like disability and domestic violence and unemployment or physical health conditions. If they have some kind of multiple issues, and are not being able to navigate the services or having issues, we also do help in those kind of clients. Just through projects. And we are also running some small projects that helps our target group, target line. For example this year we are implementing a particular program, and we're helping our clients to make resume writing, and helping them, and teaching some skills on job skills, you know, so that they know what is the work culture, Australian work culture. So these are these activities out there, and we're also trying to match up with our unemployed clients, or you can match a job seeker to a job provider, for example like now coming this 20th of November, we're doing an employment explorative program where we are inviting job providers for example like Coles or other industries to there, and we're also inviting our clients, and they will come in the one meeting point and they will talk to each other. And if maybe they will get, some client will get some kind of job opportunities or some kind of placement that will be our outcome. So these kind of things we are doing.

Interviewer: And you sort of touched on the next question, which was around who do you collaborate with to do your work. Besides the employment agencies, are there other people that you'd like to talk about? Organisations or programs?

Respondent: It's not like a one man show. Every organisation, they need some partners to collaborate and to work. So we collaborate with council, (NAME OF LOCATION) City Council and then TAFE college, and also [inaudible, 08:42] and other, (NAME OF LOCATION) interagency, other meetings, and we have

Mission Australia, Salvation Army, others out there like SSI, and Relationships Australia.

Interviewer: That's handy, them all being in the same building too, which is great.

Respondent: Yes, for reach training. There are a couple like this, depending on the project and program, not everybody comes in, it depends on projects, what projects demand what kind of partners. And look at policing, we will look at them as well.

Interviewer: And are there any organisations that you choose not to work with, for any particular reason?

Respondent: No, nothing like that. I'm telling you on my own experience, I don't know overall of this thing, but I prefer to work with all, and we are in the committees of course. They have their own role.

Interviewer: And are you aware of any services that are needed, but not available?

Respondent: Yeah, I don't know whether that is available or not, maybe I have limited knowledge, maybe there are some. What I'm feeling is like now particularly when I discuss with my staff members at team meetings, all the things that I'm seeing, especially programs or services for, especially you can say age of 9 to 12.

Interviewer: Oh, those middle years. Because there's early childhood ones, and then youth ones.

Respondent: So this group are missing, and we talk about these things a couple of times, but nothing, I can't see anything happening. But I think this is really, really important to know. This group is vulnerable.

Interviewer: I know, it's such an obvious gap.

Respondent: This is a time where they have emotional scenes, they launch the meetings and they want to be independent at the same time, but a the same time they are dependent. They ask the questions to their authorities. So many things are out there, and so many physical changes are there, the emotional changes are there, it's like early teenager things. But we don't have these kind of services, especially for culturally and linguistically diverse group. They are from different backgrounds, and for them this is a new culture, Australia is a new place, and Australian culture is a new culture. Many parents, even back in their country they struggle to manage their teenage girls and boys. Back to here, culture is different, and their kids speak a different language, and it's double. The problem hits in a double way, imagine.

Interviewer: That's great.

Respondent: I think this is something we need to, I see the gap. And there are a couple of voices out there like translation services which are lacking I think for many

reasons. There are translation services, but we need to pay, and clients are not able to, our organisations are not able to help, because that is also we didn't foresee in the budget guidelines and all those things. But this is also one thing, and many documents and many information is not there in their own appropriate language, that I'm seeing.

Interviewer: That's good, they're great. Are you aware of any services that are over utilised, in terms of high demand, long waiting times?

Respondent: Yes, in a couple of months what I'm feeling, especially my client groups, they are asking for driving, something like classes or courses on driving, something like that, or driving practice, that's in a little bit of high demand, and also like citizenship classes so that they can [inaudible 13:50] So they are also in demand, but sometimes we are not able to deliver those kind of classes.

Interviewer: And what about, on the flip side of that, services that are available but are under utilised, like there's not much, a poor update?

Respondent: I don't think that way. It's different, sometimes the same service has high demand and then sometimes the same services, seems like no demand. I think sometimes it's seasonal, I spend seven or eight months working with this space and I can see sometimes we've got so many clients, and sometimes we don't have two or three clients.

Interviewer: So it fluctuates.

Respondent: But the thing is, what I see in the positive aspect is that if we have services, no matter what, how many clients there are, that's a good thing. People can come, because it's a volunteer thing, we can't force people to come and sit in these kind of services. Whenever they feel the need, they come. It's like you have in your house, whenever you feel like you go there, otherwise you don't go there. That's the services. So I feel, I see it like that really. I don't see it like it's not being used. It has utilities, it helps to have it there, for any time, whenever a client feels they need to learn, they can get that kind of service, they can come at least and get those services. What happens in the 24 hours, you hardly use five minutes of the time, it doesn't mean you [inaudible, 15:46]

Interviewer: Fair enough.

Respondent: For me, I see it like that.

Interviewer: I see what you're saying.

Respondent: Someone is needing it, even if it's not being used, there's some kind of system.

Interviewer: Thank you. Can you tell us about the methods that you use to measure the effectiveness of your services and programs.

Respondent: We use evaluation of the products or after the event. It also depends on project to project basis, like for example if you do some kind of training and skills and knowledge base, we do pre evaluation and post evaluation things. And also like if we are organising bit events like Harmony Day or Refugee Day, we also distribute feedback forms with the client, and then again we do evaluation meetings with all the stakeholders. We invite all the stakeholders or all the organising committee members, and we go through what are the areas to implement, we discuss the things, we listen, and that could be utilised for the next community event and things like that. And also client services, we put all those datas in our system, in our database, and we see how we are progressing and whether they are satisfied or not. It is required by the funding body as well, so obviously we follow that kind of guidelines.

Interviewer: Great, thank you. The next set of questions are around how migrants and refugees adjust to Australian culture and society. So could you tell us about your understanding of how migrants you work with understand Australian culture and society?

Respondent: We need to do more. That's what I can see, because many people, like many migrants, they have low English. And of course, because of low English, they are not able to navigate the services, for one thing. And another thing is they have different cultural backgrounds. They are coming from different backgrounds, and I think their sense of belonging is really back to their origin. So this is a new place, new environment and new land. So it takes time I think to accept all the things. It is very difficult to say OK, forget what's past, right. So what I'm saying, we are more focused on three areas, education, employment and English language. But what I feel is OK, these three are private area that are understood. But we need something more. I don't know what is that. But they came here with different backgrounds, we need to open that as well, that's my feeling. That might be the reason there are multiple issues or complications instilling in Australia. So sometime we feel like Australia is a very good country, they came here all those dramatic events, war or tragedies, and they are now in a peaceful, smooth country. But we don't understand why, we feel like why they are wasting their time? Why they are not moving ahead? Why they are still struggling? Everything is there, but why? And sometimes we feel like if that opportunity is for us, maybe I can work, or I can draw on, but they are not doing these things, and we blame them sometimes. But I feel like we have to understand, there is something more we need to address. Something more we need to find.

Interviewer: And there's another bullet point here, you may have already answered it, but if you can add anything to it, that would be great. To what extent do you see your clients are being exposed to Australian culture?

Respondent: Not much, not much.

Interviewer: And would that be this region specific, do you think?

Respondent: Yeah. I feel like it is [inaudible 21:26] for service provider like us, even for the government, they give them a whole range of exposure. We don't expect

that to, even like those people who are born here, who are, even they haven't got that kind of opportunity to expose themselves all over Australia. So I'm not expecting that. But what I'm saying is maybe they won't here, they start to learn the language, they have their own communities who have a link with mainstream services. Policy makers, they know gradually about the political systems, all the service systems, they know about how marriage runs, marriage as an institution, how it runs, and different kind of issues and problems, Australian issues and problems and how come these people are dealing with these issues. Gradually they learn.

Interviewer: So much to learn.

Respondent: Yeah, so much to learn, and they gradually learn and they feel like a kind of belonging or something like that, they feel like they are active in their communities. But why coming from other backgrounds, and just coming to (NAME OF LOCATION) and staying in one place, and they are there and they don't know how to speak to the neighbours. Even if they know English, they may feel hesitant. They don't know if that neighbour is friendly or not. Maybe when you go and ring the bell, they may feel offence. So that kind of barrier there. So for maybe like sometimes the road scares them, the big road, high speed vehicles. We never see that kind of thing in that country, they are amazed. We are, many clients, they are not used to these things, from using toilets, and medicine, hygiene, and we are talking about the recycled bin, and so many bins are here, maybe that isn't… small things, but we're talking about the big things. How to cook food, how to learn the rice cooker, something like that. That's a very simple thing. And of course like the SST programs, they give some kind of lesson, they speak for one year and they help them to these things. But still, I feel that is not enough. Some clients, they are very, very good navigating the services, but not all clients are so smart, so educated or so literate. I don't know, so we need to understand that thing as well.

Interviewer: Great, thank you. And what are some of the opportunities for migrants to practice their own cultural values and practices?

Respondent: Maybe I'm not able to express in a good extent. Because there are diverse range of our clients, for example we have Tamil communities and clients from Tamil backgrounds…

Interviewer: It can be general.

Respondent: …or nowadays we have Arabic speaking clients who are increasing, especially from Syria and Iraq and Afghani, and previously we have some clients from African backgrounds. Also Bhutanese clients are there, so there's a range. It also depends on how strong your community is. Sometimes, how strong and how they are willing to contribute to their own communities with that. But in my personal opinion because I'm also from a migrant background, but with a different experience, what I feel is like if we want this Australian society a little bit kind of free like that. If you want to, we can enjoy that kind of thing, but it all depends on us. There might be like limited resources and limited things, but if we try to raise our concerns in a collective way, I think there

might be some bodies or some organisations, they will listen to these things. For example, we have clients, but we also organise Diwali celebration, and sometimes like new year celebration for African, sorry Arabic speaking people. So we have some cultural groups in the organisation, so they have some kind of events on the basis of their cultural background. I feel if they are united, they can come, a migrant resource officer like us, us again help them.

Interviewer: Great, thank you. So the next question is around migrants' sense of belonging and inclusion. So you've already answered a little bit about that. Are there any programs that support migrants' sense of belonging and cultural inclusion?

Respondent: This is also like some area I think we need to improve, that's what I think. And because like what happens, there are many cultural groups, they come here because of their language and background, and I feel like they want to be in the same group. But it's more easy and they don't need to trust anything, they understand the values and those things more easily, the same kind of food, culture. It's like [inaudible 28:35] like they asset themselves to a particular background. So yeah, but if we think about in the bigger picture, promoting harmony and multiculturalism, assimilation, something like that, we need more programs. We need more programs in that area. Just organising harmony event or let's say this is a multicultural event, and we invite a different cultural group on one platform and they dance, and they have food, and the go the whole day, and they go back, it doesn't help. That's my personal feeling. That's OK, to celebrate diversity, but I don't think that promotes diversity. Because they are just there for showcasing their culture. OK, we have this kind of culture, we enjoy that kind of food. But I don't see it has that bigger impact on multiculturalism and all those things. It's like your willingness or your attitude or your behaviour, to accept diversity. So we need to work on that avenue. That's my personal feeling, we need to work on that area where everybody from different backgrounds can say we belong here, and you also belong here. You also belong to the same place, and I also belong to the same place, something like that. It's all about acceptance and respecting. But many times what happened, we say my culture is good, let's show it, you know? So I feel like that kind of activity is a performance, and it's like showing the things, showing my culture is the best. OK, there's nothing wrong, nothing wrong, everybody has some kind of pride or value in their own culture, I don't mean that. What I mean I we need to work on something where everyone can say, that culture is also our culture.

Interviewer: That's great. So the next set of questions are about programs that are responsive to social support. And health and wellbeing. We'll start with social support. Are there any particular programs that provide social support?

Respondent: Again, exactly what do you mean by social support?

Interviewer: I suppose it crosses over with your casework, caseworker what they do, in terms of connecting with, it could be around education or work, or family issues.

Respondent: That might be like, I don't know whether I'm able to answer correctly or not, because I ask questions, what do you mean by social support, but how I feel is we have multiple more than six, seven groups, social groups. Maybe that is also what you want to hear about. For example, we have Tamil support group, so it means people who belong to Tamil background, they come here and they meet on a weekly basis, and they have their own activities, and they also celebrate their cultural things, and they have excursions, and we provide them information sessions on different things, for example like Centrelink information session, and then social security sometimes, sometimes policing, it's different on what they need. Sometimes we do civic education to them, sometimes we do road safety. Sometimes we give them information on domestic violence so they understand. Sometimes we do Australian culture as well, sometimes parenting, so there are a whole range of information sessions, and they come here, they share their experience. So that is also kind of like a social support group as well. That's not only like the Tamil group, we have an Afghani group, and we have an Arabic speaking males group, only for me. And also we have a men's group, and we also run different classes, for example as I told you, we have English conversation classes, we have swimming classes, we have Arabic swimming classes. So this group, they come here and they belong. They hear things, and they go back, and knowing they are doing these kind of activities is also helping to understand Australian culture and things. So many of these things, like we are running the swimming classes only for women, and those women, they don't know how to swim back in their country and maybe they are not allowed, I don't know. And this is the first time they are swimming, and they are boosting their confidence. And also for them, this is an opportunity to come out from the home. And I think not only swimming, it's one step to empowerment, that's what I say. Because they are coming out and they are learning swimming. There are a couple of, especially for women, and I feel like there are a couple of men who don't want their wife to swim. Necessarily maybe they have different cultural or religious background, but there are a few women who came out of that kind of boundaries, and they are doing good. So for me, it's not swimming, for me it's empowerment.

Interviewer: That's great.

Respondent: This is empowering to Australian culture. Like sense of freedom. And that's how I see it. We need that kind of activities more. I don't think it's only just swimming is for swimming, or swimming is for health, or swimming is just for living, activities. But this is something about independence. This is more about empowerment, that's how I interpret. These are the social support groups or support services. And also we provide IPA voucher, assistance in other things, if our clients are struggling to pay the bills, we support them, and we also provide a food voucher if our clients are struggling to buy something, we provide food voucher. And we also provide sometimes Telstra voucher, or water bill, we help support them. That is also the support, and sometimes if the clients come with issues in housing, if they have issues in housing, we have a housing officer, we provide them services. Not only for our only target group, but the overall. And also we have domestic violence prevention officer, if there is a case of this kind of abuse or domestic violence case, we can refer to

them, to her, and she will carry on, and implement services, a little bit there. And if they have some issues about immigration issues or visa problems, we refer them to legal aid. So there a couple of services within this organisation. So these are like at individual level we support in this way, and in the group level, we organise groups like I've already told you.

Interviewer: That's great, and you've sort of already answered the next question.

Respondent: Also we have a yoga class.

Interviewer: Which one?

Respondent: Yoga.

Interviewer: Yoga, excellent. The next set of questions are around financial literacy. So have you got any programs around financial literacy, or income generation or management, income management?

Respondent: This is also something we need to do more. We don't have specific title on that area, but what we have been doing is like, as I have told you, we have groups. So as a part of group activities, we run different kind of sessions, and yes we do sessions on budgeting and all those things. And sometimes we also give them sessions like how to save money, because they [inaudible, 38:40] like how to switch from the bills, something like that. Even also to save for the bills. How to like applying for a concession card, something like that. They are, we provide those information, and sometimes our guest workers sit with them and ask them to make household budgeting, they used to do that thing. But this is our kind of regular activities. But I think we need to do specific programs that cover those areas.

Interviewer: And are you aware of…

Respondent: We have something.

Interviewer: And are you aware of any financial challenges due to culturally specific dynamics? For example sending money home, or the gender, challenges around gender and money management, that sort of thing?

Respondent: Yeah, they obviously, for example sometimes clients might feel like they'd like to send money back to their country. There's also challenges, that is the reason for distress and sometimes mental health issues as well. If you talk about health, it's one of the different areas. Again, I don't know if you have those questions coming up or not. And gender, that is also the challenge, especially gender equality. Previously I talked about, I have seen a couple of clients who don't want their wife or their partner to go and do swimming. I don't know why, I didn't ask that question to them, but I feel like maybe because of cultural things. Because he asked whether there is any swimming classes for him, so it doesn't make sense. He wants to learn swimming, but he's not allowing…

Interviewer: He doesn't want his wife to learn.

Respondent: No, and that doesn't make sense. Maybe I'm biased, that's the thing. And also like gender issues there, and sometimes especially men, they feel like they are losing some kind of authority. Especially in money management, decision making in the household. Sometimes the male clients ask, what is our rights? They feel like they are losing their rights, and scenes are set in gender roles, if you're talking the core communities who are from different backgrounds, maybe in their home the wife used to stay at home, and they used to go work, and they are the breadwinner. And now here, it's switched, nobody is working sometimes, or sometimes the wife will get a job, and the husband has to sit in the home, or look after their children, babysitting. So they don't make friends, and they don't know how to cook and how to make food properly. Those kind of things are there. And domestic violence because of that. The rate of domestic violence is increasing and the report is also says that. So yeah, this is all because of they sit in their homes, and losing authority, and alcoholism, mental health, so many things that are associated with that. Another thing is like, especially in communities like marriage as an institution is very strong, and especially women, they get as a one time contract. Once married, there is no question of divorce or anything like that. Or once married, they feel like they are bound to live with him or her, that is strong. This is good, I don't see that that's bad, but what I see is [inaudible 43:20] is not good at all. So many times, many women and sometimes men also, they continue their life in [inaudible] environment which might be OK back in the country, but here it's not OK, and many people, they don't know these things.

Interviewer: Wow, that's good insight, thank you. So the next set of questions are around the support the clients get when they face legal challenges. So can you tell us about any of the programs that are available with legal issues around identity, visa, inviting families…

Respondent: I think I already mentioned this, we refer to legal aid. And maybe we also offer (SERVCIE NAME) services by our caseworker, so this is one aspect. But it is more intrinsic to the legal team, we refer them to legal aid. And they come weekly, and we see the clients. We take appointments with them.

Interviewer: Great. And what do you think the key laws or provisions are that migrants need to learn when they first arrive in Australia?

Respondent: The key things they should learn. Of course the first thing they should learn, I don't know how realistic, first the law. Australian law.

Interviewer: Australian law, just in general or anything specific?

Respondent: We should begin with the very basic one, for example like a family, what happens if you beat your husband or if you beat your wife? What happens if you beat your son or daughter in the name of disciplining? That's the very starting point, law means I'm not talking about beating, even though I still need to learn. Even the law, I need to update every time, but I'm not a legal

person. We should know the minimum thing. There are different types of law, legal law and then there is social law.

Interviewer: Good distinction.

Respondent: Maybe legal laws in the later days, but they need to learn social law, cultural law, something like that. For example, many times you go out and if you sneeze, you say sorry, you know, right? If you burp, you say sorry or excuse me. I don't know, I don't know if this is social law or cultural, but this is something we have to understand this. And if you ask questions, you provide the answer, and you say thank you. So it's establishing from the beginning…

Interviewer: It's like social etiquette.

Respondent: Social law, not only etiquette is there.

Interviewer: It's very particular, isn't it?

Respondent: What kind of dress suit we wear. Of course, it is good to wear cultural dress, but sometimes wearing cultural dress might not be productive. We need to understand, to tell them these things, right, OK. So I will not be well received sometimes, maybe because of our behaviour, maybe because of our attitude. Many times our attitude and behaviour is not welcoming because we are off the system. We're not off in other things. So that's the basic things, even back to many communities, for example what happened, they used some kind of language or if we translate it in English, it's very terrible. It's like offensive. If you go to, if somebody is listening to this thing and translate it and complain, it's important, maybe he'll refine or maybe he'll be arrested. That kind of language out there is spoken, but in the culture…

Interviewer: So it's taken out of context.

Respondent: And that's well accepted, and that has no hard meanings something like that. Those kind of things should be identified and tell them, OK, this is the thing you might have been practicing, but this might be a problem for you.

Interviewer: Great, good examples.

Respondent: Last time we discussed about my staff member, and he said in our culture, in this culture, when we say I love you very, very much or something like that, to express sometimes we use, if you translate it, it's like a killing, you know. I will kill you. What happen if I say to my five year old daughter or five year old girls, and the neighbours, who just understand English and translate it, I kill you, and he will call the police and protection officers would come and take those kids away with him.

Interviewer: Good example.

Respondent: That happens, that example is there. I can't give the good example, but that is an example, and because of that he has to suffer and he has to live separately with the kids. So there are so many of those examples.

Interviewer: They're good points. So in your opinion, what's the level of awareness of migrants to accessing key legal services? Are they very aware?

Respondent: No, I don't think they are very aware.

Interviewer: So they rely on service workers. How do they get their information, do you think?

Respondent: For us, we refer them to legal aid, that's one thing. And a couple of organisations, they organise some kind of sessions on law, immigration law and traffic laws and rules, all kind of laws. But they are, sometimes we invite them or sometimes we send our clients to them. But that is like obviously if they have some kind of problem or conflict with the law, of course we send them to the legal aid.

Interviewer: Great. So the next questions relate to the movement of clients from one place to another. So what do you think the key reasons for the movement of people from one place to another, or one suburb to another? And do you think it happens in the first few years, or after five years?

Respondent: I think this happens within five years, because after five years, you're settled, for me, and very few clients are still there who are not settled. You can see those kinds may need some additional support. We believe that after five years, they will settle somewhere. So what I'm seeing is the main thing they move from one place to another is like job. Job opportunity, or you can say income, in other language. The other is like their either relatives or friends, or their group, community group. They want to interact with their own cultural background, something like that. Without them, people feel isolated, sense of isolation. Again, I'm coming to hear, they feel a sense of belonging to their own culture, their own food, their own language, and that makes them to come in the group, and they speak their own language again. They've lost themselves in the group and they're not able to see the mainstream culture, and that is also a challenge. That's why I'm telling, we need to do many things to work on that sense of belonging. Just organising Harmony Day or something it doesn't help, we need to work on it. I don't know what or how. So that's one of the reasons. One is job obviously, job opportunities and services. In the city there are more services, more job opportunities. Good schools and all those things. So of course sometimes the Government or policy makers didn't design to put [inaudible, 53:20] in some countryside. This seems good. But after two years, or after one year, what happens, they come back and they never get their own people, because their own people have lived and practiced that, you know?

Interviewer: That's right.

Respondent: The fact is they need the people, they need help, and no other country groups has the particular communal system. The support system is there. And also if they go there, they have some clothes needs, dental fillings, this is something they advise them, and sometimes they may be able to find some job opportunities.

Interviewer: Great, that's good. So the next questions are about migrants' access to education and literacy programs. Can you tell us about some of the available programs to migrants using your service, in terms of school education for their children, adult literacy programs or any other education or literacy programs.

Respondent: Probably three things I can explain here. One is we used to, and we are also doing one like homework support program for our children. But not in my department, but there is one youth department is there with us and they look after these things, and they provide their own homework support, and have their huge support activities. They organise volleyball or different kind of sports activities. And they do squash and other things. So they do programs of huge other things there. And for as I already told you, we have English conversation class in two locations, one is (NAME OF LOCATION) and one is [inaudible, 55:34] every week. So community people who want to enhance their English conversation, they can come and participate in that group. We welcome all, it's not like only for our target group, it's under five years, it's open to all. So that's another thing. Another thing is we also encourage our clients to enrol in this adult migrant program. So these are like in different ways we are helping in the literacy and other things.

Interviewer: And are you aware of any of the key issues or barriers for children of your clients, to accessing school or university? What are some of the issues for the children?

Respondent: Right now I'm not aware. Maybe, I'm not aware.

Interviewer: That's alright. What about any employment opportunities for migrants? For their children, or for migrants in general, migrants and refugees?

Respondent: I can say something on this because currently I am working on that. Also I told you have been doing employment barter program, that's mainly targeted to our clients, under five years. And what we see is it's very, very difficult for our clients to get jobs, because of various reasons, but primary reason is they English. Because they're not able to communicate in English properly. So that's one reason, and that's also the reason this seems like a lack of confidence, because they don't know how to communicate. Obviously it will decrease your previous confidence level. So one thing is that, and another thing they don't know, work culture is another barrier. And also, even though if they have good qualifications and good English language, still there is a barrier, because of lack of words, lack of local experience. Or maybe they don't have any referees, because in the C.V. they have to put some referees names, and they may not have that kind of referee who can recommend them. They may not have local experience, like working experience in Australia. They may have experience in their country. We have, while we are doing

these activities, like employment activities, we work with TAFE college to run the job preparedness courses, like how to write C.V., how to write cover letter, and how to do interview. And we do mock interview as well. So what's the do's and don'ts, we explain all those things. And while doing this thing, we came to know there are men who have an engineering background, it's obvious, but he has very few English speaking capacity. But he knows his thing. And also like for us, some of them has PTSD as well. So they're very qualified. It doesn't mean like refugees and migrants, they can't speak English, they are dumb or they don't know anything. They are highly qualified, they know their work, but because of the lack of English and opportunity. And of course sometimes those people who are very qualified, because they lack this kind of, one of the issues is having accreditations on their certificate, or having recognition of their certificate by their home country by the Australian Government or Australian institutions. That is also a problem. If there are some doctors, like last time our clients shared with me, a doctor, they came here, but because their certificate has not been recognised, they did not get licensed. So now they are working in the cleaning industries. So what we are talking is like maybe the rest of the people, they are highly qualified, back to their country. They have all those skills. Maybe they have an English barrier, I don't know, but having sat with them, they told me their story, and what are we thinking, that maybe the Australian Government should recognise their capacity. And OK, if they are not allowed to practice in the same level, doctor level, maybe it's better for them to work along with the doctor. At least in the medical field. And give them a bit of time, maybe two years or three years, and give them a little bit of time. I don't know how to express it…

Interviewer: Like G.P. training.

Respondent: On the job training, traineeship, something like that. Maybe our government should do this kind of thing. There are many, many engineers, many, many doctors, and many, many P.G. holders. Maybe we should encourage them to stay in their own profession. Just help them. Maybe it will take them the five years, but at least they are in the right pathway.

Interviewer: And not cleaning.

Respondent: Not cleaning.

Interviewer: That's right. That's great, they're great points. So overall, we're getting to the end, which is good. Overall, what do you think are the key challenges migrants you work with face while adjusting to Australian culture, and settling in Australia? Just generally, what do you think are the key ones?

Respondent: One is like maybe sometimes, I don't know, they might be always afraid. Fear of being discriminated or having a racist remark. That might be a reason people are not developing a sense of belongingness. One thing that Australian culture is also challenging for them, and again, many families, they don't know how to explain these kind of changes in their life and the reason behind leaving their countries to their children. That is also a challenge to them. They

don't know how to raise their children in the Australian context. That are the challenges, to me. And getting a job.

Interviewer: They're great points, thank you. So finally, what would you like to see as possible solutions to helping or supporting migrants to adjust well to Australian life?

Respondent: What we are doing is good, but not enough. As a human being, education and employment and human rights. Human rights in the basic rights, every individual to get a job, they deserve it and they should be given these type of things. These basic things, especially when we talk about immigrants or migrants, or refugee. We need to work on, in the area of, and maybe it's the time, we need to work on some area of identity or some area of belonging. Even in Australia, I talk to my wife and I really, I talk to my wife about one, I don't know the person, but I think I attended one citizenship ceremony, maybe in (NAME OF LOCATION), and I'm really impressed by people of Australia, Aboriginal people. I know there are so many issues in that space as well. And I know there might be some kind of stress or tension, like because there are so many people coming in this land and they are settling here. And all the forests, all the land has been captured or engrossed, and different kind of rules can be used. In that perspective, if you see that perspective. When I attended that ceremony, I felt so, I can't express the feeling that I had, but what he said was…

Interviewer: Is this the Aboriginal person?

Respondent: …yes, the Aboriginal person. But what he was, we respect them as the original owners. Because they struggle so hard, it's been a couple of tickets, they get that kind of entitlement, and that's good. But what he said, he said we have been here for thousands of years, looking after this land, something like that. Maybe I'm not able to explain this the same way. But his meaning is we have been looking after this land. And how you came here, and you became a citizen of this land, right. Now it's your responsibility to look after this land. I felt amazed. I didn't know how a person can say like that, you know. Such beautiful words. I really admire, despite they are having like different issues, right. They are still struggling with government, there are still the government do so many things to get this services to them, the disability rate is high and the crime rate is high in the different areas. And so many people are still living in the same kind of condition, and so many alcohol problems there, ice problems, so many issues out there in this area. But this gentleman, he came and he said OK, and I thought, wow. I don't think really all people who are migrated have that kind of feeling or not.

Interviewer: It's very generous, isn't it?

Respondent: I don't know whether we are able to understand his words or not. How many of us who are receiving that certificate understand that particular word that he has been saying? I wish myself. And we fight for our own land, and we fight for our own identity, and we don't tolerate other people. And here, he is the first people who says…

Interviewer: Do you think more connection with Aboriginal culture and history of colonisation of the English coming to Australia, do you think that would help migrants understand?

Respondent: I don't know, that's what I'm saying, I don't know what is that. Maybe you guys are the ones if that's a research area. Or I can do myself research, that's one of the things. But what I mean is like go to the Aboriginal people, the person that said this beautiful word. What does it mean? It means, what is his history is now your history, whether you accept it or not. That's a different thing. What he means is his ancestors has been saving or protecting or nurturing this land. Now you are a citizen, you are not a traveller, you are not a tourist, right. You are not here to see and look, this is a beautiful harbour bridge, let's go and take a selfie. No, you are not here, you are as responsible as I am to look after this land. And what are his sorrows, his problems, his suffering, his happiness, is now yours. That means. And all the interest that has been done, is yours. And all the Australian Government, all the systems, now it's yours. His meaning is that, but how many people in that room is able to understand these things? So my vision is we need to work on that more so we feel a sense of belonging, otherwise we don't feel a sense of belonging.

Interviewer: That's excellent.

Respondent: Otherwise, these are just Aboriginal people, and we are different. And those people who get citizenship, they get back, they're on the YouTube and they listen to the news, that is from his background country. They even don't know a bushfire is happening in Australia. They might be more worried about this country or more worried about their father and mother, relatives or friends who are living there.

Interviewer: And I suppose that's a good point, in terms of individuals' ability or readiness, their own situation.

Respondent: Citizens are different. If the people who are administering Australia are also the same, but my constant is high literacy and belonging. OK, this culture is my culture. I'm looking for that day, when anybody, any core communities or migrants, OK, your culture is my culture. Then the fight of belonging starts.

Interviewer: Beautiful, that's beautiful.

Respondent: That's my feeling. If I started saying OK, your culture, what's your background, sorry. If I start to say your culture is my culture, then I belong to this land. Because this is where the [inaudible, 1:12:32] more than 185 countries represents. It's very, it's not one nation, it's multiple nations that come here and they are making one nation, right. So when is the day to come, to say every culture is my culture.

Interviewer: Excellent, thank you, that's a beautiful way to end the interview.

Respondent: Thank you.

Interviewer: Is there anything else…

Respondent: And thank you to the person who said these words, and it always resonates in my mind.

Interviewer: Do you know who it was, do you remember who it was? He was an elder probably.

Respondent: Uncle and Aunty.

Interviewer: Thank you so much for your time, I really, that's fantastic, all your input. So we'll end the interview now, at 2:44 pm. Thank you.
