# Supplementary material for: The importance of information acquisition to settlement services literacy for humanitarian migrants in Australia
Source: PLoS One. 2023 Jan 6;18(1):e0280041. doi: 10.1371/journal.pone.0280041 (PMC9821785; doi:10.1371/journal.pone.0280041)
Supplement: S1 Data — (ZIP) [file pone.0280041.s003.zip › SP_06_Victoria.pdf]

Speakers
K (NAME)
J (NAME)

[**start of digital**]

K This is (SERVICE NAME) with (NAME) and (NAME) as well. alright, so just before we go through these questions, I just want to clarify that we’re talking about specifically new migrants. So because obviously you’ve got other services here as well, community services so just the new migrants we’re talking about and that is migrants that have arrived in the last five years and it could be both forced or voluntary as well. just like are you actually doing anything for voluntary migrants or is it all forced as in refugees?

J What do you mean voluntary migration?

K Migrants or?

J No, we’re not funded to do that, not in our settlement program. Settlement is funded by the Commonwealth government and we’re clearly told that we provide services to people who arrive on refugee visas or family stream permanent residency visas from countries where English is not the first language. So I think it's quite specific.

K Excellent, alright so the first ten questions are about what services your organisation provides to new migrants and it could be like a bit more of a general rather than program specific and it can be educational, health, social, legal, any kind of service you provide. So what kind of services do you provide to migrants?

J Well our settlement services include casework and we have youth case workers as well so that’s one on one casework with either individuals or families, so we’ve got family bodies to operate. So when they are first referred to our service, we go through a comprehensive assessment and every individual member of that family to identify what their needs are. So from the casework we do a lot of life skills programs, lots of information sessions, we have children’s programs, we have a social recreational program as well, that’s in our settlement. We do over 100 information sessions a year. Our life skills programs are examples are swimming programs, to learn how to swim, they could be Saver Plus to learn about budgeting and banking in Australia. So it's quite broad and quite often as issues arise, we would identify how we might address those issues and they can arise in a number of ways, it's either through our individuals telling us what their issues are but also it can be service providers where an issue becomes apparent like for example fire safety in the bush or fishing regulations, understanding those.

K Yeah.

J But the CFA or the MFB will come to us and say look, we've got this problem, we feel that our new arrivals need to understand fire safety, can you help us organise to do information sessions and we do. Same with fisheries, a lot of the people who get swept off rocks fishing and a lot of drownings in Australia are newly arrived migrants or tourists and so we know – so that’s where they come to us with an issue and then we look at well what can we do to resolve this so we partnered up with Lifesaving Victoria to provide free swimming lessons, that’s the safety part but we also do – we've got a fishing day coming up for groups to go and learn about regulations and actually have a fish with fisheries so they can explain to them what they can and cannot do and they're going to offer everybody an annual free fishing licence.

K Oh excellent.

J So that’s the sort of way, that’s our settlement program.

K Yeah.

J As I said we have children’s programs and we have multicultural playgroups and we have homework support programs. Other programs that we have is – we have… we are funded by the Victorian State Government to support asylum seekers so we have what we call critical funds that can help them – it's not enough of course – but it's to help them with crisis payments where they require it and that program also works in a capacity building way with the existing associations in our region and so – and it also comes with flexible funding so, for example, the group is organising a soccer tournament and they ask for assistance with some of those costs so it's part of we would do that and we would train them about how they can apply for funding for themselves – so that’s a much more hands on capacity building program and it only works with refugees groups. We also have got aged care and disability service and that’s much broader. It works with anyone who identifies as having diversity so it includes obviously from culturally diverse backgrounds but it also includes people with dementia, people with homelessness, people with financial hardship, LGBTIQ communities, Aboriginal and Torres Strait Islander communities. So anyone who says I can't access services on my own because of my diversity I need help are eligible for that service and of course we have got a volunteer home visiting program for older people and we have 84 volunteers I think that speak different languages and that’s a home visiting program or residential aged care facility visiting program for isolated older people.

K And does that also include new migrants though or is it just…

J It's open to anybody in that category but they have to have – they're not really, they probably won’t be new because of the timeframe. I mean if they're, as people who have received a Commonwealth homecare package to stay

independent at home and also those who are in residential aged care. So they’ve probably been here more than five years but that was just generally what our service provides.

K Yeah alright excellent. And so you mentioned CFA and fisheries as a couple of examples but are there any other organisations who you collaborate with, other service providers for instance?

J We collaborate with a lot of organisations. We are generalist workers so our role is to inform people about the services that are out there so we do a lot of referral, we can do warm referral as well where we actually go with the client until they feel comfortable accessing the service themselves but in terms of our information sessions and our programs, we go to the services that are the experts in that area, we don’t try to be everything to everyone so our Saver Plus program is a partnership with [NAME 0.07.02] and the ANZ Bank because the ANZ Bank provide in that program if you open up an ANZ Bank account and save, once you have saved your first $500, the ANZ Bank match the other $500 so it's quite popular. People like it.

K Yeah, I can imagine.

J Yeah but it also teaches people budgeting and banking, what to look out for, it's quite a good program. If we were talking about any health issue we would get a health person to come and talk about it so you know hepatitis is quite a common topic, the elderly... in our region our family stream migrants are the largest populations are from China and they're often older so we've got grandparent playgroups for the Chinese grandparents and we also have regular information sessions so they choose what topics they're interested in. So they tell us what they want so after all of our programs, we have distribute surveys and have feedback so part of that is what other topics are you interested in, what would you like us to do and so it's what are you happy with, what weren’t you happy with, what could we do better and differently and what else would you want so all of that is standard for all our work and for every program we provide. For our casework we do six monthly random client surveys.

K Yeah OK.

J So, we get their feedback and also what else they think would be good and every year we make sure we do at least one to three consultations with a community. So, we invite them all to come along and tell us what they want.

K OK yeah…

J At least annually.

K Alright, excellent, and are there any services that you see are needed but aren't available and that could be that are needed from you that you don't have the capacity to offer or even that you just need more generally for new migrants?

J Oh absolutely because the welfare sector has… the resources have decreased significantly so the waiting times are in themselves a major barrier.

K So accessing what specifically?

J For any service that people need I mean there's enormous waiting times so for example if you need a mental health service, somebody is – and I'm not talking about severe psychosis – because people with severe psychosis end up in hospital and get the service but if you've got depression or you're struggling, trying to get you into a service that can assist you is very long.

K Yeah.

p And the same with… I mean aged care is another example and disability, the actual waiting time before you even get assist, before you even get on the waiting list if you like can be quite…

K So this is for, this is for new migrants and long waits for anyone as well? I remember you were saying for new migrants…

J They're included, they don't get…

K This is for universal services? Are there any specific settlement services that are needed perhaps?

J Well it's beyond my writ but I can't believe that the government only funded six community capacity build because [0.10.29] they've separated client services from capacity building and nobody has funding to do capacity building in this region at all and they only funded six across Australia… so I've suggested there's no community capacity building in a lot of areas and it is still a need.

K Yeah right, universal services and capacity building – and are there any services that are overutilized do you think as in they're there but being used too much?

J Why would anyone want to overuse the service? But it is our role…

K As in like you don't have the capacity like there's so much demand for but you don't have the capacity to deliver those services for everyone?

J I think… either I'm confusing something or you are.

K It could be probably…

J Because our role is not to be the prime service deliverer of all services people need, our role is very much around building people’s knowledge and ability to access the same services available to every Australian person. Now where the gaps really are is that the welfare sector, I don’t know if you’ll understand but to me it seems like I'm living more in the matrix than I ever was because it sounds good on paper all the services that are there, what they can do, how they can help but when you actually put your arm out to try and actually get in, they're not there… so if you call that a gap I do because it's so difficult to actually get them in the end, it takes such a lot of effort. So what happens to people do they give up? I think they do or they perhaps move on and they don’t need it after a while I'm not sure.

K Yeah.

J I think that’s the major change in the whole welfare sector for everybody, it's a bit like housing for example, We have a high demand to help us get affordable housing and we’re not housing providers, settlement workers, we’re not but you know what the waiting list is like for any public housing or any community housing? Virtually you’ve got to become homeless. So what are people doing? I think perhaps one area that should improve and I've noticed that they're actually putting more emphasis on settlement to do it is help find work because it's now so technology based that you do it all yourself. Our clients particularly those what are not proficient in English need much support than that. In the past we have been told that we don’t duplicate services that are funded elsewhere with the exception of say of employment the government is actually wanting us to help people with employment, so that a bit of change.

K Ok in recent years or?

J Hmm... because SETS only just started, it's a new one. You see we were called SGP before that and January this year is when SETS started and that settlement engagement and transition services, and every time they changed the name, they changed the emphasis a bit and that seems to be the biggest challenge I would say – is employment. They are very focussed now, the government on English education and employment.

K Yeah alright. The next questions relate to how migrants adjust to Australian culture and society and what kinds of issues they might be facing in doing so. So can you tell me about your understanding of how you see new migrants understanding Australian culture and society and what the level of understanding is?

J Look it's like anyone when you are in a new environment. You don’t always understand those unwritten rules that society expects of you and that can happen in so many different settings, in the workplace for example, what's expected of you in terms of that workplace culture. It can even be in a

swimming pool. People come from countries where they've never had public pools so they just swam in the river or whatever they did and then all of a sudden you come into this public place where there's all sorts of rules about what you should be wearing, how you behave in the pool, how you supervise your children around a pool, and so they need to learn all those things. Even shopping, culturally can be quite different. I often get asked to do Australian cross-cultural training. They want to know and we do and it's quite popular and we mix it with Australian, the topics around citizenship as well as Australian culture so that people can become more familiar with what's expected of them.

K Yeah.

J Even a school environment so we've got our play groups: people come from countries where you just send your kids to school. There's no expectation as a parent that you're involved in that and that you participate and help your child at home to learn which is where Australia has gone. So our play group would do like orientation to kinder and what the expectation of the kinder is. People don't know what a working bee is, fundraising, how do you do it, how do you bring a plate to share, this is all Australian. It's not necessarily from their culture and they have no idea what you're talking about. So I think there's a lot of work that needs to happen for people to feel more comfortable in that new environment and understanding that environment and why people are staring at them or being – because you know, if you go to a market, for example where it's very multicultural with new arrivals and you're used to standing in your line and waiting your turn as an Australian born person, expecting it to be fair and then all of a sudden there's people just pushing around you and you can react two ways, you can say 'they're awful people, look at the way they behave' and so you end up racist or you can understand that's culturally what they do. If they don't push in, you don't get whereas that's not the way Australia operates. It can happen, those sorts of misunderstandings I think are quite – that's what can cause people to get the wrong end of the idea. They don't understand that it's not what they think it is. I don't know, I mean the only way you can really do anything about that is bring your groups together all the time to learn about each other so that there's more respect and more understanding.

K Yes alright.

J We're not particularly funded, we're definitely not funded under settlement though. We have to apply for additional money to be able to do those sorts of programs.

K Yeah OK and do you, what are the impressions of how new migrants are able to practice their own culture?

J Look, there's no – I think it's an interesting question because one of the things I love about Melbourne is it's multicultural, there's certainly we have language schools so the communities can maintain their language with their children, we have celebratory days and the State government actually funds people to celebrate their cultural days in Victoria and that's all great. I think it becomes a little bit trickier when a tradition or cultural belief impacts on human rights and therefore that's kind of the bottom line that I view. If it's impacting on anybody's human rights then it's a tradition that you need to rethink, say for example family violence or patriarchy which is often – and it's here too – I'm not saying it's in one specific culture, but we all have to look at it. You can't just say well that's what we always have done. Being lawful, you know for the Southern Sudanese community, back home they would have multiple wives. It's against the law here. It's not a judgment, it's just a tradition that you really know you can't continue here. So I think that's where it becomes tricky, it's where it's bordering on illegal or against human rights that you need to be able to address that in a different way because we have a lot of Chin refugees settling in the eastern suburbs and they are Christian and the pastors and the older communities especially firmly believe that women cannot be leaders, the Bible says so. So you have your work with that to encourage leadership because women believe it too, it's not just the men and it's interesting how the communities themselves find ways around those tricky things. So we have people become understand it, we think it's a good thing to advocate more equality, some members of the community, and I thought it was gorgeous what they did for the last election for their church, three women were put up as potential candidates for the committee and the secretary was getting calls from lots of people in the community saying how could you possibly put these women up, women can't lead and he said 'oh it's nothing to do with the Bible, this is about Australian law that we should have an even committee with male and female so I'm being awful'.

K Interesting though it's not anything to do with law but it's an interesting way to phrase it.

J Yeah but it is Australian, it's not law but it's certainly something that the Australian government promotes. It's just interesting how he's put it and they didn't get voted in but those three women got 100 votes each which is 100 votes that they would never have the year before. So it's interesting to see change and so I think we need to be mindful about what we mean by promoting culture. We do, we should, there are also bigger issues that we should share, values.

K The next question is about migrants' sense of belonging and inclusion in Australian society. So can you tell us about any programs that you run here or support that help to create and enhance migrants' sense of belonging…?

J The biggest one I think that works the best is sport, it does in Victoria anyway. The sporting programs with clubs, existing clubs bring people together and anything that makes people feel like they belong is a valuable thing to do because people don't feel like they belong quite often and that's the psychology of settlement.

K New migrants don't feel that?

J No, it's the psychology of settlement really. I mean it's always people reading in promotion you've got that honeymoon period where everything is new and everyone is excited, they've got their visa, they're coming to this country, they've got their vision of what life is going to be like but then reality sets in and it's hard, it's actually quite difficult. You've got a new language, all new schools, institutions and try to find your way around with a myki even and that's when people usually turn inward and they stick with their own, they don't… and our role I think is to get them seeing goals that they can see for themselves that are achievable in getting them out of that sense of flight or fight.

K And do you run any programs, sports programs in collaboration with local clubs as in that they play?

J We do… but we often have to get additional money to be able to do it. Last year Tennis Australia gave us funding to promote tennis amongst the Chin, Burmese Chin and it was so successful that it was just extraordinary. We had 55 children and something like 21 adults learning tennis, they loved it but then of course when the program was finished, they wanted to continue with the club and the club wants them and they were ready to subsidise their membership fees but the cost of coaching in tennis was a bit prohibitive for many families, it's because of separate costs to your membership. In settlement, the sort of thing – I mean I loved this – I don't know if it's because I'm a big footy fan myself but we took 140 refugees, different community groups to a football match one year at the MCG and we had a group of Karen, a group of Chin, Hakka and a group of Southern Sudanese and we had a group of Tibetans and there were police, multicultural liaison officers that got all the tickets for free but there was a lot of staff and volunteers with them and we couldn't all sit together in one big group because we were too big and so they were moved – we were in the same section but we weren't all sitting next to each other. I've observed, it was just extraordinary because there were Sudanese children who learnt the club song of the winning team pretty quickly and they were singing it at the top of their lungs, the Tibetans were being high fived by a group of Australians that I have to say drank the whole game so we were a little bit worried about how that was going to go but by the end of it they were high fiving them. The Karen were sitting around families that explained the rules the whole time. It was – look it was the first time I actually

saw that people were totally accepted despite their race, their ethnicity whatever because of being part of a members group. They happened to be sitting in the right place for that particular team and when we were walking back to the station from the MCG, the kids were still singing the theme song and all the rest of the crowd started singing it with them, it's unbelievable. I think that's the power of sport really because for the first time ever those people felt that they were part of something bigger than… part of something Australian.

K Yeah something that they could… they had connection over that.

J They go to – through our Victorian government capacity building program, we've assisted the groups to organise festival dancing and going to Australia day parades, things like that, which they love. The other place that's also good for people to belong is religious groups when they open their doors to new congregations, so it's those sorts of, it's trickier with schools because parents don't know how to talk so they're reluctant to be a part of…

K Talk English?

J Yeah and it's so they're reluctant to become part of the school community, that's something we have to encourage them but it's about – it is hard for them.

K Yeah and speaking of religions, going to new congregations… are there… who do you see your clients turning to for social or emotional support?

J It's all based… but definitely the pastors because through our Burmese communities and they're quite diverse so when I say Chin, I'm really talking about five different language groups and the Karen and in the eastern region they're Christian mostly, majority and they split on church lines. So the Hakka Chin are the largest community but they've got eight churches already in the east and in Burma they didn't have a welfare system so if any social problem, you turn to the pastor for assistance and they've continued that here. We've been working with the pastors to try and see when there are situations where they really should be seeking professional assistance so mental health is a big issue and the pastors are happy with that because they've had a few suicides and we've got a problem with alcohol as well so the pastors want professionals involved with that as well and family violence is the other one.

K And have you worked with them at all in doing that?

J Yes, yes.

K For mental health and for?

J Yes, but they're not funded through settlement grants, they're funded through other sources. So they had a mental health project where we were training,

where we work in partnership with a mental health service and develop resources around identifying when what is a mental health issue, when does depression need help and when is it normal, if you like. Yeah so we work with them and it was a 12-month program which developed resources into these information sessions about how to identify mental health. So we're doing one at the moment on family relationships with our psychologist who pastors have asked - we do a family violence prevention program with couples and the pastors have asked for the resources from that program so they can use it for themselves when they're counselling couples in conflict, so that's a great movement really, we're also happy for that.

K And then you do… this is actually the next question is about health and wellbeing. Aside from that are you running any other programs for health and wellbeing?

J Health and wellbeing yes, as I said earlier with the older Chinese they choose the health topics they want, they're quite varied and most of their sessions are around health with the exception probably of Centrelink, that's what they want and again we have to get additional money and at the moment we've got new Arabic speaking community moving into the east so we've got funding through the local council to provide a range of health activities and introduce them to where they can access it themselves after the program is finished. So, it's things like Zumba, again they're asking for it – Pilates, swimming, gymnasium they want it… I'm not sure what age group are going to end up going to these programs but they've asked for that so we've got the funding to do that. So, health and fitness move from information about health and literacy to activity based healthy activities so we do quite a lot on healthy lunchboxes for parents and for children when they're starting school because they've asked for that. Their kids want an Australian lunch and so we do a few sessions around healthy lunchboxes.

K Cool and are you aware of any enablers or any barriers to accessing these kinds of health programs that you run?

J It's always well for our programs that we run?

K Yeah.

J Look I think there's always, it's about getting it at the right time of day so that people can come, that's always something we have to consider. We have most of our sessions after hours with the exception for our grandparents. For them it's about making sure the sessions are held in time for them to pick their grandkids up from school, things like that.

K Yeah.

J And you will never always suit everybody, that’s another thing and if we run weekend programs, we've got to be conscious of church times because they wont come if we hold it on a Sunday and then you’ve got Seven Day Adventists who wont come on a Saturday so you’ve got to know your communities and try and meet the needs of the majority so that they can attend – and bad weather – we might not get anyone.

K Yeah right.

J Even though they want it, they just won’t turn up. If anyone dies in the Burmese community which… they won’t turn up because their tradition is to go and visit and pray for the dead person. So, you're always juggling that and for us the Burmese often do the harvest so it's very difficult to get them together from October to…

K Fruit harvesting?

J Yes, in the Yarra Ranges.

K Yeah right. Alright so the next question is about programs available for migrants to enhance financial literacy or income generation or just managing money. Do you have any programs that cater for those?

J Yeah, the Saver Plus.

K The Saver Plus.

J And they ask for information sessions on superannuation, WorkCover, people ask different topics that are based towards money and saving. We did a session recently on how to buy a house… because you know amazing that many of them are buying properties after being here for five years.

K Wow.

J They're hard workers and they're quite industrious in the sense that the community groups and community members get work in particularly manufacturing industry not that that’s a growth but and because they're hard workers the employers like them and then they get their friends in. So Dollar Curtains in Ringwood I think the last I heard had 98 Chin working there.

K Yeah right, very significant.

J Yeah so they're very industrious, they're very keen to work and even though they don’t speak English, many of them around the middle age group are going to struggle because they haven’t got the… they haven’t even been educated in their own language so it's very hard to learn a new one but they do the harvests, they go around and start on the cherries I think and move to the grapes at the end.

K Yeah right…and what kind of financial challenges do you see your clients facing when settling in Australia?

J Well look the same as… I mean the biggest issue is finding a job and particularly if you have low English language proficiency, that is the biggest issue and just a reduction in what we used to call non-skilled labour. Everything is skilled now.

K Yeah and is there anything, anything that's culturally specific to new migrants you know is there any challenges they face in maybe sending money back home or…

J It used to be a big issue when we had Southern Sudanese as the main group coming through because they send a lot of money home but I haven't heard of it amongst the Chin to be honest. I'm not saying they don't do it, it's just that they never raise it.

K You aren't aware of it as a financial challenge?

J No, no.

K Any other, anything else that…?

J It seems to me with the Chin communities, they're very strongly aligned through their churches and help each other because sometimes I wonder how they're paying rent and they're eating and whatever but they seem to be able to pay their bills, very few come because they can't pay the bills. So I often wonder how they're doing it.

K Alright so maybe their financial challenges are limited at least for the Chin community anyway?

J Look I don't think you can be across the board in your research. I hope that comes through. You can't make assumptions, general assumptions about settlement applicable to all groups because they're all quite different so for example we've got in the eastern region of Melbourne, the Syrians that are coming through many of them are Armenian-Syrian, they speak four languages, they're highly educated, they don't actually need much of our support, they just need to be steered in the right direction. I mean they speak obviously Arabic, they speak Armenian, they speak English, they learnt it from school and they speak French and I was part of a women's group with them and it's the only women's group that I've been to for refugees where they were all happy to practice their English right at the beginning, they didn't have Australian English so much but they had official English and they wanted to speak it and we always provide interpreters of course but no not for that group and you could see – that group are going to do very very well very very quickly. So I don't think you can go across the board, it really depends upon

the country of origin, the culture and traditions that they've come from, their level of education, the refugee experience itself and the other thing about the Syrians here, they've got their papers to recognise their qualifications. So they're actually qualified so one of our – we employed a casual worker recently and she is a qualified teacher but to apply to become a teacher here, she has to have a higher level English at 7.5. Now her written she said is pretty good but she said her spoken is not. So I'm going to have to do other work until I get to them the 7.5 level that's required of me to get into my profession. So it's a very different problem to the say someone coming from the Karen community who's been born in a refugee camp, who's only had school within a refugee camp environment or from a Chin that have not had a refugee camp environment but haven't been able to go past grade 3 at school, it's their needs and settlement experience is quite different to someone… to another group so you can't really generalise.

K So the next question is about programs available to support your clients when they face legal challenges, now do you have any programs specifically about legal?

J Our caseworkers help them navigate the legal system. We've got referral to (NAME OF LOCATION) Community Legal Centre which is a community legal firm and we've got Victorian Legal Aid and for that matter Fair Work and whatever the issue might be whichever legal issue we might be working with, we assess whether the client needs us through that process and sometimes they do because even though they've got interpreters, the message doesn't actually get through very well. They don't quite understand the concepts that are being discussed, they don't have a term of reference for them.

K Yeah.

J So I had a client who was charged with a very serious crime and my role was very much around making sure he understood the legal process and he and his wife wanted me to come to the court which I did. He lost the first case and I talked to him about appeal because the sentence was low or it potentially could have been worse, he thought he was fine, he was happy to go through community based work but in his case he couldn't do that because it would have been automatic cancellation of his visa because of the crime and I said to him and his family you need to appeal this, you have to. You've got no choice. I don't know whether you'll be successful. It was one of the hardest talks I had really. I said but you have to try because you've lost no matter what yeah and then the decision was around should we go private or should we go with VLA and there were pros and cons of both those things but he went private and the community supported him and helped pay the fees and it was thrown out on appeal so we had a very, very positive outcome which was much to my relief

because I was even more worried because the cost of the private was so high I thought if he doesn’t win this, I mean not that it's my choice, my decision, but it just was a big worry and what did I learn out of that? If you're ever in serious trouble, go private, that’s what I learnt. Anyway, that worked out well. So it depends on the severity of it but if a client wants us to be with them to guide them through that process, then we are happy to do that. we do have info sessions about Australian law though. They all want family law.

K Yes, in relation to that, what is, what's your feeling on the new migrants’ awareness of Australian law and legal system?

J It's word of mouth and they often get it wrong. So for example, people believe that children can be removed easily in Australia and that’s not quite true. They also have I'm not sure what we define as family violence and what's acceptable and what's not and it goes around a community and quite often it takes on a folklore of it's own so for example I was told once by a group of Sudanese men that Australia had our laws upside down and I said what do you mean? They said well in our country it's men, women, children, dogs. He said in your country it's children, women, dogs and men. That’s what they believed and that went around, men have no rights in Australia.

K Yeah, that’s I think is actually very interesting. What do you suspect the reason for that is?

J Because women do have rights in Australia and…

K And they didn’t have rights it was that…

J Southern Sudan has none and they have rights here and the women use it here because it's the first time they’ve ever had the opportunity.

K Fascinating. So these questions relate to how your clients move from one place to another, some may believe, what are some of the key…

J Just hold one moment. [Interruption while talking on the telephone to someone else].

K And so yeah about mobility. What are some of the key reasons why your clients would move from one suburb to another or move around Melbourne?

J Usually it's to be with other members of their community. So they might start off in an area and then their family or friends or people they know are in a different area but people tend to like to group together within an area.

K And how about is affordability, housing affordability?

J A huge problem in the east particularly. It's not the only region where it's a problem but our region is quite big but from (NAME OF LOCATION)

Council into (NAME OF LOCATION) you can pretty much say is unaffordable for rental. So a lot of our refugee communities are settling in the Ringwood, Croydon, Kilsyth, Mooroolbark area because it's a bit cheaper but the Chin are interesting because you've got – they settle in the east and the west and very few in between.

K Yeah right.

J Their biggest communities are in the east and the west and they do talk to each other and they have national days that they share in common but it's really about being close to family and friends and the Sudanese we had had to move out, it's too expensive for them and they ended up going either southeast or the west, Werribee, Hoppers Crossing, that area because they needed big houses because of big families.

K Yeah.

J Which is a big shame because we have the best schools of Melbourne I would suggest.

K In the (NAME OF LOCATION)?

J In the (NAME OF LOCATION), yeah.

K Now the next question is about migrant's access to education literacy programs and so do you run any services to help with literacy or?

J No, we just refer them into [0.46.56] programs…the only literacy I think and it's a by product rather than a purpose built, we have multicultural playgroups and we have five interpreters at those playgroups. They are run by people like myself and we speak to them in English the whole time and interestingly enough because they started making friends across groups, we did our own evaluation some time ago and that was a by product that they had to learn English to be able to talk to each other and I think I was really pleased to see that written in there because the more you talk to people the better and my experience is that usually in a group I always talk to them like I'm talking to you and they panic first up, when they first meet me and they go looking for the bilingual worker or the interpreter, within about six months if you can have a regular group for long periods, they start to understand what you say but they're not confident in answering and usually around the 18 month mark they get the confidence to answer you. So it's a process of learning for most people.

K And so do you see, are there any issues or barriers for children of clients in accessing school or even university or anything?

J Yeah because the parents, that's why we have so many homework programs and now because we have three in primary school and three for secondary

school students close to where they live so because the parents have said to us look we can't help our kids, our English isn’t good enough they said well you do it. So that’s why we do the homework programs and we have volunteers that do one on one tutoring where a young person needs special assistance and more recently, the association are using their university students from their own community to teach other young people in their community, it's a great program and we've got two English programs started up by the associations which I think is marvellous, one that’s focussed on children, as I said university students teaching children and the other one is adults because these are adults that have got a job and realise they need English to talk to the boss and so the association set up conversational English for them.

K What's the association?

J Oh it's for that particular one it's the Australian Chin Community Association, it's a community association yeah.

K Alright and are there any – do you see employment opportunities for new migrant children as they're coming out of school and university?

J Yeah our youth workers help them apply for jobs and they get jobs and more recently there was a graduate that I knew – he was an interpreter before he finished his engineering and he came to me and asked me to help him get a job and I said to him go and volunteer at the council and see if they’ll take you and then you’ll get some work experience and see what happens, it's a big organisation, you never know. He did that now he’s employed by council as an engineer full time.

K Oh excellent.

J Australia is like that, you’ve got to… it's what I call a country of nepotism and so you’ve got to be known and of course I could give him a reference because I'd worked with him on projects as well as an interpreter because you need that too.

K Yeah definitely.

J So we do assist our people to try and get work.

K And are there employment opportunities for all the new migrants themselves, for adults?

J I don’t know the answer to that really, I haven’t seen stats. I don’t – I mean our employment caseworkers either link them into further education or help them apply for jobs and people are getting them.

K I guess that’s the question like do you see your clients getting employment or are they struggling?

J I do actually but the Southern Sudanese, no. The women yes but not the men when they were new and that’s because they were professionals at home and they didn’t want to do anything but what they did at home but the Chin will do anything so they're getting work.

K Yeah right.

J And the young people – there's always a struggle and a misfit between a young person who might be 15, 16, 17 when they arrive because they do 12 months in a language school but then they're slotted in at their age level which makes it very hard for them because they're getting towards VCE and it's too high a level, many of them had had disrupted schooling, whatever, so that group is a high need group and they – if the disengage, then you’ve got all sorts of issues around that.

K Do you have ways of overcoming that, students that have had disrupted school then having difficulties moving into school here?

J It's very difficult to tell you the truth. I mean it depends on the young person. As I said we can link them up to one on one tutors to help them try to catch up but if they’ve disengaged from school it makes it very hard because that’s when they get into trouble. Parents don’t think they’ve got any control.

K Yeah right.

J Which they don’t.

K Over kids? Parents often don’t have control over kids that much do they? If the kids don’t want them to have control.

J No, not at that age because I've known parents that have been beside themselves that their kids have disappeared, they go to the police asking for help and the police say they're 16, 17, there's nothing we can do so that’s why parents feel they have no control because they don’t.

K Hmm… and so overall, what do you think the key challenges migrants that you work with face while adjusting to life in Australia?

J I think the key challenges are English education and employment, I'm actually with the government on that.

K English education you won’t be surprised that that’s been pretty common across the other service providers as well.

J It's true, that’s the biggest because once you’ve got that covered then you can move on with your life. You can start making plans but you’ve got to have enough money to eat and live and unfortunately you’ve got to have English to get a job, it's a catch 22 most of the time with the exception of the Chin who

seem to get work but they have – because they're big workforces they've got one or two of their members that speak English that help the others.

K And have built networks that you described before anyway.

J Yes they have.

K And finally, what would you like to see as possible solutions to helping or supporting migrants to settle well in Australia?

J Look I think we provide a good service, that's my trouble and I'd like more resources to continue of course… in terms of accessing mainstream I think that technology can actually cause a barrier for many… I mean Centrelink is a good example. Have you ever tried to call them?

K Yep [laughs].

J I tell our clients when they see me, I say you're better off calling them not me. I'd wait for hours, you go through interpreter service and you won't wait as long.

K Yeah right.

J Because it costs too much to keep the interpreter waiting.

K Yes.

J But I think technology is interesting. It makes some things easier but it makes some things hard.

K Sometimes you need a person…

J I like a person, don't you?

K Yep I definitely do.

J Yes, it's nice to get a person. I think settlement services in Australia is a good model. It's more around how do we, whether we can continue to provide that, whether we will continue to be resourced to do it, I don't know any because to me settlement services are the last of the actual face to face assistance to access service. None of them are funded anymore.

K You mean none of the universal services are funded?

J No, they used to be for example, the government used to fund housing service that helped people understand their tenancy rights and responsibilities, one was for public housing and one was for hon-public housing so you could go and get advice and find out what you had to do with situations… they don't exist anymore. If you… think about it this way… if you had an elderly parent you needed to look after, where would you go, who would you ask?

K I don’t know. I’d probably do it myself. I'm one of five so we’d probably do it ourselves…

J Exactly, until it got too hard.

K Yeah.

J And then you couldn’t do it.

K Council?

J Maybe, but anyway that’s not, it's my… contact my aged care but there's that sort of generalised service which introduces people to systems and where they can go to have their needs met, that’s what we do and I think it's invaluable and I think governments are trying to take that away, that one on one and turn it into technology so that you access all these websites and learn and that’s fine, except if you can't, if you don’t know how, if you don’t have the language or the computer literacy skills, where do you go then. So I think settlement is a really important service. In fact I wonder whether they resolve a lot of issues much quicker if they even opened it up to skilled because it's not that they can't do things, sometimes they just need to be pointed in a direction and they do it themselves, would that solve some conflict issues for them in the quicker term maybe?

K Yeah.

J But it's just not seen that way and then you’ve got your international students and the institutions, the educational institutions are responsible for them, they're making huge dollars out of them but I'm not sure how much support they get but not that I think the government should be responsible for [0.58.28]. I don’t know who should be responsible, educational institutions probably but it doesn’t appear that they're doing it.

K If they're getting all the money from… I will agree with that point you made. I don’t know about that. I know in Deakin they have… and [0.58.43] all universities, I work at a couple of universities, they must… don’t have anything to do with international students, they must have specific people employed, I know they do at Deakin because I had to do a module on it myself actually when I just started but yeah I don’t know how useful the service is.

J I don’t know either.

K Alright so that’s the end of the interview. Is there anything else that you'd like to add at all?

J No, I've talked a lot.

K Thanks very much for your participation and your time.

J I look forward to seeing it.

[**end of digital**]
