# Supplementary material for: The importance of information acquisition to settlement services literacy for humanitarian migrants in Australia
Source: PLoS One. 2023 Jan 6;18(1):e0280041. doi: 10.1371/journal.pone.0280041 (PMC9821785; doi:10.1371/journal.pone.0280041)
Supplement: S1 Data — (ZIP) [file pone.0280041.s003.zip › SP_07_NSW.pdf]

Interviewer: OK, it's the (DATE), it's 10:30 am and I'm at (SERVICE NAME) with (NAME). Before we begin the interview I just want to clarify that any reference I make to newly arrived migrants, or migrants, applies to people that have arrived in Australia in the last five years or less, and it includes both refugees and migrants. OK, shall we begin?

Respondent: That's fair enough.

Interviewer: So the first set of questions are around the services you provide by your organisation that assist newly migrated people to settle in Australia. So could you start by telling us about some of the services that (SERVICE NAME) provides?

Respondent: So (SERVICE NAME) provide a range of services to migrant communities, both refugees and migrants on other streams. So the first program is the humanitarian settlement program, which supports people from when they arrive through to taking them into short term accommodation and supporting them to register with essential services, creating a bank account, Medicare, social security benefits, and also enrolling in school, whether it be in English, or primary school or secondary school for kids. Doing the initial health assessment and providing assistance to acquire long term accommodation and also to then settle in the community by linking them to community organisations and providing orientation to services. So that's the first six to 18 months, and it's based on individual needs. So that's the first program, the humanitarian settlement program.

And then down the track we have got the settlement, engagement and transition program, which is SETS, which works. So the first program, the humanitarian settlement program, works with only refugees granted visas through the humanitarian program. So there's a 200 visa subclass, 201, 202, 204, 202, and women at risk. So all of those visa subclasses are mainly humanitarian. The SETS program goes beyond humanitarian, so they also include migrant, which comes through different visas, dependent of skills visas, who lives in regional areas with limited skills, English language skills. Spouse visas are eligible for that, open visas, returnees visas, those who have left Australia and come back. And other eligible visas, based on the determination by the immigration.

So the SETS program provides a process in the initial zero to five years, and its focus on providing casework activities, linkages to local community, facilitating referral. It's a soft touch, it's not as intensive as H.S.V. It has a focus on three Es, similar to the H.S.V. program, which is English, Education and Employment. And then with the SETS program, it has two components. One stream is that it focused on client services, which is direct delivery of casework activities and group work. The second one is a community capacity building stream, which works with community groups to build their capacity to be able to commence or create a governing body. They will then go on to apply for their own grants, and set up their own initiatives. So those are some of the two programs that we have. One this side of it, in my division I have got two other programs. One is the community hubs program, which works in

primary school, with parents of kids zero to five, to provide a facilitated environment for them to understand the school requirement to prepare their kids to be ready for kindy when they start school, and to also run activities that will build the capacity of the women that attend to the hubs, to then go into jobs or into education. Some would go through a TAFE course, and from a TAFE course to a formalised education program. Or do an English course, and from an English course to a TAFE taster course, and another pathway would be created down the line. So that is another program.

[redacted]

We also have the other program, which is part of my division, is the community support program, which works with the community to engage them and canvas them about sponsoring refugees in refugee camps. Part of it is to identify people that have got the capacity to employ refugees, and part of it is to identify refugees that are capable and have those skills to be able to work in the Australian job marking, and linking those people to be able to create pathways that will ensure sustainability for people arriving from refugee camps on alternative visa pathways, rather than the humanitarian. So those are some of the things that sit in my division.

There are other programs that sit outside my division, such as employment, which had a series of employment programs. There's Parents Next, which works with parents that have left work on maternity and they need to return back to work, so preparing them to re-engage in the job market. There is a youth employment program, which provides pre-employment pathways for young people to engage with maybe mentors or industry bodies, and prepare them to be able to access job market. There is a youth pathways and preparedness program, YPPP, which works to provide youth employment support, and also engage them through our social enterprise, to provide them with work experience, through some of the initiatives that we have, whether it be in administration in warehousing, in candle making, things like that. So those programs do exist outside that.

We also have the Ability Links program, which works with people with a disability. However that is not just only for migrants and refugees, it's for the general community. People living with disability to connect them with support services or social groups, where if a person with a disability would like to engage in a particular social group, we need to provide that environment and support them to access that group. And then we have the out of home care program, which works with CALD communities to build their capacity, and also the capacity of carers, who will take on children with a cultural and linguistic diverse background. It focuses on looking at prioritising kinship placement in the first instance, and where that is not possible, to look into building the capacity, the cultural capacity of that particular carer who is from the different background from the child. So that is some of the programs that we provide.

I mean, there would be community engagement activities, where we have a large unit that works to engage the community about some of the things that they can do in supporting refugees. We've got a volunteer program where

volunteer members of the community can be enrolled into different parts of the business, whether that be to just be guides for newly arrived, or be that they actually undertake some of the social linkage directly with the client. Some volunteers run conversational English classes, to ensure that AM&P is complemented by providing that extra learning environment for new migrants to learn English. What else is relevant to refugees that we deliver? Maybe it's the arts and cultural program, which works to support the artistic skills within the CALD and newly arrived communities. So it's to look at engaging those newly arrived people, and work with them to showcase their talents through arts, be it music, be it artistic skills, drawing or dancing skills. So all we do is to support that talent.

Interviewer: Great, that's quite comprehensive.

Respondent: It is, it is. We've got suites of things. There would be other programs that I would miss out, such as the future ability which is a program that provides educational awareness to communities or people that are from cultural and linguistically diverse communities about the disability sector and the services available. Yeah, I mean that's a lot.

Interviewer: That's good. Can you tell us about some of the people that you collaborate with? Some of the organisations here?

Respondent: So in my division, I'll start with my division, the SETS program, which is the Settlement, Engagement and Transition Support program, is delivered in a consortium arrangement which SSI lead, with 20 other partners, [redacted]. Migrant resource centres, or they're formerly known as migrant resource centres, now have got a diverse range of services that they deliver. So 11 of them are migrant resource centres, 9 of them are ethno-specific organisations as well as neighbourhood centres. [redacted] and so part of the importance of that collaboration is that it brings larger organisations with smaller agencies to be able to share and learn from each other, and also be able to, given that they are embedded in their local communities, they know their communities well. So it is well aligned to the interest of their communities that we work with.

[redacted]

The 19 hubs that we currently have, we deliver it in collaboration with a range of services. Number one partnership is the school, which allowed us a space where the hubs can run their activities. Number two, because the hubs' activities are not funded, we have to then bring in support services such as early education childhood programs, we bring in. And also TAFE, we bring TAFE in for delivery. We bring a range of community groups and activities for support. We bring councils into the delivery as well. So there is a lot that happens within the community hubs environment.

While that is on the delivery side, on the strategy side, we have a collaboration with Community Hubs Australia, the national body that works to ensure the delivery of the community hubs. So along that is one of those important partnerships that we value. We also have in the humanitarian settlement program, there is a range of agencies that we collaborate with. [redacted]

Interviewer: I noticed that when I went to (SERVICE NAME). And to (SERVICE NAME)

Respondent: All of that ensures that we have that transition much more smoother.

Interviewer: And so are you co-located with all the members?

Respondent: With all the members.

Interviewer: Yeah, great.

Respondent: If I go beyond my program, there are a range of collaborators out there, and the program lead would know who they work with. But nonetheless, those are the immediate people that I would have in my work. So we have this thing called natural accountability, which is a way that we approached our work across the partnership to our partners. We collaborate and ensure that we lift our performance. We are able to deliver the required contract as well as the services that are needed. But then we go upward and downward. Downward to the communities that we work with, we are accountable to them and our work needs to speak the language that they speak. We need to be able to meet the needs of the community. And upwards are the funders, the government bodies, the Department of Home Affairs and the Department of social Services. Both departments we work with. So collaboration, we looked at it from that lateral perspective.

Interviewer: Great, thank you. So are you aware of any services that are needed but not available?

Respondent: Yeah, a lot. I can start, to be able to articulate that, I can start by identifying gaps, and that will help articulate what I'm talking about. So in the settlement services program that I lead, which is the SETS program, the community hubs and the community support program, the very first thing for the SETS is that it

is limited to people that are within five years. And I'll give you an example where a child has arrived to Australia as a 12 year old. Five years down the track, that child is a 17 year old. It's no longer eligible for services. Now he has to engage with mainstream programs. Now that child would then have significant difficulties, now that will be compounded by intergenerational conflict, the adolescent age has come in and the wonderful teenage coping with the new community, all of that starts to experience. And so it becomes difficult to support that young person outside the five years of support services. So that gap is quite big, and I would want to focus the settlement services based on the needs of a client, wherever they are in the country.

Obviously you're going to have a focus, we know that in the first three years. It is very strong, the need for settlement services. You have got the HSP program that goes up to 18 months, which is one year and six months. In that one year and six months, the learning that they got from here will assist them to kind of cope and navigate, but also to support them to be able to look at which service is still there for me, which services is still there. Five years down the track when they have kind of settled, there's nothing that they can kind of look at and say well, as a child, I learned this particular service, I need it now, I want to go and support it. So there is a gap there.

And I would want to pilot a program where we work with young people, regardless of when they arrived in the country, to be able to support them through the settlement program, based on their needs. And that would be one of the incredible things. So we don't turn people away, we don't start compartmentalising their needs. So that's one area that's needed. The other area that's needed as well, the settlement services program has not kind of looked at how do you bring employment into the settlement services? It so often focuses on employment, but it's only facilitating their access to employment, it's not actually getting them into jobs. It's just running pre-employment programs and wash your hands from there.

If you can bring employment into settlement services, there are heaps of services that will be able to support that person get employment and maintain employment, or sustain employment. It is one of the needs. It is the biggest need at the moment. Too often employment becomes kind of side tracked, and we spend our time about building the capacity here, and then we don't push them into employment as there is an employment service provider that will do that. So it creates that difficulty, OK we're running this program, where else, what else can we do? The other area is English. English currently delivered by NNP, delivered before by Namitas also. TAFE delivery, while that is all good, while that is fantastic, formalise it, there is a need for informal learning space.

What we're seeing, or what we're experiencing is that people will have different times when they are able to learn English. People will have different times when they want to work, and not learn English. 510 hours will be OK for one person, it will not be OK for another person. The delivery mode will be quite different, a young person might not need to sit in the NNP class, they might need a different service. Where this assessment can be done is in the settlement services, and so it is important that there is that share of

information, or there is a collective way to be able to provide settlement services across those domains. English or education and employment, and settlement services. If you get that, you get a long lasting settlement services program that will be able to meet the needs of newly arrived communities. There is also a need to cater to a group which are usually often have difficulty trying to articulate how best we can support them. Youth are 15 to 24. Some people will go from 14 to 25, that's fine, that group is fine. There's a group here between 13 down to 9, which have no support programs available for them. They kind of get lumped into child services.

Interviewer: Those middle years.

Respondent: So this is where things start.

Interviewer: A few people have mentioned this.

Respondent: Yeah, so this is quite an alarming issue, that there is nothing that's tailored to them here. You can have that early intervention stuff which will include 1 to 8, but there's nothing here. It is an area, I don't know why it hasn't been given attention.

Interviewer: Just historically with DOCS and FACS funding, it's always been that 0 to 8, and then 12/13 to the youth age group, and there's been that gap for many, many, many years.

Respondent: Many years, and no-one's calling it out or even thinking about listen to this and come up with something that we can deliver. So this is where I see a need really, in that respect. There is a range of things that I can say about young people. Largely because I come from a youth background, and so my previous role was youth specialist across the organisation. And so what I've seen in that is there's a lot of less attention paid to youth services in the settlement space. Given that youth work in settlement, or even across the sector, youth work is under represented, or given less attention. There seemed to be talk when we got to a kind of consortium, we go in good numbers and we talk it up, what we do with the outcome of that representation, I don't know. And for me, in at least for the case of New South Wales, we lack a service delivery approach on young people from refugee and migrant backgrounds. I would see a similar approach that's taken by Victoria, they have the Centre for Multicultural Youth, which is directly focused on service delivery area of youth. We don't have that here in New South Wales. It is the biggest gap. It is something that I would see in the future, near future if it's possible, to be able to look at a service that is providing resources to young people from migrant and refugees program. That's their sole responsibility.

Interviewer: Because the mainstream youth programs don't accommodate.

Respondent: They don't.

Interviewer: It's one size fits all.

Respondent: It's one size fits all, so that's what it needs. And I don't know why this hasn't been made, you've got multiple youth affairs network or youth advocacy network, which is a peak body.

Interviewer: MYAN?

Respondent: Yeah, it's a peak body. You have that focusing directly on policy and advocacy, but then you don't have anything that focuses on service delivery. That's the biggest gap. I would want something around that, and it would be well attuned. If I go into mental health, it's a big need in the community. I know people have talked about Headspace, even though Headspace didn't live up to…

Interviewer: That's another one size fits all. Unfortunately.

Respondent: Yeah, and then trans cultural mental health hasn't really delivered to expectation as well. And even if they do that, it's under resources and can't cater to the needs of the young people. So mental health is huge and it needs to be given attention. I would value a service around that. The final thing, I am a supporter of a service that would look at addressing what I would call the, people do have their differences in what they would look at as something that would help them. But what did help me when I arrived in Australia was the support of the community to be able to look at where do I land now in a school that has a very different approach to where I have come from? Where do I land in being able to then pick myself up, if I kind of lost myself within that school environment.

So investigating models that would work well in school to support young people, migrants, is a gap in itself. There is a focus on access, so young people can access school. There is a focus on ensuring that young people do stay at school. But there is no focus on what is it, once they experience what they go through at school, there's no focus at all in if they finish school, are they finishing with something, or are they just finishing because they have gone through their grade? And so there is nothing within the school system that supports young people to be able to pick up their grade. Something that focuses on them picking up their grade. It's usually, we're going to get you into school or uni, we're going to get you into uni. What is there at uni to support them to then continue to perform better, finish their degree, and transition into employment? Because there's no need for going to uni if you can't get a job. You would rather do other things that would get you a job, if your uni degree is going to be useless. And I can go into heaps of migrants' homes. Their degree is hanging on the wall, going and doing something different. That gap is big, and it needs to be paid attention.

There is something that we need to do go first of all understand what is the Australian job market, and this job market, is it possible to look at the needs and the population that we have in the migrant community? We have to say OK, this is where the future jobs are. This is where your studies should focus on. And try to look at within these studies, are people willing to them go through that, and support them to maintain quality of education from there.

And also you look at the barriers to accessing jobs. I mean simple things, the Mohammed name will impact on how well you access a job, or if you happen to have a John name and you arrive at an interview and you're a different person, it impacts how you get a job. So those simple things, looking at building…

Interviewer: And things that you can't prove. It's just a pattern or a feeling.

Respondent: It's an ingrained thing.

Interviewer: That's right.

Respondent: So those are some of the gaps. I hope that helps.

Interviewer: Yeah, that's great. Are you aware of any services that are over utilised, so in high demand?

Respondent: Not that I would say in high demand. But I would, some of the things that people would focus on, let's say for example settlement services now are moved on to individualised delivery. And if I looked at some of the data that comes through the work that we do, there would be things that people would need to be able to use, but have limited ability to use that. The number one things is housing support. And it's not getting outcomes, that's my difficulty with it. A lot of people go to seek housing support, you could put in 2000 applications and they're not getting through. So while it is being utilised, that service, the outcomes of it are quite difficult to achieve. And so something needs to be done about the housing issue across the nation, that would be able to then support the housing services to accommodate these demands. And you would see that people would settle in areas where they know that it will be easy to find accommodation. Otherwise if they go to the other areas, it's quite difficult to access accommodation, apart from the cultural needs that they have. But really it's about affordability and what people can access. So housing is one big area. A lot is put into it, but still it's not yielding outcomes. Another area, I don't know if it is really needed or not, but I think it's around supporting people, or under utilised maybe…

Interviewer: That's the next question, services that you think are under utilised.

Respondent: I mean legal services are under utilised, very much. And it's got, well the second one was really out of depth was the language thing, language services. So there is a lot that has changed about language services in the recent few months. The settlement services had eligibility for language support prior to January this year. But that has been taken away, and services have been asked to kind of pay for language services. If a client wants to use settlement services, and they need a particular language, the service would have to pay for it directly.

Interviewer: Interpreters?

Respondent: Yeah, interpreters. So that in itself has created both utilisation of language services and under utilisation of other agencies. So it is quite interesting to look at what has changed. Where are those people that were accessing language services, what are they doing now? And also looking at, these people that have picked up using language, in my last six or so language services that I've seen established, private language services, including a public one from the New South Wales Government language service, now if this many are opening because of that demand, what is happening? Who is monitoring that? So for me, it's to look at both ends of it. Lesser used by certain services, but arising the private language schemes that would support interpreters, translators, things like that. I think there is, I don't know, it seems fit for an academic to look into what's happening in that space.

Interviewer: I can identify a few good research projects just through our conversation, that's good, thanks. Are you, can you tell us about the methods that you use to measure the effectiveness of services you provide?

Respondent: So we do have data collection as one of the main things that we do. We found that the government system data exchange, DEX, is quite ineffective in measuring the outcomes across our partners. But none the less, they use this thing called the core data which measures satisfaction, circumstances or change in circumstances, and goals, or our outcome and our goals, if clients ever achieve those goals. And that is done pre and post. So you do a pre evaluation and once they've gone through your service, you do a post evaluation and then compare that. That's effective.

There are also activity based evaluation sheets done, so at the end of each activity there will be survey questions that are shared, and clients will tick what is relevant for them. That has a series of questions, and it depends on each agency what they wanted to do. What we have prescribed to them is the logic framework for doing that, so they are aware of the outcomes that we need, and understand what activities they need to put in place to be able to achieve those outcomes. But nonetheless they achieve those outcomes. We kind of have our own standards in the New South Wales partnership standards that we utilise, but we also use the national service framework, their standards that the government prescribe.

All of those are part of the process that we take to identify how to move toward outcomes. As I stepped into this role, we've kind of transitioned into outcome focused measurement, and so we looked at our data very cleanly and have embarked also on a data initiative program, which will allow for the collection of data through the CRMs that we have with our members. And instil that, analyse it and look at gaps, and what are the outcomes that we are achieving through that. And so yeah, a lot that we do is around looking at those things.

Interviewer: Thank you, that's great. So the next few questions are around how migrants adjust to Australian culture and society. So can you tell us about your understanding of how migrants you work with understand Australian culture and society?

Respondent: So in, there’s two ways I can answer this. There is what we do to ensure that they get that understanding, and there is also what they pick up from what we do. So the first thing that I will answer is what we do to get them to understand. So in the humanitarian settlement program, there is orientation programs which captures ten topics, and you can find the on the website from the department. Those ten topics, laws, there is transport, there is health, there is a range of them. So we do that delivery of that information and the new arrival, instil it. Too often it is too much at the very beginning, but then slowly they will pick it up and learn from that.

Social links is one of the biggest part of the orientation program, linking migrants to the communities that they lived in. So they will be able to pick up from there an initial idea about how does this community work, the social structures of the community, where can I hang out, what are the available sports, what sort of sports is there, is it health, what sort of health initiatives are there. Is it community, what sort of community organisation relates to me, things that I can look at. So that is provided in that environment. Now, what do they take from it? Every migrant, I can tell you, even the one that came before I came, do feel that at first, they want to hold on to their identity, they do not want to lose their identity, so they will be very careful in negotiating their Australian culture. So they will look at it and say OK, now that I trust this person, I have had a working relationship, I can trust them to be able to mingle with them, interchange and interact with them. I don’t yet trust this person. I will slowly kind of tread carefully until I fully trust them.

I don’t know about these sports, so foreign to me, A.F.L. for example, but down the track they may be able to understand and engage into it. Or this particular service is quite foreign to me, I don’t know how to engage with people, they look foreign to me, foods is quite strange. But a year time, they know their local community, they can engage. So those things are there.

People do tend to be cautious around this foreign culture, and they do tend to see that foreign from the very beginning, and slowly they get into it and understand that it’s a country like any other country, and that people that are engaged in Australia can keep their culture while they interact with others. And so many people do that, and as much as it is, what people find is the security and freedom that they have in Australia allows them to be able to explore those options.

Interviewer: Could you identify some of the issues and challenges around that process of cultural integration or adjustment, however you want to describe it?

Respondent: Yep. So number one is when newly arrived, comes to this country, the first thing in their mind is OK, I am going to set up my life, I am going to start my life, it’s going to be now me that is starting. But then they realise that the culture is different, so they’re going to need to learn a new language, they’re going to need to learn a new culture, and they don’t want to let go their culture as well. It’s about how they educate other people around their culture. And so culture clash becomes one of the first issues that people would have.

And so I can give an example of a time where for example I came here as a teenager and went through high school, and boarded at high school. And when I had to go back to my house for the weekend, the thing that was on my mum's mind was, am I losing my child? She wants me to be educated, but then she's worried if she's losing me through that. So her worry would always be to quiz me around cultural things, if I'm losing, who am I interacting with, am I making friends too far, do I have a girlfriend from a different culture, things like that. The fear of the other was number one. And so, it is the same thing. Cultural integration is not one way, it's reciprocal. The other party will have the same view. I had the same experiences in the community, especially boarding in a predominantly white community, northern suburbs of Sydney. It's away from the reality of western Sydney. The expectation for them would be, who is this kid? Plus your background, an African kid, all of that was quite strange. And so I had limited friends to make. So that creates social isolation.

So once you have cultural shock, cultural clash, social isolation kicks in. So it became a lot difficult for newly arrived communities to navigate their community because of that kind of I don't know you and I am keeping my boundaries. So that's the first thing.

And obviously language is a key challenge. And then the second thing would be the change in family dynamic. In some migrant communities, or mostly migrant communities, the husband is the head of the family. You come to here, the role change happens. There's more powerful women because now they are in a society that acknowledges freedom for women, which is good. Then the man, who is used to being the head of the family, the bread winner, certainly can't find a job, and maintain that bread winner. That's one thing. The parent on the other side with their kids have a difference. Now the kids quickly adapt to the community, because they now learn English very quick, they now know friends in the community, they know what to do. They can speak on behalf of their parents, they can interpret for their parents. Now the power has changed. The kids have more power than the parents. So it creates that family conflict. So the change in family dynamics is quite an issue. It breaks families most of the time. Some who don't break, it's a stressful time for them.

So it's really wonderful to be able to look at some of the programs, the response to those relationship issues at home, both for parent to parent, and parents to children. So that's another challenge. There is also the settlement aspect of it. One of the key difficulties in Australia to navigate is the Australian job market. I mean, some people come with the understanding that my niece is going to get me a job, my uncle is going to get me a job. While that is not technically what is happening in Australia, it is what is happening. You get a job from people you know. Who you know gets you into a job. Who you know gets you into uni, who you know will get you into some sort of aspiration that you want to do. So that's really number one. Finding employment or even sustainable education pathway is one challenge because of the links that they don't have. But also, a key question that is always asked by all jobs is experience. And so if you ask a refugee or a newly arrived

migrant about experience, they will tell you their experience back home. How credible that would be to an employer would be like, you don't have local experience in the Australian context. But there's no one that's giving them that local experience anyway. So it puts pressure on them, so they end up being on the lower social ladder in the community, and so cannot meet their needs to be able to engage in the community. So I can't pay for my transport to let's say a soccer game. I can't buy gear for my kids. Again, social isolation kicks in. It's one of the most difficult things.

And then the other thing is, once those things start to create social isolation and friction with the communities, the forming of the "otherness", the identity of the "otherness" starts to kick in, where people will then find sanctuary in community associations that speak their language. Further isolation. The integration chain is broken, until ten years later, this person is engaged with their community, they find I need to now engage with the rest of the community. So that process is delayed ten years. They would have achieved a lot more in that period. So those challenges…

Interviewer: That's a hard one.

Respondent: Yeah. Those challenges are there. Other than that, I think there is the usual community frictions that are created by representation of the otherness. And if I can give you an example, the impact of the African [inaudible, 50:37] coverage in Melbourne into community here. So when you look at it, that isolation again, pushed forward by media and political ambition, is then creating more rift in the community and creating that element of disengagement and the otherness. And so community representation of refugees becomes a deficit perspective, where we look at migrants, reduce them to something, label, whatever we feel. And so people now would meet with migrants knowing that they are less than the other. And where if we kind of wipe those things and say this person needs support to be able to get to the level I am in, the catching up that migrants have to do is incredible, and they still succeed. And so those are some of the key challenges. I could go on talking.

Interviewer: That's great, that's great. The next few questions are around migrants' sense of belonging and inclusion. So can you tell us about programs or supports available to help create or enhance migrants' sense of belonging and cultural inclusion?

Respondent: So there's a range of programs. I talked earlier about the humanitarian settlement program and the SETS program.

Interviewer: And the community engagement.

Respondent: And the community engagement. So there are a range of programs. I'll start with the SETS program. So the SETS program is delivered through our members, and what we do through those, we've got one, the community capacity building, which works to build the capacity of the associations, community associations, to be able to then create an opportunity to be able to

engage with the local community, to engage with council, to engage with sporting bodies, to engage with existing philanthropy to be able to then say, in our community, what would work well is this, and we can deliver it. Let's join hands and work together to be able to then support our people in the community. So we start with setting up associations, the governance structures, the project management skills, and then ensuring that we support them in the delivery of their first few projects. So that's one.

We also have innovation fund. We have it in the SETS program where we're trialling innovative community engagement activities, one of which was how do we work with disbursed communities? An initial look at it, we had communities that were settling in one area, and then they start to go into other areas. And when going into other areas, they were quite dependent on this initial place where they settled. Now it's far from them. So we created an opportunity where we work with an agency in that area to engage those community members that have gone in that area to link them with the community support services they have there. That created a lot of positive impact, where they were able to then be linked with the volunteer groups that are in that area. That volunteer will reach out to them, and support them to show them what's happening around. So those things do help.

We also have community innovation fund, where we give say $10,000 to a community group and say what would you like to deliver to a community in engaging with your community about addressing a particular topic. And so they will use that and will support them to deliver that project, to be able to then number one, talk to their community and say this is our needs, this is our aspirations. We would like to connect two or three community members to be able to then showcase what they wanted to do. It could be, we wanted to do a cultural dinner, or want to do a cultural immersion project, or we wanted to do a swimming project where they bring in local volunteers to provide swimming lessons to newly arrived communities. Those things help foster inclusion and belonging.

Community engagement has a range of things that they do, such as Surfing Without Borders, such as Community Kitchen, which is a space that brings multi stakeholders to one location in Auburn where migrants and refugees come, and in particular asylum seekers, and the community who are volunteers, come and hear what they can do for the community.

We also have a few programs such as youth programs that we do. We support a young person created his own initiative called You Belong, which is a simple movement where he would utilise the school community, do a presentation, create a key. He says this is the key to belong. He sells those keys and he uses the money to support newly arrived refugees. And so those things are there in place to support inclusion. There is also the nine priority areas in this settlement framework, which we utilise to then engage communities in those areas. We will look at philanthropic bodies and see who is willing to engage with new migrants. Allianz have got a scholarship program which they provide refugees, so it's giving them a scholarship to either attend uni, high school or TAFE, and create an opportunity there for

them to be part of the community. Those things are some of the things I could mention.

Interviewer: That's great. Now, I'm just conscious of the time. How are you going for time?

Respondent: Yeah, going.

Interviewer: How much more time could you allow for the rest of this?

Respondent: How long more?

Interviewer: I can cut it down.

Respondent: We can go until a quarter to 12.

Interviewer: That's great. Alright, so anything around programs around financial literacy, income generation? I know you've already mentioned employment issues. Anything around financial literacy?

Respondent: So the orientation program has got a specific topic on financial literacy, and we did one project that was a book that was produced, this big, I don't think it was any use. And we worked with a series of government agencies around financial literacy. We recently had a capacity building activity for our workforce around financial literacy and getting people to understand the financial literacy importance. Also as part of the AAP voucher system, each of our agencies do have a financial literacy workshop aligned to the AAP voucher. So they can attend a financial literacy workshop and then get the voucher. So those things are there. What else? I think that's about it, about income generation.

Interviewer: That's great, and any culturally specific dynamics that impact and challenge the impact of financial demands?

Respondent: Yes, yes. Both cultural, but experiential stuff as well. I'll give you the example, most migrant communities who have come from culturally connected communities, where your family extends to your uncle, great uncles, you have the responsibility of those families. So when they come here, they would have priority to give some of their money to people back home, and so that is becoming a priority. It could be a request to cover some medical needs, which they would prioritise than here. There is also issues around learning banking. Banking back home wasn't an issue for some of the communities who are pastoralists or who are more into agriculture, and so storing money in the bank was not a need back home. Or even managing money was not a need, they managed other livestock, not money. So managing money would have been one difficult thing that people will need to have the understanding. There is also a level of ownership around money, anybody over 18 years old has their own bank account. And so it's a challenge to pull together money from that aspect. So again, it creates difficulty in terms

of managing money. There's a lot of cultural issues that impact on financial literacy.

Interviewer: Thank you. Some of these questions relate, this question relates to the movement of clients from one place to another. You already mentioned some things around that, some points around that. But what do you think the key reasons for the movement of clients from one place to another, or one suburb to another?

Respondent: So number one is people from their own community that they can kind of lean onto for support. When I think about when we moved from Toowoomba, this is in Brisbane, to Sydney, what we were looking for was community support, and we had identified that community support here. So we moved to Sydney because of that, and it's similar across migrant communities. They move closer to where they have community support. That's one. Second is job affordability. If people have, or are able to get a job, they will be able to move and access that job. Or even educational opportunity.

Some also look at affordability, it's a key thing. Are they able to afford to pay rent in that place? So they will move looking for cheaper alternatives, if their local area is quite expensive.

Sometimes people consider the local neighbourhood, who is there in terms of not people from your same community, but the behaviours of people around you. If people annoy you too much in that area, you probably would want to leave. At one stage I left my suburb, not because it was a bad place, but because I've had it broken into two times. So for me it was OK, I need to move. Similar issues if migrant families find that the local neighbourhood is too difficult to live in, people are not friendly, they will probably look at places they can move to. So those things are there. I don't think there's any other, off the top of my head.

Interviewer: That's great. Now the next question is around education and literacy programs. I think we've covered off on most of that. You've talked about the early education, so the first part is around the services available for education for children, adult literacy, and you've talked about all the different levels of that, the AMEP, the early childhood programs. And then issues and barriers for children of your clients. So you've already talked about the intergenerational thing and children learning the language quicker, and that sort of thing. And the kind of employment opportunities for migrants more generally, you've touched on that as well. Is there anything you'd like to add, just around that, that you haven't mentioned already?

Respondent: No, I think I've mentioned a lot around that. For education around, early education in providing access to information is critical, and also not only once, but over a sustained period so that people have it ingrained, and they have the understanding they can access the opportunity at any time.

But the biggest issue around, take that to the other side. People who come to this country with prior learning skills, or qualifications, find it difficult having

them recognised here. So it's one that sets people backward and they have to go through a rigorous qualification process. And skill recognition is one element that is overlooked. For someone who has practiced dentistry for 10, 20, 50, 60 years and moved to Australia, a mature dentist, not being able to get into dentistry here is mind blowing.

Interviewer: Surely we could get a better system happening.

Respondent: Absolutely.

Interviewer: I know, it's incredible. It's an incredible waste.

Respondent: It's a waste of time.

Interviewer: That's right, but the impact on the individual as well is immeasurable.

Respondent: It's big.

Interviewer: There's a whole set of questions around legal support, but I don't think we'll go into detail with that, because I think you've covered off a lot of things. But in terms of I suppose your opinion of people's awareness of the legal services available?

Respondent: I mean, there's general awareness. And there is, one thing that we did sometime back was someone from the HSV, there is a legal orientation program that's done, but there is also information sessions that are delivered in CALD language around that. I remember also making an information tool kit back in the day that's still being used today. It's a DVD that they made around legal information and legal services available. And at times also I've engaged with the Department of Justice here to run some legal information session for young people particularly, so there's a lot going on.

What is not happening at the moment is this. Legal support needs to be complemented by social support. For those who get into trouble with the law, they go in, they come out. Post release, they become even 20 times more vulnerable. If they don't have that social support around them, or cultural support if you call it that way, they will then reoffend and go back again into that cycle. So what is lacking at the moment is the post release support, whether that be for a young person or for an adult migrant refugee person. It is a gap. It's an inherent gap, and I have stressed it a lot before, but I think there is much credibility put on the mainstream support that is provided, which does not meet the needs of migrant and refugee communities. So post release is one legal area.

Interviewer: Great, thank you. There's a last question, and it's overall, what do you think the key challenges migrants you work with face while adjusting to Australia and settling into Australia? Do you think you've already covered it?

Respondent: I have covered it. Employment, language, cultural.

Interviewer: That was really good, the last point you made in terms of the post release support. I haven't, no one's mentioned that before, that point. That's great.

Respondent: It's an area that needs attention.

Interviewer: OK, I think, what a great interview, thank you. Really exquisite detail, in terms of insights into different aspects. It's a different perspective altogether, so I'm really appreciative of that. Finally, would you like to see any possible, what would you like to see as a possible solution to helping support migrants adjust well to live in Australia?

Respondent: No wrong door approach. That means a lot of things, but if you can explore that, that would be wonderful. Eligibility of services is one thing, but supporting people properly so that they don't fall through the gaps is really important, and I think if we can adapt a no wrong door approach I think it would be the key to achieving integration, more successful integration, knowing that it is kind of both ways.

Interviewer: Great, thank you. So that's the end of the interview.

Respondent: Thank you.

Interviewer: Is there anything else you'd like to add?

Respondent: I would love to see the end result, the continued development of the work you do.

Interviewer: Yes, we will keep you informed. OK, so that's great, we'll end the interview at 11:39. Thank you (NAME).

Respondent: Thanks very much.
