# Supplementary material for: The importance of information acquisition to settlement services literacy for humanitarian migrants in Australia
Source: PLoS One. 2023 Jan 6;18(1):e0280041. doi: 10.1371/journal.pone.0280041 (PMC9821785; doi:10.1371/journal.pone.0280041)
Supplement: S1 Data — (ZIP) [file pone.0280041.s003.zip › SP_07_Victoria.pdf]

Interviewer: (NAME OF SERVICE) with (NAME) and (NAME). Interview 220. Start.

Alright, (NAME) so just to clarify before we go through the questions, is we're really talking about newly arrived migrants, so first five years is how we're categorising that. And it can be both forced, so refugees, you might even work with asylum seekers, I'm not sure if you get funding, and voluntary migrants. So that can be for family or economic migrants or even if possible, if you do work with people from this category, people that came in as education, for education, but then stayed.

Respondent: Right, OK. Not so much. We're certainly funded by the Department of Social Services to provide settlement services to humanitarian arrivals and refugees within five years of arrival. So we were awarded the tender last funding round, which was the end of last year, for the (NAME OF LOCATION), until 2020. So that's part of the settlement engagement transition support that we're providing to families within five years. However, we don't work in isolation and yes, we do have presentations from families and clients who are either asylum seeker, although we're not funded but we would really refer to appropriate specialised supports, or in addition to that that we have families who are on temporary protection visas shared visa holders, which is the safe haven enterprise visa, which there's a large number in the area and they have special conditions attached to their visa. And it seems to mainly apply to the Rohingya community. So there's certainly concerns around their challenges, unique challenges for them because they're not receiving settlement support per se. They're really reliant on good will in the community.

There are some minimal services really that are linked to them, but they're coming from a very low base in terms of literacy and numeracy. Yes, they can access some (SERVICE NAME) English, 510 hours, the AMEP programme. But if you've never had an experience of being in the classroom and what that's like to go and sit and you don't understand the alphabet, it's really challenging for them to sit in that environment. And also they're trying to manage, work is a priority for them, housing's a priority, financial stability, education is for their children certainly, and stability. That's very problematic for that community. However, we're not funded to support but we can in other ways. We've created strategic partnerships with another agency to assist and especially offer specialised intensive supports to them. But we're very aware in the community, in this region of their situation. We have about 20 families now from that community. Large family groups living in (NAME OF LOCATION). And it's really difficult if you haven't had a rental history, you know, rent receipts, to obtain bond loan, how do I navigate that? Even online, everything's online. If you've never… if you don't know the alphabet and you look at a keyboard or a tablet, it's not going to really make much sense to you.

And really the idea would be if we had trauma informed practitioners teaching perhaps English, because it would be recognition from where they're coming from and the challenges that they have. So at the moment what's happening is that the community itself are very aware of those challenges and have come up with some solutions as a collectivist culture and, you know, their faith-based belief system is very much about looking after one another and supporting. So if there's someone in the community that has a level of education and awareness of English, they will be… that's a resource, that's a resource, but it's a lot on that one person, tremendously difficult, and they're dealing with chronic stress over a long period of time. But services are aware in the area and we actually, I just came from a meeting today to discuss this very issue. We're putting some sort of a task force together and bringing those services together in the (NAME OF LOCATION) regional area as to what we can do, how can we deal with this?

Interviewer: So how can universal services deal with that?

Respondent: Yes.

Interviewer: So you mentioned… so I just wanted to clarify that. So you mostly work with…?

Respondent: Yes, humanitarian arrivals and refugees. And our latest new arrivals have been the Burmese community, for me, I've been… prior to that we have Afghan community. I've worked a long time with the Afghan community in the city of (NAME OF LOCATION). And really, they present as the largest group. But more recently it's been the Burmese community. So certainly I've been kept very busy on a Wednesday, I work part-time, so I've been working on a Wednesday. I'll reach capacity to meet with the families and any referrals that I receive. And sometimes it might be walk-in or it could be I receive referrals for humanitarian settlements programme AIMS. Word of mouth is very powerful as well, sometimes families present that way if they've been living interstate, there's been movement because Victoria, particularly Dandenong, is seen as the capital city, essentially they gravitate. Especially Springvale and Noble Park, for the Burmese community. And it's seen as employment opportunities are greater or educational with the thriving communities here, it's a multicultural area so they feel comfortable, more comfortable. But particularly comfortable in Springvale and Noble Park certainly.

Interviewer: So beyond SETS, so SETS is generally the case work, is that right?

Respondent: Yes, it is.

Interviewer: And then beyond SETS are there other services you provide for new migrants here?

Respondent: Well, we have a range of programmes within our organisation. So we have the economic participation team and they're working hard on social enterprise. So around cooking collective is one example of that, where we have a group of women mainly who have those skills and generally terrific cooks, but they want to seek employment in maybe hospitality or cafes or, you know, maybe the tuck shop at the school, the local school. But again, we have to inform them of, you know, what it is to put a recipe together, oc, health and safety obligations, food handling. This is all foreign, this is… you know, these society puts that on them and these are expectations that we have. So it's a great way of dipping your toe into the Australian workplace by creating a cooking collective that, you know, exposes them to all of this. And there's a chef that runs it, we have (NAME) (?), so over a period of I think eight weeks. And essentially we've got, we've accessed a community kitchen. And the women come and they're doing theory-based study as well as hands on. So they're preparing, they're measuring, they're doing budgets. We're doing, introducing topics like numeracy, literacy, also financial literacy, which they have this knowledge but in practice how you put a recipe together, what's the breakdown of costings, how you actually do that, what that looks like, what would be the expectation of an employer in that situation, how do we make a profit, and producing food.

So that's been hugely beneficial. And the women are very keen to roll into employment following that, but building their confidence, empowering them, and also their self-esteem. Building on their existing knowledge. So we're working with that. And hopefully we can do in-house catering functions. Often we have functions here for various events and forums, we can showcase that. Or the women might go on to create a company or create their own catering, their own enterprises as well. So it's a way of empowering the community and building confidence and skills. So that's one example. Another example is the cooking collective, not just the cooking collective, but bicultural interpreters collective I should say. So with the last tender we received reduced funding for interpreters unfortunately. And so we had to really think about our ways of working with the community and be creative, but how ethical practice. We're not in favour of utilising children to interpret on behalf of their parents, that's not OK. So… and many parents are tempted because they lack confidence, to take their children out of school or not let them go to school if they have an appointment here perhaps. So we discourage that and we talk about the value of education, which they do appreciate. However, they need to be able to communicate with us.

Now we do have an on-call service and it costs, we're mindful of that. But what we've done is utilise our bilingual workers that are on staff and we do have a few, except for Burmese speakers, not easy to come by. So that kind of reflects really the community, haven't (?) advanced. But I did have a social work bilingual student from

Melbourne University who was able to assist me and I was his task supervisor. So that was really fantastic to utilise his skills and we did. So in the main, that's been a challenge. But our bicultural, our interpreters collective was our response to getting employment for our bilingual workers out there and training them up so we could use them in the community and now we're just waiting on approval, it hasn't passed yet. There's insurance that we need to cover, also accreditation around their language skills. But certainly that will be advantageous for us. So that's how we've responded as an organisation to the challenges, the current funding challenges, and to still deliver services to our communities, build up that trust and rapport, it's so important. And so, yeah, we're just waiting on the go-ahead for that.

And we also noticed, because we're keeping track of trends as well and gaps in the community, in our work practice we see that. And one of the things we noticed and we got asked a lot for was from the NDIS in terms of the roll out and we had support coordination agencies who were really seeking to access bilingual staff to connect with their clients. There was a gap. And so we said, well in one way we can help. Now my social work student was able to help largely, especially for my clients because we were working collaboratively with our clients to, you know, go through the pre-planning stage and navigate… that's huge, to navigate that system. And it was also new. The rollout was here in the southeast of Melbourne in 2018. So it was very hard for our clients to get their heads around, like you're a participant and you have choices. And someone, that's a whole new way of thinking, changing their perspective. You have choices. Yeah. And so it was walking them through that.

And, you know, literacy is always challenging because you might have some level of English. For my Burmese clients not a lot, they're coming from a low base. But if you have some level of English, if you're in a legal setting, it's different terminology, in a medical setting different again. How do I explain these terms, what do they actually mean? So it's taken a lot of work from me and from my colleagues to explain and navigate that whole system. But the bicultural interpreters is one way, our response again, of equipping them with who have worked in that space as well as understand the terminology. And there's been training from I think Centre of Ethnicity and Health so that they could, they could have those skills or a knowledge around the NDIS terminology and sitting in our planning meetings and helping our clients, it's hugely beneficial. So we're hoping for free (?), for service with that as well because we do get those requests from agencies that work in disability. And certainly our families have presented with, there is physical as well as intellectual disabilities. Yeah. Not to mention children. And that's, you know, really incumbent upon us to make sure that they're getting those services. Yeah.

Interviewer: So are there any services that you see are needed but are not available for new migrants?

Respondent: You know, we have a plethora of English language classes. AMEP programme provides 510 hours. It doesn't provide enough… some of my clients, if they can write their own name or recognise the alphabet they're doing well. It's not equipping them for work readiness, for steps advancing their education. We find it takes a long, long time. And I think there's a couple of factors for that. Again, their history of resettlement. You know, being in a secondary country before they came here.

Interviewer: I should say, literacy is going to be a question that we talk about more specifically later, yeah.

Respondent: OK.

Interviewer: So you might be overlapping there. But English language programmes are something that need some work?

Respondent: Yes, I think so. Yeah. I definitely think so. Yeah.

Interviewer: Any other areas? Or any other services that are…?

Respondent: Certainly job network providers, very difficult. It's a distress for our clients. You know, they're required or asked to commit to appointments, to look for work online. Many of my clients really need help with that, they haven't got the confidence. Their children would be more savvy, certainly, with navigating that. And they do rely on their children, which presents other problems because we're seeing children parentified and access to information that they shouldn't have really access to. Especially financial, like internet banking would be one example, or making decisions for the family or for themselves that's quite beyond their capacity. You know, they haven't got all the information or facts or the capacity to make those kind of decisions yet we're, I'm seeing that. We're very mindful of that. We certainly take, go to great lengths to discourage that and if it means bringing in the family or for me to go and visit with them to go through it again… I even notice with an interpreting service, I might go through and explain, they might this much. And it means, it just requires checking in with them. Because they've a lot going on, it's very hard to take it all onboard.

Yeah, so we find that that's a challenge and I try to break it down and explain things in such a way, trying to make it as relevant as possible to their situation. So we do see that, which again comes back to literacy.

Interviewer: Yeah, it's very linked.

Respondent: It's very much linked. You can't separate. Yeah. You can't move forward. Yes, we have apps, and yes, we have MyGov, and it's easy to navigate. But if you cannot… I mean a lot of our clients are very good

with social media, they see the value in that because it's connecting with families overseas. You know, for a country like Afghanistan, it's well above 50% in terms of access to Wi-Fi, internet. There's no landlines but we've got, you know, internet access. And they utilise that. One example from my family not so long ago was an Afghan family, a lady with her six children and she'd relocated from Tassie to Victoria because she felt there was more opportunities here for her children. Great decision, very logical. And I asked her what was the priority, she had complex issues, health issues as well as settling, grappling… in fact, she'd lost her husband in the war and grappling with, you know, mental health and post-traumatic stress and a lot of trauma. And the priority for her was to get access to the internet. That was her number one thing. I was looking at all of the complexity and going, OK, but that's what she wanted. And great start. And we got her connected very quickly and straight away onto the phone.

So there is, you know… she was very au fait, was using Viber, WhatsApp, and FaceTiming family back home, her loved ones, particularly her mother and extended family in Qatar (?). So they would be quite au fait with that, as I said, with social media and they see the value in that. Sometimes that can be a good or a bad thing. But yes, it's not in their own language. You know, trying to navigate Australian concepts and systems. Very, very difficult. Very difficult. And there's a lot placed on them, demands. Especially with job active networks and if you don't you'll receive a letter from Centrelink saying your payments could be potentially cut off or you're going to have to reapply, these are the requirements. I see mothers and families, you know, the ParentsNext programme, for example, they have to go through activities to prepare for getting ready for their working life employment. There's a lot of stress associated with that.

So it comes back to English language literacy, financial literacy, and then you… that's your base. You can build from that. But if you don't have that you can't participate in society, not in a meaningful way. Maybe in your own networks, but that will only sustain you for so long and then you're back to square one.

Interviewer: Yeah. So can you tell us about any methods that you use to measure the effectiveness of the services that you provide here?

Respondent: That's a good question. I'm trying to think of what we've done. Certainly I guess we don't work in isolation. So, you know, we wrap around services to clients. Certainly we would use our own evaluation with clients as to how they're progressing. We give feedback to other services. In terms of measurement, they would be more in the form of feedback or anything that they might provide to us that we would certainly feed back and say, hey, this is not working, can we look at doing this differently? So certainly that does happen, yes, it's fed. I'm trying to think of other things that we do off the top of my head.

Interviewer: There can be a tension between particularly if you have limited resources about directing resources to actually providing services.

Respondent: Yeah, services. Yeah.

Interviewer: How much is leftover for then evaluating them?

Respondent: That's right. That's right. And clients themselves will say, you know, that OK, you know, I'm doing English classes but I don't feel I'm work ready. And they're not, they're telling us. The other thing is with employment, because I mean I know the focus is very much on the three Es, and… but employment, there's not a lot of employment out there. I mean, say for even entry level factory work on the floor you have to have a fairly decent comprehension of English language to, for safety reasons. If anything else, if nothing else, oc, health and safety, follow instructions, read instructions. Even to apply, you know, you go through an assessment. And that's quite understandable for an employer, potential employer to know, are they able to do… they may have the skills but do they have the language skills, do they have the numeracy skills, do they understand why I'm asking them to do this, can they participate in workplace conversations, can they give feedback?

You know, so there's that challenge and what I'm hearing and seeing from my clients, and they're the best judge, is they're not ready for that. They are nowhere near that at all. And that's a real gap, that's a real… yes, we can have conversational English, and that's good, it builds confidence, reduces isolation. But in terms of employment, there's very little. And in fact in (NAME OF LOCATION) it's more retail-based. So we have… yeah. Or around, what else have we got out there? (NAME OF LOCATION) would be, most people who live in (NAME OF LOCATION) commute from (NAME OF LOCATION) to (NAME OF LOCATION) for work or beyond. So there's really nothing locally or entry level. We have some agricultural work, excuse me, in (NAME OF LOCATION), (NAME OF LOCATION) region. Some of my clients have been able to avail (?) of that. But really, there's very little in terms of employment.

I notice some of my Burmese community are accessing work through their own contacts in Springvale. So that could be around the market or community gardens, working on a stall, working in a restaurant. They've been able to… but that's just good will. Their own community giving their own people a hand up who are desperate. They want to work, but they haven't been given the tools and the skills yet to do that.

Interviewer: Alright. So the next question's related to how migrants adjust to Australian culture and society and the kinds of challenges you see new migrants facing. So can you tell us about your understanding of how

new migrants understand Australian culture and society, what their level of understanding is?

Respondent: I think it's limited. I think that very, yeah, it's very difficult for them to navigate or to comprehend unless it directly impacts for them. And again, it comes down to, you know, requirements, say Centrelink. Yes, there's an interpreter provided, but why am I actually… they're being directed to go to this computer, well I don't know how to do that. They're looking to the interpreter for help. And, you know, it's… unless I'm with them, I can certainly address it. One way the organisation has done that is to give information sessions around not just language literacy but navigating Australia's concepts and systems, understanding, you know, you have obligations. Say, we have onto the road driving programmes, a very popular programme with us. But firstly, our clients have to attend five sessions and that's, we bring in Vic Roads, insurance companies to talk about insurance, the importance of insurance, having insurance, fines, civic compliance, what that looks like, what your obligations are before, and they must attend those sessions before they can actually participate in the driving programme. So that's very valuable.

So it's things like that that we need to do more of. Funding is always a challenge. And the demand, especially for that programme, we have a waitlist. We also have a learner's programme, learner's permit programme, and, you know, that's very beneficial, especially for women too who are isolated in the community. Because we want to empower them. And if we don't lift them out of the communities, because the infrastructure is a challenge. Yes, we have a bus service, but it doesn't go into suburbs, it goes around. So how do I know where to go? And if I've got little ones that's very problematic. But if I have a car then they're more likely… or if I have a car I might be able to carpool and go together. So there's certainly, we certainly endeavour to provide information sessions around Australian law. We've had Victoria Legal in, legal aid in to provide those sessions and workshops. Especially family day care was an issue for us for a while. You know, what did that mean? Some families were setting up family day care, being providers, but they didn't understand what does an ABN mean and what are your obligations. And that was very problematic. So once we saw those trends happening in the community we were addressing that very quickly.

So in that way we can provide information and support. But it takes time, it takes funding, resources, certainly… I think our clients are feeling quite overwhelmed at times. They're really… and in danger of being sidelined really, you know. And children are adapting a lot more quickly and they can't keep pace. And then you have that push-pull, that interpersonal relationships, it's a stress. So certainly that's what we are seeing. Yeah, and navigating, again, like I mentioned earlier, the NDIS system and appropriate specialised supports to, you know, knowledge, health, refugee health is certainly supportive. But again,

for our clients, if they have chronic health condition themselves or trauma or mental health, that's really challenging. Really challenging.

Interviewer: Alright. And so do you see new migrants having the chance to practice their own culture as well?

Respondent: Yes. Absolutely. It's very much, it's very valued. Very valued by them. And sometimes there's a bit of fear around that and what we might lose with that because they can see or their perception from the older generation is, oh my children are become westernised. And they see that as somewhat, oh, this kind of fear or our children won't attend, you know, they won't follow our belief system maybe or they won't practice our faith system. So yeah, I've certainly seen that with teenagers and families and parents and challenges coming, you know. But, yeah, and that's been raised with me and how do they actually deal with that. We're saying, well it's about, you know… I work with families, I work with medium intensity clients and families, children, women, and we have conversations. We're saying, let's talk about this as a family, you know. It is important and it's a value. But your relationship is also important. So if we bring it back down to that then they're OK, you know, we can address it. And for most of the families I see, yes, parents are saying, oh that's a perception, but is it based on fact or is it… you're comparing that to the past or your settlement journey but that doesn't necessarily mean that they're going to walk away from that or they're going to not value it.

And I see for most of… it's a collectivist culture, it's very strong. And we see those connections. Yes, there's strain and there's stress and they might pull apart a little bit and bend, but there's a connectedness there and that's their strength. But we're very focused on strength-based practice, but without ignoring needs and risks. That's very important. Yeah. So we try and find that balance. But it's conversation.

Interviewer: Yeah, alright. So the next question is related to migrant sense of belonging in Australia. So do you run any projects specifically that try to create and enhance migrant sense of belonging and cultural inclusion?

Respondent: We have a great programme here called Cultural Insight, which we have, we actually attended last night, just from myself, so we promote that to individuals as well as agencies, mainstream agencies to be…

Interviewer: So this is for new migrants?

Respondent: New migrants and also agencies, mainstream agencies, working with CALD community. And especially newly arrived, so like the Burmese community, Afghan. Syrian community we find they don't need our help as much. I had a large Syrian community that arrived last year, the year before, 2017, and they were very educated, had worked

previously for American or British companies overseas, and maybe just stumbled a little bit on little things and then they were off and running. They really didn't need our help as much and that's the benefit of education. We could see that. Can't draw the same parallels with our Burmese community or Afghan community, very different. As I said, they didn't need our help as much. So yes, Cultural Insight Programme we host, a very valuable programme. But we also have citizenship programmes and citizenship information sessions. And they are well sought after. So our clients after (?) four years, they can start to think about accessing or applying for citizenship. And they really value that.

Interviewer: So this programme helps them navigate that?

Respondent: Absolutely. The test, they have to sit the test, yeah. Yes. Now we used to fill in citizenship application forms, we don't because we're not legally trained and we noticed with the Department of Home Affairs it was, you know, to be very mindful because the information that clients come in on needs to match the information that they're putting into the document. And it's 29 pages. And often our clients can't remember, you know, some of the detail, it's really hard, it's really challenging, even for us it was challenging. And so as an organisation we made a decision that really it's a legality and we're not really trained to do that. So… but what we can do is provide information sessions to prepare them for citizenship test. And that's very much sought after. And of course we use that opportunity then to go through all of their obligations, what it means to be a citizen, voting enrolment. Sometimes we do it by, we do it structurally as well, but sometimes we also take opportunity like our learning space we have.

So we have another worker who goes out into the community and has a learning space where you have conversational English, maybe sewing classes, things that the ladies are interested in, and childcare, and we introduce topics to them while they're there of interest to them. What would you like to hear about, what would you like to know about? And then we will bring in the relevant organisations and services to promote or to present that. And they have the opportunities to ask questions using an interpreter as well. And that, it takes out the fear, it takes out… you know, it's a nice, comfortable space and they have the opportunity to I guess feel safe and ask those questions that are relevant or check in with each other. So that's very beneficial, we find. It's another way of doing that.

For our services here, we provide services from early childhood right through life course. So we have a carers programme, we have diverse care, which provides in-house home help and personal care services to I think ethnic, a range of ethnic backgrounds, I think it's 170 languages, huge. That's another social enterprise that we have. We have planned activity groups. We have health and wellbeing team, it works very closely with that too. So, you know, we can really deliver those services to our clients from, no matter what age.

Interviewer: So this relates to the next question actually, do you provide any programmes that are responsive to health and wellbeing? So you've got a health and wellbeing team here. So what programmes do they kind of run?

Respondent: Yeah, well they just were…

Interviewer: Or services do they offer?

Respondent: So certainly with the planned activity groups they will be delivering health and wellbeing information sessions to the groups and the groups might ask for that. So it could be around breast screening programmes, diabetes, exercise, tai chi, information of ageing, ageing well, whole range, a whole host. Anything that's relevant to women's health, families, yeah. They've also received a recent contract or been… extra funding, for that. So that's going to really open up. We have podiatry service here as well, that was opened up last year. The families can come and access that. I think the podiatrist is here on a Friday. We have, we've had large forums, seniors forum around connecting with relevant services, My Aged Care, and bringing in all those relevant services to them so they felt supported. And it would be CALD specific as well. Some people prefer that, other people would prefer to engage with the mainstream service because it's about privacy and confidentiality and they don't want someone in their community to know that they're doing that, which is fine.

So it's… we connect with [indistinct 41.51] Care, which is a big organisation in (NAME OF LOCATION) that deliver aged services as well, we work very closely with them. And they would also provide like assessments for in-home supports. They would match volunteers as well or support workers to the client. Community Visitors Programme is another one, we offer that. That can be in aged care settings where, you know…

Interviewer: But is this for new migrants then?

Respondent: As well. That's offered, yeah. So under 65 and over 65 we provide services. And we feed that into the service access team who assist, if they're under 65 and somebody presents perhaps with a disability we can refer into that team and they will assist and support them with navigating NDIS supports. If they're over 65 then it goes into My Aged Care and they also assist and you have the Carer Support Programme as well, support for carers in the community.

Interviewer: So is there any barriers specifically or enablers that you see for new migrants in accessing specifically these health services, beyond what we've already mentioned in terms of literacy? Is there anything specifically to do with health?

Respondent: I guess their first port of call is their GP and having, that's really important, and having a really good relationship with their GP and especially a GP that speaks their language. That's very much, I notice my clients will gravitate. So word of mouth in their own communities that they would trust is very important. Rapport. So that would be the first port of call and that we build up strong relationships and partnerships with health services, like maternal and child health, women's health, refugee health, all of that. Not to mention mental health services. So that would be certainly something that we liaise with strongly, not just in my team but across other teams as well. Psychologists as well and having those conversations. And insights into maybe the cultural complexities, sometimes mainstream services will struggle as to understanding why clients are missing appointments or what's the reasons behind that. So that's what, we have a space to advocate on their behalf and navigate that to them, with them.

I think certainly GPs would be pivotal. And if they don't have a great relationship with the GP, and that does happen sometimes, that we encourage them to make changes, because you can and you have rights, so it's a human rights-based approach. And we educate and inform around that and that that's OK. And it's about you feeling comfortable. And that's empowering them or building their confidence. And awareness around women's health too. I've seen that with my families, you know, that the reluctance maybe to verbalise what's going on for them and it's a very important space, especially for, well for everyone, but for young women as well to say something's going off, it's not feeling… you know, I can, I don't really want to go to mum's GP because he might tell mum or dad or whatever. And so we encourage them to have that conversation, you can make these decisions and make choices to see… and that's been very revealing. Yeah. Insightful.

Interviewer: It sounds to be. So these next questions are about any programmes that you might offer new migrants to enhance financial literacy or managing money more effectively. And you said… do you have programmes that help with that?

Respondent: Something we've been working on, we certainly… a large part, as a SETS worker would be information and referral. But we do, because we see that on the ground we feed that back to the organisation saying, hey, there's a real need in the community for this to have happen. And we really try to address that. Yes. So again, it could be in the learning space or it could be with a cooking collective, and we would bring in…

Interviewer: You build into what…

Respondent: We build it in, yes, by stealth. So they're doing it, they don't realise they're doing it. Because our biggest fear is payday lenders. And we have had… yeah, we've had that happen. And it's awful, you know. And community services are very aware of that, like financial counsellors, that's their field of expertise. But also as a worker we

have an awareness around vulnerability and people are desperate and not always of a, with, you know, what's the requirements and not knowing, oh, this sounds too good to be true, it usually is. So of course we will have a conversation around that, we'll try and introduce that by stealth, have conversations at the cooking collective, learning space, or any workshop, we will bring it up, especially when it's topical. And we see it reoccurring, that's when we need to act. We'll alert other agencies that are very informed, like financial counsellors, going, hey, this is happening, can we advocate with ACFA? Or, you know…

Interviewer: What's ACFA?

Respondent: It's the Australian Financial Insurance Commission… I had it written down. I had…

Interviewer: It's about insurance?

Respondent: It is and consumer affairs, and AFSC (?). Actually we did have one case that went directly to AFSC (?), which is the financial regulator. So it's really good to feed that back because it supports what financial counsellors is doing as well. And then we're talking about what actually exists in the community. So at Community Information Support Services there are, say for example, the NILS Loan, which is the No Interest Loan Scheme. So if you're at your home address for at least six months and you're, it's postcode related, you can access that local service, community support service.

Interviewer: Who gives out NILS Loans? Who gives out the no interest loans?

Respondent: The community, it's part of the initiative with, The Good Shepard started this, this initiative quite some time ago and it was picked up by local Community Information Support Services. They come under SS Vic (?). So the local one here would be SECL at Foster Street. They have an emergency relief…

Interviewer: Southeast Community Links?

Respondent: Southeast Community Links. They have an emergency relief programme, they have assistance with bills, utility bills, accessing utility relief grants, information, and the NILS Loan. Which is fantastic. Say, if you need a new fridge, washing machine, you can't do without… these are essential items. But you can acquire them and pay them off over time and not incur any interest. And that's a much more healthier way, financial way. And I know Community Information Support Services also have workshops around financial literacy and they deliver those in the community.

Interviewer: So what are these community information support services, I haven't come across it?

Respondent: They come under SS Vic (?). If you leave me your email contact I'll send you some information. They are postcode related. There's one in (NAME OF LOCATION). There's one in (NAME OF LOCATION). And the community, the broader community, anyone from the community, if you're on healthcare card, pension card can access, and your address is in that geographical area, postcode, you can access. Same as SECL in (NAME OF LOCATION) or here in (NAME OF LOCATION). And you can access that information. So we work very closely with them as well and we educate our community and our clients around that. This is where you can go to get material aid or you can access information or get support around financial assistance. And I believe they record data as well for the department, that's fed back because these people are living in the community, they're invested in the community, and they're seeing these trends as well. So it's very important.

Interviewer: So what financial challenges do you see your clients facing as new migrants? What are the key financial challenges?

Respondent: Lack of income.

Interviewer: Yeah, just…

Respondent: Minimal income. Dependent on Centrelink income, which is New Start, is, what, $40 a day. What's that? How are you supposed to raise a family on that? Really, realistically. And the costs, Australian costs, cost money. Put fuel in your car, if you've got one. You know, food, utilities, you know, education costs. You know, school costs. Yes, they have the CSEF programme, which is the Camps, Schools, Excursions Fund, which covers some cost, but if you have a large family it's a struggle.

Interviewer: School books and uniforms.

Respondent: Well, schools are… you have to have devices. Well, they cost. Where am I going to get that money from, if you're a mother of four or five children, where are you going to get that? You're going to make decisions around who actually gets that or something gives, you know. And I've seen that, I've seen parents go without so their children do get education, they value education. And they will go without, go hungry, so their child gets that device because schools require that. That's a requirement. You can't not have it, you must have it. So that's a stress. And they don't want their children to be left behind, they want them to advance. And often we have this discussion with say teenagers, great people, young people who are going, I don't really want to go through and do VCE, I'd like to do another pathway, I'd like to go through VCAL or TAFE system, but mum and dad are so focused on them acquiring their VCE, they're pushing, they push them. And so we have to go and really sit down with the family and go, there's actually different pathways, educational pathways that you have

not considered perhaps that are just as valuable and worthy and you can do this and still achieve, still advance yourself.

So I've had a few conversations like that in the past with the Burmese community. Because parents are, they're fearful. They want their children to achieve even if they cannot. They see their role as OK, I can't speak the language, I may not yet, I may not be able to participate here, but I can work or I can save or I can borrow from my own community so my child gets that opportunity. And there's a lot of that because it is a collectivist culture.

Interviewer: And are there any cultural financial challenges? Like sending money back home?

Respondent: Absolutely. Always. And I've worked with the Afghan community and I would see that all the time. Tremendous pressure from family overseas because their perception is oh, you're doing well, you're in Australia. But they don't know what it takes to make ends meet here, they've no idea. But families wouldn't say no to them. They will, they will send money back. It's an obligation. It's something that's really important to them. You know, this is my mother, this is my loved one, I can't, I know how she's doing, it's tough. I might be doing it tough, but this is my… it's very, very valued. And I see that same thing in the Burmese community. They will… I have a family, the mum, that wants to sponsor, she wants to sponsor her son and they've been given approval. He's in Nu Po camp in Thailand, he's coming to Australia. They're just waiting on the final medical. They had to put a deposit together firstly to get the loan from International IOM, what is it?

Interviewer: Organisation of Immigration.

Respondent: Exactly. They did it. And I know they are doing it tough but they did it. They scrimped and they… they probably didn't eat. But they've got it together because that's their loved one and they're going to get them out of that situation and get them here. Once they're here, that particular family can repay that loan through Centrelink by deducting from NewStart, because that will be the first thing that he'll probably be on.

Interviewer: And just out of… I guess also further on that, is there any other kinds of things? Someone mentioned, you know, is there gendered decisions on finances? Someone mentioned that, which was interesting considered that women are often getting money into their accounts for children which then might pose a problem. So is there any other culturally significant challenges?

Respondent: Absolutely. Yeah, absolutely. It can cause conflict or, yeah, because in Australia we have equal rights and certainly for, you know, it is a patriarchal society or system, men might just feel threatened and, well, you know, I have the final say. So they will take it as, you're attacking

me, attacking my authority. Where is my place in the home, I used to have, you know, this sense of being the provider. And… but of course women are receiving because, let's face it, for a lot of the women in the community, they can make their money go further. They really can. They have the skills to do that and it's well documented here and abroad that the community benefits. Give it to the male head of the household, it's usually individual decisions are made, he's not including the other members. He might discuss that with another male in the family but the women are not included in that discussion. Whereas the women are more likely to share that or it would be compromises or collectively make decisions or consultative. Whereas the men would not. And that's the breakdown.

Of course if there's a shift and it's perceived as the male as a shift in power, we're bringing the woman up, she's having more decision making, he's perceiving it in a negative way, which can go to you're attacking me. Yeah, you're not respecting me, where is my place now. That can lead to, feed into interpersonal problems or go towards substance abuse or, you know, yeah. Because it's hard. I understand it, it's hard. It's a big shift for them. It's difficult. So it does, certainly we have seen that. It does cause conflict, these inter-conflict, which is, yeah, something we do try to address or have conversations around that. This is why… yeah.

Interviewer: Well, so speaking of conflict, the next question's related to legal challenges. So do you offer any services or programmes which supports clients, your new migrants, with legal issues?

Respondent: We used to have a migration lawyer, a migration team, but we lost funding for that quite some time ago. So we actually do get a lot of requests for migration support and assistance, especially with sponsoring loved ones from overseas, citizenship queries of course. And, you know, our clients, you know… it takes a lot for them to advocate for themselves. They're not likely to go to their local member, very few, even though we explained that, to perhaps tap into as to what's happening with their current applications with the department, et cetera. Some do. We do make them aware of that. So we do tap into Refugee Legal, which is in the city, in Collingwood, for support. But again, they're under pressure, financial pressure too, and there's a big demand on their service. So they will do maybe one bit, whatever that query is, part of the application but not all of it. And then… so you're given one part of the jigsaw puzzle, not all of it, and that's very piecemeal. So… and I understand it, it's very difficult.

Certainly there are private migration agents working in the region who are somewhat affordable and the communities have awareness around, especially the Afghan community, would have awareness of who to go to. Or they might come to me and ask. Again, we're not legally trained but I would certainly refer to the migration agent. I would offer them Refugee Legal but also private. We do have a private migration

agent who used to work for us who's now working for herself. And she will give, you know, affordable rates because we've referred. We don't, strictly speaking, it's not a partnership as such but she's certainly, you know, inclined. She will do… and we encourage our clients to go through accredited, registered migration agents. Please do that. Sometimes clients might scroll or find someone or someone said in the community try this person or Google and it's not reputable, they're not registered and they don't know. And that has happened in the past so we're very much at pains to… don't go down that track.

Interviewer: So what about the legal services or programmes in relation to violence or discrimination or criminal activities, other things aside from migration? Is there anything around that?

Respondent: Certainly, I mean WAYSS Family Violence Services, certainly we refer to a lot and since the Royal Commission into family violence there's been a lot consciousness raising and awareness around that. So they're a fantastic service that we do and have referred to and they're very specialised and sensitive to CALD specific. We have referred to In Touch family violence service, which is also, they've definitely got the legal lens as well and migration attached. So they have a very good understanding of visa and sponsoring, you know, families to here and the problems that might present because once they're here, partner visas, they can't access Centrelink for two years, they're on a 100 visa, and that can create problems. So that we certainly do refer to and they're well equipped to deal with both family violence as well as the legalities. And they have a good understanding of visa requirements. So that, I feel, is very relevant.

Interviewer: And is there any challenges for new migrants in accessing legal services for migration or for other, any kinds of, you know…?

Respondent: Well, Refugee Legal is inundated usually. There's a long wait. It's not unachievable, it is, but it's definitely a… it's not as responsive.

Interviewer: And community legal centres?

Respondent: Community legal centres will but they haven't got the migration aspect of law. They don't… they generally don't deal with that at all. So they would refer to a private migration agent or Refugee Legal.

Interviewer: But what if the issue wasn't about migration though? What if it's just another legal issue?

Respondent: Yes, they would. They would certainly, they would certainly work with the client, certainly, yeah. And that's wonderful and we're very thankful. The (SERVICE NAME) Legal Service does terrific work in that space as well as (SERVICE NAME) Community Legal Service. And they also have a tenancy worker, which is great to have because she can really navigate that, you know, that whole field. She's taken a

couple of cases to VCAT, where clients have been disadvantaged or exploited. Yeah, yeah.

Interviewer: Alright. So the next question is about movement of clients from one place to another. So you know, do you see any trends or are there any reasons why you see people, you've already mentioned people moving to (NAME OF LOCATION) for instance, and what the reasons for people moving around?

Respondent: They do, yeah. The perception is again Victoria is education, employment, better opportunities. Better opportunities for their children and possibly for themselves, financial benefits of that. That they can, yeah, that it is progressive. And we have clients move from Tasmania to here for that reason. From New South Wales, Coffs Harbour, I had a client come in from… and they just, they found me. I was working, I was collocated with another agency in (NAME OF LOCATION) because that's what we do, we go outreach, and (NAME OF LOCATION), and they found me, which was great. And yeah, that was definitely their perspective and their reason for moving. Also clients do move frequently from townships, like from (NAME OF LOCATION) to (NAME OF LOCATION), and the biggest reason for that is rental affordability. Not housing affordability, rental affordability. Because they have large families and they need the room, so they're moving out. Of course that causes other problems in terms of infrastructure and transportation and getting around. And then services are not there, we're here. I mean services are funded but they're not going out. We certainly are. But not enough.

So not enough decentralisation of services to regional, semi-rural communities. I certainly do and I certainly go to Living Learning (NAME OF LOCATION) and there's a community hub there and it's a fantastic model that [indistinct 66.53] council have put together whereby we have Brotherhood of St Laurence, Campbell Page, which is an employment service, we've got (NAME OF LOCATION) Community Legal Service, we've got a lawyer from (NAME OF LOCATION) Legal Service in the library just down the road, we've got civic participation in terms of the men's shed. You know, that's that intercultural connections happening on the ground. Women's Friendship Café, again another great resource. English classes have been hosted upstairs to VCAL students, [indistinct 67.34] programmes, it's all there and it's great to be in that space and coming together at 11 o clock for community lunch or whatever. Everybody comes out and we're mixing. Civic participation, it's good stuff.

But services are not doing it enough and I'm not sure why, I don't have the answer for that. But it needs to happen because, you know, local, like (NAME OF LOCATION) Shire Council and Police Force would be very appreciative of that. It's breaking down those barriers, it's… new Australians are, you know, getting to know the landscape, you know. They're getting familiar. They have ownership. We want to

build that, we want to build that. And when they put roots down they're invested in the community and it's affordable. That's why they're moving. But services need to do more of that and encourage to do that. For shared visa holders it's more problematic because they have minimal supports. Employment is a big factor that will also determine as well.

Interviewer: Yeah, where people are moving to.

Respondent: Where they're moving to.

Interviewer: Alright. So next question's about migrants access to education and literacy programmes. So do you here run any education or literacy programmes at all?

Respondent: No. It's more a conversational, we really leave that to teacher training who are equipped to do that. Certainly we would like I think to see new models with that, like trauma informed practice in the classroom, like I alluded to earlier. I think that will be hugely beneficial because then we would have that lens, that understanding of where our clients are coming from and how difficult it is to actually… they've never sat in a classroom, they don't know the terminology. It's really quite overwhelming. And if you're coming from a very low base, and I mean zero, it's a good place to start if you have someone who has got lens on, has that innate understanding or insight into the complexities or challenges, and to walk them through. And for that to be meaningful. We have a plethora of English classes out in the community. We do. And great community centres who are doing their best. I'm not taking it away from them. But we would be lucky if our clients were able to write their name or have a good understanding of the alphabet and putting sentences together or to be able to fill a form. Even that, it's not, it's not enough. I think it comes down to training.

Interviewer: Of people providing courses?

Respondent: Yeah. Volunteer… yes, we have volunteers but it's not enough. Yes, it's good to have volunteer's mentoring, but still it's not enough. It's not pitched well. It's not… and you know, they're not work ready. They're just not and we're setting them up to fail. That's really what we're doing. We think we're helping but we're actually not. It's just I think a gesture. It's a gesture. It's a good gesture and it's a start but it's not enough. I feel that it needs to be rehashed and relooked at, how can we do this better? And I think one place would be to start is that trauma informed practice and really getting an insight and understanding where our clients are coming from, what they can achieve, and guiding them, guiding them through that.

Interviewer: Do you see any barriers to children of your clients in accessing education?

Respondent: No. On the whole, I have to say no. Because, you know, families are keen for their children. They value this. They recognise the value. And so we see children enrolled in kindergartens, enrolled in, you know, play groups, supportive play groups and kindergartens, going into schooling. They want to be there and we see a lot of good will and a lot of work, teachers work hard. We have health and wellbeing teams in the schools, you know, integration aids. There's definitely support there, assessments have been… I had one family and the youngest girl, it was picked up that she had speech pathology issues. She had a stutter, or we call it a bump, in her language, a bump. And we… the department was certainly offering some support around that but not enough and we didn't want her to fall through the cracks. And it's crucial to have early intervention, especially with speech. You can imagine how that stutter would impact on your own self-esteem and being able to verbalise your needs and wants. Fortunately I was able to link through a really great specialist from (NAME OF LOCATION) Health who was as concerned as I was and we were linked to (NAME), who is one of the leading specialists, speech pathologists in the region. And she provided her services pro bono. And that little one went every week initially for six months and then every fortnightly and now she's three monthly and it was fantastic. And (NAME) was also able to utilise for her own research as well working with CALD communities for, you know, as a workshop. She works with students.

And it involved working intensively with her mother and daughter to speak in her own language and then in English. So the daughter could speak English, she was only six, but she could practice in her own language. So they were able to do that to iron out the bumps, as we said. She made fantastic progress. But that kind of intervention is needed and that's not always available I'm afraid in government schools. They try very hard but it's not, it's not available. But that's what's needed. But we were able to achieve that.

Interviewer: Alright. That sounds a good outcome in that instance.

Respondent: It was.

Interviewer: So overall, I mean you mentioned a lot already, but what do you think the key challenges migrants that you work with face while adjusting to Australian culture and settling in Australia?

Respondent: I think all that I've spoken…

Interviewer: It's just more overarching.

Respondent: Yeah, overarching. Gosh, I think to allow, yeah, allow them to be, and that might sound strange, but they come with existing knowledge and skills and solutions, they do. It's not always factored or recognised or allowed for. And I think that's hugely important, I think it's very important.

Interviewer: So working with existing capacities? Existing skills?

Respondent: Absolutely. Yeah. And recognition for, you know, the journey that they have had, their settlement journey.

Interviewer: So who would be recognising that? The general community or government?

Respondent: Absolutely, the government, across three tiers of government, I don't see why not. Existing services certainly would have recognition but don't always have the time or wherewithal to do it, they don't have the resources. So I think giving that community a voice, allowing time to settle. We know it might be just on five years when they're just maybe taking a breath. They're so focused on the doing. You know, it's not easy to get a house, it's not easy to get an income. It's not easy to learn a language other than English. And you're also dealing with parenting issues or family issues or looking after someone or a child with disability or even your parent with disability. I've seen that, where I've had families that are looking after both, huge, these are huge issues. And as a society, we don't allow time for healing. You know, you're here now, you should be grateful, do it, get on and do it. And it's huge. It's massive. And they're doing their level best, they really are. And as a community they're doing, tapping into their own resources as well. But sometimes it's just allow time to be, to take that breath, get settled, and, you know, not be kind of fearful about am I going to have my Centrelink income or is that going to be compromised because I haven't done X, Y, and Z.

Interviewer: So how could you facilitate this to happen, providing that space?

Respondent: I think I'll need a shift in viewpoint, policy change, and appreciation of what our settlement communities are doing. And they're doing great work right now already. But I think just giving that recognition and allowing that, you know, recognition to filter through and supporting them, not demanding of them or from them because they will give back. They're already doing that. But over time to allow, yeah, allow them to just be, to give that, to give that space because often those personal issues are pushed to one side and then it's not until you get to the five years and they finally have OK, you know, we're settled, we're familiar, we know where to go, we know what to access possibly and might have to do it through my child because I still don't have English language skills, but I have a fair sense of… and they swap information as well. And they have their community, you know, supports too or organisations. But again, I think that's where the personal stuff comes up that hasn't been dealt with yet and that can have a real impact. And so early intervention, support, support for families, is very important I think. And they will give back in two-fold, you know, two-fold.

Interviewer: Alright. So that's the end of the interview. Is there anything that you would like to add?

Respondent: I think there's enormous good will in the community. I came from a meeting today so I'm still feeling that great… it was specifically about safe haven enterprise visas and, you know, particular challenges for that group. We had a very good meeting, people coming together. It was organised very quickly and it was just I guess the barriers to delivering, who's actually going to deliver this, you know. We've lots of great ideas but it needs to be formalised and I think we will get there and we will do that. But again, we all have our own commitments with our own organisations, time poor, conscious of funding, we need to divulge to what we are funded for, and so it's there. But I see great good will in the community, both formally and informally and intercultural connections. And I think regional areas are fantastic for that, they just… because they've had to, it's not there always for them, so they've had to create their own solutions. And they've done that. Little bit more support around that would be great, or recognition. Decentralising services for our communities because they are living, they're living out and beyond and that's problematic. You know, and so I think greater recognition and understanding, a little bit of kindness would be nice. Yeah, maybe I'm an idealist but it would be nice if we had kindness.

Interviewer: It was a pretty positive thing you said, we can have more kindness. Alright. Well, thanks very much for your participation and all your expertise and experience. We really appreciate it. Interview end at 3:40.
