# Supplementary material for: The importance of information acquisition to settlement services literacy for humanitarian migrants in Australia
Source: PLoS One. 2023 Jan 6;18(1):e0280041. doi: 10.1371/journal.pone.0280041 (PMC9821785; doi:10.1371/journal.pone.0280041)
Supplement: S1 Data — (ZIP) [file pone.0280041.s003.zip › SP_08_NSW.pdf]

Interviewer: OK, I'm at (SERVICE NAME) with (NAME) and (NAME), and it's 12 minutes past one on the (DATE). So for the purposes of this research, when I use the term newly arrived migrants, or migrants, I'm referring to people that have arrived, living in Australia for five years or less, and it includes migrants and refugees. Alright, so the first set of questions are around the services that your organisation provides, and we'll talk specifically around your casework for newly arrived migrants. So could you start by telling us a little bit about those services or programs.

Respondent: OK, I'll start with a program actually funded by the Department of Social Services through SSI, and the Settlement Enhancement and Transition Support is the name for the service we provide. So we have bilingual workers to support the support, because the people who have arrived newly in Australia, they definitely need the language support because they are very, mainly very poor in their English language and other skills. So through casework and by having information sessions, we impower them to be independent in Australia, to make their own decisions and to contribute to the Australian community positively. So that's our work at the moment. So we do casework and we do information sessions, and also we encourage them to participate in the main events, like for example International Women's Day, Harmony Day and Refugee Week events. And we have different programs as well underneath. Community capacity building also another program under sits, so we are covering that area.

Respondent: In addition to that, those services are being provided after one year of their arrival in Australia.

Interviewer: Is it after the H.S.P.?

Respondent: Yes, proceeded from there.

Interviewer: For one year?

Respondent: Yes. They're generally, we refer the clients to the other in kind programs, like families, youth, aged care, NDIS, which is within the centre.

Respondent: So it's kind of internal stakeholders, we are referring them to them internally as well as externally, to make a positive outcome. Ours is accountable collaboration and excellence of values, so we work according to that, because we are accountable to the first client to come and raise the issue with them, and then we do the excellent service by doing our part and also collaboratively linking with the other service providers. So that's a positive.

Interviewer: And that's the next question actually. Talking about who you collaborate with, so would you like to talk a little bit about those main…

Respondent: Collaboration? I'll talk about internal collaboration, because we, for example, one client came yesterday, that's a previous client, but he came for the employment. So he heard about the employment project, and I referred him to employment accelerator program. And different stakeholders are coming to

provide jobs to the refugees and migrants on Wednesday, on Thursday at TAFE. So I am referring him to that program. He just finished the high school, and referring, and he'll possibly get the job. So they were able to make a resume for him, and other stuff. So that's the internal. And also if there is any aged person come, we refer them to the aged care people. And if they have any children, we refer them to the family support to raise their children positively in Australia. So this is the internal and also the external stakeholder we collaborate with.

Respondent: With legal aid for example, if they have some legal issues, we refer them to legal aid, and also to TAFE, to Centrelink, and we have quite good network with those stakeholders. So we work together to solve clients' issues.

Respondent: And another thing we left out is the financial support we provide. We have a, what is that, linking or partnership with [inaudible, 05:21] and also IPAR providers and Telstra company, and then past program with Sydney Water. We have a partnership. So we have trained to give vouchers to the needy people, because when they arrive in Australia, they have financial hardship with the rent and other stuff. So it's financial thing with the external stakeholders, is very helpful for the new people.

Respondent: We do at least twice a week…

Respondent: Providing electricity vouchers, and then electricity, Telstra and water twice a week. We take ten appointments each week, and we take five for financial assistance, giving food vouchers and other stuff for needy people.

Respondent: We prioritise our clients first, and then to the mainstream clients.

Interviewer: Thank you. Are there any organisations with whom you choose not to work with, and why?

Respondent: We haven't come across any we choose not to work with. No.

Interviewer: OK.

Respondent: And also we work with [inaudible, 06:43], the domestic violence, we refer clients to [inaudible 06:44] And also (SERVICE NAME), if there is any clients we come across with the domestic violence.

Respondent: And also other communities, for example Afghan Community Association, normally I keep in touch with them, in order to refer the clients for some Afghani and Iranian celebrations, or any community events. We work with the communities as well. The Iranian community, Pakistani community.

Respondent: And Arabic community as well, the Arabic Association, Tamil community.

Interviewer: Thank you. Are you aware of any services that are needed, but not available?

Respondent: Yes, there are some services that for example, translation interpreting services for private company, for example insurance company. The client has, for example, has had an accident and he wants to explain it to the insurance company and they cannot use TIS for these purposes, because TIS is specified for specific services, not all the services. Or for example tenancy, if they want to talk to their real estate agent, and they cannot talk to them, there's no one, they have to go and find somebody within the community to translate for them.

Interviewer: What, it's not one of my questions, but just out of interest, what would be the cost for somebody to employ a private interpreter?

Respondent: It's about onsite individual cost is more, $250 to examine [inaudible, 08:45]. And other than that, I don't know how much. It's reminded me to tell about the Domestic Violence Advocacy Scheme, you know it? When a client comes for interview, they have to face the court. So when a client goes to there without any language support, it's hard for them. Only the interpreter comes, but he's the official interpreter. Not from the Domestic Violence Advocacy Scheme. It is better to have a bilingual worker with the domestic violence program. So that is missing at the moment, bilingual support in the court.

Interviewer: Thank you. And are you aware of any services that are over utilised, like there's a long wait, or high demand for it, for that service?

Respondent: Centrelink is the one. Family Tax Benefit applications.

Respondent: And also citizenship.

Respondent: Immigration is the most wanted one.

Interviewer: And on the other side of that, programs or services that are under utilised, there's not a good pick up?

Respondent: The same, mainly my clients are coming for their citizenship applications, although they are out of target, over five years in Australia, still there is not a service for them. So they return back to us. So they come back to us and ask for help, and here our hands are tied. We can't help, we can't spend more time with them. And when they call the Department of Immigration, they just hear the same one response, wait and wait. There are some applications that have been lodged since 2015, and still they are waiting.

Interviewer: Wow, wow.

Respondent: Yes, they are frustrated.

Respondent: The clients target group we service five years, but when they apply for the citizenship four years, the outcome has to be within one and a half years. So four years finished, and they are coming after one year, there is no outcome from the citizenship application. So they need to come to us to find out something about their, how it is going. So we are the one following up with

the citizenship applications. So if they close the gate at the time of five years, where will that client go? They can't go anywhere, so we need to extend the gap for the refugee people. Even the tremendous delay in the citizenship process. So we need to extend the time period to serve, so that at least seven years is the recommended time, not five years. Five years is very short at the point of how the thing is going.

Interviewer: Who has suggested seven years?

Respondent: No, I'm just…

Interviewer: Oh you're saying it.

Respondent: Yes, we are saying we need to keep them up to seven years.

Respondent: The thing is, the ten years I've been in this office, we notice that there is a service gap, and we keep raising it, raising it, it hasn't reached to the authorities.

Interviewer: And maybe that H.S.P. stage should be separate from the next stage.

Respondent: Yes, so if it is one year with them, another five to six years with us.

Respondent: Earlier it was about six months, now it has ended up one to one and a half years. So when the client is officially exited to us after one and a half years, then they have three and a half years only. So it's not enough for us to serve.

Respondent: Especially my clients who have come on 200 refugee visas, or migrants on the spouse visa. Most of them, they haven't done any schooling back on Afghanistan, so they are illiterate in their own language, and it is very, very difficult for them to catch up English within five years, and to be able to find a job to learn. But still I can see the passion in them, that they just want to learn something, or do, or contribute to the society. A lady who has never done any schooling, she even now, when we give her the attendance sheet to write her name, she just copies from her Centrelink card the name, because she's unable to write it with confidence. She copies her name each time, and I notice, and she is very talented lady. She is a mother of five, came on 200 visa, and she wants to do sewing, to do some…

Respondent: Business.

Respondent: …yeah, business, but because of language and there's no one around her to support her, so she's just stuck. And she keeps coming to me, and she says, I just want to run a business. I put my garage to use it as a place, she found a friend of hers, that she is also good at sewing clothes, for dressmaking as well, especially that Afghani style dressmaking. And we are here to support her, so while we are in the planning of forming an association, a group and registering the group, and after that to go ahead for applications for funding support.

Interviewer: Excellent, great. What's the next question here? Yes, can you tell us about the methods you use to measure the effectiveness of your programs and services?

Respondent: Mainly our monthly report is the one that we highlight our success in there. And also some challenges that we face. And M.P.R., which is monthly performance…

Respondent: Monthly performance review.

Respondent: …review, we talk about how we go for each program, for each project that we run. We measure that, and also…

Respondent: We have a C.D.S. system, and at the beginning, when a client comes, we do take an assessment, what's their goals and what's their intent to get an outcome from us. And then there are scores in that one, satisfaction score and circumstances score…

Respondent: Goals.

Respondent: …and goals. So we take that score, initially when we assess. And when the client comes repeatedly, from time to time we map their progress. So it's going from one, two, three four, and then at the end five. So within that six months' period, we can assess from zero, to two, they will have come to four. Today they will be homeless or they are applying for the housing, and then the housing will give them a house after three months, and they are secure in the house for up to six months. We give the score up to five or four after six months time. So that's how we measure the outcome.

Interviewer: Great.

Respondent: And also we write comprehensive case notes about all the successes.

Interviewer: Thank you. So the next few questions are around how migrants and refugees adjust to Australian culture and society. Can you tell us about your understanding of how migrants you work with understand Australian culture and society?

Respondent: Yeah, it is mainly through SSI and after that to us. So we provide information sessions on different services for them, and we encourage them to come and attend. Although they have the English classes attendance challenges, and Centrelink that they have to attend their English classes, but we try to allocate those times, catering for them, for example on Fridays, they don't go to classes, so what we do, we organise our sessions on Fridays so they can come and attend. So they'll learn about other services. They learn about Australian society.

Interviewer: What's an example of something you do around that?

Respondent: Excursions.

Respondent: Exact name of the program? We have a support program.

Interviewer: Just some ideas.

Respondent: We have an Afghani social group.

Respondent: I have a Tamil support group who meets every first Friday, and the third Friday. So we do info sessions like Australian system, OK, like last Friday I have done what ombudsman N.S.W., who is ombudsman in N.S.W., what they can hear from you, and how do you make the complaint, and other stuff. So before that, I booked in for information sessions with TAFE, so that's the education and employment part of their program. So something like important topics, time to time. Likewise we do the Australian culture, and also we celebrate events, and Harmony Day and other stuff, Refugee Week. So in that situation they will know.

Interviewer: Great, so what do you think some of the issues and challenges are for migrants, around the process of integrating or adjusting to Australian culture?

Respondent: First of all, I want to talk about this migrants, rather than refugees. Those migrants who come on spouse visa, there is a service gap there. The challenge is that the spouse come here to Australia on a temporary visa, and she is not, for example a woman, is not aware of her rights in Australia. So she comes in without any benefits from Centrelink, so she has to wait for two years in order to become, now it says four years.

Respondent: Centrelink benefit, two years.

Respondent: Two years. Alright, so she has to wait. So within these two first years, she only is able to attend English classes. Some families, they don't let them to attend English classes to learn about her rights, so she stays home. By two years, she will have a child. Then if that woman is not supported, if that woman, hundreds of them here, and they are not aware of our services. Because on the point of entry, nobody introduced those services to them. And usually the in-laws are trying to prevent her accessing those services. And then after two years or three years, or even four years, the family and domestic violence issues come across, because they have to take their child to childcare, or they want to go and study, and the challenges start from there, because they cannot drive, they have no friends. And I know one of my previous clients, who's been here in Australia since 2008, she hasn't even applied for her citizenship, because she didn't know. When I talked to her in a park, just by chance, she says I have no idea, what are you talking about? I don't have my passport renewed, I have to ask my husband, I don't know whether he accepts it or not. Now she has, I think four or five children of school age, and it's very hard for her, for her example. So with migrants, I suggest that at least there should be a kind of induction for those ones who come to Australia. For example, going to English classes, or somehow they should…

Respondent: Yeah, the same thing to my clients also, because my clients are mainly from migrant backgrounds, spouse visa people. But they will expect a husband to

support them to introduce the services. But what happens, sometimes the husband, he doesn't have any time. The wife stays there for some time, it's her honeymoon period, she is happy. And after six months, she is feeling isolated and then out of the, but no link to the other services. They come to us with how to apply for P.R. and other stuff. So they don't have the computer skills, they don't have the language skills, and they need to apply online, so they don't know anything. So that's another issue for them. And also the birth certificate, children born and how to apply for a birth certificate, they don't know. So the languages and isolation are the challenges for the migrant people.

Interviewer: Thank you. The next set of questions, and you've already touched on it, around migrants' sense of belonging and inclusion in Australian society. So can you tell us about some of the programs or supports available that help enhance migrants' sense of belonging and inclusion?

Respondent: We organised an excursion on mental health week celebration. So we get those clients to a park and there they go the opportunity to make friends with others and to overcome social isolation and know about Australian native animals. So they felt included within that.

Respondent: And also during events like Harmony Day and Refugee Week events, we don't hire any external people to come and give programs, dancing and [inaudible, 23:38] and drumming and other stuff, we choose people from our own country, people who are talented in that one. One African will come and do the D.J. and Arabic group will come and do the dancing or fashion parade, and then drumming from Afghan, or something. Tamil people from dance, so we actually make them come to the stadium and do the performance. So they're also showcasing their talents, and also kind of inclusion in the society. So that's Refugee Week and mainly Harmony Day we do that.

Respondent: And also I remember last year for Ramadan, Afghan community, they held a dinner to celebrate Ramadan, and then approached(SERVICE NAME), and (SERVICE NAME) was able to get most of the clients who are non-Muslims, they attended the dinner. And that's the kind of getting together. And also there was another program about Sydney Wanderers, tickets that were given, free tickets for our clients, so our clients attended the games, which they felt a sense of belonging. It happened only three years ago, so we would like to have a similar type of inclusion program.

Interviewer: Fantastic. And who do you think the key people are that your clients contact for social and emotional support when they need it?

Respondent: The caseworkers.

Respondent: The caseworkers are the first line of contact. They feel more trusted and feel more comfortable if they talk to a caseworker who can speak their language. And then from there, they caseworker is trying to build confidence in using other services within the centre.

Interviewer: Great, thank you. So the next few questions are around social support and health and wellbeing. Can you tell us about the types of programs that you know of, that provide social support?

Respondent: Other programs?

Interviewer: You've already talked about the vouchers and that sort of thing.

Respondent: Financial support?

Interviewer: Yeah, or other support, or housing support, counselling.

Respondent: Yeah, we have a homelessness project, so there is, it's funded from Mission Australia. So he works full time, so we refer all the clients who have any housing issues to him, and he will work with them and try to get housing accommodation, affordable housing for them. So that's one thing.

Respondent: And also, social support, and what is the other one?

Interviewer: Health and wellbeing.

Respondent: Health and wellbeing, what we have is we have a very good rapport with (NAME OF LOCATION) Hospital. And(NAME OF LOCATION), (NAME OF LOCATION) Health Service. We have, our previous team leader has gone to the other site, the hills sector, he organised with the partnership with(SERVICE NAME), International Men's Day, or domestic violence or health, (NAME OF LOCATION) Hospital tour, Hello Doctor event, something like that. So other clients were participating in that and benefiting from that. And also time to time, we invite the guest speakers from health and then give them information sessions. Last week, last month I had a nutrition program that brought in healthy eating and physical exercise, so it's a five week program with bilingual community education program from (NAME OF LOCATION) Health area. And also we time to time invite to speak on mental health from other outside cross cultural mental health. Last year we had a transcultural mental health event. Sorry, Mental Health Week event. We invited outside guest speakers from transcultural mental health and the people were running workshops for clients, so last year.

Respondent: And also with the Women's Health Week, we were able to refer a lot of ladies to breast screening program, and from there the G.P. The ones who were at risk, the G.P. got their referral as well, so they are following up, especially the one, I remember one of my clients, she was not that interested in attending the sessions, but because she had to wait for someone to pick her up. So I ask her, by the time he comes, you stay in the session and get some information. And then she put her name for the screening, and then she went the other day for the screening, and it turned out to be positive. So lucky it was at the early stage. She went for chemotherapy, and she's very thankful to (SERVICE NAME) for that, just a few minutes that she attended, and she put her name, and she said I just want to go and check for it. And she said, if it wasn't that information held, she wouldn't go until it developed severely.

Interviewer: Fantastic, thank you. And are you aware of any barriers that your clients have experienced when they're accessing these programs?

Respondent: Language.

Respondent: Yeah, that's the main thing, because we need to interpret for everything, otherwise outside interpreter, they have to book an bring it with them. So language is the main barrier. Otherwise with our bilingual workers, they can come and visit the physicians, they won't understand a single word of that, it's an issue.

Respondent: Language is an issue, and also now days, technology. A client who applies for citizenship, they have to go and pay online, and it is a big challenge. So they have to come to us in order to do it online, because they have no idea about the terms used in there, for example, e-Me account. Create an e-Me account. It's just we understand. It's very time consuming, because sometimes they don't know what their email address is. We have to first create an email address, and then create an e-Me account, and then to check if they have money in the bank to pay for their citizenship. So sometimes this procedure takes hours and hours. That's why we don't get time to have our lunch.

Respondent: One of the gaps is to get the physician, the ladies, mostly ladies come with children, and we don't have any child care to look after the children while they are listening to the physician, so it's very hard for them, for ladies with children.

Interviewer: It's definitely bad.

Respondent: We run driver knowledge test support program in our language. So I do it twice a month for them. I practice the questions with them, in order for them to get ready to go for the test and book an interpreter for the test as well. And there the same thing, the children plays around, and it creates…

Respondent: Noisy.

Interviewer: Distraction.

Respondent: The other participants have to cope with that, because this is the only time and opportunity they have to come and attend the sessions to learn about it, to get their learner licence.

Interviewer: Thank you. So the next questions are around financial literacy and income generation. So can you tell us about the programs that are available for financial literacy?

Respondent: We normally run an information session for them, how to budget their finances with Centrelink income, time to time. Financial counsellor comes and does that session. Or sometimes we do it, we develop a power point presentation and do it the simple way. And also when they come for e-power

voucher, sometimes we go through that, they don't know, they don't have any idea about what's in the paper. So just bring it for there, so we explain it to them, what they can do, and to bring down the cost of electricity usage, and how to cut the money from that one. And we assist with the vouchers, e-power vouchers online. And also if they have a big bill, we will send it to the bring your bill day. We have a bring your bill day on the first Friday of every month. The Ombudsman Advocacy Service Officer comes, the Electricity Water Ombudsman Service, from that office comes here and look after their big bills, and if there is anything they can cut down, or talk on behalf of the client, and liaise with the service provider and support for them. And also we provide water bill assistance, if they have a big water bill. We talk to the bill coordinator in the water, the coordinator of the pass program, and give them assistance for them. And also the financial assistance by giving food vouchers.

Respondent: And also what can develop out of that, another scheme that if they attend a specific hours of specific courses, then there will be some relief in there for paying their fines, especially if they have fines, to work on the development of the program.

Interviewer: And what kind of financial challenges do your clients face?

Respondent: Rent.

Respondent: Rent.

Respondent: Very high rent. Especially our clients on 200 visa or 202, one single person paying a single, one bedroom unit, it's very difficult for them to support themselves financially.

Respondent: And also the big bills, the electricity bill, I've got a client who came with $1400 with one quarter bill. I went through the bill and it sounds like to me it's a kind of a hot water system. The old houses have the electricity, heated by electricity, and then they don't have the gas line, so they use the hot water very frequently during the winter season, and then they don't cook in the gas top, it's in the electric cooker. So $1,000 when it comes for the quarter, how can they pay, with one person income? So very hard for them to do, so that's another, big bills are the issue. And also increasing cost of other expenses. Earlier it was like you can buy tomatoes for $2, one kilo, now it's going on $5 a kilo. So it's cost of living increasing for them.

Interviewer: That's right. And are you aware of any culturally specific dynamics that impact on the management of their financial demands?

Respondent: Yes, for example of we gave them food vouchers, they have to go and buy it only from Woolworths or Coles or Kmart. For example from Afghanistan, people go and buy Halal meat. There is no Halal meat in Woolworths. So there's less chance to buy meat from there. And also bakery which is Afghani bakery, and Afghan special food store, they do not accept those gift cards, for example. When I heard about the scheme, that they were in Centrelink, instead of cash they would give them cards, those cards would be a disaster for

migrants who culturally use their cultural food, instead of buying from Woollies or Coles, they go and buy it from Afghani shops.

Interviewer: That's a good point, thank you. What about other things like the gender imbalance around managing money, or sending money back home, those sorts of things?

Respondent: It's a culturally thing from my country, because men are the ones who mainly earn an income, and then they bring money to home, and the wife manages the money, but the husband keeps the income with him. So the wife has no liberty because if she doesn't have a Centrelink income, to get that money from the husband because she is relying on the husband at the beginning, so that's another issue.

Respondent: It still does in the Afghani community, they give the authority to the men to manage financial issues.

Interviewer: And are there, how do they overcome these challenges do you think?

Respondent: Sometimes it's ended up in domestic violence, because financial mainly is the reason, the husband sometimes does not pay in the end, and the problem starts. Or the children who are above 18, they live with their parents, and they think, for example the father is controlling their money. That's another challenge for the young people.

Respondent: So to want to get their independence, to get their money in their hand, they move out from the house and live independently, and then the other issue comes with the peer pressure and bad habits and other stuff with them. So if they live with the parents, and if the parents give guidance and money, liberty to the child, they're living in the house, and then they will do, it's not like in the Australian way, they move out after 16 years. We used to keep our elder children up until they get married, with us.

Interviewer: OK, thank you. So the next set of questions are around legal support. So can you tell us about the programs and supports available for your clients with legal issues around visas, identity, inviting family members?

Respondent: OK, that's a main issue for them, because when they come as a single person, they get married, and the partner with the application, it's from $1,500, 600. Now it's gone up to $7,600. So money for the application charge is an issue. So after the wife comes, the partner, he has to support his wife, and the visa, she has to get the permanent visa later on. So they come to us to get the permanent visa application. So online, they don't know how to apply online. Now they made it online, so we need to do it for them. It takes hours and hours to finish it, and make documents. And also the website is not simplified for them to get quick access. It's long steps to go through the steps.

Respondent: Actually the system is made for Australians, not for migrants.

Respondent: Australians, they don't need the visa.

Interviewer: That's right, that's right.

Respondent: And we have an immigration lawyer coming here every Friday of the month, and she takes five appointment, and we refer clients to the immigration lawyer, so she gives advice. But this is only advice, not the paperwork. So after the advice, they will come to us for the paperwork and online application support.

Respondent: There are no such services anywhere in Australia to help with filling in the forms. Filling in the forms is another headache. We ask our volunteers to help, but sometimes the volunteers are not familiar with those forms and questions, so it ended up again, back to us. So we have to go through the form and help them.

Interviewer: What about, I know you've already mentioned the (SERVICE NAME) and (SERVICE NAME), but any programs around physical violence or other forms of violence and discrimination?

Respondent: The policy system, we run sessions in the family harmony, not in the direct domestic violence, if you mention domestic violence no client will come. So we encourage them to come for the session, we are doing a family harmony and healthy relationships. So they come for the session and they find out what to do when they have any issues, and they know how to call 000. But when they call 000 and the police come and take the husband, and she is in the home, what happens after one month? The husband comes and tells the lady, I won't do it, can you withdraw the [inaudible, 42:29] and the lady withdraws the [inaudible] and they live together, then the domestic violence happens. And she is now, doesn't go to the police because it's happening again and again. So this is the stage with the ladies, they don't go and tell out. And also if they go out, the general services are only for the English speaking people, but they can't live in the other refuges or other houses…

Respondent: Share accommodation.

Respondent: Share accommodation, without the language support. So it's very hard for them to speak out. For the migrant people, domestic violence support services are not enough.

Respondent: Not much, it's not adequate. Let me give you an example of an elderly woman who couldn't speak any word in English, even not thank you. So she, in the middle of the night, she was shivering, she was very cold, all she needed was a blanket or a heater, and the accommodation provider, the lady, she couldn't understand her.

Interviewer: Was this a refuge?

Respondent: Yeah, women's refuge. So then they had to call the ambulance. They thought she is sick, but she said she kept shouting on people, she said I need a blanket. In our language, it's kampal. She said kampal, kampal, and nobody

understood her. Until finally they called me at that time of the night. Lucky I give her my number, in case. I knew that these problems will arise. Then I told the ambulance officer, she's fine, she says she's OK, she doesn't have any health issues, only she is feeling very cold and she needs a blanket.

Respondent: Simple one.

Respondent: So much confusion.

Interviewer: That's so hard. So what do you think the key laws or provisions are that migrants need to learn in their first year, about being in Australia?

Respondent: Legal system is important one. And their rights and responsibilities. And then Australian culture, and how to raise children in the Australian way. Family harmony, healthy relationships, we need to give them that Australian thing. Doesn't accept the violence happening in the community, and also child protection. We have to inform them, because their disciplining is different from the countries they originated from, and here it is different. We need to teach them.

Respondent: And also those issues that arise after a year, normally, or within a year. So they don't use their Opal card properly. Either they don't carry their concession card, they receive fines, and sometimes they just trespass over the gate and they think it's OK, and when they receive a $200 fine, they come to us. And also to educate them not to sign for those energy companies, they ask them to come and sign up, and they end up receiving bills from different companies, and they are worried

Interviewer: Oh my goodness, wow. So the next question is around the movement of people from one area to another. So can you tell us a bit about what you think the key reasons for the movement of people from one place to another, or from one suburb to another?

Respondent: From my point of view, my clients move from Queensland, Victoria, and sometimes from Perth. This is for the cultural support, for the family community support, because Sydney has lots of Tamil people living around, and also worship places, lots of temple and other stuff. So they move from there. And also for the job opportunity. Here you can make a self business, restaurants, and open up shops, retail shops and other stuff. And also you can establish a cleaning company also here, it's a very diverse area to get employment successfully created. So they created their own jobs here.

Respondent: And also from Coffs Harbour, many Afghani women who are [inaudible 47:15], they are placed there first and then they said we felt discrimination, direct discrimination because of language. They were scared to report it to the police, so they just sneak out. They said we had to come to Sydney where our friends are, somebody at least we know. And we feel safe in here. And now, this new lodgement of visa, it's very encouraging and I hope it works, to shift people to other parts of Australia, regional parts of Australia, but there is a challenge that one of the visa criteria is they have to know an employer there

to sponsor them to go. And they have no one. Even those people who have family members who want to come and work in regional Australia, they don't have any connection with that rural area.

Interviewer: Surely they would have realised that? It's quite impossible.

Respondent: Yes, it is.

Respondent: People move from suburb to suburb also, another reason is for children's education. Because for example, people move to, they don't live near (NAME OF LOCATION) area because they think the local school is not good because there's lots of problems in the schools, gangsters and other stuff. So they move from that area to another area, maybe (NAME OF LOCATION), or maybe (NAME OF LOCATION) or (NAME OF LOCATION) or something, they will move. And then they have to face the increased rent in that area. So education, because we focus on education, so the children discipline. They don't have money to send for the private school or Catholic school. So they move from suburb to suburb for the child's education. There is any good school around (NAME OF LOCATION) has a good school there, so my people move from (NAME OF LOCATION) to more (NAME OF LOCATION) area. So that is also another reason to move from place to place. And also if the temple is nearby, they move, a cultural wish for the worship.

Respondent: Most of my clients as well, when they move, because of high rent, then they find a cheaper rent further, not close to train station, and there they have got their own issues, because they can't drive, and the bus comes late, or other problems. So then they move back to close to station. So in one year, you can see twice they moved out within six months' time, and then in two years, four places, and they said all our money is gone from disconnecting the power, to reconnecting, and paying for the removalist. So these are also the problems. So if somebody, for example the caseworker, has explained to them, if you move, think of these costs, it will be cost for you in future, they wouldn't have done it. But once, after four times moving, then they realise, and they get stuck in a place that they rented.

Interviewer: Wow, thank you, that's great. So the next set of questions are around education and literacy programs. So can you tell us about the services available to migrants using your services, in terms of school education for their children, which you've touched on a little bit, adult literacy programs and any other educational literacy programs.

Respondent: Here the program, the TAFE providers is a good provider for them, because when they come new, they don't know English. They have an interview with them and they put in the level one, level two, level three. So according to that, they will go, they improve their English language from there. And also we have English conversation classes on Wednesdays for the target group clients. And computer literacy, for the groups we arrange computer literacy programs. We invite TAFE to come and deliver the program, outreach program for them.

Interviewer: Great.

Respondent: And youth also, they have their own study service.

Respondent: Homework support.

Interviewer: Oh good, great. And what do you think some of the key issues or barriers for children of your clients to accessing school or university education?

Respondent: A few of my clients' children, they go to school until Year 10. After that, they stay home, even not attending Centrelink to ask for a payment. They stay with their parents, just locking themselves inside the home. Four of my clients have got this problem. They parents say we can't do anything, they don't want to go, they don't want to get out.

Interviewer: They don't do the two last years?

Respondent: Yeah, they don't, because it's getting difficult for them. Or they get engaged with bad friends in Year 10 or Year 9, they start smoking and things like that, which is not common in Afghani communities.

Interviewer: So they don't have a transition program at the high school?

Respondent: They do, they do have it, but sometimes they fall into gaps, and they just stay home.

Respondent: The same with me, my clients are, I can't say many, but some of my clients got into the bad habits, and then they are addicted to drugs, and the parents are getting trouble from them. And they stopped the schooling. The other people, the parents, the problem is language issue for the parents. They don't know what is happening at school, and how to liaise with the counsellors and other staff. So when a child is problematic, they have to prevent it at the beginning. So if it is gone for a long time, they can't stop it. So the language issue, and also the gap between parents, the generation gap, is the main issue to stop them mingling with the bad friends.

Interviewer: OK, thank you. Can you tell us about any special packages or subsidies provided to support educational opportunities?

Respondent: They can apply for scholarships, because time to time, (SERVICE NAME) opens the scholarship program for refugees. We encourage them to apply for it. But mainly our part is the settlement, we refer to the youth team, and the youth team has more contact, more links with the (SERVICE NAME) and they will encourage their youth clients to apply for the programs.

Interviewer: Thank you. Are you able to outline the kind of employment opportunities that you are aware of that are offered to migrants' children when they finish school or university?

Respondent: For the children? We are not much into youth programs, because they have their own support programs for their employment opportunities. The case

worker is working very hard, the youth case workers, to get them involved with the resume and face to face interviews with the employers. So as much as I am aware, because I refer my clients who are young, and they want to look for a job, to the youth project, and I see they follow up with the client, to see how they progress, and if they get a job.

Respondent: From time to time also we run information sessions on the employment pathways and education programs. And interview skills, and resume making programs as a part of the information sessions for that group.

Respondent: Even sometimes we individually help clients to develop their resume letter, or what you call it, cover letter, and encourage them to go ahead, not to be scared. It's just an interview. So it's one to one support also we do provide.

Respondent: It's the caseworker will show them how apply for jobs online, and cover how to address the cover letter, and how to attach a resume online, when they apply for jobs. We teach them the skills.

Interviewer: Great. And overall, what do you think are the key challenges that migrants you work with face, while adjusting to the Australian culture and to settle in Australia?

Respondent: Language. Housing, finance. Those are the main ones.

Interviewer: What did you say?

Respondent: Culture.

Respondent: Generational gap, intergenerational gap.

Respondent: Those are the main challenges, especially language. Language is the main challenge. The first thing that for example they come across with their neighbours, and they don't know their neighbours, and it is a big trauma for them, because back home, they know at least 15 houses, who lives, how many people live, what they do. But here, they are even scared of saying hello, because sometimes they don't get the reply back, even saying hello to them. So the problems start from the neighbourhood, and then it continues. Back home they were offering food, but here they say, they might sue you if he gets sick, don't do that. And they step back, not to socialise with others. That's why I'm saying culture. Otherwise if it was language, if they could speak, body language could make a relationship with their neighbours, but here they feel lower. They don't feel that confident to have a connection with the neighbour. That's why the trauma starts from there.

Interviewer: And how aware do you think the migrants that you work with, or refugees that you work with, are aware of the services that are available?

Respondent: Earlier, when they come to Australia, it's a takeover. So they introduce the services to them. And when they come to us, mainly they know the basic services, at least Centrelink, TAFE and other legal services to them. And then

when they come to us, we introduce the services when they get the issue. For example, [inaudible, 58:45] they come with the bill, they don't know where to call, what to call, how to tell, what to pay or something. So we will tell them, this is the recovery centre, and it recovers money from you. If you don't pay money, they will send it to the other agency, they will charge you more. So we are the main people introduce the other services to them.

Interviewer: How big do you think word of mouth is, between…

Respondent: Very strong.

Respondent: Very strong. For example, I go to an Afghani wedding party, and I see a young couple come to Australia on a refugee visa for example, or on a spouse visa. And I go and I start talking to them and I say look we have these services. And if you want to drive, come on, we help you. We have a class. So from there, the word of mouth is really strong. And then just today a client of mine came and she said, this lady she is a new arrival, she has no idea how to do her my-Gov account, and she came to my home, I've got my kids, I referred her to you, she might come tomorrow or on Thursday to see me. And also because of this gap of over five years, services for over five years, what I do, I already established an Afghani women's group at the park. So they are over five years in Australia, and they get together once a week, every Wednesday at the park, and I voluntarily help and support them. So what I do, whatever services we have for general other mainstream clients, then we explain it to them, and then they talk to their families and friends, and whoever, new arrivals, and then they refer them to us. Word of mouth is very strong. And especially these groups that they get together. There is an Afghani senior men's group. They get together once a week to overcome social isolation, and for the women as well. It started with seven or eight women, now it's up to 50. They get together. And how we empowered them, for example I started with Viber, so teaching them how to use Viber and be a part of the group. And if the ones who cannot read, I record my voice on the next program. So our program will be in this place and we'll do such and such activities. So they are now mastering using Viber or WhatsApp group.

Interviewer: Excellent. And finally, what would you like to see as possible solutions to helping or support migrants to adjust well to life in Australia.

Respondent: More bilingual workers in all the services, like in Centrelink and CALD for the people to access the services like us, more bilingual workers.

Respondent: Yes, and also job or work agencies also should be, they should have this cultural…

Respondent: Competency.

Respondent: What's that, cultural awareness, so they know how to deal with those clients. For example, client of mine, they say we are asked to go and look for a job. They've never touched a computer before, so they say, I sit in front of the computer with a blank mind for one hour, because I was told to, to go and sit

for a job. I've no idea how to look for a job. So she says I go and sit there for one hour and then I leave, so I can sign that I've been there.

Interviewer: Oh my goodness.

Respondent: So those services should have some kind of mercy or leniency or understand the situation of those newly arrived ones.

Respondent: And also the complex problem starts after five years, or after they're established. The women get the words to speak up after four years, five years. So if there is domestic violence happening, or anything happening, it [inaudible, 1:03:34] and then she comes out. She comes out when she wants to speak about the domestic violence, she comes to us, she is out of target group. It's five years. So what can we do? We have to send them to the general services, like police or other general services, but they don't have the English language to speak out, and they don't understand the culture of the lady who comes from saying that she experienced domestic violence. So that is also another issue, people who have complex issues, when they become out of target group clients.

Interviewer: Now, it's not within the questions, but I was thinking around mental health. How do you support people around the stigma around mental health, or what are the sort of services you would refer them to?

Respondent: First of all, I attended a training which I recommend for all my colleagues to attend about trauma. So we should understand between the trauma and mental health, some people are just traumatised and they need some time to heal. But we label them as a mental health issue. So first we have to train ourselves to identify those, and diagnose actually to see if the client is really mentally ill, or just gone through a trauma and needs time to heal. And then without any judgement or perception, you just go and work with them and you will see the result. Even with children, when I say that at school, a child misbehaves, and then they label them as ADHD. That child may have gone through a war or a trauma, something, and he hasn't told that time. So training is important, and also more programs for inclusion. For example, camping. Before that, we had this funding opportunities to get 85 or 75 women, and their daughters to a camp for two nights.

Respondent: Now it's only cut down to 20 clients.

Respondent: The budget to 20 clients, but still that 20 clients, only for two hours or three hours excursion. So within that two nights camping, we were able to get all majority of the services from family law to fines to education and TAFE. So they come and talk to them, while they're all camping, we're investing.

Interviewer: Fantastic.

Respondent: So the regular outings and the excursions are the main thing for them mentally, because they establish friendship and rapport. So friendship is the main thing for them.

Interviewer: Thank you, that's excellent. That's the end of the interview. Is there anything else you'd like to add?

Respondent: Thank you, thanks for listening to us.

Interviewer: I really appreciate your time and your lovely insights, you've given a lot of great information that will complement what we've already heard from other people, so thank you.

Respondent: Thanks a lot.

Interviewer: So this is the end of the interview, at 19 minutes past 2.

Respondent: It was a long interview, wasn't it?

Interviewer: Yes, thank you.
