# Supplementary material for: The importance of information acquisition to settlement services literacy for humanitarian migrants in Australia
Source: PLoS One. 2023 Jan 6;18(1):e0280041. doi: 10.1371/journal.pone.0280041 (PMC9821785; doi:10.1371/journal.pone.0280041)
Supplement: S1 Data — (ZIP) [file pone.0280041.s003.zip › SP_08_Victoria.pdf]

Interviewer: So this is (SERVICE NAME). And the time is 10:45. Sorry, I should just write that down. And with (NAME) and (NAME). So…

[CHAT]

Interviewer: The first thing, before we start, I just wanted to clarify that we're… because obviously you run a bunch of services here, so aged care and community services, so for this we're specifically referring to the settlement services for new migrants, so voluntary and involuntary for the first five years settled in Australia. And just as a side question, do you work with any voluntary migrants here? Provide services for voluntary migrants, like economic or family reunion?

Respondent: You said voluntary, sorry, what do you mean?

Interviewer: So as an economic migrants or family reunion?

Respondent: Yeah. Family reunion, yes, but not skilled migrants. Not skilled migrants.

Interviewer: Family reunion, I presume refugee?

Respondent: Yeah. So we're funded to deliver settlement services to refugees who arrive either through family reunion or through the Refugee Special Humanitarian Programme and you know, other visas like the Women At Risk Visas. So there's sort of a range of visas that we are funded to support. But yes, skilled migrants doesn't come within that scope.

Interviewer: Excellent. Alright, so the first set of questions are about the services that are provided at (SERIVE NAME). And this can be obviously education and health, social, legal services. So can you tell us a bit about (SERVICE NAME) settlement services? And you don't have to tell us specific programmes because we'll ask a bit more after about the specific programmes. But can you tell us a bit of a background?

Respondent: So I guess in terms of our services, (SERVICE NAME) has… well, it was formerly (SERVICE NAME) because (SERVICE NAME) is the merged entity following the merger of (SERVICE NAME) and (SERVICE NAME). (SERVICE NAME), I guess specifically in relation to this, has had a long history in delivering settlement services to refugee migrants, over 35 years of delivery services. And in terms of the scope and where we deliver those services it's the (NAME OF LOCATION) region of Melbourne, (NAME OF LOCATION). But our, I guess, greater concentration of our settlement services are within the (NAME OF LOCATION) region as well. OK.

So I guess in terms of what we are funded to do, I mean we provide a suite of settlement programmes and those programmes I guess support clients, families, communities from arrival and generally up to the five-year period. In terms of the services we provide within that scope, we

do, we provide case management support, and within that we obviously work with individuals and families. So I guess it's a case management family support worker around particular issues, whether it might be with things like, you know, employment or health, it might be about family relationships, parenting, you know, health literacy, access to information about rights and responsibilities and civic participation. So a broad range. I mean, in accordance with… I'm not sure if you're familiar with the Settlement National Outcomes Standards?

Interviewer: Yeah.

Respondent: Yeah. So I guess within the scope of those topic areas, we would provide whether it's case management support or we'd provide group information sessions. We run, within that we also run a number of group programmes. So we might run play groups, homework support programmes, and I guess those sort of platforms allow for individuals, families to connect. But also it's a platform where we disseminate information about particular topics that the groups themselves identify as an area of need. So you know, it might be around, you know, how to get a driver's license, it might be accessing employment or accommodation, health services. So that's the vehicle in which we, yeah, we provide and disseminate that information. We do that with, I guess, partnerships with other organisations that perhaps specialisations in those particular areas, whether it's legal services or health services, we will engage with those providers to come in and speak to the participants of the programmes. And I guess in doing so what we are also fostering is, I guess facilitating, is a connection to that service provider because we… in some ways it's like a warm referral. We, I guess, make that connection and through the introduction then, you know, our clients, our participants of the groups and programmes will make that connection and if they need to access the service they know where to go and who to go.

So, you know, I guess in sort of, in terms of facilitating that independence, that's the way we would do that. We also are funded to work with communities under capacity building initiatives where some communities, I guess, have the capacity and an interest in establishing their own association and with that then, you know, we'll seek out support through, I guess, you know, identifying potential leadership programmes that they need in terms of being able to achieve that. We will sort of facilitate governance training, you know, we will provide them with opportunities and, I guess, support them through funding applications if that's something that they have a particular need within their community that they're wanting to address. We will support, you know, individuals within the communities to apply for funding. And if they don't have all the relevant DGR status and incorporation and so forth, as an organisation we also agree to auspice, those communities. So we will enter into the agreement with the department or the funding body and then work closely with the community in their delivery, you

know the planning and implementation of the project, and support them.

I mean, I guess, we do that with the view that we work with the community and in that process we coach and mentor as well. So, you know, within that scope we work with, you know, a number of communities who may have an interest in sort of establishing language, cultural programmes or recreation programmes for communities. You know, a number of women's programmes we run in collaboration with various communities. So in doing so what we, as I said, it's really much about trying to facilitate that sort of independence and within the community associations to be able to, I guess, establish and make those connections with providers, funding bodies, yeah. And I guess we try to do that in a respectful way that, you know, whilst yes, we are the auspice, we are a partner in that relationship and not the controller or the… I mean, we've got obligations as an organisation because we've got the agreement with the funding body but we try to do that with communities in a very collaborative way.

So, you know, if I can give an example, a recent example that we're sort of currently in the processes, we supported a community that is looking at establishing recreation programmes for young people within their community and, you know, we've sort of supported with the application process, we are the auspice. They're looking at recruiting so we're doing that together with the community and, you know, letting them drive a lot of the decisions around that. But again, using that sort of platform to coach and mentor communities as part of their own development as well.

So receive funding both from the Commonwealth and the state. And within the state programmes that we run, again, they're very much focused around capacity building. So it is very much working with communities, talking through in terms of having sort of an inter-cultural dialogue around what their issues and what their needs are and I guess working together in terms of assisting them with facilitating sort of a prioritisation of what they're wanting to address. And I guess being a bit of a broker in that relationship. So if there's issues around justice, you know, being able to bring those providers in because we've got those connections, we will bring the providers in and I guess support that relationship building as well. And so very much working with communities in that space.

We also have a family violence project as well. Again, supporting communities to I guess identify responses that will prevent violence against women and children and how best to manage that. So that particular programme again is funded by the state. And also within the settlement space but outside of settlement services, we are funded through… we are funded for the Adult Community Further Education Programme. And so that's a pre-accredited English as well as prevocational skill development programme for migrants and refugees

as well. So I guess it's an entry point for clients who have an interest in I guess reconnecting with education and employment pathways. So it's a starting base as well, yeah.

And the other one is associable enterprise. So this is something that's an initiative that came through the work that we do in settlement, working with communities. There was a sort of a demand. I mean obviously employment focused, but a strong interest around gardening and agriculture and looking at how we can support communities and members of the communities to develop those skills. So the (SERVICE NAME) enterprise programme was something that was established in about, I think it was about 2015. So it was prior my time, before my time. And there what we're providing is really opportunities for migrants and refugees to engage with training and around gardening and cleaning and for us to support employment opportunities, whether it's… well, at this stage it's primarily internally. So we've got two teams, one in the southeast and one in the northwest, small at this stage. But the team in the southeast are currently doing the gardening services for our residential aged care sites and in the northwest we have a number of clients that are… the team is actually supporting clients in the community, so the sort of private residencies that they would go out.

So we're looking at expanding that and in doing so it's looking at, again, connecting with education and employment providers in terms of expanding that opportunity. So I'm currently in negotiations with a TAFE provider to see whether we can develop a training programme that is targeted specifically to I guess the needs or where the communities are. Because obviously the English literacy is a challenge and participating in education, there's a barrier around that. So looking at how we might be able to facilitate that and expand that opportunity as well.

So I guess in terms of what we do, you know, we are obviously… we receive funding to deliver services and we do that either as a direct relation with the funding body, which is the Department of Home Affairs, or we are a subcontractor to AIMS for the delivery of the HSP, which is the Humanitarian Settlement Programme. So we've got varied… I guess what I'm trying to point out is that we have various relationships with the funding bodies, rather a direct, as I said, a direct recipient of funding from the funding body, whether it's in Commonwealth or state, or we are a subcontractor to a lead agency within Victoria for the delivery of, say, the HSP programme.

Interviewer: Alright. That's a lot of things it sounds like.

Respondent: Yeah.

Interviewer: Are you aware of any services that are needed that aren't really available for new migrants?

Respondent: That's a very broad question. In terms of services that are needed?

Interviewer: Yeah. So I guess can you see any kind of gaps in terms of needs that new migrants have that a service could be, whether it's a health need or financial or legal need, that there's kind of, there could be some kind of service delivered to help them?

Respondent: Look, I think, I think there are a broad… I mean I think… broadly speaking, there are services. Whether we're talking employment or accommodation, you know, there are services but there are limitations within those services, that's where the gaps arise. So, you know, so if we're talking about employment services, yes, there's a Jobactive, but it's the processes of that funding… I mean we're not talking about providers per se, but the barriers and the gaps are created because of policies or, you know, the way… there is a disjointment sort of between the different sectors. So, you know, there is… I mean I guess under settlement services there is a strong focus around education, employment and English, the three Es, in terms of being able to then achieve participation. But when the three… you know, when we work together, and, you know, there are efforts to try and work together, but the way settlement services are funded and the way employment services are funded, that causes tension, this tension between those funding structures or service models that make it really quite challenging. And that's where clients fall between the crack. Do you know what I mean?

So, you know, talking about, you know, family violence for example. You know, if we're talking about… some of the challenges there again is we know that for clients, you know, for women to make the decision to leave a violent, domestic violence situation, you know, it's a significant, I mean it's a significant decision and it's a difficult decision for anyone. But for a woman from, you know, from… you know, you're in emerging communities, there are all the other complications around, you know, the isolation that they can experience, you know, the family composition and how that sort of will work. But the services, if the woman doesn't… you know, so often what we will see is, you know, women might be in that situation but may be really hesitant to leave. But until she makes the decision to leave she's not able then to access the service. So then she's stuck. You know, stuck in a situation where, you know… so you can yes, there are options, but there are no real options for somebody who… you know, there's really examples where, you know, a mum in a domestic violence situation, she may have children under the age of 10 but she also may have adolescents, well refuges don't accept, you know, adolescents. So what does she do? You know.

So I guess I'm highlighting those as examples where there, you know, there are a broad range of services but I think from our experience, what we see, it's either the policy context or the funding model or the

definitions and the scope of services that don't meet the needs, whether they're cultural needs or the individual needs. And often then what happens is clients then decide it's just too hard. You know. So… and so part of our work there is again trying to facilitate those, you know… like I said, I'm working closely with education providers. I've also been trying to work with Jobactive providers to try to build that understanding and that connection and looking at models of how we might be able to do that on the ground because some of these decisions, you know, I would say (?) it's policy. And look, certainly our role is to advocate those issues through our peak bodies or, you know, government departments. And they're well aware. I mean there's been a… I'm not sure if you're familiar but there's the Shergold Report that came out, so that was only last week. So there is constant, you know, review. And I guess even in that report what's come through is, you know, there is, the coordination between settlement services, the coordination between employment/education providers, that's where the challenges are. You know, that's where the challenges are.

The fact that we are, you know, we work with clients up to five years but, you know, we've obviously got the established relationship so what happens after the five years as, you know, as we all go through life stages, when there is a new issue that emerges we tend to go back to what we know. And so often we will see clients outside of the five years. They come back to us and representing and so, you know, whilst we're not in a position to support them we, you know, we will try to provide some information. But we're limited. You know, just because somebody's been in Australia for five years doesn't mean that they are settled. You know, and some people settle very quickly. Some people, you know… like, and, you know, pre-arrival experiences, you know, English literacy, if they've got a qualification in something. You know, there are factors or variables that enable somebody to settle more than others, quicker than others. You know, obviously there's different sort of stages. But some people don't ever settle. You know, they struggle.

You know, so… and, you know, the expectations that… you know, there are gaps around… you know, if you're talking about interpreting services, we talk about access to information, you know, in a written, the written communication of information, well, you can provide it but at the same time, if people are not literate in their own language…

Interviewer: Yeah, it's very hard to become literate in another language. So are there any services that are over or under utilised?

Respondent: Services. So I can't… I mean I could probably just… I guess from my… I have to try and think in terms of… I mean I think maybe a different sort of way, I mean, what we're hearing… I mean housing is a significant issue. Housing is a significant issue that seems to keep coming up as… you know, people don't have stable accommodation then, you know, there are other flow on effects within that. In terms of,

you know… I'm just trying to think. I mean in terms of [indistinct 22.32] networks, I mean housing seems to come up… housing comes up, employment services keep coming up. But again, the employment services is that it's the system that's not working. It's the system that's not working for the groups. And look, and the departments, the Department of Home Affairs, as you will probably see within the Shergold Report, that comes up as a feature, is that, you know, the department is looking at innovative ways of how to improve that. Looking at flexible models around employment pathways. The other one that comes up also is English. You know, with the AMEP programme, new arrivals have access to 510 hours. Again, some of the challenges are that if you've got mums, you know, caregiving responsibilities, being able to access those programmes can be really quite difficult. So again, the Shergold Report talks about that and looking at, you know, the department's keen to look at ways of making those sort of programmes more accessible for mums or others.

Often what we have also, what we hear from communities, because of course when you look at the AMEPs 510 hours, it's classroom setting. But then there's also been the obligation with, you know… and there's been a change to that, with the 13 weeks in order for them to get the income support, you know, that sort of responsibility. So there's a push that they have to find a job in 13 weeks but then they also have to do the 510 hours. So I mean, fortunately that issue has been raised and it's been taken onboard. I mean I think it's been pushed to… that 13 weeks pushed to 26 weeks. And I think as of next year it will be pushed to 12 months. So it means that, you know, a new arrival can participate in the English and not needing to do the jobs, sort of search requirements, until 12 months after arrival. So it gives that time. Because of course, if somebody doesn't attend their English classes, English which is essential to being employable, yeah. So there was a tension.

So I guess an under utilisation of the AMEP English hours because I guess people want to work. You know, they come here, they want to work. Yes, it's important that they have English, they recognise that, but if they have to prioritise, they prioritise work opportunities than AMEP. So again, the Shergold Report will talk more to that as well. So… and look, health services, and again, you know, if I think about the southeast because prior to coming to (SERVICE NAME) I worked for an organisation, an MRC in the southeast. And so I have sort of a familiarisation with the providers. I mean, you know, you have your large health providers like (NAME OF LOCATION) Health and (NAME OF LOCATION) Council being heavily involved in the planning and delivery of services to refugees and migrants because that is the main population. So I guess what I would… the point that I guess I'm saying is that I think there are also differences. So I can only give a perspective around perhaps the (NAME OF LOCATION). And I know in the west too, you know, there are strong drivers from councils and health providers as well. But my sense is that in the

southeast, you know (SERVICE NAME) Health has been a significant player in the space of delivering health services to refugees and asylum seekers. Yeah.

So I guess there are variables within the region and within catchments too. So when I think about is there… you know, I think (SERVICE NAME) Health here plays a very significant role in health literacy and health support. Yeah. And you've also got, you know, if you're talking about family relationships, you know, there are family support organisations, mainstream, that work with a client group. But again, some of the models there in terms of being able… because access has been an issue. I mean, you know, it's how do we make services accessible. So there are arrangements like service agreements. So the organisation that I worked previously had, was subcontracted by a mainstream family support organisation to deliver services to the communities. So I guess… so there are models, I guess, in terms of trying to improve access to ensure that there's no under utilisation and people are connected to the services. Is this, is this the type of information you're needing?

Interviewer: Yeah. Well, yeah. Yeah. I guess those questions were more about, sorry, if there's services that are on/off, which there are, you're doing case work management, you know, there's health literacy, there's, you talked about the domestic violence programmes. So when we're thinking about all those services, are any of them in particularly high demand or are any of them… you know, are new migrants saying, we need this, this is what we need, we need more of this? Or is there anything that's being offered and it's just not really being used?

Respondent: Look, I think… look, I mean I think… yeah. Through our case work and group work, I mean we obviously will provide information around those topics. You know, driving is a high demand. Yeah, because obviously with driving, you know, if you can drive then you can be connected to the community, employment, and the like. So driving keeps, is sort of a high demand area. We run a number of women's programmes and I guess within that it is very much promoting also I guess topics around women's health, parenting, communication and relationships. And, you know, with our youth programmes, I mean obviously education but also, you know, with young people it's looking at, and what we're probably needing, it's more around youth leadership and programmes that promote that, is certainly another area.

I guess a high demand area which is becoming increasingly difficult for clients to access is around migration advice and the family reunion. And so that has then implications because, you know, often we will have somebody who arrives and within the first two weeks they will say, right, I want to bring my wife or my children or… and they themselves are not established. So there are challenges around that. You know, previously immigration advice was something that was funded through the settlement programmes but that's now becoming

less and less a reality. So the charges, you know, for fees and if somebody was to go out and get private migration advice, there's high costs associated with that. And I guess with all the changes to visa and, you know, for some family reunion's not going to be a reality at all. You know, so family reunion and access to migration advice is a significant issue.

Interviewer: Alright. So the next question's related to how migrants are adjusting to Australian culture and society and what challenges or issues they might face. So can you tell us about your understanding of how you see new migrants adjusting or understanding Australian culture and society I suppose?

Respondent: You know, the big question, because I'm sort of representing, you know… obviously you'll be speaking to the communities themselves, but I think in terms of my perspective, I think… on the point that I raised earlier, I think communities and individuals, you know, for each and every one it's a different experience. And there are, you know, variables that support individuals or communities to adjust better or quicker than others. And so some of those things, as I mentioned earlier, were around, you know, if people… I mean certainly if we talk about, when we're looking at different communities that we support in the west, for example, you know we work with the Burmese community, we also work with a Syriac, Syrian community. And obviously as communities they, you know, the Syrian community often have come with I guess a high level of English literacy potentially or they've had education. So their opportunities to settle in some ways or to adjust is… it's easier, not that it's easy, but it's easier because they have an understanding of some of the systems and processes.

Interviewer: And so that's what this question is kind of getting to. Like are new migrants understanding the systems and… because I mean Australian culture, I'm not really sure what that is today.

Respondent: What that is, yeah.

Interviewer: But…

Respondent: But if it's about navigating the systems, I mean look, I think… I mean that's part of our role. I mean, you know, so there's an ongoing need. I mean, as I was saying earlier too, that, you know, while we're funded to support refugee communities up to, you know, in the first five years, often what we will see is there are some communities or individuals that keep coming back because they still require that support. And so, you know, the navigation is always a challenge and I think barriers to that is around, obviously, the English literacy, you know, providers not utilising interpreting services, you know, to be ensuring that their services are responsive and that they're being able to understand what the particular needs are. So yeah. I think… I know. I just think the question's a bit broad. I don't know…

Interviewer: It is. And I mean it varies and unsurprising this has been the response from everyone. It depends on who you're speaking to. And not just the cultural group but also the people within that cultural group. Each individual family might have different capacities or different drive to engage more with Australian culture.

Respondent: Yeah. Or, you know, there might be linkages. You know, like if there's… so, you know, for example, I mean obviously as part of the settling, often where communities are settled. You know, like in (NAME OF LOCATION) it was always, you know, there's been a sort of high prevalence of the Afghan community. I mean in the early 2000s it was South Sudanese and then the Afghani community as well. So if there are strong links within the community, sometimes there are community members that support that role and that function too. You know, it's where… but then, you know, you're relying on communities to know things and some are better at knowing than others, and don't. So, you know, it's variables. So the adjustment I think is subject to the length of time, somebody could be… so I think, you know…

Interviewer: The length of time they've been in Australia?

Respondent: In Australia, and the linkages that, you know, people have or individuals, families have. You know, their… yeah, if they have… but also I guess it also… you know, it's not necessarily just about the individual. It's also about I think the service system around and how aware and how willing they are, you know, how inclusive they are. So, you know, I talked about, you know, (NAME OF LOCATION) for example.

Interviewer: So universal as well as settlement services?

Respondent: Yeah, yeah. Absolutely. And how they all work together too. So it's not only about the individual. It's about that whole, where they're at and how inclusive their own community is. And you know, I mean, you know, I can certainly… you know, if I talk about, you know, the southeast, you know, I could say… as I said earlier, you know, you have key providers, significant providers like (SERVICE NAME) Health and (NAME OF LOCATION) working very closely with, you know, the(SERVICE NAME), that's where I was previously, and other settlement providers within the region, the Asylum Seeker Resource Centre. So a really strong network. You know, there was Medicare local as well at that point in time that there's all been funding changes to. So if you've got a network of services that are prepared to work together then that also facilitates the settlement of… so it's not only about the individual's capacities or community's capacities, but it's also about the capacity of the community to support and be inclusive and, you know, to respond to those particular needs.

And so, you know, if I compare both, like if I sort of say the southeast and the west, I mean I think certainly in the west as well you have some significant drivers there through local councils and health providers. You know, as well as, you know, there's West Justice, you know, the Western Bulldogs that we work with as well. So, you know, if you… it's that network of… that collaboration. And that's important. Because we, as a settlement provider, can't do it all. And you know, our role, I mean I think our role is about providing those linkages, you know, supporting communities and families and individuals to make connections with the mainstream society. I mean that's essentially our function. It's about being that broker. Yeah. So it's a loaded sort of question. You know, there are so many… but you know, my sense is that, again, just broadly speaking, I think there are some communities that settle better than others and some of those variables that I spoke to that I think facilitate that.

Interviewer: And so do you see that new migrants still have opportunities to practice their own culture as well?

Respondent: Look, certainly from our experience, yes. Because we, you know, we… when I talked about the capacity building, capacity building initiatives that we have, it is very much about supporting communities to respond to what their desires and aspirations are for their community. And so, you know, we work with a number of communities in the establishment of cultural and language support programmes. You know, or running multicultural festivals and events. You know, the state government funding that we receive through capacity building, I mean there are many grant opportunities that is about promoting, you know, individual's culture and, or community's culture and language and support that and celebrating that too. So yes, in many ways I think those opportunities are available. Could there be more? Possibly, yes. But certainly I think, you know, the Victorian Multicultural Commission, the department, the premier, and cabinet, you know, provide specific funding around promoting that diversity and… but also, you know, local councils. Even local councils through their community grants programme. So these are some of the… so these are some of the funding opportunities that we support communities with applying for and delivering. So through those grants, at local government as well as state government, the VMC or the DPC, you know, there are opportunities for communities to apply for.

And, you know, I was speaking to Bunnings yesterday. I mean it's sort of, a bit of a diversion. Talking about, you know, it's not only certain providers but I was talking to Bunnings about the Hope@Hand programme and seeing how we might be able to work collaboratively together, whether it's coaching, mentoring, or whatever else it might. And even Bunnings in their own community involvement programmes provide workshops or spaces for women to come together and, you know, they will provide like a facilitator or one of their staff members

who might be interests in crafts, for example, to deliver a craft session to women of new and emergent communities. So I'm just using it as an example where mainstream, I mean that's Bunnings, but a different… you know, I think that's an example where there are providers outside of settlement that are also thinking about culture and promotion of, you know, if you're talking about gender equality and supporting refugees and migrants. I mean they do a broad range of things, but that was an example of what they do.

So there's probably… you know, there are philanthropic organisations also provide funding opportunities for, you know, for vulnerable, you know, new and emerging communities as well. But, you know, a lot of… you know, I think also churches. You know, churches also.

Interviewer: So the next question's relate to migrant sense of belonging and inclusion in Australian society. So do you have any programmes that specifically support migrant's ability to feel that they belong or feel included in Australian society?

Respondent: Well, I think again, it's very much through the work that we do. You know, the work that we do in our capacity building, it is about, you know, providing opportunities for communities to I guess work within their communities and respond to their community issues and to then be able to advocate for those issues. And I guess our role there is very much around brokering and facilitating those discussions or meetings or whatever the case might be. So it's through the initiatives that we support communities with.

Interviewer: So does that make them I guess have enhanced sense of belonging in Australia?

Respondent: You're talking to a social worker so I was thinking in terms of belonging, what does that mean. But anyway, look, you know, people will say, yes, they feel connected to Australia. You know, they feel that they can promote and talk about their culture or they can talk in their language. You know, if we're talking about the language programme, you know, one of the groups was saying that through the delivery of the programme they are feeling more confident speaking outside in the broader community in their own language whereas previously they had hesitations to do that. So look, I think there is, you know, I think through our settlement programmes we aim to promote that sense of belonging and inclusion and participation. And we try to do that. I mean the way we do that is very much working with communities and the organisations that they need to reach out to build those sort of common understanding. So some of our… so codesigning responses to, you know, or initiatives that will ensure or aim to improve access or a sense of belonging and participation.

So I guess that is what we… I mean again, settlement services is about making those connections but it is about ensuring that people can

participate both socially, economically, politically as well. That they can participate in society. And to do that, if they need English literacy, well we've got the English classes. Or you know, we will make referrals. I mean that's how we would… you know, we would work with providers, make referrals. We would either, you know, for some clients, depending on where they're at in terms of their adjustment, is, if they, you know, if they need physical assistance to access that then we will provide that, for others it might be just a contact. You know, so again, that's what I'm saying, it's very, very broad. I mean what we're talking about, that is the aim of settlement services but we would be working with individual's capacities and then identifying whether we do it through case work and we advocate through our case work or we advocate through our peak bodies or, you know, ministers or whatever. So we will be advocating some of those issues.

But also, if there's opportunity for the communities themselves to advocate on their issues then we would do that. So in the west we have a network, the My Community Voice Network, which is represented by I think 50 communities, and there it is about, you know, them coming together and talking about their issues and working with other multicultural communities, where required the department coming in or other providers coming in to hear those issues. So again, our role is very much around brokering so that people and communities can achieve that sense of belonging and understanding, again, through the work and the practice, we're demonstrating the process, you know. So it's…

Interviewer: Yeah, makes sense.

Respondent: So we don't, yeah… I mean we don't…

Interviewer: It's through all the things you do essentially. Your aim is to increase sense of belonging.

Respondent: Absolutely. And again, you know, if you ask me, I think, yeah, there'll be some clients that do and some that don't and some that will never do. You know, like it's… that's… it is a personal perspective. You know, I can say that as a provider we are funded to do that. And this is where I think, you know, the DECs (?) reporting, I mean that's some of the things. We don't capture that information, we've got anecdotal experiences. And, you know, who will say, you know, I feel proud to be in Australia, I feel safe living in Australia. Others will say, I don't feel safe living in Australia, you know. You know, I think a lot of the media, policies. And so those things are outside of the control of providers, outside the control of individuals and communities. So in fact, we might… and that's the challenge as a provider, is that that is what we promote but then we can also receive resistance. And resistance not only from broader community but resistance also could be because, you know, the competitive nature of the funding as well.

Interviewer: So competitive between providers?

Respondent: Between providers. I mean I think, you know, I think that's improving, that's improving, but it's very competitive. You know, I mean the last round of funding that was released, now it's 2018, you know the department made changes and, you know, some organisations, you know… so settlement programmes were funded as a collective. So your case work, your group work, and your capacity building was one settlement programme for the department under this new contract. And this is for not the on arrival. I don't know, are you familiar with the suite of services?

Interviewer: A bit.

Respondent: OK. So there's the HSP, which is the first, from arrival to about 12-18 months, and then there's the SETS, settlement engagement from that sort of 12-18-month period up to five years, and in that space we've got a direct funding relationship with the department. But the department separated the capacity building as one grant opportunity and the group work, and the group work and the case management is another activity. As a provider, we receive funding in both, which was great, not a lot but great. But, you know, other MRCs who have had a long history received…

Interviewer: They didn't get funding for capacity building?

Respondent: Yeah. And so the challenge there is that sometimes a capacity building… sometimes… because there's such a fine line between the group work that we do and the capacity building because sometimes it's through the group work that we do, say, with women's programmes, that we might say, well, you know, there are women here that are starting to talk about leadership opportunities. And from that then we move it into capacity building or vice versa. You know, through the work that we do with capacity building we might identify a need around, I don't know, driving, for example. And we will do that through that. So that's an example where, you know, changes in funding then impacts on the relationships that we have also with communities and what we can and can't do and adds additional barriers. I mean also within that funding, if we're talking, and this will be an interesting thing to monitor over time, is that under this new contract interpreting services, where we used to have access to free interpreting services…

Interviewer: Was that through TIS?

Respondent: TIS, yes. TIS. Now we're… it's a budgeted item. So of course in order for us to do our work, you obviously rely heavily on interpreting services, but we also work with communities and, again, part of that building capacity, bilingual support. So we, if we work with communities and there are leaders within the communities, we will

obviously look at how we might be able to, yeah, engage with community members to do some of the work with us. I mean obviously there are sensitive issues we won't be doing that sort of interpreting. But, because there's challenges around that too. But you know, we often recruit members from the community as bicultural workers to support some of the programmes and activities that we do, which is about encouraging that participation and connectedness as well. So under this new contract, so there's a concern that if, you know… I mean if we're looking at a budget item, it's about $100,000 a year. That's impacted on… you know, that's something now, that's $100,000 that we've actually to map (?) on, from the budget. Whereas something that we had access to… and so the concern is that, you know, providers are not going to access interpreting services.

Interviewer: Well, yeah. That's a very reasonable concern, right.

Respondent: Yeah. And again, that's been advocated and raised to the department. But, you know, it's these sort of decisions that impact on people's access or people's sense of belonging or a sense of adjustment. It's some of those structural challenges that impact on that. So yeah.

Interviewer: So do you run any programmes that look at or support health and wellbeing?

Respondent: Yeah. So we will run, as I said, information sessions. But they may be run through a women's group, right. So we might… so we've got… an example there would be that in the west we have the relationship with cohealth. And we will invite cohealth to run. So again, it's part of that codesign. If the women's group are saying, look we're needing information on A, B, and C, then the team member will then identify which provider needs to come in and deliver that training. So yes, so we will run health. If it's, you know, financial literacy, you know, we've got West Justice that come in.

Interviewer: So that's the next…

Respondent: OK. So yeah, so that's what we do. I mean, that is what we do.

Interviewer: So this is health literacy programmes?

Respondent: Health literacy, financial literacy. You know, if we're running around family relationships, absolutely. I mean around those nine topic areas of the national outcomes, that's what we'll provide as part of our scope of services, whether it's information, whether it's referrals, whether it's advocacy, whether it's…

Interviewer: Yeah. So in these health, in this health and wellbeing kind of information, health literacy programmes that you collaborate and that you run, what works and what doesn't work with that are you seeing?

Respondent: So look, I think… so one of the things that I've sort of talked about is that running… so to say, look, we're going to run an eight week or two-week health literacy programme and send it out to the community and expect communities to come to a workshop, things don't work. But if you're working with a group and there's a safe space so that where the information gets disseminated, and the higher the sensitivity of the information I mean obviously it becomes more critical, but you have more success if you're working with a community or if you're working with women or young people, it's about then talking to them, you know, building the relationship. You first have to build a trusting relationship in order for them to be able to share what their concerns are. So I mean obviously that's… I mean our work is very much relational. And then through that dialogue they will talk about, oh… and they may not even, they might not even identify that, oh I think we need information around driving, for example, or we might need somebody to come and tell us about the legal system or whatever. But they might say things like, oh, you know… and you know, I've got a fine in this particular area.

And so the work that we do with the group and the work that we do with the case manager and if there are particular themes that come up, that's how then we start then the conversation. Say, well, you know, you talked about, you know, you got a fine, it's your second fine, or you know, somebody else in the group might say, yeah, look, I did too. Maybe a parking fine or driving fine or whatever. So sometimes you have to do sort of the thinking around, well what might be this particular theme and explore that with… so you can't just sort of say, this is what, this is what the group, I think, needs. It's actually that codesign approach around… and gathering the sort of the themes and the trends, whether it's through the group work or the case management that we do that we identify what those themes are and then talk to the community about, you know, what their thoughts are and what information they might need around that. So… and then we would invite the guest speaker in to talk about that.

So that's… so in terms of deciding on what to cover in the topic is something that really needs to be I guess related to what the community feels that they need. Yeah. And sometimes they're not able to articulate that because if they don't know what they don't know. So if we're hearing… so we're sort of that, we're hearing and listening and then presenting, this is what we're hearing, this is what we think, what do you think? What else do you need? And so involving them in that process rather than just, we're going to run this, you know, a six-week parenting programme, but not really targeting what the issues are for that community. The time of when those sessions… so if we're looking at a group, I mean, you know, if we're wanting to, you know, ensure that people participate and access, you know, ensuring that the venue's accessible, transport's available. You know, if there are children, you know, that need to be… because obviously if somebody's… sometimes if it's a small group then it's OK but if it's a

large group then you might need to organise like child supervision so that parents can be, you know, parents or individuals, mums or dads or individuals can pay attention to that. Obviously access to interpreting. I mean I'm giving you practical sort of stuff, is that…?

Interviewer: Yeah, no, that's fine. I'm just going… because also I'm just taking, because now, this question was about health and wellbeing services, but I'm just, these are all what works and what doesn't work for, it sounds to me, all the kind of programmes and services.

Respondent: All the programmes.

Interviewer: Whether it's about financial literacy, whether it's about legal issues, whether it's about health. So yeah, no, so that's right.

Respondent: Yeah. And I think, you know, I think… you know, the other thing is also, you know, the information needs to be accessible. And when I say, in terms of not only available in writing but, you know, as I mentioned earlier that you need to understand if, you know, the literacy level of the community you're working with in terms of their own language. There's no point giving them something in their language, which is great, if they can't understand it. So ensuring that the information is in an accessible format to the community is really, really important. And that's not only, as I was saying, related to just being in the language. And… but also being able to explain some of the concepts too. So again, when you're sort of translating concepts, there are no, there are no like concepts. So being aware that some concepts are not understood in other… Western concepts are not understood within their cultural context.

You know, I was sort of thinking, you know, it's something a little bit unrelated, but packages, for example, the home care, aged care packages. People talk about packages, packages, but if you say what packages are, you can say it, [speaks foreign language 60.12] in Greek, but what does that mean? It's still not… it's not still known, understood.

Interviewer: [indistinct 60.17] as well.

Respondent: Yeah.

Interviewer: I should just mentioned that we're at an hour already, which is how long I said this would take, and we've still got a few questions to go. So I don't know what your timeline is like. I imagine you're busy if you've been away.

Respondent: Look, I can… another 15 minutes, is that going to be… do you think?

Interviewer: Alright. We'll have to go through it quickly.

Respondent: Let's see how we go, let's see how we go. OK.

Interviewer: So can you tell us briefly then about any financial literacy or money managing kind of services you run or programmes you offer, sorry?

Respondent: So again, we will do that in collaboration with West Justice. So, you know, again, I mean if you're… I mean basically it's around managing money, bank accounts. I mean I don't have the particular details around the topics but, you know, I'm aware that we have an existing relationship with West Justice as well as Women's Health in the southeast, no, Women's Health in the west, that we run financial literacy, money management programmes. But, you know, we also invite Jobactive providers in that space too because obviously there's… and the Department of Human Services around Centrelink and… so we would bring in… again, depending on what the group identify and obviously [indistinct 61.48], we will then bring the relevant partners around the table to provide information that, it might be around their income support or their benefits or their bank statements, understanding, to be able to read their bank statements.

Interviewer: That's good. And how about programmes that focus on legal issues? Do you have any of that or is that in partnership with West Justice?

Respondent: Well, that's with West Justice, for example. Yeah. So we would do that with West Justice, and again, some of the things that come there is around… OK, yeah. So it might be around fines, you know, how to respond to fines. It could be about court hearing. Look, and I know that in the inner Melbourne there's a project that the team members run there because the team members are based at the Juvenile Justice Centre in (NAME OF LOCATION) and there they have a partnership, again, with the centre where they run programmes where particular women can come in and they get a tour of the services and who's… like it might be police, it might be… I mean, obviously our workers are there. You know, in terms of looking at the court and understanding the court system. So they're being provided information about understanding our court system in Australia.

So again, it's very much in partnership. We, you know, we… our role. again, is very much working with communities, hearing what their issues are, and then facilitating a process where they either connect or refer or they come in. You know, we coordinate a session, whether it's for an existing group or, you know, separate. But it's primarily more through the existing group. Because the success of getting, you know, running a programme and just communicating it broadly, that doesn't necessarily work.

Interviewer: Alright. So the next questions relate to the movement of your clients from one place to another, so mobility. What are the key reasons why people you work with might move to different parts of the…?

Respondent: Well, housing affordability, employment. I mean they're often the main reasons. Yeah, housing being the first.

Interviewer: Any trends that you're seeing at all?

Respondent: So trends that we've seen. So basically what happens, and again, and I can talk about it from both the southeast and the west, is that, you know, even if we look at our office in the west. So our main office is in (NAME OF LOCATION) and we have another office in (NAME OF LOCATION). And we've got some collocation outreach posts in (NAME OF LOCATIONS) to make sure that services are accessible. Now what we're seeing is clients have moved from (NAME OF LOCATION), they're moving more out to (NAME OF LOCATION) and more to the outer (NAME OF LOCATION) areas because of the affordability. In the southeast, what we're seeing is because when, in sort of the 2010 we were seeing a number of males from Afghanistan coming to (NAME OF LOCATION) and settling in(NAME OF LOCATION). But when they were reunited with their family they were then, what we were finding is they were moving then to the City of (NAME OF LOCATION) or to the City of (NAME OF LOCATION) because then the types of housing… so it's not only about the affordability but it's the actual stock of the house and accommodating large families and the like too.

So in terms of trends, it would be one around, as part of that initial settlement they might come to a hub, particularly if there's… and if it was just a male. But once they've reunited with family and they wanted a bit more space and obviously the costs would mean that they'd be moving further out into the outer regions. That, again, provides a challenge around, you know, obviously transport. So families, for example, I can talk about (NAME OF LOCATION), and I'm sure it's the same in the west, is that the transport system was not, you know… the availability of public transport for women who wanted to come from City (NAME OF LOCATION) TO (NAME OF LOCATION) was quite difficult. Particularly with large families as well. So the infrastructure, the transport infrastructure is… so whilst, yes, it might be more affordable, you know, there are other then barriers to sort of this participating or engaging with community if the transport infrastructure isn't available to support that.

And in the west I think, you know, similarly I think it is around the accommodation but it's also… I think certainly some of the new suburbs that are coming up, the transport infrastructure is not available there. Yeah. And, you know, I think about sort of cases in (NAME OF LOCATION). So often mums in (NAME OF LOCATION), so as they would move to (NAME OF LOCATION), if they, again, who are further isolated in terms of not being able to access public transport, and often sometimes what we would hear is risk taking behaviours. They would drive without licenses.

Interviewer: Yeah, right. To come into (NAME OF LOCATION)?

Respondent: Yeah. Yeah. And, you know, I think, you know… you know, we, at the time, had also the council were saying, well, you know, you need to deliver the service where the families are. Say, if it was (NAME OF LOCATION), that's fine. But… so we were… of course we would support that and we did have a site there but what we were finding is particular women would prefer to come to (NAME OF LOCATION) because we were located to the (NAME OF LOCATION) market. They'd come to (NAME OF LOCATION), do their shopping, drop in if they had a letter that they needed some assistance with understanding. But we had to say, well no, we'll come and meet you at our (NAME OF LOCATION) office. But they said, but I want to come here. You know, and so we had this sort of advocacy because it would mean by the time they went back it would be after five. So again, I'm just highlighting those as examples where sometimes as providers and as funding bodies we might have good intentions but it's really got to be driven by what the community and what the clients need.

Interviewer: Yeah. Alright, so do you run any programmes, literacy programmes, English language programmes here?

Respondent: We do. Yeah, we do. I mean look, like we do it through the ACFE, the Adult Community Further Education Programme, but we also run conversational classes as well. Yeah. Because I guess one of the… some of the feedback that we had from clients and communities is that, yes, they do have access to the 510 hours but for some that's still quite advanced. They need that sort of pre-step. You know, prior to sort of engaging into a classroom setting. And increasingly I think that English literacy happens… I mean, if we talk about language development, it happens through being immersed in the language. Doesn't happen… I mean it happens in the class, it can happen in the classroom. But being in community is… and hearing and practicing. It's when you go to the shops, when you, you know, go to the school. You know, that's where they learn and pick it up. So providing those opportunities, that sort of more informal conversational opportunities is something that we promote.

And obviously, and there are a number of other providers. I mean a lot of the church-based organisations also run, you know, services and English conversation classes as well. You know, the point that I was raising around the Hope@Hand and my intent to connect with the TAFE is about how can we develop the training programme around gardening and horticulture in a way that's in context and in language for the team members that we are working with? Because they don't have English. They're shown… and how do we go through an assessment process rather than, you know, sitting down and being a test? Can it be competency based and demonstrating, being shown and demonstrating that they understand what it means to, you know, OHS within a gardening context. So for us, I think… and I think that's

something I think again that the department has taken onboard. I mean it will be interesting to see what changes. I mean they're talking about changes to the AMEP, which is the Adult Migrant Education Programme. It will get changes around that. You know, I think hopefully they've understood that it is about that being in context and when people are engaged in an activity and there's conversation, that's… it's when they see it and hear it and feel it that they understand. Yeah.

Interviewer: Yeah, for sure. Alright. So overall, what do you see as broadly the key challenges new migrants are facing in settling well in Australia?

Respondent: Well, OK. So broadly speaking, the things that we've talked about. I think some of the structural challenges, you know, I think is a challenge. Just repeat the question again.

Interviewer: So what, broadly, what do you see as the key challenges for new migrants in settling well in Australia?

Respondent: The key challenges. I think… I'm sort of struggling between that and bringing it down. The key challenges. Yeah, look, again, I think it's some of those structural… I'll go back. Some of those policy structural are barriers. You know.

Interviewer: So it's the actual, this set up of how we are dealing with new migrants essentially that is a challenge?

Respondent: All the service system and how we, you know, how we're working together. And again, you'll see that's come up as part of the Shergold Report. I mean it's been an ongoing issue around that. So the integration of services and how they come together. You know, we… you know, we, for example, I'll go back to the case of the family violence sort of provider. I mean we will provide that sort of early intervention information, work with individuals around, you know, developing, keeping, ensuring that they're safe, it's safe to plan, we will do some of that work. But sometimes they need that in-between and unless they make the decision to leave, they're not going to get any support. And so that then causes a problem not only for… but also for a provider like ourselves in that we're then carrying increasingly complexity around that. So it's a systems and some of the processes that are barriers to people sort of settling, new and emerging communities settling.

You know, there's a lot of stuff around, you know, the… because I guess I'm looking at the external rather than the internal. Like, you know, we know what the challenges are internally around literacy and, you know, for the individual. But I think that sort of civic society around, that supports, is that if the media… I mean, the narrative around refugees and asylum seekers is not positive. And so that could be a barrier to new and emerging communities settling. Because if

they're not, if they're not welcomed in their community and connected then they're not going to settle. Do you know what I mean?

Interviewer: And it impacts policy as well.

Respondent: Absolutely.

Interviewer: Because if you have a public support for certain actions or if you have public, lack of public support for certain actions then they're not going to fund it. So…

Respondent: Yeah. So, you know, I think we are, you know, we are funded to do… you know, we, as a provider, are funded to do part of the work. A lot of that, you know, is reliant on working in close partnership with other organisations. Because as I said, we, you know, we're not the experts at everything. You know, and, you know, a lot of the work that we do, whether it's in health literacy, financial literacy, you know, employment accommodation, whatever, it is about connecting with the providers and building that relationship. And I think there's a lot of effort that goes into establishing those relationships and connections. And we do that because obviously that's part of our role and that's what we do to support our communities and our clients. But often that doesn't, that effort doesn't get acknowledged. You know, we get… and I'll go back to the funding. You know, we don't get funding to do that sort of work but that's critical to the success of the programmes that aim to then meet the objectives.

And so sometimes that can be too difficult and things then don't happen, and who misses out? You know, if I use again the example of the Hope@Hand and some of the, trying to look at the partnerships. I mean I've contacted five organisations and I have been trying to do this over the last 12 months. And I can confidently say that I'm making some progress after 12 months. And sometimes it's out of our control. I mean, you know, and I've also not been able to respond in some instances because I'm caught up with, you know, other priorities. You know, we don't have designated positions to be driving some of these partnerships and, you know, what it takes to actually make things happen on the ground. You know, Bunnings, it was about six calls. Social Creators (?) was about eight calls. You know, the TAFE, it's been about five. You know, and that's just one programme.

So, and again, I'm just using that as an example that sometimes things take time. To make things happen, sometimes things take time. And there are variables for that. You know, organisations are going through constant change. You know.

Interviewer: And so finally, which is kind of, which is the antithesis of this question I just asked, so you've kind of probably answered it in a way. What would you like to see as some possible solutions to helping or supporting migrants to adjust to life in Australia?

Respondent: Look, I think that work, which is around, you know… because it takes a whole community to support, you know, refugees and migrants into Australia. So I think it is about, in some ways, it is about acknowledging the collaborative effort and what that takes and, you know, other, I guess, resourcing in some ways that effort to be able to, yeah, to, I guess, to ensure that things are sustainable too or for the communities. I mean, yeah. And we can talk about, yes, more funding. But the reality is there's not going to be a lot of new funding around that. But… and you know, sometimes it doesn't take funding. You know, like I said with the Bunnings example, there's already… but it's about the knowing and reaching out and persevering and the effort to make that happen. I think there is good will. You know, there is good will generally amongst service providers and systems but I think, yeah… what would I want? I don't know.

Yeah. I mean I think, yeah. I mean I think, you know, if we're talking about some of the narrative in the community it's about promoting more positive stories around success and the contributions that communities make.

Interviewer: So for the general population to see that?

Respondent: Yeah, the general population to see that. And, you know, I know that, you know, there are efforts around doing that. But the policy is so strong. You know, the messaging from, whether it's politicians…

Interviewer: From the department that you get funded from actually?

Respondent: You know, it's… yeah, it's half-way because, you know, we were in [indistinct 82.05], you know. So, you know, you hear stories where, you know… like I was in Port Douglas and went to get some fish and the woman there started talking and she was from Melbourne and had been in Port Douglas for six years and she was talking about, oh I came to… and this is not even knowing what my background is, my work is, or anything like that. But she said, you know, visited Melbourne, I'm never going to go there again, never ever… you know, I just can't do it anymore. And I said, why? She was like, I just don't feel safe. I said, oh OK, what's… I was sort of thinking traffic, just… and she said, oh, she said, well, you know… so she was originally from Bacchus Marsh, and she said, you know, we visited, my son's wanted to go into the city and they wanted to catch the train and, you know, they talked about a group of young Sudanese boys, and my heart just sank. And I'm just sort of thinking, this is what people… like that was what made her feel unsafe to come back to Australia, to come back to Melbourne. And I was sort of thinking, you know, it's no wonder.

So it's heart breaking to hear those stories because communities are being demonised, communities are saying… you know, sometimes they will say… even through the family violence project that we're

doing, so we've got to be really sensitive. I mean, see things are sort of flowing through. But, you know, sensitive about how we approach it because, you know, we're saying, OK, we're targeting X amount of communities and they will say, why are you targeting us? Is it because, you know, the department thinks that this is an issue for us?

Interviewer: And is that what's happening?

Respondent: Well, in some cases it could be, but in this particular case, no, it was because it was more around, well which are the communities in the west that we, you know, so the most represented communities… so it was more around, who are the major communities in the region? And it had to do with a readiness to start talking about the issue. And there'd been some work that had already been done with some of these communities around talking about family violence. So we had to talk that through to say, well, no, it's not because we, you know, there's a view that it's specifically an issue for your community but we identify the community because you are the major community in the region and we've already done some work in that. They're fine with that. But, you know, there are specific programmes that have been targeting, you know, in recent years around the African young people. You know, like the community support groups, for example.

So you've got the media, then you've got programmes, and then you've got communities saying, but this is not our… you know, it's not an issue for the whole community. So there's a disconnect with what's being projected.

Interviewer: So we need some changes. What would be good is some change in the narrative, perhaps the media narrative around demonising new migrant groups or refugee groups particularly. Funding was mentioned there at the beginning, I'm sure that's everyone that…

Respondent: Yeah, and look, I mean the reality is we're not going to see many changes around that. I mean, you know, I think what's happening is we've moved towards more flexible sort of programme, design, and funding but now we've sort of gone back into rigidity, and that then… on the ground, things don't work that way. And so there's a tension around, you know, clients needing more than what we can give. And we're working with increased complexity. So yes, there's an element of funding around that but it's also recognising… and look, I think, particularly in the settlement, I think the programmes, the departments acknowledged, for example, now, that some clients receive low intensity case work and then medium intensity case work, whereas in the past it was just information and referral. It's more than information and referral. Like, you know, to sit down with a client and to actually understand what information and referrals they need you need to be able to do an assessment. So there was a bit of a minimisation around what was actually required. But I think, you know, I think… because

for a period of time the programmes were sitting under the Department of Social Services, they were integrated.

So it will be interesting to see if there's going to be any changes around that. But yeah, look, I think certainly a change in the narrative, promoting more positive stories and images of communities. Because I think when people make the connection with people they realise it's not as daunting, you know. And I've had to have those conversations within my own family, network, and communities as well. You know, so… I mean that's one that I can think of at this point in time.

Interviewer: No, it's good, I think very important. Alright, I think we can wrap it up. So thanks very much for all your time and your expertise in the area and really…

Respondent: I hope it's been helpful. I know… I mean I've struggled a little bit because I think the questions are broad and, you know, I can talk about what we do but then I've got no evidence to sort of say, well, yes, I think people do have a sense of belonging but that's been through some of the anecdotal experience, but I also know that people still don't have a sense of belonging.

Interviewer: Well, that's what, that's what the (?) the coding and analysis of all this information will achieve. Interview ended 12:14.
