# Supplementary material for: The importance of information acquisition to settlement services literacy for humanitarian migrants in Australia
Source: PLoS One. 2023 Jan 6;18(1):e0280041. doi: 10.1371/journal.pone.0280041 (PMC9821785; doi:10.1371/journal.pone.0280041)
Supplement: S1 Data — (ZIP) [file pone.0280041.s003.zip › SP_09_NSW.pdf]

Interviewer: It's the (DATE). I'm at (SERVICE NAME) in (NAME OF LOCATION) with (NAME). And we will begin the interview. So for the purposes of this research study, whenever I refer to newly arrived migrants or migrants, I'm referring to people that have arrived in Australia in the last five years. And it includes refugees and migrants. Alright, so thank you for taking this interview with us. The first questions are around the services that your organisation provides. So maybe we'll start there by if you could just tell us about the services you provide here at (SERVICE NAME) to assist newly arrived migrants.

Respondent: OK, actually in general (SERVICE NAME), we have different services. We have services for clients from age zero to the end. So we have a playgroup which is from 0 to 4, and we have PP Program. PP Program is for kids, preparing them for school. With the tutoring, materials, and it's all free. And we have Right Start also for kids under five, to recognise if there is any issues with the development of the children with their education.

Interviewer: Are all those programs available for…

Respondent: For everyone. This one in general, for everyone. We have youth program, and we have two youth programs. One for the new arrivals, just for the new arrivals. One is general and includes casework and school holiday activities, tutoring, if they need tutoring for any subjects.

Interviewer: And the young people, the very young children, they're able to access, if they're newly arrived, they can access the general one as well?

Respondent: Yes, yes.

Interviewer: Alright. Great, but not the other way around.

Respondent: Yes, not the other way, because the fund is specifically for those people, for the new arrivals. We have family workers, and it's for general, like myself, just for the CALD community and the new arrivals. And I work with the families, with the parents and the kids. Also organise for the kids school holiday programs. If they have any issue in the school, I will go with the parents. For the parents, we have different programs. We have English classes, information sessions, social groups, advocacy referrals, all these things. I casework with them. And we have the aged care program. It's for the new arrivals, and it's general, for everyone. Also they have group outings, and the package, the service they provide like helping in the house, our outings. I've been taking them to shopping, to the doctors, all the aged service. Also we have a disability service, which includes like casework and coordinating their plan. And we have what they call vacation, not vacation, what they call it? Respite house for the disability, like weekends or during the day, if the carer couldn't look after them, so we have a house for them. This one is general, we do. And we have other programs, we have emergency relief, if somebody is in crisis financially, this one is for everyone. And we have NIL, no interest loan, so this one is for everyone. So we have here and there some programs for general. For my program, settlement program, we have three workers. One for

the youth, one for family and one general, for everyone. And usually we work together. We organise things together sometimes, and sometimes we have different programs for the different target groups, I mean age. For us it's just the age sometimes. Or sometimes a single one for the generalist worker, she will look after him or her.

Interviewer: Great. And what are those programs, a bit more detail about those settlement…

Respondent: The settlement programs?

Interviewer: Especially your family ones.

Respondent: Let's say the family, usually we have the family the second stage of settlement. So they've already passed through the first settlement…

Interviewer: H.S.P.

Respondent: H.S.P., yeah, which means maybe 12 months to 18 months they've been here. So when they came, most of them, let's say most of them, when they come to our area, they are looking for school. So if they are moving from the area, the first thing is the school is very important for them. Primary and high school, so we try to help with the enrolment. If they have issues with the housing, with the accommodation, if they have issues with the last accommodation, to settle the bond, all this stuff. English class, sometimes people, they don't start directly with the English class. So maybe the wife, the mother, she has a baby. She couldn't start school, so we navigate with Novitas to contact them and organise the interview to do the assessment or the enrolment. At the same time, we give them information if they have children, for the childcare, to enrol them near the Novitas, or near their house, so it will be easier when they come. The other things, we have social group for, because sometimes they finish their class or English, or they are not fulltime in the English classes, so we have social groups. This group actually, it's running every week during school terms. Also we have the school holiday programs, so we take the whole family. What we've found, new arrivals, they don't send their youth to the programs. Because still sometimes it's a culture, or it's not a trust. So until we build the trust with the clients, usually we organise the activities together. So we organise an outing, and we have activities for the youth, we have activities for the young children, and the parents, on the same day, so everyone is happy, everyone is involved. Casework, as usual, whatever is coming. If the family have a disability or something, we follow up with these things. So actually, even sometimes very, very small things like some Muslim families, they wanted Halal shop, which aren't around in the area, they don't know where to find their groceries. So small, small things. And sometimes really I get questions like one lady, she wanted to buy flour, but she said, I went to the shop, but there is so many flours. I bought a flour in English, but I found so many flours. What's the difference between this one, wholemeal, plain, self raising? So small, small things. But it matters. So we have also in these things.

Interviewer: Great, fantastic. What are some of the services the organisations… sorry, what are some of the other organisations that you work with?

Respondent: Novitas, Centrelink, legal aid, schools, like whenever we do the classwork program. But in general, it’s internal referral, because we have the emergency relief, we have the NILS, we have the playgroup. So we refer internally. But still, we refer if we have an issue, we don’t have the service, we extend the referral.

Interviewer: Fantastic, thank you. Are there any organisations with whom you choose not to work with?

Respondent: Not really, not really. But we found the police is very hard to work with, with the CALD community.

Interviewer: Can you give me an example of something that’s difficult with them?

Respondent: The have the assumption, the stereotype, that always there is an issue, it’s always they blame our CALD community. I’ll give you an example. I’m not sure if we should record it or not.

Interviewer: It will be de-identified.

Respondent: Like the Australian husband threatened a CALD wife with a knife actually, and she came, she was very frightened. When she was talking to me, she came to me after, she was talking to me. She has one child, I think four years old. While she was talking, she said also he grow marijuana in, it’s actually not a house, they have a small garage, and she lives in the garage with her husband and her child, and the house has the mother in law and the brother in law. And there is no toilet there, so sometimes she goes and knock on the door for them to open the door for her. And sometimes they don’t let her, so they told her to do it in plastic bags. Anyway, when she told us about that threat with the knife, we contacted the police. And we went there after a few hours to collect her things, because they told her, it is too dangerous for you to stay there. We went there, they took the marijuana, it was maybe 12 plants in the same garage. They took it away, and they were talking to the husband very normal. And when I was with her in the taxi, and she took her things and went with her daughter, the police officer, he came and he told her, don’t think to run away and take your daughter overseas. And in very nasty way. Even the taxi driver, he was shocked, he said, what’s happened? Why’s he saying that to her? And he was talking and laughing with the husband. The husband was sitting and drinking his coffee.

Interviewer: Oh my God, that’s horrible.

Respondent: I really have a bad experience always with the police. A few incidents happened similar to this one. So unfortunately here, especially in the (NAME OF LOCATION) area, they don’t have the awareness, and they stereotype…

Interviewer: And that’s racist.

Respondent: Unfortunately.

Interviewer: Thank you for that, that's good. Now, are you aware of any services that are needed, but not available?

Respondent: I'm not sure if it's available. I'm thinking about the people who come with the skills, but they don't have qualifications to work. So really we have very good skills, but unfortunately they can't work, and if they work, they will be illegal. Like a painter or a plumber, trade skills. No problem if they are going to get their licence. The English is very hard. So before actually they have special courses which are more practical than theory. So they test the student while he's doing practical things, not writing things. This one was a few years ago, but now it's not available. It was a private college or something, they provided these services.

Interviewer: That's good to know, we'll look into that. And are you aware of any services that are over utilised, like there's a high demand, and long waiting times?

Respondent: Actually, what we need, immigration services, we have issues with the visa and some people, they want to bring the rest of the family. This is actually not available. We have legal aid here, but we don't have specifically here for immigration. They need to go to another area, Fairfield or something. And there is a waiting list to see the solicitor.

Interviewer: Right, so that's putting extra demand on Fairfield's worker too.

Respondent: Yes.

Interviewer: And are you aware of any services that are under utilised, so not a good uptake? People aren't using them?

Respondent: The police [laughter].

Interviewer: The police, fair enough. OK, so can you tell us about the methods you use to measure the effectiveness of your services?

Respondent: For us, it's really, our work is very rewarding, when you compare the family when they come here, and after a few years you find them, they like speaking the language, working, the kids grow up speaking English very well, no accent. This is really rewarding. This is the best result. When you see them settle and they know their way.

Interviewer: But internally, in the organisation, do you do evaluation?

Respondent: We do evaluation for a session sometimes, but not for the whole progress. Yes, we do evaluation before and after, we have to do it.

Interviewer: OK, great. And can you tell us about any other issues regarding access to settlement services that migrants are facing?

Respondent: I think the job. It's a big issue. Even for Australian people, to get a job. But for the CALD community, it's more, like I told you. They have the skills, they have the qualification to work, but it's hard for them, the process to get the job. I have clients who volunteer here with the aged team, and they really loved him. Because he's really doing very well with the people, and he was driving the car, looking after the car and all these things. And he was volunteering. When they told him to apply for the job, he has to sit for the other people with the interview. He doesn't understand the procedure and what to say. He told him, please, don't ask me any questions, just give me the job and see me. I will do very well. But this one is not…

Interviewer: Oh, that's hard.

Respondent: So he didn't get the job. Because he doesn't know the formality of the job and all this stuff. Even if you train him for the interview. But still, the language is very… but as I say, they do it, they can do it very well, but to write about it, to talk about it, it's hard. So it's the job, yeah. And even now with the job service with the Centrelink, I don't like the way they are forcing people to go and look for three, four jobs and write it down. I have one client, he doesn't know what to do, he doesn't know how to write it, and he asks somebody from the nieces or the nephews, just to write for him any job, and he'll go and give them the report. And they know. He told the guy, the staff there, I didn't do this one, my nephew did it for me. But still, it's the eligibility to continue your payment, otherwise it will cut. There is no practical solution for this. Maybe they should sit together and find a job together and try to match the job with the person.

Interviewer: That's right, I've heard a lot about this sort of thing. It's not working very well.

Respondent: No, it's not working. It's not a solution, it's just, I'm not sure what to say, but it's not getting any positive results. It's frustrating actually for the clients. He wants to work, but he doesn't know how to apply. Because it's different. First because the system is all different, and now it's different with the computer and all this stuff. So everything is different. Getting the job active people, it's not doing what it's supposed to. In the end we need to find this guy a job, but this is not working.

Interviewer: It's not working. Thank you. So the next set of questions are around how migrants adjust to Australian culture and society. So could you tell us about your understanding of how migrants that you work with, understand Australian culture and society?

Respondent: I think especially for refugees, this is from their feedback, they say here in Australia, there is a value for time and planning. Because they say, what we're used to, we don't plan. Because we don't know what's happening. If we leave the house, we don't know what's happening tomorrow. We can't plan for a long time. But here in Australia, you can plan. Next month, next year, I'm going to do this one and this one. So it's really, a few clients actually they told

me that, and sometimes, because I am the same sometimes, the same background, but because maybe I lived here for a long time, so I'm used to these things. But for them, it's something new to plan ahead. Because from where they came, we don't have this. It's something, we take it for granted. We have it, but they don't have these things. So this is one of the things, to adjust to the Australian system, to learn.

Interviewer: Good. And can you tell me about some of the opportunities that migrants have to practice their own cultural values, traditions?

Respondent: One of the things actually, the availability of the food. It's around, you can get it, especially this year it's become easier to find, the cultural food, Halal food, all these things.

Interviewer: Even just walking from the carpark to here I saw a few shops with Halal.

Respondent: Yes, before we didn't have it. Maybe just one Turkish one, that's it, but now it's opening, and even the groceries, we have a few here. And the availability of the mosques and churches, we have the Coptic church in Ingleburn, and they speak Arabic, for the Egyptians and Palestinians. And the mosques, we have a few mosques around here.

Interviewer: Great. And what are your impressions of how the cultural values and practices of migrants are being recognised and respected by the people in the community?

Respondent: Not sure. Like in Ramadan, usually we do Iftar, and we invited people, not just Muslims, from different religions. And people, some people they come and like to attend these things. And we celebrate also the Eid in the park, and we invited services and people. It's giving the clients a feeling of acceptance.

Interviewer: Thank you. And what are some of the issues and challenges around the process of cultural adjustment that migrants may be facing?

Respondent: Most of the time it's related to the kids. Because migrants and refugees, they suffer a lot, and they want to protect their kids. It's the nature, it's become the nature. So if sometimes there is some Australian culture that is not acceptable in the CALD community culture, the things about boyfriends, girlfriends, and talking about the body, this one in P.B.H., they find it in the beginning, it's very confronting. How come they talk about these things for small ears, kids. So these things. It's affecting, I think sometimes. But I think when we talk to them, explain to them, this is for the safety of the children. It's to protect them, it's not… they will talk about, like if somebody comes to me, like last week she says, I want to change the school from the public school to private school. Because my daughter, she came and said girls, it's OK to marry a girl, and it's OK to have a friend. So she says, I want to change the school, I don't want them to see these things and hear these things.

Interviewer: Did she realise that it's probably in every school? They'll be saying the same thing.

Respondent: That's what I told her, that's what I told her. Even in the news you will hear this one. It's up to you to explain to her, to talk to her.

Interviewer: I'm glad you brought those issues up, because they haven't been raised before, so that's great, thank you. So the next set of questions are around migrants' sense of belonging and inclusion in Australian society. So can you tell us about the programs or supports available that help to create and enhance migrants' sense of belonging and inclusion, cultural inclusion?

Respondent: I thought it's the same question, like the other one, when we do the Iftar, the Eid events. So we try sometimes, for us we work with the new arrivals. But sometimes we do for the mainstream also. Like International Women's Day, Refugee Week. We invite everyone, and we include everyone. So they will have a chance to talk with each other, from different backgrounds, with Australian people. So these things I think will also give them a sense of belonging. And also it's an awareness for the people, the other way, to meet people and learn about them. Not just what you hear…

Interviewer: Not the stereotypes. Great. And what are your observations of how your clients meet and interact with people from their own communities to maintain their sense of belonging?

Respondent: Yes, I think this is a good point. When you come here, you need the support. So if you see somebody from your country, from the same background, you really want to, you feel comfortable to approach them, to ask them questions, what happened when you came here, if you faced this one, what you are going to see. We have playgroups, and one of the playgroups is a multicultural one, and it's actually mainly for the Arabic. This playgroup is always full. Always we have, and they really enjoy it because they found a place where they can speak their language, and they kids play with the same age. So they enjoy themselves, because they found somebody to talk to them, and at the same time, their kids are playing, they are learning. So we try sometimes to have groups, just from the same new arrivals, and sometimes it's with the mainstream.

Interviewer: Great. And who are the key people that your clients contact for social and emotional support when they need it?

Respondent: Me. I think as a social worker, really if you don't have the heart to listen, you will not be here in this position. And people, when they find you like this, they become attached to you, and you gain their trust. So whatever the issue, they will come and approach, I think.

Interviewer: So the next set of questions around programs that are responsive to social support and improving the health of migrants. Now you've already talked a little bit about the social support, the NILS and the vouchers and all those sorts of things. But are there any other types of programs?

Respondent: We do health. We invite speakers from different services, like Refugee Health Service, or from the health service in general, talking about women's issues, children's issues, health issues. In the beginning, the Medicare health card, what's the difference, what you need. And also, we work sometimes in partnership with WILMA, or with Benevolent Society and organise wellbeing and health. So always we have something for them. So it's social group, but in the same time, we provide different information every time.

Interviewer: Great. And are you aware of any things that are barriers to people attending those sorts of events?

Respondent: Sometimes the time, because if, for the new arrivals, most of them they are attending English class, and our groups always clash with the same time with their… so sometimes the time. But we try to sometimes do it like the day where they are not in. So usually Friday they don't have a class. So we try to organise Friday, such things. If we have a big number from Novitas to attend. Or we have this group, the social group I told you, it's running every week. So during this session.

Interviewer: Great. So the next set of questions are around financial literacy and income generation. Would you be able to tell us about any programs available for financial literacy?

Respondent: We usually contact Saver Plus.

Interviewer: So that's through the Benevolent Society?

Respondent: Yes, the Benevolent Society. We refer to them, and one of the conditions to do Saver Plus, you need to attend four information sessions regarding budgeting. So if the clients, most of them from the same background, they don't have English, and for example they are Arabic, I will be attending the session with (NAME) to help with the language. I think sometimes, I'm not sure if she provides an interpreter, but usually if they are our clients, I will attend with them and will explain to them.

Interviewer: Great. And any programs available around income generation?

Respondent: What do you mean, income generation?

Interviewer: I suppose we've talked a bit about employment agencies and things like that. Let's leave that one. How about managing money effectively?

Respondent: That's one of the budgeting, Saver Plus. And the emergency relief actually. We help with the IPER voucher, with the electricity bill. But we talk to them about budgeting. This is one, second we advise them if they still didn't do the Centre Pay, which is they contacted Centrelink to take from the electricity company will detract from their Centrelink payment every payment, like $30 every fortnight. So this one, when the bill comes, it will not be very high. So they can pay it. So it's something like budgeting.

Interviewer: That's great. And what kind of financial challenges do your clients face while adjusting to life in Australia? Where do you start?

Respondent: The accommodation is very expensive, the rent is very expensive, and taking a lot from their income. Most of them, they want private school. Also it's taking, I told you, in the beginning it's culture shock. They still find it hard. So the school, private schools take a lot from the budget.

Interviewer: Wow. And what are some of the culturally specific dynamics that impact their ability to manage their financial? Sending money home, or the gender roles in managing money?

Respondent: What I found, it's a culture also. Because I used to work also as a complex caseworker, I have clients from (NAME OF LOCATION). Families there, from (NAME OF LOCATION) I didn't find this issue here, they don't have gambling problem here in (NAME OF LOCATION), but in (NAME OF LOCATION) and (NAME OF LOCATION), I found that they have gambling issues which affects the family. Because the husband will take the money, the children's money and the wife, the family payment from Centrelink, and do the gambling. And this will affect financially, the abuse will start. Like sometimes physically. So there is a gambling issue. But in (NAME OF LOCATION), I didn't find, because I think some cultures, the gambling is forbidden, they don't do it, and some it's allowed. And here, finding the machines everywhere, the gambling machines everywhere, it's tempting to play and play and lose and lose.

Interviewer: Exactly. OK, so how do they overcome these challenges? What sort of supports are there for the gambling?

Respondent: There is a service, there is a gambling service, but I think maybe the language barrier would be to access these things. Sometimes they deny, they don't have a problem.

Interviewer: OK. So what are some of the other supports provided by other organisations to support your clients with their financial challenges, besides the other gambling service and counselling service?

Respondent: Just I think the Centrelink, if there is a crisis or something, there is the special benefit. But financially, we don't refer to Centrelink, or here, we have the emergency relief. We help sometimes if there is a real crisis in their family. Not just with the bills, also we can sometimes pay for stuff for them. And the NILS program, no interest loans. So if they are eligible, most of them are eligible, to buy for them if they want. Like washing machine or fridge, T.V. all this stuff. We can help them, and this one will be deducted from their Centrelink payment with the small amounts every fortnight.

Interviewer: Great.

Respondent: It's up to $1,200, the NILS program.

Interviewer: Fantastic, that's great, thank you. So the next set of questions are around legal challenges. Now you've touched on some of these already. Can you tell us about the programs and supports available for your client with legal issues around identity visas, inviting family members to Australia?

Respondent: Usually, we used to have a migrant agent here, volunteer. She comes every fortnight, so always there is a long waiting list. And she helps with filling the application, but not that much, because she's a volunteer and she just comes two hours. Sometimes, if we have more than one client and they have almost the same issue with visa, applying for somebody to come, or their citizenship, when they apply, all these things, we will invite a speaker from legal aid to come and explain to them, and sometimes in the same session, we will do some referrals to them to book them, to help them. Otherwise we send them to legal aid, and the good things, we are the same building, so we can send them there or go with them sometimes to explain to the solicitor there, what's the issue.

Interviewer: And around physical violence or other forms of violence or discrimination?

Respondent: Again, we refer them to police, but really there is no response. When the New Zealand accident happened, and we had a few incidents happen here in (NAME OF LOCATION). Two of them, like in that time the liaison officer in the police, she approached me and she told me, there is an issue. And I told her, we have these two cases. And she said, please encourage them to go and report it. One of them, she was talking in (NAME OF LOCATION) here, one lady was abusing her verbally, very badly, and no one talked to the other lady, and she just ran away to the medical centre and hid there. This is one thing I knew. The other one, she was driving, somebody was chasing her in the car and abusing her also verbally. She was just 19 years old. She was very scared. Maybe for six kilometres, going after her. This young girl, the other one, she was over 50 and we told her to go and complain. She says no, I'm too scared to go to the police. The other one she's young, she has the English, it's good, but she was wearing a scarf. We told her to go and complain because that's what the liaison officer, she said it's good for the police to know what's happening. So she went there and she reported. A few weeks after, I think the liaison officer contacted me or I contacted her. I told her about this girl, and she went there and she reported. She said, I will check it. When she checked it, she said there is no report. They didn't write it down.

Interviewer: Oh my goodness.

Respondent: And you know, this girl, I talked to her a lot to convince her to go to the police. And she said, they will not do anything. Why I go? I told her, it's good, just for them to report it, there is an incident happened in (NAME OF LOCATION). And when they check, they didn't report anything. They don't have her name, don't have her number. They have nothing.

Interviewer: Wow. That's illegal.

Respondent: And this is, really we have so many incidents with them. One day we were in the park and we had like big festival. And there is so many stalls, and one of them, the police, they have a stall there. One of our clients, she's starting to park her car and somebody came, left his car and came to her and started punching her car, so she was really scared. And then he left her and he went somewhere, his house was near there. She was very scared. She came and she told me, and I took her to the police stall, and she told them. They say sorry, we can't do anything. We are here just for information, we can't do anything, we can't take an action. But we told her, we know where is he and where is his place. They say yeah, we can't do anything.

Interviewer: They didn't even advise that they'd ring the police, get somebody who can do something to come?

Respondent: I can't remember what she said, but we were surprised. There were a few of them there. It's not one alone, so to leave the stall or something. A few of them. But the way, the careless is what hurts. It doesn't matter.

Interviewer: Wow. So there's big problems here with the police in (NAME OF LOCATION).

Respondent: Actually I write a report, but then my manager, she said it's better not to send it to the police, we don't want to make a problem with them.

Interviewer: But then…

Respondent: This incident was a few years ago. But sometimes, things still in your mind, stay in your mind because it really hurts, and really feel discrimination.

Interviewer: And they haven't shown that they've changed their behaviour.

Respondent: No, I told you the New Zealand one, it was last year. I think there's no awareness about the culture, it's just a stereotype, what they hear in the news. Especially for…

Interviewer: They need some training.

Respondent: But, if they hear a parent hit their child, you will find 20 of them, not just one, over their head, over the parents' head. This is a problem really. Like I agree 100% you are not allowed to hit your child. But I understand why the parents hit their child, especially from the CALD community. Because they discipline, it's part of the discipline, and because they're worried about them, because they're scared about them, because they love them, they want to discipline them. It's not the other way. They don't want to hurt them. So unfortunately…

Interviewer: There really needs to be some sort of parenting program, because there is a parenting program, PPP and Circle of Security.

Respondent: Actually we do this one, we have the PPP and we have 1, 2, 3 Magic. So really, we are working on these things. But not by taking the parents to the

court, it's the solution. No, it's the other way. Here, they destroy the family actually, when they put this big problem. Taking them to the court is a big issue, it's a shame.

Interviewer: And fear.

Respondent: Fear, and also it will affect the relationship between the kids and the parents. It's destroying the family, really. Like you will change the dynamic of the family. The kids will not care about the parents, what they say, because the government is with me, the police is with me, whatever I do, I can do it here.

Interviewer: It's very tricky.

Respondent: Really, but I think they should speak to both, you have to listen to your father, this is your father, this is parents. But not to give them the no, he's wrong and you are right, and that's it. Unfortunately.

Interviewer: What would you say some of the key laws or provision are, that migrants need to learn when they first arrive in Australia?

Respondent: I think the language. The language, it's very important to learn. Even it's hard, but they need to try their best to learn. The language is the key for everything, I think.

Interviewer: In your opinion, what's the level of awareness of migrants to accessing key legal services when they need them?

Respondent: Usually they will contact us. I have always there is an issue. I have one client, she called me two weeks ago. She said, my husband contacted job agency and they told him they are going to deduct from his account $30 or $50 every month. And she said the first time they took $30, then $200. And they told him at the beginning, there is no contract. And she say, we are calling them to stop it, to cancel it, but still they are doing it. So I told her, you need to go to legal aid. And so we refer to legal aid.

Interviewer: OK, thanks. So the next few questions are around movement of your clients from one place to another. So what do you think the key reasons for people moving from one place to another, or one suburb to another?

Respondent: Accommodation. Like people moving to (NAME OF LOCATION) because it's cheaper than (NAME OF LOCATION). It's much cheaper here. Sometimes the job, I have a client who moved to here from Newcastle actually. They are from Syria. She moved here because her husband, he's a truck driver, and the company is in Ingleburn, so she moved to (NAME OF LOCATION). But mostly the rent, accommodation. This is the reason.

Interviewer: And are you seeing any trends in mobility in the early years of migration, or after several years? After being here for several years?

Respondent: Regarding what, accommodation?

Interviewer: No, just their mobility, like any trends. Are they more likely to move in their early years or later?

Respondent: Later, actually, later. Because some of them, we have a few people, it's what they call the second settlement for them. A few people moved from (NAME OF LOCATION) and buying accommodation, a house here. After they work, and have some money or things, they're starting to come here. We have a few Iraqis [inaudible, 53:58] who used to live in (NAME OF LOCATION) for a long time, but they start to move to (NAME OF LOCATION) area.

Interviewer: Great. So the next questions are around education and literacy programs. Now, you've talked a lot about early education for children, and some issues around schools and things like that. So if you have nothing else to add, that's fine, but I'll ask them anyway, just in case. So please tell us about the services available to migrants using your service in terms of school education for their children, adult literacy programs or any other educational literacy programs.

Respondent: Like we said before, we have the PP programs, and really this one is a really good one, and help the parents and the child, to help the child to go to school. Prepare the child, while giving them some books, all the stationery actually. All this service is free. Then follow up with them how to teach him. Even it's just they help, this program helps them to learn the concept of teaching, even if they use their own language. So what's the difference between big and small, tall and short, and these things. And also like how to hold the pen, all this stuff. And we have the Start Right, this program also, it's from 0 to 3 years, to check their ability for learning, if there is any issue, to fix it from early. And we have the playgroup, and we have the tutoring program, as education. And we have the English class running, not just Novitas, also we have English class, after they finish ours and they still want conversation class, we have here, for adults.

Interviewer: Fantastic. And you've already touched on a few of these as well…

Respondent: Sorry, one thing I remember, and we do outreach TAFE courses, so we partner with TAFE and do courses like, now there is one, the computer class, and next year the sewing class is running. Sorry.

Interviewer: That's good. What do you think are the key issues or barriers to children of your clients to accessing school or university education? You've already mentioned a few of these.

Respondent: I think, what I've found, the kids in primary school, they adjust very quickly. But for the youth, it is very hard, because already they are in very hard age, like teenagers, it's very hard to get friends, the language, to learn the language. So I have an issue with applying to the university. Not just applying to the university, if the student was in Year 10, 11, 12, it's very hard to adjust and learn the language, and to study. It's like a shock for them. They were very bright in their country, but they were learning in their language. But when they come here, they find it very hard to adjust and to learn the language. And I

think not all schools give good attention to those people. I have clients, the family, they have two teenagers when they came here. One of them, it was very hard for him to finish Year 12, because he spent all the time putting his headphones on in the class and sitting. He doesn’t want to talk, because he was worried, if he talked somebody will laugh on him because of the accent, because he doesn’t know the words. So he isolates himself. And he doesn’t know that much about the system, about the options of getting courses. Maybe you can’t continue your study. Because in their country, the education system is very different. If you don’t do well in the school, that means you are not eligible for any further study. So that’s their thinking. And I have another student, almost the same issue. When she came, she was very bright, but then she gave up, she found it very hard. Even she, the problem also with the school, they don’t give that much support. They told her maybe it’s better for you not to do H.S.C. because if you do it, your marks, it will be very low, and it will affect the whole school. So I don’t think you will be OK if you hurt other people.

Interviewer: Oh my goodness.

Respondent: So I found it’s very hard for the youth, when they come in this age. Very hard to adjust.

Interviewer: Are you able to tell us about any special packages or subsidies provided to support educational opportunities?

Respondent: That we have here? No, just for the kids, for the young kids, the PP Program. For the young we don’t have any packages. Sometimes we provide the tutoring after school.

Interviewer: And are there any supports, I think you’ve already touched on this one, for migrant opportunities more particularly, any support for them to gain employment?

Respondent: Just regarded education actually, I remember the Western Sydney University, this student, I told her, they told her you will not, it’s better not to do your H.S.C. Then they contacted her after a few weeks before the H.S.C., they said no you can do it, but it will be mystery marks, it will not be counted with the other students, so your mark will not affect the other marks. Actually I said for her, because she wanted to go to university, she does have a dream, her parents dreams, to go to university. And she doesn’t want to go to TAFE. I found out in Western Sydney, the provide a diploma, for I think one and a half years. And then this will allow her to apply for university. And she will be not in the first year, she will be in the second year. So they will count this. So I like Western Sydney University really, because it gives opportunities for those student in this situation. So she applied actually, and she found the subject that she wanted to study. She found it into university, Western Sydney University and Macquarie University, but because she lives her, so she prefers Western Sydney. So she applied, and I hope she will get it.

Interviewer: Excellent, that’s great.

Respondent: For employment, you asked me about employment, if we have any programs. For settlement, it's not our job, a settlement worker, to find them a job. But with the disability actually we have a program. It's for general, but if somebody with a disability is looking for a job, and a new migrant, he's eligible to our program, and she will sit with him and look with him, what he needs, and she will match him with something. This worker, really she's doing very well with this job, I think for employment or something related to employment.

Interviewer: Great, good, we're nearly there. So overall, what do you think the key challenges are for migrants that you work with, while adjusting to Australian culture and settling in.

Respondent: Maybe it's the key for the mainstream is the awareness of refugees and the issues they've been through. Because really, not everyone knows about refugees and the number of refugees here in Australia. They think there are millions of refugees in Australia taking their jobs, and really there is a lack of awareness. They don't know, Australia is the 88th rank of the countries taking refugees. We are taking a very, very, very small number of refugees. And awareness for the police, are the issues really. The issue they have. It's not actually just the police, the child protection service also. They need to provide parenting guidance for them instead of taking the kids and breaking the families. These things. This is my passion, like to awareness about these things, these two things. For people to know about refugees and what they are suffering, and they are here to do good things, not to take your place. They want to live, but they want also to be part of their community and give back to Australia, really. They just need the chance. As I told you, they need work, but they don't know how to apply.

Interviewer: And I think there's a poor understanding around the enormity of adjustment. All the tiny little things, even just not knowing who your neighbours are, or being, prior to migrating they would have known everybody. It's very different.

Respondent: Very different. You know in the beginning, also we found it very hard to find accommodation. No one wanted to rent for them to give them, because they don't have a history of renting, and they are refugees. But really, I have one client, he's a painter, so always, every few months, he will paint the house. And the owner of the house, he was impressed. He moved to another, he said please, I will reduce the rent, but just to stay, because he's looking after the house very well. Really. So these stories actually, you don't hear it. You hear always, the house is become small to us so we want a bigger house because the kids are growing up. Please stay, I will help. And the last time also, he painted the house before he left. So every time they come to check the house, do an inspection, when they do the inspection, she is always impressed.

Interviewer: Wow, that's a good story. And finally, what would you like to see as possible solutions to helping or supporting migrants to adjust well to life in Australia?

Respondent: It's the same things. I want people to understand really, to welcome people. To welcome them here. Mostly they are welcoming really, to say, but still there is some people…

Interviewer: It only takes one bad experience, and you remember that one. It's normal for all humans, for all of us.

Respondent: I think also to find a job, to make it easy, not complicated, the process of getting a job like this one, to make them more settled. To help them actually find a job in Australia makes them very proud. They are working in Australia, it's really something important and good. So I have a few clients, actually they are now working with the Department of Education, to sit with child with a disability in the bus or in the car to take them to the school. This job is very small, but I find out it really makes them very proud, because they are working with the Department of Education in Australia. So even a small job, it makes them happy. But actually, applying for this job is not easy, because they need to take some courses, like the first aid, and fill in all these forms. But we try to help them to get it. But still, they are very happy, very proud to have it.

Interviewer: That's good. Alright, well that's the end of the interview, how's that? Is there anything else you'd like to add?

Respondent: Not really, I take it out, all.

Interviewer: That's good, thank you so much for your time and wonderful insights. There's a lot of points there that no one else has made. That will all make the data much richer. So thank you very much.

Respondent: Thanks for you, thanks.

Interviewer: So we're finishing the interview at… where's my phone gone? What time is it?

Respondent: It's 12:00.

Interviewer: It's 12 o'clock. OK, thank you.
