# Supplementary material for: The importance of information acquisition to settlement services literacy for humanitarian migrants in Australia
Source: PLoS One. 2023 Jan 6;18(1):e0280041. doi: 10.1371/journal.pone.0280041 (PMC9821785; doi:10.1371/journal.pone.0280041)
Supplement: S1 Data — (ZIP) [file pone.0280041.s003.zip › SP_09_Victoria.pdf]

Interviewer: (DATE), 2019, 9:45 a.m., (SERVICE NAME) with (NAME) and (NAME). So, (NAME), thanks again for taking the time, just to clarify, all these questions refer specifically, because obviously you do other services here, you do a whole bunch of community and social services, this is specifically about newly arrived migrants and the services you offer them, starts with the first five years and both voluntary and involuntary. Obviously in voluntary, refugee is voluntary, economic migrants, which, actually, just an important distinction before I start the key questions, is, do you, what migrants do you work with? Do you work with only voluntary or do you also work with family reunions and economic migrants?

Respondent: We work with everyone who has been in Australia for five years or less and has permanent residency or citizenship. So that can range from refugees and non refugee communities as well but generally, most of our clients are from refugee background not from migrant background, but yeah, we kind of think a bit more broadly about our eligibility criteria to support people depending on what their needs are especially when we are seeing people who are on temporary visas, we try and include them in our group work and provide some case work to them where we can, but yeah our capacity to do that has changed since we lost a bit of our funding in the last SETS round, so SETS funding, we lost some money, yeah, we lost some money in December, so it means we're being quite, kind of sticking to, what our funding body requires in terms of our, yeah, eligible participants.

Interviewer: Which is people from humanitarian…

Respondent: Humanitarian, yep, yeah.

Interviewer: So you do get still, you do do some work, you get cont- you get approached and contacted by other migrants, not humanitarian…?

Respondent: Yeah, and we particularly receive referrals through the main torture and trauma counselling service Foundation House, for people who are not eligible for our, for our supports but yeah we're SETS funded, so humanitarian and newly arrived and I'm just trying to think of our economic migrants and how that looks in terms of our work, so on the newly arrived they're able to access our services.

Interviewer: Yeah, OK, but it sounds as though that you don't come across that as much, so they're not contacting you, coming to you, so much. Alright, and, alright so, the first question is basically about what services you offer newly arrived migrants, I guess generally, so obviously the SETS.

Respondent: So as a program we mentioned SETS, we also receive funding from different bodies and I can't remember them exactly right now and one of them is local council and we also have, so we've got our community development projects

which are either funded by an alternative funding body or partly funded by SETS, or all SETS, and then we have homework club which is a whole other, it comes under settlement, certainly because we're working with young people and the eligibility criteria is also similar to SETS, however, that doesn't look like that in reality, there's a lot of young people who need homework club in that area, it's a basic learning tool and yeah, so, yeah essentially it's a broader group but with the focus on having SETS eligible participants so that we're funded to do the work. Just thinking about programs, so we run a number of programs, I think it's best just to list them for you but, we do kind of information education based programs, social programs, and you know topical programs depending on what the interest or need is for the community, so, over the past few years some of our community development projects include parenting, volunteering, brad citizenship course or training to prepare people for the test, like I mentioned homework club, cooking and conversational English class, gardening and conversational English classes, Arabic speaking, social, intercultural Arabic speaking social groups, ethno specific groups, we've run groups particularly with the Chin, Hakha Chin community, looking at how we can support them to be incorporated, and that's now being taken over by another organisation. They're the programs that are coming to mind at the moment, there's women's groups, there's parenting as well, did I mention parenting?

Interviewer: Yeah.

Respondent: There's parenting groups, yeah, that's what's coming to mind at the moment.

Interviewer: That's alright.

Respondent: And then we have our case work, so we provide case work support, we're also housing support workers, as we have, we provide support to our families and individuals who are in transitional housing which are managed by Salvation Army so, for that reason we can be involved quite long term with people particularly around housing.

Interviewer: Excellent and so you mentioned Salvation Army, who, what other kind of organisations do you collaborate with in doing your work?

Respondent: The main city council, Salvation Army, we have a worker who works across, she's been working across three sites more or less and she's partnered with a number of organisations around information sessions and that's been something that's been ongoing and established really well in terms of individual organisations coming in and out of our community development space and work, I'm just thinking about other partnerships.

Interviewer: Do you deal with service providers, other settlement service providers?

Respondent: We tried that on a number of occasions in a number of different ways. It's, from what my co-ordinator has reported back, it's been really great since the different structure, you know, the newer structure with settlement there as a requirement for all co-ordinators of the pro- of SETS programs to come together and collaborate and inform each other of what they're doing, and so…

Interviewer: Oh, so that's a part of the SETS funding program to collaborate.

Respondent: Part of the SETS funding, yeah, so there's a person, I could get you those details, there's, he's from Brotherhood of St Laurence and he's been fund-, he, they, Brotherhood of St Laurence have received funding to co-ordinate this meeting that SETS, yeah, SET providers, where SET providers managers meet and discuss things and collaborate.

Interviewer: Yeah, right, I didn't know that at all.

Respondent: So in actual…

Interviewer: There's a lot I don't know!

Respondent: Yeah, right!

Interviewer: But.

Respondent: In actual like, in terms of tangibility, I don't know what it looks like. Like, if there's actually stuff that they're doing, so I could definitely suss that out for you and find out more information. Collaboration. Yeah, no, I think that's been really hard like even receiving referrals from other SETS providers, it's pretty rare.

Interviewer: Yeah, right.

Respondent: We refer out to settlement and that's, yeah, not blowing our own horn in terms of how we engage with other SETS providers, but we often refer to other SETS providers, but we do not receive referrals from SETS providers.

Interviewer: Why do you think that is?

Respondent: I think we're a small organisation and I think numbers are important in terms of that competition.

Interviewer: Yeah.

Respondent: I think everyone is so focussed on their own programs.

Interviewer: Yep.

Respondent: That there's little time and resource to be thinking about how they can refer to other programs.

Interviewer: Yeah, OK.

Respondent: There's not a lot of incentive or, I don't know, I think it's how we work is different in terms of every organisation and the culture and I feel like we do more than we need to be doing with our clients in terms of, for example, the way SETS is structured, in my interpretation, it's more of a, as needed service, whereas we often end up case managing because the penny doesn't drop elsewhere so it's not a parenting issue so it's not Child First, it's not mainstream because they can't access what they need through mainstream services due to language interpreting, there's a number of things, so there's this expectation that we are kind of like, because we're culturally competent, because we work in settlement, that we can do everything and anything and in some essence we are really flexible and able to be creative with our program so that is fantastic and I wouldn't want that to change, but on the other hand I'd like to see that in the next ten years we won't need settlement providers because we will have competent, culturally competent and able people across all organisations, mainstream and specialist, to be able to support people who are newly arrived because essentially it's a large, you know, proportion of our population and people who need things, what people need in the first year, in the third year and in the tenth year, there's an overlap and there's not that you know, kind of clear path of where people are at in those years so we also are really under resourced in terms of supporting people past five years where things can really fall apart and, yeah, anyway I'm sure there will be a question about housing and where, once people are in these services what happens, but yeah, people do shop around for settlement services and we can talk about that later as well, but yeah I do not have confidence or gratitude for the amount of people who have been referred to us all, like cross program referrals through agencies.

Interviewer: Alright, and so do you see any services that are over or under utilised at all, anything that's big demand for and you can't offer it or is there anything that, there is, you know, funding for it exists, but no-one's using it?

Respondent: Under utilised?

Interviewer: Mm.

Respondent: Nothing's coming to mind.

Interviewer: Over utilised at all?

Respondent: Over utilised, as in, the, you, kind of, when you say over utilised are you referring to, there's definitely a need for more of whatever your s- whatever that service is providing?

Interviewer: Yeah, yeah, yeah.

Respondent: I see the Foundation House go beyond what they're doing to try and connect people to services and referrals, I think they do that really well, but on the other hand they're counsellor advocates so I don't know how much time and resources they actually have to do that, so I feel like they go a bit outside of their role sometimes. But also there's that huge level of trust; I think, what there needs to be more of, is the ability to be therapeutic and practical, so we offer a lot of therapeutic support throughout the work we do and we, like brief counselling and we ask a lot of questions and we, you know, we go in to detail about things around people's mental health because we want to understand what their needs are, how we can support them and what other services we can put in place, so settlement is over utilised in terms of mental health, I think, and housing certainly like, a large proportion of what we do is housing.

Interviewer: Yeah, right. Excellent. So, can you tell us a bit about your understanding of how migrants that you work with, understand Australian culture in society?

Respondent: There's a very strong, like, I don't know what to call it, gratitude consciousness, like this need to be grateful that they're here, so that's, this over arching kind of lens I think, that people see their support through. People who know people already in the community whether it be family or friends always are doing better, nearly always are doing better.

Interviewer: In terms of navigating the kind of…

Respondent: Well there's less…

Interviewer: … cultural structures and you know the services…

Respondent: There's that but there's also less fear because there's someone to trust and there's someone to fall back on, so in terms of housing for example, people often end up couch surfing with their children, staying with families that they actually don't know very well and can be often taken advantage of financially because they've got, so there's that sometimes within the community, kind of that, I don't know what to call that, because it's abusive and it's financial violence in a sense but it's not family, it's someone in the community trying to make some money and yeah, so, that can happen. Overall though that's a small, that's a very small percentage of people that I've come across. Housing becomes an issue very early and that's because our families and individuals are often put into proper- are placed in private rental through the H.S.S. and they can't afford to stay in them, they can't afford their bills, they can't afford basic living and their rent, so often within a year they can, that falls apart and we are then seeing people who have exited H.S.S.

Interviewer: Yep, yeah. So that I will…

Respondent: I have kind of side tracked!

Interviewer: Yeah, yeah, no that's OK.

Respondent: Sorry. Because there's all this stuff that starts to come up from a general question.

Interviewer: Yeah, so I guess it's just about, your…

Respondent: Their perspective.

Interviewer: … how do you see new migrants understanding Australian culture in society, you know the rules, the norms, you know, that kind of, if there is standards and rules and norms, which I think there are to an extent.

Respondent: For families, most, most majority of our families they've come from political conflict…

Interviewer: Yeah.

Respondent: … and there are a lot of questions around that, so in terms of their understanding of Australian culture, I think it's, they think it's like fairly peaceful and equitable and straight forward until they hit certain road blocks, like, I've been trying to find a cleaning job for three and half years and I can't find a cleaning job. So then there's this kind of shift in, well, how fair a go do I have in this country when I'm keen and ready and able and I'm not being able to move forward, so employment's a big factor I think and how people see culture in society in Australia, and they hear from their friends or their friends have friends that they, you know, have found jobs so their expectations of Australian culture versus the reality of Australian culture, for many individuals is quite different. I think the culture of which they come from will certainly influence how they interpret Australian culture in society. Because we see common attitudes and behaviours depending on where people are from. So without singling out, but, like I've found the Tibetan community often just seem to, I don't know if there's a word for it, settle better, and their approach to Australian society is one that is of, I don't know, appreciation and there's a calmness about this community in general that they come with and there's a lot of torture and a lot of oppression and pain and suffering and trauma that this community come from, but, and I'm not saying that they're better than any other, but I'm seeing with time they find work and they're linked in well and honestly, when I think about how do people who are newly arrived, think about Australian society, it depends on the level of compassion and respect that they receive as well and that judgement or discrimination can be often based on how we look or what's in the media at that time.

Interviewer: Yep. Yeah. So we can imagine to that extent, obviously people coming in permanent and backgrounds with the media and government narrative that's been pushed in the last year is going to impact their…

Respondent: So it's harder for the Muslim young people to find work, particularly women because they identify different you know, in their appearances, somewhat obvious in terms of what religion they practice and colour. Yeah, then you see like Sudanese young people that are just so overly well behaved and well mannered and there's obviously a pressure to be a certain person too so, to make up for all the others who are in the media and being targeted as you know, being the bad lot who should be returned home, so. Also in terms of culture in society, we had a, there was a period, I think it was last year, we thought about, how we could actually support our African communities who are, who maybe facing discrimination or racism, because of the level of crime that's been portrayed by young African boys in the media and so we had agreed to individually meet with our clients and have a chat about that and just to open that conversation about society, safety and how your sense of belonging may or may not be impacted by these things and that we are aware of the issues and largely our clients were actually feeling safe and comfortable so and that it was not something they thought about too much politically. Yeah. And I think there's an expectation, going back about cul- what people might interpret about this country, that they will be able to reunite with family and so when there's that realisation that it's not going to happen or likely to happen, their sense of belonging here deteriorates quite quickly because it's like, oh we should be grateful we're safe but we don't have our family and I haven't seen my children in x amount of years, so, especially for the older ones, older, like more, you know, the older generation, who have adult children and don't see themselves reuniting, it's like, yeah, I'm grateful to be here but also really unhappy here and I wish I had settled elsewhere. There's a lot, you're asking a big question!

Interviewer: Yeah, yeah. So do you see that your clients have opportunity to practice their own culture?

Respondent: Yeah, yep, yep. I don't have a lot to say about that but certainly yes, and depending on where they live, more so. So, I think, ethno specific association type and you know, community leaders can do that quite well and religious institutions bring people together but then again the people that might need it the most are people who might not even want to engage with their own cultural community so it's really great that we have SETS programs where we can bring newly arrived people together who are not of the same background.

Interviewer: Yep, right. So the next questions relate to migrants sense of belonging and inclusion in Australian society and now thinking about your programs here at (SERVICE NAME) rather than broadly, can you tell us about the programs or supports available here that help to create to help and enhance migrants sense of belonging in Australia?

Respondent: In my experience, because, in my experience, I, I imagine my colleagues might have similar things to say, my programs have been mostly socially based, or conversationally English based, conversational English base, which means people are coming together not for a particular skill- to enhance a particular skill set other than engagement in English, so it's social and so the groups I run is a social group, a photography group focussing on diversity and harmony within the community, that is skill set, I mean it's learning how to do digital photography, but essentially that program was about exploring their local community and their identity in like, cultural connection, well connection to their local community, yes, so cooking and gardening and their, the regular participants, their feedback was, that they felt a sense of belonging, they are really grateful to have somewhere to go out and enjoy their life with somebody else or meet someone new, I've lost the sense of the question, can you repeat the question?

Interviewer: The programs that you offer here that increase migrants' sense of belonging in Australia.

Respondent: Yeah, yep. In terms of increasing their belonging, I think it really helps to have spaces where things are not heavy, people can simply socialise, meet, hang out, maybe eat a meal together create something we've done, artwork shops as well and the general feedback is, we need more of this and there are people in our groups where their only social activity or only social program is the program that we're delivering.

Interviewer: Wow, yeah right.

Respondent: So, we do a lot of cross cultural programs. So, it's really easy, it's ten times easier to run a program just for the Karen, or just for Somalia or African or just for Tibetans but having, and that's great, because there's definitely a need for that, but yeah, often it's that cross relationship or intercultural relationships that makes kind of a different experience for people and so we like to, we've spent a lot of time diversifying our programs because historically we worked a lot with particular communities depending on the need of that time and having bi-lingual workers will obviously attract a particular client group, cultural group; what else can I say about that? And yeah, the relationship that participants who attend these groups have in terms of their identity and sense of connection they have a lot, they, there's a great sense of value and respect towards the person who's facilitating, it's almost like they put them on a pedestal at times, there's just so much respect for that relationship and I think it's mainly because that's their connection to Australian society outside of, we're all here, we're all new, you know and yeah, having easier access to people of their own community, yeah, so like one program that was running in this area, it's called Welcome Dinner and it's a really, called Welcome, Welcome Dinner or Welcome… I'll have to double check it, it's not the

Welcome Dinner, anyway, but the idea of that is, we've got local people interacting with newly arrived, so we invite people to dinner and someone agrees to host and then yeah it's an opportunity for newly arrived migrants and refugees to meet people who yeah, have, are not new, are not newly arrived or Australian or Australian born or have lived here for a really long time, and that's a g- very successful program. Yeah.

Interviewer: Alright.

Respondent: And, sorry, I might say this, sometimes it's quite simple things in terms of feeling that familiarly of sharing food or them sharing their food with us, it's the simple things that go a long way sometimes, yeah.

Interviewer: Totally. So can you tell us about the types of programs that are currently being implemented to support health and wellbeing of new migrants?

Respondent: From our programs?

Interviewer: Yeah, from (SERVICE NAME)

Respondent: Health and wellbeing? Kim, one of our settlement workers provides series of information sessions focussing on different topics, it could be around access to disability services, it could be around getting a breast check or a, yeah, a particular health issue so that's yeah, a way of sharing that information with newly arrived communities but mainly Vietnamese, she works mainly with the Vietnamese communities, yeah.

Interviewer: And so do you see anything working or not working in providing these health information sessions?

Respondent: I don't know actually.

Interviewer: Yep. That's fine.

Respondent: I don't know, but from a case work point of view, having someone be able to support participants to co-ordinate their services when there's a multitude of issues including health and mental health issues, the, I think the thing that helps them the most in terms of things not kind of falling apart and the important things staying on the agenda is often a case person, a person who's case co-ordinating.

Interviewer: Yeah, OK.

Respondent: Yeah, so if you're in and out, yeah, it depends on the needs of the client but often when we're talking about health and wellbeing, the difference between an average I guess case worker support and an exceptional one is someone that's helping co-ordinate the services and help with the communication between services.

Interviewer: And so, next questions are about financial literacy, income generation, managing money, so can you tell us about any programs that you offer that relate to any of those?

Respondent: Well we offer, we've been trained to provide assistance in terms of budgeting and looking at alternative ways to save money on electricity, gas, water, so we pass on that training to our clients and also they know what services are available if they cannot afford their bills, and also we can refer to financial counselling through another organisation as well.

Interviewer: Excellent. And what, what are some of the key financial challenges you see your clients facing in settling in Australia?

Respondent: Housing affordability, understanding Centrelink, needing advocacy around Centrelink, being unclear about their, the support they can and can't receive, and just in terms of job networks and that certainly relates to financial because without participating in those things they could lose their Centrelink. Understanding concessions like, where can you get concessions, financial resilience, everything!

Interviewer: Yeah. And is there any culturally specific factors contributing to financial literacy or managing money, anything to do with being a new migrant that might…?

Respondent: For some families they can eat quite poorly, and sometimes that means that they're over spending on convenience food and food that they think will make their kids happier, it's sweet or you know, attractive and targeting kids so less healthy food which has all sorts of other effects on the children and you know, they're behaviour in terms of what they're eating and affording everything else when you're spending money, over spending money on food.

Interviewer: Yeah.

Respondent: Food accessibility; we have a number of programs we can refer to for food aid.

Interviewer: Yep.

Respondent: Biggest issues is affording rent…

Interviewer: Yeah, of course.

Respondent: … and affording utilities.

Interviewer: Yep, so what about things such as sending money home, is that an issue at all?

Respondent: Yes, yes.

Interviewer: I mean, this is their home but sending money to family back where they migrated from.

Respondent: Yeah, there's, it can be quite personal thing to talk about because of the fear of you know, will people help me with other things if I'm trying to send family money, and it's nearly always for a really good reason why they're sending money back home, because someone's sick, someone needs an operation, someone hasn't seen someone else and they're trying to reunite them or they're trying to get them a visa to visit them here because they need emotional or, you know, practical support with their kids, so there's a range of things why they would be sending money back.

Interviewer: And how about, something else that I've got a feel, out of this, what about in terms of gender balance, I know often you know, say women can receive money from Centrelink for children rather than the men, is there any kind of gender ailments to managing money that would show up in Australia as people come here?

Respondent: I've definitely seen financial abuse, financial abuse based on, yeah, just seeing that men who may have better English or better understanding of the system, you know, serve the system, navigating systems, yeah, can often dominate the decisions and leave their partners in the dark. There are extreme examples of that as well. I've worked in settlement with pr- I've worked in settlement and I've worked with asylum seeker groups as well in my time so, over the last say 12 years and yeah, often if there is some sort of gender, if there's gender issues or family violence relating to finances, I think it's pre existing and I think it can get harder for women once they start to realise what their rights and you know, what their rights are in Australia, then relationships can become more tense and sometimes break down and I think finances certainly stops women from leaving regardless of being newly arrived or not but being newly arrived means that there's further vulnerability because of language.

Interviewer: Yeah, maybe less connections.

Respondent: Yeah.

Interviewer: So, next question is about how any programs that you have to support clients when they face legal challenges.

Respondent: We refer on mostly and help co-ordinate that relationship.

Interviewer: So to community legal?

Respondent: Yeah, community legal and specialist legal services like, yeah, with women or family violence.

Interviewer: And do you do, you don't do anything for migration at all in terms of migration services?

Respondent: Not since 1st January this year, previously we had a migration agent provide support, however we do help people like the citizenship, like help them with the forms and also provide citizenship training to help them prepare themselves for tests.

Interviewer: Yep. And so I guess, in your opinion, what's the level of awareness of migrants in accessing legal services or understanding law here in Australia?

Respondent: Not a great deal of understanding. Not a great deal at all and I think little interest but not because it's not important it's just, I think, on one hand they've had such a political, politically heavy experience or pol- I don't know, where politics and what happens in their country is just so abrupt and so horrific in terms of what that looks like, so, I think people are just happy to be here and safe now and so, what's capacity or readiness to connect with the structural picture, they certainly do that but it's, it's not a lot, like, they won't go into a lot of depth and ask a lot of questions about political things, so I don't know if that's how, it's about how we deliver services? but I think there's so many things that they're trying to do for the first time in a new country that, that's with, you know, completely understand why it's not a priority.

Interviewer: Yeah. And do you see, are there any trends or any key legal challenges that…?

Respondent: I think that people always trying, more than not, trying to do the right thing, like where do I park, I actually don't understand where I'm supposed to park because of this street, there's no disability parking on this street, like, parking, that's come up heaps, riding a bike in public and not knowing that a young person, not know that he needs to get off his bike when you ride on a train station and got a ticket, then in another situation he wanted to go and speak to whatever, the, station staff, because he realised he didn't have his myki card and so he jumped the gate to go and speak to them and then they fine him on the spot for jumping the thing when he was trying to do the right thing so then he had to do community service to pay off those fines and he'd been for a very short time. Yeah. Those sorts of things can by mis- by not understanding, what, like, no-one really tells them that, no-one, it's, there's a lot to cover, where do you park, how do you ride your bike, where do you buy a helmet from, when should you wear a helmet, you know, it can be really smallish things and then their relationship becomes...

Interviewer: You can ride on the road, but you can't ride on the footpath…

Respondent: … yeah…

Interviewer: … for instance.

Respondent: Yeah, so their relationship with the law is very confused and sometimes they're targeted because of how they look, by police, so relationships yeah, their relationship with and wanting to know more about the law, it's about, OK, what do I need to know to do the right thing, it's kind of, most important part.

Interviewer: Yeah, alright. So these questions relate to movement of your clients from one place to another say in Melbourne, so what are some of the key reasons why…

Respondent: Yep.

Interviewer: …so this is seven, eight, nine and ten, we'll go really quickly, so do you see any key reasons for the movement of clients from one place to another?

Respondent: Do I see any reasons why people move?

Interviewer: Or what are the key reasons yeah?

Respondent: So, families often really struggle by choosing to stay in one spot because they want to maintain their connection with family or school or the community. So there's a real reluctance to move outside of where they, what's familiar. But the reason for moving is often to be closer to family or housing affordability.

Interviewer: Excellent, alright. So next question is about access to education literacy programs. Do you run any services, you said conversational English, do you have any other?

Respondent: We have because we're also, our (SERVICE NAME) is also a registered training organisation which is called (SERVICE NAME) and we've collaborated with them to deliver English language classes alongside sort of social, practical activities, so like I've mentioned, so there's actually a trainer who was providing English training, oh and then there's called, it's called Language For Life, so there's several programs that we deliver, English, numeracy, literacy, yeah.

Interviewer: Yeah, so quite a bit. Are there any, do you see any issues or barriers with children of your clients in accessing school or university?

Respondent: Not with primary school aged, no.

Interviewer: No.

Respondent: But then yeah, in terms of higher education, yes, not knowing where to go and what's most appropriate for their skills and is most likely to get them a job, that's what we do a lot of, so, it's not so much that they're limited or unable to access higher education, it's just knowing where to go.

Interviewer: Yeah, for sure, and so linked to that do you see employment pathways for children of new migrants?

Respondent: Very little.

Interviewer: Yeah, OK.

Respondent: There's specific programs that come up around you know, like the campaign to get people into the Australian work, Australian Police Force or something like that, it mainly depends on their English language in terms of the chances of them getting work, but thankfully, in our case, because we have a program we can refer to which is also part of the, part of (SERVICE NAME), we can refer on, we can help them to find work. I can't say that they all find work but by having settlement providers be able have a good relationship with other employment services, that certainly increases the chances.

Interviewer: For sure.

Respondent: But I don't hear a lot of people getting work through job networks, I think they're mostly…

Interviewer: As in the government job network?

Respondent: Yeah, I think they're mostly useless.

Interviewer: Yeah, that's beyond, I was once involved in their actual person finding job years ago. And so, what about employment opportunities for migrants more generally speaking. Are there employment opportunities out there?

Respondent: No, not really.

Interviewer: Yeah.

Respondent: No. There's a lot of talk about it, but I don't find clear pathways for my participants to find work.

Interviewer: OK. And so overall what do you think are the key challenges migrants you work with face, while adjusting to Australian culture and settling in Australia? So you've probably mentioned them all already, but what do you see as the key ones?

Respondent: Housing.

Interviewer: Housing.

Respondent: Employment.

Interviewer: Housing and employment.

Respondent: Yeah, yep.

Interviewer: Alright. And finally, what would you like to see as possible solutions to helping or supporting migrants to adjust well to life in Australia?

Respondent: I think a lot of, like most of my participants that I work with are really keen and ready to work. I'd like to see more jobs available for particularly women who need school hours employment and more entry level positions available to people who may not speak adequate English, because we know that there are many jobs that people can do and not require a high level of English, so I don't know if that means the government providing some sort of incentive to employ people who are ready to work and who are able to work and just seem less attractive because of their level of English. In saying that, yeah, there's a lot of cash in hand work that happens as well. What else would I want to see? I think settlement services would definitely benefit from having partnerships or within the settlement program, therapeutic, like an aspect of it which can be therapeutic or acknowledges therapeutic and funded as therapeutic because a lot of the practical things we do with people often has practical, has therapeutic benefit, and often, more than, yeah, most often participants are not familiar with that counselling relationship, like you sit in a chair, I sit in a chair, I talk about my problems, you help me understand my feelings, it's the things that happen on the side of the road, or in the car or while we're waiting for a doctor, there's a lot of yeah, therapeutic benefit that can come from practical settlement support, so, yeah. More resources.

Interviewer: Yep.

Respondent: I don't know, there's a whole lot of stuff, I'm just trying to narrow it down. We were in the past more able to deliver cultural competency training to mainstream services, helping them understand, how to use an interpreter, who can provide free interpreting, like how you can access free interpreting, what are the needs of this client group, so you kind of continued to get that word out and build the capacity of your community more broadly, yeah, so, but yeah, there's a lot of competing priorities and that's not one of ours at the moment, but we do certainly see that as an important part of what we can be doing.

Interviewer: And so it goes back to something you mentioned at the very start about having culturally competent people working in mainstream services across the country.

Respondent: Yeah.

Interviewer: Yep.

Respondent: Yeah.

Interviewer: Alright, so that's the end of the interview. Is there anything final you'd like to add at all?

Respondent: No. No.

Interviewer: Right, excellent, thank you (NAME). So, thanks for your participation and contributing your expertise and knowledge and the interview ended at 10:30 a.m.
