# Supplementary material for: The importance of information acquisition to settlement services literacy for humanitarian migrants in Australia
Source: PLoS One. 2023 Jan 6;18(1):e0280041. doi: 10.1371/journal.pone.0280041 (PMC9821785; doi:10.1371/journal.pone.0280041)
Supplement: S1 Data — (ZIP) [file pone.0280041.s003.zip › SP_10_NSW.pdf]

Interviewer: OK, we're starting the interview at (SERVICE NAME), (DATE) , it's 10:15 am. I have (NAME) and (NAME). Is that how it's pronounced?

Respondent: Yep.

Interviewer: OK, so I have a lot of questions here. Because there's two of you, I'll give you both an opportunity to answer each question. And if there's things that we've already covered, we'll just jump over those ones. It can be a little bit of doubling up. OK, so before we begin, for the purpose of this interview, when I refer to newly arrived migrants, or just migrants, I'm actually referring to people that have arrived in the last five years, and that includes both forced migration such as refugees, and voluntary migrants, OK. So the first set of questions are about the services you provide at (SERVICE NAME) So could you start by telling us about the services provided by this organisation that assist newly arrived migrants?

Respondent: I'll go first? So I work primarily with young people aged 15 to 25, so newly arrived people from refugee backgrounds. So I help them with education, employment, vocational training and sports activities. So I help them with resumes to get a job, or finding work. I help them apply for scholarships or give them advice on courses that they can do, and I also organise training sessions, so white card training, student teacher training, first aid training, anything that helps them get a job. And a lot of time sports is providing, it's the minimal part of the project, but providing some sort of recreation activities for them. We run our learn to swim program, art classes, yoga classes, just depending on what they're after. I also run emergency relief. That's open to everybody in Australia, in our local area that is, but we do provide financial support from people from refugee backgrounds like helping them with bills, or food. And clothing.

Interviewer: Fantastic, thank you.

Respondent: Yeah, main one is the covering for the SETS program. I think settlement engagement and transition program. That's from 2019 up to 2022. We're working for, there is two parts of this, the clients that we send, communication capacity building. There's priorities in E.T., employment education and English. This is priority.

Interviewer: Yes, I've been hearing about the Three Es.

Respondent: Yes, the Three Es, focusing for that, and also the community capacity building, targeted for refugee migrants, humanitarian needs, focusing on this already. Higher needs client, very good for the migrant. Once we do form a group, information, we give them information about the programs, assistance how they're going to get a job, how they're going to get housing, how they're going to get for example Centrelink income. All that information needs to understand. It is a result, because new arrivals, they don't know what to do, where to go. That is they are coming in our centre, we need to work together to show them and give them information. And mostly specially focusing on higher needs. Language and financial is the most higher edge, because if you

don't have any communication skills, how are you going to go outside to communicate, as they're going around without any, that's why we are here to support them. We organise English classes, we organise sewing class, we organise for computer class, we organise for gardening knowledge class, we organise citizenship class, we organise there's a housing program, legal, all the settlement needs, we organise a program. That's what we're focusing, especially to find the needs through the casework. One client who comes to me for example for assessment goes through this higher needs, and once when I [inaudible] she has got domestic violence or she has got housing issues, she has got something, I need to target her program first. That's why according to her issue, we need to do it. As I said, information, advice and advocacy, we have them writing the letter for the Department of Housing, advocates, we have them, they have got assistance program, how we're going to help them, working as service providers to solve the problem. That's what, access to medical services, this is what we're doing, casework and programs and projects. And mostly the outcome is successful settlement, needs to be there. Personal independence, of course productive labour, social engagement, wellbeing, community connection. That's what we do.

Interviewer: Excellent, well you've actually segwayed right into the next question.

Respondent: Already.

Interviewer: The next two questions are about other organisations that you work with. So can you name some of the relevant services that other organisations are providing, in terms of helping newly arrived migrants, and then also something about who you collaborate with to do the work that you're doing?

Respondent: As I said before, we're working with all, for example as soon as they arrive in this country, they need to register, yes, Centrelink, Medicare, SS, everything. But the Centrelink is hard work. We're working closely with Centrelink because there is some forms need to fill it out, and some information doesn't understand. We're working closely and sometimes we're there to provide our service, and they're coming to provide information. Housing, hospitals, employment providers, legal aid, immigration agent, we do have our three case workers in here, because a lot of migration problems, because they left behind their families, they want to sponsor. Even they don't know how to apply or have any information. We have the plans, we're working closely with all. With the community, with other service providers, housing, education, TAFE, universities, the same like you, doing the research for last time for the Afghan community, we do that researching program with the Afghan community with the Western University in Sydney. I think we're working for all the ones in settlement needs.

Interviewer: That's great.

Respondent: Sure. Youth Off the Streets and (SERVICE NAME) run a mentoring program for young girls every week. It's not just for refugee people, but a lot of our clients go. We have a close relationship with local employer, (NAME OF LOCATION) Aquatics Centre or (SERVICE NAME). We have a called

academy with them, which is like a refugee learn to swim and employment program, which gives our clients free swimming lessons and then employment opportunities. We help fund the training and then they get jobs straight away. We work with Dress for Success and Dress for Work, which run employment workshops for our clients and give them free clothing for interviews. We have very close relationships with local schools and (NAME OF LOCATION) Council. (NAME OF LOCATION) Council provides us with a lot of materials and services for our clients. Local youth centres as well, like (NAME OF LOCATION) Youth Centre, we run lots of activities together. They run free training, like hospitality training for young people.

Interviewer: That’s great, thank you.

Respondent: What are the businesses that are on in (NAME OF LOCATION), in our area too, for the clients to be a volunteer, and they offer them that.

Interviewer: Great, especially because there are so many businesses here run by people who are migrants themselves, and who probably speak the same language, it makes it easier.

Respondent: Yeah, we’re working with religious leaders becomes some of the clients, they believe in their understanding is different from us because of they’re coming from, and they trust to come and really face the leaders. And sometimes they get counselling from them, that’s why we need to work closely with them. That’s what we do. And the same with community leaders, we do have community leadership round table quarterly, every three months, for all communities, run the training. We do that to educate them and to empower them to give the skills how they’re going to register as a community in this country. That’s what we do, and how they want to start a small business, we’re working with Australian Taxation Office how to run the small business, how to register for A.B.N., all that kind of things, that’s what we do.

Interviewer: Fantastic, and are there any organisations that you choose not to work with?

Respondent: No, as long as there’s a need.

Respondent: I don’t think we’re that specific.

Respondent: Because there’s needs, the client needs his help, that’s why we are here for the client.

Interviewer: And of everyone I’ve asked that question, no one’s ever given me a yes. It’s like no, we’ll have help from whoever. Fair enough. Are you aware of any services that are needed, but not available?

Respondent: Because of the limited, for example we’re working only five years, but mostly the new arrivals, it is more than ten years, seven years, eight years. Still, they need us there, but we are very limited to serve them. We’re not saying no, because we give them information and refer as an organisation, but they need,

once the country is opening the door to come to this country, the need is there. But our limitation is five years.

Interviewer: Five years, I'm hearing the same thing again, by many.

Respondent: Did you? This is because a number of clients come in our door, and ask us. We said we can't, we are here for five years.

Respondent: By the time they've settled, then learned English, then done their course, the five years is up, and then they're looking for a job.

Interviewer: All that initial H.S.P. program is up to 18 months, and a lot of people are saying that should be separate from the five years.

Respondent: Yes, especially some of them have issues, even then coming to learn English. They're staying home one year, two years, three years. By the time it's gone.

Interviewer: That's a good point, that's a good point.

Respondent: Yeah, housing issues, family breakdown issues, it's different issues, stuck there when they arrive. Some of them it's very hard to adjust, to understand, some of them, because of living behind their family, depressed. And I think there are a lot of things coming up, and the time is finished. Three years is finished, by two years, how are they going to get a service? These kind of things happen, through our experience.

Interviewer: Good point, thank you.

Respondent: And more support for asylum seekers, because we are very limited by the visa number.

Interviewer: And because of that, because this study is settlement service literacy, it excludes asylum seekers because they're not using these services, because they aren't eligible.

Respondent: I think like some sort of very comprehensive service directory would be useful. I haven't come across such a thing.

Interviewer: Great. And are you aware of any services that are over utilised? Like there's a high demand, long wait times?

Respondent: Housing. High demand still, financial. Even the financial, because there's no job, employment, it's breaking for the family to lead them to health, for the hospital because of this reason. Housing rental market is very high, they've not getting it easily, ten years, five years, seven years to wait. And the Centrelink they can afford to pay, because of that there's no job.

Respondent: Housing, employment. The legal aid lawyer is booked up until April.

Interviewer: There you go.

Respondent: Because of, see, family.

Interviewer: And on the other side of that, are you aware of any services that are underutilised, like there's not a good uptake on them?

Respondent: As an organisation you mean?

Interviewer: In terms of the services that you provide, or the ones that the other services, other organisations are providing? Something, anything that's not really used well?

Respondent: For us, what we link them, we refer them. Because as you said, large number, maybe the queue migration of course up to next year, because there is a demand. And there's outreach only one day a week coming to our organisation, is that right?

Respondent: Which outreach is that?

Respondent: Migration and legal aid. Once a week, hey?

Respondent: Yeah, it's only once a week. That's very utilised.

Respondent: Yes, but we refer to them, in their office on (NAME OF LOCATION) and still the problem is there, migration is a problem there, housing is a problem there. Employment is a problem there.

Respondent: It also depends on the age group. Like often youth info sessions aren't well attended, even though they'll be very beneficial for them. Also it's because of the school hours and that sort of thing.

Interviewer: OK, thank you. So can you tell us about some of the methods you use measure the effectiveness of the services you provide?

Respondent: Client feedback and outcomes.

Respondent: Yeah, feedback. We refer stories, they share their stories, their experience, and feedback. Yeah.

Interviewer: Great, thank you. And can you tell us about any other issues regarding access to settlement services that you've noticed that migrants are facing?

Respondent: I think just lack of knowledge about what's there, and what's available and what they're entitled to.

Respondent: That's right.

Interviewer: Thank you. OK so the next set of questions are around how migrants adjust to Australian culture and society. So can you tell us about your understanding about how migrants you work with understand Australian culture and society?

Respondent: That's why we do, this is our job to understand, as long as they are coming to Australia, that's our job to do, understand about Australian culture and society. We need to link them to the wider community and society. That is our job to provide. That's why we organise all services for them, understanding about Australian culture. This is our job, that's what we do all the time. We are running citizenship training, we are running all projects, they are running every day. I can't say that. Afghan, Arabic, Tamil, and multicultural groups, we run these programs in conjunction with our library or (NAME OF LOCATION) Council because of this need to understand about the system and culture. That's our job.

Respondent: I think they think when they come to Australia they'll have lots of opportunities and support. I think it's a little bit more difficult when they get here, actually adjusting. I think they need more support in terms of understanding what help is available and they can go, and that's part of our job is referring people.

Respondent: Yes, it's part of our job.

Respondent: But if they don't come to us, then we can't refer them.

Interviewer: And to what extend do you see your clients being exposed to Australian culture?

Respondent: I think the government tends to send them to specific areas in terms of housing, so I think often community groups stay together, which is really good for them adjusting. But then a lot of young people that I work with want to make Australian friends, but they don't really have that… like Australian born friends, but they don't really have that opportunity because they're living within their communities. Which is great, but also they don't have the opportunity.

Respondent: Even the focus about one step. We organise one step program quarterly. What we are doing, there is Australian people around North Sydney. We're working with them, taking our client, Afghan client, Arabic clients, to meet with Australian people, and spend hours and hours to communicate. This is our program.

Interviewer: That's excellent, I haven't heard a program like that before, that's really good.

Respondent: And going back to gaps in services, a lot of my young people would like a buddy program, where they're matched up with an Australian student that they can be their friend and get shown around and shown basic things like how to use an Opal card, that a lot of young people, they don't know because it's not the same as where they're from.

Interviewer: Excellent, thank you.

Respondent: They can share their experience, and they're very excited, Australian people, because they don't know about Afghans, and they share their history and they are very surprised. Oh my good, this is a very good opportunity. And some of them, they have a connection with each other, like friends, and family. This is what we do, this is our job.

Respondent: The one step business.

Interviewer: And what are some of the opportunities provided to migrants to practice their own culture and practices, cultural practices?

Respondent: There's a community kitchen very week in the (NAME OF LOCATION) Centre for Community, down the road. So every week they have different foods and activities from different cultures, and people are invited to share their cultures.

Interviewer: Beautiful.

Respondent: We refer the clients to there. Also we participate for their new year. They celebrate their new year, they celebrate their holidays, what is the Afghan…

Respondent: Yeah, whichever the local culture…

Respondent: But our worker will always be there.

Interviewer: Excellent, and then of course there's Harmony Day, Refugee Week.

Respondent: Yeah, we have refugee youth awards during Refugee Week and we invite young people to share their culture in performances, and we pay them to do that.

Respondent: There's national day.

Respondent: Yeah, we invite people to share their cultures on national day.

Interviewer: Fantastic. And what are your impressions about how migrants are being recognised and respected by people in their community around their cultural values?

Respondent: In their own community or in Australia?

Interviewer: I think just in this community in particular. There's a mix obviously of people from different cultural backgrounds sharing their own cultural practices and values.

Respondent: Yeah, they do. That's why we organise the same, what we said. And we educate them to respect how they live in harmony. Everyone has got their belief, their ideas.

Interviewer: So it's a bit bigger than just adjusting to Anglo or Australian culture. It's also being exposed to Indian culture, all these other cultures as well. Different beliefs.

Respondent: Yeah, even in our community leadership program, one of the speakers, he's a pastor, and he said that we need to acknowledge each other, we need to respect each other. The next generation, he gave the example, if my son comes to me and wants to marry the Australian girl, because we are here, I would say yes. Because we are in Australia, go for it if you are interested, if you are happy. We need to educate them instead of to live on our own, because we are here, in Australia. And all are happy, because we need to educate this kind of understanding. Some communities, they have their own culture, they hold their culture, but when it comes to Australia, they need to open up their mind, learn about for other communities, other society, and improve. We need to improve, we need to learn more in this country, that's what I see. We teach them to respect this.

Interviewer: Fantastic. The next set of questions are around migrants' sense of belonging and inclusion in Australian culture. Now, this might overlap with some of the responses you've already just given. Can you tell us about the programs or supports available that help to create and enhance migrants' sense of belonging and cultural inclusion? Is there anything different? I know you've already mentioned your sewing groups and all those sorts of groups, and then the events with the kitchen and the youth programs.

Respondent: I think employment. Once they get a job, the change in them is quite profound, and you can really see the difference in how they feel like they finally belong. Because a lot of people's identity is tied up with work. And once they get a job, they really feel like they belong and they have that new environment when they can make friends as well. A lot of local organisations do awards. We talked about refugee awards, but other organisations do that as well, and I think that gives people a sense of belonging. Like we took some clients to the Fakir Conference in Tasmania as well, and that really was empowering for them.

Respondent: I think the sense of belonging is starting from the casework. That's what I can say. Once when they see welcoming, we stretch out our hand in our organisation, and that is the first thing. To feel safe, some hears their problems, someone to walk with them, someone to direct them where to go, what to do. That's what we're doing, I can say that.

Respondent: They feel valued.

Respondent: Yeah, valued.

Respondent: And heard.

Respondent: When we find a house, or they find a house, I remember one feedback is, I feel now it's my home. That's what they say, when they found a house, when the job actually stayed, when they go to English classes, no. When they find some

friends for Mum, we have playgroup in here, and we're working with the schools. Now we've moved for different schools. Running the playgroup. All the mums come together, they share their experience, the story, and when she's pregnant, she shares for others, pregnant women, young women, this is what we're doing. They feel they belong here, this is my home. This is what we do. The programs, always we're creating for our clients, that's what we do.

Interviewer: Great, thank you. And the next set of questions is around, and you've already touched on some of these things, programs that are responsive to social support and improving health of migrants? So maybe we'll just start with the social support ones. You've already mentioned the vouchers and the casework, all those sorts of things. Is there anything else you'd like to add to programs around social support?

Respondent: We do excursion to C3 Church, which provide free brand new clothing items, free food, so they give us a bus and they take a couple of busloads of our clients to the church and give them things.

Respondent: We do an excursion to link the historic places for our clients, mostly sisters to learn about Australia. We do that in our project and program. Also when they ask us, the organisation for programs, the services, most of the time are part of capacity building. We support them. Yeah, social, we'll do programs as part of capacity building program under SETS, that is one of our, working as a community.

Interviewer: What about health and wellbeing?

Respondent: Health, we're working closely with the health services. Even last time, we hosted one of the health programs in our organisation, coming from (NAME OF LOCATION) Hospital, coming from (NAME OF LOCATION) Hospital, even invited the nurses, midwife, for our community to provide information to our community. This is what we do, always we're running this kind of program, and are aware we need to check up the women, some of them don't want to, because of the title, because of the culture, but we're breaking that, and it's very important to check for that, the breast and pap smear, all that kind of thing. All community, we do, that's our job. That's why we organise the guest speaker to come.

Respondent: Yeah, and for the youth, we combine guest speakers with game days so that young people are encouraged to come. We had like health information stalls at our youth summit, and then we do sports programs, we do like mindfulness, yoga and dance with an instructor who is trauma informed, so she used language that is mindful that they may have experienced trauma. We do a sports program which helps them connect with other young people, with swimming, and it's good for their physical health and mental health, just having that space. Particularly for the girls, the have the program course, so they've got a curtain, so they can wear what they want, and it's girls only.

Interviewer: I remember when they first set that up, it was like wow, that's ground breaking.

Respondent: Yeah, it is, they've been very good. Just seeing the girls in that environment is really good, really good for them.

Interviewer: OK, so the next set of questions are around financial literacy and income generation, those sorts of things. So can you tell us about programs available for financial literacy and managing money effectively?

Respondent: We have a financial counsellor who's available for appointments. I have a brochure for emergency relief, but I think a lot of people think that electricity is going to be free in Australia, and it's quite a shock to them. So we provide IPER(?) vouchers, but they're only available every six months, so I work on like a one on one basis on casework, as emergency relief, just doing one off, trying to get them to set up payment plans and understand budgeting, and the referring them to other services, like the financial counsellor here, or St Vincent de Pauls.

Interviewer: Great. What kind of financial challenges do your clients face while adjusting to life in Australia?

Respondent: A lot of them are on Newstart Allowance, and that's not even enough to pay rent, so they're really below the poverty line, which they shouldn't be when they've come to Australia. And then looking for housing, and a lot of people come from very large families, so some of my clients have like six kids or eight kids, and trying to find accommodation that's suitable for them, and within their budget, like it's impossible. Especially under the Newstart Allowance. Just paying them a decent amount is a very small investment in what they're going to return in the future, if you're looking at it purely financially, when they get a job and stuff. It's just a big challenge to afford basic living expenses.

Respondent: Also they're seeing specialists regularly, they pay a lot of money for the specialist, because it's regularly the specialist seems to pay, they refund one, they're always buying the medicine and paying this one off. This is one of the big issues.

Respondent: A lot of my clients have injuries. Or they've been shot or paralysed or lost their legs. So it's very difficult.

Interviewer: Wow. And are you aware of any culturally specific dynamics that impact and challenge the management of financial demands? Like sending money home or dowries or things like that? Gender imbalance in terms of managing money?

Respondent: Quite a few clients send money to their families. Though they're in poverty themselves. They're always sending money back home.

Respondent: It's a problem for them because they left behind the family, the children or the wife. They send them money. And also the application fee, it's hard for them

to apply for. When they apply, they need to pay, for example if they're going to marry, how much is it?

Respondent: $7,000 for the partner visa.

Respondent: Exactly.

Interviewer: Wow.

Respondent: And where's she going to get, or where's he going to get that?

Respondent: And then if it's denied, they don't get it back. I've had clients like that. They've paid it, it's denied, they're still in poverty and their partner is not here.

Interviewer: It's horrible.

Respondent: And for women who are facing domestic violence and they leave their partners, the threat of we'll take your visa away, even though that's not legal, and they don't have separate bank accounts, or their own money, is very difficult, particularly for women.

Respondent: Yes, it is.

Interviewer: And in terms of these challenges, are you aware of any, other than the financial support provided, any other organisations that support your clients through these challenges?

Respondent: It starts off with mental health support.

Respondent: We do that, we start, and charity organisations around in our area, like St Vincent de Paul or Salvation Army, Welcome House and Independent Mission, all that is around. We work in closely with them.

Respondent: Domestic violence support. We have a family team that helps families.

Respondent: And Speak Out, we work in closely with that too.

Interviewer: Great, thank you. So the next set of questions are around the support your clients, for your clients when they face legal challenges. So we've touched on a few of these things already. But can you tell us about programs available for your clients with legal issues around identity, visas, inviting family members to Australia? So legal aid, obviously.

Respondent: That's what we said, it's a long queue, it's a long waiting list. We do have [inaudible, 34:55] for migration from legal aid. But there is a problem. The problem is they're not filling the form. They give advice, and they lead them to what they need to do, but no one helps them to…

Interviewer: Fill the form.

Respondent: But I had one yesterday even that is I.A.A., Immigration Australia…. There is the support. We'll find out, now we're going to refer the client to them. The other one, I got it, I think you know that?

Interviewer: I think I know what you mean, I think I've got a brochure somewhere.

Respondent: I think some days at (NAME OF LOCATION) Library they do it.

Interviewer: Is this to help them fill in forms? Because this has been brought up by quite a few different service providers.

Respondent: The youth program we're not supposed to do anything related to form filling or Centrelink or citizenship applications, but a lot of young people need that.

Respondent: Just give me one second, I'll bring it back.

Interviewer: I can get it later. I can get it later at the end.

Respondent: Yeah? This organisation in the future we will refer to them.

Interviewer: It will be good to have that documented in the research.

Respondent: There is a help there to identify who it is in our catch up meeting. There is services, the client outcome.

Interviewer: Because I think a lot of other services will appreciate that, to know about that. And what about programs around, or supports available for people who are experiencing physical violence or other forms of violence or discrimination?

Respondent: It depends on their specific issues. Refer to the police, to the family law court.

Respondent: Reporting, all those channels, we refer to all those kind of things straight away when we find out issues, report them. We are aware.

Interviewer: And what do you think the key laws are that migrants need to learn when they first arrive in Australia?

Respondent: Like…

Respondent: So related to I guess acceptable behaviour towards your spouse and children, because there's a group that are stricter. The road laws, like littering, paying for transport. A lot of young people have fines for not swiping their Opal cards.

Respondent: They need to understand the systems.

Respondent: But also their rights, they need to know their rights when it comes to interacting with police and what they're allowed to do and what they're not allowed to do.

Respondent: They need to know yeah.

Interviewer: That’s correct. And any key laws for, within the first five years? I suppose it’s more of the same, but anything more you’re like to add?

Respondent: Particularly related to domestic violence, I think.

Respondent: Migration specific, and legal, they need to know more, different kinds of family law, they need to know all these things.

Respondent: And they ask often if they can leave Australia on their visa and stuff. The clear clarification of what their visa means they can and can’t do.

Interviewer: Great, thank you. And in your opinion, what is the level of awareness of migrants to accessing key legal services when they need them?

Respondent: What they need to know.

Respondent: I think a lot of people struggle with knowing where to go. We’re like the fist point of call for a lot of them, so we’re responsible for referring them. I’d say low knowledge, particularly when they first arrive in Australia.

Interviewer: And the kinds of challenges they may face when they access legal services? I know you’ve already said there’s a high demand for them.

Respondent: Language.

Respondent: Yeah, language. Cultural difference, cultural, because where they come from here, is absolutely different. It’s really hard to accept one, even one year, two years, three years, they get all this, especially when the come above 50. It’s very hard to change then and understand. We need to work hard with them. The children…

Respondent: Yeah, the young people are much different.

Respondent: They pick it up straight away. Think about someone who comes, he’s a doctor, same like what we learned, he’s a doctor, he’s a respected person, he has got good money, he has got very good income, he’s got a family. Once, everything’s lost and he comes to Australia. What do you think for that person? Because he carries a lot of things. One by one is to take out the financial problems, family issues, and housing issues, emotional things. All these things, and to take it out one by one, it takes how many years?

Respondent: And they can be very…

Respondent: Needs to deal with everything, emotional, he covers everything, financial, he doesn’t have any family members around him. This is what we’re working with.

Interviewer: That's great.

Respondent: And a lot of older women can't even read or write in their own language, so that's…

Interviewer: And then there's an expectation that they become literate in English.

Respondent: Yeah, so it's very difficult.

Interviewer: I've heard that a lot too, in terms of even with the English courses that are available, they don't really take into account your status, whether you have literacy in your own language. If it's your first time…

Respondent: And then expecting to understand legal terms and laws is a bit ask.

Respondent: We teach them instead of watching, some of them they are watching Arabic T.V. You know what I mean? They're disconnected with Australia, information, all that. Even something happen on the T.V. they are there. Who's going to tell them the information? Tell me? We educate them. They need to see the news, at least to know what's happening around. If the fires comes, who's going to tell them, if they're watching Arabic or Chinese or whatever? This kind of, we educate them now.

Interviewer: The next set of questions are around movement of your clients from one place to another. So what do you think the key reasons for the movement of people from one place to another, or one suburb to another?

Respondent: Financial. Because of the rent, and because of the community. Because they want to secure rent, but the main thing is finance. They want to be for the house, they need to access affordable housing.

Interviewer: Sure. And are you seeing any trends in mobility, possibly in the early years after migration or after several years?

Respondent: I don't think I could say. I don't know. I mean a lot of my clients have been in the area, they've just stayed here.

Respondent: Because of employment, some of them moved. That's what we know for our clients. They couldn't find a job here, so they moved there. And if they are coming for longer, they come here because of the housing service or available service in the area. Because of the service here, we already see from Wollongong.

Respondent: And some people come all the way from Newcastle to see our caseworkers here because they don't feel like they get the same service in Newcastle.

Respondent: Yeah, because there is no service around and also the financial problem.

Respondent: Finances are the big one.

Respondent: The big one.

Interviewer: Thank you. OK, the next set of questions are around education and literacy programs. We're there. Can you tell us about services available to migrants using your service, that's badly worded here, in terms of school education for their children? So you've mentioned the connection with schools and playgroups and things. Adult literacy programs or any other educational literacy programs.

Respondent: Sure, we have Homework Help every Tuesday, which is for young people. Sometimes the young people come to me and seek individual homework help, which I give.

Respondent: Individual Arabic, Afghan, multicultural and Chinese, regularly, two times a week for our clients. When they finish, we're working with Granville TAFE, Ultimo TAFE, they're coming to one for the programs, English classes. And even we run Certificate III programs, the courses, trainings, education and training, first aid training. We work in partnership with TAFE. That's what we're doing.

Respondent: And we help them apply for scholarships, help them know what they can do. Because a lot of people don't know. They have these great goals they want to achieve, but they don't know how to get there, so we help them, show them what courses they can do.

Respondent: Especially the youth groups, help them with their resume, and take them to the employer to show them around, which kind of job available.

Respondent: And we write them recommendation letters for scholarships, or whatever education things they need.

Respondent: That's what we do.

Interviewer: Oh good. And what do you think some of the key issues or barriers are for children of your clients to accessing school or university education?

Respondent: A lot of my young people feel like they can't go to university because they need to work fulltime to support the family because their parents can't work. So that's a big barrier for them, and if they do go to university or further studies, I've even got like people who just finished H.S.C. and they're the only person in their family working, so there's a lot of pressure on them to work. That's the challenge for them.

Interviewer: Wow.

Respondent: That's not [inaudible, 46:49] the family doesn't give in to them for whatever, because they want to…

Respondent: Yeah, and if they're on a 101 visa, because a lot of, the Dad comes over and then sponsors the children, and the wife or partner. Because of the visa,

they're not eligible for the HECS help loan, so they have to pay their fees up front, as far as I'm aware, which is a big barrier, if they're paying that much. Because they're not paying the international fees, but as far as I'm aware, they're not eligible for that one.

Interviewer: No, I haven't heard that one before, that's great, thank you. And can you tell us about any special packages or subsidies provided to support educational opportunities? You've talked about applying for scholarships and things like that. And the 101 is actually the opposite, it's not a subsidy.

Respondent: That we provide, or just that we're aware of?

Interviewer: That you're aware of.

Respondent: There are equity sponsorships at most universities for refugees, SSI Offer scholarships. Each university has their own set of scholarships available. And then there's Career Seekers, which provides internships for refugees after they're in university, they're paid. I think that's about it.

Interviewer: And are you able to outline any kind of employment opportunities that you're aware of that are offered to migrants children when they finish school or university education? So you've already said those internships.

Respondent: Yeah, Career Seekers, what's that other one? There's the Ignite Small Business one with SSI. We have our specific program with (SERVICE NAME), which they're hoping to expand nationally, which is an employment program for young people from refugee backgrounds.

Respondent: The one you're working with, with leadership.

Respondent: Yeah, they do the training, which is low cost or free, depending on your visa. And they help with work placements.

Respondent: Work placements, and give you training and then give them the job in a city area, café.

Interviewer: Is that for young people only?

Respondent: Young to old.

Interviewer: Everyone.

Respondent: Yeah, everyone.

Respondent: The (NAME OF LOCATION) Project do a similar thing, barista training. There's that other one for refugees who have graduated. What's it called? They help them find jobs, and they match them up with employers. It's like a specific website, like a job seeking website for refugees, I've forgotten what it's called.

Respondent: We're working with this Ignition, the one they run, the training gives them an opportunity for work placement, and then they got a job.

Respondent: (SERVICE NAME) does the same thing. They give money to (NAME OF LOCATION) Youth Centre, which we have a partnership as well, to train them in hospitality and then they employ them at their venues.

Interviewer: So there's quite a few, that's great.

Respondent: There are.

Respondent: Building partnerships.

Respondent: It's still limited.

Interviewer: I can see that, in the type of work?

Respondent: Yeah, in the type of work it's very limited. A lot of them are suitable for part time jobs to support them while they further their education and get to where they really want to go. They're not careers that they want forever.

Respondent: Often business areas are sealed to you. We're working there.

Respondent: More like survival jobs, rather than what they want to do.

Respondent: In U.N.S.R. they employ some clients, they need bilingual workers to promote that on the street. They are coming to our organisation, what can we bring, as much as we need to.

Interviewer: And have you got any comments about, it's always a big issue, recognition, qualifications from overseas?

Respondent: It's so heartbreaking to see someone who's 23, who's done university and has to go back to high school. So degrading for them. But they have to go back to Year 10, even though they've completed university. It's very difficult. And some universities have pathways that they can go straight into university, but if the language level isn't there…

Interviewer: See, that's not often mentioned, in terms of school level. It is for university or professional qualifications for adults. Not often for children. High school completion in their own country and then not being recognised here.

Respondent: Yeah, a lot of young people I find are in that situation. It's either one of two extremes. Either they didn't have any education at all in their home country, and then they're rushing to catch up in a different language in Australia, or they've finished it all and have to go back. It's just really, breaks your heart.

Interviewer: And in terms of people with professional qualifications, have you…

Respondent: Yeah, lots of difficulties. They don't have local experience. It's really hard. You can try and make it stand out in their resume, and you show that they've got all this experience, but often they don't have the paperwork to back it up. Or the people that they worked with, they don't know where they are anymore.

Interviewer: Very tricky. OK, so after all that, overall, what do you think are the key challenges that migrants you work with face while adjusting to Australian culture, and settling in Australia? What's the key challenges?

Respondent: Communication, language, key challenge. Once you can communicate, I think everything's… key challenge. As I said to you, above 50 doesn't speak, doesn't go anywhere, stay home to talk with his wife, there's no social. There's no other person around him, unless they speak his own language. That's why communication, once when we learn the language, this is what they learn. And confidence. Once they leave their country, they've lost their confidence. It's the same like me, I've lost my confidence when I arrived in this country. You start from scratch. My expectations were very high, and then confidence. When we not communicate, whilst my confidence is building, I can do this thing, I can do that, for me this communication. And housing as we said, housing and finance.

Respondent: More financial support so that they can be comfortable. More job opportunities. More scholarships, particularly from the 101 visas, the family stream visas, and the services in the area, they offer amazing programs, but they're in high demand. We need more funding to support more programs so that everyone can access them and know about them and get more support. I think just raising the amount they get from Centrelink is really important, so that they can be confident and comfortable and secure, which then gives them the mindset and ability to focus on other things, rather than just survival.

Interviewer: Great. And the last question, what would you like to see, you've already touched on this just then, as possible solutions to helping or support migrants to adjust well to life in Australia?

Respondent: I'm not sure if this happens already, because I don't work with SSI, but a very clear orientation package that shows them specifically what's available and what they can do, and a very clear program for resettlement. These are the English classes you're able to take, but without any sort of mandatory requirements for payments for Centrelink, anything like that. Just being very clear, these are the pathways that you can take to succeed in Australia and resettle.

Respondent: Pre-orientation before they arrive. I think pre-orientation in the country. They do that I think. Do you think that is enough? I don't think so.

Respondent: That's my feeling. I don't think so.

Respondent: There, not here. There.

Respondent: Because they see SSI first and other organisations, and when they come to us, if they don't know how to use an Opal card, and they're coming to us, they've already been here for quite a long time. So I think there needs to be more funding for a very clear orientation program. And like a mentoring program.

Respondent: Yes, before they arrive.

Respondent: A mentoring program with local Australians so that they can get a local connection and make friends.

Respondent: Before they arrive.

Interviewer: That's a good idea.

Respondent: And I think that would also be beneficial for local Australians who often have prejudices against newly arrived people. It's because they're not exposed and able to make friends.

Interviewer: Great, fantastic. I think, what time is it? It's almost an hour that we've been doing. So thank you so much for your time. Is there anything else you'd like to add, that we haven't covered, that you'd like to…

Respondent: We've covered, I think.

Interviewer: We've covered a lot of ground.

Respondent: They all want to do really well in Australia, and they've got a lot of ambition. They just need the opportunities to do it.

Interviewer: Thank you both for some amazing points there, that I haven't heard from other providers, so that's excellent. I really thank you for your time.

Respondent: You're welcome.

Respondent: Thank you.

Interviewer: We'll end the interview at 11:13.

Respondent: You'll send it to us? I'll pass it to my CO for information.
