# Supplementary material for: The importance of information acquisition to settlement services literacy for humanitarian migrants in Australia
Source: PLoS One. 2023 Jan 6;18(1):e0280041. doi: 10.1371/journal.pone.0280041 (PMC9821785; doi:10.1371/journal.pone.0280041)
Supplement: S1 Data — (ZIP) [file pone.0280041.s003.zip › SP_10_Victoria.pdf]

Interviewer: Alright. (SERVICE NAME) with (NAME) and (NAME). And interview start 2:25. Alright. So let's go back to what you were saying then before we started this recorder. Can you tell us a little bit about the services that are provided by (SERVICE NAME) that assist newly arrived migrants?

Respondent: So in terms of services and the area that I'm working is involved with the settlements, the SETS. Yeah, SETS programme. In addition to that we also have an asylum seekers support programme, which is supporting asylum seekers. It's not very… it's a limited programme because it's more philanthropic. It's not funded by government. So asylum seekers, like nowadays when they are on bridging visa there's no publicly funded programme. And then we have some houses provided by philanthropic entities and then we just accommodate them for one year with the aim that they settle well and then enter to the private market. We also provide some income and other support based on whatever we have. But our main area that I am in involved is the SETS, which is Settlement Engagement and Transition Support. And it's funded by the government. It used to be managed through the Department of the Human… Department of Social Services. But now the portfolio has shifted to the Department of Home Affairs. So they are the main funder. And then from (SERVICE NAME) perspective, so we are one of the grantees for SETS implementation, and then (SERVICE NAME) Melbourne together with (SERVICE NAME) (NAME OF LOCATION), so we are the awardee and we got the grant. So one of the locations is in (NAME OF LOCATION) and then other locations are in (NAME OF LOCATION), which are located in (NAME OF LOCATIONS). So in different locations we have got settlement workers engaging with this population and try to support them in line with the grant.

Interviewer: So for SETS eligibility, it's new migrants within the first five years and you have to have a humanitarian visa?

Respondent: Yeah. So we have got a list of eligible visa types. I can show you if you want, but based on that. And also whatever is, there are some differences, whatever is in the grant agreement. So as you mentioned, yeah, so one of the eligibilities, they have to arrive within the last five years. So even though we, based on our work we see that some migrants because of the very low literacy and lots of problems and English language, I mean five years is not adequate. Recently we had a meeting with the department, very senior people, the settlement workers organisations, we had a sort of network meeting with them and then we had lots of arguments saying that, look, this five years is not adequate. But they still were a bit rigid saying that we're funding this for only five years, if they are not… more than five years, you have to refer them to other services, which are funded by maybe by government or others. But still the, one of the sort of recommendations of our settlement, settlement sort of partner [indistinct 3.50] is that, look, this five years is not adequate, we need to provide, we need to

extend or expand this because some do need support and assistance for more than five years.

And then on the other side, for the community, it's also a confusion for them sometimes as a group and they are like a group of society or neighbours or same community when they approach here and then once we screen them then we say, OK, look, you are eligible five years and then you are not. We can help you and then you are not able to get support because you have more than five years. And it's also a bit traumatising for that community and then confuse them while, you know, the same people, how… and then probably that client who is more than five years, maybe their needs are, in some cases, more than the eligible one. So that's why still we recommend that, based on experience, five years is not adequate and we would like the government to review this. We have to share this issue.

Interviewer: You're not the only service provider to have that feedback.

Respondent: No, I think all of them. You know, listen to them (?). In southeast we have got a network of SETS providers and that was one of the very hot discussion we had just last month or so.

Interviewer: And so do you work… are any of your clients from family reunion or any of the clients kind of economic migrants? Or is it mostly just from humanitarian?

Respondent: So generally it's… we have got a list of, I'm going to share that with you, so we just go through that one. So 200 (?) I guess classed, it's mainly humanitarian. So people who came through a humanitarian programme. And then from government side, they have got the sort of packages to help them so that they settle well. And then one of them is the settlement programme. And yeah, just the… yeah. In the programme we just offer for them. But still, that's not adequate, you know. Their needs are immense and also the funding is not adequate. So we try to, yeah, just support them. And also, partnership and collaboration with other providers is also another area. Our case workers, based on the needs, if they see that our programme is not affording maybe some support then we can refer, talk to other organisations case by case. I mean we seek support from them. And also, like I give an example, one of the areas under SETS is to support them in terms of employment so that they integrate into the workforce. So we are fortunate that at the moment we have got funding from a philanthropic entity called (SERVICE NAME). So they give us some funding under refugee job readiness programme.

So this programme will help the SETS clients sort of get some job readiness support. For example, to give them a session about job market, how is the job market in Australia, how to be prepared, how to submit your application, how to prepare your resume. And also, we cover some short prerequisites of the requirements or fees like white

card costing or a, let's say, an… in order to attend a course so that, to be eligible for that employment. So this job readiness provide that as well. In SETS maybe we may not have that adequate budget to cover those things so we are fortunate to have that with us in let's say three locations, in (NAME OF LOCATION), (NAME OF LOCATION). But if we talk about this issue with other organisations probably then they may not have this, they may refer them to other job providers.

Interviewer: OK. Yeah. Do you run any… so you've obviously got the case work through the Settlement Engagement Transition Support, do you run any other kinds of complementary community development programmes aimed at new migrants? So obviously you've got this, you've got the work job support programme, do you have anything else?

Respondent: So if you visit like our website, so we have got a range of services. But for migrants primarily our support is through the SETS programme. And yeah. So as I mentioned, the asylum seeker support programme is supporting the asylum seekers who are not eligible for SETS. So that's also a good opportunity for us. However, that area, in that area we even though that's a very touching sort of area, but because no funding by government, so funding, fundraising is one of the challenge. Anything we get from especially philanthropic and other organisations is very helpful to support them. Yeah.

Interviewer: Alright. So the following question is related to how migrants adjust to Australian culture and society and the kind of issues and challenges that migrants, new migrants, are facing. So can you tell us about your understanding of how migrants that you work with understand Australian culture and society?

Respondent: I think, yeah, migrants are, I mean, very excited if they come here to start a new life. Some of them, they may have a very good overseas education, work experience, and whereas when they come here then they see that those sort of experience is very hard to be applied because they may have sort of different sort of work structure in Australia or they may have other barriers. For example, English language and also how to apply and how to sort of find a relevant job. So I think there are lots of challenges for the migrants. These can be sort of cultural issues, also whether the migrant is alone and the family members are overseas. So that's also a lot of concern and anxiety for them. And also they may have other priorities. For example, housing could be another issue, and then mental health and the trauma they experienced based on their sort of background in their country. But in my, and based on my own experience, I would say that their challenges are immense. But the key one could be English language, employment, and also literacy of the services available. Because, I mean based on my experience overseas, overseas I mean it's very straightforward, it's very simplified system, like health system or other systems, whereas in Australia we have got a very like, let's say we have got one of the best systems, for example health system, and also more digitalised system, English language, and

this… and also on the other side, the system sort of workers, they are also maybe their cultural competency towards this refugee may not be strong enough. So there are lots of barriers. So we have got very, let's say, complicated and advanced sort of providers and the system and when the refugees come, so it takes a lot of time. It takes years for them to improve their literacy and understand that and then utilise that. And one of the ways is to communicate, communication is one issue. So then English language, if it is low then there's a very big barrier for them to understand and engage with this system.

Interviewer: So is that English language a barrier to understanding Australian culture or whatever it might be?

Respondent: Exactly, exactly. Still, because I mean culture and sort of beliefs, when they come they still stick to that one and practice that.

Interviewer: As in their existing culture?

Respondent: Yeah, yeah. Because they stick to that one even though if they are here. So I guess socialisation, workplace, place of education, and also engagement with the general population is a great opportunity for them to communicate and exchange sort of the values and then that will pave the way for them to understand each other and improve. Yeah.

Interviewer: And do you feel that new migrants are getting enough of that opportunity?

Respondent: I think so, yeah. Because when they come here, that's why through these programmes they aim to assist them to settle well. And so for example, English, we call it three Es, like English, employment, and education. So these are the key needs of them. So some of them, they need to improve their education or update that or adjust that so that they'll be eligible for some work, and also English language. And once improved, then based on our sort of team and our engagement with the community, I mean they are very passionate to find jobs. And finding jobs is a very good remedy for their wellbeing, for their socialisation, for their cultural sort of communication or cultural sort of engagement and understanding. And also some refugees, I guess in general they don't want to be a burden for the welfare system. They want to work, they want to be productive, they want to be a tax payer, and they… I mean I'm sure there is lots of evidence that refugee background people have got lots of strengths and they are more likely to be very sort of hard workers, they want to own their own house, they want to serve, they want to… I mean in principle. I mean they have to help to integrate into the Australian society and then they have to be productive. Then they will be a real Australian, productive Australians.

So otherwise, if you see them not contributing, not engaging, then I'm sure they will also not feel well, then they will be in isolation and then

they will be… these things can also pave the way for other issues, including mental health, including low socioeconomic status. So as a developed country I also suggest that when we offer them sort of asylum and humanitarian visa, we have to make sure that all those services supporting them to settle well should be well, adequately funded otherwise, you know, any gaps can have negative consequences both for service providers and for the refugees as well.

Interviewer: OK. And do you see that new migrants are having opportunities to practice their own culture?

Respondent: Yeah, I think so. Yeah, in terms of culture, generally I would say that Australian society is one of the very highest multicultural society, I mean even globally. And also Victoria, and particularly Melbourne, it's very multicultural. We have people and food products and cultures from different cultures. I guess there's no cultural sort of issues. But even though some from time to time maybe we see that there may be some sort of cultural barriers or discrimination or racism. So there are concerns raised by refugees about these issues. But as a general, yeah, we are lucky and happy that we are living in a multicultural society. But from government and from the providers and researchers and from all sort of people who are taking care of the society, I mean we have to explore and also prevent or minimise any ways of discrimination and cultural sort of differences, particularly from, in terms of employment. So like one of the concerns among the society, this population based on my personal knowledge, they are arguing that they see some sort of discrimination in terms of employment. And I guess one of the reasons for that would be maybe language barrier. And if you have more language improvement then that will bridge the, minimise any gaps, and also improve the intercultural sort of engagement and strengthen that sort of multiculturalism.

So generally, it's a very good multicultural society but we have to try our best to minimise any sort of discrimination and sort of racism and these things. Yeah.

Interviewer: Definitively. So the next questions relate to migrants sense of belonging and inclusion in Australian society and the services that you run here at (SERVICE NAME). So can you tell us about any programmes or support that you offer that help create or enhance migrants sense of belonging?

Respondent: So we have got lots of such programmes. Number one, we have got, for example, as part of our citizenship preparation courses we, with the support of our settlement workers and also community volunteers, I would like to also acknowledge that these community volunteers are very helpful. So we, what we do, we run citizenship classes because the test… so you have to be a real Australian citizen. So in order to do that you have to undertake a test and then so for some sort of recently arrived population there's barriers. So English language and also how

to undertake that test. So we offer them that, I mean classes, every year. And then they get support and then they learn English and then they pass that citizenship test successfully and then they are invited for a citizenship sort of ceremony and then they get their Australian passport. So that will also, you know, get them very excited. And then they will find that, oh, I'm now able to travel overseas as an Australian, having an Australian passport. So then they are really, that improves their sense of belonging. And also there are certain sort of employment or certain sort of rules that you have to have citizenship. So if you don't have that then still you see, oh I'm in Australian, I'm Australian but how I am not eligible. So now we realise that, they realise that that, there's a barrier. So this as an example that I give you, is, you know, showing that how we help them to become citizens.

And also, like through our employment support, if they find a job, I mean we have got examples, that they feel very sort of, you know, happy and productive and then go to the workforce, engage with others. And then that, you know, from a refugee now to an Australian, real Australian citizen. So that will also, I'm sure, give them more confidence and feeling that now they are Australian. And also when they travel or if they want to invite their kids, so not only themselves, their family members, they all will become, you know, sort of Australian citizen and they will be part of this society, contribute, and I mean they are now, yeah. I mean that shows that by these projects we make a difference.

Interviewer: Enhancing sense of belonging?

Respondent: Yeah, sense of belonging.

Interviewer: Alright. The next questions are about any programmes that are responsive to health and wellbeing. So do you have any programmes here that are currently being implemented to support health and wellbeing of new migrants?

Respondent: So in terms of specific health and wellbeing, so there are lots of health providers in our area. So they are funded maybe by government or other entities. So health and wellbeing of recently arrived people is also very critical. So I guess they are now living, they are living with disability, they may have traumas of war (?), they may have… I mean mental health should be on the top, and some other issues. So these are like specialised services and when we assess as part of our assessment, so the case worker will assess them if there are any health and wellbeing issues. So we will just refer them to the providers. For example, if there's any medical health issue or a disability, so the case worker knows who the service providers are and then give a referral letter or contact them how we can help them. So we do that.

Also from our side, like the groups we are running, you know, like one of the factors for sort of lack of proper wellbeing is isolation and lack

of support. And through these different groups what we do is we've got like a social group English, social group preparation group, English language classes. We have got some like other activity like knitting, sewing groups. And also we have got homework groups for kids. I give an example in (NAME OF LOCATION), for example. In (NAME OF LOCATION) we are running homework class, in those classes students from primary and secondary, the refugee students, they, once they finish the class they come to a community centre, which is rented by this programme, and then we have got volunteers who are helping them in terms of homework and literacy, maths, and these things.

And on the other side, their parents, they get together and then they go to kitchen next door and then there's a volunteer teaching them how to cook. And the food and raw material is provided by us, some of them taken from a food bank and some of them from our budget. So they're learning cooking, they are engaging with each other, making fun and enjoying that sort of life. So when the kids finish then the food is served for the kids as well. And then they take some home. So in my knowledge, I mean it's a good media for these sort of socially sort of deprived and isolated community to come together and then socialise and that will also improve their health and wellbeing, like especially their mental health wellbeing.

We have the same sort of group, we call them health and wellbeing group, in (NAME OF LOCATION). In (NAME OF LOCATION) also we have a similar group. They get together and then we have a volunteer cook, we provide the venue and raw material and the material needed. So they cook, they learn cooking, and also it's improving. Like today, we have another group, and then I saw that some Afghani migrant women, they came and they brought some food. I asked them, why you bring? And they said, oh we brought them voluntarily and then today's our end of year celebration, end of term celebration, we want to just enjoy, and I'm very happy to be here. So all these groups is a good way of sort of social wellbeing. But in terms of like specific advanced, specialised health, so that's out of our programme. We just refer them to other services.

Interviewer: And those programmes also would I guess respond to sense of belonging as well?

Respondent: Yeah, exactly.

Interviewer: So as well as wellbeing. Excellent. Are there any barriers, do you see any enablers or any barriers to, might be beyond the scope, but any enablers or barriers for new migrants accessing health services?

Respondent: In terms of health services, yeah, I'm pretty sure that there are lots of barriers. So in Australia, as I mentioned, we have got one of the best health systems and then a range of services are provided. Whereas if

you go to the community… and also the way it's managed is very much complicated, especially for, you know… like imagine a refugee with very low literacy. So there are lots of misunderstandings. They may not know that which services are, you know, sort of eligible for free. Because for them, because they have low income, so they want to know which services are free for them and then how can they use them and where to go, how to contact. So they finally contact, then they have a language problem, so there's no proper interpreting access for them or understanding. So I guess, I mean, I suggest that for recently arrived people we should have lots of awareness about the health system for them so that they clearly understand. We should provide them material in their own languages and also their community leaders and community volunteers should also be trained because they have got very strong community links. So if somebody has got some health issues they have to know where to go. So in my knowledge, yeah, as I mentioned, it's a very complicated health system and it looks more complicated to the migrants, so their literacy is very low. And still, the services are under utilised because of the barrier, because they may not know adequately and they have got misunderstanding and they misbelieve probably, they think that, OK, this service might be associated with a charge or fee and then they just avoid that one.

And also, especially like for women, you know, some refugees are coming from a patriarchal communities where men are the one who working and then maybe women, they may not have the green light from the husband to go out alone or they may have to, because of language they have to accompany a child to go to a service. So they may have a driving barrier, they may not have a car, they can't afford. So I think there should be more funding in terms of raising awareness about services, Australian health system. And in a very simplified way we have to sort of train and educate them and we have to promote and produce lots of material about promotion of those, promotion of these material, so they properly understand and utilise them.

And also training of the workers, health workers, around that issue. And also we have to rely, employ bicultural workers because bicultural workers are the ones who can bridge the service providers to the community. And also, culturally, some, based on my experience I mean, some refugees probably they prefer to be served by a provider who is from that same culture. They want to approach and seek help from a bicultural worker who is able to speak that language and also better, I mean in a culturally appropriate manner. So yeah, in general there are huge barriers and a lot of misunderstanding and that paves the way for under utilisation of services. And we have to improve this.

Interviewer: Alright. So the next question is about programmes available for migrants to enhance their financial literacy or income generation or managing money effectively. So do you have any programmes that specifically respond to any of those concerns?

Respondent: So we don't have any specific programmes for that but as part of our settlement programme, so we… and also, in a way, in our job within this programme, we provide and our case workers will support the refugees. For example, we have got the clients who are struggling to pay their supply or electricity bills and some of them, they are in crisis. They have got very huge bills. Like recently we ran a series of Justice Education Programme and then like six sessions over two months, every Tuesday, and then the last one, which was on the second, Tuesday, the first week of December, was on electricity and how to manage your bills and financial issues. So we had settlement workers and also other emergency relief sort of staff from our department, our organisation. So they educated the clients on how to manage their electricity bills and also how to… for example, there's a government funded electricity sort of supplier… government funded website that you put your background and then it will recommend which suppliers are the best. So we trained a group of, let's say as an example for your question, a group of Afghani and other recently arrived people in (NAME OF LOCATION) how to manage their electricity bills and, you know, supply bills.

And also we offer for some or link them to some emergency relief providers if they are in crisis. And also, for example, to my memory, we have like another client a couple of months ago that the family, they were struggling with their huge bills. And then what we did, the case manager, the case worker connected them to their supplier because they also have some grants to sort of support them financially so that they manage with those huge bills. And then on the other side, we were trained or raise awareness, just educate them how to take care of their consumption. Because some of them, like for example, in some countries water is supplied for free. Whereas they come here, they may not know, and then they end up with a very huge electricity. So then we tell them how to efficiently utilise electricity, gas, and all those things that will improve their financial management.

Interviewer: So beyond the [indistinct 34.10] and beyond the electricity bills, what other kind of financial challenges do you see arising?

Respondent: So in terms of financial challenges, I think… I mean generally majority of them are low income and they, I mean some of them, they get funding from Centrelink and they… I mean the programmes we… as an example, in collaboration, I give another example, with Centrelink, recently we organised a session for a group of migrants, how to manage their My Gov account. So that's also, nowadays I mean more it's digitalised, so you have to have an account, you have to put the details. So we… you know, people from Centrelink, they educated them how to manage, you know, those financial issues. And also referral to other services is also… there are maybe, there are some grants with no interest. Like some micro…

Interviewer: Like no interest loans?

Respondent: Yeah. Loans, really for them to do loans. And also if they have any financial difficulties, so I think our case workers also, they can review and then they can give them some advice, then they are the one that… I mean the client, they may consume, but at least our clients will give them some clues about how to manage, you know, your payments. For example, we have to educate them that OK, look, if you pay on-time there will not be a penalty but if you don't pay on-time then that will complicate. So all because of literacy because they don't know that's the issue. Also we had another, for example, client, she was… I don't know because of any reason, she had a record sort of like a penalty, like a sort of outstanding payment. So we helped. Because it's also financial management, so we educate them that you can send requests for review if you have got any financial or bills, let's say, from traffic or whatever. So our case worker is helping them how to request, submit a request for review, or how to extend a payment so that you'll be able to pay properly. So in different ways we can do that.

We can also have like IT and, for example, for another group of refugees we run IT and computer classes. So in those, for example, we teach them how to navigate the websites, how to improve their typing literacy, computer literacy. Because nowadays I mean lots of things are taking place online. So how to check your inbox, how to open an account. So all these things in general in a way that contribute to their financial literacy. Yeah. But still their needs, as I mentioned so far, financial literacy, so it depends. If the refugee has got some background literacy from their own country, so that's helping them. But I mean with some, culturally, like backgrounds or some countries, I mean the clients are coming with very low literacy, so they are even illiterate in their own language as well. So that will also, that will affect their sort of financial management when they come here. So it depends, I mean, to the background of the client.

Interviewer: Definitely. And related to that, are there any kind of cultural factors that you see impacting on financial challenges? You know, people sending money home or is there anything to do with gender and money? For instance like, I'm aware that sometimes women will be getting money from Centrelink for children into their kind of account and that might pose an issue in their family or anything like that.

Respondent: I think so, I think so. I think for example, to my knowledge I mean once, I did some review of some literature about sort of family violence, domestic violence. The literature just indicated that… I think I agree to some extent, because as I earlier mentioned, so they are coming from a country where the man was the bread winner and the woman was taking care of the house. And when they come to Australia, so they get that funding of the Centrelink to kids, direct to the woman's account. And then if they properly manage that, that's good, otherwise it can cross, trigger some violence, and then the man probably will feel, you know, some sort of trigger, sort of behaviour or

whatever. So that can be like one issue. But it's more about… I mean I just go back to my previous discussion that when we invite or bring people on the humanitarian visa, so we have to make sure, provide a lot of awareness, including, we have to aware them about the Australian sort of culture and systems. For example, we have to in a prevention way we have to educate the men. We used to be sort of… we used to be, the men the sort of decision maker in their own country. So we have to educate them that look, it's Australian society and we have to adhere to the law and then you have to be careful otherwise you will be guilty, you can end up in a court or whatever.

So yeah, that's I think… sometimes because of lack of understanding. So we have to provide adequate education. And I think with the refugees, yeah, as I mentioned, they have got family members overseas so they may send some money overseas and then that can also affect their financial sort of capacity and just, yeah, maybe financial… maybe violence or deteriorate (?). But it depends, if you're a hard worker, if you have adequate income. I mean with some cultures, they have got very big social and family, so they help each other. And I mean if they manage properly, that's fine. But in general I mean we have to educate them about a bit of skills, give them opportunity so they go to the workforce, they work, and then I mean in terms of management of their financial issues it's up to them. But we have to train them if they need any assistance. Yeah.

Interviewer: Alright. So the next question's about legal challenges that your clients may face. So can you tell us about the programmes and supports that you offer here for your clients when they face any legal issues?

Respondent: Definitely. I think… so we, as I mentioned, I mean we may not have that much adequate funding and resources to help them as part of settlement programme, yeah. So helping them to understand the Australian legal system is also another aspect. For example, as I mentioned, we just recently, in November and December, we ran a sort of six-day sessions under the sort of justice education programme, so in partnership with (NAME OF LOCATION) Magistrate Court. So we ran sessions and then, for example, the group… I think we do this every year over the last, for the last past six years. So this year was the seventh year that we did that one. So group of refugees, this year they went to the court and then the magistrate and also another person from the legal aid, so they provide that orientation about the justice system and also told them the difference of Australian justice system with say overseas justice system. Because here the system is very independent and there's no corruption and it's very fair, so we have to, I mean, give that… because some of them, they're coming from overseas where the justice system is very corrupt and then they don't trust, there's a barrier.

So this training was a good education for them to train them that, look, this is the system, and then you can use them. And also I mean

because the justice sort of system is also, sometimes for refugees, there's a funding barrier. So as part of that education, for example, the magistrate also told them that, look, if you are struggling with the income, we also have some community sort of legal, as part of community legal sort of programme we have got lawyers coming certain days to the court and if you've got some legal issues with your bills or whatever they can help you. So it's more about like, we distribute some flyers so they understand. So if they have some legal issues, they can… and also I think there's lots of cases, like the refugees, they've got some legal issues and then their understanding and the literacy is very low, they don't know that they have got such rights they have to apply. So our case workers, you know, they also train, case by case, they can just review that case, they can contact other providers, or they can contact maybe a lawyer, maybe professional in this regards, to help them.

But in general, I mean similar to health system, the legal system also looks very complicated for them. And also the costing barrier is another issue. So yeah, so we have to… I don't know. Even though there are lots of… I mean there are programmes for helping refugees in terms of the legal issues, but we also need more opportunities to take care, to help them, especially in terms of costing. Yeah.

Interviewer: Yeah, definitely. Alright, so the next questions relate to the movement of your clients from one place to another. So what are the key reasons for the movement of your clients from one place to another or one suburb to another suburb across Melbourne?

Respondent: So I mean generally we don't have a very high rate of movement because, unless they are newly arrived as part of settlement, but once they are settled then they find their own community and then they want to join that sort of community. That's what we have, for example, in (NAME OF LOCATION), we have got… I mean like most of our clients are from, as you mentioned, Myanmar sort of community and then they are working to get, living sort of close to each other and helping each other. So we have very less sort of movement, right. But if there are any movements, so we can refer them to our branches or we can refer them to another service settlement provider. But the movements also, especially (?) with a lot of challenges. For example, they are struggling in terms of changing the school of their kids and also how to change their contract for the supplier to another one. So they still have challenges, it's difficult. So it depends on the case, if they need any support they come to our settlement workers and then they help them.

But generally, I think… and also we have got clients who are coming from other states and then there may be a client from another settlement provider for that state. So it's… I'm sure as part of settlement sort of service, when your clients move to another location so you will also make sure you provide them some information, that

look, if you go to that suburb, so this is the place (?), you just call them and then they can provide you the same service. Yeah.

Interviewer: Alright, excellent. So the next question is about migrant's access to education and literacy programmes. So can you tell us about the services available here that you offer which respond to… yeah. Sorry, let me rephrase that. Do you offer any education or literacy programmes here?

Respondent: So as I mentioned, like we have got English language, that's also a contributor for the literacy. We also provide computer and IT classes. We also, for example, citizenship classes and also we have got some ad hoc, you know, sessions. For example, as part of… I mean regularly we, in line with the… I mean in collaboration with other providers, so based on the needs, so we identify a speaker and then they come and educate them on certain issues. For example, in west part (?) we have got homework groups, as I mentioned. In western suburbs we have got homework groups where kids and teenagers, like secondary and primary kids, refugee kids. They get together and then their literacy and education and maths and other issues, the volunteers help them to improve that. And also as part of our employment job readiness programme, so if the client need a course, for example, want to get like white card or a hygiene or food handling course or any sort of short-term courses which pave the way for employment, so we can even cover the costing for that even though that's not covered by settlement programme but under the job readiness programme, we can cover that one.

And yeah, I think we run a variety of sessions on all different topics, so that will raise their awareness, and they take that knowledge and then apply it, not only for themselves, for their family, kids. And yeah.

Interviewer: Excellent. So on the courses, building on that, do you see… is there employment opportunities for migrant children when they're finishing school and university?

Respondent: So employment… I guess for the kids, they are more, in a better position compared to their parents because they have got an Australian education. And I guess even in the high schools also there are programmes, career sort of team, which help them. But as part of our youth programme, we… yeah, if we have got clients who need employment, I mean we are happy to support them. But based on our sort of data, I mean majority of these barriers, employment barriers are among the parents because they have got the English barrier and they have got overseas experience and work. So I guess youth, yeah, I mean refugee youth and the young people, they, still, I mean they also have a lot of challenge. But we also have other specific youth sort of providers or organisations and youth projects. So we can also refer our clients to them and yeah.

Interviewer: Yeah. So for the parents you've mentioned obviously language skills and then also perhaps qualifications or experience from overseas as barriers. But do you see, are there any employment opportunities for new migrants, do you know?

Respondent: I think so, yeah. I mean in Australia, because migrants are very hard workers and very honest and they want to contribute, and I mean I'm sure if the employers sort of engage with them and then see their work they are happy. So there's lots of success stories about refugees who manage to enhance their employment. But yeah, I think generally there are lots of opportunities. For example, if you have some overseas background, I bring my example. So I did overseas work and overseas education and overseas experience, so for me, I mean working maybe in another sector was a big challenge for the first time. But working for refugees, refugee community, for refugee projects by a refugee, let's say, experts or workers is also a good opportunity. So there are lots of funding and sort of opportunities and projects which are aiming the refugees. So if you are from that background, because you have, you know, the language, you have other skills, you have access to them, so you also have a great opportunity to work in the refugee sort of sector. So that would be number one.

And then the second one, they are sort in the farming and the sort of warehouses or other sort of industrial areas. And they work, I mean the need is high. I'm sure there are lots of need. It's more about how to channel this human resource sort of pool of refugees to them because they, by themselves it's hard to go and then, these projects are just… for example, we have contact with different sort of job providers. We've got very good relationships. And I mean whenever they… I mean we have some [indistinct 54.05] clients who get some training and some assessment and some support. We help them to prepare their resume and we refer them and then if they are referred by us or by providers it's more powerful rather than if application is submitted because they may not have a reference. Because we can also be a referee for the client. We can… the case worker can call to contact them and then help whatever prerequisite or whatever requirements are needed so we can help. And then that will sort of improve the relationship between employee and employer and then pave the way for employment. But in general, yeah, there are lots of opportunities and the refugee population, they are very hard workers. They've got the potential and yeah, we can help. Yeah.

Interviewer: Alright. So overall, I mean you mentioned this at the very beginning but I just have to ask it again, what do you think the key challenges migrants that you work with face while adjusting to Australian culture and settling here in Australia?

Respondent: Which one?

Interviewer: What are the key challenges migrants face in settling in Australia?

Respondent: Oh, the challenges. I think lots of challenges.

Interviewer: So what would the key ones be?

Respondent: The key ones should be sort of the employment barriers, English language, and also literacy of the Australian sort of system like providers. So they have to be trained about the Australian system because it's very complex versus their own country. Also I mean funding, not adequate funding for this sector, for refugee settlement sector. We need more funding by government. And also, we have to also sort of strengthen the involvement of bicultural workers because they are the ones who are very efficient and they know better. So we have to incorporate them so that they, that will further strengthen the multiculturalism workforce. And also for the workforce, we have to improve their cultural competency and also provide access to interpreters. We need more interpreters, very qualified ones.

Interviewer: More interpreters and bicultural workers? So it seems like there's people there that could do that?

Respondent: Yeah, exactly. Because bicultural worker really not adequate, but…

Interviewer: Oh, but I mean they could learn to be, it seems.

Respondent: Yeah. So we can have like a few bicultural workers who is providing the service but we have got other staff. Let's say in a hospital, I mean there may be a few bicultural worker but majority are, say, from different backgrounds. So you need still interpreters to offer service.

Interviewer: Definitely. Alright, so you've actually… the final question here, you just mentioned a couple, but what, aside from say strengthening bicultural workforce, more funding for settlement services sector, what other… are there any other kinds of solutions that you'd like to see to support migrants settle well or adjust well in Australia?

Respondent: So in my knowledge, I think we have to focus a lot on to make sure they are productive and enter the workforce because it's very good for the Australian, let's say, government. Because once they are productive, I mean they may be very happy, feel happy. No mental health… less burden to the welfare. And then they not only help themselves, they can also help family and also because they have got very close relationships, social relationships, if you employ one refugee, let's say, in a factory, that can also pave the way for other people or other sort of… to come and join, which is good. So we have to focus a lot on employment. And also refugee population, they have got overseas education. So there's a barrier. So the recognition of their overseas education is one issue. So we have to have some systems to help them to top up or to upgrade that because government, I mean at the moment we don't have, I mean we want the government

to have some schemes to… let's say, if you have an overseas, let's say, a general doctor or, let's say, a vet, but can't practice here, I mean you have got already a lot of time and resources spent on that one. Here you are doing a low level job, like cleaning or factory, which is maybe not in-line with their passion and probably you will end up with a disability or you're not enjoying that but you just use that for the sake of an income.

But if the government just case by case provide a range of sort of offers to help these people to go, let's say if you are a vet, overseas vet, or overseas general doctor, so the government should help you to take some more classes or studies and then to get certified and then enter the workforce. So while we are also relying on overseas skilled migrants, so I mean we're helping them to become a sort of worker in Australia, this will also strengthen their sense of belonging. But if you're a sort of overseas refugee citizen but not productive, not working, I mean that's also, I mean then you don't feel a sense of belonging because you rely still on government, which is also for some refugees maybe they not feel comfortable if you get from, you know, tax payers. You have to be, you have to pay tax in order to get tax. So you have to get them in those things (?) once you are retired. So I suggest there should be more projects on sort of to help them to be productive. And there are barriers in terms of English, sort of education, awareness. So we have to have more projects than (?) this one.

And also the government should sit with these communities, consult with them, and also see what are their needs and in a culturally appropriate way. It's a multisectoral sort of planning. But in general, as I… I would say that refugees are a great potential (?) in this country and they contribute for the development and prosperity of the society rather than to rely on our tax or rely on government schemes. But it's mainly depends, I mean it's government's job to come up, I mean, or maybe researchers come up with good sort of remedies. Maybe we can go… one example, for example, some European or Scandinavian countries, they've got very good lessons or schemes that if you're an overseas migrant they just assist you and then they can provide you some grant or some workplace sort of education so that you get accredited and then your education is accepted and then top up with some extra training and then you end up with a sort of accredited work in that field that you had that training. So then that's more sustainable, very cost effective. And then that's also a real sense of citizenship or Australian… so you know. So we want that way in Australia as well.

So in Australia, I mean, if you have that… I mean I'm talking about some tertiary education. I mean without support of government you can't afford. That's why you go and then do a taxi or a very low-level job, which is not in-line with their passion and then you end up with maybe physically you're not fit and then you end up with some disability and then you will be a burden. So that's that one. And yeah, and then there are lots of employment opportunities. I mean we have

to train and educate.  And another job could be also we have to work with the employers.  I mean working with employers and companies and private sector is another job because they may have a misunderstanding, they may have a sort of… I mean we have to also advocate this human resource and this sort of potential to then, so look, OK, if you invest on them they can be a good profit for you.

Interviewer: Excellent.  Well, that's the end of the interview there.  So thanks very much for taking the time to participate.  We really appreciate that and your expertise and knowledge in the field as well.  Interview end at 3:30 PM.
