# Supplementary material for: The importance of information acquisition to settlement services literacy for humanitarian migrants in Australia
Source: PLoS One. 2023 Jan 6;18(1):e0280041. doi: 10.1371/journal.pone.0280041 (PMC9821785; doi:10.1371/journal.pone.0280041)
Supplement: S1 Data — (ZIP) [file pone.0280041.s003.zip › SP_11_Victoria.pdf]

Interviewer: Alright. So we are (SERVICE NAME) with (INTERVIEWER NAME), (NAME), and (NAME). So thanks again. I just wanted to clarify actually before we go through these questions that for the purposes of this research study we're referencing just newly arrived migrants, that's within the first five years, and that can be both forced and voluntary migrants as well. So when we go through these questions it's always about the services you offer for newly arrived migrants. Alright.

Respondent 1: So about services?

Interviewer: Yeah, that's right. And services and programmes that you run, you know. So the first set of questions is about services being provided by your organisation that assist newly migrated people to settle in Australia. And that could be education, health, social, legal, or any other kind of social support services. So could you tell us a little bit about the services or programmes being provided by (SERVICE NAME) for new migrants?

Respondent 2: So (NAME), will I do the… I can talk about the SETS funding that we get. So we get Settlement Services funding to run…

Respondent 1: Sure, (NAME).

Respondent 2: Yeah, to run a couple of programmes, one of which is a programme where we're meeting with anyone who's newly arrived, young people. Mostly we're working with 15 to 25 years old's but sometimes a little bit earlier, sometimes a little bit later. And we're meeting with them to really do some leadership training, so leading yourself, leading others, what does it mean to have a voice, how do you do that, how do you be brave and step up. And then through that process of those conversations we'll be making an assessment of where they're up to and actually talking with them about their journey and what they're looking for in their sort of settlement journey. We would then either funnel them off to our Employment Empowers programme, which is looking for employment pathways for young people. So it might be either teaming them up with a mentor that they'll have for 12 months, and then it will be, once they've, while they've got that mentor they'll be looking at CV, cover letter, job applications, job interviews, all of those soft skills that you need for employment. And that mentor will be talking to them about really the employment pathways in Australia because they're different from those jobs overseas. So either they'll go off into Employment Empowers or they'll go back into our leadership programme and there'll be more kind of conversation and what else do they need so that they're ready for that employment. And it might be also a conversation about education pathways as well.

So that's the kind of the SETS funding to do that. We also get some funding from the federal government to do some work with community or with organisations. So sector support services where we get those

services together to talk about work that they're doing and how they could do that better.

Interviewer: OK. So as in mainstream services?

Respondent 2: Yeah, or settlement services.

Interviewer: Or other settlement services as well. OK.

Respondent 2: Yeah. So that's the actual funding we get. But outside of that we see a lot of young people form refugee and migrant backgrounds who are newly arrived in our programmes. For example, Welcome Football, up in (NAME OF LOCATION), which is a football/soccer programme. They call it football. And basically that's working with young people. They might have only been here three or four weeks. They might have come from the English language school, we've got a lot of connections with the [indistinct 3.29] school in (NAME OF LOCATION), and they would come and play soccer after school. They get opportunities for coaching and referee training, there's opportunities for members of clubs. So they get… and through all of those sorts of things they're getting practice in English language. We also do work with newly arrived young people at the English language schools through a You Can Too Programme. So You Can Too is done in partnership with Foundation House and that is a six-week engagement programme where again we're talking with those young people about where they're up to, what they're looking for, education employment pathways, language acquisition, fun and games, recreation opportunities in Australia. That kind of thing.

So they're the kind of things I can think off the top of my head (NAME). Is there anything else from you, other services that would be…?

Respondent 1: Yeah, so our Reconnect programme works with newly arrived young people, so within the five-year time frame, although there are some, you know, exceptions to that in a case work framework. Sort of early intervention where young people are at risk of homelessness, which is a fairly broad criteria, if you like. It's actually interesting in the way that we do that I think is that we have, we deliver that programme in about several LGAs across the southeast and northwest and to [indistinct 5.08] and to be at the coalface, if you like, we have case workers placed in a couple of schools in the southeast and the northwest where there are high numbers of newly arrived refugees. And that's obviously because that's where there's identified cohort but it also offers a framework of early intervention and capacity building with the staff of those schools as well that we develop relationships and work alongside. So that's a DSS funded project and we've run that for several years.

What I often do, (INTERVIEWER NAME), is I develop a brochure that talks about programmes and services in the northwest. I'll email you that.

Interviewer: Yeah, that'd be great.

Respondent 1: So you've got a snapshot of it. And it's a snapshot, so it's not an cumbersome document, it just provides you a snapshot of what we do. Then then there are other, you know, other programmes that, you know, may or may not… look, I don't know whether you're interested in this or not but we also see a significant number of young people who've arrived on orphan visas, and because of the nature of orphan (?) visas, and because of the nature of those visas they actually don't get settlement support. So they're at huge risk. So they're omitted from that process, I just thought I'd take the opportunities to note that.

Interviewer: No, that's great to note that.

Respondent 1: Yeah. So what I'll do is I'll send you this document, which I'm about to do now. So it's (INTERVIEWER NAME), isn't it?

Interviewer: That's right. Yeah.

Respondent 1: Yeah, OK. So I'm flicking that to you now so I won't forget. There are other programmes that we manage that may also be inclusive of those young people who are newly arrived. But the contract doesn't necessarily state that they have to be within five years or seven years of arrival. So some of those other programmes are our Handshake programme, our Youth Learning Pathways programme, which are all outlined in the flyer that I will send you. I think… I might be jumping the gun because it might be included in the 10 questions, but I think what… literacy…

Interviewer: Literacy is, yes.

Respondent 1: I'm sorry?

Interviewer: Literacy is included in the 10 questions.

Respondent 1: Yes. But I think what's, what's interesting when you say that is families might be literate. I'm talking about forced marriage. So you know, families might be literate but may not necessarily act because they understand that there's conflict of interest or that there's legislation that, you know, talks to the opposite of what their wishes are. But I think there seems to be a lack of information at the point of induction for some of, for many of our families around, you know, what are the expectations? You could even broaden that out to child protection. And I don't think that's necessarily an omission, I just don't think that… there's so many broad issues to cover, I just don't know if there's the capacity to do that. Does that make sense?

Interviewer: Yeah, it does.

Respondent 1: I may have gone off topic.

Interviewer: No, no. Not at all.

Respondent 2: And lots of our programmes do see newly arrived young people even though they're not funded through any kind of particular funding. So the La Mana Pasifika Youth programme, there'll be young people coming from the Pacific region who are coming to Australia who again don't, wouldn't be counted as having those settlement services, support services, and yet, yeah, so they're struggling in school or they're struggling to find work, so our workers would work alongside them.

Interviewer: Yeah, so this is an interesting, I think it's interesting what you're saying because I found so far that most people seem to spend almost all their time with people through, you know, through their SETS funding essentially. So that's going to be people on humanitarian entrance that can access that. And it seems that there is a gap, my feeling, that there is a gap for people that aren't on the right visa that still need support in settling. So it's interesting to see that you are incorporating…

Respondent 1: And I also wonder whether we actually take, utilise, or access the information that we have. Because what we do know is that things can become unsettled or continue to be unsettled into your fifth, sixth, seventh, eighth, ninth year, yeah. By design, you know, a lot of the families and young people aren't eligible for services and there are deficiencies, if you like, in mainstream around access and equity.

Interviewer: Yeah, definitely.

Respondent 2: Yeah. At a recent SETS meeting in the southeast I was hearing a lot of services talking about, there was almost unanimity around the table of the 15/18 services that were there, saying that it's often that people come in and they were eligible but they've only been kind of been made aware that there are these supports and things at the kind of seven year mark or eight year mark. That's when they've landed enough to kind of go, oh, so there's all this stuff out here? Oh, well we need a bit of help with this. But by that time they've actually missed the five-year window. So those services then are saying, we've got to know you, we've built up a lovely rapport, and we think you're great, but we can't help you because we're not funded to. So that gap is huge.

Interviewer: Alright.

Respondent 2: Because people… yeah. People's settlement services, literacy, there's people... people's literacy, full stop, you know. And there's all those settlement issues around coming to a new country, new language. If

you've come from a country where there isn't actually a service support system, just even recognising that there is one, yeah, it's huge.

Interviewer: Definitely. And so are you aware of any services that are needed but aren't available for new migrants?

Respondent 1: Look, I think, yeah, I think if you look at intergenerational conflict and family violence I would say there's a significant gap. You know, if you broaden out, you know, there's… we did a bit of work at (SERVICE NAME) only a couple of years ago where there was, you know, a significant gap in mainstream services being inclusive of our newly arrived or diverse communities. So there was a disconnect. Does that make sense? We know that it's an issue that exists within the community but they were insufficiently represented in family services data.

Interviewer: Yeah, right. So there was, it wasn't being addressed essentially? The problem exists but it's not being acknowledged by mainstream services?

Respondent 1: Well, you know, are they culturally competent? Do they have a diverse workforce? Do they have a bicultural workforce that connect with…? Do you know, the other problem we have with our systems is that, you know, it's often the most needy that get looked over, and I'll tell you why that exists. I think it exists because it's a bottleneck, you know. Services can be at capacity without having to go out and agitate for business. So in saying that, you take those that are more receptive, who are more readily at your finger tip, for want of a better word. You know, they become your client base rather than services necessarily thinking about, well, there's a gap in our service, how do we connect? Do you see what I'm saying?

Interviewer: No, definitely. Yes.

Respondent 1: So it's, you know, the… and, which is, you know, obvious in some ways because if you're born here and you speak the language and you feel safe and comfortable using as a service user and you know how to navigate, you're going to be at the front door, you're going to be first in line. So our communities are at a distinct disadvantage.

Respondent 2: Yeah, we recently were talking about young people who have caring responsibilities, maybe their parents have mental health issues, and they're doing some of the caring and navigating the services for their parents but they're not being seen in any of the stats that are being collected because they wouldn't have that idea [indistinct 14.34] for them. Maybe that language around that idea of being a carer for someone. And yeah, they're not knocking on the doors of services saying, we need to be, we need some help here. So then there's a double layer of mental health issues maybe for them in terms of the stresses that they are facing but that's not being seen.

Interviewer: Alright. And so are you aware of any, or do you have, any services that are kind of over-utilised that there's a lot of demand for and you can't meet that demand?

Respondent 1: Pretty much all our… well, yeah. I can say from youth support side of things is that, you know, we generally don't have any issues in meeting targets at all, KPIs. You know, there is one programme, our Youth Learning Pathways programme, which, you know, there's a bit of a lack of take-up, I mean we're getting there with that. But I wonder whether that's because, you know, the sector's so chaotic and divided in some ways that that prevents successful referral pathways and streamlined pathways or whether there are so many services in the system doing similar things that it's hard for young people to navigate and understand.

Respondent 2: Or there are so many programmes that get like eight months funding or 12 months funding and they come and go because there's the time to build the networks, the relationships that support really good referral pathways for between workers, much less between the young people and the workers.

Respondent 1: Yeah. I mean the other thing, for example, in the Youth Learning Pathways, which is inclusive of kids across the board, newly arrived and post that five or six years, is that, you know, we have a Western lens over our programme development often. You know, and I mean this is a Department of Education funded programme and it's a great programme and it will gather momentum and it is developing a profile and a brand and a reputation. But, you know, in terms of what we require of the kids to participate, did we give it enough thought? You know, was there codesign with young people? Are we expecting people to be too many steps up the path rather than… do we need to shave it back so that they're, you know, it's more of a step one and two programme than, you know, kids being ready for a certificate or a pre-apprenticeship course?

Respondent 2: And then English language would be another thing that's in high demand. I've heard lots of stories up in the north, for example, in Hume, the Hume region, young people just going straight into school and skipping the English language classes because they're too full, they've got no space for them. Yeah.

Respondent 1: Look, something else we've seen really is extremely common, particularly with our South Sudanese community, is navigating, you know, systems like MyGov, you know, utility bills, financial literacy. There is just not enough of that around to support these and educate and support these families. There is just not enough.

Respondent 2: Yeah, I can't even navigate MyGov much less any of the young people who are speaking maybe four or five other languages and have only acquired English recently and yeah.

Respondent 1: Yeah, yeah.

Interviewer: Yeah.

Respondent 1: But so many of our families find themselves in absolute dire financial straits, situations, and we have not as a service system adapted to meet those needs. You know, even in the design of our workforce. You know, are we creating enough? Do we have a, you know, I think we do it at (SERVICE NAME), but do we have a commitment to employ young people who are, who can be mentors and role models because, you know, they can speak to experiences in a very real way? And are our diverse… are our workforce is diverse enough? And I'm talking more about mainstream.

Interviewer: Totally. And so can you tell us about any methods that you use to measure the effectiveness of the services that you provide?

Respondent 1: Well, I mean we… all our programmes have contracts. So, you know, we're required to meet a certain number of targets. Often there's quantitative as well as qualitative evaluation. Yeah, look, it depends on which programme, (INTERVIEWER NAME), and, you know, we often… Youth Leadership, for example, you know, always have client feedback. We often do a new support. So we try and measure and get feedback from young people. But we also, yeah, have a commitment to consulting young people. (SERVICE NAME) has a, has youth advisory groups across regions and (NAME OF LOCATION). So young people are very much… and part of our strat plan is my voice, so hearing the voice of young people, understanding their journey. And do you think anything… what have I missed out, (NAME)?

Respondent 2: Yeah, no, I think, yeah, going back to the strategic plan, which we can give you a copy of if you, I think there's one out there, is, yeah, really a strong kind of centring of young people. So we have a youth advisory group here, one in (NAME OF LOCATION), and one in Ballarat, and we're regularly consulting them about a whole range of issues to get their opinions and views. Yeah.

Interviewer: Excellent. And you mentioned Foundation House before, you do some work with them. What are some of the other organisations or even departments or councils that you collaborate with?

Respondent 2: Well, it would be any of the local government that we're in. We're a state-wide service, so in all the LGAs that we work in. Brotherhood of St Laurence would be another partner in a project we've got. There's a whole range of organisations we do kind of training and support for

just across the board, all sorts of organisations. (NAME), have you got any other particular ones that we're doing…?

Respondent 1: Actually, if you, (NAME), I don't know whether you have it at your finger tips, but in our, I don't know if it was in our last but it was in a previous annual report, a list of all our partners. And it went through in brackets like from local government to state government, federal, community, education. Maybe it might be best to show (INTERVIEWER NAME) that. Do you know…?

Respondent 2: Yeah, I'll find that.

Interviewer: That's good. I think there was one just on the wall.

Respondent 1: It will be on our website anyway, (INTERVIEWER NAME).

Respondent 2: Yeah, I'll get that…

Respondent 1: But look, I mean, I could… to answer that question, (INTERVIEWER NAME), could… like we operate at all levels of government and partner with. We're funded through state, sometimes local government, federal, sometimes philanthropies. We work with community education centres, we work with language schools, we work with police for our YRIPP programme, it's endless.

Respondent 2: Schools. Every school that any young people are in we're working with. We've got an education programme that works with clusters of schools, five clusters of schools each year across Melbourne where there's high growth for young people from refugee backgrounds, and that's also in partnership with Foundation House.

Interviewer: Excellent. Alright, so the following questions are related to how migrants adjust to Australian culture and society and the kinds of issues and challenges migrants might face. So can you tell us about your understanding of how migrants that you work with understand culture, Australian culture, and society?

Respondent 2: (NAME)?

Respondent 1: Can you say that again, (INTERVIEWER NAME)? Sorry.

Interviewer: Yeah. Could you just tell us a little bit of your understanding about how migrants that you work with understand Australian culture and society?

Respondent 1: How they work to understand it?

Interviewer: Or how they do, what their level of understanding of Australian society and culture is.

Respondent 1: Oh, look, I think it often happens through their children, which is, you know, fraught potentially. I mean I think for that question, you know, it's better off directed towards someone who's had those experience, I suppose, because they can, you know, they can talk to it. But… so the question is… sorry, (INTERVIEWER NAME), can you say it again?

Interviewer: Yeah. Can you tell us about your understanding of how migrants you work with understand Australian culture and society?

Respondent 1: Yeah. Well, look, I think it varies. I think it varies depending on networks that exist here within their own communities. So, you know, do they have networks that can help the transition period?

Respondent 2: How long have the more original people been there and how well networked are they into the community? So it might be different for the South Sudanese as opposed to the Karen. Yeah, that depends.

Respondent 1: Yeah. You know, what's the… you know, what have been the premigration and the journey experiences there. You know, people may have experienced a whole lot of grief and loss and trauma and, you know, be suffering with a range of issues or, you know, mental health. So are we adequately assessing to ensure that they get the services they need to settle or do we expect that they will just access at some point? What we do know is that, for example, kids in the Refugee Minor Programme, there was some research done by (SERVICE NAME) several years ago and, you know, one of the recommendations was that before kids exit that Refugee Minor Programme, they be introduced into services because at the point of exit they're generally OK. It's six months later that things break down and do they know how to re-access or re-enter the system or are they familiar and comfortable in asking for that help? So it was about orientating kids.

Respondent 2: And there's definitely that, there's some graphs around that talk about that they, prearrival, hopes and dreams are high, they arrive here and it's like, wow, this is amazing, and then there's a dip, and that dip goes down as the reality hits and they realise it's hard to get a job, hard to get into education. Some of the hopes and dreams they had may not be realised and it's whether they can kind of pull themselves out of that through being connected to services or whether they continue on down.

Interviewer: Yeah, right. And so because there's the International Organisation of Migration does orientation courses, is that right, for Australia pre-departing?

Respondent 1: I suppose, yeah.

Interviewer: And then there's also obviously the HSP. So is there something, I mean, because that's the first 12 months. So is there something kind of missing, I guess? If you're saying that… or is just… are the services

OK but it's just the reality hits of the difficulty in migrating that becomes the…?

Respondent 1: Look, people, there's stuff that, is it ASCO, do overseas before people migrate is people don't often know until the very last minute that they're coming. And from previous conversations, they may spend the entire period allocated to teaching someone how to put a nappy on a kid on a plane. So my understanding is it's a very broad brush.

Interviewer: Yeah. Definitely.

Respondent 1: Yeah.

Interviewer: Alright, and so do you see that new migrants…

Respondent 1: And, sorry (INTERVIEWER NAME), it can also depend on, you know, language, English acquisition. If you speak English prior to arrival, you're probably OK. If you've got networks, you're probably OK. But if you're, if you're way behind in terms of your social capital and employment in something that doesn't happen for 20 minutes, for 20, you know, 15 years, you're at a disadvantage. In the youth sector we often talk about young people having issues around social capital and access equal opportunities in employment and, you know, not being at a disadvantage but we miss the parents because the parents are often in the same boat.

Respondent 2: (NAME), I'm aware it's 4:04, do you need to head off?

Respondent 1: Yeah.

Respondent 2: Yeah.

Respondent 1: Is that OK? I mean is there anything else of your questions, (INTERVIEWER NAME), that you think I might…?

Interviewer: I mean I'm not sure. And there are more questions but we'll just have to finish it there.

Respondent 2: Well, I'll stay here and see what I can do. Yeah. Thanks (INTERVIEWER NAME).

Interviewer: Thanks a lot (NAME).

Respondent 1: OK. And (INTERVIEWER NAME) , if there's anything you think I can add in terms of my particular area just let me know and I can flick an email response off to (NAME).

Respondent 2: Yeah, thanks (INTERVIEWER NAME).

Respondent 1: Thank you.

Respondent 2: Cheers, bye.

Interviewer: Thanks, bye.

Respondent 1: Cheers.

Interviewer: Alright. So do you, so (NAME), do you see that migrants have the opportunity to practice their own culture once they're in Australia?

Respondent 2: I know that in the government policy says it's about integration and kind of moving in. I would say that there's more room, there should be more room to move. And I would say that they don't necessarily feel that, you need to ask them obviously. But my sense would be, from what I've heard, there are often lots of restrictions, they feel. There's often some real fear about expressing their own culture, yeah. That there's some danger in doing that, community kind of slamming if they do that. You know, just look what happened in Bendigo around building a Mosque in Bendigo a few years ago. I reckon that's a kind of day to day experience of lots of young people, whether it's how they dress, how they wear their hair, where they're going, recent conversations with libraries saying, with librarians talking about two or more young people together in a group is a gang, and librarians really fearing…

Interviewer: In libraries?

Respondent 2: Yeah, in libraries. You know, access to Wi-Fi, not having to have sibling responsibilities, a public space that's safely kind of being navigated by adults that's not a shopping centre where you're going to get followed around by security. So libraries are actually a really important part of young people who are newly arrived to kind of sort of community possibilities. But yeah, if the library's not receptive to that then that becomes really tricky.

Interviewer: Alright. So these questions relate to migrant's sense of belonging and inclusion in Australian society. So can you tell us about any programmes that (SERVICE NAME) offers that supports migrant's or helps to enhance migrant's sense of belonging and cultural inclusion?

Respondent 2: I would hope that all our programmes did, just generally and broadly. But probably I'm thinking that the Youth Leadership programme that we run, there's a whole range of programmes that run through there, sports engagement, recreation engagement. So there's a young women's sports hub down in (NAME OF LOCATION) and that's for young women who are wanting to maybe try out some sports. They're playing volleyball and badminton down there at the moment. And it's a safe space for young women to go. There are some issues in terms of gender around young women feeling safe or the parents feeling safe that their daughters can go to those spaces, so that's been a really

successful one. I reckon lots of the sports stuff down in Morwell has been really about, you know, bringing people our of their homes and into the public atmosphere to play together. And in Ballarat they run a street party once a year called Rock The Block, which is about getting young people out and on the street and kind of really celebrating young people's culture from wherever young people are from. There's a whole range of programmes we've got that do that sort of work.

Interviewer: Excellent. And what, I guess, what are some of the key people that your clients contact for social and emotional support? So it's obviously linked to kind of social belonging, whether they're insular or whether they're starting to spread out.

Respondent 2: Yeah. Well, I think one of their biggest supports is each other. It's a huge [indistinct 32.27] and it works. And then, you know, there are workers throughout (SERVICE NAME) and all the different programmes we've got. But yeah, I think the biggest one is each other. The… it's interesting, I reckon, the experience of migration now as opposed to maybe even eight years ago or 10 years ago in terms of social media and what a difference that's made for young people and their experience of settlement and isolation or connection. So a lot of the youth leadership programmes we're doing now have a really strong social media component about really kind of highlighting those voices in those sort of arenas as well. We were funded by the Victorian Electoral Commission to do a programme on young people and democracy, sort of looking at what are the informal as well as the formal ways that you can have your voice heard in Australia or in Victoria, it was the Victorian Electoral Commission. We had a whole series of workshops, 40/50 young people at a time culminating in a small group that came together to do a really intensive… so there's a one day workshops and intensive three day workshop and then some young people were selected to run some social media campaigns around, you know, be seen, be heard, have your voice heard in terms of advocacy for your community and advocacy for yourself. Yeah, so strong advocacy focus in the work that we're doing.

Interviewer: Great. So the next question's about programmes that are responsive to wellbeing and health of migrants. So do you have any programmes that are currently implemented to respond to health and wellbeing?

Respondent 2: We've just launched a programme recently that's come out of conversations with the youth advisory group here in (NAME OF LOCATION) where they were talking about those carer responsibilities in terms of the mental health of their parents and also their own experience of mental health kind of buckling under the weight of that and needing some support themselves. And it's a programme that's going to run for two years that's around talking about mental health in schools. So young people with an experience of mental health issues, going into schools and talking with the young people about, you know, it's OK to get help, it's OK to say, "I need help," there is help out

there. And that help might be in the form of some social stuff, you know, burn off some stress by playing a basketball game or it might be actually need a kind of trained professional who can give a hand with this. So that would be one of them but it's a common subject that's talked about around, you know, mental/emotional health. And also health and wellbeing in terms of sport. You know, sport is a big tool here that's used for engagement of young people, to have those conversations about what else do you need, what more do you need. So yeah.

Interviewer: Excellent. And so do you see any enablers or barriers that your clients have experienced in accessing programmes around health and wellbeing?

Respondent 2: There are, there are some trickiness's with navigating the service system more generally, it's complex. There are some… so there's the service system…

Interviewer: So the universal services, health services?

Respondent 2: Yeah. Who are generally not particularly working hard to get these young people in even though we know that 48% of young people in Victoria have one or both parents born overseas. So that's a huge percentage, that's now, you know, half the population we're talking about. So there's the mainstream or the general service system, there are specialist services who I think, you know, do struggle with some of this stuff as well in terms of employing people who are going to be friendly and welcoming and who are going to, those young people are going to be able to connect with. Because they can see that person that looks like me, they'll know what I'm feeling. And then there was another part to your question, I completely forgot it.

Interviewer: Just about if there's any enablers. That's a barrier, is there anything that…?

Respondent 2: Yeah, barriers and enablers. Yeah. So yeah, well the enablers are going to be a more diverse workforce, yeah. And also consultation with young people, bringing young people in to talk to them about what might stop them from being in there. Like it's a whole piece I reckon around engagement. How do you engage with those young people to help to get them to help you have an easier navigation path through into your service? So the willingness of services to do that I reckon is, you know, there's still some stuff to be done there. The Multicultural Youth Advocacy Network, MYAN, who's the national body of… (SERVICE NAME) the state body and MYANs the national body. Do you know… do you know of MYAN?

Interviewer: Yeah, yeah.

Respondent 2: Yeah, yeah. So have you seen their "it's not just ticking a box" report, which is around youth participation?

Interviewer: No, I haven't seen it.

Respondent 2: Yeah, that's on their front website, that's worth having a look at because that's a whole range of things for services to be thinking about and doing in terms of youth voice, youth participation, codesign, that kind of thing. It's not just ticking a box. And then the other one is the National Youth Settlement Framework. That is, that's a framework that we do training in for anyone looking to work with young people from refugee and migrant backgrounds. National Youth Settlement Framework has a whole stack of good practice guidelines and a, basically a framework for what does it take to settle well into Australia. And it's kind of really a frame that organisations can use to put over their work to see if they are kind of ticking all the boxes in terms of making it sort of easy and welcoming for young people to settle well.

Interviewer: Excellent. The next questions are about any programmes that you have that works to enhancing new migrant's financial literacy, income generation, or managing money?

Respondent 2: Yeah, financial literacy. Apart from the fact that it's, you know, the conversation that's being and all the time, not a specific programme, kind of economic wellbeing and employment-y kind of stuff, we've got our Employment Empowers programme, which is that work with someone for a year, you get a mentor for a year, you get placed into a job and you've got that support going… often job placement programmes, once you've got the job you're out, you're on your own. Whereas this is a mentor for a year. We also have another programme, which is lying a little bit fallow at the moment, we're just about to have a conversation tomorrow about it, which is called, let me just think. It's called… not connecting communities. It's a start-up programme for kind of businesses. So it's how do you start up a business in Australia? Often the business start-up programmes for sort of entrepreneurs and that kind of thing are six weeks and it's, what's an ABN, how do you do a business report, how do you do a business plan, how do you get your budget sorted, righto, you're off on your own. This is a 14-week programme that's called Enterprising Communities. That's really creating social cohesion with that group. We get mentors from the community who are already running businesses to mentor these new business owners. And there's supports about how to run a business. But they're also making networks and meeting new people all the time. So that's another kind of specific one around income generation.

Interviewer: OK. So what are the key financial challenges you see clients facing when adjusting to life in Australia?

Respondent 2: Well, as (NAME) was saying, there's some people in some fairly dire straits in terms of financial commitments. You know, for young people I think there's often a pressure to send money home as well as generate their own, you know, income to be able to live because, yeah, it looks easy and there's, you know, fresh food and fresh air and clean water and housing, that kind of thing. But that, you know, that all obviously costs so that makes it very tricky. I think, yeah, I think there's lots of, more possibilities that could be made available to young people because I see them, yeah, all the conversations are around, how do I get a job? We know that for young people who are Anglo and born in Australia it takes like three years for them when they leave university to get their first job in their chosen profession. It takes more than seven years for a young person from a refugee/migrant background to get their first job in their chosen profession after leaving university. And that's, you know, there's some really clear evidence around that stuff, around young people changing their names to something like Smith or Jones and suddenly they're in the "in" pile in the way that they weren't. So some of those practices around, in terms of organisations doing blind CVs and taking names and ages and gender off CVs in order to assess…

Interviewer: So organisations do do that?

Respondent 2: They should be doing that.

Interviewer: They don't?

Respondent 2: There are some that it's considered best practice. And certainly, you know, there was a government kind of, Victorian government, "how you should be doing employing people" kind of booklet that came out that was suggesting that there are possibilities there. And, you know, there was a horrible tiny percentage of women in orchestras around the world until they started doing blind auditions where they'd put up a screen, get everyone to take their shoes off, and they'd walk across, they'd start playing. It went from virtually no percentage of women in orchestras to 40% almost overnight when they started doing blind auditions. Because, you know, you could hear the click of the heels across the, if they were wearing high heels, or you could see them if they were, you know, male or female, put a screen up and have no shoes, didn't know who it was, we're just going on the musicianship and suddenly there's all these women in orchestras.

Interviewer: Right. So obviously this seems to be an issue where people have to interview but certainly… I hadn't heard of this. I mean that seems entirely… what a good thing to do, I think. Definitely.

Respondent 2: Yeah, yeah, yeah. And a really clear way where you could make, you could at least be checking yourself to make sure that you are, you know, you've got these ideals about having a diverse workforce but what are those unconscious biases that just have you defaulting to

someone who's familiar? Someone who looks like me or sounds like me or… yeah.

Interviewer: Which could be hard because you could almost never get rid of that at smaller organisations. Obviously, an organisation that has an HR team, you know, there's a person inboxing to people then there's some step where, you know, you could remove names, you could remove… but before that you're always going to have to have that contact, that name, whatever.

Respondent 2: Yeah, yeah. Absolutely.

Interviewer: Yeah. Interesting. And so you mentioned sending money home. Are there any other kind of culturally specific dynamics that make managing money difficult or a challenge?

Respondent 2: Well, I think financial literacy is an issue for everyone. I think it's, you know, understanding where your money's going, how it's going, how fast it's going, how much you've got, what are the limits, you know, budgeting yourself. They're all skills that I reckon take years and years to acquire, they're not something that you just have instantly. So yeah, there's probably lots of sort of debt that young people are accruing that we don't even know about, we won't know for several years, and then we'll find it out.

Interviewer: Alright. So the next questions are about programmes available to support your clients when they face legal challenges. So do you offer any programmes and support in responding to legal issues?

Respondent 2: Yeah, we have our YRIPP programme, that's the…

Interviewer: What is that, sorry?

Respondent 2: YRIPP. YRIPP. YRIPP. And now you're going to make me say what it is. It's a youth…

Interviewer: That's OK. You don't have to say what it is. It's a programme, it's called the letter Y, RIPP.

Respondent 2: It's so irritating that I can't remember it though.

Interviewer: And what does it do?

Respondent 2: And basically what it does is we have a workforce of something like 250 volunteers across Victoria who we train up and keep trained so that if a young person gets called to a police station and they don't have a guardian or a parent who can come and be with them, that volunteer would be called in so that they can sit with them and ensure that the police processes are going well and that the young people understand their rights fully so that that young person's not alone. So the last year,

it was over 800 volunteer hours were put into being with young people in police stations basically. Did I say 800 or 8,000?

Interviewer: 800.

Respondent 2: 8,000.

Interviewer: 8,000?

Respondent 2: Over 8,000. It was just over 8,000 volunteer hours were, you know, were volunteers sitting with young people in police stations supporting them through that pretty scary process. So… and that then is a whole range of work we're doing in terms of advocacy with police and any other of those assorted kind of areas of the law. So that's really at the pointy end of the law. We would be referring people to, you know, local legal aid services. Yeah.

Interviewer: Of course. And so in your opinion, what's the level of awareness of new migrants in accessing legal services? So obviously you have volunteers to support them when they're faced with that but what about other kind of legal services?

Respondent 2: Yeah, see again, I think that that's an issue for everyone. I don't think… so for someone who's newly arrived and maybe doesn't have great English skills or doesn't understand the system or has a significant fear of the system because it's in their own country it's been a fairly unequal kind of or abuse kind of system. Yeah, I reckon there are huge barriers in the way of young people being able to understand all of that and make that all, make sense of all that.

Interviewer: Alright. So the next questions relate to movement of your clients from one place to another. So what are the key reasons that you see clients moving from one place to another or from one suburb to another suburb?

Respondent 2: Yeah, that's going to be an interesting thing to be thinking about over the next few years as the government policy around moving newly arrived people out to country areas becomes much more of a player. Those country areas aren't necessarily set up, in fact they're often completely anti the idea. So just how those young people are able to integrate, so… and it's interesting, we don't have really a mechanism for tracing secondary migration or tertiary migration once people arrive in Australia. But what I hear is people talking about employment and social sort of stuff really, they're two really big ones.

Interviewer: Social as in the community?

Respondent 2: As in the community, yeah, yeah. Where can I be where there's more of the community who looks like me?

Interviewer: Do you see any trends?

Respondent 2: Well, I see, it's kind of that interesting thing. I know there's a really big Karen community in Bendigo, in Wyndham, and in Nhill and that's based around employment. But obviously, you know, those people are talking to others and saying, "hey, come here, there's employment here." So there's bigger communities of those particular groups there. I did hear of a lovely project that was talking with some people of African background who were living in the flats in (NAME OF LOCATION), just around the corner here. And those people had been settled there when they first arrived in Australia, they were under the mistaken belief that they had to stay there, they were kind of stuck there. In fact, they're all farmers and actually wanted to farm. And so there was an organisation I was talking with who were having some conversations with, out Horsham way, there were farms who were experiencing that kind of drift of young people into the city and not coming back to the farm. And the farmers need help. So there was a conversation around there's empty houses, there's farmers that need work, here are some people who want to be there, don't want to be in the city. They didn't want to live in the flats. So there was a kind of how do we make it, a pathway for them to go and live somewhere that kind of fits much more with how they want to live. So yeah, that seemed like a really lovely example of, let's get, let's get a bit more tailored in why we're sending people wherever we're sending them and how do we, how do we make it possible for people to kind of feel settled and happy.

Interviewer: Yeah. Alright, so the next question is about migrant access to education and literacy, this time (?) language programmes. So what services do you offer which respond to literacy and education? Yeah.

Respondent 2: So we've got our RESP programme, the Refugee Education Support Programme. It's funded by the Department of Education in Victoria. And that is a programme that works with clusters of schools, so there's five clusters of schools, between five and 10 schools in each cluster, across Victoria where there are high numbers of newly arrived young people attending the school. And this is really some work around professional learning for the teachers but also support for family engagement and making sure that parents and other family members are engaged with the school. And we do that by brokering partnerships with community organisations who are based in the area and who have got those ties with the community. So we also have our Learning Beyond the Bell programme, which is really a programme that supports homework clubs. So homework clubs after school and on the weekend, run in community centres, libraries, schools, staffed mostly by volunteers, often they've got a paid coordinator for a few hours a week. And we provide professional learning and support for the coordinators and also for tutors around, you know, how do you have fun and games with maths and how do you run a homework club so

that it's following all the child safety regulations that needs to be followed, that kind of thing.

So then we also, we partner with an organisation called Australian Communities Foundation to provide money for homework clubs to get some funds. So we've just released, ACF has just released $304,000 worth of funding out to 24 homework clubs so that they can have some money to start up, get some resources, get some books and pens and that kind of thing and, you know, get a paid coordinator for a little bit. So we've got those that are very education specific. But a lot of our programmes are working in schools doing youth-led programmes. There's one called Aspire to Lead that's run through Youth Leadership that's around youth voice and getting young people designing programmes that can support each other to lead well. There's a, there's our… there's another couple of programmes that are also working in schools identifying young people and kind of talking with them about, you know, leadership and leading themselves, how do they manage themselves in conflict, what are the kind of issues they're facing, how do they make a project out of that to educate the whole school about it?

Our Le Mana Pasifika Programme and also the Community Strengthening Programme, which is a South Sudanese programme. Both of those programmes are working often in schools with the young people directly or with the teachers as cultural consultants around how can they work with that community better or even as a bridge for leaders from those communities to be in the schools of the schools to be talking to the leaders. I think that's lots of the school stuff we're doing. There's probably others. But yeah, that's lots… You Can Too would be in the English language schools, talking about them before. That's a six to eight-week engagement programme with, in partnership with Foundation House. Yeah.

Interviewer: And do you see any key issues or barriers for your clients in accessing school or university education?

Respondent 2: Oh wow. There's a couple of papers our policy people have written on that sort of stuff which would be good, I can send you. One of the biggest ones would be, I mean if you think about where do young people find out about education pathways and what's possible in education. Where do Australian-born young people from an Anglo background find out about careers and what's possible in school and university, that kind of thing? They mostly find out from their parents and family. If you don't have that kind of cultural capital, if you don't have parents and family who understand that system, where do you go, who do you talk to? And, you know, we've certainly heard, I've heard lots of stories of careers teachers saying to young people from refugee and migrant backgrounds, oh VCAL, just shoot them into there instead of actually having a conversation about what's your pathway, what's the pathway you would like and how do we build a bridge for you to get there?

And then in terms of tertiary education, you've got young people from Pasifika background who have to pay international student fees if they want to go to university. They're not treated, we don't have reciprocal rights with New Zealand anymore so the Howard government took that away, so yeah, now they have to pay international student fees, which are, you know, huge amounts of money, huge amounts of money and impossible kind of debts for them to incur. So yeah. But I think it's just a lack of knowledge about what's out there. Certainly some of the young people talk about the kind of pressure it is from the family on them to be doctor, lawyer, maybe a dentist, but really doctor or lawyer. And whether they've got the interest in that or whether they've got the skill or aptitude in that is a whole other question. And if it's not that then what is it? To have the conversations and take the time for parents to understand that, you know, becoming a plumber could actually be more financially lucrative than being a doctor or a layer and might be a little bit less painful. And they might enjoy it more, there might be more aptitude there. But just how do we broaden up everyone's idea of what's possible? The young people as well as the parents and families, but also the system, so the system's not categorising these young people before they even get in the door. So there's a… yeah, on all of those levels we need work, I think.

Interviewer: Excellent. And what about employment opportunities? So you obviously have the Employment Pathways Programme, but do you see that there are employment opportunities out there for the people, for new migrants you work with?

Respondent 2: No. What I hear is it's really hard. Those young people, we've got one young guy… so we've got a programme called Shout Out, which is a speaker's bureau. We train up and support young people to do public speaking. So they go to a meeting and talk about their life experience or they might facilitate a conference, fabulous young people. Shout Out was talking about, at an employment programme we ran recently, talking about what job opportunities there were. He was talking about having applied to 40/50 jobs and not even getting a reply to say, "thanks for your application but no." Just dead silence and looking around at all his friends and going, "how come they're all getting jobs and I'm not?" And, you know, it was clear that they were from Anglo-Australian born backgrounds and didn't have names that said Shadub (?), Sadiq. So, you know, that really, that was a kind of a lesson for him in the way the system works.

So it was interesting, he talked about starting up an internet business where he was importing things from China and on-selling them. And that worked for a while but then stopped. But then he found a superior product from America, so he was importing that and then selling that and then that stopped. And then somehow in a programme he got given a mentor and the mentor was talking with him and asking him about himself, and the mentor said, "so you haven't got anything on

your CV about languages. What do you… do you speak any of the languages?" And yeah, he speaks five other languages other than English but he hadn't thought to put that down. He wants a career in banking but hadn't thought to put that down, didn't think that was a skill. And then the person said, "OK, there's a bit of a gap between when you've left school and when I've met you, like what was going on there?" And he said, "well, I had these two internet businesses but in the end they failed so I didn't want to put them down because that would look dodgy." And the mentor's going, well, but no it shows entrepreneurship and ingenuity and, you know, self-sustaining, and all these skills, and internet skills. Like all these things it shows, but his misunderstanding was that it would look badly, would reflect badly on him because they'd failed rather than talking up the fact that he did it in the first place.

So there's some kind of cultural misunderstandings. I remember talking with a group of young people a little while ago, mostly Afghani young men, and they were talking about the real lightbulb moment for them was when they learnt the idea that it was good to ask questions because in their culture it would be bad to ask questions because you don't want to show that you know nothing, you don't want to ask a question that's awkward that the boss doesn't know because you'll put shame on the boss. So what you do is you keep your questions to yourself. And so it was a lighting bolt from the sky for them to realise, no, no, in Australia we want you to ask questions because that will keep you safe and because then you'll get to know more and the boss will be able to work out what you do and don't know and be able to give you more skills and that that's seen as showing that you're keen. Whereas staying silent was the better thing to do in their culture. So like absolute opposite in terms of cultural stuff. And like where is that in any employment manual? Like it just isn't. You only find it out accidentally, often by doing the wrong thing a lot until you get in trouble. So yeah, just there's so many sort of gaps and misunderstandings I reckon around employment. It's really tricky.

Interviewer: Yes. Massive challenges, which leads to the next question. Overall, what do you think the key challenges migrants that you work with face while adjusting to Australian culture and settling in Australia?

Respondent 2: So I think it's navigating a system that's unfamiliar. And to put it in really two things, navigating a system that's like incredibly unfamiliar and often confusing and often even the workers in it don't understand the system or don't know enough about it or don't have all the networks to all the new programmes. As we said before, if the programme's only funded for 12 months, by the time you find out about it it's finished. And then… so I think it's that navigation and then I think there's that stuff around social capital. You know, how do you get referees for a job? In Australia you need to have two referees for a job, most jobs, how do you get those jobs if you don't know anyone, if the only people you're hanging out with are the people in

your community? Yeah, I always think of LTP programme as a really good one. Do you know the L to P programme in local councils?

Interviewer: No. Oh, the driving L to P? Yeah.

Respondent 2: Driving, yeah. Getting your 120… you're trapped in a car for 120 hours with someone who you've never met before who over the course of that 120 hours finds out that you're a keen learner, you've got a good sense of humour, you want to succeed, you're punctual, all of the qualities that it takes to make a good worker. And then they refer you to their friend who's got a business or they'll be a referee for you who's someone outside… you know, those bridging networks are so important in terms of social capital. It's all very well to have bonding networks but the bridging networks will get you ahead. So how do you get those bridging networks if you don't have any way to get into Australian society? Yeah.

There's a lovely piece of research done… a researcher was following around these three guys in a town outside of London in England and was looking at networks and conversations with them about how they were settling in. And they said the highlight of their week was on a Tuesday night there was a scratch soccer/football match up at the local pitch. There was a guy who had a car boot full of vests and boots and balls and nets and things and he'd just turn up, whoever turned up got a game. And they would go there every Tuesday night and from that during the course of the week they'd have people saying hello to them in the street who ordinarily they haven't met. They learnt a lot of language, maybe a lot more swearing than they needed to learn but a lot of language, colloquial language rather than the more formal language they might have studied. And yeah, they got, you know, hellos and cups of teas and job offers and all sorts of things just out of that one sort of piece of engagement. So how do you, how do you build that social capital, those networks? They would be the two things, I think.

Interviewer: Excellent. That football sounds a lot of fun. And it seems like there's a lot of people on, doing a lot of sports things because they see obviously it has value in it. Yeah.

Respondent 2: Yeah. Well, you know, you can turn up and not speak a word of the language and still have a good game of football. You know, and over the course of that you get to literally bump into people but you get to, you know, have a sense of a team spirit. Like it can also, there can also be a sense of freedom for those young people. For the rest of their time they're working hard to understand what's going on, learn the language, try and find out how to make their lives work in Australia. That 20 minutes they're playing football, you know, hour they're playing football could be the freest and happiest time because they can be carefree and they know the rules and they've got it. Yeah. So it's, sport is such an important tool to offer that, just even that little moment

of a breather. But it has all these other spin offs as well. You know, particularly when we look at sport as a pathway into employment. We've run a programme called Game Changers here, which was a young women's sport leadership programme. So it was how can you… so it was about playing sport, for young women playing sport, but they were also thinking about how can they lead their sporting clubs in some better ways to work with young people from refugee and migrant backgrounds. So they went back into their clubs and did some projects in their clubs but they also met, you know, women who were in sports career, who had sports administrative careers or… and kind of learnt that there's this whole kind of mechanism where there's jobs, paid jobs in the background of sport and kind of widen their idea about what was possible.

Interviewer: Yeah, great. And so finally, what would you like to see as possible solutions to helping migrants to adjust well to life in Australia?

Respondent 2: Possible solutions? It's interesting. This new lot of SETS funding…

Interviewer: Which is just the beginning of this year?

Respondent 2: Yeah, yeah, yeah. Has taken a much more coordinated approach than other SETS funding in other years, in other sort of settlement funding in other years. So, you know, there's, there are network meetings happening that just were really hard to pull together previously. So it feels like there's the possibility of that, but if the service system could be more coordinated and more, yeah, coordinated with each other and themselves, that would be really helpful. So there's that stuff around how do we get those services to connect with community members. So it's not just young people who we're talking to, we're talking to the whole of the community. Because while those young people might be excited to be a part of that service or a part of that programme or go into that bit of education or that bit of university or that bit of employment, sometimes the blockers are the elders in the community who maybe don't see or don't understand. So how do we, how do we engage the whole community in a kind of process of, you know, listening and learning and understanding?

Yeah, they'd be two big things. It would be about the engagement of the whole community and yeah, better coordination. It's been interesting, we've done some work recently around mental health for Orygen Youth Mental Health and we've been looking particularly at mental health services for young people from refugee/migrant backgrounds. And, you know, a lot of the services, headspace services, just really are not servicing that cohort at all. And so the conversation has been, how do we… and it's been at a whole bunch of levels, how do you get those services to work better for these young people? And some of it's around engaging the young people, some of it's around engaging the rest of the community so the elders who might have blocked will be opening up. How do you educate the workers so that

they're working, they've got better skills to work with whoever, whichever young person walks in the door rather than being nervous about some community groups? How do you… so it's at a whole range of levels, how do we make all of that happen?

How do we change the system so the young people aren't constantly having to shove themselves into the wrong fitting hole? You know, how do we change the system rather than changing the young people? Yeah. And I suppose… oh, can I also just say the mental model that's around that this is some kind of specialist, tiny group would be the other thing. This is not a specialist, tiny group, this is now 48% of the population of young people in Victoria. It's no longer a niche kind of, you know, one-off… if people's stats in their services aren't reflecting that there's 48% of the young people walking through their door from refugee or migrant backgrounds then they're missing the population they should be servicing. So for me, there's something around the mental model that's around saying that this is a niche or a specialist area. This is now generalist work.

Interviewer: Yeah. OK. Thanks very much, thanks (NAME). Yeah, really appreciate, again, your participation.

Respondent 2: No worries.

Interviewer: I think… you said I think a lot of different things than some, than what I've heard from some of the other service providers, which is interesting. Just some of the programmes, like the leadership programme, seems a little bit more… I guess the way… maybe it's because you work with young people specifically. Also working with schools, I'm not sure anyone else has really mentioned that.

Respondent 2: Oh wow, OK. Yeah.

Interviewer: Yeah. So yeah, interesting. Interview end at, I don't know what time it is, 4:46.
