# Supplementary material for: The importance of information acquisition to settlement services literacy for humanitarian migrants in Australia
Source: PLoS One. 2023 Jan 6;18(1):e0280041. doi: 10.1371/journal.pone.0280041 (PMC9821785; doi:10.1371/journal.pone.0280041)
Supplement: S1 Data — (ZIP) [file pone.0280041.s003.zip › SP_12_Victoria.pdf]

Interviewer: Anyway, that's on. So this is (SERVICE NAME) with (NAME) interviewer and (NAME). So first set of questions are about the services that (SERVICE NAME) provide to assist newly migrated people to settle into Australia. So that could be education, health, social, legal, any other kind of support. Can you tell us a bit about what your organisation provides?

Respondent: OK. I'll give you a quick summary, a brief summary about (SERVICE NAME). (SERVICE NAME) [redacted] (SERVCIE NAME) is government by a board of [indistinct 1.35] people, people that either with lived experience themselves are migrant or refugees or been of a parents, migrant parents, that they are well-established and really keen to contribute back to the community and support the community to really reach their full potential because they strongly believe every refugees… for someone, migrant, they have the choice to migrate to a new country. With refugees, to be like forced to leave their country of origin, leave everything behind from possession to memory to family members, it's not that easy.

We sometimes take things for granted, but it's not easy for refugee, and as I said, being a migrant myself, I can't relate to that even though the decision was mine. But however, leaving everything behind wasn't easy and to really come to a new country with no language and to try to learn the language, you might or might not have university degree or a trade. To go through the process, it might take time. So that was the original purpose of establishing (SERVICE NAME). And then, as I said, during the Gulf War, we've been asked to work with the newly arrived refuges from Arabic speaking, mainly from Kuwait, especially the [indistinct 3.25] and then, as I said, from Iraq. [redacted]

OK. The service that we provide, we provide like a variety of services. We provide, of course, the core of our work is settlement and that the settlement programme we provide case work, case management through one-to-one, through scheduled appointment, a proper intake assessment to identify the needs of a client and through that, whether it is issues related to legal, health, financial, migration assistance. We have registered migration agent, we have three, four actually, on our team. Our workers, we have a policy, internal policy within the organisation, we employ people, they have to be bilingual, bicultural, however they have to have a qualification and the skills to support people coming through our doors.

Our approach is a little bit different since we started. We do a lot of outreach, even despite the size of our organisation that we are really spread very well across. We provide outreach at AEMP, Adult Migrant Education Programme, across the northern metropolitan region. So some of our staff are outposted at (NAME OF LOCATION) TAFE, for example, three days a week at Melbourne Polytechnic across all sites, whether (NAME OF LOCATIONs), depends on the needs, at least one day per week. And Meadow Heights Learning Shop, some of registered training organisations, some of employment agency. So wherever the needs we feel because in order to provide quality services to our community, because sometimes they don't, they newly arrived don't have the need for transport or to get to you. So we are there responding to their, whatever issues, identified issues, in, within their reach. And also we work with the language schools at schools, we work very closely with the schools. Primary, secondary schools, and mainly the schools that they have the English as additional language or a foreign or newly arrived migrant or people who have language barriers.

So through that, as I said, we do the one-to-one, whether through case work or case management. If people need further assistance, like counselling, we have on our team qualified social workers, we have about four. We have family therapists, we have youth workers. So we refer them for internal. We tend, we are well-known by working hand-in-hand and in partnership with all mainstream service providers because we see really, really the need for such, for such… first, to really encourage and educate our, the people that come through (SERVICE NAME) about what's there available to them, encourage them to access. We go a step further, when we try to refer them to services we tend to follow up with that service whether they've been to that service and if they didn't why, follow up. Not that, OK, we refer, tick the box. No. We really feel that we have the moral obligation, before the obligation of whatever funding body request from us, and we do a lot of group work. We do conversation in English because we get a lot feedback that even they go to the language, to the English programmes, being the majority of or sometimes the whole class from Arabic speaking background, they tend to communicate mainly in Arabic, so they are not… and they are keen to practice.

So we do that through not only like through disseminating some of the information that are relevant to them, as I said, whether legal, housing, health, social, recreational, getting to know Australia better, cultural, our political systems, our voting systems, and as well as we have some peer support group to different age groups. And we run specific programmes targeting at, encourage special, encouraging women that they've never driven in their life to get their Ls. So we have a really a defined schedule programme like… and to get their Ls we take them through the computer literacy and to go to Vic Road and sit their test and pass it. Then we apply to Vic Road and we were successful and thankful to Vic Road to support that programme by providing some driving sessions to the participant. We also provide, this is, as I said, specialised programme whether to address healthy relationship, to address intergenerational gap, to address parenting programme, to address the citizenship when they apply to citizenship and we run the citizenship course. So we do a lot. So I don't want to… yes.

Interviewer: That's alright. And I think you'll get a chance to maybe tell a bit more of the specifics of those programmes in the second part of the interview. So the following questions relate to how migrants adjust to Australian culture and society and the kinds of issues and challenges new migrants are facing. So can you tell us about your understanding of how migrants that you work with understand Australian culture and society and maybe what challenges or what, yeah, what challenges they may face as well?

Respondent: It is a very, very big question. And…

Interviewer: Yes, it is, it's very open.

Respondent: Yeah, and very open and really, really they face a lot, a lot of challenges. From renting a house, it's a big challenge because they don't have a record, they don't have in most cases someone to sponsor them because they are on Centrelink income. So this is the basic. Enrolling their children into school, they don't know the system, they don't know which school they should be… they get the myth within the community, some of them for example, the Christian, they don't want to send their kids to the public schools because they believe that they won't get the proper education. They tend to send them to Catholic school and with that it puts, impose financial difficulties on them. And trying to educate them about the education system, for example when it comes to the teenagers, when they are here, and especially, especially that critical age, whether they are 17/18 years of age, coming here with no English background, some of them, and being interrupted with schooling for the last two/three years. For someone 17/18, or 16 to be put with other kids that are much younger than them, of course they don't feel that they belong, they don't feel that they can achieve much, so they leave school. And here they are trapped because the parents are putting high expectation on the children. They're always sort of telling

them off because we made that decision because of you, we want you to have a better future, we want you to be educated, we want you to be highly achievers. So really, they feel that they… and yet, they go, even with people where like… especially in people thriving to achieve, they go to the language centre, even if they have a high goal or aim to… they are told by the school, whether the teacher or the schools, don't put your hope high. And we've been told that again and again. You might be good to do this or to do that and mainly sort of refer them to VCAL or some of the apprentice or to go and find a job in retail, that sort. So they feel that really, really they are not valued as a human being. They are thankful to Australian government that they gave them the opportunity but why they get humiliated when they get to access services. So… and of course it impacts on their mental health. And again, mental health is an issue that is really taken loosely by the majority of service providers.

Interviewer: What do you mean loosely? As in not concerned about it?

Respondent: Not concerned or haven't been addressed because I believe every refugee need, to a certain extent, need to talk to someone about… because they've been through an experience and so, and they've been traumatised. But again, with the eligibility criteria to certain funded organisation to provide the torture and trauma counselling is like, we come across it, we have to clarify with the, that particular client, being exposed to torture and trauma. And I said, every single refugee is being exposed. The fact that they make that claim being accepted so due to being exposed either they lost someone dearly or their village been bombarded or… so yes. So they feel like, again, and because sometimes they… and I'm not saying about the stakeholders or the service providers that unfortunately due to the amount of funding we receive in most of the cases they don't support, like qualify, the people to be able to identify those issues at the beginning.

Interviewer: Yeah, OK. And so it hasn't been…

Respondent: So really what would happen, it will be sort of untouched until it is too late then it comes up to the surface where they need more work on it to address, whether, as I said, family relationship, family violence, mental health. So yes.

Interviewer: OK. And so do you see that new migrants have the chance to practice their own cultural values and practices?

Respondent: They do to a certain extent. As I said, while we're working with them they are overwhelmed with the really necessity of things to do, to have a roof over their head, to put their children into schools, to get themselves into English classes. Some of them really, really are forced to work because what they get, it's not enough to live on. So it's a combination. So really they are not in a state where relax, settle, and they look at whether that social inclusion and harmony to be practiced,

even within their own group let alone with the community or the society in general.

Interviewer: Alright. So these questions relate to migrant's sense of belonging and inclusion in Australian society. So can you tell us about the programmes or supports that you offer to help create and enhance migrant's sense of belonging and cultural inclusion?

Respondent: As I said, we… when they come here, they come with focusing on what they need. And because our services are voluntary, of course we do a bit of that when we work in partnership with other service providers. And that way to introduce them to other services, encourage them to participate at different location. We encourage a lot to participate in the local government activities, whether they have events, festivals, celebration of whatever kind. And in order for us to ensure that they do participate, workers from our team will go with them and attend with them and sometimes we have a stall and we encourage volunteers from our group, from our client, to be part of that and so they are encouraged to participate, to see a different… and we tend to do a lot of, I think again, it depend on the resources we have, but we don't stop at that, we always like explore options of working in partnership.

Like for example, we work very closely with the police where we invite them to get a few members from the police force to go on a picnic with a full bus of our people as a families, where mother, father, younger children first to really, really provide that comfort environment for them to connect with the police, to feel that the police here are different than back home because they have that really barriers and it's scary because they look at police as authority only back home, they cannot talk to them. So when they go on a picnic and feel that, oh, we get the comment like, "oh my God, I've never felt that… it's wonderful, there are people like us." So that emphasis, really, really encouraging that to feel that they belong, and not only from one end but to have it with both. Like for the police to understand them and for the community to be linked and feeling that connection.

And we do with other, as I said, with other… like whether it was the school or where the school staff will go on an outing with our staff. We ran a lot of a project, as I said, in partnership with other service delivery because to encourage them and to be the role model for our targeted group that it is important to work with everyone. And we try as much as we can to expose them to like, for example, any activities at the library, at the leisure centre, at the local library, local leisure centre, or local council, we tend to encourage them to participate and take part of it.

Interviewer: Great. So can you tell us about the types of programmes that are currently being implemented here to provide new migrants social support or health and wellbeing?

Respondent: We have a lot and, as I said, peer support group, it is the key of our work because through that we try to achieve many objectives. First, like conversation in English. Second, dissemination of information. Third, to encourage them to access and educate them to access other services, taking places to visit. Like, for example, utilising public transport, take them to say the museum in the city, and that way for them to learn where the younger people, they would be the lead to take their family or for the parents that they will encourage to take their family members, their relatives. This is from the social and recreation and education, and health, it is a key to our programme. We provide a lot of health problems, health topics and mainly we focus on prevention. For example, we work very closely with the Cancer Council Victoria, we run a lot of a project from breast cancer to bowel cancer to preventing all type of cancer or understanding what services that they can get, what tests should be done, for example, with bowel cancer to educate the community how to order the kit and order the kit, do the test, send that, follow up. And like it is fortune, unfortunate to get like two participants have been, the result has been positive, so to follow it up and prevent that.

We work with Peter Mac where we took a group of our people to really, really take them and mainly a client with either a relative who has cancer or how to access all the services that are available at Peter Mac. And so we take groups to, for example, DPV, the Dianella and Lower Plenty community health services in Broadmeadows to have a session. And the reason we really focus on the business visits and the site visits because to encourage them first to show them how to get there, to really organise a session with a dietician, paediatrician, whatever… depend on the group of people we're taking. But the whole purpose is really, really to show them. We take them to Centrelink, to the court, like family violence, we take them to the court, Magistrate Court in (NAME OF LOCATION). They then ask question, have that interaction. And to really see that the people here are helpful if you know how to navigate your system and to have help. We always encourage that everyone of us should have a voice and how to air your voice in a nice matter to get what, to where you want to be.

Interviewer: Great. So can you tell us about any programmes that you provide or offer that help with financial literacy, income generation, or managing money?

Respondent: Yes. We have for years we've been running like financial programmes in partnership with [indistinct 27.54], MoneyMinded. However, last year at the beginning of the year we got all our, like six of our workers, being trained to deliver that due to the needs, whether to deliver it to one-to-one or to groups. So… and mainly focusing on budgeting, how to open a bank account, how to really look after your finance, how to put priority in place, how to pay online, how to pay direct debit. Yeah, all of that literacy.

Interviewer: And what are some of the key financial challenges that new migrants are facing that you work with?

Respondent: What do you mean?

Interviewer: What are the key financial challenges they have in coming to Australia? Is it, say, is there issues with generating income? Are there issues with managing household budgets or…?

Respondent: Of course. It's not enough when you have… like some of our client that come in was like paying almost 60% of their income into rent. And especially I'm talking where either to single people, like siblings, or a couple on their own or individual. So with the family they have a little bit more because as a whole family they can combine their income, but yes, we find they're struggling to really, really pay the rent. And in some instance they are left with little, a little to pay the bills and the food and sometimes the dietary can be, be sacrificed. And that's where you find that they easily can get sick or yeah.

Interviewer: Any culturally specific issues or challenges? You know, sending money back home or gender imbalances?

Respondent: Sometimes they do. Sometimes, sometimes they do. But, as I said, first they have to look after themselves here and unfortunately, if you tell me, I've been with the organisation for over 25 years, when I first started it was the pattern where they really, really want to look after their relatives back home and now you probably come across but the percentage is very low because they don't have enough to really fulfill their own needs let alone to send money. Yeah.

Interviewer: So do you… can you tell us about any programmes or support that you provide for your clients to do for when they're facing legal issues?

Respondent: We work, again, we work very closely with community legal centres and with Victorian Legal Aid. We've run a lot of project for our young people with Youth Law. And yes, and we have… we not only refer but really, really we follow up and we advocate on behalf of our client to get the support needed.

Interviewer: Excellent. And you also have migration, registered migration agents here as well from migration services?

Respondent: Yes, yes. Yes.

Interviewer: Excellent. And, in your opinion, what's the kind of level, what's the level of awareness or understanding of the Australian legal system for new migrants, of course?

Respondent: For new migrants?

Interviewer: Yeah.

Respondent: It is bare, bare minimum. Yeah.

Interviewer: And are they facing challenges because of that?

Respondent: Of course. For example, infringement almost every day. Every day we get a person or a client with that issue. They don't know how to read the signs properly or dropping off kids at school where they park their car in non-standing… or yeah. So… and I believe, yeah, we do a lot where we invite guest speakers from the council to really educate them about that. For example, with Myki we run a lot of information session how to use Myki, what the implication if you don't have a Myki and if you haven't topped up with money. So we, like all the prevention, we strongly believe in prevention because we believe like knowledge is equal to power. And when they are knowledgeable about things they sort of are aware how to deal with matters.

Interviewer: Great. And so these next questions are about movement of your clients from one place to another. So what are some of the key reasons why people you work with might move around the city of Melbourne from one area to other areas?

Respondent: We don't, we don't see that much move. We see it interstate, like especially from Queensland, like a lot of people, they do settle… like or they arrive to Queensland, they stay for 28 days, then they are moved here. The main reason, even if [indistinct 33.46] happen… when they are here, they tend to go where the community is. So that's where you really with people from Arabic speaking background, the northern metropolitan region is the region for… we have in the south-eastern region pockets, but the majority are in the northern metropolitan region, that's why most of our work's in the (NAME OF LOCATIONS)

Interviewer: We've also, I've also heard from other providers that they're seeing some people moving further out due to rental prices. So say if they're…

Respondent: With that, even, look… and it's not like cheap. Like for example, I'll give you an example about the Iraqi when they first started to come, like they built [indistinct 34.57] Park, like the majority of them, and they really work hard, the whole family after hours, before hours, and the cleaning, whatever. And this is one thing, always financial implication that they tend to work really for cash and that would, really they are subject exploitation because they get paid little money, but to them it is something extra on top of what they get from Centrelink. We do a lot of talks and educate them but we felt like you can't help it because the needs are there and they will do anything to really make sure to meet their ends at the end of the day.

Interviewer: I think it's a pretty common thing, not just for new migrants.

Respondent: But it is really, really sad to see. And sometimes for people from the same community, it is sad, but what can you do when they don't want to talk about it and sometimes they regret telling the workers? And yes.

Interviewer: Alright. So could you tell us about any services that you offer to new migrants that are about education or literacy?

Respondent: Yes, we have, as I said, we provide a lot of, most of our programme, it is about education, about literacy, it is the conversation in English and to help with… we have programme mainly focusing on employment from resume writing, how to search online, if there are adult, we take them through a computer skills training at the library, local library. So how to present yourself, like mock interviews. If we have, sometimes we link them through our network to industry visits. For example, whether it is… and we know that might have application or opportunities to have a job. So we do that.

Interviewer: Excellent. And do you see that they're… are there employment opportunities for new migrants?

Respondent: Look, I must say, and some of them are doing very well, but unfortunately, even with our people, like the younger people that are graduating from university struggling to get work, let alone with some people who is limited English, not have their degree recognised, and sometimes they don't have the language. Of course, the limitation of the… and to overcome. And there are not too many job. And this is where I always, instead like for the government I advocate on their behalf for the government to provide opportunities. Where are the jobs? Let them provide opportunities and then we can really educate the community or sort of put the target to get trained or to get the skills in that particular area in order to get the work. But some of them, for a job like part-time jobs or casual work, it doesn't pay because they would be losing some of their entitlement. So sometimes, not allowed to really cost for clothes, for petrol, for other, other, not to take that job and stay on Centrelink. So the incentive, it's not there.

I'd rather give the incentive. OK, in the first year, in the first six months, at least let them test how important to work. Whatever you earn, if you are working 20 hours or 15 hours per week, that will be free of tax or it doesn't impact on your income. And so this is one incentive instead of keep them out of work and then having employment agency, and the money will be there also. They will spend more money on someone rather than… an internship, pay them little bit and when they prove themselves they probably might get a job with whatever opportunity provided. So yes, we need to really revisit

our employment/education services and really focus on how we can make it work for the people that we're working with.

Interviewer: Great. And so overall, what do you think are the key challenges migrants would face while adjusting to Australian culture and settling in Australia?

Respondent: I mean…

Interviewer: You've said a lot.

Respondent: We've said a lot. And…

Interviewer: Where do you see the key ones? You probably mentioned them.

Respondent: Yeah. The key one, as I said, language, employment, housing, legal aspect, mental health. That, the really, really, the changing of family structure here, that the head of the… they're coming from a culture where men are seen as the head of the family. They come here and we have seen an increase of mental health among men because they feel that they lost their identity, they are not anymore looked as the bread winner, as head of the family because they are not fulfilling their role. So that shift impacting, impacting on the whole relationships within the family.

Interviewer: Definitely. And finally, what would you like to see as possible solutions to helping or supporting migrants to adjust well in Australia?

Respondent: As I said, about the employment, this is one of the recommendations, just to give them a chance to… and whatever, even if they get to work 10/15 hours per week not to be impacting on their income because then it is an incentive to motivate them to work more to give them that taste of, for example, aged service. I know people from Arabic speaking background, they are not keen, they look at it as, oh, I'm not here to, sorry, clean the dirt of someone. But if they learn that it is a job and it doesn't have to be, sorry, cleaning the shit off someone, but it can be trying to support them, taking them shopping, cooking for them, cleaning for them, whatever. So really, really, and this is the only way when they get like a taste of the job, yeah. And to be provided with opportunity. For example, big industry, to really, really be involved, to be brought to the table, talk to them, what, how you can contribute. Like whatever. Big banks. OK, I'm expecting each banks to take 20 people per year for internship or work experience and you never know, they might start with… again, explore opportunities and help them (?). Yeah.

Interviewer: Great. So this is the end of the interview. Is there anything else that you'd like to add?

Respondent: I believe I spoke a lot and I told you a lot, yeah.

Interviewer: Right. No problem. Well, thanks for your participation. I really appreciate all your time and expertise. So interview end, what's this, 2:57.
